# Spatial Clustering Properties in the Temporal Variation of Suicide Rates/Numbers among Japanese Citizens: A Comprehensive Comparison and Discussion

**DOI:** 10.1371/journal.pone.0127358

**Published:** 2015-07-10

**Authors:** Makoto Tomita, Takafumi Kubota, Fumio Ishioka

**Affiliations:** 1 Clinical Research Center, Tokyo Medical and Dental University, Bunkyo-ward, Tokyo, Japan; 2 School of Management and Information Sciences, Tama University, Tama, Tokyo, Japan; 3 School of Law, Okayama University, Okayama, Okayama, Japan; Medical University of Vienna, AUSTRIA

## Abstract

**Objective:**

The number of suicides in Japan has remained high for many years. To effectively resolve this problem, firm understanding of the statistical data is required. Using a large quantity of wide-ranging data on Japanese citizens, the purpose of this study was to analyze the geographical clustering properties of suicides and how suicide rates have evolved over time, and to observe detailed patterns and trends in a variety of geographic regions.

**Methods:**

Using adjacency data from 2008, the spatial and temporal/spatial clustering structure of geographic statistics on suicides were clarified. Echelon scans were performed to identify regions with the highest-likelihood ratio of suicide as the most likely suicide clusters.

**Results:**

In contrast to results obtained using temporal/spatial analysis, the results of a period-by-period breakdown of evolving suicide rates demonstrated that suicides among men increased particularly rapidly during 1988–1992, 1993–1997, and 1998–2002 in certain cluster regions located near major metropolitan areas. For women, results identified cluster regions near major metropolitan areas in 1993–1997, 1998–2002, and 2003–2007.

**Conclusions:**

For both men and women, the cluster regions identified are located primarily near major metropolitan areas, such as greater Tokyo and Osaka.

## Introduction

In the years up to and including 1997, the number of suicides in Japan was estimated at around 25,000 per year. However, in 1998 the number suddenly jumped to over 30,000 and persisted through 2011 [1]. According to Vital Statistics of Japan from Japan’s Ministry of Health, Labour, and Welfare (MHLW), the 30,827 Japanese people who committed suicide in 2007 (the most recent year for which data were considered in the present study) made that year second only to 2003 for the largest number of suicides in Japanese history. Clearly, suicide has become a major societal problem. According to the World Health Organization, in 2006, Japan’s annual rate of 23.7 suicides per 100,000 people ranked eighth highest in the world; thus, Japan’s suicide rate is extremely high even by international standards, and indeed has been reported to be the highest among the seven leading nations of the world [2]. The promotion of comprehensive suicide-prevention measures has become a pressing national issue, and in October 2006, Japan’s government enacted a “Basic Law on Suicide Countermeasures” (Cabinet Office, Government of Japan, 2008). Japan’s cabinet ministry, following the Basic Law and the Comprehensive Suicide Prevention Principles [2], provides a set of anti-suicide policies to prefectural governors and other local government officials and works to implement suicide prevention measures in various regions around Japan [2]. The MHLW first took up the cause of suicide prevention with the launch of its “Healthy Japan 21” project, an initiative that aims to secure the health of the Japanese population in the 21st century. At that time, the Ministry announced a goal of reducing suicides to fewer than 22,000 per year by 2010 [2]. However, with suicides in 2011 again topping 30,000, this objective was not fulfilled. In an effort to understand regional variations in the suicide rate and to assist in promoting suicide-prevention programs and proposals in the various regions of Japan, the Center for Suicide Prevention, a unit of the National Institute of Mental Health within Japan’s National Center of Neurology and Psychiatry, recently revised its Regional Statistics on Deaths by Suicide among Japanese citizens in Japan [1]. The new statistics include choropleth maps depicting age-adjusted death rates in secondary treatment networks, segregated by gender, for six 5-year intervals (the first interval was 10 years). However, this map does little more than simply indicate higher and lower suicide rates; it does not provide immediate insight into spatial and temporal/spatial clustering.

In a review of the landscape of epidemiologic and spatial statistical suicide data outside Japan, we found that the Australian state of Queensland used circular scans [3] to analyze the geographic clustering and spatial distribution [4, 5] of deaths by suicide between 1999 and 2003, identifying regions of high suicide concentrations. The Tuscany region of Italy performed a spatial analysis of 4,764 cases of death by suicide between 1988 and 2002 and used the Standard Mortality Ratio (SMR) to characterize the results; the findings revealed a significant risk of suicide for men in agricultural villages and regions [6]. Similarly, a number of reports in South America have discussed the extent to which the nature of murders, suicides, and traffic accidents in Brazil are correlated with factors such as geographic area, age, gender, race, and skin color [7].

On the other hand, in the temporal trends, [8] shows that the trends in divorce, marriage, unemployment, gross domestic product (GDP) per capita and alcohol consumption were compared with trends in suicide rates graphically and using time-series analysis. From their graphical analyses, trends in GDP showed a recession for two years (1998–1999) in Japan. Time-series analysis indicated male suicide was attributable to increases in unemployment. Furthermore, they show strong relationships between gender and social factors given by the temporal trends of the graphs.

Various proposals have addressed methods for detecting spatial clustering, including using the perspective of spatial autocorrelation [9] and investigating clustering by scanning small regions within larger geographic areas according to certain fixed rules [10, 11]. Similarly, the detection of clustering properties as defined by [12] and spatial scan statistics [13] have been proposed as methods to investigate geographical clustering of diseases using spatial epidemiology [14].

We used large-scale datasets on Japanese citizens to perform geographic analysis of spatial clustering of suicide. By considering temporal variations, we developed a combined temporal/spatial analysis to understand detailed trends and transitions in various regions. As a result, we were able to evaluate temporal and spatial clustering properties that had gone unnoticed and identified a number of trends in recent years. In particular, we found significant increases in suicides among men in the Tohoku and Kyushu regions of Japan, while suicides among women increased and decreased in the Northern Kanto, Hokuriku, and Hida regions. However, each single temporal/spatial region covered a long time period, creating the possibility that various trends, or indications of significant effects, may have been overlooked. In the present study, we used period-by-period changes in suicide rates to analyze trends for each of the four most recent periods covered by our data, that is, 1988–1992, 1993–1997, 1998–2002, and 2003–2007. Furthermore, using background factors of gender and age categories reveals complex structures can be found from neighboring information since each connected time period covers 5 years. The situation regarding these relationships is very complex and we feel more time is needed to discuss a solution of handling the spatial-age; as such, the spatial-age problem would not be a limitation of this work. In addition, we used gender as a background factor as the above [8].

## Materials and Methods

All deaths identified as suicides according to ICD-9 and ICD-10 were extracted from vital statistical data of Japan [1]. For geographic statistics on deaths by suicide, we used and modified death records from the Vital Statistics of Japan assembled by the MHLW. These data are published at a depository at the National Institute of Mental Health, National Center of Neurology and Psychiatry as “Statistics of Community for the Death from Suicide in 1973 to 2009”. These data were edited, updated, and segregated by municipality and secondary medical zone [15] (See also Appendix-I). In addition, the data for secondary medical zones, including statistics on numbers of suicide deaths, rates of suicide deaths, and comparisons to national averages, were segregated by age category and gender. (The data are for ages 10 and older. Note that the phrase “suicide deaths” here refers to “the number of suicide deaths by Japanese citizens in Japan.”) In the present study, although we were more interested in the absolute number of suicides than relative suicide rates, we chose to consider suicide rates to eliminate the influence of changes in the overall size of the population. We focused on the data from the secondary medical zones discussed above. We subdivided the study interval into six subintervals, labeled: 1973–1982, 1983–1987, 1988–1992, 1993–1997, 1998–2002, and 2003–2007. We performed cluster analyses with particular focus on 1988–1992 through 2003–2007 with special attention paid to the year 1998, in which the number of suicides first exceeded 30,000. In cases where the population served by the data-gathering organization was small, slight changes in the number of suicides can lead to large variations in the suicide rate. Accidental or coincidental fluctuations in numbers of suicides can thus cause instability in the numerical values of suicide rates. For this reason, there are many cases in which the population served by a data-gathering organization is so small that comparisons with calculated suicide counts would be inappropriate. To address this difficulty, we attempted to mitigate the impact of accidental fluctuations due to small population sizes by using admissible guiding data (namely, the trend in total national suicide deaths) to estimate the expected data observed by data-gathering organizations [2]. We used the following equation to determine age-adjusted death rates:

$$\begin{array}{ccc} & & \text{Age-adjusted}\;\text{death}\;\text{rate}\;\text{(Bayesian}\;\text{estimate)}\\ & & \;=\sum_{i}(\frac{\text{The}\;\text{number}\;\text{of}\;\text{suicides}\;\text{for}\;\text{age}\;\text{category}\;\text{reported}\;\text{by}\;\text{data-gathering}\;\text{organization}+\hat{\beta_{i}}}{\text{Population}\;\text{for}\;\text{age}\;\text{category}\;\text{reported}\;\text{by}\;\text{data-gathering}\;\text{organization}+\hat{\alpha_{i}}}\\ & & \;\times \frac{\text{Population}\;\text{for}\;\text{age}\;\text{category}\;\text{reported}\;\text{by}\;\text{standards}\;\text{organization}}{\text{Population}\;\text{reported}\;\text{by}\;\text{standards}\;\text{organization}}) \end{array}$$

where, the index *i* runs over age categories and $\hat{\alpha_{i}}$ and $\hat{\beta_{i}}$ are parameters in the Γ -distribution selected to represent the prior distribution of nationwide suicide deaths. These parameters are determined by a moment estimation in which the weights are taken as the populations of the secondary medical zones. Details on the approach to calculate the estimators for *α* and *β* are provided by [14]. Their method was based on the standard mortality ratio (SMR) in which the expected value and variance of the SMR of the prior distribution in a given municipality are equal to the expected value and variance of the SMR in the larger region (the secondary medical zones) containing that municipality. In contrast, the present study was based on the suicide rate in the larger surrounding region; therefore, the expected value and variance of the suicide rate in the secondary medical zones were equal that of the nationwide suicide rate. Thus, we used the estimated value discussed above. Because we were unable to acquire age-class-specific segregated data on the population of Japanese citizens within various secondary medical zones and municipalities, for convenience, we instead used the total Japanese population.

We next analyzed data on the number of suicides obtained for each region, segregated by secondary medical zones, to investigate whether clusters of suicides (regions with a statistically significant high number of suicides) were present. We used spatial scan statistics [13] to detect clusters. Spatial scan statistics is a technique for detecting whether an event that took place at a particular location within a particular region was random (coincidental); it is a likelihood-ratio statistic that identifies groups of regions with statistically significant excesses. Let G denote the full set of 348 secondary medical zones we considered, and let Z denote a single one of these regions or a collection of multiple secondary medical zones aggregated together. Suppose the probability for the number of suicides among the full population is *p*_1_ within region Z and *p*_2_ outside of that region. Assume *p*_1_ and *p*_2_ are mutually independent. Let the null hypothesis be *H*_0_: *p*_1_ = *p*_2_ = *p*, while the alternative hypothesis is *H*_1_: *p*_1_ > *p*_2_. Assuming that the ratios of suicide to the total population in various regions obey mutually independent Poisson distributions, we used a spatial scan statistical model based on the Poisson distribution [3, 13]. Within a given region Z, let the age-adjusted expected number of deaths be e(Z) and let the actual number of suicides be c(Z). Then the likelihood-ratio statistic takes the form as follows.

$$\begin{array}{c} \lambda \left(Z\right)=\{\begin{array}{cc} {\left(\frac{c(Z)}{e(Z)}\right)}^{c(Z)}{\left(\frac{c(Z^{c})}{e(Z^{c})}\right)}^{c(Z^{c})}, & \text{if}\;c(Z)>e(Z)\\ 1, & \text{otherwise} \end{array} \end{array}$$

Regions Z, for which λ(*Z*) is at maximum, are identified as cluster regions. To determine whether the maximum likelihood ratio λ(*Z*) was statistically significant, we considered the distribution of λ(*Z*) expected under the null hypothesis. However, because it is difficult to obtain analytical solutions, we computed the value of p based on a distribution estimated by the Monte-Carlo method [16]. To detect high-likelihood cluster regions, a decision procedure (a scanning method) for identifying regions Z for which the value λ(*Z*) is large, is important. However, in Kulldorff’s method, the data are taken to lie within a circle centered at the center of the region in question; hence, this method is capable only of detecting cluster regions that happen to be shaped like circles. A number of scanning methods, including upper level set scanning [17], simulated annealing scanning, and flexible scanning [18], have been proposed to address this difficulty; nonetheless, at present it remains challenging to analyze the sort of highly specific, large-scale data we were considering in this study. For this reason, we chose to use echelon analysis [19, 20] as a scanning technique (echelon scanning). Echelon analysis is a technique for systematically and objectively revealing the topologic structure of spatial data by subdividing locations in space according to the heights of data points on a surface. As depicted in the left portion of S1 Fig, suppose we have nine regions assigned labels ranging from A to I, and suppose each region has a value for some quantity of h. In this case we can divide space into seven topological classes (“echelons”) based on the values of h and on the position data (adjacency data) for the various regions (S1 Fig, center). This reveals that our data set contains four peaks and exhibits a stratification structure consisting of three foundations. This structure of the data set may be expressed in the form of the echelon dendrogram shown in the right portion of S1 Fig. The procedure of cluster detection is performed as follows [20].

1. Scan the region and add it to Z from the upper to the bottom echelon based on the hierarchical structure of the dendrogram.
2. Detect the cluster region that has the maximum log likelihood ratio statistic.
3. Estimate the p-value using Monte Carlo simulation under the distribution of the null hypothesis.

The result is that, by using echelon analysis to determine the topologic structure of our spatial data and then considering the stratification structure, it is possible to use the region-scanning method outlined below to identify cluster regions in large-scale spatial datasets.

The data on secondary medical zones considered in this study were obtained with reference to the 2008 Editorial Committee of the National Reference Guide to Japanese Municipalities and adjacency data were constructed from those data. For regions abutting ocean coasts, underwater tunnels, large connecting bridges, or other primary sea routes were used to construct adjacency data. Of 354 secondary medical zones nationwide, we excluded one region in Kagoshima Prefecture (south of and up to Amami Oshima) and five regions in Okinawa Prefecture, yielding a total of 348 considered regions. We excluded Okinawa from an analysis of all Japanese regional governments on the ground that it was extremely far removed from other regions. In performing echelon analyses, we “fixed” the data for all secondary medical zones for each period to the 2008 baseline, as discussed above. To identify prominent trends in the period-by-period evolution of the data, for each of the 348 secondary medical zones, we considered the per-period increase/decrease rates from the age-adjusted death rate in one period to the age-adjusted death rate in the next period, and used this measure to assess the ways in which clustering properties varied from period-to-period. Here, the “per-period increase/decrease rates” (to which we refer below in brief as the “increase/decrease rates”) were determined by dividing the age-adjusted death rate for the next period by the age-adjusted death rate for the target period. The region Z for which the echelon scan yielded the maximum likelihood ratio was termed the “most likely cluster,” while the region with the second-greatest likelihood ratio was the “secondary cluster.” We imposed an upper limit of 35 regions (approximately one-tenth of the total number of regions) that could be identified as cluster regions. The reason being that, because spatial-scan statistics is a model that attempts to maximize the likelihood ratio, it has a strong tendency to identify an excessively large number of regions as clusters; an upper limit of 35 regions was designed to mitigate this effect.

To compare results, we arranged our six periods in chronological order and performed temporal/spatial echelon analysis. More specifically, we grouped all six periods together to yield a total of 348 × 6 = 2,088 regions under consideration for each gender (data for men and women were considered separately). We proceeded similarly by using the echelon structure of this set of regions to identify clusters. We imposed an upper limit of 200 (approximately one-tenth of the total number of regions of 2,088) on the number of regions that could be grouped together into any one cluster. Using this approach, we proceeded to identify the most likely and secondary clusters.

## Results

S1 Table presents the clusters obtained by applying echelon scanning within each period, as well as the results of 999 Monte-Carlo detection steps applied to those clusters.

S2 Fig is an echelon dendrogram for a spatial analysis using the change in suicide rates among men from 1988–1992 to 1993–1997. The vertical axis indicates the change in suicide rate. Conducting an echelon scan of this dendrogram identified the clusters indicated in red near the center of the plot as the most likely clusters, that is, the clusters for which the likelihood ratio was greatest. The clusters shown in orange toward the right side of the plot are the secondary clusters (i.e., those with the second-highest statistics). These clusters correspond respectively to the regions indicated in red and in orange in S3 Fig. Next, considering changes from 1993–1997 to 1998–2002, and from 1998–2002 to 2003–2007, we obtained the most likely and secondary clusters plotted in S4 and S5 Figs. In analyzing the evolution in suicide rates from 1988–1992 to 1993–1997, and from 1993–1997 to 1998–2002, we found that most likely clusters tended to lie in the greater Tokyo or Osaka areas, while secondary clusters were identified in parts of Kyushu, northern Tohoku, and Donan. In contrast, analyzing the evolution from 1998–2002 to 2003–2007 did not detect cluster regions in major metropolitan areas such as the Tokyo or Osaka areas. For the spatial analysis of changing suicide rates among women, the most likely and secondary clusters for the transitions from one period to next are shown in S6, S7 and S8 Figs respectively. For the evolution from 1998–1992 to 1993–1997, and the evolution from 1993–1997 to 1998–2002, the most likely clusters were located in a vicinity including part of the Osaka area as well as in the Tokyo area. Secondary clusters for the evolution from 1993–1997 to 1998–2002 were located in Donan, parts of Do-ou, and northern Tohoku. For the evolution from 1998–2002 to 2003–2007, clusters were located in Hokuriku and in parts of the greater Osaka area.

We also conducted an echelon temporal/spatial analysis of the six periods arranged in chronological order. More specifically, we considered all six periods together, yielding a total of 348 × 6 = 2,088 regions under consideration for each gender (data for men and women were considered separately). We proceeded similarly by using the echelon structure of this set of regions to identify clusters. Using this approach, we proceeded to identify the most likely and secondary clusters (The statistical quantity log and the p value for each of these cases are listed in S1 Table). Among the six periods studied, 1998–2002 through 2003–2007, were found to be particularly noteworthy for their especially rapid increases in suicides among both men and women. Performing an echelon temporal/spatial analysis of suicide data for men over all six periods arranged in chronologic order revealed 200 regions deemed most likely clusters and 197 regions deemed secondary clusters. The most likely and secondary clusters identified by analyzing each period are shown in S9, S10, S11 and S12 Figs, respectively. Although a glance at these plots would seem to indicate the presence of non-adjacent regions among the cluster regions, these regions are in fact temporally adjacent. As these Figs. clearly indicate, during the most recent evolution from 1998–2002 to 2003–2007, the most likely clusters were primarily located in the Tohoku region while the secondary clusters spread out from a nexus located in the Kyushu region. Similarly, performing an echelon temporal/spatial analysis of suicide data for women over all six periods arranged in chronological order revealed 129 regions deemed most likely clusters and 112 regions deemed secondary clusters. The most likely and secondary clusters identified by analyzing the evolution from one period to the next are shown in S13, S14, S15 and S16 Figs, respectively. Again, although these plots seemed to indicate the presence of non-adjacent regions among the cluster regions, these regions were in fact adjacent in the time domain. These findings indicated that regions identified as most likely clusters exhibited significant variation in the northern Kanto and Hokuriku regions and near Hida, while regions identified as secondary clusters exhibited significant variation in the Tohoku region. However, the data for women did not reveal the striking increase in suicide rates observed for men in recent years (1998–2002 and 2003–2007). In contrast to the results of spatial-clustering analysis conducted using period-to-period changes in suicide rates, this analysis identified no cluster regions in major metropolitan areas such as the greater Tokyo or Osaka areas during any of the periods studied.

Furthermore, we focused on suicide numbers of males in 1993–1997, 1998–2002 and 2003–2007 only in the Kanto region. Their data were counted in 350 municipalities, that is, cities, wards, towns or villages, in the Kanto region. We then imposed an upper limit of 100 regions that could be identified as cluster regions. The most likely clusters, secondary clusters, and significant clusters identified by analyzing each period, 1993–1997, 1998–2002 and 2003–2007, are shown in S17, S18 and S19 Figs, respectively.

## Discussion

In the present study, we considered gender-specific suicide data for six time periods, focusing in particular on four periods exhibiting rapid growth in the number of suicides; namely, 1988–1992, 1993–1997, 1998–2002 and 2003–2007, spanning a total interval of 20 years. We have presented the analyses results of the period-by-period evolution in suicide rates over the studied interval.

We used choropleth maps [15] to present results for age-adjusted death rates in each of the four periods comprising the 20-year window of primary interest. According to these results, suicide rates among men appear to have increased rapidly during the 1998–2002 and 2003–2007; however, some secondary medical zones in small non-adjacent regions also exhibited large values, and in 2003–2007, large numbers were observed for almost all regions. Nonetheless, it is clearly evident that suicide rates in 1998–2002 and 2003–2007 differ significantly from those in 1988–1992 and 1993–1997. The data on suicide rates for women indicate lower numbers in 1993–1997 relative to the other periods; however, no other clear trends are immediately visible in the data.

The results of our temporal/spatial analysis revealed that the number of suicides among men grew significantly as time progressed through the term of our study; in particular, 1998–2002 and 2003–2007 saw heavy increases in suicides among men, particularly in the Tohoku and Kyushu regions, thus underscoring the grave severity of Japan’s rapidly growing suicide problem. Data on suicides among women revealed significant variation in the most likely clusters, primarily in the regions of North Kanto and Hokuriku and near Hida, with the secondary clusters in the Tohoku area. These findings allow a clear understanding of the temporal evolution of the phenomena in question.

Inspection of the most likely and the secondary clusters for 1988–1992, 1993–1997, 1998–2002 and 2003–2007, appears to reveal many non-adjacent regions among the clusters; however, these regions are in fact temporally adjacent. The results of our spatial analysis-based on a period-by-period breakdown of changing suicide rates reveal that, for men, regions near major metropolitan areas were the major clusters for suicides, particularly in 1988–1992, 1993–1997 and 1998–2002, which saw especially rapid increases in suicides among men. For women, regions near major metropolitan areas were detected as clusters in 1993–1997, 1998–2002 and 2003–2007. Thus we see that, for both men and women, the regions identified as clusters of high suicide rates were located in major metropolitan areas such as the greater Tokyo or Osaka areas; however, the data for men and for women exhibited distinct patterns and trends. Nonetheless, our study based on temporal/spatial analysis failed entirely to identify major metropolitan areas as clusters of high suicide rates.

Indeed, if we consider data for men (the population among which suicide is more common) and look at the 1998–2002 and 2003–2007 periods, which are believed to postdate the major increase in the number of suicides, then even the results of our spatial analysis based on period-by-period breakdown of evolving suicide rates fails to identify major metropolitan areas as high-suicide clusters. In view of this apparent puzzle, the authors wonder if changing suicide rates in major metropolitan areas before the onset of the ultra-high-suicide-rate era (1998–2002 period and thereafter, starting with the year 1998 when the annual suicide rate first topped 30,000) might not be exerting an influence on the increasing numbers of suicides observed today. In other words, perhaps large metropolitan areas, which would be considered cluster areas based on unadorned temporal and spatial data on suicide death rates, were being masked by other high-suicide areas and thus not properly identified as clusters. One way to interpret the findings of the present study is that we have used data on changes in suicide rates to identify these undetected clusters. Furthermore, we have found the most likely cluster located in central areas of major metropolitan cities on spatial-temporal analyses in only the Kanto region. This cluster also included many more areas in 1998–2002 and 2003–2007 compared with 1988–1992. These phenomena appear to support our above-stated hypothesis.

A limitation of the analyses reported in the present study is that we focused attention only on the temporal and spatial clustering properties of the number of suicides by gender. The problem of identifying suicide risk factors among the various regional characteristics (such as age, society, economy, labor, culture, health, medical care, social-welfare programs, and social security) is a topic for future research.

A previous investigation reported data on the relationship between factors such as suicide methods, marital status and spousal relationships, and occupational status. Other analyses have incorporated related societal factors, such as the total unemployment rate and those dealing with the loss of a loved one, into spatial correlation models [1]. By conducting a thorough and comprehensive analysis incorporating societal and environmental factors, and carefully investigating the underlying phenomena, we hope to have made some contribution to the establishment of a strategy for addressing the presently dire state of Japan’s suicide problem.

## Supporting Information

S1 Fig Steps between regional data and Echelon dendrogram. (EPS)

S2 Fig Echelon dendrogram of increase/decrease rate between 1988–1992 and 1993–1997 for males. (EPS)

S3 Fig Most likely and secondary cluster of increase/decrease rate between 1988–1992 and 1993–1997 for males. (EPS)

S4 Fig Most likely and secondary cluster of increase/decrease rate between 1993–1997 and 1998–2002 for males. (EPS)

S5 Fig Most likely and secondary cluster of increase/decrease rate between 1998–2002 and 2003–2007 for males. (EPS)

S6 Fig Most likely and secondary cluster of increase/decrease rate between 1988–1992 and 1993–1997 for females. (EPS)

S7 Fig Most likely and secondary cluster of increase/decrease rate between 1993–1997 and 1998–2002 for females. (EPS)

S8 Fig Most likely and secondary cluster of increase/decrease rate between 1998–2002 and 2003–2007 for females. (EPS)

S9 Fig Most likely and secondary cluster of 1988–1992 by spatial-temporal study for males. (EPS)

S10 Fig Most likely and secondary cluster of 1993–1997 by spatial-temporal study for males. (EPS)

S11 Fig Most likely and secondary cluster of 1998–2002 by spatial-temporal study for males. (EPS)

S12 Fig Most likely and secondary cluster of 2003–2007 by spatial-temporal study for males. (EPS)

S13 Fig Most likely and secondary cluster of 1988–1992 by spatial-temporal study for females. (EPS)

S14 Fig Most likely and secondary cluster of 1993–1997 by spatial-temporal study for females. (EPS)

S15 Fig Most likely and secondary cluster of 1998–2002 by spatial-temporal study for females. (EPS)

S16 Fig Most likely and secondary cluster of 2003–2007 by spatial-temporal study for females. (EPS)

S17 Fig Most likely and secondary cluster of 1993–1997 in Kanto regions by spatial-temporal study for males, where log-likelihood ratio (LLR) notation is denoted as the LLR statistic for the cluster. (EPS)

S18 Fig Most likely and secondary cluster of 1998–2002 in Kanto regions by spatial-temporal study for males, where log-likelihood ratio (LLR) notation is denoted as the LLR statistic for the cluster. (EPS)

S19 Fig Most likely and secondary cluster of 2003–2007 in Kanto regions by spatial-temporal study for males, where log-likelihood ratio (LLR) notation is denoted as the LLR statistic for the cluster. (EPS)

S1 Table Statistics log λ and *p* values for each cluster. (TEX)

S1 Data Appendix-I. Our raw data are counted by each secondary medical zone.
(PDF)
