# Supplementary material for: Spatial Clustering Properties in the Temporal Variation of Suicide Rates/Numbers among Japanese Citizens: A Comprehensive Comparison and Discussion
Source: PLoS One. 2015 Jul 10;10(7):e0127358. doi: 10.1371/journal.pone.0127358 (PMC4498741; doi:10.1371/journal.pone.0127358)
Supplement: S1 Data — Our raw data are counted by each secondary medical zone. (PDF) [file pone.0127358.s021.pdf]

Appendix-I

Our raw data are counted by each secondary medical zone.
And we used only "Total" columns in our study.

Column "Num per year": the number of suicide as an average by year.
Column "Rate /100,000": the death rate per 100,000 persons.
Column "x100": the ratio (%) for an average of all area.

Secondary Medical Zone ID: 001

	Male						Female					
	Suicide			Suicide			Suicide			Suicide		
	Num per year	Rate /100,000	×100	Num per year	Rate /100,000	×100	Num per year	Rate /100,000	×100	Num per year	Rate /100,000	×100
	Total (>10 y/o)			45–54			Total (>10 y/o)			45–54		
1983–1987	71.6	29.3	86.9	20.6	53.5	105.8	38.6	13.6	77.2	7.2	18.4	107.0
1988–1992	49.2	25.0	101.2	9.4	31.8	92.7	32.6	12.6	98.3	4.2	13.1	89.4
1993–1997	51.8	25.7	104.6	15.8	46.3	126.5	26.8	10.4	95.7	4.8	13.0	100.5
1998–2002	88.0	42.1	120.2	20.2	60.7	108.5	31.2	12.0	94.7	5.6	15.2	100.8
2003–2007	93.8	46.2	129.7	22.8	78.8	140.9	34.0	13.8	107.2	5.8	16.7	122.1
2008–2012	80.0	41.5	123.1	15.8	63.7	131.0	33.0	14.3	107.7	5.2	17.8	119.1
	15–24			55–64			15–24			55–64		
1983–1987	4.8	9.9	70.7	11.0	37.6	88.4	3.2	7.2	106.0	5.4	16.9	85.7
1988–1992	2.8	9.1	92.0	9.6	35.0	98.5	1.4	4.6	87.6	6.0	18.4	103.3
1993–1997	3.0	10.8	95.1	12.2	44.1	109.5	1.0	4.1	78.9	5.8	17.3	109.1
1998–2002	4.4	17.0	107.3	20.0	71.2	112.1	1.6	6.7	95.1	7.4	21.1	112.3
2003–2007	3.6	17.3	99.1	25.0	78.3	135.6	1.6	8.3	92.2	6.4	17.4	104.0
2008–2012	4.6	24.2	114.3	20.4	61.0	125.6	2.6	12.8	132.0	6.0	15.9	106.4
	25–34			65–74			25–34			65–74		
1983–1987	10.4	26.7	104.3	6.2	34.0	77.6	4.4	11.6	107.5	6.0	23.5	75.1
1988–1992	7.0	25.2	132.0	5.8	34.6	98.1	2.8	9.2	105.8	6.8	28.4	108.7
1993–1997	4.6	19.2	98.4	7.0	34.2	107.2	1.0	5.6	66.7	5.8	21.4	109.6
1998–2002	12.0	41.0	151.8	12.6	52.3	115.0	2.0	9.0	77.7	4.8	17.4	82.8
2003–2007	10.8	41.1	132.5	9.6	40.5	94.4	4.0	14.6	112.5	7.8	23.6	126.6
2008–2012	7.2	35.2	108.1	11.6	46.1	116.7	3.2	15.1	105.1	6.0	18.9	106.0
	35–44			>74 y/o			35–44			>74 y/o		
1983–1987	13.0	30.4	95.5	5.0	39.8	50.9	7.2	15.2	128.7	5.2	19.7	34.2
1988–1992	9.4	26.1	110.2	5.0	54.8	85.0	5.6	12.7	131.7	5.8	35.3	73.9
1993–1997	5.2	19.2	78.3	4.0	41.9	80.4	3.0	9.0	105.3	5.4	29.5	85.8
1998–2002	12.8	48.1	136.7	6.0	48.6	92.1	2.2	8.7	84.4	7.2	30.5	98.1
2003–2007	14.8	58.4	145.7	7.0	43.0	95.6	5.0	16.1	127.5	3.4	12.7	56.6
2008–2012	14.8	52.6	141.5	5.4	29.9	72.1	3.6	13.4	98.8	6.4	18.1	94.4

Secondary Medical Zone ID: 002

	Male						Female					
	Suicide			Suicide			Suicide			Suicide		
	Num per year	Rate /100,000	×100	Num per year	Rate /100,000	×100	Num per year	Rate /100,000	×100	Num per year	Rate /100,000	×100
	Total (>10 y/o)			45–54			Total (>10 y/o)			45–54		
1983–1987	4.6	27.6	65.8	1.0	39.0	77.1	3.4	13.6	75.2	0.4	15.9	92.5
1988–1992	5.2	25.2	109.0	1.6	41.7	121.4	3.0	12.7	100.9	0.4	14.5	99.4
1993–1997	5.0	25.7	110.7	0.8	35.8	97.8	2.6	10.9	106.4	0.6	14.1	109.1
1998–2002	6.4	35.9	105.9	1.2	53.8	96.1	2.2	12.4	96.2	0.2	14.2	94.7
2003–2007	5.0	35.5	96.6	1.2	56.2	100.5	1.8	12.4	94.9	0.8	16.2	118.3
2008–2012	4.2	34.2	98.1	0.2	41.7	85.8	2.0	12.8	100.2	0.2	14.7	98.3
	15–24			55–64			15–24			55–64		
1983–1987	0.4	12.3	88.1	0.8	36.5	85.9	0.2	6.7	97.9	0.8	20.7	104.9
1988–1992	0.4	10.7	108.3	1.0	36.2	101.9	0.0	4.8	91.5	0.8	19.7	110.4
1993–1997	0.0	10.4	91.9	1.0	40.4	100.4	0.0	4.8	92.9	0.4	15.8	99.5
1998–2002	0.0	14.4	91.1	1.6	66.2	104.1	0.0	6.6	93.7	0.2	17.4	92.6
2003–2007	0.4	18.7	107.1	1.2	57.1	99.0	0.0	8.4	93.1	0.0	14.9	88.7
2008–2012	0.2	21.4	101.0	0.6	44.6	91.9	0.0	9.2	94.8	0.4	15.1	101.4
	25–34			65–74			25–34			65–74		
1983–1987	0.8	24.2	94.8	0.6	37.1	84.7	0.2	10.0	93.3	1.0	31.2	100.0
1988–1992	0.0	15.2	79.3	0.6	35.6	101.2	0.0	7.8	89.7	0.2	21.2	81.4
1993–1997	0.2	18.3	93.7	1.4	44.2	138.4	0.0	7.7	92.1	0.8	23.4	119.8
1998–2002	0.6	27.8	103.1	1.4	51.3	112.7	0.0	10.7	92.2	0.6	21.8	103.9
2003–2007	0.0	26.5	85.4	0.8	42.1	98.1	0.0	12.0	92.0	0.2	16.9	90.8
2008–2012	1.0	37.1	113.9	0.8	40.5	102.6	0.0	13.5	94.1	0.4	17.8	100.0
	35–44			>74 y/o			35–44			>74 y/o		
1983–1987	0.8	28.0	87.9	0.0	35.6	45.5	0.2	10.9	91.9	0.6	25.0	43.4
1988–1992	1.2	28.3	119.4	0.4	54.5	84.5	0.6	11.0	113.8	1.0	50.7	105.9
1993–1997	0.8	27.0	110.2	0.8	59.2	113.7	0.2	8.5	100.4	0.6	34.7	100.8
1998–2002	0.4	32.0	90.9	1.2	65.4	124.1	0.2	10.4	100.4	1.0	37.6	121.1
2003–2007	0.6	39.2	97.8	0.8	47.7	105.9	0.2	12.6	100.0	0.6	22.7	101.0
2008–2012	0.8	39.1	105.1	0.6	39.9	96.2	0.2	13.6	100.2	0.8	21.7	112.8

Secondary Medical Zone ID: 003

	Male						Female					
	Suicide			Suicide			Suicide			Suicide		
	Num per year	Rate /100,000	×100	Num per year	Rate /100,000	×100	Num per year	Rate /100,000	×100	Num per year	Rate /100,000	×100
	Total (>10 y/o)			45–54			Total (>10 y/o)			45–54		
1983–1987	9.0	32.0	96.0	2.0	47.9	94.7	2.8	13.0	70.1	0.6	16.3	94.5
1988–1992	6.8	24.9	105.9	1.4	36.1	105.2	4.0	12.7	99.2	0.2	13.1	89.3
1993–1997	5.0	24.0	89.3	1.6	39.0	106.5	3.8	11.0	106.7	0.6	13.4	103.9
1998–2002	8.0	35.2	95.9	2.0	55.3	98.9	3.8	12.6	101.0	0.4	14.4	95.5
2003–2007	8.8	38.2	106.2	2.6	67.0	119.8	3.8	12.7	105.6	0.8	15.4	112.6
2008–2012	6.8	34.2	99.0	2.0	56.4	116.0	2.2	12.5	92.4	0.6	16.1	108.0
	15–24			55–64			15–24			55–64		
1983–1987	1.4	19.8	141.6	1.0	36.0	84.7	0.0	5.8	84.3	0.8	19.6	99.2
1988–1992	0.6	11.2	112.7	0.6	27.9	78.6	0.0	4.6	88.1	0.4	16.0	89.7
1993–1997	0.0	9.9	87.6	1.2	38.4	95.3	0.0	4.7	89.7	0.6	15.9	99.9
1998–2002	0.2	14.7	93.1	2.0	63.9	100.6	0.0	6.4	90.9	0.8	19.5	103.7
2003–2007	0.2	16.6	95.2	1.6	55.1	95.5	0.0	8.1	89.7	1.0	18.5	110.8
2008–2012	0.2	20.2	95.4	0.6	40.6	83.5	0.0	8.8	91.0	0.6	15.2	101.9
	25–34			65–74			25–34			65–74		
1983–1987	0.6	21.6	84.6	1.2	45.1	103.1	0.0	8.5	78.8	0.6	26.1	83.7
1988–1992	0.8	21.0	110.0	1.6	45.4	128.7	0.4	9.3	107.0	0.8	25.9	99.4
1993–1997	0.6	20.3	103.8	0.8	30.6	95.8	0.4	9.1	109.4	1.4	26.5	135.9
1998–2002	0.6	26.2	97.0	1.4	45.3	99.7	0.2	11.2	96.7	1.0	23.0	109.5
2003–2007	0.8	31.2	100.7	0.8	38.0	88.5	0.0	11.5	88.7	0.8	19.7	105.8
2008–2012	0.2	29.2	89.8	0.8	37.7	95.3	0.0	13.0	90.5	0.0	14.8	83.1
	35–44			>74 y/o			35–44			>74 y/o		
1983–1987	1.6	34.0	107.0	1.2	70.7	90.3	0.2	10.4	88.4	0.6	31.0	53.7
1988–1992	1.2	25.7	108.6	0.4	49.4	76.6	0.6	10.5	108.4	1.6	58.5	122.3
1993–1997	0.4	20.3	82.7	0.4	41.2	79.1	0.0	7.1	83.9	0.8	32.0	93.1
1998–2002	0.8	32.9	93.5	0.8	48.3	91.6	0.4	10.9	105.4	1.0	30.7	98.9
2003–2007	1.6	48.6	121.2	1.2	46.6	103.5	0.2	12.1	95.9	1.0	23.9	106.6
2008–2012	1.0	38.2	102.6	2.0	52.3	126.0	0.2	13.1	96.9	0.8	18.8	97.7

Secondary Medical Zone ID: 004

	Male						Female					
	Suicide			Suicide			Suicide			Suicide		
	Num per year	Rate /100,000	×100	Num per year	Rate /100,000	×100	Num per year	Rate /100,000	×100	Num per year	Rate /100,000	×100
	Total (>10 y/o)			45–54			Total (>10 y/o)			45–54		
1983–1987	239.2	30.8	95.8	61.2	52.3	103.5	103.6	12.6	80.7	20.4	17.0	98.5
1988–1992	188.4	23.0	94.0	42.0	32.9	95.9	92.6	10.3	79.4	17.4	12.7	87.2
1993–1997	217.6	23.7	94.5	58.2	37.4	102.3	89.0	8.6	78.0	15.8	9.6	74.3
1998–2002	376.6	37.4	104.5	101.4	60.8	108.6	138.8	12.0	93.3	30.2	16.3	108.1
2003–2007	390.8	37.4	101.0	98.4	64.0	114.5	152.6	12.7	97.7	26.4	15.4	112.4
2008–2012	367.6	34.5	98.8	73.0	50.9	104.7	163.6	13.3	100.1	24.6	15.3	102.7
	15–24			55–64			15–24			55–64		
1983–1987	20.6	12.1	87.0	34.4	41.5	97.7	10.4	6.5	95.7	13.0	15.2	76.9
1988–1992	19.2	10.9	109.7	30.0	30.3	85.3	9.8	5.8	110.2	15.8	15.1	84.5
1993–1997	17.6	10.0	88.6	41.8	37.4	92.8	7.4	4.4	84.5	15.0	12.4	78.3
1998–2002	26.4	16.2	102.6	83.2	65.7	103.5	8.8	5.8	82.1	23.2	16.6	87.9
2003–2007	24.6	16.6	95.4	83.0	54.4	94.2	14.8	10.3	114.0	25.8	15.1	90.3
2008–2012	29.0	22.3	105.2	86.8	51.4	105.7	14.6	11.4	117.3	28.6	15.1	101.2
	25–34			65–74			25–34			65–74		
1983–1987	40.8	26.6	104.0	15.6	35.6	81.2	18.8	11.9	110.1	13.4	24.7	79.1
1988–1992	24.0	17.0	89.0	19.2	36.1	102.5	11.6	7.7	89.3	13.4	20.5	78.6
1993–1997	27.4	18.5	94.6	18.4	25.9	81.2	10.8	6.9	82.5	12.0	14.4	73.7
1998–2002	52.6	32.5	120.2	38.4	42.2	92.9	23.6	13.5	116.3	19.8	18.8	89.7
2003–2007	53.6	32.8	105.6	39.6	38.6	89.9	21.8	12.6	96.9	18.4	15.3	82.1
2008–2012	48.0	33.1	101.6	40.2	35.1	88.8	23.8	15.2	105.9	23.8	17.2	96.8
	35–44			>74 y/o			35–44			>74 y/o		
1983–1987	53.0	32.4	101.8	12.4	52.2	66.6	16.8	10.1	85.3	9.8	23.9	41.5
1988–1992	40.4	24.0	101.4	13.2	49.9	77.4	11.6	6.8	70.6	12.6	29.5	61.6
1993–1997	39.2	25.4	103.7	14.6	43.1	82.8	11.8	7.3	85.6	15.8	27.6	80.2
1998–2002	54.8	37.7	107.0	18.6	40.6	76.9	15.4	9.8	94.9	17.4	22.2	71.5
2003–2007	67.6	44.2	110.2	23.2	35.7	79.2	24.6	14.6	115.3	20.6	19.1	85.0
2008–2012	59.0	35.4	95.2	29.6	34.6	83.5	24.2	13.5	99.5	23.4	16.7	87.1

Secondary Medical Zone ID: 005

	Male						Female					
	Suicide			Suicide			Suicide			Suicide		
	Num per year	Rate /100,000	×100	Num per year	Rate /100,000	×100	Num per year	Rate /100,000	×100	Num per year	Rate /100,000	×100
	Total (>10 y/o)			45–54			Total (>10 y/o)			45–54		
1983–1987	45.8	27.9	79.8	12.4	46.2	91.4	24.2	12.5	70.6	4.0	15.8	91.5
1988–1992	35.2	25.6	105.5	9.0	42.5	123.6	22.6	12.9	100.6	4.4	17.2	117.8
1993–1997	29.0	23.6	91.3	6.2	33.0	90.1	15.0	9.6	85.0	2.2	11.2	86.6
1998–2002	42.4	34.3	94.0	10.2	53.2	95.1	19.4	12.0	94.1	2.2	12.5	83.1
2003–2007	42.2	36.6	97.0	9.0	55.4	99.1	12.0	10.8	71.9	3.6	17.1	125.0
2008–2012	41.8	38.1	108.4	9.2	61.5	126.5	11.2	10.8	74.7	1.0	11.3	75.6
	15–24			55–64			15–24			55–64		
1983–1987	1.8	6.9	49.1	8.6	39.0	91.8	1.6	6.1	88.9	3.4	15.4	78.1
1988–1992	2.2	10.3	104.0	9.0	42.8	120.5	1.0	5.1	96.7	3.4	16.2	90.8
1993–1997	2.0	11.5	101.5	6.4	36.0	89.4	0.6	4.4	84.4	3.2	15.2	95.8
1998–2002	1.6	13.5	85.8	11.8	66.1	104.0	1.0	7.0	99.4	4.8	21.0	111.5
2003–2007	1.4	15.1	86.7	11.2	59.4	103.0	0.6	7.4	82.2	2.0	12.7	76.0
2008–2012	2.6	24.0	113.2	9.8	51.0	105.1	0.6	8.5	88.1	2.4	13.1	88.0
	25–34			65–74			25–34			65–74		
1983–1987	4.8	21.9	85.8	5.0	35.6	81.3	1.8	9.3	86.7	5.2	26.9	86.0
1988–1992	3.0	19.4	101.4	4.2	33.6	95.3	1.8	9.8	112.6	4.6	26.4	101.1
1993–1997	3.4	22.2	113.8	5.0	34.0	106.5	0.8	7.0	84.0	3.2	17.7	90.6
1998–2002	2.8	22.8	84.5	6.6	42.6	93.6	2.2	12.8	110.6	5.2	24.6	117.1
2003–2007	4.2	31.1	100.4	5.0	34.7	80.9	1.0	10.4	80.3	2.6	15.2	81.5
2008–2012	4.8	38.2	117.4	4.8	34.7	87.9	1.4	13.9	97.2	2.2	14.2	80.0
	35–44			>74 y/o			35–44			>74 y/o		
1983–1987	9.0	32.1	100.8	4.2	44.6	56.9	3.6	12.5	105.9	4.6	24.3	42.1
1988–1992	5.0	23.6	99.7	2.8	44.2	68.5	2.4	10.1	104.4	5.0	41.8	87.4
1993–1997	3.4	21.5	87.8	2.6	37.8	72.6	1.2	7.6	88.9	3.8	29.5	85.6
1998–2002	5.0	35.2	99.9	4.2	46.7	88.5	0.4	6.7	64.9	3.6	22.8	73.5
2003–2007	6.6	46.2	115.4	4.6	40.7	90.3	0.8	9.6	75.6	1.4	9.3	41.5
2008–2012	6.2	41.4	111.2	4.2	33.3	80.3	0.4	9.2	67.5	3.2	14.8	76.8

Secondary Medical Zone ID: 006

	Male						Female					
	Suicide			Suicide			Suicide			Suicide		
	Num per year	Rate /100,000	×100	Num per year	Rate /100,000	×100	Num per year	Rate /100,000	×100	Num per year	Rate /100,000	×100
	Total (>10 y/o)			45–54			Total (>10 y/o)			45–54		
1983–1987	41.6	29.9	88.4	11.4	50.8	100.5	17.0	12.5	64.2	5.0	20.9	121.1
1988–1992	31.0	27.8	117.4	9.4	52.7	153.3	13.8	11.8	86.2	1.8	12.7	86.9
1993–1997	27.0	26.5	105.4	5.2	35.1	95.9	16.4	11.9	115.5	3.6	16.9	130.8
1998–2002	41.2	39.6	112.4	11.2	68.3	122.0	17.0	12.8	105.9	2.0	13.8	91.8
2003–2007	44.8	44.5	126.1	8.6	62.9	112.5	17.4	13.7	114.5	3.2	17.8	129.8
2008–2012	36.0	37.6	115.0	6.2	52.4	107.9	15.2	13.9	108.0	1.8	15.2	101.8
	15–24			55–64			15–24			55–64		
1983–1987	3.0	11.2	80.7	7.6	41.6	98.0	1.6	7.2	105.1	2.8	16.7	84.7
1988–1992	1.0	8.5	85.6	6.0	36.5	102.9	1.2	6.5	123.7	2.2	15.1	84.6
1993–1997	1.6	11.6	102.7	6.2	40.9	101.6	0.8	5.6	107.5	3.8	19.8	124.3
1998–2002	2.0	16.7	105.4	6.8	53.1	83.6	0.0	4.7	66.4	4.2	22.3	118.5
2003–2007	1.6	17.2	98.8	11.4	73.0	126.5	1.2	10.5	116.8	2.6	16.4	97.8
2008–2012	1.4	20.0	94.2	7.6	50.4	103.8	1.0	10.8	111.2	3.2	17.1	114.9
	25–34			65–74			25–34			65–74		
1983–1987	3.4	20.6	80.8	4.4	38.2	87.3	0.4	6.5	60.2	2.8	21.4	68.4
1988–1992	3.0	23.0	120.5	2.8	30.3	85.9	0.6	7.2	83.5	3.2	25.7	98.3
1993–1997	2.6	22.1	113.0	2.2	22.9	71.9	0.8	7.9	94.8	2.0	16.2	83.4
1998–2002	3.6	30.0	111.2	5.8	44.6	98.1	1.6	12.7	109.2	3.2	21.1	100.7
2003–2007	4.8	39.1	126.1	5.2	41.2	96.0	0.8	11.2	86.0	4.8	25.8	138.4
2008–2012	4.2	39.9	122.4	7.2	52.4	132.6	1.6	16.3	113.8	2.8	18.4	103.2
	35–44			>74 y/o			35–44			>74 y/o		
1983–1987	6.2	29.8	93.7	5.2	59.0	75.4	3.2	13.8	116.8	1.2	10.6	18.3
1988–1992	4.6	27.1	114.2	4.2	70.1	108.6	2.4	11.7	121.1	2.2	28.3	59.1
1993–1997	5.2	33.7	137.3	4.0	60.6	116.4	1.2	8.5	100.6	4.0	39.4	114.6
1998–2002	7.0	50.3	142.9	4.8	60.4	114.6	1.2	10.1	97.9	4.8	37.4	120.5
2003–2007	8.8	68.2	170.2	4.2	44.3	98.4	1.8	13.9	110.3	3.0	20.3	90.5
2008–2012	5.0	42.0	113.0	4.2	38.1	91.9	1.8	14.7	108.7	3.0	17.4	90.4

Secondary Medical Zone ID: 007

	Male						Female					
	Suicide			Suicide			Suicide			Suicide		
	Num per year	Rate /100,000	×100	Num per year	Rate /100,000	×100	Num per year	Rate /100,000	×100	Num per year	Rate /100,000	×100
	Total (>10 y/o)			45-54			Total (>10 y/o)			45-54		
1983-1987	31.2	33.6	104.9	8.0	52.6	104.0	14.8	14.6	92.4	2.8	18.0	104.5
1988-1992	24.0	28.5	125.7	5.6	43.3	126.1	11.6	12.6	99.2	1.0	11.9	81.7
1993-1997	23.0	28.2	123.4	6.6	49.8	136.1	11.0	11.1	109.1	1.4	12.4	96.1
1998-2002	29.0	39.2	114.9	8.0	70.2	125.4	13.8	13.7	116.9	1.8	15.4	102.4
2003-2007	26.4	40.2	113.0	5.8	65.2	116.6	15.2	15.0	133.6	2.2	17.2	125.6
2008-2012	27.0	39.6	128.1	6.0	67.5	138.9	10.0	13.5	105.7	1.2	15.4	103.4
	15-24			55-64			15-24			55-64		
1983-1987	4.0	20.0	143.3	5.2	44.1	103.9	1.0	7.0	102.3	3.0	22.2	112.5
1988-1992	0.4	8.1	82.2	5.0	41.0	115.4	0.8	6.1	116.3	2.6	19.7	110.6
1993-1997	0.6	10.2	90.5	4.8	42.6	105.7	0.4	5.1	97.5	2.0	16.4	102.9
1998-2002	1.2	16.6	105.1	6.2	62.6	98.6	0.4	6.8	96.3	3.6	23.9	126.7
2003-2007	2.4	24.5	140.3	5.8	58.4	101.3	0.2	7.6	84.5	3.4	21.6	128.9
2008-2012	1.6	25.2	118.6	5.4	52.7	108.5	0.2	8.4	86.9	1.0	13.0	87.1
	25-34			65-74			25-34			65-74		
1983-1987	3.2	26.0	101.7	2.6	37.6	85.9	1.2	10.7	99.0	1.4	20.7	66.3
1988-1992	2.6	25.3	132.6	2.6	36.2	102.7	0.2	6.6	76.1	3.0	30.6	117.3
1993-1997	0.6	15.0	76.7	3.0	35.1	110.2	0.2	6.7	80.7	2.2	21.4	109.7
1998-2002	2.4	29.7	110.1	4.8	49.6	109.0	1.6	14.3	123.7	2.4	22.1	105.3
2003-2007	2.2	32.1	103.7	2.8	35.7	83.1	1.4	15.3	117.6	2.6	21.3	114.1
2008-2012	2.8	39.2	120.5	3.6	41.3	104.5	1.4	17.3	120.7	2.8	21.4	120.4
	35-44			>74 y/o			35-44			>74 y/o		
1983-1987	5.2	35.5	111.4	2.6	61.5	78.5	1.6	11.5	97.7	3.8	50.7	87.9
1988-1992	4.4	32.4	136.7	3.4	75.2	116.6	1.4	10.3	106.3	2.6	44.1	92.1
1993-1997	3.2	30.8	125.6	4.2	76.2	146.3	0.6	7.7	90.5	4.0	49.4	143.4
1998-2002	3.2	39.4	111.9	3.2	55.9	106.1	0.6	9.5	91.7	3.4	35.4	114.1
2003-2007	3.6	47.9	119.5	3.8	52.4	116.4	2.0	16.9	134.1	3.2	27.3	121.6
2008-2012	3.0	40.5	108.8	4.6	52.0	125.3	1.4	15.3	113.2	2.0	17.1	89.1

Secondary Medical Zone ID: 008

	Male						Female					
	Suicide			Suicide			Suicide			Suicide		
	Num per year	Rate /100,000	×100	Num per year	Rate /100,000	×100	Num per year	Rate /100,000	×100	Num per year	Rate /100,000	×100
	Total (>10 y/o)			45-54			Total (>10 y/o)			45-54		
1983-1987	9.0	33.6	108.5	2.6	56.3	111.3	4.2	15.1	96.8	0.8	17.6	102.2
1988-1992	7.4	26.2	118.5	1.8	40.4	117.6	3.0	12.2	89.9	0.4	13.9	95.0
1993-1997	8.2	27.2	126.8	2.4	46.4	126.8	2.4	10.3	89.4	0.4	12.6	97.4
1998-2002	8.8	36.4	109.2	2.8	68.0	121.6	4.6	13.0	112.9	0.6	15.5	103.1
2003-2007	8.4	37.9	112.3	1.8	61.9	110.8	2.8	12.3	97.3	0.2	12.9	94.5
2008-2012	8.8	37.7	121.4	2.6	65.4	134.5	3.8	13.8	110.0	1.0	18.7	125.3
	15-24			55-64			15-24			55-64		
1983-1987	0.6	14.6	104.6	1.0	38.4	90.3	0.0	5.8	85.5	0.4	17.8	90.2
1988-1992	0.2	9.6	97.3	1.2	34.9	98.3	0.0	4.6	87.7	0.6	17.6	98.4
1993-1997	1.0	14.6	129.0	1.0	36.6	91.0	0.2	5.5	105.5	0.0	12.2	77.0
1998-2002	0.2	15.0	95.0	0.8	49.8	78.4	0.4	8.1	115.5	1.4	22.4	118.9
2003-2007	0.4	18.0	103.4	1.2	51.8	89.7	0.2	9.4	103.9	0.8	17.7	105.6
2008-2012	0.4	22.0	103.7	1.8	52.2	107.4	0.0	8.9	91.9	0.4	14.6	97.8
	25-34			65-74			25-34			65-74		
1983-1987	0.8	25.9	101.3	1.2	47.8	109.1	0.0	8.9	82.6	1.2	35.9	115.0
1988-1992	0.4	18.7	97.6	0.8	35.6	101.0	0.6	10.5	121.3	0.2	19.6	75.1
1993-1997	0.4	19.6	100.5	2.0	47.0	147.3	0.4	9.3	111.6	1.0	23.6	120.9
1998-2002	0.4	26.2	96.9	1.8	50.3	110.6	0.2	11.4	98.1	0.8	21.9	104.4
2003-2007	1.6	40.2	129.7	1.4	44.6	104.0	0.2	12.8	98.4	0.8	19.8	106.4
2008-2012	0.8	34.9	107.3	0.2	31.7	80.3	0.4	15.4	107.4	0.4	16.9	94.7
	35-44			>74 y/o			35-44			>74 y/o		
1983-1987	1.6	35.6	111.9	1.0	74.7	95.3	0.4	11.4	96.7	1.4	64.3	111.5
1988-1992	1.2	27.5	116.0	1.8	86.4	133.9	0.6	10.7	110.7	0.6	36.4	76.0
1993-1997	0.8	26.0	106.2	0.6	46.0	88.3	0.0	7.4	86.5	0.4	24.1	70.1
1998-2002	0.8	35.2	100.1	2.0	73.2	138.9	0.0	9.1	88.3	1.2	34.5	111.0
2003-2007	0.6	37.4	93.3	1.4	51.2	113.7	0.0	11.3	89.7	0.6	19.5	87.1
2008-2012	1.4	42.7	114.6	1.4	45.2	109.0	0.8	15.9	117.4	0.8	19.3	100.1

Secondary Medical Zone ID: 009

	Male						Female					
	Suicide			Suicide			Suicide			Suicide		
	Num per year	Rate /100,000	×100	Num per year	Rate /100,000	×100	Num per year	Rate /100,000	×100	Num per year	Rate /100,000	×100
	Total (>10 y/o)			45-54			Total (>10 y/o)			45-54		
1983-1987	42.4	32.5	100.1	14.6	64.7	127.9	14.2	11.7	63.8	4.4	19.5	113.2
1988-1992	22.4	22.5	86.7	7.0	38.3	111.4	13.4	11.5	83.9	3.0	15.1	103.2
1993-1997	25.0	23.8	93.5	6.6	37.0	101.2	12.0	10.1	88.0	1.6	10.5	81.4
1998-2002	36.6	35.7	95.8	6.2	42.5	75.9	12.2	11.2	80.0	1.6	12.1	80.2
2003-2007	35.2	36.9	95.5	6.2	48.4	86.6	12.2	12.0	85.2	1.6	12.6	92.2
2008-2012	35.6	36.7	106.2	4.6	45.0	92.6	18.4	15.7	117.3	3.4	21.0	140.8
	15-24			55-64			15-24			55-64		
1983-1987	2.0	10.6	76.2	7.8	48.1	113.1	1.0	5.9	86.4	2.0	15.0	76.1
1988-1992	1.0	8.1	82.1	4.6	31.4	88.4	1.2	6.2	118.4	3.4	19.5	109.2
1993-1997	1.4	10.2	89.8	5.8	37.4	92.9	0.8	5.4	104.4	2.6	15.4	97.1
1998-2002	2.0	15.6	98.9	8.8	57.4	90.3	0.6	6.5	92.8	2.0	14.8	78.8
2003-2007	2.4	19.2	110.1	7.2	47.4	82.1	0.8	9.1	100.7	2.4	14.8	88.1
2008-2012	2.8	23.9	112.7	8.2	50.2	103.3	2.0	15.7	161.9	3.4	16.8	112.7
	25-34			65-74			25-34			65-74		
1983-1987	4.8	26.9	105.1	4.4	41.6	94.9	1.4	9.2	85.5	2.4	22.0	70.4
1988-1992	2.4	19.8	103.8	1.6	23.4	66.4	0.4	6.3	72.3	2.0	19.3	73.9
1993-1997	1.6	16.8	86.0	4.4	37.8	118.6	0.8	7.6	91.1	2.6	19.4	99.8
1998-2002	4.2	31.2	115.5	5.8	45.4	99.9	0.4	8.4	72.1	3.2	21.5	102.4
2003-2007	5.2	38.7	124.7	5.0	38.2	89.1	0.8	10.5	81.0	1.6	13.7	73.5
2008-2012	5.6	43.8	134.6	5.6	40.3	102.0	2.6	19.0	132.8	1.8	14.1	79.5
	35-44			>74 y/o			35-44			>74 y/o		
1983-1987	7.0	31.0	97.3	1.6	35.7	45.6	1.6	9.0	75.9	1.4	16.2	28.0
1988-1992	3.8	22.0	92.9	1.8	43.0	66.6	1.6	9.0	92.9	1.8	27.2	56.8
1993-1997	2.8	21.3	86.7	2.4	43.4	83.3	2.2	11.3	133.4	1.4	18.7	54.4
1998-2002	6.2	47.5	134.8	3.2	46.4	88.0	2.0	12.8	124.0	2.4	21.6	69.6
2003-2007	6.0	50.4	125.8	3.0	36.2	80.5	2.8	16.9	134.1	2.2	16.0	71.5
2008-2012	5.6	41.8	112.5	3.2	32.4	78.2	2.4	15.8	116.6	2.8	16.6	86.4

Secondary Medical Zone ID: 010

	Male						Female					
	Suicide			Suicide			Suicide			Suicide		
	Num per year	Rate /100,000	×100	Num per year	Rate /100,000	×100	Num per year	Rate /100,000	×100	Num per year	Rate /100,000	×100
	Total (>10 y/o)			45–54			Total (>10 y/o)			45–54		
1983–1987	33.4	34.2	109.4	7.6	51.6	102.0	13.2	14.0	87.7	2.4	17.0	98.5
1988–1992	24.4	25.9	108.4	6.0	38.2	111.2	11.0	11.8	88.9	1.8	13.4	91.9
1993–1997	27.2	26.5	108.8	6.6	37.6	102.8	12.2	10.9	101.5	2.0	12.2	94.1
1998–2002	46.8	41.0	121.0	9.2	51.8	92.6	14.0	12.3	95.4	4.0	18.2	121.1
2003–2007	47.2	41.6	120.3	11.0	66.7	119.2	14.0	12.7	96.3	3.8	18.4	134.0
2008–2012	44.2	37.9	119.3	8.0	56.0	115.3	13.6	12.7	95.1	1.8	14.3	95.6
	15–24			55–64			15–24			55–64		
1983–1987	4.2	21.6	154.8	4.2	40.5	95.3	1.2	7.4	108.1	2.2	19.8	100.2
1988–1992	2.0	11.7	117.9	3.8	32.8	92.4	1.0	5.9	111.5	1.8	16.3	91.5
1993–1997	2.6	14.1	124.8	5.8	42.8	106.3	0.6	4.7	90.5	2.0	15.3	96.0
1998–2002	3.0	19.1	120.7	12.4	80.6	126.8	1.8	9.6	136.4	3.4	20.6	109.6
2003–2007	2.4	19.0	108.9	11.2	65.6	113.7	0.8	8.3	92.7	2.0	14.3	85.3
2008–2012	2.4	22.2	104.8	12.6	63.2	130.2	1.0	10.2	104.7	2.4	14.0	93.7
	25–34			65–74			25–34			65–74		
1983–1987	5.6	30.3	118.7	2.0	36.5	83.5	1.4	9.5	88.0	2.4	31.8	101.8
1988–1992	3.4	23.3	121.7	2.2	34.0	96.5	1.0	8.0	92.7	1.8	23.5	90.1
1993–1997	3.6	23.9	122.6	1.8	25.2	79.0	1.0	7.9	94.9	1.8	18.5	95.1
1998–2002	5.2	31.9	118.1	5.2	48.2	106.0	0.8	9.0	77.9	2.2	19.7	94.0
2003–2007	7.2	42.0	135.5	5.0	43.4	101.0	1.6	12.2	94.0	2.2	17.7	95.0
2008–2012	3.6	30.1	92.5	5.8	43.7	110.7	1.8	14.5	101.5	2.4	17.4	98.0
	35–44			>74 y/o			35–44			>74 y/o		
1983–1987	8.2	40.0	125.7	1.0	45.5	58.1	1.4	9.5	80.7	2.2	40.6	70.3
1988–1992	4.4	23.8	100.3	2.6	71.0	110.1	1.6	9.1	94.2	1.8	38.4	80.3
1993–1997	4.0	24.6	100.4	2.8	61.4	117.9	2.6	12.1	142.1	2.0	31.4	91.3
1998–2002	7.8	49.2	139.7	4.0	65.3	123.9	0.4	6.9	66.9	1.4	19.5	62.9
2003–2007	6.0	43.3	108.0	4.0	50.9	113.1	2.2	14.1	111.5	1.4	14.5	64.5
2008–2012	6.8	41.8	112.3	5.0	49.7	119.9	1.8	13.1	96.7	2.4	17.3	90.2

Secondary Medical Zone ID: 011

	Male						Female					
	Suicide			Suicide			Suicide			Suicide		
	Num per year	Rate /100,000	×100	Num per year	Rate /100,000	×100	Num per year	Rate /100,000	×100	Num per year	Rate /100,000	×100
	Total (>10 y/o)			45-54			Total (>10 y/o)			45-54		
1983-1987	19.6	36.2	126.1	4.4	55.1	109.0	8.0	15.1	101.6	1.6	19.0	110.1
1988-1992	15.0	28.5	132.9	2.6	37.2	108.4	8.2	13.8	119.2	1.0	14.8	101.0
1993-1997	13.4	27.3	118.8	1.8	33.0	90.1	7.0	11.8	117.8	1.4	15.0	116.4
1998-2002	15.6	36.4	103.0	3.4	53.6	95.8	6.6	12.7	103.8	1.2	15.9	105.6
2003-2007	17.2	37.8	114.3	3.4	57.2	102.3	8.6	13.9	125.6	1.0	14.7	107.3
2008-2012	16.4	36.8	118.7	3.6	58.7	120.7	4.8	12.5	96.6	0.2	12.6	84.6
	15-24			55-64			15-24			55-64		
1983-1987	0.8	13.8	99.1	5.2	65.4	153.9	0.4	6.7	97.9	2.0	24.4	123.7
1988-1992	1.0	11.8	118.9	3.6	47.1	132.7	0.6	6.4	120.7	2.2	23.4	131.0
1993-1997	1.0	12.9	113.9	3.2	46.4	115.1	0.2	4.9	94.6	1.2	17.1	107.8
1998-2002	1.2	17.9	113.3	3.8	66.4	104.4	0.2	6.6	94.1	2.2	23.6	125.2
2003-2007	1.4	21.3	121.9	4.2	65.7	113.8	0.0	7.3	81.3	1.8	19.7	117.8
2008-2012	0.8	22.3	105.3	4.8	60.7	125.0	0.4	10.5	107.8	1.2	15.9	106.7
	25-34			65-74			25-34			65-74		
1983-1987	2.4	28.9	112.9	2.6	58.3	133.2	0.6	10.1	94.1	1.2	29.9	95.8
1988-1992	1.4	21.1	110.3	1.2	34.7	98.6	0.8	10.0	114.9	2.0	34.5	132.2
1993-1997	2.2	26.8	137.4	1.8	36.6	114.8	0.8	9.8	117.5	1.6	24.6	126.0
1998-2002	2.0	30.2	111.8	2.4	47.5	104.4	0.2	10.0	86.4	0.4	15.9	75.8
2003-2007	1.4	29.4	94.7	2.2	44.2	103.1	1.0	14.8	114.0	1.8	23.2	124.4
2008-2012	1.4	32.4	99.3	1.8	39.7	100.6	0.2	12.8	89.3	0.8	17.1	96.1
	35-44			>74 y/o			35-44			>74 y/o		
1983-1987	2.8	34.4	108.0	1.4	70.5	90.0	1.0	12.2	103.6	1.2	42.6	73.8
1988-1992	2.2	26.6	112.4	3.0	102.2	158.4	0.2	8.0	82.7	1.4	45.6	95.3
1993-1997	2.2	29.1	118.7	1.2	51.7	99.3	1.2	11.5	134.8	0.6	22.9	66.5
1998-2002	1.6	32.8	93.1	1.2	47.5	90.1	0.4	9.8	94.6	1.8	35.0	112.7
2003-2007	1.6	36.7	91.5	3.0	64.0	142.2	1.2	15.2	120.5	1.8	27.6	123.2
2008-2012	1.8	37.4	100.5	2.2	46.7	112.6	0.4	12.7	93.5	1.6	21.8	113.1

Secondary Medical Zone ID: 012

	Male						Female					
	Suicide			Suicide			Suicide			Suicide		
	Num per year	Rate /100,000	×100	Num per year	Rate /100,000	×100	Num per year	Rate /100,000	×100	Num per year	Rate /100,000	×100
	Total (>10 y/o)			45–54			Total (>10 y/o)			45–54		
1983–1987	82.4	38.8	123.3	23.0	68.1	134.6	30.4	13.4	84.1	6.0	18.2	105.6
1988–1992	47.8	25.8	105.5	13.4	43.7	127.1	30.8	13.5	108.5	4.0	13.2	90.5
1993–1997	50.6	26.4	105.1	11.6	36.6	100.1	21.8	9.7	88.3	4.0	11.8	91.6
1998–2002	87.0	41.7	120.6	20.2	62.3	111.4	28.4	12.0	93.7	4.8	14.1	93.5
2003–2007	87.8	44.1	123.1	20.0	71.8	128.4	28.0	12.1	95.0	3.0	11.3	82.7
2008–2012	67.2	36.8	104.6	14.6	59.8	123.1	30.8	13.5	103.5	3.8	14.6	98.0
	15–24			55–64			15–24			55–64		
1983–1987	5.8	14.7	105.7	13.8	53.7	126.3	2.8	7.3	106.5	4.0	16.6	83.9
1988–1992	2.8	9.7	97.7	7.8	31.6	88.9	2.2	6.4	120.7	5.2	18.8	105.1
1993–1997	3.0	11.1	98.0	11.6	42.6	105.8	0.4	3.0	58.2	3.2	12.4	78.1
1998–2002	3.2	14.3	90.2	24.0	83.2	130.9	1.6	6.7	95.7	5.0	16.9	89.6
2003–2007	3.6	18.0	102.9	22.6	72.3	125.3	1.4	7.8	86.3	7.6	19.9	118.6
2008–2012	3.4	21.3	100.5	15.6	50.0	102.9	2.0	10.9	112.1	5.6	15.3	102.8
	25–34			65–74			25–34			65–74		
1983–1987	13.2	37.9	148.5	5.2	36.9	84.3	2.2	7.9	73.3	3.6	21.9	70.0
1988–1992	5.0	20.0	104.8	3.6	26.7	75.9	2.6	9.1	105.0	6.2	31.3	120.1
1993–1997	6.4	24.9	127.6	4.6	26.2	82.2	1.8	7.4	88.7	5.2	22.5	115.4
1998–2002	8.2	31.1	115.2	9.4	42.7	93.9	2.4	9.9	85.3	5.6	21.8	103.6
2003–2007	8.8	35.1	113.1	8.6	37.0	86.3	2.2	10.3	79.3	4.8	17.6	94.6
2008–2012	7.2	34.3	105.3	7.2	31.5	79.9	2.6	13.0	90.7	4.6	16.0	90.2
	35–44			>74 y/o			35–44			>74 y/o		
1983–1987	14.2	36.6	115.1	7.0	77.6	99.1	5.8	13.7	116.0	6.0	39.0	67.6
1988–1992	9.4	27.4	115.7	5.6	67.6	104.7	4.8	11.8	122.3	5.8	46.6	97.4
1993–1997	8.6	29.3	119.7	4.4	46.5	89.3	2.6	8.4	99.0	4.6	30.6	88.9
1998–2002	12.6	47.8	135.8	9.2	69.3	131.4	3.4	11.6	112.2	5.6	28.2	90.8
2003–2007	14.8	59.3	147.9	9.4	54.4	120.8	4.2	14.4	114.2	4.6	18.2	80.9
2008–2012	10.8	42.1	113.1	8.4	40.3	97.2	5.2	16.7	123.0	6.6	20.1	104.3

Secondary Medical Zone ID: 013

	Male						Female					
	Suicide			Suicide			Suicide			Suicide		
	Num per year	Rate /100,000	×100	Num per year	Rate /100,000	×100	Num per year	Rate /100,000	×100	Num per year	Rate /100,000	×100
	Total (>10 y/o)			45–54			Total (>10 y/o)			45–54		
1983–1987	14.6	29.7	86.7	3.8	46.0	90.9	10.4	15.5	102.7	1.8	18.8	109.2
1988–1992	15.0	28.1	128.6	2.4	35.6	103.6	8.6	13.8	116.5	0.8	13.7	93.6
1993–1997	13.6	27.0	121.0	2.4	37.6	102.7	7.0	11.5	114.7	0.8	12.6	97.9
1998–2002	17.0	38.2	112.6	4.8	68.1	121.7	9.8	14.3	130.3	1.8	18.4	122.1
2003–2007	20.4	43.4	138.4	4.2	71.3	127.5	7.6	13.7	117.1	0.6	13.2	96.7
2008–2012	13.0	36.0	105.5	2.8	54.4	112.0	6.6	13.2	110.7	0.8	15.7	105.5
	15–24			55–64			15–24			55–64		
1983–1987	1.0	12.1	86.9	3.0	42.7	100.4	0.8	7.3	106.4	1.4	19.7	99.6
1988–1992	0.6	10.0	101.1	3.0	40.3	113.6	0.2	4.9	92.0	0.8	15.9	88.9
1993–1997	0.8	12.4	109.7	3.0	43.8	108.8	0.0	4.2	81.4	1.2	16.6	104.7
1998–2002	0.2	13.5	85.6	2.4	53.0	83.4	0.4	7.4	105.8	2.2	23.1	122.8
2003–2007	1.0	19.7	112.8	5.2	75.7	131.1	0.2	8.4	93.9	1.0	16.6	99.0
2008–2012	1.0	23.8	112.3	3.6	55.2	113.6	0.4	10.4	107.3	1.8	18.3	122.6
	25–34			65–74			25–34			65–74		
1983–1987	2.0	26.8	104.8	2.4	49.7	113.6	0.6	10.1	93.6	1.8	31.9	102.1
1988–1992	1.8	24.9	130.4	1.6	37.0	105.0	0.6	9.2	106.6	3.0	42.1	161.4
1993–1997	1.4	22.8	116.5	2.4	40.9	128.3	0.6	9.2	110.2	1.8	25.3	129.8
1998–2002	2.2	33.0	122.2	3.2	52.4	115.2	1.0	13.5	116.8	0.8	18.2	86.6
2003–2007	1.8	34.3	110.6	2.6	46.7	108.7	1.2	16.1	124.2	1.6	21.4	115.2
2008–2012	1.0	31.0	95.3	1.0	33.3	84.2	0.0	12.0	84.0	1.8	21.4	120.4
	35–44			>74 y/o			35–44			>74 y/o		
1983–1987	1.6	25.4	79.8	0.8	47.1	60.1	1.0	11.9	100.6	3.0	58.1	100.7
1988–1992	3.2	34.5	145.4	2.0	72.3	112.1	1.0	10.8	111.7	2.2	55.0	115.0
1993–1997	1.0	21.9	89.5	2.6	74.5	143.0	0.8	9.9	116.5	1.8	39.1	113.5
1998–2002	2.4	41.8	118.8	1.8	53.7	101.9	0.6	10.9	105.2	3.0	47.6	153.3
2003–2007	3.0	54.2	135.4	2.6	55.2	122.5	1.2	15.6	123.5	1.8	26.4	117.5
2008–2012	2.2	41.4	111.2	1.4	35.6	85.7	0.2	12.1	89.4	1.6	21.0	109.1

Secondary Medical Zone ID: 014

	Male						Female					
	Suicide			Suicide			Suicide			Suicide		
	Num per year	Rate /100,000	×100	Num per year	Rate /100,000	×100	Num per year	Rate /100,000	×100	Num per year	Rate /100,000	×100
	Total (>10 y/o)			45–54			Total (>10 y/o)			45–54		
1983–1987	9.8	32.2	96.5	2.2	46.2	91.4	4.4	14.1	86.4	0.6	16.1	93.4
1988–1992	5.8	24.2	96.2	1.4	35.5	103.5	4.4	13.3	110.4	0.8	15.6	106.6
1993–1997	7.8	27.1	117.7	2.6	47.0	128.5	6.0	12.6	143.6	1.4	16.9	130.8
1998–2002	10.8	38.2	115.9	2.2	58.1	103.9	5.4	13.6	121.0	0.6	15.3	101.8
2003–2007	10.0	39.1	112.2	2.4	63.8	114.1	3.2	12.5	99.0	0.4	13.6	99.0
2008–2012	8.6	35.6	107.1	1.4	48.3	99.5	3.0	12.8	98.5	0.0	12.7	84.9
	15–24			55–64			15–24			55–64		
1983–1987	1.2	17.3	124.3	1.8	43.9	103.3	1.0	9.8	143.0	0.8	20.0	101.2
1988–1992	0.2	9.3	94.0	1.0	32.3	91.0	0.0	4.6	87.3	0.8	18.8	105.3
1993–1997	0.2	10.7	94.6	0.4	30.9	76.8	0.0	4.6	88.6	1.2	19.9	124.9
1998–2002	0.6	16.8	106.6	1.6	59.1	93.0	0.0	6.3	89.6	1.4	22.4	119.2
2003–2007	1.0	20.9	119.9	0.8	45.7	79.2	0.2	9.1	100.7	1.0	18.5	110.6
2008–2012	0.2	19.9	93.9	1.8	49.8	102.5	0.2	9.8	101.3	0.6	15.3	102.3
	25–34			65–74			25–34			65–74		
1983–1987	1.0	24.0	93.8	1.8	54.8	125.3	0.2	9.5	88.4	0.8	29.0	92.8
1988–1992	0.6	18.3	95.9	0.4	30.4	86.3	0.4	9.2	105.7	1.2	32.5	124.8
1993–1997	0.4	17.8	91.0	0.8	32.1	100.5	0.6	9.8	117.7	1.0	24.0	123.1
1998–2002	1.6	31.8	117.6	1.2	44.3	97.5	0.8	13.5	116.1	0.6	20.5	97.8
2003–2007	1.2	33.0	106.3	1.2	42.4	98.8	0.0	11.1	85.6	0.4	17.3	92.7
2008–2012	1.2	35.0	107.5	1.6	44.9	113.7	0.0	12.6	88.2	1.2	21.2	119.1
	35–44			>74 y/o			35–44			>74 y/o		
1983–1987	1.4	30.5	95.9	0.4	50.8	64.9	0.2	10.3	87.2	0.8	35.6	61.6
1988–1992	1.4	27.2	114.8	0.8	64.3	99.6	0.0	8.2	84.8	1.2	56.3	117.6
1993–1997	2.2	37.1	151.5	1.2	65.5	125.7	0.2	8.1	95.2	1.6	54.3	157.8
1998–2002	2.0	44.2	125.5	1.6	67.1	127.3	0.4	10.7	103.9	1.6	44.6	143.7
2003–2007	2.4	54.7	136.4	1.0	45.7	101.4	0.6	13.7	108.4	0.6	20.7	92.2
2008–2012	2.0	43.4	116.5	0.4	32.4	78.1	0.6	14.4	106.3	0.4	16.1	83.7

Secondary Medical Zone ID: 015

	Male						Female					
	Suicide			Suicide			Suicide			Suicide		
	Num per year	Rate /100,000	×100	Num per year	Rate /100,000	×100	Num per year	Rate /100,000	×100	Num per year	Rate /100,000	×100
	Total (>10 y/o)			45-54			Total (>10 y/o)			45-54		
1983-1987	13.0	30.6	87.4	2.4	39.4	77.9	5.8	13.2	74.4	1.0	16.2	94.2
1988-1992	10.2	25.8	112.2	1.8	33.8	98.5	5.6	12.8	100.7	1.0	15.2	104.0
1993-1997	9.2	25.5	108.0	1.8	36.3	99.1	6.8	12.0	129.5	0.8	13.3	102.8
1998-2002	12.6	36.7	107.8	3.0	58.5	104.5	5.4	12.7	105.3	0.2	12.8	84.7
2003-2007	11.8	37.5	107.9	2.6	59.2	105.9	4.0	12.4	96.8	0.8	14.7	107.6
2008-2012	8.6	33.8	97.3	1.4	46.1	94.9	3.0	12.6	92.1	1.0	17.7	118.4
	15-24			55-64			15-24			55-64		
1983-1987	0.2	8.2	58.6	2.2	39.9	94.0	0.0	5.2	75.6	0.4	15.0	76.1
1988-1992	0.6	10.5	106.1	2.0	36.3	102.2	0.2	5.2	98.1	0.8	17.0	95.3
1993-1997	0.2	10.5	92.6	1.6	37.2	92.3	0.0	4.5	86.3	1.2	18.0	113.4
1998-2002	1.2	19.7	124.9	3.0	65.8	103.5	0.0	6.2	88.6	1.2	19.9	105.9
2003-2007	0.4	17.3	99.1	2.6	58.8	101.8	0.0	7.8	87.3	1.0	17.4	103.9
2008-2012	0.2	20.0	94.3	2.2	49.4	101.6	0.0	8.7	89.4	0.4	13.7	92.1
	25-34			65-74			25-34			65-74		
1983-1987	1.4	24.3	95.2	2.6	55.7	127.3	0.6	10.6	98.1	1.6	31.0	99.2
1988-1992	1.2	22.3	116.7	1.0	33.9	96.3	0.6	9.7	112.2	1.6	31.2	119.5
1993-1997	1.2	22.9	117.5	2.0	41.6	130.5	0.4	8.8	105.2	1.8	27.6	141.4
1998-2002	0.4	23.2	86.0	2.0	47.1	103.6	0.6	12.5	107.5	1.0	21.4	101.8
2003-2007	2.0	38.7	124.8	1.2	38.2	89.0	0.2	12.0	92.6	0.6	17.1	91.7
2008-2012	0.6	30.9	94.9	0.8	34.6	87.6	0.2	13.6	95.2	0.2	14.8	82.9
	35-44			>74 y/o			35-44			>74 y/o		
1983-1987	3.0	37.2	116.9	1.2	54.7	69.8	0.8	11.6	98.4	1.4	32.7	56.6
1988-1992	1.6	26.0	109.6	2.0	82.1	127.3	0.6	9.8	101.5	0.8	34.8	72.7
1993-1997	0.8	22.1	90.3	1.6	65.1	125.1	0.4	8.6	100.9	2.2	53.9	156.7
1998-2002	1.6	37.8	107.2	1.4	56.1	106.5	0.4	10.5	101.2	2.0	41.8	134.6
2003-2007	1.0	35.8	89.3	2.0	55.0	122.2	0.4	12.6	99.7	1.0	21.7	96.7
2008-2012	1.8	41.2	110.8	1.6	43.3	104.3	0.4	13.5	99.7	0.8	17.4	90.3

Secondary Medical Zone ID: 016

	Male						Female					
	Suicide			Suicide			Suicide			Suicide		
	Num per year	Rate /100,000	×100	Num per year	Rate /100,000	×100	Num per year	Rate /100,000	×100	Num per year	Rate /100,000	×100
	Total (>10 y/o)			45-54			Total (>10 y/o)			45-54		
1983-1987	21.0	37.7	128.0	5.0	57.3	113.3	6.6	14.2	87.1	1.0	15.7	91.4
1988-1992	10.6	24.6	99.4	3.2	40.2	117.1	6.4	12.8	100.9	0.8	13.7	93.8
1993-1997	10.6	24.9	99.3	2.0	33.3	91.1	7.0	11.6	118.6	1.0	13.2	102.4
1998-2002	17.4	37.6	111.6	4.8	62.6	111.9	7.0	13.1	108.9	1.0	15.0	99.4
2003-2007	22.2	44.4	142.7	5.2	73.2	130.9	5.4	12.3	99.6	1.0	14.6	106.3
2008-2012	13.6	35.5	105.0	3.2	54.7	112.5	4.4	12.6	94.6	0.6	14.4	96.8
	15-24			55-64			15-24			55-64		
1983-1987	1.2	15.0	107.2	3.6	50.7	119.4	0.2	5.6	82.5	0.8	17.2	87.4
1988-1992	0.4	9.3	94.3	2.0	33.4	94.1	0.6	6.4	120.8	1.0	17.2	96.6
1993-1997	0.8	12.3	108.5	2.2	38.0	94.5	0.2	5.0	96.6	1.0	16.0	100.4
1998-2002	0.2	13.4	85.1	3.6	63.4	99.8	0.6	8.3	117.9	2.0	22.5	119.8
2003-2007	1.6	22.9	131.4	5.8	79.4	137.6	0.0	7.4	82.5	1.6	18.9	112.9
2008-2012	0.8	22.7	107.0	1.6	39.6	81.6	0.4	10.7	109.9	1.0	15.1	101.5
	25-34			65-74			25-34			65-74		
1983-1987	2.8	30.1	117.8	2.4	56.2	128.4	1.0	11.5	106.8	1.4	32.0	102.3
1988-1992	1.0	17.8	93.0	1.0	32.3	91.8	0.6	9.0	103.3	1.0	24.3	93.2
1993-1997	1.4	21.7	111.2	1.4	32.7	102.5	1.2	11.3	136.4	1.6	24.7	126.5
1998-2002	1.6	28.0	103.5	4.4	65.9	144.9	0.6	11.7	101.4	1.6	24.5	116.6
2003-2007	1.8	32.6	105.2	2.4	46.9	109.3	0.4	12.2	93.9	1.0	18.8	100.8
2008-2012	2.2	37.8	116.2	2.8	48.3	122.3	0.4	13.8	96.0	0.8	17.2	96.6
	35-44			>74 y/o			35-44			>74 y/o		
1983-1987	3.6	38.1	119.8	2.4	97.2	124.1	0.8	11.1	94.0	1.4	46.9	81.4
1988-1992	2.2	26.1	110.1	0.8	54.2	83.9	0.4	8.5	87.9	2.0	60.4	126.3
1993-1997	1.8	25.9	105.5	1.0	49.7	95.4	0.2	7.0	82.9	1.8	45.0	130.7
1998-2002	2.2	37.5	106.4	0.6	39.1	74.2	0.4	9.7	93.7	0.8	23.6	76.0
2003-2007	3.4	54.4	135.7	2.0	52.9	117.5	0.2	10.9	86.6	1.2	22.5	100.5
2008-2012	2.0	38.0	102.2	1.0	34.9	84.1	0.4	12.8	94.4	0.8	16.4	85.4

Secondary Medical Zone ID: 017

	Male						Female					
	Suicide			Suicide			Suicide			Suicide		
	Num per year	Rate /100,000	×100	Num per year	Rate /100,000	×100	Num per year	Rate /100,000	×100	Num per year	Rate /100,000	×100
	Total (>10 y/o)			45–54			Total (>10 y/o)			45–54		
1983–1987	48.8	35.0	108.5	13.6	60.1	118.8	18.8	13.1	79.9	2.0	12.8	74.2
1988–1992	31.8	26.5	111.4	7.0	38.0	110.6	19.8	13.8	114.0	4.0	17.9	122.2
1993–1997	29.6	25.2	100.7	5.8	32.9	89.8	18.4	12.3	119.9	1.8	10.9	84.6
1998–2002	47.2	38.0	108.1	9.2	49.9	89.1	18.6	12.8	104.1	2.2	13.0	86.0
2003–2007	52.4	42.5	120.0	12.6	70.7	126.4	19.2	13.3	110.3	2.6	14.3	104.6
2008–2012	43.6	37.3	109.8	10.0	60.6	124.7	20.2	14.1	116.0	4.4	21.9	146.8
	15–24			55–64			15–24			55–64		
1983–1987	3.0	11.9	85.6	5.0	33.9	79.7	1.4	6.5	94.9	2.8	17.8	90.4
1988–1992	2.2	10.8	109.5	4.6	30.5	85.9	1.2	6.1	115.0	4.2	22.0	123.4
1993–1997	1.8	10.6	93.6	5.2	34.6	86.0	1.8	7.9	151.0	4.0	20.2	127.1
1998–2002	1.8	13.4	84.6	8.8	57.5	90.5	0.6	6.0	85.3	4.4	21.9	116.4
2003–2007	2.8	19.0	109.1	11.8	65.7	113.8	1.0	9.0	99.7	4.6	20.3	121.2
2008–2012	2.2	20.2	95.4	8.8	48.3	99.4	1.0	10.2	104.9	1.4	11.1	74.4
	25–34			65–74			25–34			65–74		
1983–1987	7.0	30.2	118.0	5.6	52.9	120.7	1.2	7.9	73.0	4.2	32.4	103.5
1988–1992	3.2	19.4	101.6	4.4	43.5	123.3	1.8	9.6	110.9	3.4	28.0	107.4
1993–1997	5.0	27.7	141.9	4.2	35.5	111.4	1.2	8.1	97.3	3.4	23.4	120.1
1998–2002	5.4	31.5	116.6	7.2	51.1	112.3	2.2	12.8	110.7	3.4	22.0	104.9
2003–2007	5.8	35.5	114.5	4.8	36.9	86.1	2.2	14.1	108.2	3.4	20.1	108.2
2008–2012	5.2	36.9	113.4	4.4	35.0	88.6	2.0	15.3	106.8	3.6	19.9	112.0
	35–44			>74 y/o			35–44			>74 y/o		
1983–1987	8.8	35.7	112.1	5.8	84.6	108.0	2.0	9.9	84.1	5.2	44.9	77.9
1988–1992	6.2	28.2	119.0	3.8	72.0	111.7	2.2	9.9	102.8	3.0	41.0	85.7
1993–1997	3.8	22.3	91.0	3.8	61.7	118.6	2.6	11.4	134.2	3.6	38.2	110.9
1998–2002	8.8	49.4	140.2	5.8	70.9	134.5	1.4	9.6	92.5	4.4	35.2	113.4
2003–2007	9.0	55.4	138.2	5.4	52.5	116.7	1.8	12.4	98.0	3.6	23.0	102.6
2008–2012	7.0	42.5	114.1	5.8	45.6	109.9	1.8	13.1	96.3	6.0	28.4	147.7

Secondary Medical Zone ID: 018

	Male						Female					
	Suicide			Suicide			Suicide			Suicide		
	Num per year	Rate /100,000	×100	Num per year	Rate /100,000	×100	Num per year	Rate /100,000	×100	Num per year	Rate /100,000	×100
	Total (>10 y/o)			45–54			Total (>10 y/o)			45–54		
1983–1987	20.4	36.2	118.1	6.0	62.6	123.7	10.2	15.7	107.3	1.6	18.0	104.3
1988–1992	12.0	25.1	106.3	2.8	37.9	110.5	9.0	13.9	119.5	1.2	15.0	102.8
1993–1997	17.0	29.4	138.9	4.0	46.8	127.9	7.0	11.2	112.2	0.4	10.9	84.5
1998–2002	20.4	39.1	123.8	4.6	63.6	113.7	7.4	12.7	106.3	0.2	11.9	79.2
2003–2007	22.6	43.9	143.2	6.2	87.2	155.8	7.0	13.1	108.5	0.8	13.8	100.6
2008–2012	15.8	36.3	115.9	2.4	49.1	101.0	8.4	14.1	121.5	1.0	16.3	109.3
	15–24			55–64			15–24			55–64		
1983–1987	1.4	15.6	111.8	3.8	49.8	117.3	0.4	6.6	96.1	1.8	21.8	110.5
1988–1992	1.0	11.4	114.9	3.6	44.4	125.0	0.2	4.9	93.4	1.6	19.5	109.0
1993–1997	0.2	9.6	85.0	5.2	58.9	146.3	0.2	5.0	96.0	1.4	17.4	109.3
1998–2002	1.2	18.3	115.9	4.0	65.5	103.1	0.0	5.9	84.4	0.8	16.8	89.3
2003–2007	0.8	18.7	107.1	4.8	70.1	121.5	0.4	9.7	107.9	2.0	20.0	119.6
2008–2012	0.4	20.2	95.2	4.2	57.2	117.8	0.2	9.5	98.1	2.2	19.3	129.6
	25–34			65–74			25–34			65–74		
1983–1987	1.2	21.4	83.7	2.0	45.9	104.8	1.0	11.9	110.8	1.6	31.1	99.4
1988–1992	0.6	15.8	82.6	1.8	38.7	109.8	1.2	11.6	134.3	2.6	37.7	144.5
1993–1997	1.2	21.1	107.8	2.4	40.0	125.5	0.0	6.6	79.9	2.0	26.1	134.1
1998–2002	1.0	25.1	93.1	2.6	46.3	101.8	0.6	11.8	101.9	2.6	29.3	139.7
2003–2007	1.2	29.6	95.5	3.6	54.1	126.2	0.4	12.3	94.4	1.0	17.6	94.6
2008–2012	1.8	36.2	111.0	2.4	43.0	108.8	1.0	16.8	117.0	1.4	19.2	107.8
	35–44			>74 y/o			35–44			>74 y/o		
1983–1987	4.0	41.5	130.4	2.0	78.0	99.6	1.2	12.6	107.0	2.4	56.5	98.0
1988–1992	0.8	18.4	77.5	1.4	61.9	96.0	0.6	9.3	96.3	1.6	46.4	96.9
1993–1997	1.8	27.2	110.8	2.2	67.9	130.4	0.4	8.0	94.1	2.6	51.6	150.0
1998–2002	2.2	38.2	108.6	4.8	100.3	190.2	0.4	9.7	94.0	2.8	44.3	142.6
2003–2007	2.6	47.0	117.3	3.4	65.1	144.6	0.8	13.6	107.4	1.6	23.7	105.6
2008–2012	1.8	38.0	102.1	2.8	49.7	119.8	0.4	12.8	94.5	2.0	23.1	120.0

Secondary Medical Zone ID: 019

	Male						Female					
	Suicide			Suicide			Suicide			Suicide		
	Num per year	Rate /100,000	×100	Num per year	Rate /100,000	×100	Num per year	Rate /100,000	×100	Num per year	Rate /100,000	×100
	Total (>10 y/o)			45-54			Total (>10 y/o)			45-54		
1983-1987	58.8	34.3	106.5	17.4	60.9	120.4	26.2	14.3	91.6	2.6	12.5	72.4
1988-1992	43.0	26.4	110.5	10.8	42.3	123.3	27.6	14.1	117.7	5.0	17.3	118.5
1993-1997	42.2	25.3	103.1	9.0	35.5	97.0	33.4	14.4	152.8	5.4	16.8	129.8
1998-2002	68.8	39.3	112.2	15.8	58.1	103.8	27.4	12.9	108.1	4.0	14.5	96.2
2003-2007	68.6	40.2	110.8	17.2	67.9	121.3	30.6	13.6	120.4	4.8	16.8	122.3
2008-2012	59.6	36.4	104.2	11.0	48.8	100.5	29.0	14.1	114.8	4.6	18.0	120.5
	15-24			55-64			15-24			55-64		
1983-1987	6.0	18.7	134.2	7.8	39.6	93.1	1.8	6.7	98.7	4.4	20.8	105.6
1988-1992	2.2	9.8	98.8	8.6	38.2	107.5	1.0	4.7	89.1	5.8	23.1	129.2
1993-1997	2.4	11.3	99.8	7.0	33.1	82.1	0.8	4.4	83.8	8.6	29.3	184.2
1998-2002	4.0	18.7	118.1	13.6	61.1	96.2	1.6	7.6	107.8	4.4	18.1	96.2
2003-2007	3.0	17.8	102.3	18.0	70.8	122.7	1.4	8.7	96.5	6.4	20.7	123.4
2008-2012	4.2	25.1	118.5	12.2	47.2	97.1	2.0	11.7	120.4	5.2	16.9	113.2
	25-34			65-74			25-34			65-74		
1983-1987	5.6	21.0	82.0	4.8	41.2	94.1	3.0	10.8	100.5	4.6	31.4	100.6
1988-1992	4.8	20.2	105.6	5.0	38.9	110.4	2.2	8.9	102.7	4.4	28.5	109.2
1993-1997	4.8	20.9	107.3	6.2	37.8	118.5	0.8	5.7	68.0	6.6	32.3	165.9
1998-2002	9.0	35.3	130.7	7.2	40.0	88.0	2.8	11.7	100.8	4.6	22.1	105.1
2003-2007	7.4	32.6	105.3	5.6	31.4	73.2	2.0	10.7	82.0	6.4	25.1	134.7
2008-2012	6.2	32.2	99.0	7.0	36.7	93.0	2.8	14.5	100.9	5.4	20.9	117.2
	35-44			>74 y/o			35-44			>74 y/o		
1983-1987	11.0	35.9	112.8	6.0	77.5	98.9	3.6	11.7	99.1	6.2	50.6	87.6
1988-1992	5.6	20.8	87.8	6.0	79.4	123.0	3.0	9.9	103.0	6.2	57.7	120.7
1993-1997	5.2	21.4	87.2	7.4	81.0	155.6	3.2	10.8	126.7	8.0	58.4	169.6
1998-2002	10.4	42.5	120.8	8.6	76.0	144.1	2.2	9.7	93.7	7.8	45.0	145.0
2003-2007	11.0	47.4	118.2	6.4	45.8	101.8	2.2	10.8	85.7	7.2	32.1	143.2
2008-2012	12.0	47.1	126.5	7.0	40.0	96.5	2.4	11.7	86.5	6.4	23.5	122.3

Secondary Medical Zone ID: 020

	Male						Female					
	Suicide			Suicide			Suicide			Suicide		
	Num per year	Rate /100,000	×100	Num per year	Rate /100,000	×100	Num per year	Rate /100,000	×100	Num per year	Rate /100,000	×100
	Total (>10 y/o)			45–54			Total (>10 y/o)			45–54		
1983–1987	42.8	31.2	94.0	13.6	55.3	109.4	23.2	15.1	98.4	3.4	16.0	92.6
1988–1992	37.4	27.8	118.8	8.6	39.4	114.7	18.6	12.7	101.9	4.2	17.0	116.6
1993–1997	34.2	25.8	105.3	8.4	38.0	103.8	19.0	11.7	114.5	4.4	16.2	125.5
1998–2002	53.0	39.3	111.4	17.0	72.1	128.7	17.8	12.0	93.8	2.0	11.5	76.4
2003–2007	57.0	42.0	122.9	13.0	66.1	118.2	21.6	13.5	113.5	2.6	13.3	97.4
2008–2012	49.2	39.7	118.5	10.8	62.6	128.8	20.6	13.9	111.6	3.8	19.0	127.4
	15–24			55–64			15–24			55–64		
1983–1987	3.8	14.3	102.8	6.2	39.7	93.5	1.0	5.4	79.7	3.2	19.4	98.4
1988–1992	2.6	11.3	114.4	7.6	41.6	117.2	0.4	3.6	68.5	4.6	22.6	126.6
1993–1997	0.8	7.9	69.5	8.0	43.0	106.9	0.6	4.2	81.1	4.2	19.6	123.3
1998–2002	1.8	13.5	85.3	12.4	67.5	106.2	0.4	5.1	72.2	2.8	16.1	85.8
2003–2007	2.8	19.2	109.8	16.2	78.7	136.3	0.8	7.6	84.7	3.6	16.7	99.9
2008–2012	2.6	22.2	104.6	11.2	53.6	110.4	0.6	7.9	81.8	3.4	15.1	101.1
	25–34			65–74			25–34			65–74		
1983–1987	4.6	20.1	78.6	4.0	44.7	102.1	4.2	14.7	136.4	3.4	30.4	97.3
1988–1992	4.0	20.4	106.9	2.4	29.8	84.6	1.4	7.8	89.6	2.8	24.7	94.6
1993–1997	3.0	18.2	92.9	3.4	30.6	95.8	1.2	7.4	89.3	3.8	25.1	128.5
1998–2002	5.2	28.9	107.1	4.4	36.1	79.5	2.0	11.4	98.2	3.8	23.0	109.4
2003–2007	6.6	38.0	122.6	7.0	45.5	106.2	3.0	15.7	121.1	4.2	22.0	118.0
2008–2012	5.6	38.4	118.0	5.2	36.6	92.7	2.4	16.1	112.4	4.0	20.1	113.1
	35–44			>74 y/o			35–44			>74 y/o		
1983–1987	9.0	32.9	103.3	1.2	37.2	47.4	2.6	10.5	88.7	5.2	56.4	97.8
1988–1992	7.6	29.3	123.6	4.2	81.8	126.9	2.2	9.1	93.9	2.8	40.6	84.8
1993–1997	5.6	26.2	107.0	4.8	77.9	149.6	1.6	7.9	92.4	3.2	35.8	104.2
1998–2002	9.0	47.0	133.5	3.2	48.6	92.2	2.6	12.3	119.4	4.2	35.0	112.6
2003–2007	6.8	42.7	106.4	4.6	48.9	108.6	2.4	13.4	106.4	4.8	30.1	134.1
2008–2012	8.8	48.4	130.1	5.0	42.4	102.1	2.6	14.7	108.2	3.8	20.3	105.6

Secondary Medical Zone ID: 021

	Male						Female					
	Suicide			Suicide			Suicide			Suicide		
	Num per year	Rate /100,000	×100	Num per year	Rate /100,000	×100	Num per year	Rate /100,000	×100	Num per year	Rate /100,000	×100
	Total (>10 y/o)			45-54			Total (>10 y/o)			45-54		
1983-1987	13.6	32.6	103.3	3.6	51.0	100.9	6.6	15.3	101.8	1.0	16.6	96.4
1988-1992	11.2	25.6	112.1	1.8	31.5	91.7	6.6	13.6	112.2	1.2	15.5	106.3
1993-1997	10.0	24.9	99.7	1.6	31.5	86.0	5.8	11.3	110.9	0.0	9.5	73.9
1998-2002	17.8	38.2	115.9	3.4	52.6	94.0	6.2	12.7	103.7	1.0	14.9	99.2
2003-2007	18.0	40.3	118.2	6.0	80.2	143.5	6.8	13.2	110.8	1.4	15.9	116.3
2008-2012	20.8	40.4	137.3	3.6	56.0	115.3	7.0	13.6	112.2	0.6	14.0	93.8
	15-24			55-64			15-24			55-64		
1983-1987	1.4	16.9	121.3	3.0	51.7	121.8	0.6	7.3	106.7	1.8	24.9	126.3
1988-1992	0.8	10.8	109.6	2.2	37.9	106.8	0.2	4.9	92.6	1.0	18.2	101.9
1993-1997	1.0	12.9	114.4	2.4	41.7	103.7	0.2	4.9	94.7	1.8	20.9	131.3
1998-2002	0.4	14.2	89.7	4.2	71.1	111.9	0.6	8.1	115.3	0.2	14.8	78.5
2003-2007	0.4	15.9	91.3	3.6	60.3	104.5	0.2	8.2	91.5	1.0	16.6	98.9
2008-2012	2.0	29.1	137.1	5.6	65.2	134.3	0.6	11.5	118.1	1.0	15.0	100.7
	25-34			65-74			25-34			65-74		
1983-1987	2.0	25.8	100.8	1.2	45.2	103.3	1.0	11.6	107.7	0.4	24.6	78.7
1988-1992	1.6	21.5	112.4	1.0	35.7	101.3	0.6	8.8	102.0	1.8	35.9	137.5
1993-1997	2.4	27.0	138.3	0.8	28.3	88.6	0.2	7.2	86.6	0.8	19.5	100.3
1998-2002	1.6	27.2	100.6	3.8	63.0	138.4	0.4	10.6	91.8	1.4	24.2	115.3
2003-2007	3.0	39.4	127.1	1.4	38.3	89.3	1.2	15.3	117.9	0.8	17.7	95.3
2008-2012	3.0	41.0	125.8	1.6	38.8	98.3	0.8	15.1	105.4	1.4	19.9	111.9
	35-44			>74 y/o			35-44			>74 y/o		
1983-1987	1.4	25.2	79.2	1.0	76.0	97.1	0.6	10.7	90.5	1.2	59.1	102.4
1988-1992	1.0	18.9	79.9	2.6	108.5	168.2	0.4	8.6	88.6	1.4	56.5	118.1
1993-1997	1.0	19.9	81.2	0.8	50.3	96.6	0.6	8.6	101.4	2.2	58.4	169.7
1998-2002	3.0	42.5	120.5	1.4	58.6	111.1	0.4	9.4	91.1	2.2	47.1	151.7
2003-2007	1.8	36.0	89.7	1.6	50.4	112.0	0.8	13.1	103.7	1.4	27.1	121.0
2008-2012	2.8	41.5	111.5	2.2	50.3	121.2	0.8	13.9	102.6	1.8	25.6	133.1

Secondary Medical Zone ID: 022

	Male						Female					
	Suicide			Suicide			Suicide			Suicide		
	Num per year	Rate /100,000	×100	Num per year	Rate /100,000	×100	Num per year	Rate /100,000	×100	Num per year	Rate /100,000	×100
	Total (>10 y/o)			45–54			Total (>10 y/o)			45–54		
1983–1987	54.2	38.7	124.6	13.2	58.9	116.4	23.2	14.5	94.0	4.6	17.9	104.0
1988–1992	42.4	29.1	122.7	9.4	43.6	127.1	22.8	12.6	98.3	4.4	16.8	114.8
1993–1997	49.0	30.9	132.8	11.6	48.2	131.8	19.4	10.5	94.4	3.4	13.3	103.3
1998–2002	80.2	48.3	147.6	21.0	83.1	148.5	24.4	12.7	99.9	5.4	18.5	123.0
2003–2007	88.4	54.4	163.7	23.6	103.5	185.1	21.0	11.3	92.6	3.6	14.6	106.7
2008–2012	61.8	40.0	126.2	11.0	56.1	115.5	18.8	11.5	88.2	1.8	11.3	75.8
	15–24			55–64			15–24			55–64		
1983–1987	5.6	22.1	158.4	9.0	49.1	115.4	2.4	8.7	127.1	3.8	18.7	94.6
1988–1992	3.0	12.1	122.1	7.6	36.9	103.9	1.8	6.6	124.5	4.8	19.3	108.2
1993–1997	1.8	9.9	87.5	14.0	61.8	153.6	1.6	6.1	117.1	3.8	15.7	99.0
1998–2002	3.4	16.5	104.4	23.4	109.6	172.4	0.8	5.5	77.7	5.4	21.3	113.4
2003–2007	2.8	16.6	95.1	20.8	91.0	157.7	0.2	4.7	51.9	4.8	18.4	109.8
2008–2012	2.0	16.8	79.0	16.4	66.0	135.8	1.4	9.4	96.6	3.2	13.7	91.7
	25–34			65–74			25–34			65–74		
1983–1987	6.6	29.2	114.3	4.8	46.4	106.0	1.4	7.8	72.7	4.6	32.3	103.3
1988–1992	7.0	31.5	164.7	3.2	29.5	83.7	1.4	7.6	87.5	3.0	19.9	76.4
1993–1997	5.2	25.6	131.2	5.0	32.8	102.8	2.2	9.8	117.4	4.0	19.9	102.2
1998–2002	7.6	36.7	135.8	11.4	59.1	130.0	2.8	13.1	113.2	3.2	16.1	76.7
2003–2007	8.2	41.8	134.7	11.6	59.4	138.4	1.6	10.9	83.8	4.4	18.9	101.3
2008–2012	7.2	40.4	124.0	10.4	54.9	139.0	1.4	11.7	81.9	3.2	15.9	89.6
	35–44			>74 y/o			35–44			>74 y/o		
1983–1987	11.4	44.9	141.1	3.4	70.7	90.3	3.2	12.3	103.9	3.2	40.8	70.8
1988–1992	7.2	28.5	120.2	4.8	71.1	110.2	2.8	10.3	106.7	4.6	40.4	84.4
1993–1997	6.8	29.6	120.7	4.6	59.6	114.4	1.4	7.0	82.4	3.0	22.6	65.8
1998–2002	9.6	45.3	128.7	3.6	43.1	81.7	1.8	9.2	89.1	5.0	28.4	91.4
2003–2007	14.8	72.5	180.9	6.4	51.6	114.5	1.0	8.7	68.7	5.2	23.0	102.5
2008–2012	8.2	43.2	116.1	6.4	43.1	103.8	2.0	12.2	89.7	5.8	21.0	109.0

Secondary Medical Zone ID: 023

	Male						Female					
	Suicide			Suicide			Suicide			Suicide		
	Num per year	Rate /100,000	×100	Num per year	Rate /100,000	×100	Num per year	Rate /100,000	×100	Num per year	Rate /100,000	×100
	Total (>10 y/o)			45–54			Total (>10 y/o)			45–54		
1983–1987	64.8	43.5	145.1	17.4	72.2	142.8	31.4	18.3	128.7	4.2	16.9	98.1
1988–1992	51.4	32.0	140.9	13.8	54.4	158.5	29.8	15.4	129.8	5.8	19.4	132.8
1993–1997	53.4	31.2	133.7	11.8	43.5	118.9	24.4	11.7	117.4	3.2	12.3	94.9
1998–2002	77.8	43.8	129.5	16.8	59.9	107.0	35.4	14.8	136.5	6.4	19.5	129.8
2003–2007	91.0	50.0	149.5	21.4	81.4	145.5	30.2	13.8	121.7	3.8	14.3	104.6
2008–2012	74.8	41.9	133.8	16.2	68.1	140.1	27.8	13.1	114.2	1.8	10.8	72.4
	15–24			55–64			15–24			55–64		
1983–1987	3.8	15.6	111.6	11.2	61.6	144.9	2.2	8.2	120.2	6.4	28.1	142.3
1988–1992	3.0	11.0	111.6	8.0	39.3	110.8	1.0	4.8	91.7	5.0	20.5	115.1
1993–1997	3.8	13.6	120.1	9.6	44.5	110.5	0.2	3.1	59.3	3.8	15.9	99.7
1998–2002	3.4	15.6	99.0	20.0	86.4	136.0	1.0	6.2	88.2	5.0	19.8	105.3
2003–2007	3.8	19.1	109.6	20.8	80.1	138.7	2.0	10.6	118.0	3.6	14.9	89.3
2008–2012	3.8	22.9	107.9	16.4	58.4	120.2	1.6	10.5	107.9	4.2	15.0	100.4
	25–34			65–74			25–34			65–74		
1983–1987	8.8	32.6	127.4	5.2	54.4	124.3	2.8	10.7	99.4	6.0	44.5	142.4
1988–1992	6.0	25.0	131.1	4.8	41.5	117.9	2.0	8.7	100.2	7.8	45.1	172.8
1993–1997	5.0	22.1	113.1	6.0	39.8	124.7	2.4	9.7	116.5	5.8	28.3	145.2
1998–2002	8.6	34.9	129.2	10.0	54.6	120.0	1.6	9.3	80.7	9.0	35.8	170.4
2003–2007	8.6	38.4	123.9	13.4	64.8	151.1	2.4	12.4	95.2	6.4	24.9	133.7
2008–2012	7.0	37.1	114.0	10.8	51.6	130.7	1.8	12.4	86.8	4.8	19.7	110.8
	35–44			>74 y/o			35–44			>74 y/o		
1983–1987	13.6	46.3	145.6	4.6	105.2	134.3	3.2	11.6	97.9	6.4	88.9	154.2
1988–1992	11.4	36.5	154.1	4.2	73.9	114.6	2.4	8.9	92.2	5.6	57.6	120.3
1993–1997	9.6	34.4	140.3	7.6	99.3	190.7	1.6	7.1	83.2	7.2	54.8	159.2
1998–2002	13.2	52.2	148.1	5.6	63.3	120.0	2.0	9.3	89.8	10.4	59.4	191.3
2003–2007	14.6	61.5	153.4	7.8	62.7	139.2	2.8	12.5	99.3	9.2	40.8	181.7
2008–2012	10.2	43.4	116.6	10.2	62.5	150.5	3.4	14.5	107.1	10.2	35.4	184.2

Secondary Medical Zone ID: 024

	Male						Female					
	Suicide			Suicide			Suicide			Suicide		
	Num per year	Rate /100,000	×100	Num per year	Rate /100,000	×100	Num per year	Rate /100,000	×100	Num per year	Rate /100,000	×100
	Total (>10 y/o)			45-54			Total (>10 y/o)			45-54		
1983-1987	58.2	39.8	131.2	17.0	71.7	141.8	26.2	15.7	106.6	5.4	19.7	114.6
1988-1992	43.8	29.0	124.9	11.6	48.2	140.4	21.8	12.4	97.0	4.4	16.2	111.0
1993-1997	45.6	28.4	118.6	12.0	44.7	122.1	16.6	9.5	83.6	1.6	8.7	67.4
1998-2002	70.0	41.4	120.1	18.6	67.3	120.2	21.4	11.8	88.3	2.4	11.1	73.8
2003-2007	86.4	49.4	147.1	22.0	87.0	155.6	21.0	11.6	89.9	4.0	14.8	107.9
2008-2012	64.2	40.2	122.0	15.8	68.1	140.1	26.2	13.9	108.7	4.0	16.4	110.1
	15-24			55-64			15-24			55-64		
1983-1987	4.4	18.4	132.0	10.2	56.5	133.0	1.2	5.8	85.1	6.8	28.8	146.0
1988-1992	1.4	8.2	82.3	8.8	43.3	121.8	1.4	5.6	106.4	4.6	19.1	107.2
1993-1997	3.2	12.7	112.7	11.0	50.6	125.8	0.8	4.3	82.1	3.8	15.7	99.0
1998-2002	3.2	15.3	96.6	18.8	83.9	132.1	1.0	5.9	83.9	4.4	18.3	97.2
2003-2007	3.0	17.2	98.3	23.4	91.3	158.2	1.4	8.3	92.8	4.4	16.6	99.0
2008-2012	2.2	18.4	86.6	16.6	62.0	127.7	2.0	11.7	120.7	4.2	15.0	100.8
	25-34			65-74			25-34			65-74		
1983-1987	7.8	29.8	116.5	3.8	42.3	96.6	3.6	12.4	115.0	2.2	22.2	71.0
1988-1992	5.8	25.0	130.9	4.2	37.4	106.2	1.0	6.2	71.0	4.0	26.3	100.8
1993-1997	5.8	25.4	129.9	5.0	34.6	108.4	1.8	8.2	98.2	4.0	20.9	107.2
1998-2002	6.8	30.1	111.5	7.2	43.4	95.4	3.2	13.0	112.1	4.2	19.8	94.2
2003-2007	8.0	36.4	117.5	9.6	51.5	120.0	2.2	11.5	88.7	4.0	17.9	96.2
2008-2012	7.6	39.4	121.0	7.6	41.8	105.8	2.0	12.8	89.1	5.4	21.5	120.6
	35-44			>74 y/o			35-44			>74 y/o		
1983-1987	11.4	39.9	125.2	3.4	81.4	103.9	3.4	11.7	99.1	3.6	51.7	89.6
1988-1992	9.8	33.5	141.4	2.2	46.3	71.7	3.0	10.0	103.4	3.4	36.9	77.1
1993-1997	6.2	25.2	102.9	2.4	39.9	76.7	1.8	7.5	88.1	2.8	24.5	71.2
1998-2002	11.0	45.8	130.0	4.4	52.3	99.2	2.8	11.1	107.5	3.4	23.0	74.0
2003-2007	13.6	59.4	148.2	6.6	55.8	123.8	1.4	9.1	72.0	3.6	18.3	81.8
2008-2012	10.2	44.6	119.8	4.2	33.6	80.9	3.6	15.0	110.6	4.8	18.8	97.8

Secondary Medical Zone ID: 025

	Male						Female					
	Suicide			Suicide			Suicide			Suicide		
	Num per year	Rate /100,000	×100	Num per year	Rate /100,000	×100	Num per year	Rate /100,000	×100	Num per year	Rate /100,000	×100
	Total (>10 y/o)			45–54			Total (>10 y/o)			45–54		
1983–1987	30.8	37.7	127.5	9.4	69.8	138.1	15.2	16.4	109.8	1.8	15.2	88.3
1988–1992	22.2	27.7	118.8	4.0	36.6	106.7	10.2	12.0	86.7	2.4	16.5	113.1
1993–1997	29.8	31.7	148.1	4.6	39.9	108.9	11.2	11.0	102.8	2.0	14.4	111.3
1998–2002	38.6	43.2	136.0	8.2	65.3	116.8	15.0	13.7	114.4	1.8	14.9	99.2
2003–2007	48.2	54.0	171.4	12.8	102.2	182.7	13.8	12.7	112.9	1.6	13.8	100.6
2008–2012	31.6	38.8	127.9	7.2	65.3	134.3	10.6	12.3	98.8	1.0	13.0	87.3
	15–24			55–64			15–24			55–64		
1983–1987	2.6	19.6	140.6	6.2	55.6	130.8	1.8	10.2	148.7	2.4	20.5	104.1
1988–1992	1.2	10.9	109.9	4.2	36.9	104.0	0.6	5.6	105.4	1.2	13.7	76.9
1993–1997	1.4	12.6	111.8	5.4	45.8	113.7	0.4	5.0	96.1	1.2	12.5	78.3
1998–2002	1.0	14.4	90.9	10.6	92.0	144.7	2.0	11.8	168.5	2.2	18.6	98.7
2003–2007	1.0	16.2	92.9	9.6	81.0	140.4	0.2	7.0	78.0	3.0	20.0	119.6
2008–2012	1.0	19.9	94.1	7.2	57.6	118.6	0.2	7.9	81.6	1.4	13.8	92.3
	25–34			65–74			25–34			65–74		
1983–1987	2.0	20.8	81.2	3.6	54.1	123.5	1.2	10.2	94.8	2.2	29.3	93.7
1988–1992	3.0	25.6	134.1	3.0	40.2	114.2	0.8	8.2	94.9	2.6	27.9	106.9
1993–1997	3.6	29.7	152.0	6.2	57.6	180.6	1.2	9.8	117.6	2.0	19.0	97.3
1998–2002	3.4	33.0	122.2	7.8	65.4	143.9	1.2	12.3	106.4	3.4	24.7	117.3
2003–2007	4.6	43.6	140.7	7.2	61.4	143.0	1.0	12.9	99.4	3.0	21.3	114.4
2008–2012	1.4	28.2	86.7	4.2	44.1	111.6	0.8	13.7	95.9	1.4	15.5	86.9
	35–44			>74 y/o			35–44			>74 y/o		
1983–1987	4.6	36.7	115.1	2.2	73.5	93.8	1.6	12.1	102.6	4.2	79.6	138.0
1988–1992	4.8	33.0	139.3	2.0	55.6	86.2	1.0	9.0	93.0	1.6	29.1	60.8
1993–1997	3.8	30.3	123.6	4.6	84.5	162.2	1.2	9.2	107.8	3.2	37.8	109.9
1998–2002	5.0	44.5	126.5	2.6	49.8	94.5	1.0	10.0	97.1	3.4	33.4	107.5
2003–2007	8.8	78.2	195.1	4.2	55.2	122.5	0.6	10.6	83.6	4.4	31.9	142.1
2008–2012	4.6	45.8	123.1	6.0	59.9	144.4	1.2	13.8	101.6	4.6	27.0	140.6

Secondary Medical Zone ID: 026

	Male						Female					
	Suicide			Suicide			Suicide			Suicide		
	Num per year	Rate /100,000	×100	Num per year	Rate /100,000	×100	Num per year	Rate /100,000	×100	Num per year	Rate /100,000	×100
	Total (>10 y/o)			45–54			Total (>10 y/o)			45–54		
1983–1987	40.8	43.8	154.5	9.8	69.1	136.6	19.0	18.4	131.0	3.4	20.4	118.6
1988–1992	35.4	34.4	164.9	7.4	50.9	148.1	22.0	16.9	157.4	4.2	21.3	146.0
1993–1997	35.4	32.8	152.0	8.2	49.7	135.7	22.6	15.1	173.6	2.6	15.1	117.1
1998–2002	54.0	47.8	154.6	11.2	66.7	119.3	19.4	14.6	130.2	2.8	16.5	109.8
2003–2007	64.0	52.8	182.1	12.6	81.5	145.7	19.8	14.5	135.4	3.2	17.8	129.6
2008–2012	49.4	43.3	151.2	10.0	70.6	145.3	17.6	13.6	124.0	1.4	13.6	91.5
	15–24			55–64			15–24			55–64		
1983–1987	1.8	14.3	102.8	6.8	58.8	138.4	1.4	8.5	124.5	3.0	22.8	115.7
1988–1992	1.6	11.0	111.5	8.6	60.9	171.4	0.8	5.8	110.4	4.0	23.8	133.4
1993–1997	1.2	10.7	94.3	6.2	48.1	119.6	0.4	4.6	88.7	3.4	20.6	129.9
1998–2002	1.2	13.5	85.6	15.0	108.0	170.0	0.2	5.4	76.3	3.4	21.9	116.3
2003–2007	3.2	23.4	134.0	15.4	99.2	171.8	0.6	8.2	90.6	3.2	19.3	115.2
2008–2012	1.6	20.5	96.6	12.0	68.5	141.1	0.2	7.2	74.7	3.0	17.0	114.1
	25–34			65–74			25–34			65–74		
1983–1987	6.0	35.4	138.5	5.0	71.5	163.4	2.0	12.1	112.1	3.6	42.1	134.7
1988–1992	5.0	32.0	167.6	4.2	51.9	147.4	1.0	8.4	97.0	4.6	42.3	162.2
1993–1997	3.2	23.6	121.0	7.6	68.2	213.7	0.8	7.9	94.4	6.4	43.6	223.6
1998–2002	4.0	29.6	109.6	7.8	65.2	143.4	1.8	13.2	113.5	4.6	29.9	142.3
2003–2007	6.2	42.2	136.2	11.4	85.8	199.9	0.8	10.9	83.5	3.0	21.0	112.9
2008–2012	4.6	37.7	115.6	7.4	58.4	147.8	1.2	13.9	97.0	3.8	23.7	133.1
	35–44			>74 y/o			35–44			>74 y/o		
1983–1987	7.2	42.8	134.6	3.8	125.5	160.2	1.0	9.5	80.5	4.6	100.6	174.4
1988–1992	4.4	26.3	110.9	4.2	102.2	158.5	2.0	10.9	113.1	5.4	83.8	175.2
1993–1997	5.2	31.8	129.9	3.8	79.1	151.9	2.4	12.2	143.4	6.6	76.2	221.4
1998–2002	8.6	56.1	159.3	6.2	95.4	181.0	2.0	12.8	123.7	4.6	44.0	141.6
2003–2007	6.0	47.5	118.7	9.2	102.3	227.1	2.4	15.9	125.7	6.6	45.7	203.8
2008–2012	6.2	44.6	119.8	7.4	69.6	167.8	2.2	15.7	115.9	5.8	31.9	165.7

Secondary Medical Zone ID: 027

	Male						Female					
	Suicide			Suicide			Suicide			Suicide		
	Num per year	Rate /100,000	×100	Num per year	Rate /100,000	×100	Num per year	Rate /100,000	×100	Num per year	Rate /100,000	×100
	Total (>10 y/o)			45–54			Total (>10 y/o)			45–54		
1983–1987	14.2	34.8	113.9	4.2	60.4	119.5	7.2	15.6	104.8	0.8	15.8	91.5
1988–1992	14.4	29.0	138.4	2.6	39.1	113.9	7.0	13.1	109.2	1.0	15.0	102.6
1993–1997	15.8	29.8	140.7	3.4	43.1	117.9	6.4	11.2	111.2	0.6	11.9	92.4
1998–2002	23.0	43.0	140.0	6.6	75.1	134.1	7.8	13.1	113.4	1.4	16.4	108.8
2003–2007	23.4	44.8	145.5	8.2	99.7	178.3	6.2	12.8	103.2	1.2	15.1	110.2
2008–2012	18.6	38.4	125.7	3.4	54.2	111.5	5.6	12.6	100.6	1.0	16.1	107.8
	15–24			55–64			15–24			55–64		
1983–1987	0.4	11.9	85.4	2.2	45.2	106.3	0.6	7.7	113.3	1.6	22.7	115.2
1988–1992	0.6	10.2	102.9	2.8	44.2	124.6	0.0	4.3	80.9	1.6	20.7	115.7
1993–1997	0.6	11.3	100.1	3.6	51.9	129.0	0.2	5.0	96.3	1.8	20.5	128.7
1998–2002	0.6	15.2	95.9	6.6	93.1	146.5	0.0	5.9	83.5	1.6	20.9	111.3
2003–2007	0.4	16.1	92.1	5.0	71.7	124.2	0.2	8.4	93.3	1.6	18.9	112.6
2008–2012	1.6	27.1	128.0	3.2	48.5	99.8	0.2	9.3	96.3	1.2	15.6	104.5
	25–34			65–74			25–34			65–74		
1983–1987	1.4	23.3	91.2	2.6	63.8	145.8	0.6	10.1	93.6	1.4	33.8	108.1
1988–1992	1.2	19.9	103.9	1.2	36.7	104.2	0.0	6.6	76.6	1.4	28.3	108.6
1993–1997	1.8	24.4	125.1	2.0	40.7	127.5	0.2	7.5	89.6	1.4	22.7	116.5
1998–2002	1.6	27.7	102.6	2.0	45.1	99.1	0.4	10.9	94.0	1.8	25.3	120.3
2003–2007	3.0	40.5	130.5	2.4	46.3	107.8	0.2	11.1	85.7	1.0	18.3	98.1
2008–2012	2.0	36.1	110.9	2.8	47.1	119.3	0.2	12.6	88.2	1.4	19.8	111.3
	35–44			>74 y/o			35–44			>74 y/o		
1983–1987	2.0	30.1	94.4	1.4	88.2	112.6	0.8	11.6	98.6	1.4	59.2	102.7
1988–1992	3.0	31.4	132.7	3.0	111.1	172.3	0.6	9.3	96.0	2.2	61.8	129.2
1993–1997	2.6	31.5	128.4	1.8	69.3	133.0	0.8	9.5	111.9	1.4	35.5	103.3
1998–2002	3.6	47.9	136.0	2.0	66.0	125.2	0.4	9.6	92.8	2.2	39.8	128.1
2003–2007	2.2	40.9	102.2	2.0	54.0	119.9	1.0	14.2	112.7	1.0	19.3	86.1
2008–2012	2.8	42.1	113.2	2.8	56.0	134.9	0.4	12.6	92.6	1.2	18.5	96.2

Secondary Medical Zone ID: 028

	Male						Female					
	Suicide			Suicide			Suicide			Suicide		
	Num per year	Rate /100,000	×100	Num per year	Rate /100,000	×100	Num per year	Rate /100,000	×100	Num per year	Rate /100,000	×100
	Total (>10 y/o)			45–54			Total (>10 y/o)			45–54		
1983–1987	84.8	44.8	148.2	21.8	74.8	148.0	34.8	17.1	114.7	6.0	18.7	108.5
1988–1992	61.0	29.9	128.5	12.0	40.4	117.6	37.2	15.0	125.6	5.0	15.4	105.6
1993–1997	68.4	30.6	129.2	15.6	44.6	122.0	31.2	11.5	113.8	4.4	12.8	98.8
1998–2002	101.4	42.5	125.5	26.6	70.4	125.8	38.6	13.0	113.7	6.2	16.2	107.3
2003–2007	110.2	46.0	133.1	24.6	70.4	125.9	45.6	14.7	132.5	5.6	15.2	111.2
2008–2012	94.6	40.5	122.2	18.6	57.7	118.8	40.4	13.9	118.0	4.8	14.9	99.7
	15–24			55–64			15–24			55–64		
1983–1987	6.8	20.7	148.4	13.6	58.5	137.7	1.8	6.3	92.0	5.8	22.3	113.0
1988–1992	3.6	10.6	106.7	12.6	46.5	131.0	1.4	4.6	87.6	4.6	16.6	92.9
1993–1997	2.0	7.9	69.7	16.0	56.1	139.2	1.2	4.1	79.4	4.8	15.8	99.5
1998–2002	4.8	15.1	95.4	20.4	72.2	113.6	1.0	4.6	65.4	6.6	20.4	108.6
2003–2007	7.0	21.8	124.8	25.4	76.7	132.9	2.4	8.4	93.5	8.0	21.0	125.6
2008–2012	8.0	27.7	130.8	17.0	48.7	100.3	2.8	10.4	107.6	7.0	17.5	117.6
	25–34			65–74			25–34			65–74		
1983–1987	10.2	29.4	115.0	10.4	78.2	178.7	4.4	12.1	112.4	6.0	37.3	119.5
1988–1992	7.2	22.4	117.3	5.6	38.1	108.1	3.2	9.5	110.1	7.2	35.0	134.1
1993–1997	6.4	20.7	106.1	8.0	39.9	125.0	2.6	8.3	100.1	7.4	28.7	147.1
1998–2002	10.2	30.6	113.2	15.2	62.2	136.8	2.2	8.6	74.4	7.6	26.0	123.8
2003–2007	13.6	40.9	131.7	11.4	47.4	110.4	5.4	15.7	120.6	8.6	26.4	142.0
2008–2012	11.4	38.8	119.2	11.4	45.5	115.1	3.0	12.1	84.5	9.2	26.8	150.7
	35–44			>74 y/o			35–44			>74 y/o		
1983–1987	16.0	44.4	139.5	5.6	97.5	124.5	2.4	8.6	72.6	8.0	90.1	156.1
1988–1992	12.0	31.3	132.1	7.8	94.1	145.9	2.6	8.0	82.8	13.2	98.0	204.9
1993–1997	12.0	32.8	133.8	8.0	79.4	152.6	2.2	7.0	82.2	8.4	49.0	142.4
1998–2002	14.4	42.8	121.6	9.6	74.4	141.1	2.4	8.3	80.4	12.4	54.4	175.2
2003–2007	16.4	51.6	128.8	11.8	67.4	149.7	3.8	12.2	96.8	11.8	39.7	177.1
2008–2012	15.8	47.1	126.7	12.2	56.9	137.1	4.4	13.7	101.1	9.0	24.9	129.2

Secondary Medical Zone ID: 029

	Male						Female					
	Suicide			Suicide			Suicide			Suicide		
	Num per year	Rate /100,000	×100	Num per year	Rate /100,000	×100	Num per year	Rate /100,000	×100	Num per year	Rate /100,000	×100
	Total (>10 y/o)			45–54			Total (>10 y/o)			45–54		
1983–1987	54.2	46.9	165.2	16.4	90.8	179.6	28.8	20.2	149.9	3.8	19.2	111.6
1988–1992	43.2	34.4	161.9	10.2	57.5	167.4	30.6	17.8	168.0	3.0	16.0	109.4
1993–1997	42.8	32.9	148.4	9.0	48.1	131.5	20.0	12.0	127.1	2.6	14.0	108.1
1998–2002	62.0	46.3	145.8	16.4	82.6	147.5	28.2	15.1	145.9	3.2	16.6	110.3
2003–2007	63.4	48.4	148.9	13.8	77.9	139.4	22.4	13.4	125.0	1.8	12.6	91.7
2008–2012	54.8	42.4	139.0	10.6	65.1	134.0	23.4	14.5	130.0	2.2	15.3	102.8
	15–24			55–64			15–24			55–64		
1983–1987	2.4	16.7	119.8	10.8	67.7	159.2	1.0	7.3	107.3	4.8	25.4	128.6
1988–1992	1.8	11.1	112.5	8.0	47.0	132.2	0.8	5.7	107.2	6.6	28.6	159.9
1993–1997	1.0	9.3	82.4	11.0	61.8	153.5	0.2	3.9	73.8	3.2	17.0	107.2
1998–2002	3.4	19.9	125.8	12.0	76.6	120.5	0.6	6.2	88.7	4.2	21.7	115.5
2003–2007	2.4	18.7	107.4	12.6	73.5	127.4	0.6	7.5	83.4	4.6	21.5	128.1
2008–2012	4.6	31.8	150.0	13.0	65.7	135.3	0.8	9.2	94.8	2.2	13.8	92.7
	25–34			65–74			25–34			65–74		
1983–1987	4.4	27.5	107.7	5.0	56.8	129.6	1.6	10.6	98.9	7.4	56.4	180.4
1988–1992	3.8	24.7	129.3	5.8	52.6	149.3	1.6	10.1	116.8	8.0	51.6	197.8
1993–1997	2.8	20.3	103.7	5.6	42.4	133.0	0.8	7.5	90.5	5.8	31.9	163.8
1998–2002	6.4	36.4	134.9	8.4	56.1	123.5	1.6	11.8	102.0	6.6	32.4	154.0
2003–2007	5.6	35.0	112.9	9.4	61.0	142.2	1.6	12.6	97.1	5.4	26.2	141.0
2008–2012	4.8	35.1	107.7	6.2	44.8	113.5	2.0	15.2	106.3	4.4	22.7	127.3
	35–44			>74 y/o			35–44			>74 y/o		
1983–1987	9.4	49.1	154.4	5.6	123.2	157.3	3.2	15.1	127.7	7.0	103.1	178.7
1988–1992	6.0	30.8	130.0	7.2	118.1	183.1	1.8	10.0	103.3	8.8	92.8	194.0
1993–1997	8.2	42.0	171.2	5.2	76.1	146.0	1.0	7.5	87.7	6.4	53.8	156.4
1998–2002	7.4	45.2	128.4	8.0	89.5	169.9	1.8	11.4	110.6	10.2	64.5	207.6
2003–2007	10.8	66.3	165.4	8.6	74.2	164.7	1.4	11.7	92.6	7.0	35.7	159.2
2008–2012	6.8	42.2	113.5	8.8	63.7	153.5	3.0	16.9	124.7	8.8	35.7	185.5

Secondary Medical Zone ID: 030

	Male						Female					
	Suicide			Suicide			Suicide			Suicide		
	Num per year	Rate /100,000	×100	Num per year	Rate /100,000	×100	Num per year	Rate /100,000	×100	Num per year	Rate /100,000	×100
	Total (>10 y/o)			45–54			Total (>10 y/o)			45–54		
1983–1987	28.6	38.6	134.6	9.0	74.8	147.9	14.2	16.7	117.4	2.0	17.1	99.2
1988–1992	24.0	30.3	138.0	5.6	48.8	142.0	12.2	13.3	109.2	2.2	16.8	115.0
1993–1997	22.4	28.8	122.7	4.8	42.3	115.5	9.0	10.4	95.6	1.6	13.8	106.8
1998–2002	32.8	40.3	122.6	9.0	71.7	128.1	15.8	14.0	128.0	2.0	16.4	108.6
2003–2007	39.8	47.2	147.3	11.0	91.3	163.2	12.8	12.2	114.6	0.6	11.0	80.6
2008–2012	29.2	37.7	119.3	7.4	67.4	138.7	11.6	12.5	109.3	0.6	12.1	81.1
	15–24			55–64			15–24			55–64		
1983–1987	2.2	20.0	143.6	5.0	51.0	120.0	0.0	5.0	72.9	2.4	21.2	107.5
1988–1992	0.8	9.9	100.4	3.6	35.6	100.1	0.4	5.2	99.3	2.0	17.5	98.1
1993–1997	1.2	12.1	106.8	3.6	37.8	94.0	0.0	3.8	73.0	1.0	12.4	78.0
1998–2002	1.0	14.6	92.4	6.8	69.7	109.7	0.6	7.3	103.8	2.4	20.4	108.2
2003–2007	1.0	16.4	94.1	9.0	79.2	137.1	0.6	9.1	101.0	2.0	17.7	105.5
2008–2012	1.4	21.7	102.5	8.0	61.7	127.0	0.2	7.9	81.3	1.4	14.3	95.8
	25–34			65–74			25–34			65–74		
1983–1987	3.6	31.6	123.6	2.2	42.1	96.2	1.0	10.6	98.6	4.0	45.3	145.1
1988–1992	3.0	27.2	142.6	3.2	44.4	126.0	0.6	8.2	94.5	2.6	28.9	110.9
1993–1997	2.6	25.2	129.1	3.2	37.6	118.0	1.2	10.4	124.7	2.4	22.1	113.6
1998–2002	3.2	31.5	116.8	6.0	57.6	126.8	0.2	9.1	78.4	4.0	29.1	138.7
2003–2007	4.6	41.6	134.0	4.6	48.0	111.8	0.4	10.6	81.2	3.0	22.7	122.0
2008–2012	2.8	33.8	103.8	2.8	36.9	93.5	0.6	12.7	88.8	2.4	20.2	113.8
	35–44			>74 y/o			35–44			>74 y/o		
1983–1987	3.8	35.3	110.8	2.8	95.0	121.3	1.6	13.1	111.3	3.2	73.3	127.0
1988–1992	3.8	29.6	125.0	4.0	92.6	143.6	0.8	8.9	92.7	3.6	57.7	120.5
1993–1997	4.8	37.1	151.3	2.2	51.7	99.3	0.4	6.9	80.9	2.4	32.5	94.5
1998–2002	4.2	40.9	116.1	2.6	50.7	96.3	1.6	13.0	125.8	5.0	47.4	152.6
2003–2007	4.8	50.7	126.6	4.8	61.6	136.7	0.0	8.8	69.9	6.2	44.5	198.5
2008–2012	2.4	33.7	90.7	4.4	49.3	118.8	1.2	14.1	104.2	5.2	31.2	162.0

Secondary Medical Zone ID: 031

	Male						Female					
	Suicide			Suicide			Suicide			Suicide		
	Num per year	Rate /100,000	×100	Num per year	Rate /100,000	×100	Num per year	Rate /100,000	×100	Num per year	Rate /100,000	×100
	Total (>10 y/o)			45–54			Total (>10 y/o)			45–54		
1983–1987	24.4	35.0	111.1	4.6	46.6	92.2	17.2	17.7	127.8	1.2	13.9	80.8
1988–1992	19.4	25.9	109.9	3.6	36.5	106.1	17.6	15.5	139.7	1.8	15.6	106.7
1993–1997	21.6	27.7	115.0	5.0	42.7	116.6	10.8	11.0	105.9	1.4	13.3	102.6
1998–2002	38.6	45.1	139.2	9.4	72.8	130.0	15.2	13.5	122.6	1.4	14.4	95.7
2003–2007	32.8	42.6	124.2	6.6	60.5	108.2	12.8	13.0	114.9	1.4	13.8	101.0
2008–2012	29.4	39.9	122.5	5.6	56.9	117.0	13.0	13.7	118.7	2.0	17.8	119.6
	15–24			55–64			15–24			55–64		
1983–1987	1.2	14.5	103.9	4.6	44.6	104.9	0.4	6.4	93.7	4.0	26.9	136.3
1988–1992	0.6	9.2	92.5	5.6	45.9	129.2	0.6	5.9	112.2	3.6	23.0	129.0
1993–1997	0.8	10.7	94.4	3.6	37.7	93.6	0.2	4.5	86.3	2.6	19.0	119.4
1998–2002	1.4	16.3	103.1	7.6	75.3	118.6	0.6	7.3	104.5	1.6	17.6	93.7
2003–2007	0.8	15.7	89.9	7.2	67.0	116.0	0.4	8.3	92.2	2.0	17.9	106.7
2008–2012	2.0	25.8	121.8	7.2	57.1	117.6	0.0	7.1	73.1	2.8	18.8	125.8
	25–34			65–74			25–34			65–74		
1983–1987	3.6	30.8	120.4	2.6	43.6	99.5	2.0	14.6	135.7	4.0	43.4	139.0
1988–1992	2.0	21.1	110.2	2.0	31.2	88.5	2.0	13.4	154.8	4.0	36.8	141.2
1993–1997	1.8	20.9	107.1	3.0	33.4	104.8	1.0	9.7	116.2	1.0	13.1	67.2
1998–2002	5.2	42.8	158.6	4.2	44.6	98.2	1.0	12.2	105.4	3.2	24.3	115.8
2003–2007	3.2	35.8	115.5	3.8	42.8	99.6	1.2	14.2	109.5	2.6	21.0	112.7
2008–2012	3.6	39.8	122.2	2.8	37.2	94.2	0.6	13.1	91.7	2.0	19.0	106.9
	35–44			>74 y/o			35–44			>74 y/o		
1983–1987	5.2	42.5	133.5	2.6	81.0	103.4	2.4	16.1	136.0	3.2	61.9	107.2
1988–1992	3.4	27.2	114.5	2.2	58.2	90.2	1.2	10.2	105.5	4.4	62.8	131.3
1993–1997	4.8	36.5	148.7	2.6	53.4	102.5	0.4	6.9	80.6	4.0	46.0	133.6
1998–2002	6.8	57.6	163.4	3.8	60.3	114.3	0.8	9.9	96.0	6.4	55.1	177.4
2003–2007	6.2	63.1	157.6	4.6	55.9	124.1	0.4	10.5	82.8	4.8	33.9	150.9
2008–2012	4.6	46.9	126.0	3.6	41.4	99.8	1.2	14.5	106.7	4.4	26.3	136.6

Secondary Medical Zone ID: 032

	Male						Female					
	Suicide			Suicide			Suicide			Suicide		
	Num per year	Rate /100,000	×100	Num per year	Rate /100,000	×100	Num per year	Rate /100,000	×100	Num per year	Rate /100,000	×100
	Total (>10 y/o)			45-54			Total (>10 y/o)			45-54		
1983-1987	16.2	37.0	127.9	3.6	55.3	109.3	7.8	15.9	108.0	1.0	16.5	95.7
1988-1992	10.8	26.1	112.8	2.2	36.6	106.6	10.8	15.5	145.0	1.0	14.9	101.8
1993-1997	11.4	26.9	114.3	2.8	41.8	114.2	7.6	11.8	123.8	0.8	12.9	100.2
1998-2002	18.0	39.4	123.3	3.8	61.4	109.7	8.6	13.4	121.3	1.4	17.2	114.0
2003-2007	19.0	43.7	134.3	6.8	99.5	177.9	6.2	12.7	105.5	0.4	12.5	90.9
2008-2012	12.8	35.8	106.4	4.2	66.4	136.6	6.2	13.7	107.9	0.8	15.6	104.6
	15-24			55-64			15-24			55-64		
1983-1987	0.8	14.5	103.7	1.4	36.0	84.6	0.6	7.9	116.0	1.6	21.9	111.1
1988-1992	0.2	8.9	89.4	2.0	35.3	99.5	0.2	5.2	98.7	1.4	19.0	106.3
1993-1997	0.0	9.3	82.2	2.0	38.5	95.5	0.0	4.5	85.4	1.4	17.9	112.7
1998-2002	0.4	14.8	93.7	3.6	65.7	103.4	0.2	6.9	98.2	1.6	20.7	110.2
2003-2007	1.2	21.0	120.4	3.8	64.2	111.2	0.2	8.7	96.9	1.4	18.3	109.4
2008-2012	0.8	22.5	106.1	1.8	43.2	88.8	0.4	10.8	111.4	1.2	16.2	108.9
	25-34			65-74			25-34			65-74		
1983-1987	1.4	26.2	102.4	2.0	53.8	122.9	0.4	10.0	92.4	1.2	30.8	98.6
1988-1992	0.8	18.6	97.6	1.8	42.4	120.4	0.6	9.6	110.3	2.2	35.5	136.0
1993-1997	0.4	16.7	85.5	1.0	28.0	87.7	0.6	9.4	112.8	2.0	26.5	135.9
1998-2002	1.2	28.0	103.5	4.8	67.1	147.5	0.6	12.2	105.6	1.8	24.3	115.8
2003-2007	1.2	31.8	102.4	2.2	43.1	100.5	0.6	13.8	105.8	2.2	24.6	132.3
2008-2012	0.8	31.6	96.9	1.2	34.1	86.4	0.4	14.3	100.1	1.2	18.6	104.4
	35-44			>74 y/o			35-44			>74 y/o		
1983-1987	4.0	47.3	148.6	3.0	125.7	160.4	0.4	10.4	87.9	2.6	80.2	139.0
1988-1992	2.0	28.0	118.2	1.8	76.0	117.8	1.6	13.3	138.0	3.8	86.2	180.2
1993-1997	2.6	35.2	143.5	2.4	76.4	146.6	0.6	9.2	108.2	2.2	45.1	131.1
1998-2002	3.0	47.3	134.4	1.2	47.4	90.0	0.2	9.1	87.8	2.8	43.8	141.2
2003-2007	2.2	45.4	113.2	1.6	43.9	97.6	0.0	10.3	81.4	1.4	21.3	95.0
2008-2012	1.2	35.5	95.4	2.8	50.7	122.3	1.0	15.5	114.6	1.2	17.1	89.1

Secondary Medical Zone ID: 033

	Male						Female					
	Suicide			Suicide			Suicide			Suicide		
	Num per year	Rate /100,000	×100	Num per year	Rate /100,000	×100	Num per year	Rate /100,000	×100	Num per year	Rate /100,000	×100
	Total (>10 y/o)			45–54			Total (>10 y/o)			45–54		
1983–1987	15.6	37.0	131.4	4.0	59.8	118.2	6.4	15.4	103.7	0.8	15.6	90.3
1988–1992	8.2	24.8	101.6	1.4	31.1	90.7	5.4	12.8	100.9	0.4	12.8	87.6
1993–1997	8.4	25.3	102.1	2.4	40.5	110.6	6.0	11.6	116.8	1.2	15.0	116.0
1998–2002	17.4	41.0	135.8	5.2	77.4	138.3	3.4	11.7	83.2	0.4	13.7	90.9
2003–2007	16.8	43.6	142.1	3.6	71.4	127.6	3.6	12.2	91.0	0.4	13.0	94.8
2008–2012	12.0	37.3	117.5	3.0	61.8	127.2	5.2	13.7	108.9	1.2	18.5	124.3
	15–24			55–64			15–24			55–64		
1983–1987	0.4	12.9	92.5	3.2	53.8	126.5	0.0	5.7	82.8	1.2	20.4	103.3
1988–1992	0.2	9.3	94.2	2.0	37.3	105.1	0.4	6.1	115.8	1.0	17.6	98.3
1993–1997	0.6	12.4	109.9	1.4	35.4	88.0	0.2	5.4	102.9	1.0	16.2	102.0
1998–2002	0.2	14.6	92.7	4.4	77.7	122.3	0.2	7.1	101.1	0.0	14.2	75.6
2003–2007	0.8	20.0	114.6	3.8	69.6	120.6	0.2	9.1	100.8	0.8	16.6	99.0
2008–2012	0.6	22.8	107.6	2.2	48.6	100.0	0.4	11.2	115.8	0.6	14.5	97.5
	25–34			65–74			25–34			65–74		
1983–1987	2.2	32.4	126.8	1.6	49.0	112.0	0.6	11.0	102.0	1.2	32.1	102.8
1988–1992	1.4	25.1	131.4	1.2	36.6	103.8	0.0	7.1	81.9	1.4	29.1	111.5
1993–1997	0.8	20.7	105.8	1.4	34.1	106.8	0.2	7.9	95.2	1.0	19.9	102.0
1998–2002	1.0	28.0	103.9	3.6	60.9	133.9	0.2	10.9	93.8	0.8	18.7	89.1
2003–2007	2.6	45.4	146.4	1.6	40.7	94.9	0.4	13.2	101.7	1.2	19.7	106.0
2008–2012	0.6	31.6	97.0	2.0	43.1	109.1	0.4	14.8	103.1	1.4	20.3	114.1
	35–44			>74 y/o			35–44			>74 y/o		
1983–1987	2.8	38.9	122.2	1.2	83.7	106.9	1.4	14.6	123.3	1.0	55.2	95.7
1988–1992	1.0	21.9	92.4	1.0	62.2	96.4	0.6	10.0	103.3	1.6	55.7	116.3
1993–1997	1.0	23.8	97.1	0.8	46.4	89.2	0.8	10.5	123.0	1.6	41.7	121.0
1998–2002	1.4	36.5	103.7	1.6	58.2	110.4	0.4	10.5	101.2	1.4	31.8	102.5
2003–2007	2.2	50.7	126.5	2.2	57.0	126.6	0.0	10.7	84.9	0.6	16.2	72.1
2008–2012	2.0	42.9	115.4	1.4	40.4	97.4	0.2	12.7	93.7	1.0	17.8	92.6

Secondary Medical Zone ID: 034

	Male						Female					
	Suicide			Suicide			Suicide			Suicide		
	Num per year	Rate /100,000	×100	Num per year	Rate /100,000	×100	Num per year	Rate /100,000	×100	Num per year	Rate /100,000	×100
	Total (>10 y/o)			45-54			Total (>10 y/o)			45-54		
1983-1987	23.0	38.0	132.1	5.4	57.6	113.9	11.0	16.4	113.7	1.8	17.9	104.0
1988-1992	21.6	31.3	155.0	4.0	42.6	124.0	8.8	12.8	103.5	1.2	14.3	97.7
1993-1997	24.4	33.5	165.9	4.4	44.8	122.6	8.0	11.1	106.7	0.2	9.7	74.9
1998-2002	28.6	43.9	142.2	7.6	77.2	137.9	11.8	14.1	126.5	1.2	15.0	99.9
2003-2007	24.2	41.8	129.6	4.4	60.4	108.1	8.6	13.0	109.2	1.2	14.7	107.4
2008-2012	20.0	38.4	120.0	3.8	55.8	114.9	8.2	13.1	109.6	0.8	14.7	98.8
	15-24			55-64			15-24			55-64		
1983-1987	1.0	14.3	102.3	5.0	59.0	138.9	0.0	5.1	75.2	1.2	17.7	89.6
1988-1992	1.4	12.9	129.9	3.8	43.1	121.3	0.4	5.5	104.6	1.4	17.1	95.9
1993-1997	1.4	14.4	127.3	5.6	56.5	140.4	0.0	4.1	79.4	2.2	19.7	123.8
1998-2002	0.2	13.1	83.0	7.2	82.7	130.2	0.4	7.3	103.4	2.4	22.0	117.1
2003-2007	1.0	19.0	109.1	6.8	76.1	131.9	0.6	10.3	114.3	1.0	15.4	92.2
2008-2012	1.0	23.2	109.2	3.4	46.6	96.0	0.2	9.1	93.4	1.4	15.7	105.6
	25-34			65-74			25-34			65-74		
1983-1987	4.2	39.6	154.7	2.0	47.7	108.9	1.4	13.3	123.2	2.8	41.8	133.7
1988-1992	1.8	23.2	121.5	4.4	64.8	183.8	0.4	8.0	92.7	2.6	34.0	130.4
1993-1997	2.8	30.1	154.3	3.8	50.2	157.4	1.0	10.4	124.7	2.2	24.6	126.5
1998-2002	3.2	37.3	138.0	5.2	62.0	136.3	0.2	9.9	85.6	2.8	27.3	130.2
2003-2007	1.4	30.3	97.8	4.0	51.4	119.9	0.8	13.8	106.5	1.6	19.0	101.9
2008-2012	2.2	38.1	117.1	2.8	41.4	104.9	0.4	13.6	95.1	1.4	17.8	100.3
	35-44			>74 y/o			35-44			>74 y/o		
1983-1987	3.6	36.5	114.6	1.8	79.8	101.8	1.8	14.4	122.3	2.0	60.1	104.2
1988-1992	3.2	30.7	129.5	3.0	90.0	139.5	0.8	9.6	99.4	2.0	46.0	96.2
1993-1997	3.4	35.6	145.3	3.0	77.6	149.0	0.4	7.6	89.6	1.8	32.9	95.5
1998-2002	3.6	46.1	130.8	1.6	47.8	90.6	1.8	15.4	149.3	3.0	39.4	127.0
2003-2007	3.6	52.3	130.5	2.8	52.9	117.5	0.6	12.3	97.4	2.8	29.7	132.4
2008-2012	4.0	48.9	131.5	2.8	45.6	109.8	1.0	14.8	109.2	3.0	25.9	134.9

Secondary Medical Zone ID: 035

	Male						Female					
	Suicide			Suicide			Suicide			Suicide		
	Num per year	Rate /100,000	×100	Num per year	Rate /100,000	×100	Num per year	Rate /100,000	×100	Num per year	Rate /100,000	×100
	Total (>10 y/o)			45-54			Total (>10 y/o)			45-54		
1983-1987	20.2	44.3	181.0	6.0	87.2	172.4	10.6	19.2	152.9	1.8	21.4	124.3
1988-1992	19.2	35.4	205.0	3.4	50.7	147.6	10.6	16.4	163.3	2.2	20.6	140.9
1993-1997	22.0	38.4	219.0	3.0	46.2	126.3	10.0	13.9	167.9	1.0	14.4	111.8
1998-2002	19.6	43.2	150.5	4.4	70.3	125.6	10.4	15.0	151.2	1.6	18.4	122.3
2003-2007	21.0	46.9	163.3	5.4	86.3	154.3	8.8	14.1	137.9	0.4	12.6	91.6
2008-2012	14.6	37.4	126.4	2.8	55.4	114.1	6.8	13.7	118.9	1.2	17.7	118.7
	15-24			55-64			15-24			55-64		
1983-1987	1.0	16.0	114.7	4.6	73.7	173.4	0.4	7.1	104.2	1.4	22.8	115.7
1988-1992	0.6	10.5	106.0	4.6	64.0	180.2	0.0	4.4	82.5	1.8	22.5	125.9
1993-1997	0.8	12.7	112.0	6.4	80.3	199.4	0.2	5.2	98.9	2.4	24.8	156.0
1998-2002	0.6	15.9	100.9	5.4	93.2	146.7	0.2	6.8	97.3	1.4	21.0	111.8
2003-2007	0.4	16.7	95.9	3.8	70.9	122.8	0.6	10.8	120.4	1.6	20.1	119.8
2008-2012	1.0	24.5	115.3	1.4	42.0	86.5	0.0	8.2	84.8	0.8	15.1	101.3
	25-34			65-74			25-34			65-74		
1983-1987	2.2	31.8	124.4	1.4	51.6	117.9	1.4	14.8	137.3	2.4	48.4	155.0
1988-1992	1.8	26.4	138.0	2.2	52.9	150.1	0.2	7.8	89.7	2.8	47.1	180.6
1993-1997	2.2	29.5	151.3	2.0	43.6	136.7	0.6	9.4	112.7	1.6	26.2	134.6
1998-2002	2.2	34.6	128.0	2.0	47.9	105.3	1.0	13.9	120.0	2.2	29.3	139.5
2003-2007	1.8	36.5	117.7	3.6	62.0	144.5	0.2	11.8	90.6	2.4	27.5	147.5
2008-2012	1.4	35.6	109.2	2.2	45.8	115.9	0.2	13.3	92.6	2.2	24.8	139.1
	35-44			>74 y/o			35-44			>74 y/o		
1983-1987	2.2	37.2	116.8	2.6	132.8	169.5	0.8	12.6	107.1	2.4	94.7	164.1
1988-1992	3.0	36.0	151.9	3.4	132.2	205.0	0.2	8.5	87.9	3.4	95.8	200.1
1993-1997	5.2	57.1	233.0	2.4	89.9	172.6	1.0	11.0	129.6	3.2	71.6	207.9
1998-2002	2.2	41.1	116.7	2.8	86.4	164.0	0.6	11.0	106.2	3.4	61.6	198.4
2003-2007	3.6	62.0	154.8	2.4	63.1	140.2	0.6	13.0	103.3	3.0	43.1	192.2
2008-2012	1.6	38.6	103.8	4.2	75.0	180.8	0.6	14.0	103.1	1.6	22.7	118.2

Secondary Medical Zone ID: 036

	Male						Female					
	Suicide			Suicide			Suicide			Suicide		
	Num per year	Rate /100,000	×100	Num per year	Rate /100,000	×100	Num per year	Rate /100,000	×100	Num per year	Rate /100,000	×100
	Total (>10 y/o)			45–54			Total (>10 y/o)			45–54		
1983–1987	20.8	43.1	168.4	5.8	80.7	159.5	13.8	21.0	173.5	1.0	16.9	98.3
1988–1992	17.8	33.1	181.1	4.0	55.4	161.4	14.2	17.9	192.3	1.0	15.5	105.8
1993–1997	15.8	30.8	157.4	3.2	46.6	127.5	12.6	15.0	191.0	1.0	14.4	111.4
1998–2002	22.4	44.5	158.7	5.8	79.5	142.0	11.2	14.6	154.6	0.2	12.8	84.9
2003–2007	18.8	41.4	145.9	5.0	81.0	144.9	9.4	14.1	142.3	0.6	13.5	98.6
2008–2012	18.0	38.8	145.0	2.4	52.6	108.2	7.4	13.4	123.9	0.2	13.1	87.6
	15–24			55–64			15–24			55–64		
1983–1987	1.0	16.5	118.0	4.0	60.3	141.9	0.6	8.1	118.8	2.6	28.3	143.2
1988–1992	0.8	11.7	117.7	3.6	50.8	143.0	0.4	6.1	115.1	2.6	25.5	142.7
1993–1997	0.4	11.3	100.2	3.4	52.0	129.2	0.4	6.1	116.7	2.2	22.9	144.1
1998–2002	0.4	15.2	96.2	4.6	81.8	128.7	0.0	6.2	87.6	2.2	24.4	129.4
2003–2007	0.4	17.0	97.6	3.0	61.6	106.7	0.0	7.7	85.3	1.0	17.5	104.7
2008–2012	0.4	20.8	98.2	4.4	61.7	127.0	0.6	12.2	125.4	0.6	14.4	96.5
	25–34			65–74			25–34			65–74		
1983–1987	2.0	30.1	117.8	3.0	68.5	156.6	1.0	13.0	121.1	4.0	63.0	201.6
1988–1992	0.6	17.5	91.3	3.0	57.1	162.1	1.2	12.5	143.6	2.0	34.8	133.5
1993–1997	1.8	27.2	139.4	2.4	43.7	137.1	0.0	7.1	84.7	2.4	30.6	157.0
1998–2002	2.2	35.5	131.4	5.0	71.5	157.3	0.2	10.8	93.2	3.2	34.4	163.9
2003–2007	1.0	31.3	101.0	4.4	67.6	157.5	0.4	13.1	100.8	2.4	26.7	143.4
2008–2012	1.6	37.4	114.9	2.6	48.7	123.2	0.2	13.6	94.7	1.4	20.5	115.0
	35–44			>74 y/o			35–44			>74 y/o		
1983–1987	2.8	41.2	129.3	2.2	110.8	141.5	1.0	13.5	113.9	3.6	115.2	199.6
1988–1992	3.0	35.9	151.4	2.8	106.4	165.0	1.6	13.7	141.7	5.4	128.1	267.8
1993–1997	1.6	27.3	111.4	3.0	94.1	180.8	0.8	10.3	120.8	5.8	108.0	313.7
1998–2002	2.4	43.1	122.3	2.0	64.1	121.5	0.2	9.3	90.0	5.2	79.3	255.2
2003–2007	0.8	32.2	80.3	4.2	83.0	184.4	0.6	13.3	105.6	4.2	51.4	229.2
2008–2012	1.8	40.8	109.7	4.8	77.1	185.8	0.0	11.7	86.5	4.4	41.5	215.9

Secondary Medical Zone ID: 037

	Male						Female					
	Suicide			Suicide			Suicide			Suicide		
	Num per year	Rate /100,000	×100	Num per year	Rate /100,000	×100	Num per year	Rate /100,000	×100	Num per year	Rate /100,000	×100
	Total (>10 y/o)			45-54			Total (>10 y/o)			45-54		
1983-1987	26.0	32.2	98.4	5.8	49.5	97.9	17.4	16.8	115.5	3.4	21.8	126.5
1988-1992	21.0	24.2	98.6	4.0	33.9	98.8	15.0	13.6	109.6	2.0	15.5	106.3
1993-1997	20.6	23.5	91.7	5.2	37.0	101.2	12.8	10.8	106.0	1.8	13.4	103.5
1998-2002	38.2	38.5	111.6	9.8	61.6	110.0	13.6	12.2	98.3	1.6	13.4	89.1
2003-2007	39.6	41.9	114.9	10.2	69.5	124.3	13.8	12.6	103.3	2.6	16.1	117.5
2008-2012	37.2	38.5	116.4	8.2	62.8	129.2	14.4	13.9	108.4	1.8	15.4	103.3
	15-24			55-64			15-24			55-64		
1983-1987	2.0	15.6	111.8	4.0	37.7	88.7	0.6	6.3	92.2	2.4	19.0	96.1
1988-1992	0.6	7.9	79.4	4.0	34.1	96.1	0.4	4.6	86.7	1.8	15.6	87.6
1993-1997	2.0	12.6	111.1	3.6	34.8	86.5	1.0	6.2	118.8	3.0	19.9	125.4
1998-2002	1.8	14.7	93.3	5.8	54.3	85.4	1.0	7.5	106.1	1.8	17.4	92.4
2003-2007	2.4	18.7	107.1	6.2	49.2	85.3	0.8	8.3	92.7	1.8	15.6	93.4
2008-2012	2.8	24.5	115.4	8.4	52.8	108.7	1.4	12.2	125.5	1.6	13.3	89.2
	25-34			65-74			25-34			65-74		
1983-1987	3.4	24.7	96.8	1.8	33.9	77.5	1.2	9.9	92.3	3.0	33.5	107.3
1988-1992	1.8	16.8	88.0	2.4	31.9	90.5	2.0	12.1	139.9	3.6	31.5	120.9
1993-1997	1.8	18.2	93.1	3.4	33.5	104.9	0.2	6.2	74.0	2.6	20.6	105.5
1998-2002	6.0	40.0	148.3	5.2	47.6	104.6	1.4	12.2	105.4	2.8	21.5	102.3
2003-2007	4.6	35.8	115.6	4.8	44.8	104.5	0.8	11.0	84.3	2.4	19.2	103.3
2008-2012	2.8	29.9	91.7	4.8	43.0	108.9	2.4	18.1	126.1	1.6	16.4	92.2
	35-44			>74 y/o			35-44			>74 y/o		
1983-1987	6.4	40.6	127.5	2.4	67.4	86.0	2.2	14.1	119.1	4.4	72.9	126.3
1988-1992	4.4	27.0	113.8	3.6	73.0	113.2	1.8	10.9	112.6	3.4	46.9	97.9
1993-1997	2.0	17.7	72.2	2.6	48.8	93.6	0.2	5.4	64.1	4.0	41.7	121.1
1998-2002	4.8	38.6	109.5	4.4	60.2	114.1	0.8	9.0	86.9	4.2	34.9	112.3
2003-2007	8.0	64.9	161.9	3.4	40.5	89.9	1.2	12.4	97.9	4.2	27.3	121.6
2008-2012	6.0	46.6	125.4	4.2	41.3	99.5	1.6	14.4	105.9	4.0	22.5	117.0

Secondary Medical Zone ID: 038

	Male						Female					
	Suicide			Suicide			Suicide			Suicide		
	Num per year	Rate /100,000	×100	Num per year	Rate /100,000	×100	Num per year	Rate /100,000	×100	Num per year	Rate /100,000	×100
	Total (>10 y/o)			45–54			Total (>10 y/o)			45–54		
1983–1987	133.8	29.5	90.5	33.0	45.9	90.7	66.6	13.9	89.8	10.8	14.8	85.6
1988–1992	110.4	21.6	87.4	24.2	29.9	87.0	59.0	10.6	81.7	11.4	13.9	94.8
1993–1997	131.2	22.4	89.1	31.4	32.5	88.7	63.6	9.8	90.2	13.6	13.7	106.3
1998–2002	212.2	33.1	91.8	55.0	52.8	94.4	82.8	11.6	91.4	12.8	12.6	83.8
2003–2007	248.0	37.4	101.4	53.2	55.1	98.5	90.4	12.3	96.4	15.2	15.1	110.2
2008–2012	221.6	33.4	94.9	43.2	47.8	98.3	84.6	11.9	87.5	12.4	13.5	90.8
	15–24			55–64			15–24			55–64		
1983–1987	16.4	16.4	117.9	16.0	32.5	76.4	8.0	8.4	122.4	8.6	15.6	79.1
1988–1992	11.8	10.1	102.0	16.4	26.9	75.7	3.8	3.8	72.7	12.0	17.6	98.5
1993–1997	16.6	13.0	114.6	26.0	37.1	92.1	7.2	6.0	115.9	10.2	13.8	86.8
1998–2002	20.8	17.7	111.9	43.8	55.7	87.6	6.8	6.4	90.5	13.4	16.6	88.0
2003–2007	20.4	19.9	114.0	50.6	54.9	95.1	11.0	11.1	123.3	15.4	16.1	96.3
2008–2012	18.2	21.0	99.0	45.0	45.4	93.5	8.6	10.1	104.3	12.4	12.3	82.6
	25–34			65–74			25–34			65–74		
1983–1987	20.8	23.0	90.1	10.0	38.8	88.7	9.2	9.9	92.2	9.0	26.7	85.5
1988–1992	17.0	19.1	99.8	9.0	27.6	78.3	5.0	6.0	69.3	7.2	17.2	66.1
1993–1997	19.0	19.4	99.5	8.4	19.8	62.0	5.8	6.3	76.0	8.0	14.9	76.4
1998–2002	30.0	27.3	101.0	22.4	40.5	89.0	14.0	12.6	108.9	14.4	21.7	103.5
2003–2007	41.8	37.4	120.7	27.2	43.1	100.4	12.6	11.6	89.0	10.6	15.1	81.2
2008–2012	35.0	35.5	108.8	25.2	36.3	91.8	16.0	15.5	107.9	10.4	14.0	78.4
	35–44			>74 y/o			35–44			>74 y/o		
1983–1987	29.8	31.0	97.5	7.8	63.6	81.2	9.0	9.7	81.8	11.8	60.6	105.1
1988–1992	22.8	22.4	94.3	8.8	53.0	82.2	7.8	7.9	81.6	11.6	42.1	88.0
1993–1997	20.6	21.2	86.7	9.2	43.3	83.2	6.2	6.6	77.6	12.4	33.9	98.4
1998–2002	29.0	30.9	87.8	11.0	39.1	74.2	9.2	9.8	94.4	12.2	24.9	80.2
2003–2007	42.4	43.0	107.2	12.0	31.0	68.9	10.8	11.2	88.7	14.2	21.5	95.9
2008–2012	41.8	38.2	102.7	13.0	26.7	64.3	13.0	12.2	89.8	11.6	14.3	74.5

Secondary Medical Zone ID: 039

	Male						Female					
	Suicide			Suicide			Suicide			Suicide		
	Num per year	Rate /100,000	×100	Num per year	Rate /100,000	×100	Num per year	Rate /100,000	×100	Num per year	Rate /100,000	×100
	Total (>10 y/o)			45-54			Total (>10 y/o)			45-54		
1983-1987	47.4	34.5	108.9	11.6	54.6	107.9	26.0	15.9	105.0	4.0	16.9	98.2
1988-1992	35.0	25.2	101.9	7.0	36.7	106.9	21.8	12.5	97.9	2.2	12.8	87.6
1993-1997	42.4	27.8	115.2	9.8	43.3	118.3	21.6	11.4	108.7	1.6	10.4	80.3
1998-2002	62.2	40.4	115.9	17.4	67.9	121.3	23.6	12.6	103.1	4.4	17.3	115.1
2003-2007	73.4	47.4	136.1	20.6	85.1	152.1	21.6	11.8	101.5	2.0	11.2	81.9
2008-2012	64.4	41.8	130.3	11.0	56.0	115.3	20.0	13.4	99.4	3.2	16.4	109.9
	15-24			55-64			15-24			55-64		
1983-1987	2.6	14.9	106.8	7.8	40.0	94.1	2.8	11.3	165.2	4.0	18.3	92.7
1988-1992	2.6	12.3	124.4	6.0	29.7	83.6	0.4	4.0	76.0	3.4	15.2	85.2
1993-1997	2.2	11.6	102.6	8.6	42.8	106.3	0.6	4.4	84.1	4.6	18.5	116.6
1998-2002	4.6	20.9	132.4	13.6	73.1	115.1	1.2	7.0	99.6	4.0	19.6	104.1
2003-2007	3.8	20.9	119.8	15.2	71.2	123.4	1.6	9.9	109.9	2.8	15.4	91.9
2008-2012	3.0	22.7	107.1	15.4	59.7	122.9	2.6	15.4	158.7	2.4	12.6	84.5
	25-34			65-74			25-34			65-74		
1983-1987	7.4	30.2	118.0	7.2	59.8	136.5	1.6	8.6	80.0	3.8	27.0	86.5
1988-1992	3.6	19.1	100.0	5.2	38.1	108.1	1.6	8.7	100.7	3.4	21.1	81.1
1993-1997	3.8	21.9	111.9	6.6	37.7	118.0	1.8	9.8	117.3	4.6	21.3	109.1
1998-2002	5.6	31.3	116.0	8.6	46.0	101.2	1.6	11.1	95.8	4.0	18.3	87.0
2003-2007	9.0	45.0	145.0	7.2	41.8	97.4	1.0	9.8	75.6	5.4	22.6	121.4
2008-2012	10.2	49.8	152.9	7.6	45.9	116.2	1.2	11.5	80.2	2.0	14.1	79.3
	35-44			>74 y/o			35-44			>74 y/o		
1983-1987	8.4	37.3	117.2	2.2	49.5	63.2	2.6	12.4	105.3	7.2	74.8	129.7
1988-1992	6.2	24.7	104.1	4.2	61.1	94.7	3.2	11.9	122.7	7.6	60.4	126.2
1993-1997	7.0	28.7	116.9	4.0	48.3	92.8	2.2	9.3	109.3	6.0	39.3	114.3
1998-2002	7.8	39.0	110.7	4.6	45.2	85.8	1.4	8.9	85.7	7.0	36.2	116.7
2003-2007	10.2	57.1	142.4	7.0	49.1	109.0	1.2	10.3	81.9	7.6	30.1	134.1
2008-2012	8.2	45.7	122.8	8.8	50.5	121.8	2.4	14.6	107.9	6.2	21.2	110.2

Secondary Medical Zone ID: 040

	Male						Female					
	Suicide			Suicide			Suicide			Suicide		
	Num per year	Rate /100,000	×100	Num per year	Rate /100,000	×100	Num per year	Rate /100,000	×100	Num per year	Rate /100,000	×100
	Total (>10 y/o)			45–54			Total (>10 y/o)			45–54		
1983–1987	62.8	33.7	103.6	14.6	47.7	94.4	33.0	15.1	99.0	5.0	15.8	91.9
1988–1992	52.2	26.6	108.5	10.4	35.0	102.0	34.2	13.5	110.9	5.0	15.7	107.4
1993–1997	52.2	25.8	102.8	11.8	37.1	101.5	29.8	11.3	109.8	4.6	14.2	109.6
1998–2002	84.8	40.3	114.4	20.6	61.6	110.1	35.4	13.0	111.7	4.8	15.1	100.5
2003–2007	97.4	48.1	134.0	25.8	84.3	150.8	30.4	12.7	104.7	4.6	15.1	110.0
2008–2012	66.8	35.5	103.6	14.2	55.2	113.6	26.2	13.0	96.5	3.6	14.7	98.3
	15–24			55–64			15–24			55–64		
1983–1987	5.4	18.7	134.3	11.0	43.0	101.3	1.6	6.4	93.3	6.0	20.1	101.6
1988–1992	3.6	12.1	121.8	9.4	33.2	93.4	2.6	7.9	150.7	6.2	19.0	106.3
1993–1997	2.2	9.6	84.9	11.6	40.5	100.7	2.0	6.8	129.5	4.8	15.5	97.3
1998–2002	3.6	15.3	96.7	16.0	58.9	92.7	1.2	6.1	87.4	4.0	15.5	82.3
2003–2007	4.0	18.7	107.2	20.2	66.9	115.9	1.8	9.3	102.9	3.8	14.3	85.6
2008–2012	3.0	19.3	90.9	16.0	50.4	103.7	2.2	12.1	124.6	4.0	13.7	91.9
	25–34			65–74			25–34			65–74		
1983–1987	8.0	25.5	99.7	7.4	50.7	115.7	3.6	11.3	105.3	6.2	31.6	101.0
1988–1992	7.8	26.8	140.3	5.0	30.8	87.4	1.2	6.2	71.2	6.4	27.4	105.0
1993–1997	7.2	26.7	136.9	6.8	31.6	99.1	1.8	7.9	94.7	4.8	18.0	92.2
1998–2002	9.2	35.0	129.7	13.0	51.3	112.7	2.6	11.5	99.1	5.8	19.8	94.4
2003–2007	11.8	45.8	147.6	12.0	47.5	110.8	1.8	10.4	80.3	7.2	22.8	122.5
2008–2012	7.4	34.9	107.0	8.2	35.9	90.9	2.8	14.5	101.3	4.2	16.2	91.2
	35–44			>74 y/o			35–44			>74 y/o		
1983–1987	10.4	31.7	99.6	5.6	76.7	98.0	4.0	12.3	104.2	6.6	56.5	98.0
1988–1992	11.4	31.5	132.8	4.4	52.2	80.9	2.8	9.0	93.2	10.0	62.6	130.9
1993–1997	7.4	24.1	98.5	5.0	47.6	91.4	1.8	6.9	81.6	9.8	48.4	140.6
1998–2002	14.2	50.2	142.5	8.2	58.9	111.7	3.2	11.5	111.7	13.6	52.0	167.6
2003–2007	16.0	63.6	158.8	7.6	43.6	96.8	3.2	13.3	105.6	8.0	25.4	113.3
2008–2012	8.8	38.5	103.4	9.0	42.5	102.3	2.6	12.7	93.9	6.8	18.8	97.6

Secondary Medical Zone ID: 041

	Male						Female					
	Suicide			Suicide			Suicide			Suicide		
	Num per year	Rate /100,000	×100	Num per year	Rate /100,000	×100	Num per year	Rate /100,000	×100	Num per year	Rate /100,000	×100
	Total (>10 y/o)			45-54			Total (>10 y/o)			45-54		
1983-1987	30.0	40.4	141.4	10.8	85.1	168.3	14.0	16.5	113.8	2.8	19.7	114.2
1988-1992	23.6	30.2	142.2	3.8	38.4	111.7	14.8	14.6	127.5	2.6	17.8	121.8
1993-1997	22.6	29.2	131.9	6.4	52.0	142.0	11.2	11.5	113.6	2.6	17.1	132.1
1998-2002	39.8	47.7	159.6	9.2	78.3	140.0	12.8	13.2	113.1	1.8	15.9	105.9
2003-2007	36.4	50.0	154.7	7.6	77.0	137.6	18.4	16.2	156.1	2.8	19.5	142.2
2008-2012	23.2	36.9	113.7	3.4	46.7	96.1	11.2	12.8	112.9	0.6	12.7	84.9
	15-24			55-64			15-24			55-64		
1983-1987	0.6	11.6	83.5	6.0	56.7	133.4	0.6	7.1	103.8	2.6	21.8	110.4
1988-1992	0.6	9.7	98.0	5.6	47.6	134.1	0.8	6.8	129.2	3.4	22.9	128.4
1993-1997	0.4	9.9	87.6	4.6	44.4	110.3	0.2	4.7	90.4	2.4	18.1	113.6
1998-2002	1.4	17.8	112.8	9.0	88.1	138.6	0.2	6.2	88.9	3.2	22.9	121.9
2003-2007	1.0	18.1	104.0	7.4	72.3	125.2	0.6	9.9	109.6	3.6	23.0	137.4
2008-2012	1.6	25.4	119.7	6.2	56.4	116.1	0.2	8.6	89.0	1.6	15.2	101.9
	25-34			65-74			25-34			65-74		
1983-1987	2.8	27.9	109.3	2.6	46.7	106.7	0.8	9.8	91.5	1.8	27.8	88.9
1988-1992	3.2	30.3	158.5	4.0	50.9	144.6	0.4	7.6	87.5	2.8	30.0	115.1
1993-1997	2.0	23.6	120.7	4.4	46.9	147.1	0.2	6.9	83.3	1.8	18.5	94.9
1998-2002	3.6	37.1	137.4	6.6	62.4	137.1	0.8	11.8	102.0	3.4	26.3	125.1
2003-2007	4.4	46.3	149.3	5.4	55.1	128.4	1.2	14.8	114.0	4.0	27.1	145.4
2008-2012	2.8	38.0	116.8	3.0	39.7	100.5	0.6	13.7	95.5	3.2	23.2	130.4
	35-44			>74 y/o			35-44			>74 y/o		
1983-1987	5.0	41.3	129.8	2.0	81.8	104.5	1.4	12.1	102.6	4.0	86.2	149.4
1988-1992	3.2	27.7	116.9	3.0	82.1	127.3	0.6	8.4	86.8	4.2	67.2	140.5
1993-1997	3.2	30.5	124.4	1.6	45.1	86.6	0.8	8.6	101.0	3.2	41.6	121.0
1998-2002	5.6	55.0	156.2	4.4	76.4	144.9	0.6	9.5	91.8	2.8	30.7	98.9
2003-2007	8.0	83.7	209.0	2.6	43.6	96.8	2.0	17.1	135.5	4.2	33.1	147.6
2008-2012	2.6	38.1	102.4	3.6	45.9	110.7	0.4	11.9	87.5	4.6	29.0	151.0

Secondary Medical Zone ID: 042

	Male						Female					
	Suicide			Suicide			Suicide			Suicide		
	Num per year	Rate /100,000	×100	Num per year	Rate /100,000	×100	Num per year	Rate /100,000	×100	Num per year	Rate /100,000	×100
	Total (>10 y/o)			45-54			Total (>10 y/o)			45-54		
1983-1987	14.8	40.3	162.2	2.8	59.9	118.4	8.4	18.3	144.2	1.2	19.4	112.6
1988-1992	10.6	29.6	148.5	3.2	54.3	158.0	6.6	14.4	131.2	1.2	17.3	118.5
1993-1997	11.0	29.6	148.3	2.0	42.3	115.7	6.6	12.6	142.6	1.8	18.8	145.5
1998-2002	14.2	41.4	143.2	3.6	72.4	129.3	5.8	13.4	121.6	0.2	13.7	90.7
2003-2007	12.6	42.0	139.2	3.6	81.4	145.5	4.6	13.1	113.2	0.4	13.6	99.5
2008-2012	8.4	35.8	112.5	1.6	52.3	107.6	5.6	13.7	123.6	0.6	16.2	108.3
	15-24			55-64			15-24			55-64		
1983-1987	0.6	15.9	114.1	3.8	65.7	154.6	0.6	8.7	127.5	1.4	23.3	118.1
1988-1992	0.6	11.5	116.6	2.4	45.2	127.4	0.4	6.5	123.8	0.8	17.7	99.3
1993-1997	0.2	11.1	98.3	2.8	52.6	130.8	0.0	4.8	91.9	1.2	19.0	119.4
1998-2002	0.2	15.3	96.7	3.8	83.9	132.1	0.2	7.4	105.0	1.2	21.2	112.6
2003-2007	0.4	18.2	104.3	2.8	67.8	117.4	0.0	8.3	91.8	0.6	16.8	100.4
2008-2012	0.6	23.6	111.4	2.8	57.7	118.8	0.2	10.4	106.8	0.4	14.5	96.9
	25-34			65-74			25-34			65-74		
1983-1987	1.2	29.1	113.7	1.8	56.4	128.9	0.4	11.1	103.3	2.2	47.0	150.3
1988-1992	0.8	21.9	114.7	0.8	34.0	96.5	0.4	9.5	110.0	1.2	29.8	114.2
1993-1997	1.4	27.2	139.1	1.6	39.3	123.3	0.2	8.4	101.4	1.2	23.4	119.9
1998-2002	0.4	26.0	96.1	3.0	60.2	132.3	0.0	10.6	91.9	1.2	23.2	110.6
2003-2007	1.4	38.3	123.4	1.8	47.9	111.5	0.4	14.0	107.7	1.0	20.1	108.1
2008-2012	0.4	31.8	97.6	1.0	38.7	97.9	0.2	14.4	100.3	1.2	20.8	116.9
	35-44			>74 y/o			35-44			>74 y/o		
1983-1987	2.8	47.0	147.6	1.8	111.6	142.4	0.8	13.5	113.9	1.8	80.2	139.1
1988-1992	1.4	28.3	119.2	1.4	77.4	120.0	1.2	13.0	134.7	1.4	53.1	111.0
1993-1997	1.6	32.3	131.8	1.4	64.6	124.1	0.2	8.2	96.4	2.0	52.6	152.7
1998-2002	1.4	40.3	114.4	1.8	67.1	127.2	0.4	11.0	106.7	2.6	53.0	170.7
2003-2007	1.0	41.3	103.0	1.6	52.0	115.5	0.4	13.3	105.2	1.8	31.6	141.0
2008-2012	0.2	33.5	89.9	1.8	48.9	117.9	0.2	13.4	98.9	2.8	33.9	176.4

Secondary Medical Zone ID: 043

	Male						Female					
	Suicide			Suicide			Suicide			Suicide		
	Num per year	Rate /100,000	×100	Num per year	Rate /100,000	×100	Num per year	Rate /100,000	×100	Num per year	Rate /100,000	×100
	Total (>10 y/o)			45-54			Total (>10 y/o)			45-54		
1983-1987	30.0	45.1	172.3	9.0	88.7	175.3	11.4	16.4	114.6	3.0	23.0	133.4
1988-1992	25.8	35.2	185.4	6.6	63.6	185.2	12.0	14.4	127.9	0.8	13.1	89.4
1993-1997	26.6	35.0	182.5	4.8	48.1	131.5	14.6	14.0	164.7	2.8	19.2	148.7
1998-2002	38.8	51.5	189.6	9.4	90.0	160.8	15.8	15.0	155.2	1.4	15.7	104.4
2003-2007	37.2	53.7	194.3	9.8	108.9	194.8	14.8	15.5	154.4	1.8	17.0	124.0
2008-2012	24.6	40.8	144.2	4.2	59.8	123.1	12.0	13.8	134.4	1.6	18.3	122.4
	15-24			55-64			15-24			55-64		
1983-1987	0.6	12.8	91.8	6.2	67.4	158.5	0.4	6.8	99.6	1.8	20.2	102.5
1988-1992	0.6	10.2	103.3	7.0	65.9	185.5	0.6	6.5	122.5	2.4	21.0	117.9
1993-1997	1.2	13.8	121.6	7.0	67.6	167.9	0.2	5.0	95.4	2.8	22.3	140.4
1998-2002	0.8	16.2	102.4	9.8	108.1	170.1	0.0	5.9	83.4	2.6	23.1	122.5
2003-2007	1.2	20.4	116.8	9.6	99.9	173.0	0.6	10.6	117.3	2.8	22.0	131.4
2008-2012	1.0	23.7	111.9	5.0	56.0	115.4	0.0	8.1	83.1	1.8	17.0	113.8
	25-34			65-74			25-34			65-74		
1983-1987	3.4	35.4	138.5	3.8	64.7	147.8	1.4	13.5	125.1	1.6	29.0	92.8
1988-1992	2.2	27.1	141.9	4.0	57.3	162.5	0.2	7.4	84.8	3.0	35.7	137.0
1993-1997	1.2	21.0	107.7	4.6	56.1	176.0	0.4	8.2	98.8	2.8	27.7	142.2
1998-2002	2.4	33.7	124.8	5.6	64.3	141.5	0.6	11.8	101.6	4.4	35.1	167.2
2003-2007	1.8	33.7	108.8	6.4	71.7	167.2	1.2	15.9	122.5	3.2	26.6	142.7
2008-2012	1.6	34.7	106.5	6.2	66.9	169.5	0.0	11.9	83.1	3.0	24.9	139.7
	35-44			>74 y/o			35-44			>74 y/o		
1983-1987	3.8	39.4	123.7	3.2	120.1	153.3	0.8	11.0	93.1	2.4	65.7	113.9
1988-1992	1.8	22.8	96.1	3.6	103.2	160.0	0.4	8.3	86.1	4.6	83.9	175.3
1993-1997	4.2	41.1	167.6	3.6	86.4	165.9	0.6	8.4	99.3	5.0	70.7	205.4
1998-2002	5.0	58.1	164.9	5.6	104.6	198.5	0.4	9.4	90.6	6.4	71.2	229.4
2003-2007	4.0	58.6	146.4	4.2	68.5	152.1	0.6	12.4	98.1	4.4	40.3	179.4
2008-2012	3.0	44.5	119.6	3.4	50.7	122.3	0.4	12.6	92.8	5.2	36.6	190.4

Secondary Medical Zone ID: 044

	Male						Female					
	Suicide			Suicide			Suicide			Suicide		
	Num per year	Rate /100,000	×100	Num per year	Rate /100,000	×100	Num per year	Rate /100,000	×100	Num per year	Rate /100,000	×100
	Total (>10 y/o)			45–54			Total (>10 y/o)			45–54		
1983–1987	75.4	41.2	135.4	21.6	73.7	145.7	35.6	17.2	116.8	5.6	17.7	103.0
1988–1992	59.4	30.4	130.2	13.8	46.4	135.0	35.6	14.6	122.0	5.0	15.6	106.9
1993–1997	63.8	30.3	127.6	16.6	48.7	133.1	29.8	11.5	112.0	5.4	14.9	115.6
1998–2002	95.6	43.7	127.6	27.0	75.6	135.1	39.6	14.3	121.1	6.8	17.8	118.2
2003–2007	108.2	49.5	143.2	28.2	87.9	157.1	32.4	12.7	104.8	3.0	11.0	79.9
2008–2012	87.0	41.2	127.3	16.6	60.2	123.8	35.6	14.0	115.6	3.2	12.6	84.8
	15–24			55–64			15–24			55–64		
1983–1987	6.0	20.3	145.4	13.4	57.1	134.4	1.6	6.2	90.2	6.2	22.5	114.0
1988–1992	2.6	9.3	94.0	11.4	42.6	120.1	0.6	3.4	65.3	6.6	20.9	116.8
1993–1997	4.0	12.4	109.8	12.6	46.2	114.8	0.6	3.4	65.3	5.6	17.7	111.3
1998–2002	4.0	14.8	93.8	21.2	76.5	120.3	1.0	5.2	74.4	7.0	21.6	114.9
2003–2007	4.2	17.8	101.9	26.4	82.5	142.9	1.8	8.4	93.2	6.6	18.8	112.3
2008–2012	3.8	20.6	97.1	20.2	58.6	120.6	2.4	11.5	118.3	5.6	15.6	104.6
	25–34			65–74			25–34			65–74		
1983–1987	10.4	32.3	126.3	5.4	44.5	101.7	3.0	9.8	90.9	7.6	43.7	139.7
1988–1992	9.0	30.8	161.1	8.4	51.2	145.4	2.0	7.7	88.6	7.8	36.2	138.7
1993–1997	6.6	24.4	124.8	7.6	38.3	120.0	2.6	9.1	109.6	4.4	18.1	92.9
1998–2002	10.0	34.4	127.5	12.2	52.8	116.0	5.0	15.5	133.7	6.8	23.3	110.9
2003–2007	10.2	37.0	119.2	12.4	51.3	119.6	4.0	14.3	109.9	4.8	17.1	92.0
2008–2012	9.4	39.4	121.0	14.0	54.9	138.8	3.8	15.9	111.0	6.6	21.4	120.4
	35–44			>74 y/o			35–44			>74 y/o		
1983–1987	12.8	37.9	119.0	5.8	101.6	129.7	4.8	13.2	112.0	6.8	76.4	132.5
1988–1992	8.8	25.2	106.5	5.2	68.0	105.5	3.8	10.3	106.7	9.6	72.2	150.8
1993–1997	9.6	29.6	120.7	6.8	67.9	130.5	2.2	7.4	86.9	8.8	50.5	146.8
1998–2002	13.8	47.1	133.6	7.2	58.1	110.2	3.2	10.7	103.9	9.8	42.6	137.3
2003–2007	17.2	62.5	155.9	9.4	56.0	124.3	3.6	13.0	103.0	8.6	29.0	129.3
2008–2012	13.6	48.0	129.0	9.4	46.7	112.7	3.6	13.6	100.1	10.0	27.2	141.3

Secondary Medical Zone ID: 045

	Male						Female					
	Suicide			Suicide			Suicide			Suicide		
	Num per year	Rate /100,000	×100	Num per year	Rate /100,000	×100	Num per year	Rate /100,000	×100	Num per year	Rate /100,000	×100
	Total (>10 y/o)			45–54			Total (>10 y/o)			45–54		
1983–1987	33.8	47.5	178.6	10.6	96.8	191.5	18.8	20.9	161.6	2.4	20.1	116.7
1988–1992	26.4	33.9	172.8	5.4	52.7	153.3	20.4	18.3	185.7	2.0	17.1	117.0
1993–1997	28.8	34.1	175.6	5.0	46.2	126.4	20.4	15.9	203.0	1.4	13.8	106.7
1998–2002	41.4	49.7	175.7	13.2	103.2	184.4	20.8	16.6	178.0	2.2	17.7	117.5
2003–2007	38.8	49.5	171.3	8.2	81.6	145.9	20.4	15.3	180.7	1.4	14.4	105.4
2008–2012	27.4	38.6	133.5	6.2	67.9	139.6	17.6	15.3	159.5	1.2	15.4	103.1
	15–24			55–64			15–24			55–64		
1983–1987	1.6	18.1	129.7	5.0	55.1	129.7	0.0	5.2	76.0	3.4	27.2	137.7
1988–1992	0.6	9.7	98.2	5.4	51.8	145.8	0.4	5.5	104.2	4.2	28.1	157.2
1993–1997	0.6	10.6	94.0	8.4	73.6	182.8	0.8	6.9	132.0	4.0	27.2	171.1
1998–2002	1.0	15.5	98.0	7.6	84.9	133.6	0.4	7.0	99.4	2.4	21.8	115.8
2003–2007	1.6	20.0	114.7	9.6	91.2	158.0	0.4	8.7	96.9	2.6	20.7	123.8
2008–2012	0.6	18.5	87.4	6.6	58.9	121.2	0.6	10.8	111.6	1.8	16.2	108.3
	25–34			65–74			25–34			65–74		
1983–1987	3.2	30.6	119.8	5.0	80.0	182.7	1.0	10.9	101.0	4.2	51.6	165.2
1988–1992	3.0	29.1	152.2	3.6	52.3	148.4	0.6	8.4	97.3	5.2	52.4	200.9
1993–1997	2.4	26.1	133.8	4.8	54.7	171.5	1.0	10.1	120.9	4.0	34.0	174.2
1998–2002	1.2	24.0	88.8	7.8	77.1	169.4	0.4	10.4	90.1	5.2	38.1	181.1
2003–2007	3.8	42.3	136.4	5.8	63.8	148.7	0.6	12.3	95.0	2.4	22.0	118.1
2008–2012	1.4	29.5	90.7	4.2	50.7	128.4	1.0	15.5	108.1	4.2	29.4	165.1
	35–44			>74 y/o			35–44			>74 y/o		
1983–1987	5.2	45.7	143.6	3.0	113.7	145.2	1.8	14.5	123.0	6.0	134.1	232.4
1988–1992	3.2	28.1	118.5	5.2	134.5	208.5	1.2	10.5	108.8	6.8	114.2	238.8
1993–1997	2.8	28.1	114.5	4.8	105.8	203.2	0.6	7.9	92.7	8.6	110.9	322.1
1998–2002	5.4	54.6	155.0	5.2	93.5	177.4	1.4	12.9	124.3	8.8	88.9	286.3
2003–2007	4.6	57.4	143.3	5.2	74.6	165.6	0.6	11.8	93.1	12.4	94.8	422.4
2008–2012	2.4	37.7	101.4	5.8	69.0	166.4	0.8	13.5	99.8	8.0	50.3	261.6

Secondary Medical Zone ID: 046

	Male						Female					
	Suicide			Suicide			Suicide			Suicide		
	Num per year	Rate /100,000	×100	Num per year	Rate /100,000	×100	Num per year	Rate /100,000	×100	Num per year	Rate /100,000	×100
	Total (>10 y/o)			45-54			Total (>10 y/o)			45-54		
1983-1987	38.4	43.1	151.5	9.2	68.1	134.7	30.8	24.2	194.2	4.8	25.8	149.7
1988-1992	31.6	32.6	157.6	6.0	48.3	140.5	26.2	18.5	183.3	2.4	16.7	114.5
1993-1997	28.2	29.6	138.3	4.6	39.2	107.2	22.4	14.5	178.3	2.0	14.6	112.8
1998-2002	44.4	45.9	152.0	10.4	75.3	134.5	25.2	16.4	172.3	3.4	20.0	133.0
2003-2007	50.0	53.9	177.7	12.8	100.3	179.3	17.2	13.7	134.4	1.8	14.6	106.5
2008-2012	34.8	39.8	138.5	6.6	62.8	129.3	16.6	14.6	132.8	1.4	14.9	100.1
	15-24			55-64			15-24			55-64		
1983-1987	1.6	16.5	118.0	6.0	50.3	118.2	0.4	6.2	91.5	3.0	21.3	108.0
1988-1992	0.6	9.3	94.2	8.8	60.0	169.0	0.4	5.2	99.2	5.4	28.1	157.6
1993-1997	1.8	14.5	128.0	6.8	52.7	130.8	0.2	4.5	87.1	3.6	21.7	136.2
1998-2002	1.2	15.8	100.1	12.0	101.3	159.5	0.4	6.7	95.1	5.6	30.8	163.7
2003-2007	1.4	18.9	108.1	9.8	81.6	141.4	0.0	6.4	71.4	2.8	19.7	117.7
2008-2012	0.8	19.7	92.9	8.4	61.5	126.5	0.2	8.3	85.4	0.8	11.9	80.0
	25-34			65-74			25-34			65-74		
1983-1987	3.8	29.8	116.6	6.2	75.6	172.8	2.8	16.5	153.0	8.0	70.1	224.4
1988-1992	1.6	18.1	94.6	3.2	39.5	112.0	1.0	9.3	107.2	8.0	61.1	234.4
1993-1997	2.2	23.3	119.1	6.2	54.0	169.1	0.4	7.4	88.9	5.6	36.2	185.9
1998-2002	3.6	35.4	131.0	7.2	59.7	131.2	0.4	10.0	85.9	6.8	38.7	184.1
2003-2007	3.8	39.9	128.6	6.6	57.8	134.8	1.0	13.4	103.1	2.8	20.5	110.0
2008-2012	2.2	32.5	99.8	6.2	56.8	143.8	2.0	18.9	132.1	3.2	22.7	127.4
	35-44			>74 y/o			35-44			>74 y/o		
1983-1987	5.6	41.7	130.9	6.0	145.2	185.4	3.2	17.8	150.3	8.6	144.2	250.0
1988-1992	5.8	36.9	155.6	5.4	108.4	168.1	1.2	9.6	99.9	7.8	101.0	211.1
1993-1997	2.6	23.5	95.7	4.0	72.6	139.4	0.4	6.5	76.6	10.2	102.1	296.6
1998-2002	4.2	40.5	115.1	5.8	81.4	154.4	0.6	8.9	85.7	8.0	65.2	209.9
2003-2007	7.6	74.2	185.3	7.8	82.4	182.9	1.0	12.6	100.0	7.8	48.9	218.1
2008-2012	3.6	42.1	113.2	7.0	63.9	154.1	2.0	17.3	127.5	7.0	35.9	186.5

Secondary Medical Zone ID: 047

	Male						Female					
	Suicide			Suicide			Suicide			Suicide		
	Num per year	Rate /100,000	×100	Num per year	Rate /100,000	×100	Num per year	Rate /100,000	×100	Num per year	Rate /100,000	×100
	Total (>10 y/o)			45-54			Total (>10 y/o)			45-54		
1983-1987	20.2	35.4	117.6	3.4	45.4	89.8	15.0	18.7	138.3	2.6	21.6	125.3
1988-1992	17.4	27.2	126.4	1.6	28.1	81.8	14.2	15.7	143.3	0.8	13.3	91.2
1993-1997	21.6	30.9	146.6	6.4	57.6	157.4	13.2	13.2	149.3	1.4	14.3	111.0
1998-2002	28.2	42.7	137.3	8.2	79.1	141.4	13.0	14.1	131.5	2.0	17.6	117.1
2003-2007	28.6	44.8	145.0	6.2	71.2	127.3	14.0	14.7	145.0	1.4	14.9	108.7
2008-2012	25.2	40.6	137.4	4.6	59.6	122.7	12.6	14.5	134.9	2.0	19.5	130.4
	15-24			55-64			15-24			55-64		
1983-1987	1.2	16.7	120.1	3.6	45.3	106.5	0.2	6.1	90.0	1.8	19.5	98.9
1988-1992	0.2	8.6	87.3	5.4	52.9	148.9	0.4	5.7	108.7	1.4	16.5	92.4
1993-1997	0.8	12.0	106.1	3.4	42.8	106.4	0.8	7.2	137.6	1.4	15.9	100.0
1998-2002	1.0	16.7	105.6	5.6	74.0	116.5	0.6	8.0	114.6	2.0	21.1	112.1
2003-2007	1.4	20.9	119.7	6.6	76.4	132.4	1.0	12.4	137.8	1.2	16.4	98.0
2008-2012	1.2	24.3	114.7	6.4	62.4	128.5	0.4	10.2	105.6	2.2	18.0	121.0
	25-34			65-74			25-34			65-74		
1983-1987	1.6	22.6	88.3	3.6	61.4	140.3	0.8	10.4	96.4	3.4	44.8	143.4
1988-1992	0.6	14.8	77.2	3.8	53.1	150.8	0.6	8.8	101.2	3.6	39.7	152.1
1993-1997	0.8	17.9	91.8	2.4	34.5	108.2	0.2	7.3	88.0	4.8	39.4	202.3
1998-2002	2.2	31.8	117.6	4.2	52.4	115.1	0.2	10.0	86.6	3.4	29.2	139.0
2003-2007	2.6	37.8	121.9	4.6	56.7	132.0	1.2	15.6	120.1	3.0	25.8	138.5
2008-2012	3.0	40.8	125.3	3.8	49.8	126.1	0.6	14.2	98.9	1.8	20.1	113.2
	35-44			>74 y/o			35-44			>74 y/o		
1983-1987	3.8	39.9	125.4	2.8	99.5	127.0	0.6	10.5	89.0	5.6	123.4	213.9
1988-1992	2.6	26.7	112.6	3.2	89.8	139.3	1.4	11.5	118.9	6.0	102.5	214.1
1993-1997	3.6	34.9	142.5	4.2	91.0	174.8	0.8	8.9	105.1	3.8	54.7	159.1
1998-2002	3.6	44.5	126.2	3.4	67.0	127.0	0.4	9.1	87.9	4.4	49.1	158.2
2003-2007	3.2	49.3	122.9	4.0	61.1	135.8	0.4	11.4	90.0	5.8	48.6	216.4
2008-2012	2.4	39.8	107.1	3.6	49.5	119.4	1.0	14.7	108.6	4.6	32.4	168.6

Secondary Medical Zone ID: 048

	Male						Female					
	Suicide			Suicide			Suicide			Suicide		
	Num per year	Rate /100,000	×100	Num per year	Rate /100,000	×100	Num per year	Rate /100,000	×100	Num per year	Rate /100,000	×100
	Total (>10 y/o)			45-54			Total (>10 y/o)			45-54		
1983-1987	18.0	37.9	132.7	5.2	70.1	138.6	11.2	17.7	134.5	2.8	24.6	142.7
1988-1992	13.4	27.5	125.8	2.2	37.5	109.3	8.8	13.9	121.7	1.8	18.3	125.3
1993-1997	17.2	30.4	148.7	2.6	40.5	110.6	10.6	13.0	152.1	1.2	14.8	114.9
1998-2002	20.6	40.7	132.2	4.2	62.1	110.9	9.0	13.9	122.6	0.8	14.7	97.6
2003-2007	22.0	44.5	146.2	5.2	74.3	132.9	8.4	13.9	123.1	1.2	15.4	112.5
2008-2012	16.2	37.3	122.1	3.8	59.5	122.4	6.6	13.1	110.3	0.6	14.3	95.8
	15-24			55-64			15-24			55-64		
1983-1987	1.8	22.2	159.0	3.0	45.7	107.6	0.0	5.7	82.8	1.6	20.6	104.4
1988-1992	0.8	11.6	116.7	2.4	36.5	102.9	0.0	4.4	84.3	1.2	17.2	96.4
1993-1997	0.4	11.1	98.3	3.6	50.5	125.3	0.0	4.4	85.0	2.2	21.7	136.8
1998-2002	0.4	14.8	93.9	4.2	73.4	115.6	0.4	7.7	109.1	2.8	26.4	140.4
2003-2007	1.6	23.2	133.2	4.4	70.9	122.8	0.8	12.0	133.5	0.6	15.5	92.6
2008-2012	1.0	24.5	115.7	2.4	45.8	94.3	0.4	10.7	110.8	0.2	12.5	83.5
	25-34			65-74			25-34			65-74		
1983-1987	2.6	32.4	126.8	2.4	54.3	124.0	0.8	11.5	106.7	3.0	47.7	152.6
1988-1992	2.4	29.6	155.1	2.8	48.9	138.7	0.4	8.5	98.0	1.8	29.1	111.5
1993-1997	0.8	19.4	99.3	3.6	50.3	157.6	0.0	6.9	82.6	3.8	37.7	193.3
1998-2002	1.8	32.0	118.6	5.0	65.9	144.8	0.4	11.5	99.1	2.0	24.3	115.8
2003-2007	1.8	36.2	116.9	1.8	39.8	92.7	0.6	13.8	106.3	1.4	19.8	106.5
2008-2012	1.2	33.6	103.0	1.6	38.7	98.0	0.6	15.3	106.7	1.0	18.2	102.0
	35-44			>74 y/o			35-44			>74 y/o		
1983-1987	1.6	30.4	95.5	1.4	79.7	101.7	0.8	12.4	104.7	2.2	72.8	126.1
1988-1992	1.8	25.6	108.2	1.0	54.8	84.9	1.0	11.1	115.2	2.6	65.6	137.0
1993-1997	3.2	36.8	150.3	3.0	82.2	157.8	0.2	7.2	84.7	3.2	59.3	172.2
1998-2002	3.0	44.8	127.2	2.0	55.8	105.8	1.0	12.5	120.9	1.6	29.0	93.3
2003-2007	3.2	56.1	140.0	3.6	65.2	144.7	0.6	13.0	102.7	3.2	36.4	162.2
2008-2012	2.2	42.7	114.9	4.0	61.2	147.5	0.0	11.5	85.1	3.8	33.4	173.8

Secondary Medical Zone ID: 049

	Male						Female					
	Suicide			Suicide			Suicide			Suicide		
	Num per year	Rate /100,000	×100	Num per year	Rate /100,000	×100	Num per year	Rate /100,000	×100	Num per year	Rate /100,000	×100
	Total (>10 y/o)			45-54			Total (>10 y/o)			45-54		
1983-1987	66.4	29.0	86.3	17.8	47.6	94.2	52.4	17.2	118.1	7.0	17.5	101.8
1988-1992	64.4	25.0	102.0	11.2	30.9	90.1	40.0	12.3	98.8	5.8	15.3	104.3
1993-1997	68.0	25.3	100.6	15.0	36.4	99.5	33.8	10.0	94.6	4.8	12.4	96.1
1998-2002	85.0	32.2	85.9	22.6	51.8	92.6	41.0	11.8	97.5	4.0	11.3	75.0
2003-2007	101.2	37.1	100.8	20.6	51.6	92.3	38.2	11.8	95.9	3.0	9.5	69.5
2008-2012	81.8	32.6	89.9	17.4	49.6	102.1	37.0	12.3	95.5	3.2	10.9	72.7
	15-24			55-64			15-24			55-64		
1983-1987	3.6	12.0	85.9	12.8	39.1	92.0	4.0	10.0	145.7	7.4	18.9	95.6
1988-1992	3.4	10.0	101.4	13.2	35.7	100.6	1.4	4.5	85.6	6.4	16.1	90.0
1993-1997	3.4	10.2	90.1	14.6	40.4	100.4	2.4	6.1	117.3	4.8	13.1	82.7
1998-2002	3.8	12.7	80.2	15.2	45.7	71.9	3.0	8.1	115.9	6.2	17.5	92.9
2003-2007	5.6	18.3	104.7	25.0	62.6	108.4	2.8	9.1	101.0	6.4	16.6	98.8
2008-2012	6.6	24.4	115.2	16.2	39.6	81.6	4.4	14.2	146.3	4.4	12.0	80.2
	25-34			65-74			25-34			65-74		
1983-1987	7.2	19.8	77.3	5.8	31.6	72.2	2.4	7.4	68.9	11.6	43.2	138.1
1988-1992	6.8	19.6	102.7	9.0	37.8	107.3	2.2	7.1	82.2	9.2	29.1	111.6
1993-1997	8.2	23.8	121.8	8.8	30.5	95.5	1.2	5.4	65.2	5.0	14.4	74.0
1998-2002	8.8	26.3	97.2	12.0	38.3	84.2	3.4	10.7	92.6	5.8	16.0	76.0
2003-2007	13.2	36.6	118.1	10.8	35.1	81.7	3.2	10.7	82.3	6.2	17.1	91.9
2008-2012	8.0	27.0	82.8	10.2	34.1	86.4	3.8	13.1	91.2	5.4	16.3	91.6
	35-44			>74 y/o			35-44			>74 y/o		
1983-1987	12.6	30.4	95.6	6.4	65.5	83.6	4.0	10.6	90.1	15.8	90.8	157.5
1988-1992	12.4	27.4	115.7	8.2	61.1	94.8	2.4	7.1	73.2	12.4	55.0	115.0
1993-1997	9.8	24.2	98.7	8.2	50.8	97.6	3.6	8.9	104.7	12.0	43.2	125.4
1998-2002	13.8	37.8	107.3	8.6	43.6	82.7	2.6	8.2	79.6	15.8	44.2	142.3
2003-2007	14.2	42.1	105.0	11.2	43.3	96.1	4.6	13.4	105.7	11.6	26.1	116.5
2008-2012	12.8	37.8	101.6	10.6	35.4	85.2	3.4	11.5	84.8	12.4	23.7	123.4

Secondary Medical Zone ID: 050

	Male						Female					
	Suicide			Suicide			Suicide			Suicide		
	Num per year	Rate /100,000	×100	Num per year	Rate /100,000	×100	Num per year	Rate /100,000	×100	Num per year	Rate /100,000	×100
	Total (>10 y/o)			45-54			Total (>10 y/o)			45-54		
1983-1987	17.2	35.5	117.6	3.8	53.7	106.1	7.6	15.1	96.9	0.8	14.9	86.3
1988-1992	15.4	27.8	128.9	2.6	38.0	110.5	11.6	15.2	138.8	0.6	13.3	90.7
1993-1997	15.8	28.2	126.8	3.6	43.7	119.5	8.0	11.4	116.9	0.8	12.9	99.5
1998-2002	26.8	43.6	147.7	4.4	57.6	103.0	10.8	13.9	129.7	1.6	17.2	114.4
2003-2007	28.6	48.5	163.1	8.0	93.3	166.9	12.6	14.6	149.3	0.6	12.4	90.7
2008-2012	20.6	38.2	131.1	3.0	50.2	103.4	10.4	14.1	131.8	0.8	14.6	98.2
	15-24			55-64			15-24			55-64		
1983-1987	1.0	15.6	112.0	4.0	52.0	122.3	0.0	5.4	79.7	0.8	15.8	80.0
1988-1992	1.2	12.6	127.2	3.8	46.0	129.6	0.2	5.0	95.5	1.4	17.6	98.6
1993-1997	0.4	10.6	93.4	3.0	43.7	108.6	0.0	4.3	81.6	1.6	18.1	113.9
1998-2002	1.4	19.0	120.0	5.4	79.5	125.2	0.4	7.4	105.1	1.4	19.8	105.2
2003-2007	1.4	20.9	119.9	6.6	83.4	144.5	0.2	8.2	90.9	1.2	17.5	104.5
2008-2012	1.8	28.0	131.8	3.0	45.9	94.6	0.4	10.2	105.0	1.4	16.4	109.9
	25-34			65-74			25-34			65-74		
1983-1987	2.8	30.9	121.1	1.2	38.6	88.1	1.2	12.6	117.0	2.6	41.4	132.5
1988-1992	2.2	26.0	136.3	2.0	39.6	112.3	0.6	8.9	102.7	3.2	40.5	155.1
1993-1997	1.6	23.3	119.3	1.8	32.3	101.3	0.2	7.4	89.0	3.0	30.6	157.2
1998-2002	2.6	35.0	129.7	4.6	61.3	134.7	0.2	10.3	88.5	2.6	27.0	128.7
2003-2007	3.4	45.1	145.6	3.2	50.6	118.0	0.6	13.2	101.8	3.4	30.2	162.0
2008-2012	1.0	30.4	93.5	3.8	54.1	136.9	0.6	14.7	102.3	1.4	19.6	110.3
	35-44			>74 y/o			35-44			>74 y/o		
1983-1987	2.4	33.3	104.5	2.0	97.3	124.2	0.4	10.4	88.0	1.8	61.3	106.3
1988-1992	1.2	19.7	83.2	2.4	85.2	132.1	0.2	8.0	82.7	5.4	110.2	230.3
1993-1997	2.2	27.2	111.1	3.2	84.6	162.5	0.4	7.7	90.7	2.0	39.2	114.0
1998-2002	4.2	51.2	145.4	4.0	83.6	158.7	0.4	9.4	90.6	4.0	53.5	172.2
2003-2007	2.8	48.3	120.6	3.0	55.9	124.2	1.4	16.0	126.5	5.0	49.5	220.5
2008-2012	2.8	44.6	119.9	5.2	71.0	171.2	0.8	14.5	106.8	5.0	39.6	205.9

Secondary Medical Zone ID: 051

	Male						Female					
	Suicide			Suicide			Suicide			Suicide		
	Num per year	Rate /100,000	×100	Num per year	Rate /100,000	×100	Num per year	Rate /100,000	×100	Num per year	Rate /100,000	×100
	Total (>10 y/o)			45-54			Total (>10 y/o)			45-54		
1983-1987	40.8	36.3	114.1	11.6	64.7	128.0	21.2	15.5	102.9	3.6	18.7	108.7
1988-1992	32.6	27.2	114.2	7.0	41.4	120.5	23.2	13.9	120.3	1.6	12.5	85.8
1993-1997	35.0	27.4	116.4	8.8	45.9	125.4	20.4	11.4	121.9	1.4	11.0	85.4
1998-2002	47.6	37.9	110.3	14.0	70.0	125.0	21.6	12.6	114.1	1.6	12.4	82.5
2003-2007	51.2	42.7	119.6	13.0	72.1	128.9	15.8	11.8	95.8	2.0	13.1	95.6
2008-2012	50.0	40.3	127.4	8.8	56.5	116.3	15.8	12.5	99.7	2.2	15.3	102.7
	15-24			55-64			15-24			55-64		
1983-1987	2.6	16.2	116.1	6.8	42.6	100.1	0.4	5.3	76.9	3.4	18.2	92.2
1988-1992	2.4	12.3	124.6	5.0	31.2	88.0	0.8	5.5	103.9	3.8	18.9	105.7
1993-1997	1.0	9.1	80.5	9.0	51.7	128.4	0.8	5.5	105.4	1.6	11.9	74.9
1998-2002	1.2	11.9	75.5	8.0	56.2	88.4	0.2	5.0	71.8	3.2	19.6	104.1
2003-2007	2.2	17.0	97.3	9.0	55.7	96.5	0.6	7.4	82.5	2.6	16.7	99.7
2008-2012	2.4	21.3	100.6	10.4	54.8	112.8	0.2	6.6	67.8	1.4	12.0	80.4
	25-34			65-74			25-34			65-74		
1983-1987	4.8	27.0	105.8	3.6	40.2	91.9	1.6	10.2	94.7	3.0	26.2	83.7
1988-1992	4.4	25.6	133.8	3.8	34.7	98.5	1.0	7.9	91.1	5.6	33.8	129.5
1993-1997	3.6	23.2	118.7	6.0	41.2	129.1	0.6	6.8	82.1	5.6	28.0	143.5
1998-2002	3.0	24.2	89.7	8.6	55.3	121.6	0.8	9.5	82.1	4.6	23.9	113.8
2003-2007	5.0	34.6	111.7	6.8	47.5	110.6	1.4	12.4	95.3	1.6	13.4	71.9
2008-2012	5.0	37.1	113.8	6.8	47.7	120.8	2.0	15.9	110.9	2.0	15.9	89.1
	35-44			>74 y/o			35-44			>74 y/o		
1983-1987	7.8	40.6	127.6	3.6	76.4	97.5	1.8	11.5	97.7	7.4	82.6	143.2
1988-1992	4.8	24.8	104.5	5.2	80.8	125.3	1.2	8.4	87.5	9.0	78.3	163.6
1993-1997	3.2	20.1	81.8	3.4	48.9	93.8	1.2	7.9	93.0	9.2	64.9	188.6
1998-2002	6.6	40.5	115.1	6.2	65.3	123.8	1.4	10.0	97.1	9.8	54.6	175.7
2003-2007	9.8	62.6	156.3	5.4	47.3	105.0	1.4	11.9	93.9	6.2	28.8	128.3
2008-2012	8.2	50.5	135.7	8.2	57.5	138.5	2.0	14.5	107.0	6.0	24.3	126.2

Secondary Medical Zone ID: 052

	Male						Female					
	Suicide			Suicide			Suicide			Suicide		
	Num per year	Rate /100,000	×100	Num per year	Rate /100,000	×100	Num per year	Rate /100,000	×100	Num per year	Rate /100,000	×100
	Total (>10 y/o)			45-54			Total (>10 y/o)			45-54		
1983-1987	64.2	42.4	139.5	19.0	80.8	159.8	35.4	18.1	127.4	5.4	19.9	115.5
1988-1992	48.4	30.0	132.1	12.0	52.9	153.9	30.4	14.1	120.2	4.0	16.2	110.9
1993-1997	52.0	30.6	134.5	13.8	53.8	146.9	28.6	12.6	128.7	2.8	12.5	97.1
1998-2002	75.0	44.4	133.6	17.2	66.8	119.4	31.6	13.7	124.0	2.8	13.3	88.2
2003-2007	89.6	52.9	161.7	22.6	94.7	169.3	28.4	14.3	121.1	4.2	16.8	122.2
2008-2012	64.0	41.0	128.8	11.6	58.1	119.6	24.8	13.1	112.2	2.4	13.7	92.1
	15-24			55-64			15-24			55-64		
1983-1987	3.0	16.3	117.0	10.8	51.6	121.3	1.8	8.5	123.9	8.0	28.2	143.1
1988-1992	1.8	10.1	102.3	11.2	48.0	135.1	1.0	5.5	104.9	7.2	24.3	136.2
1993-1997	0.6	7.6	67.0	13.0	57.2	142.1	1.0	5.6	106.9	6.0	21.6	136.1
1998-2002	3.6	19.0	120.3	16.4	79.7	125.4	0.8	6.4	90.6	4.4	19.3	102.6
2003-2007	2.2	16.5	94.5	21.4	90.9	157.4	1.8	11.4	126.5	4.2	17.7	105.9
2008-2012	3.6	25.5	120.4	17.6	66.6	137.1	0.0	5.5	56.3	4.0	16.0	107.1
	25-34			65-74			25-34			65-74		
1983-1987	7.4	30.8	120.6	6.6	51.8	118.4	2.6	11.4	105.5	6.4	36.7	117.4
1988-1992	3.2	17.6	92.2	7.2	47.4	134.6	1.4	8.0	92.2	4.8	24.9	95.3
1993-1997	3.8	21.2	108.3	9.0	47.9	150.2	1.8	9.3	111.7	5.4	22.4	115.1
1998-2002	7.4	36.1	133.8	9.8	50.2	110.4	2.2	12.3	105.9	8.6	31.2	148.5
2003-2007	10.4	50.4	162.5	11.4	57.8	134.7	2.4	14.0	107.5	5.8	22.8	122.5
2008-2012	7.2	41.8	128.3	7.8	44.0	111.4	2.8	17.0	119.0	3.8	18.0	100.9
	35-44			>74 y/o			35-44			>74 y/o		
1983-1987	10.4	41.6	130.7	6.8	104.0	132.8	2.6	11.5	97.1	8.6	78.8	136.6
1988-1992	5.8	23.3	98.2	7.2	86.3	133.8	1.6	8.1	83.8	10.4	71.3	149.1
1993-1997	6.2	26.3	107.5	5.4	57.8	110.9	2.2	9.3	109.5	9.4	52.9	153.6
1998-2002	9.8	45.9	130.3	10.4	83.9	159.2	1.8	9.8	94.6	11.0	49.3	158.8
2003-2007	11.4	59.3	147.9	10.0	64.4	142.9	2.6	13.7	108.8	7.4	27.3	121.8
2008-2012	6.6	38.2	102.6	9.4	52.3	126.0	2.8	15.0	110.5	9.0	27.5	142.9

Secondary Medical Zone ID: 053

	Male						Female					
	Suicide			Suicide			Suicide			Suicide		
	Num per year	Rate /100,000	×100	Num per year	Rate /100,000	×100	Num per year	Rate /100,000	×100	Num per year	Rate /100,000	×100
	Total (>10 y/o)			45–54			Total (>10 y/o)			45–54		
1983–1987	60.8	30.9	92.4	12.8	41.7	82.5	38.2	15.7	104.6	4.2	13.9	80.4
1988–1992	53.0	24.5	98.3	12.4	37.9	110.3	30.6	11.7	91.6	3.6	12.3	84.0
1993–1997	52.0	23.4	89.6	11.2	31.8	87.0	29.8	10.4	98.3	4.0	11.8	91.2
1998–2002	81.4	34.4	93.8	19.0	49.7	88.8	34.4	11.7	95.6	6.6	16.6	110.2
2003–2007	96.4	40.9	109.3	21.0	58.7	105.0	35.2	12.5	101.2	4.6	13.2	96.7
2008–2012	80.8	34.8	100.6	14.4	47.1	97.0	35.0	13.4	102.6	5.8	17.4	116.6
	15–24			55–64			15–24			55–64		
1983–1987	5.2	16.5	118.3	7.6	30.1	70.7	2.2	6.8	99.8	6.8	20.8	105.6
1988–1992	5.2	13.6	137.3	6.8	24.1	67.9	1.8	5.2	99.4	4.0	13.4	75.3
1993–1997	4.0	11.6	102.8	11.2	37.2	92.5	1.4	4.5	85.7	4.4	13.9	87.6
1998–2002	4.6	15.2	95.9	16.0	53.3	83.9	1.0	4.7	67.2	5.4	17.3	91.9
2003–2007	5.8	20.0	114.4	19.0	54.7	94.8	2.6	9.3	102.9	7.8	20.2	120.4
2008–2012	5.4	22.3	105.4	16.4	44.1	90.8	3.2	12.2	125.7	6.2	15.9	106.3
	25–34			65–74			25–34			65–74		
1983–1987	9.8	28.0	109.4	5.6	38.5	87.9	4.0	11.2	104.4	6.2	30.8	98.5
1988–1992	6.4	20.5	107.3	7.2	37.5	106.4	1.4	6.1	69.8	7.2	28.5	109.3
1993–1997	6.6	21.4	109.5	6.2	26.6	83.4	2.2	7.7	92.6	4.8	16.7	85.6
1998–2002	8.8	27.7	102.6	10.2	38.7	85.2	2.6	9.6	82.7	5.8	18.6	88.4
2003–2007	12.6	38.3	123.5	10.2	38.4	89.5	3.8	12.6	96.9	5.2	17.0	91.2
2008–2012	10.4	36.7	112.5	9.4	35.7	90.4	3.4	13.4	93.5	4.8	16.4	92.0
	35–44			>74 y/o			35–44			>74 y/o		
1983–1987	13.8	36.5	114.6	6.0	70.4	89.9	4.6	12.4	105.4	10.2	75.8	131.5
1988–1992	9.8	24.8	104.6	5.0	50.0	77.6	3.8	9.9	102.4	8.6	50.4	105.4
1993–1997	9.8	26.7	108.8	3.0	28.4	54.6	3.2	8.8	103.9	9.8	45.3	131.5
1998–2002	13.6	40.9	116.1	9.0	55.6	105.6	2.2	8.1	78.3	10.6	38.1	122.8
2003–2007	18.8	58.6	146.3	8.6	41.6	92.3	2.6	10.1	80.0	8.4	24.2	107.7
2008–2012	12.2	39.0	104.8	12.2	47.2	113.8	4.0	13.2	97.2	7.6	18.7	97.3

Secondary Medical Zone ID: 054

	Male						Female					
	Suicide			Suicide			Suicide			Suicide		
	Num per year	Rate /100,000	×100	Num per year	Rate /100,000	×100	Num per year	Rate /100,000	×100	Num per year	Rate /100,000	×100
	Total (>10 y/o)			45-54			Total (>10 y/o)			45-54		
1983-1987	70.4	33.8	105.3	15.0	48.0	94.9	39.4	16.3	108.9	5.2	15.9	92.2
1988-1992	56.2	24.9	102.2	9.2	29.4	85.5	42.4	14.7	123.5	6.6	18.1	124.0
1993-1997	63.6	25.9	104.1	12.0	32.4	88.5	32.0	10.7	103.8	5.2	13.9	108.0
1998-2002	93.0	36.1	100.5	21.8	51.6	92.2	38.2	12.4	102.4	4.2	12.0	79.6
2003-2007	126.4	46.1	130.6	34.6	81.9	146.5	39.8	13.1	107.7	5.0	13.0	94.8
2008-2012	102.0	38.2	113.7	20.0	53.5	110.2	41.6	13.3	111.7	6.6	17.2	115.5
	15-24			55-64			15-24			55-64		
1983-1987	7.8	20.3	145.7	10.2	38.8	91.3	1.8	5.9	86.8	7.6	23.3	118.3
1988-1992	5.0	12.2	123.1	11.6	37.8	106.5	1.0	3.8	71.4	7.2	20.3	113.7
1993-1997	6.0	13.9	123.3	13.0	42.5	105.5	1.6	4.7	90.3	4.2	13.5	84.8
1998-2002	6.2	16.2	102.8	17.6	57.8	91.0	2.6	7.4	104.8	7.0	20.7	109.8
2003-2007	6.2	18.0	102.9	24.6	66.6	115.4	3.6	10.8	120.1	6.0	16.9	100.8
2008-2012	6.2	21.2	100.1	23.6	55.8	115.0	1.6	7.3	75.6	6.2	15.3	102.9
	25-34			65-74			25-34			65-74		
1983-1987	10.2	25.5	99.6	7.0	47.7	108.9	4.2	10.6	98.5	7.8	38.1	121.9
1988-1992	7.6	20.5	107.1	7.2	38.9	110.5	3.2	8.8	101.1	10.4	40.5	155.3
1993-1997	9.4	25.0	127.8	7.2	31.3	98.0	2.2	7.1	84.8	4.6	16.6	85.2
1998-2002	11.0	29.2	108.1	12.2	46.2	101.7	4.2	11.8	101.7	6.0	19.2	91.6
2003-2007	18.0	44.8	144.3	14.4	52.4	122.0	4.6	13.0	100.1	7.6	22.5	120.8
2008-2012	14.4	40.4	123.9	9.6	36.8	93.2	4.4	13.9	97.4	5.6	18.3	102.6
	35-44			>74 y/o			35-44			>74 y/o		
1983-1987	16.0	40.2	126.2	4.0	61.2	78.2	4.8	12.8	108.3	8.0	66.1	114.6
1988-1992	11.4	26.2	110.3	4.2	49.8	77.3	3.2	8.5	88.4	10.6	64.5	134.8
1993-1997	10.8	25.6	104.5	4.8	44.2	84.9	4.0	9.5	112.1	10.0	47.8	138.9
1998-2002	17.4	43.8	124.2	6.8	47.4	90.0	3.0	8.8	84.9	11.2	41.9	134.9
2003-2007	19.0	51.5	128.5	9.6	48.6	108.0	4.0	11.8	93.8	9.0	26.4	117.6
2008-2012	16.2	43.4	116.7	11.8	48.5	116.9	5.4	14.8	109.2	11.8	27.7	144.1

Secondary Medical Zone ID: 055

	Male						Female					
	Suicide			Suicide			Suicide			Suicide		
	Num per year	Rate /100,000	×100	Num per year	Rate /100,000	×100	Num per year	Rate /100,000	×100	Num per year	Rate /100,000	×100
	Total (>10 y/o)			45-54			Total (>10 y/o)			45-54		
1983-1987	24.4	37.3	122.2	4.6	51.3	101.5	10.4	15.0	97.7	1.6	17.0	98.6
1988-1992	16.6	25.1	103.8	2.4	30.3	88.2	10.8	13.2	105.7	1.8	16.5	113.1
1993-1997	19.0	26.1	107.9	3.6	35.4	96.8	13.0	12.4	132.1	1.8	15.0	116.1
1998-2002	30.2	38.4	113.7	6.6	54.9	98.0	13.4	13.4	118.1	1.4	14.3	94.9
2003-2007	35.0	42.7	126.9	10.0	76.7	137.1	12.0	13.3	111.9	1.6	14.0	102.2
2008-2012	28.0	36.3	110.0	5.0	48.4	99.6	11.8	13.3	110.5	1.0	13.1	87.8
	15-24			55-64			15-24			55-64		
1983-1987	4.2	30.6	219.7	3.2	40.2	94.7	0.4	6.3	92.4	2.0	20.0	101.5
1988-1992	1.0	10.4	105.2	4.2	41.5	116.8	0.6	5.8	110.2	1.2	15.1	84.4
1993-1997	0.6	9.8	86.4	3.8	42.0	104.2	0.2	4.4	83.6	1.2	14.4	90.4
1998-2002	3.8	25.4	160.9	7.0	74.1	116.7	1.0	8.3	118.7	2.0	20.2	107.4
2003-2007	2.4	21.5	123.4	6.6	62.5	108.3	0.6	8.6	95.6	3.0	21.7	129.4
2008-2012	1.6	21.7	102.4	5.6	47.8	98.4	0.6	9.5	98.0	2.4	17.2	115.6
	25-34			65-74			25-34			65-74		
1983-1987	4.6	34.1	133.5	2.6	50.2	114.8	0.8	9.3	86.8	2.0	31.5	100.8
1988-1992	2.2	20.5	107.5	1.6	31.8	90.3	1.0	9.2	106.5	2.0	26.3	100.8
1993-1997	2.0	20.5	104.9	2.6	34.5	108.0	1.2	10.0	120.2	2.4	23.2	118.9
1998-2002	2.6	27.5	101.7	3.2	42.5	93.4	0.6	10.2	88.3	2.2	21.6	102.6
2003-2007	4.0	36.6	118.0	3.0	40.6	94.6	1.2	13.4	103.1	2.2	20.8	111.7
2008-2012	3.8	36.6	112.3	3.8	44.5	112.6	1.6	16.1	112.1	2.0	20.0	112.6
	35-44			>74 y/o			35-44			>74 y/o		
1983-1987	3.4	32.8	103.0	1.8	75.6	96.5	1.4	13.0	109.9	2.2	54.9	95.1
1988-1992	3.4	26.3	111.1	1.6	57.5	89.2	0.8	8.9	92.3	3.4	59.2	123.6
1993-1997	3.6	27.9	113.7	2.6	63.6	122.2	1.0	8.7	102.4	5.0	65.0	189.0
1998-2002	3.4	33.2	94.3	3.6	68.4	129.7	1.2	11.0	106.0	5.0	52.2	168.1
2003-2007	4.8	47.3	118.1	4.2	59.9	132.9	1.0	12.3	97.1	2.4	22.6	100.6
2008-2012	4.4	41.9	112.6	3.6	46.5	112.2	0.8	12.4	91.6	3.4	24.3	126.5

Secondary Medical Zone ID: 056

	Male						Female					
	Suicide			Suicide			Suicide			Suicide		
	Num per year	Rate /100,000	×100	Num per year	Rate /100,000	×100	Num per year	Rate /100,000	×100	Num per year	Rate /100,000	×100
	Total (>10 y/o)			45–54			Total (>10 y/o)			45–54		
1983–1987	52.2	38.9	124.7	14.6	69.1	136.7	23.6	14.8	97.2	4.4	18.8	109.2
1988–1992	29.2	23.2	90.1	7.6	39.8	115.8	18.6	11.3	85.3	2.4	13.6	92.7
1993–1997	31.0	23.7	91.4	7.2	36.4	99.5	13.4	9.1	74.1	2.4	12.8	99.2
1998–2002	54.4	38.2	109.5	14.2	63.2	112.9	17.6	11.6	83.8	2.8	14.5	96.4
2003–2007	63.4	45.5	129.6	16.2	76.4	136.5	17.6	12.1	90.7	3.0	14.7	107.1
2008–2012	47.8	37.2	110.3	10.0	55.2	113.6	18.0	12.9	96.9	2.2	13.9	93.3
	15–24			55–64			15–24			55–64		
1983–1987	2.6	15.6	111.7	8.6	44.3	104.4	0.4	4.9	71.4	4.6	19.9	100.9
1988–1992	1.4	9.7	97.8	6.2	31.8	89.4	0.6	4.6	88.0	4.6	19.0	106.3
1993–1997	1.2	9.7	85.6	7.4	39.9	99.1	0.4	4.1	78.6	2.0	11.7	73.5
1998–2002	2.4	15.9	100.4	10.6	62.2	97.8	0.8	6.4	90.8	2.8	16.7	88.8
2003–2007	3.2	20.5	117.4	12.6	65.3	113.1	1.2	9.1	101.1	3.0	16.5	98.7
2008–2012	2.8	22.8	107.5	11.8	54.1	111.4	1.4	11.0	113.5	4.4	18.4	123.7
	25–34			65–74			25–34			65–74		
1983–1987	8.0	35.5	138.7	5.4	46.8	106.9	1.8	9.8	91.3	4.4	29.6	94.6
1988–1992	3.2	18.5	96.7	2.4	22.6	64.1	1.4	8.4	96.5	3.8	22.0	84.1
1993–1997	3.6	21.6	110.5	3.8	25.4	79.6	1.4	8.6	103.5	3.0	15.4	79.0
1998–2002	5.4	32.2	119.3	8.8	49.3	108.4	2.0	12.5	108.0	3.8	18.3	87.2
2003–2007	6.4	39.4	127.1	8.8	51.4	119.9	1.4	11.8	90.8	1.6	11.8	63.2
2008–2012	4.6	34.3	105.2	7.4	46.6	118.1	2.0	15.3	106.7	2.6	16.2	91.1
	35–44			>74 y/o			35–44			>74 y/o		
1983–1987	8.0	37.8	118.7	5.0	87.2	111.4	2.6	12.8	108.2	5.2	53.1	92.1
1988–1992	5.2	23.6	99.8	3.0	47.1	73.1	0.4	6.0	61.7	5.4	41.8	87.3
1993–1997	4.2	21.3	86.8	3.6	43.5	83.6	0.8	6.2	72.5	3.4	22.7	65.9
1998–2002	6.6	36.1	102.5	6.2	56.0	106.2	1.4	9.2	88.7	4.0	21.1	67.9
2003–2007	10.0	58.6	146.3	6.2	45.4	100.9	2.0	12.8	101.4	5.4	21.7	96.5
2008–2012	6.0	40.5	108.9	5.2	35.5	85.6	1.2	11.6	85.4	4.2	15.5	80.7

Secondary Medical Zone ID: 057

	Male						Female					
	Suicide			Suicide			Suicide			Suicide		
	Num per year	Rate /100,000	×100	Num per year	Rate /100,000	×100	Num per year	Rate /100,000	×100	Num per year	Rate /100,000	×100
	Total (>10 y/o)			45–54			Total (>10 y/o)			45–54		
1983–1987	7.4	33.8	113.4	0.6	39.0	77.1	5.2	16.6	120.3	0.6	17.7	102.6
1988–1992	6.0	26.2	116.5	0.6	31.5	91.7	3.8	13.1	108.6	0.2	13.8	94.7
1993–1997	6.0	26.3	115.5	1.4	40.9	111.8	3.2	10.9	108.8	0.0	11.6	89.6
1998–2002	7.4	36.6	108.6	1.6	58.2	104.0	2.4	12.3	94.2	0.6	16.3	108.3
2003–2007	10.0	41.4	138.9	2.0	66.4	118.8	4.2	13.2	120.4	0.4	14.3	104.1
2008–2012	7.8	37.3	121.1	1.4	52.7	108.5	2.4	12.7	101.8	0.2	14.6	97.6
	15–24			55–64			15–24			55–64		
1983–1987	0.2	13.5	96.6	1.2	42.7	100.6	0.0	6.3	92.4	1.0	22.1	112.1
1988–1992	0.4	11.0	110.9	1.6	41.4	116.6	0.0	4.9	93.6	0.4	16.9	94.4
1993–1997	0.0	10.5	92.8	1.2	41.2	102.4	0.0	4.9	93.9	0.4	15.5	97.5
1998–2002	0.6	18.0	114.2	1.0	57.1	89.8	0.0	6.6	94.4	0.2	17.4	92.7
2003–2007	0.0	16.2	92.7	2.0	66.0	114.3	0.2	9.6	107.0	0.6	17.8	106.3
2008–2012	1.0	27.0	127.3	1.6	52.3	107.7	0.0	9.1	93.9	0.4	15.2	102.2
	25–34			65–74			25–34			65–74		
1983–1987	1.8	36.3	142.2	1.2	50.8	116.0	0.0	9.4	87.0	1.2	36.8	117.9
1988–1992	1.0	25.0	130.6	0.6	33.8	95.8	0.4	9.9	113.8	1.2	31.8	121.8
1993–1997	0.6	21.6	110.4	0.8	32.5	101.9	0.2	8.6	103.8	0.4	17.6	90.4
1998–2002	0.4	26.7	98.8	0.8	41.3	90.8	0.0	10.8	93.3	0.6	20.7	98.7
2003–2007	0.8	34.2	110.1	1.8	51.7	120.6	0.4	14.3	109.7	0.2	16.4	88.3
2008–2012	0.8	35.7	109.6	1.4	45.1	114.2	0.0	13.5	94.4	0.4	17.7	99.4
	35–44			>74 y/o			35–44			>74 y/o		
1983–1987	0.6	29.9	94.1	1.8	109.2	139.4	0.4	12.4	105.3	2.0	87.2	151.1
1988–1992	1.2	29.0	122.4	0.6	58.1	90.1	0.4	10.4	107.8	1.2	51.4	107.5
1993–1997	0.8	26.7	108.8	1.2	63.6	122.1	0.0	7.6	89.1	2.2	60.5	175.7
1998–2002	1.0	38.0	108.0	2.0	75.2	142.7	0.2	10.4	100.6	0.8	28.0	90.2
2003–2007	2.0	58.5	146.1	1.4	52.4	116.3	0.2	12.6	100.1	2.2	39.9	178.0
2008–2012	1.0	40.4	108.7	0.6	35.9	86.4	0.0	12.7	93.8	1.4	24.8	128.8

Secondary Medical Zone ID: 058

	Male						Female					
	Suicide			Suicide			Suicide			Suicide		
	Num per year	Rate /100,000	×100	Num per year	Rate /100,000	×100	Num per year	Rate /100,000	×100	Num per year	Rate /100,000	×100
	Total (>10 y/o)			45-54			Total (>10 y/o)			45-54		
1983-1987	34.4	37.3	120.4	8.8	63.4	125.5	18.8	17.0	116.4	3.6	21.5	124.5
1988-1992	28.8	28.1	123.8	5.0	37.6	109.4	16.6	13.7	112.5	1.6	13.9	94.8
1993-1997	25.2	25.3	103.1	6.8	41.7	114.0	13.2	10.9	103.3	1.6	12.4	96.3
1998-2002	46.0	42.1	126.1	10.8	62.7	112.0	18.2	13.5	117.2	3.6	18.9	125.5
2003-2007	39.2	38.6	109.4	6.8	47.2	84.5	15.0	12.4	106.2	2.2	14.4	105.1
2008-2012	33.8	35.0	103.0	3.6	35.5	73.1	12.8	12.0	97.6	1.4	13.4	89.7
	15-24			55-64			15-24			55-64		
1983-1987	3.0	20.5	146.9	6.0	47.6	112.1	0.4	5.7	83.5	2.2	17.4	88.1
1988-1992	1.0	9.4	95.0	5.2	38.7	109.0	0.8	5.9	110.9	3.4	20.5	114.8
1993-1997	1.6	11.9	104.8	4.8	39.1	97.0	0.0	3.4	64.4	3.0	19.0	119.8
1998-2002	2.6	18.5	117.1	9.4	72.9	114.8	0.4	5.9	83.3	2.6	19.6	104.4
2003-2007	2.8	21.5	123.1	11.4	73.8	127.9	0.8	8.8	97.5	2.2	16.5	98.8
2008-2012	2.6	24.5	115.4	8.6	51.0	105.1	0.4	7.9	81.9	2.4	15.3	102.3
	25-34			65-74			25-34			65-74		
1983-1987	4.6	28.2	110.3	3.2	45.0	102.7	1.6	10.8	100.3	1.6	22.1	70.8
1988-1992	4.0	26.1	136.5	5.0	50.3	142.8	1.2	9.0	103.8	3.2	27.8	106.5
1993-1997	1.6	16.3	83.6	2.8	28.8	90.3	1.0	8.5	102.7	2.8	20.5	105.2
1998-2002	5.0	34.5	127.9	6.0	50.5	111.0	0.6	9.4	81.2	3.8	24.6	117.0
2003-2007	6.0	41.5	133.8	2.6	30.7	71.5	0.8	10.8	83.1	3.2	21.7	116.6
2008-2012	5.2	39.2	120.4	6.2	50.2	127.2	1.0	12.8	89.4	2.2	18.2	102.4
	35-44			>74 y/o			35-44			>74 y/o		
1983-1987	6.4	38.9	122.2	2.4	69.3	88.5	2.4	14.2	120.6	7.0	106.3	184.2
1988-1992	4.8	27.0	113.7	3.8	75.7	117.3	2.0	11.1	114.7	4.4	54.5	114.0
1993-1997	3.8	24.3	99.1	3.8	62.7	120.4	1.2	8.4	98.4	3.6	36.4	105.8
1998-2002	7.4	49.2	139.6	4.8	64.0	121.3	1.8	12.0	116.1	5.4	41.2	132.8
2003-2007	4.2	37.6	93.8	5.2	55.0	122.2	0.8	10.6	84.0	5.0	30.4	135.4
2008-2012	4.6	38.3	103.1	3.0	33.5	80.7	0.8	11.5	84.5	4.6	23.9	124.1

Secondary Medical Zone ID: 059

	Male						Female					
	Suicide			Suicide			Suicide			Suicide		
	Num per year	Rate /100,000	×100	Num per year	Rate /100,000	×100	Num per year	Rate /100,000	×100	Num per year	Rate /100,000	×100
	Total (>10 y/o)			45-54			Total (>10 y/o)			45-54		
1983-1987	42.8	31.0	94.0	12.0	52.5	103.7	28.0	16.3	109.9	3.2	14.7	85.0
1988-1992	38.8	25.1	102.4	10.4	42.2	122.8	27.0	13.7	111.8	3.6	14.8	101.1
1993-1997	43.6	26.1	105.7	9.6	36.7	100.3	28.8	12.9	129.5	3.6	13.5	104.8
1998-2002	69.6	39.6	113.1	16.0	57.9	103.4	25.6	12.7	100.9	3.6	14.2	94.1
2003-2007	76.6	44.4	123.3	15.2	60.2	107.6	24.6	13.2	101.6	4.2	15.7	114.6
2008-2012	66.6	39.2	117.7	10.2	47.5	97.7	24.4	13.3	103.1	3.0	14.3	96.0
	15-24			55-64			15-24			55-64		
1983-1987	2.0	11.4	82.1	6.2	34.7	81.6	1.4	6.7	98.2	5.4	22.6	114.4
1988-1992	1.8	9.0	90.6	7.4	34.6	97.4	1.6	6.3	119.2	5.4	20.9	117.0
1993-1997	3.0	12.3	108.5	10.8	47.5	117.9	1.2	5.4	102.7	3.4	14.8	92.8
1998-2002	2.8	14.3	90.7	17.0	73.6	115.8	1.6	7.6	107.9	3.6	16.6	88.2
2003-2007	5.0	23.0	131.6	16.2	63.6	110.2	2.8	13.0	144.4	4.0	16.1	96.3
2008-2012	4.8	26.3	124.0	14.6	53.6	110.4	1.8	10.9	112.9	3.4	13.7	92.2
	25-34			65-74			25-34			65-74		
1983-1987	6.4	26.1	102.3	3.4	34.4	78.5	3.8	13.7	127.5	5.0	33.2	106.2
1988-1992	3.4	16.6	86.9	4.4	33.6	95.3	2.0	8.9	102.5	5.6	29.8	114.0
1993-1997	4.2	19.4	99.4	5.0	30.8	96.5	2.0	8.9	106.5	6.4	27.8	142.7
1998-2002	8.4	33.7	124.8	9.8	51.3	112.9	3.0	12.7	109.7	5.2	22.6	107.5
2003-2007	9.0	38.0	122.5	9.2	47.2	110.0	3.2	14.3	109.9	4.0	17.9	96.2
2008-2012	6.4	33.4	102.7	10.4	49.4	124.9	2.4	13.8	96.3	6.0	23.2	130.1
	35-44			>74 y/o			35-44			>74 y/o		
1983-1987	7.4	29.1	91.3	4.8	88.9	113.5	3.4	12.5	106.2	5.8	69.5	120.5
1988-1992	6.2	22.7	95.9	5.2	71.3	110.5	3.0	10.5	108.4	5.8	49.9	104.2
1993-1997	7.8	29.1	118.7	3.2	40.7	78.1	4.2	13.5	158.4	8.0	50.8	147.7
1998-2002	10.6	43.8	124.3	4.8	46.6	88.4	2.2	10.1	97.3	6.4	33.1	106.7
2003-2007	15.6	65.4	163.3	6.2	45.3	100.6	2.4	11.9	94.2	4.0	17.7	78.9
2008-2012	13.2	51.8	139.3	6.8	42.3	102.0	3.6	15.2	112.3	4.2	16.0	83.1

Secondary Medical Zone ID: 060

	Male						Female					
	Suicide			Suicide			Suicide			Suicide		
	Num per year	Rate /100,000	×100	Num per year	Rate /100,000	×100	Num per year	Rate /100,000	×100	Num per year	Rate /100,000	×100
	Total (>10 y/o)			45–54			Total (>10 y/o)			45–54		
1983–1987	61.4	33.5	105.3	12.2	44.0	87.1	32.2	15.7	103.9	5.0	17.6	102.0
1988–1992	44.2	23.1	92.3	10.6	34.2	99.6	33.8	14.0	115.0	6.6	19.0	129.9
1993–1997	52.2	24.6	98.3	12.8	36.4	99.4	23.2	9.9	89.4	4.8	13.7	105.9
1998–2002	76.0	34.5	95.1	18.2	51.0	91.2	32.2	13.0	100.5	5.2	14.9	98.7
2003–2007	88.0	39.5	106.7	19.2	58.0	103.7	34.8	13.5	109.0	4.8	14.4	105.2
2008–2012	77.0	34.9	100.4	14.2	47.9	98.5	36.4	14.5	112.7	5.0	16.3	109.2
	15–24			55–64			15–24			55–64		
1983–1987	5.8	18.2	130.6	9.4	43.0	101.3	1.8	6.3	91.5	6.0	22.8	115.6
1988–1992	4.8	12.8	129.0	8.0	32.0	90.3	2.0	5.7	108.6	6.0	20.8	116.2
1993–1997	4.8	13.3	117.8	9.6	36.3	90.2	1.8	5.4	103.3	4.4	15.8	99.4
1998–2002	3.6	13.4	84.6	18.2	62.2	97.8	2.2	7.3	103.6	5.4	18.4	97.9
2003–2007	5.8	20.4	117.1	19.6	58.9	102.0	2.6	9.7	107.7	7.4	20.2	120.5
2008–2012	4.0	19.0	89.8	15.8	46.1	94.9	3.0	12.0	124.1	6.2	16.6	111.4
	25–34			65–74			25–34			65–74		
1983–1987	12.4	35.3	138.0	4.2	36.8	84.0	3.2	10.1	93.4	5.2	31.7	101.3
1988–1992	4.4	15.6	81.4	4.2	30.4	86.2	1.8	7.0	80.7	4.6	24.0	92.1
1993–1997	6.4	20.6	105.6	6.8	35.1	109.9	2.4	8.1	97.7	3.2	15.1	77.4
1998–2002	10.0	29.8	110.2	9.6	43.7	96.1	5.0	14.2	122.9	5.2	20.5	97.7
2003–2007	10.6	33.0	106.5	10.0	41.9	97.7	4.2	13.5	104.0	5.8	20.8	111.9
2008–2012	10.2	35.3	108.5	8.8	35.2	89.1	5.4	17.8	123.9	4.0	15.5	87.2
	35–44			>74 y/o			35–44			>74 y/o		
1983–1987	13.8	37.2	117.0	3.4	58.0	74.1	4.4	12.3	104.4	6.4	60.3	104.4
1988–1992	7.6	21.0	88.7	4.6	56.7	87.9	3.8	10.2	105.7	8.8	61.4	128.4
1993–1997	6.4	19.8	80.8	5.0	50.5	96.9	1.0	4.9	57.7	5.6	32.5	94.5
1998–2002	10.8	34.9	99.0	5.6	46.0	87.2	3.8	11.7	113.6	5.4	25.1	80.9
2003–2007	17.2	53.6	133.7	5.6	35.5	78.9	4.2	13.4	106.4	5.8	20.9	93.0
2008–2012	15.2	43.5	117.0	8.8	42.8	103.2	5.2	15.2	112.5	7.4	21.8	113.2

Secondary Medical Zone ID: 061

	Male						Female					
	Suicide			Suicide			Suicide			Suicide		
	Num per year	Rate /100,000	×100	Num per year	Rate /100,000	×100	Num per year	Rate /100,000	×100	Num per year	Rate /100,000	×100
	Total (>10 y/o)			45-54			Total (>10 y/o)			45-54		
1983-1987	32.2	28.7	83.3	6.6	35.9	71.0	18.4	14.7	94.6	3.6	18.2	105.8
1988-1992	23.6	20.4	76.2	5.8	27.7	80.5	14.6	11.1	82.5	3.0	14.3	98.1
1993-1997	27.8	22.2	81.8	6.4	30.2	82.5	14.2	9.8	87.5	2.2	11.2	86.4
1998-2002	43.4	32.4	86.1	9.8	47.3	84.4	19.6	12.7	98.9	4.0	16.8	111.6
2003-2007	50.2	37.7	99.8	11.8	60.7	108.5	17.6	12.6	95.5	3.0	14.9	108.4
2008-2012	42.2	33.1	93.5	7.0	43.4	89.4	18.4	12.6	101.3	3.6	18.5	124.1
	15-24			55-64			15-24			55-64		
1983-1987	3.0	14.0	100.7	4.6	36.8	86.5	1.0	5.9	85.7	2.2	16.4	82.9
1988-1992	2.0	9.3	94.2	4.0	27.8	78.2	0.6	4.0	76.6	3.4	19.3	107.9
1993-1997	2.4	11.2	99.3	4.0	26.5	65.8	1.2	5.6	107.9	2.0	12.9	81.2
1998-2002	2.0	13.4	84.5	9.6	49.3	77.6	1.2	7.2	102.5	2.2	14.4	76.7
2003-2007	2.4	16.7	95.7	9.4	46.9	81.2	2.0	11.7	130.4	3.8	17.2	102.6
2008-2012	3.4	23.3	109.8	9.8	48.0	98.9	0.6	7.7	79.0	3.6	16.0	107.5
	25-34			65-74			25-34			65-74		
1983-1987	6.0	26.4	103.1	3.0	38.0	86.7	2.0	10.1	93.8	2.2	23.4	75.0
1988-1992	3.6	17.8	92.9	2.6	30.0	85.3	0.0	4.3	49.3	1.8	17.1	65.6
1993-1997	4.2	19.6	100.4	3.0	28.0	87.7	1.8	9.0	107.8	4.0	24.9	127.8
1998-2002	5.6	26.4	97.9	6.0	44.4	97.5	3.2	14.1	121.9	3.2	20.9	99.4
2003-2007	6.0	31.8	102.7	5.4	35.6	83.1	2.0	12.5	95.8	3.2	18.8	100.9
2008-2012	5.4	34.3	105.3	6.4	36.1	91.3	0.6	10.1	70.7	4.2	20.2	113.6
	35-44			>74 y/o			35-44			>74 y/o		
1983-1987	6.6	27.9	87.7	2.4	68.5	87.5	3.6	13.6	114.8	3.8	64.5	111.9
1988-1992	3.8	18.3	77.0	1.8	42.7	66.2	2.6	10.6	109.4	3.2	40.1	83.8
1993-1997	6.0	27.9	113.7	1.8	34.1	65.5	0.8	6.2	72.4	2.2	22.3	64.8
1998-2002	6.8	36.1	102.5	3.4	45.9	87.1	1.8	10.3	100.1	4.0	28.8	92.7
2003-2007	10.2	51.8	129.4	5.0	49.3	109.5	1.2	9.9	78.7	2.4	15.8	70.5
2008-2012	6.0	33.7	90.5	4.2	37.0	89.2	2.4	13.6	99.9	3.4	18.1	94.2

Secondary Medical Zone ID: 062

	Male						Female					
	Suicide			Suicide			Suicide			Suicide		
	Num per year	Rate /100,000	×100	Num per year	Rate /100,000	×100	Num per year	Rate /100,000	×100	Num per year	Rate /100,000	×100
	Total (>10 y/o)			45–54			Total (>10 y/o)			45–54		
1983–1987	45.0	31.3	95.6	9.0	40.3	79.6	29.0	16.8	114.4	4.8	19.8	115.1
1988–1992	35.4	23.1	90.9	6.8	28.1	81.8	25.0	13.1	106.1	4.6	17.2	117.6
1993–1997	37.8	22.7	88.6	8.8	32.8	89.6	21.0	10.7	99.1	3.8	13.7	106.0
1998–2002	54.0	31.4	84.3	9.8	38.6	68.9	25.0	12.7	98.2	4.8	16.5	109.6
2003–2007	60.2	35.6	91.4	14.8	56.6	101.2	26.2	13.0	105.0	4.0	15.0	109.4
2008–2012	67.8	37.6	108.7	13.0	52.8	108.5	21.2	11.9	89.8	2.8	13.3	89.3
	15–24			55–64			15–24			55–64		
1983–1987	2.4	11.8	84.8	7.2	39.8	93.7	1.0	5.6	82.1	3.4	17.6	89.1
1988–1992	4.0	13.4	135.4	7.2	34.2	96.2	1.0	4.7	88.5	4.4	19.2	107.7
1993–1997	3.0	12.0	106.5	8.0	36.2	89.8	0.4	3.4	65.2	3.8	16.6	104.7
1998–2002	3.2	15.7	99.2	13.2	55.0	86.5	1.6	7.4	105.9	4.6	19.1	101.7
2003–2007	2.8	16.3	93.4	10.8	43.2	74.8	1.4	8.4	92.8	4.8	17.6	104.8
2008–2012	4.8	25.9	121.9	12.0	44.6	91.8	1.6	9.9	102.3	3.4	13.5	90.6
	25–34			65–74			25–34			65–74		
1983–1987	6.4	26.2	102.5	5.8	50.5	115.3	2.4	10.5	97.6	7.4	46.4	148.4
1988–1992	6.2	24.7	129.2	3.2	27.7	78.7	1.4	7.4	85.9	4.2	25.1	96.3
1993–1997	3.4	15.6	79.9	4.4	28.3	88.7	2.2	9.0	108.7	4.0	20.2	103.5
1998–2002	6.8	26.3	97.4	7.2	40.2	88.3	2.6	11.1	95.7	4.4	21.0	100.2
2003–2007	7.4	30.2	97.4	6.8	35.3	82.3	3.8	15.1	116.2	4.4	19.9	106.7
2008–2012	10.6	44.2	135.6	9.6	41.7	105.4	3.0	15.0	104.9	4.0	17.7	99.5
	35–44			>74 y/o			35–44			>74 y/o		
1983–1987	10.2	35.9	112.6	3.8	68.6	87.6	4.2	14.1	119.6	5.8	63.3	109.7
1988–1992	4.8	18.6	78.6	3.2	47.7	74.0	2.4	9.2	95.3	6.6	54.3	113.6
1993–1997	4.6	19.0	77.4	5.4	58.4	112.2	2.4	9.1	107.2	4.4	30.1	87.4
1998–2002	8.0	32.8	93.0	5.6	50.9	96.5	2.6	10.7	103.9	4.4	24.3	78.3
2003–2007	14.2	53.8	134.3	3.4	28.2	62.6	2.4	11.2	88.9	5.4	23.0	102.3
2008–2012	10.4	38.2	102.7	7.0	40.3	97.2	1.6	9.6	70.8	4.8	18.0	93.4

Secondary Medical Zone ID: 063

	Male						Female					
	Suicide			Suicide			Suicide			Suicide		
	Num per year	Rate /100,000	×100	Num per year	Rate /100,000	×100	Num per year	Rate /100,000	×100	Num per year	Rate /100,000	×100
	Total (>10 y/o)			45–54			Total (>10 y/o)			45–54		
1983–1987	33.4	32.5	101.8	6.8	44.7	88.3	15.4	14.8	95.9	3.0	18.9	109.6
1988–1992	27.4	24.5	100.2	7.2	38.6	112.3	14.8	12.4	96.2	2.8	16.1	110.3
1993–1997	36.4	27.7	115.9	8.6	38.8	106.0	14.8	10.8	102.3	2.0	11.8	91.6
1998–2002	56.2	40.3	116.0	13.2	55.7	99.6	17.0	12.7	96.9	5.2	20.2	134.1
2003–2007	61.8	42.5	121.9	13.4	61.3	109.6	18.8	12.5	105.4	4.0	17.4	127.0
2008–2012	53.4	36.7	112.1	9.2	50.8	104.4	17.0	12.7	97.6	1.4	11.7	78.4
	15–24			55–64			15–24			55–64		
1983–1987	4.0	21.5	154.1	5.6	45.8	107.8	1.0	6.8	99.8	2.4	18.6	94.5
1988–1992	2.2	11.4	115.2	2.8	24.5	69.1	2.2	8.8	167.6	2.8	18.5	103.8
1993–1997	4.6	18.2	161.1	7.0	43.7	108.5	1.0	5.5	106.3	2.4	16.0	100.5
1998–2002	3.2	17.9	113.3	11.4	65.1	102.4	1.2	7.4	104.6	2.6	17.5	93.1
2003–2007	3.6	21.1	120.8	14.2	65.3	113.2	1.0	8.3	91.9	3.6	17.7	105.7
2008–2012	2.6	20.4	96.2	11.8	50.0	102.8	1.0	9.3	95.5	3.0	14.4	96.6
	25–34			65–74			25–34			65–74		
1983–1987	5.4	25.8	101.0	2.4	40.1	91.5	2.2	11.3	105.2	2.6	30.1	96.2
1988–1992	3.4	19.2	100.3	2.8	35.3	100.3	0.4	5.7	65.8	2.4	23.3	89.2
1993–1997	4.8	24.7	126.3	4.2	37.3	117.0	0.4	5.7	68.9	3.0	21.7	111.2
1998–2002	7.2	33.7	124.7	7.6	54.6	120.1	2.8	13.9	119.6	2.0	16.7	79.3
2003–2007	7.8	37.4	120.5	7.6	50.2	117.1	1.4	10.7	82.0	3.0	19.3	103.8
2008–2012	7.6	39.1	120.0	8.2	47.1	119.3	2.2	14.5	101.3	3.6	20.1	113.1
	35–44			>74 y/o			35–44			>74 y/o		
1983–1987	8.2	36.5	114.8	1.0	47.6	60.8	1.8	10.7	90.6	2.4	48.6	84.2
1988–1992	6.8	27.3	115.2	2.0	52.8	81.9	2.2	10.1	104.3	2.0	31.7	66.3
1993–1997	5.8	26.2	106.8	1.2	32.7	62.8	1.8	8.9	105.2	4.0	41.1	119.3
1998–2002	11.0	53.2	151.1	2.4	42.0	79.6	1.2	8.7	84.3	2.0	19.4	62.6
2003–2007	10.2	53.3	133.1	5.0	53.2	118.0	1.2	10.2	80.5	4.6	28.6	127.2
2008–2012	7.6	38.3	102.9	6.2	52.0	125.3	2.8	14.8	109.0	2.8	16.6	86.2

Secondary Medical Zone ID: 064

	Male						Female					
	Suicide			Suicide			Suicide			Suicide		
	Num per year	Rate /100,000	×100	Num per year	Rate /100,000	×100	Num per year	Rate /100,000	×100	Num per year	Rate /100,000	×100
	Total (>10 y/o)			45-54			Total (>10 y/o)			45-54		
1983-1987	30.0	30.9	92.5	8.0	49.2	97.4	16.8	15.3	100.6	3.2	19.0	110.3
1988-1992	25.0	23.1	91.6	5.4	30.4	88.4	17.0	13.1	105.2	3.0	15.8	107.9
1993-1997	30.6	24.9	98.5	8.4	38.2	104.5	16.2	11.6	106.9	3.6	15.3	118.5
1998-2002	48.2	36.3	101.9	10.8	51.3	91.6	19.2	13.1	104.3	3.6	16.2	107.7
2003-2007	50.0	38.5	104.1	9.8	53.2	95.1	16.2	12.2	93.7	1.6	11.3	82.8
2008-2012	40.0	33.4	91.5	7.2	45.5	93.6	12.6	11.4	80.5	0.6	9.5	63.6
	15-24			55-64			15-24			55-64		
1983-1987	3.6	19.1	136.6	4.2	38.0	89.3	1.8	8.7	127.5	2.2	18.0	91.2
1988-1992	1.8	9.7	98.1	4.4	31.9	89.8	1.2	5.8	109.8	3.0	19.2	107.5
1993-1997	3.2	14.0	123.5	5.0	33.9	84.2	0.8	4.8	91.3	3.0	17.7	111.2
1998-2002	3.6	18.8	118.8	11.8	65.0	102.3	2.4	10.3	146.5	2.8	17.4	92.4
2003-2007	4.0	22.5	129.2	9.6	49.5	85.8	2.8	14.4	160.1	3.0	15.9	94.7
2008-2012	2.8	21.6	101.7	10.0	48.3	99.5	1.0	9.0	93.1	1.0	10.0	66.9
	25-34			65-74			25-34			65-74		
1983-1987	4.6	25.8	100.9	2.0	35.0	80.0	1.4	9.4	87.8	2.8	30.5	97.6
1988-1992	4.0	22.1	115.8	1.8	27.4	77.9	1.6	9.4	107.9	3.4	29.4	112.8
1993-1997	3.2	18.3	93.6	2.4	26.1	81.7	1.8	9.5	113.8	2.0	17.0	87.3
1998-2002	5.0	25.7	95.3	5.4	43.7	96.2	1.4	9.8	84.3	3.4	22.9	109.1
2003-2007	8.2	38.7	124.8	5.4	39.8	92.7	1.2	9.9	76.2	1.4	13.4	72.2
2008-2012	5.8	34.2	105.0	4.2	30.8	78.0	2.0	14.0	98.0	2.2	15.5	86.9
	35-44			>74 y/o			35-44			>74 y/o		
1983-1987	5.8	28.8	90.4	1.8	58.1	74.2	1.8	10.3	87.2	3.4	61.3	106.3
1988-1992	5.4	24.7	104.3	2.0	49.7	77.0	2.2	10.2	105.2	2.6	37.6	78.5
1993-1997	5.8	28.6	116.8	2.6	47.7	91.7	3.4	13.7	161.3	1.6	20.1	58.3
1998-2002	7.0	39.1	111.1	4.6	61.6	116.8	2.2	11.9	115.3	3.4	28.0	90.2
2003-2007	8.8	47.9	119.5	3.8	42.5	94.3	2.2	13.1	103.6	4.0	25.0	111.4
2008-2012	7.2	37.4	100.5	2.4	27.4	66.0	2.4	13.7	101.0	3.4	18.8	97.7

Secondary Medical Zone ID: 065

	Male						Female					
	Suicide			Suicide			Suicide			Suicide		
	Num per year	Rate /100,000	×100	Num per year	Rate /100,000	×100	Num per year	Rate /100,000	×100	Num per year	Rate /100,000	×100
	Total (>10 y/o)			45-54			Total (>10 y/o)			45-54		
1983-1987	32.6	32.7	100.6	6.4	44.6	88.1	23.4	18.7	136.3	2.6	18.3	106.4
1988-1992	25.0	22.8	90.7	5.2	30.5	88.8	14.4	12.2	94.0	2.4	14.9	102.0
1993-1997	32.8	25.2	102.2	8.0	36.6	100.0	17.8	11.7	116.9	2.2	12.0	93.0
1998-2002	46.2	34.1	93.8	13.4	58.8	105.0	19.2	12.7	103.7	2.4	13.1	87.1
2003-2007	50.2	35.5	95.7	10.2	51.5	92.1	15.8	11.4	88.4	1.2	9.9	72.2
2008-2012	43.6	30.9	86.1	6.0	35.8	73.6	19.8	12.9	100.6	2.6	14.8	99.0
	15-24			55-64			15-24			55-64		
1983-1987	4.0	19.5	139.7	3.8	36.1	85.0	1.8	9.0	132.1	3.6	24.3	123.2
1988-1992	3.4	12.2	123.7	4.4	33.7	94.9	1.2	5.6	105.6	2.0	16.5	92.3
1993-1997	4.0	13.7	121.5	5.2	37.1	92.3	1.4	5.7	109.9	1.8	14.5	90.9
1998-2002	3.2	14.6	92.1	7.8	49.9	78.6	1.6	7.4	105.0	2.6	17.7	94.3
2003-2007	4.4	19.3	110.8	10.8	53.6	92.9	2.4	11.1	123.6	2.8	15.6	93.3
2008-2012	5.2	24.4	114.9	8.0	40.3	82.9	2.2	11.5	118.7	3.2	14.9	99.8
	25-34			65-74			25-34			65-74		
1983-1987	5.6	29.0	113.4	3.8	52.6	120.1	3.6	16.1	149.8	3.6	36.9	118.2
1988-1992	3.8	19.9	104.0	2.4	32.8	93.1	0.8	6.9	79.0	3.0	27.8	106.4
1993-1997	5.2	22.9	117.5	3.6	34.9	109.3	1.8	9.1	109.7	2.8	21.7	111.3
1998-2002	6.2	26.4	97.6	4.8	43.0	94.6	2.2	11.2	96.5	4.4	28.7	136.8
2003-2007	5.0	23.4	75.4	5.8	44.5	103.8	1.0	8.4	64.7	1.8	15.9	85.5
2008-2012	7.4	32.6	100.1	5.8	38.5	97.5	2.0	11.9	83.0	3.0	18.8	105.9
	35-44			>74 y/o			35-44			>74 y/o		
1983-1987	5.6	27.4	86.1	3.4	91.1	116.3	5.2	19.3	163.3	3.0	55.5	96.2
1988-1992	3.8	18.7	79.0	2.0	51.1	79.3	1.8	9.0	93.3	2.8	39.9	83.3
1993-1997	4.4	22.1	90.3	2.4	47.6	91.5	1.8	8.8	103.9	6.0	58.3	169.5
1998-2002	7.2	36.1	102.4	3.6	53.5	101.5	1.4	9.0	87.2	4.4	35.2	113.3
2003-2007	10.2	46.6	116.3	3.6	42.6	94.5	2.4	12.7	100.2	4.2	26.7	118.8
2008-2012	6.4	29.2	78.6	4.6	42.8	103.2	3.2	13.9	102.6	3.6	20.2	104.9

Secondary Medical Zone ID: 066

	Male						Female					
	Suicide			Suicide			Suicide			Suicide		
	Num per year	Rate /100,000	×100	Num per year	Rate /100,000	×100	Num per year	Rate /100,000	×100	Num per year	Rate /100,000	×100
	Total (>10 y/o)			45–54			Total (>10 y/o)			45–54		
1983–1987	38.6	29.1	85.5	8.4	37.8	74.7	20.8	14.4	93.2	4.0	18.2	105.8
1988–1992	35.0	22.0	85.8	9.6	31.0	90.2	20.8	11.9	91.2	3.8	13.9	95.0
1993–1997	45.8	23.2	92.0	12.6	32.9	90.0	22.4	10.3	97.1	4.6	12.5	96.7
1998–2002	69.8	32.5	88.0	16.6	44.5	79.5	28.0	12.1	95.0	4.4	12.5	82.8
2003–2007	75.4	34.0	91.4	15.8	49.8	89.1	32.8	13.0	107.7	3.4	11.5	84.0
2008–2012	79.2	35.1	100.3	15.0	52.2	107.4	28.0	12.4	92.7	3.4	13.1	88.1
	15–24			55–64			15–24			55–64		
1983–1987	6.2	22.8	163.6	4.0	31.4	73.8	2.0	8.0	117.5	3.4	20.1	101.7
1988–1992	2.4	8.5	85.5	5.4	29.1	81.9	1.2	4.5	86.1	3.6	18.0	101.0
1993–1997	6.4	15.6	138.1	9.0	36.4	90.5	2.6	6.7	128.7	4.2	17.1	107.4
1998–2002	5.4	16.4	103.7	16.6	52.8	83.1	2.6	7.8	111.4	4.8	16.9	89.8
2003–2007	5.2	18.3	104.8	19.4	51.2	88.6	2.2	8.7	96.2	8.2	19.9	119.1
2008–2012	3.6	17.0	80.2	16.6	44.0	90.6	2.2	9.8	101.3	4.6	12.6	84.8
	25–34			65–74			25–34			65–74		
1983–1987	3.6	18.8	73.4	3.0	37.6	86.0	2.2	9.9	92.3	2.2	22.2	71.0
1988–1992	4.4	19.2	100.3	4.0	37.2	105.5	1.8	8.3	95.8	2.4	19.1	73.4
1993–1997	5.6	20.1	102.9	3.6	27.0	84.8	0.8	5.3	64.2	4.4	23.6	121.3
1998–2002	8.2	26.0	96.1	8.4	45.5	100.1	3.6	11.8	101.9	4.4	21.6	102.6
2003–2007	12.4	36.6	118.0	7.4	34.1	79.5	3.0	11.0	84.4	5.8	22.7	121.6
2008–2012	13.0	40.9	125.6	9.6	33.7	85.2	4.4	15.4	107.2	6.2	20.0	112.2
	35–44			>74 y/o			35–44			>74 y/o		
1983–1987	10.2	29.6	93.0	2.8	68.1	87.0	3.4	10.6	90.2	3.6	54.1	93.8
1988–1992	6.8	20.2	85.1	2.4	46.4	72.0	3.8	10.2	105.8	4.2	43.1	90.1
1993–1997	5.2	18.1	73.6	3.0	43.8	84.2	1.4	5.8	68.6	4.4	33.4	97.1
1998–2002	9.4	33.1	93.9	5.2	55.4	105.1	2.6	9.6	93.1	5.6	32.8	105.5
2003–2007	9.6	33.4	83.3	5.6	44.8	99.4	4.4	14.5	114.5	5.6	25.5	113.8
2008–2012	13.8	39.1	105.0	7.6	45.9	110.7	4.0	13.0	95.9	3.2	13.9	72.4

Secondary Medical Zone ID: 067

	Male						Female					
	Suicide			Suicide			Suicide			Suicide		
	Num per year	Rate /100,000	×100	Num per year	Rate /100,000	×100	Num per year	Rate /100,000	×100	Num per year	Rate /100,000	×100
	Total (>10 y/o)			45–54			Total (>10 y/o)			45–54		
1983–1987	43.0	36.2	113.8	7.6	44.9	88.8	20.4	15.4	102.3	2.6	16.4	95.1
1988–1992	31.0	24.8	100.8	7.4	37.8	110.0	20.8	13.5	110.1	2.2	13.7	93.9
1993–1997	36.2	26.4	107.2	10.6	44.6	121.9	17.8	11.0	105.6	3.2	14.4	111.5
1998–2002	54.6	38.3	108.9	12.2	52.4	93.7	19.6	12.5	99.6	2.4	12.8	85.3
2003–2007	52.6	38.4	104.6	12.2	57.5	102.8	19.4	12.5	103.0	3.2	15.0	109.3
2008–2012	45.0	35.0	98.7	8.8	50.2	103.3	18.0	12.9	99.6	0.8	9.8	65.8
	15–24			55–64			15–24			55–64		
1983–1987	4.4	21.3	153.1	6.6	43.8	103.1	1.0	6.3	92.9	4.0	21.9	111.1
1988–1992	1.4	8.6	86.6	5.0	31.7	89.2	0.6	4.2	80.4	5.4	25.4	142.5
1993–1997	2.0	10.6	94.1	4.2	29.6	73.5	1.4	6.1	117.2	2.8	16.5	103.6
1998–2002	5.8	25.0	158.4	10.6	60.6	95.4	0.6	5.5	77.7	4.2	21.8	115.7
2003–2007	4.0	22.1	126.9	11.4	55.7	96.5	0.8	7.3	81.4	4.0	18.5	110.7
2008–2012	3.8	25.5	120.4	9.0	42.1	86.7	1.0	9.1	93.5	3.0	14.3	95.7
	25–34			65–74			25–34			65–74		
1983–1987	7.0	31.0	121.1	2.8	36.5	83.3	2.2	10.9	101.5	4.2	34.4	110.0
1988–1992	3.8	20.3	106.0	3.4	34.1	96.8	1.4	8.4	97.2	3.6	25.9	99.2
1993–1997	4.2	22.1	113.2	4.6	34.8	109.1	0.8	6.7	80.6	2.8	18.2	93.2
1998–2002	4.4	24.5	90.7	6.8	47.2	103.7	2.0	11.5	99.1	3.2	20.2	96.1
2003–2007	7.8	38.0	122.6	4.2	33.1	77.2	1.6	11.2	86.3	3.6	21.2	113.6
2008–2012	7.0	38.8	119.1	6.0	39.4	99.8	3.2	17.8	124.1	2.0	15.2	85.7
	35–44			>74 y/o			35–44			>74 y/o		
1983–1987	11.6	47.1	148.0	2.8	66.9	85.4	3.2	13.8	117.2	3.2	46.7	80.9
1988–1992	5.2	22.1	93.4	4.8	79.0	122.5	3.2	12.1	124.8	4.4	45.8	95.6
1993–1997	6.8	29.5	120.4	3.6	53.5	102.7	1.6	8.2	96.4	5.0	40.1	116.4
1998–2002	8.8	44.6	126.6	5.8	64.9	123.1	2.2	11.6	111.9	5.0	32.4	104.3
2003–2007	8.2	46.1	114.9	4.8	44.8	99.4	2.0	12.5	99.3	4.2	22.5	100.5
2008–2012	6.6	36.4	97.9	3.8	33.7	81.1	3.4	16.6	122.7	4.6	21.4	111.2

Secondary Medical Zone ID: 068

	Male						Female					
	Suicide			Suicide			Suicide			Suicide		
	Num per year	Rate /100,000	×100	Num per year	Rate /100,000	×100	Num per year	Rate /100,000	×100	Num per year	Rate /100,000	×100
	Total (>10 y/o)			45–54			Total (>10 y/o)			45–54		
1983–1987	29.0	32.4	101.7	6.2	46.1	91.1	16.2	16.5	111.3	2.6	18.6	108.0
1988–1992	25.4	25.2	104.1	6.8	39.3	114.5	15.0	13.3	108.2	2.6	15.9	109.0
1993–1997	29.8	26.4	107.4	6.6	34.7	95.0	16.4	12.2	122.2	3.4	16.0	123.7
1998–2002	46.6	38.4	110.3	10.8	53.7	95.9	17.2	12.9	107.7	2.0	12.9	85.5
2003–2007	45.6	38.4	106.4	10.6	58.8	105.2	15.2	12.4	99.6	2.0	12.8	93.4
2008–2012	42.8	36.2	107.1	7.8	50.7	104.3	16.8	12.8	107.2	1.6	13.1	87.8
	15–24			55–64			15–24			55–64		
1983–1987	2.4	15.1	108.6	4.6	43.9	103.2	1.0	6.8	99.4	1.6	17.0	86.0
1988–1992	2.0	10.6	106.7	4.6	36.7	103.2	1.0	5.5	105.0	2.4	18.6	104.0
1993–1997	1.6	10.2	90.1	6.2	43.5	108.0	1.2	6.0	114.3	2.8	18.6	117.2
1998–2002	3.6	19.3	122.2	10.6	66.7	105.0	0.8	6.2	88.7	2.8	18.9	100.5
2003–2007	3.6	21.4	122.6	9.2	51.9	90.0	1.8	11.1	123.4	3.0	17.0	101.7
2008–2012	2.4	20.4	96.0	7.6	42.0	86.4	0.8	8.5	87.3	3.2	15.9	106.9
	25–34			65–74			25–34			65–74		
1983–1987	6.8	35.5	139.0	3.4	53.8	122.8	2.8	14.3	132.4	1.6	25.3	81.0
1988–1992	3.4	21.0	110.0	1.6	28.5	80.8	0.6	6.7	76.8	3.0	30.3	116.1
1993–1997	3.2	20.0	102.2	2.8	31.5	98.9	0.4	6.0	71.5	3.8	29.1	149.1
1998–2002	4.2	25.8	95.5	5.4	49.1	108.0	1.4	10.4	90.1	4.0	28.3	134.8
2003–2007	6.6	37.0	119.2	4.4	39.3	91.6	1.8	12.5	96.3	1.2	14.0	75.2
2008–2012	5.2	34.7	106.6	5.2	38.9	98.5	1.4	12.8	89.6	4.2	23.9	134.3
	35–44			>74 y/o			35–44			>74 y/o		
1983–1987	4.4	25.8	81.1	1.2	56.7	72.4	2.2	12.3	103.9	4.2	86.6	150.1
1988–1992	3.6	19.5	82.2	3.4	82.5	127.9	2.0	10.1	104.8	3.0	49.6	103.7
1993–1997	6.0	30.3	123.7	3.0	62.4	119.8	1.0	7.2	84.2	3.8	45.5	132.3
1998–2002	7.6	44.1	125.3	4.4	69.4	131.7	1.8	11.1	107.3	4.4	41.1	132.2
2003–2007	6.8	43.4	108.3	4.4	54.7	121.5	1.6	12.0	95.3	3.8	28.7	128.1
2008–2012	9.4	48.8	131.1	5.2	52.3	126.0	1.6	12.4	91.5	4.0	25.0	130.0

Secondary Medical Zone ID: 069

	Male						Female					
	Suicide			Suicide			Suicide			Suicide		
	Num per year	Rate /100,000	×100	Num per year	Rate /100,000	×100	Num per year	Rate /100,000	×100	Num per year	Rate /100,000	×100
	Total (>10 y/o)			45–54			Total (>10 y/o)			45–54		
1983–1987	54.2	36.7	115.4	11.6	53.2	105.2	34.0	18.7	129.9	4.0	17.9	104.0
1988–1992	45.6	27.1	115.2	8.2	34.9	101.7	36.6	16.7	147.2	3.0	14.2	97.1
1993–1997	53.2	28.5	120.4	9.6	34.6	94.6	28.8	12.9	128.7	4.2	15.0	115.8
1998–2002	76.6	39.3	113.8	19.8	61.0	108.9	36.2	14.9	132.1	6.2	18.8	125.1
2003–2007	85.0	43.0	120.6	19.0	63.0	112.7	29.0	12.8	111.2	5.6	17.8	129.7
2008–2012	85.8	41.6	127.1	16.6	60.9	125.3	34.2	14.8	126.6	5.6	20.2	135.3
	15–24			55–64			15–24			55–64		
1983–1987	6.2	24.9	178.7	9.2	46.8	110.2	2.2	8.9	130.0	4.8	21.0	106.2
1988–1992	3.0	11.4	115.3	8.0	37.1	104.4	1.4	5.8	109.1	5.0	20.3	113.6
1993–1997	4.0	13.8	122.4	11.2	49.2	122.3	1.0	4.7	89.4	4.2	17.9	112.4
1998–2002	6.4	22.2	140.7	14.0	61.8	97.3	1.6	7.0	99.4	4.8	20.3	108.0
2003–2007	5.6	22.3	128.0	15.6	56.4	97.6	1.2	7.2	79.6	4.6	17.2	102.9
2008–2012	5.8	26.8	126.6	19.0	56.8	117.0	0.8	6.8	69.6	3.6	13.2	88.4
	25–34			65–74			25–34			65–74		
1983–1987	7.2	26.3	102.8	6.0	52.5	119.9	4.2	14.4	133.4	7.8	46.8	149.8
1988–1992	5.8	22.5	117.9	6.2	44.1	125.3	3.6	12.8	147.1	8.4	42.5	162.8
1993–1997	4.6	19.1	97.9	8.4	46.4	145.4	3.2	11.7	140.4	7.0	30.0	153.8
1998–2002	8.2	29.6	109.4	8.6	46.0	101.1	3.0	12.2	105.4	6.4	26.6	126.4
2003–2007	14.0	45.6	147.0	8.4	43.4	101.2	2.4	11.3	86.8	3.6	17.3	93.1
2008–2012	9.4	35.3	108.4	12.4	54.5	138.1	5.2	19.8	138.4	4.0	18.3	102.9
	35–44			>74 y/o			35–44			>74 y/o		
1983–1987	8.2	30.7	96.3	5.4	92.0	117.5	3.4	12.9	109.5	7.6	79.1	137.1
1988–1992	9.2	28.1	118.5	4.8	67.4	104.4	5.6	15.3	158.7	9.6	74.4	155.5
1993–1997	9.0	29.2	119.1	6.4	70.0	134.3	2.2	8.3	97.1	6.8	42.8	124.5
1998–2002	10.0	37.7	107.1	9.6	80.6	153.0	3.2	12.1	117.0	10.8	51.8	166.9
2003–2007	13.4	51.3	128.0	9.0	59.9	132.9	2.4	11.5	91.0	9.0	34.7	154.8
2008–2012	14.8	49.4	132.8	7.6	44.2	106.6	4.2	15.7	115.5	10.6	33.8	175.7

Secondary Medical Zone ID: 070

	Male						Female					
	Suicide			Suicide			Suicide			Suicide		
	Num per year	Rate /100,000	×100	Num per year	Rate /100,000	×100	Num per year	Rate /100,000	×100	Num per year	Rate /100,000	×100
	Total (>10 y/o)			45-54			Total (>10 y/o)			45-54		
1983-1987	24.2	30.7	92.6	5.2	43.5	86.0	17.2	16.8	114.8	3.4	21.5	125.0
1988-1992	25.0	26.6	113.2	5.4	38.8	113.0	20.4	15.8	140.6	2.8	17.4	119.3
1993-1997	28.2	27.5	116.5	5.4	36.0	98.3	16.2	12.1	126.3	1.0	10.3	80.1
1998-2002	41.2	39.3	115.2	8.4	53.4	95.5	17.2	13.7	115.1	2.6	16.0	106.4
2003-2007	38.8	39.5	109.0	9.8	65.4	117.0	13.4	12.4	99.4	2.2	14.8	107.7
2008-2012	35.2	35.1	108.2	6.0	48.2	99.2	11.6	12.4	93.3	1.2	12.9	86.4
	15-24			55-64			15-24			55-64		
1983-1987	2.2	16.6	119.1	5.4	46.0	108.2	0.6	6.2	90.4	2.6	19.8	100.4
1988-1992	0.4	7.4	74.3	3.2	28.8	81.1	1.0	6.2	118.5	2.8	19.4	108.4
1993-1997	3.4	17.3	153.2	5.0	40.2	99.8	0.8	5.6	107.8	2.6	18.0	113.1
1998-2002	3.8	23.5	148.5	9.2	69.3	109.0	1.2	8.3	117.7	3.6	22.6	120.1
2003-2007	1.6	17.0	97.6	7.2	51.5	89.3	0.8	8.7	96.6	1.8	15.0	89.4
2008-2012	1.6	20.2	95.2	8.4	51.5	106.0	1.2	11.5	118.3	2.4	15.4	103.0
	25-34			65-74			25-34			65-74		
1983-1987	3.2	24.5	96.0	2.8	43.9	100.2	1.6	11.5	106.9	2.6	30.6	97.9
1988-1992	2.8	21.4	112.0	3.2	39.1	110.9	1.4	10.0	114.8	4.0	34.4	131.8
1993-1997	3.4	23.9	122.4	3.4	34.2	107.2	1.2	9.0	108.1	4.2	29.2	149.9
1998-2002	4.0	29.0	107.3	5.4	48.6	106.8	1.8	12.7	110.0	2.0	18.1	86.0
2003-2007	3.2	28.3	91.1	5.0	45.0	104.9	1.8	14.0	107.6	2.2	18.3	98.1
2008-2012	3.4	33.0	101.3	3.8	36.6	92.7	1.0	12.9	90.0	2.0	17.3	97.0
	35-44			>74 y/o			35-44			>74 y/o		
1983-1987	3.0	24.1	75.7	2.2	70.3	89.7	1.4	11.0	93.4	5.0	87.2	151.1
1988-1992	5.8	32.3	136.1	4.2	88.0	136.4	2.6	12.9	133.6	5.8	76.1	159.0
1993-1997	3.4	23.8	97.1	4.2	74.1	142.2	1.6	9.9	117.0	4.8	51.2	148.9
1998-2002	6.2	43.5	123.6	4.2	62.5	118.5	1.2	10.1	97.7	4.6	39.6	127.4
2003-2007	8.2	58.6	146.2	3.8	46.5	103.2	0.8	10.4	82.0	3.8	26.5	118.1
2008-2012	5.0	38.2	102.6	6.6	60.5	145.9	1.2	12.3	90.8	2.6	17.4	90.3

Secondary Medical Zone ID: 071

	Male						Female					
	Suicide			Suicide			Suicide			Suicide		
	Num per year	Rate /100,000	×100	Num per year	Rate /100,000	×100	Num per year	Rate /100,000	×100	Num per year	Rate /100,000	×100
	Total (>10 y/o)			45-54			Total (>10 y/o)			45-54		
1983-1987	51.8	31.1	94.6	13.8	50.4	99.6	28.8	15.7	104.1	3.8	15.1	87.8
1988-1992	50.4	25.6	106.1	13.8	41.2	120.0	33.0	14.5	120.9	4.2	13.9	95.2
1993-1997	53.2	24.8	99.4	12.8	34.2	93.4	21.8	9.8	88.9	3.2	10.2	78.7
1998-2002	73.2	32.9	89.1	17.2	46.4	83.0	34.0	13.1	107.7	5.8	15.4	102.2
2003-2007	84.0	35.5	96.5	18.2	53.5	95.7	30.8	12.6	98.1	5.4	15.4	112.6
2008-2012	80.2	34.1	97.7	15.0	47.3	97.2	33.0	12.9	102.4	3.0	11.7	78.4
	15-24			55-64			15-24			55-64		
1983-1987	3.8	13.4	96.3	8.0	43.3	101.8	2.4	7.7	112.6	6.2	26.0	131.8
1988-1992	3.0	9.0	90.6	8.4	36.4	102.5	1.8	5.4	101.7	7.2	25.5	142.9
1993-1997	5.2	13.1	116.1	8.8	35.2	87.5	1.2	4.3	81.5	4.8	17.4	109.5
1998-2002	5.4	16.6	105.2	17.2	58.3	91.8	2.4	7.8	110.4	7.0	21.7	115.4
2003-2007	5.2	18.5	105.9	19.2	55.2	95.7	2.6	9.8	109.3	5.4	15.9	94.7
2008-2012	7.2	27.4	129.0	16.2	45.8	94.4	2.6	11.1	114.3	6.2	16.2	108.7
	25-34			65-74			25-34			65-74		
1983-1987	8.4	25.4	99.5	3.2	34.9	79.8	3.0	9.6	89.3	5.4	36.8	117.8
1988-1992	7.8	22.6	118.2	5.6	42.5	120.7	3.0	9.3	106.7	6.2	34.3	131.3
1993-1997	7.6	21.1	107.8	6.8	40.2	126.1	1.6	6.2	74.7	4.0	20.0	102.7
1998-2002	9.4	24.3	89.9	6.4	36.0	79.3	3.2	9.9	85.2	6.2	25.6	121.7
2003-2007	12.2	30.2	97.3	8.0	37.3	87.0	3.0	9.8	75.6	6.4	23.4	125.5
2008-2012	10.6	30.6	93.9	10.6	40.9	103.6	5.0	15.2	106.4	5.0	17.9	100.5
	35-44			>74 y/o			35-44			>74 y/o		
1983-1987	11.4	29.7	93.2	2.8	62.3	79.5	3.6	10.2	86.5	4.2	56.8	98.5
1988-1992	9.2	23.3	98.3	2.4	42.5	65.9	5.2	12.1	124.8	5.4	49.6	103.6
1993-1997	6.6	19.8	80.6	5.2	61.4	117.8	2.6	8.0	93.5	4.4	31.6	91.8
1998-2002	11.6	34.5	97.9	5.6	54.0	102.5	3.2	10.2	98.9	5.8	31.4	101.2
2003-2007	15.2	41.7	104.1	5.8	43.1	95.7	4.6	13.4	106.3	3.4	15.7	70.0
2008-2012	14.0	35.0	94.0	6.4	38.7	93.3	3.8	11.4	84.1	7.4	25.0	130.2

Secondary Medical Zone ID: 072

	Male						Female					
	Suicide			Suicide			Suicide			Suicide		
	Num per year	Rate /100,000	×100	Num per year	Rate /100,000	×100	Num per year	Rate /100,000	×100	Num per year	Rate /100,000	×100
	Total (>10 y/o)			45–54			Total (>10 y/o)			45–54		
1983–1987	23.2	35.3	115.6	4.4	50.3	99.4	13.8	17.3	122.9	2.8	23.2	134.7
1988–1992	20.8	27.6	123.6	4.2	40.8	118.8	14.6	15.1	137.0	1.4	15.4	105.0
1993–1997	19.4	26.1	108.5	4.0	35.6	97.2	11.4	11.8	122.3	1.2	12.7	98.2
1998–2002	24.8	34.0	95.2	5.8	47.9	85.6	10.2	12.0	99.7	1.6	14.6	96.8
2003–2007	25.0	34.9	93.2	6.4	54.2	96.9	11.8	13.0	112.6	1.0	12.0	87.4
2008–2012	28.6	36.4	111.2	6.6	59.5	122.5	12.8	13.4	118.2	1.6	15.8	106.2
	15–24			55–64			15–24			55–64		
1983–1987	2.2	19.3	138.7	3.6	44.3	104.3	0.2	5.6	82.3	1.8	19.7	99.8
1988–1992	1.4	11.4	115.0	4.6	45.6	128.4	1.0	7.0	132.7	2.2	20.4	114.3
1993–1997	1.2	11.5	101.5	3.8	42.7	106.1	0.2	4.3	81.5	1.2	15.2	95.8
1998–2002	1.6	16.3	103.1	5.0	59.4	93.6	0.2	5.6	80.2	1.4	18.3	97.1
2003–2007	2.0	19.9	114.0	5.0	49.6	86.0	0.6	8.6	95.7	1.2	15.3	91.2
2008–2012	2.4	26.2	123.7	5.0	43.2	88.9	0.6	9.6	98.9	2.8	18.1	121.3
	25–34			65–74			25–34			65–74		
1983–1987	3.2	27.3	106.6	1.8	41.2	94.0	0.6	8.7	80.8	3.2	42.1	134.6
1988–1992	2.0	19.8	103.8	2.4	40.0	113.4	1.2	10.3	118.3	2.8	32.9	126.1
1993–1997	2.0	20.8	106.3	3.0	39.1	122.6	0.4	7.3	87.5	3.6	31.9	163.8
1998–2002	2.8	28.0	103.8	3.6	47.0	103.3	0.6	10.1	87.5	1.6	19.8	94.3
2003–2007	2.4	27.3	88.1	2.6	38.6	90.0	2.0	16.2	124.7	2.4	23.2	124.6
2008–2012	2.0	27.7	85.1	4.2	47.3	119.7	0.6	12.1	84.2	1.4	18.0	101.3
	35–44			>74 y/o			35–44			>74 y/o		
1983–1987	5.8	43.3	136.1	2.0	77.7	99.2	1.4	12.7	107.8	3.8	82.9	143.7
1988–1992	3.6	26.0	109.7	2.4	70.5	109.3	1.0	9.3	96.5	5.0	82.6	172.6
1993–1997	3.6	27.8	113.6	1.4	43.2	83.0	0.8	8.0	94.3	4.0	54.3	157.6
1998–2002	2.2	27.0	76.6	3.8	71.3	135.3	0.6	8.9	86.2	4.2	45.7	147.1
2003–2007	3.2	36.5	91.2	3.4	53.5	118.8	0.8	11.6	91.9	3.8	33.6	149.8
2008–2012	4.2	39.7	106.7	4.2	53.5	128.9	0.8	12.2	89.9	5.0	35.1	182.6

Secondary Medical Zone ID: 073

	Male						Female					
	Suicide			Suicide			Suicide			Suicide		
	Num per year	Rate /100,000	×100	Num per year	Rate /100,000	×100	Num per year	Rate /100,000	×100	Num per year	Rate /100,000	×100
	Total (>10 y/o)			45–54			Total (>10 y/o)			45–54		
1983–1987	57.0	33.0	102.1	13.8	51.1	101.1	31.6	16.4	110.0	5.4	19.8	114.7
1988–1992	47.4	24.7	100.9	11.6	36.6	106.7	29.0	13.3	106.3	6.6	19.6	134.1
1993–1997	53.0	25.1	99.9	13.4	36.0	98.3	27.0	11.1	105.6	3.6	11.3	87.7
1998–2002	79.0	34.8	96.8	15.6	41.7	74.4	28.8	12.1	93.1	4.6	13.3	88.7
2003–2007	76.2	32.9	90.0	16.4	46.8	83.7	30.4	12.2	98.2	4.4	13.2	96.5
2008–2012	77.4	33.5	97.4	12.6	42.3	87.0	30.4	12.4	97.7	2.2	10.0	67.0
	15–24			55–64			15–24			55–64		
1983–1987	4.6	16.1	115.2	6.2	33.1	77.8	1.4	5.7	84.1	5.2	21.3	108.1
1988–1992	4.8	12.8	129.0	7.2	31.0	87.2	2.8	7.2	137.3	3.8	16.1	90.2
1993–1997	3.6	10.8	95.8	8.8	34.8	86.5	2.6	6.7	129.0	3.6	14.5	91.2
1998–2002	6.6	19.6	124.2	18.8	62.6	98.6	1.6	6.0	85.6	4.4	16.6	88.1
2003–2007	4.4	16.5	94.4	21.0	58.7	101.7	2.8	10.0	110.9	6.8	18.8	112.2
2008–2012	5.0	20.8	98.3	18.0	47.0	96.8	2.2	9.6	98.5	6.0	15.7	105.6
	25–34			65–74			25–34			65–74		
1983–1987	10.2	30.0	117.3	6.8	55.2	126.0	3.4	10.8	100.2	4.2	28.6	91.6
1988–1992	3.8	14.3	74.7	6.2	42.3	120.2	1.0	5.5	63.4	3.8	21.6	82.6
1993–1997	7.8	24.4	124.8	6.8	36.6	114.8	2.6	8.7	104.4	4.0	18.3	94.0
1998–2002	10.2	29.8	110.3	10.0	47.0	103.3	4.6	13.4	115.7	5.2	21.5	102.2
2003–2007	10.0	30.2	97.4	7.8	35.6	83.1	3.8	12.4	95.6	4.0	16.9	90.8
2008–2012	9.4	32.4	99.4	10.6	40.2	101.8	3.4	13.1	91.5	4.4	16.9	94.7
	35–44			>74 y/o			35–44			>74 y/o		
1983–1987	12.6	34.1	107.2	2.8	55.4	70.8	5.4	14.5	122.7	6.6	71.1	123.2
1988–1992	9.0	23.2	97.9	4.8	65.0	100.7	4.4	11.2	115.9	6.4	52.4	109.5
1993–1997	8.0	22.8	93.0	4.2	48.4	93.0	2.8	8.3	98.1	7.8	47.8	138.9
1998–2002	10.4	33.3	94.5	7.4	62.7	118.9	3.8	11.7	113.1	4.6	23.5	75.8
2003–2007	9.0	30.3	75.5	7.6	49.6	110.2	2.6	10.2	80.6	6.0	23.1	103.0
2008–2012	13.4	38.2	102.5	8.2	44.2	106.6	5.4	15.5	114.2	6.6	21.4	111.3

Secondary Medical Zone ID: 074

	Male						Female					
	Suicide			Suicide			Suicide			Suicide		
	Num per year	Rate /100,000	×100	Num per year	Rate /100,000	×100	Num per year	Rate /100,000	×100	Num per year	Rate /100,000	×100
	Total (>10 y/o)			45–54			Total (>10 y/o)			45–54		
1983–1987	40.4	33.5	103.1	9.6	50.4	99.7	30.6	19.3	138.5	6.2	25.6	148.8
1988–1992	38.0	27.5	117.2	6.2	31.1	90.5	22.4	13.8	113.4	4.6	18.6	127.5
1993–1997	38.6	27.3	111.2	10.4	42.0	114.7	20.6	11.8	116.1	2.6	12.1	93.8
1998–2002	52.4	36.4	103.5	13.0	56.1	100.2	23.4	13.1	112.1	3.0	14.1	93.7
2003–2007	51.4	36.9	102.2	12.0	60.6	108.4	21.4	13.1	108.6	2.2	12.6	91.9
2008–2012	49.0	35.7	106.6	10.0	55.8	114.8	22.0	14.0	112.7	3.8	19.0	127.5
	15–24			55–64			15–24			55–64		
1983–1987	3.8	17.7	127.0	4.6	33.7	79.4	1.4	7.1	104.4	2.8	17.3	87.8
1988–1992	2.0	9.8	98.8	9.0	47.4	133.6	0.8	4.7	89.0	2.4	14.9	83.6
1993–1997	2.4	11.6	103.0	8.8	46.4	115.3	0.8	4.8	92.3	4.2	19.7	123.7
1998–2002	1.2	11.2	70.6	10.4	56.0	88.2	1.2	7.3	103.7	4.4	20.6	109.7
2003–2007	2.8	18.3	105.2	13.2	59.3	102.7	1.2	9.0	100.1	4.2	18.0	107.2
2008–2012	2.8	22.2	104.8	12.8	54.7	112.6	0.4	6.9	71.1	2.4	12.8	85.7
	25–34			65–74			25–34			65–74		
1983–1987	4.2	22.9	89.5	4.0	43.8	100.0	2.8	12.8	119.3	6.2	44.9	143.6
1988–1992	3.8	20.6	108.0	5.6	47.4	134.6	1.2	7.8	90.0	4.2	28.3	108.5
1993–1997	4.8	24.2	124.0	2.6	23.5	73.8	1.6	8.8	105.2	5.0	27.4	140.4
1998–2002	6.0	30.1	111.3	8.8	55.5	122.1	1.0	8.8	75.6	4.6	24.6	117.3
2003–2007	4.8	28.1	90.7	5.4	37.4	87.2	2.4	13.7	105.0	2.4	15.6	83.6
2008–2012	5.6	35.1	107.6	6.8	40.5	102.6	2.6	16.2	113.2	3.6	18.8	105.5
	35–44			>74 y/o			35–44			>74 y/o		
1983–1987	8.6	34.7	108.9	5.2	102.5	130.9	4.0	14.8	125.3	7.2	93.7	162.4
1988–1992	6.6	26.7	112.6	4.6	73.6	114.0	3.2	11.9	122.8	6.0	61.7	129.0
1993–1997	5.6	26.9	109.6	4.0	56.4	108.3	1.8	8.9	104.5	4.4	36.9	107.4
1998–2002	7.4	39.7	112.7	5.6	63.1	119.7	2.0	11.0	106.7	7.2	45.2	145.7
2003–2007	7.4	41.3	103.1	5.8	52.6	116.9	2.8	14.5	115.1	6.2	31.9	142.3
2008–2012	5.2	31.1	83.6	5.8	45.1	108.7	4.0	17.8	131.5	5.2	23.2	120.8

Secondary Medical Zone ID: 075

	Male						Female					
	Suicide			Suicide			Suicide			Suicide		
	Num per year	Rate /100,000	×100	Num per year	Rate /100,000	×100	Num per year	Rate /100,000	×100	Num per year	Rate /100,000	×100
	Total (>10 y/o)			45–54			Total (>10 y/o)			45–54		
1983–1987	47.6	35.2	111.5	9.8	47.7	94.4	27.0	17.1	116.9	5.2	21.5	124.8
1988–1992	33.6	23.9	96.0	9.8	40.7	118.6	23.4	13.4	108.6	3.4	14.8	100.9
1993–1997	40.0	25.6	103.4	8.4	33.4	91.4	25.0	12.5	124.4	4.4	15.3	118.8
1998–2002	49.6	32.4	86.9	11.8	47.4	84.7	26.8	13.2	112.0	4.6	16.7	110.9
2003–2007	60.0	37.6	101.6	11.8	52.2	93.3	24.6	12.8	106.6	4.0	15.9	116.1
2008–2012	56.8	35.2	103.2	9.2	45.6	93.9	24.8	14.0	107.2	3.0	14.7	98.6
	15–24			55–64			15–24			55–64		
1983–1987	4.6	19.5	139.6	5.8	38.7	91.0	1.2	6.2	90.5	4.6	23.4	118.7
1988–1992	2.6	10.6	107.5	5.0	28.9	81.3	0.8	4.3	80.8	3.0	16.1	90.3
1993–1997	3.8	14.2	125.7	7.8	39.5	98.0	1.4	5.7	108.4	4.6	19.8	124.3
1998–2002	3.0	15.3	96.7	12.8	60.2	94.7	1.2	6.5	93.2	4.0	18.3	97.4
2003–2007	3.4	18.4	105.3	16.4	65.5	113.4	2.2	11.0	122.2	4.2	16.8	100.3
2008–2012	4.8	26.2	123.3	11.0	45.3	93.2	2.4	12.7	130.9	4.2	15.8	105.9
	25–34			65–74			25–34			65–74		
1983–1987	6.6	27.9	109.0	4.4	47.7	108.9	2.2	10.1	93.8	2.6	24.9	79.8
1988–1992	4.2	19.1	100.0	3.6	34.4	97.7	2.8	10.9	125.9	3.6	25.6	98.2
1993–1997	4.8	20.7	105.9	3.2	25.8	80.8	2.0	8.7	104.2	3.2	19.1	98.2
1998–2002	6.6	27.4	101.5	6.4	40.9	89.9	2.2	10.4	89.7	5.4	26.0	123.9
2003–2007	8.6	35.9	115.7	6.2	37.3	86.9	2.0	10.8	82.7	4.6	21.5	115.5
2008–2012	5.4	29.7	91.2	10.0	48.7	123.4	2.8	14.7	102.7	4.6	20.2	113.7
	35–44			>74 y/o			35–44			>74 y/o		
1983–1987	10.4	37.3	117.2	5.4	100.7	128.6	4.8	15.5	131.2	6.4	78.5	136.1
1988–1992	5.2	20.3	85.8	3.2	55.3	85.8	2.8	10.2	105.4	7.0	65.5	136.8
1993–1997	5.8	24.4	99.6	6.2	76.7	147.3	2.8	10.5	124.0	6.6	49.3	143.1
1998–2002	6.0	29.4	83.4	3.0	37.8	71.7	2.4	10.8	104.8	7.0	41.7	134.2
2003–2007	10.4	45.3	113.0	3.2	30.8	68.4	2.2	11.2	88.8	5.4	26.1	116.4
2008–2012	11.0	42.8	114.9	5.4	37.0	89.3	4.0	15.4	113.9	3.8	16.5	85.7

Secondary Medical Zone ID: 076

	Male						Female					
	Suicide			Suicide			Suicide			Suicide		
	Num per year	Rate /100,000	×100	Num per year	Rate /100,000	×100	Num per year	Rate /100,000	×100	Num per year	Rate /100,000	×100
	Total (>10 y/o)			45-54			Total (>10 y/o)			45-54		
1983-1987	53.4	32.8	101.2	12.6	49.5	97.9	33.2	17.2	117.0	5.8	20.6	119.6
1988-1992	35.4	21.3	82.2	8.2	29.5	85.9	27.0	12.7	102.4	4.0	14.3	97.9
1993-1997	41.2	22.2	86.5	9.0	29.3	80.0	24.8	10.9	102.6	3.8	12.2	94.3
1998-2002	71.8	35.3	98.7	17.2	53.2	95.0	29.8	12.9	102.0	4.2	13.8	91.6
2003-2007	74.0	36.3	99.2	13.6	49.3	88.2	28.2	12.6	99.1	3.4	12.8	93.5
2008-2012	75.2	36.7	107.2	13.2	50.7	104.3	27.4	12.7	96.7	2.6	12.1	81.1
	15-24			55-64			15-24			55-64		
1983-1987	2.4	10.5	75.5	8.6	43.3	102.0	2.2	7.8	113.9	4.6	20.4	103.2
1988-1992	3.2	10.5	106.0	4.8	23.9	67.4	1.4	5.0	95.2	4.8	19.1	106.7
1993-1997	3.0	10.8	95.2	11.8	46.1	114.5	2.2	6.6	127.4	4.6	17.3	109.0
1998-2002	5.0	18.5	117.2	16.2	59.7	94.0	1.8	7.3	103.3	4.4	16.9	89.8
2003-2007	4.6	19.4	111.4	18.2	57.9	100.4	2.2	9.8	109.3	4.4	14.9	89.1
2008-2012	5.8	25.7	121.3	16.0	50.1	103.2	2.4	11.2	115.8	5.2	15.6	104.5
	25-34			65-74			25-34			65-74		
1983-1987	6.6	24.5	95.7	5.4	46.3	105.7	4.2	13.8	127.8	4.8	31.3	100.2
1988-1992	5.0	19.2	100.3	3.8	30.0	85.0	1.2	6.4	73.6	5.6	29.9	114.5
1993-1997	3.8	15.3	78.5	4.6	27.8	87.0	2.4	8.7	104.8	4.4	20.6	105.6
1998-2002	8.8	28.6	105.8	9.0	44.6	98.1	4.4	13.7	117.9	5.2	22.0	104.9
2003-2007	7.6	27.2	87.7	9.4	42.6	99.4	5.2	16.3	125.2	3.8	16.5	88.7
2008-2012	6.8	29.2	89.6	9.2	37.9	96.1	3.2	13.7	95.9	5.2	18.9	106.1
	35-44			>74 y/o			35-44			>74 y/o		
1983-1987	10.8	31.9	100.1	6.8	101.3	129.4	5.2	14.3	121.0	6.4	66.0	114.5
1988-1992	4.2	14.9	62.7	5.8	71.1	110.2	3.0	9.4	97.2	6.8	52.2	109.0
1993-1997	5.4	20.0	81.5	3.4	39.0	75.0	1.0	5.5	64.4	6.2	37.5	109.0
1998-2002	8.6	32.9	93.3	6.8	56.2	106.5	3.2	11.6	112.3	6.6	32.1	103.5
2003-2007	13.2	45.8	114.3	7.4	46.9	104.2	2.8	11.3	89.3	6.4	24.7	110.3
2008-2012	15.6	46.4	124.8	8.6	44.5	107.2	3.6	12.6	92.8	5.2	17.6	91.6

Secondary Medical Zone ID: 077

	Male						Female					
	Suicide			Suicide			Suicide			Suicide		
	Num per year	Rate /100,000	×100	Num per year	Rate /100,000	×100	Num per year	Rate /100,000	×100	Num per year	Rate /100,000	×100
	Total (>10 y/o)			45-54			Total (>10 y/o)			45-54		
1983-1987	18.2	35.6	116.9	4.2	54.7	108.1	11.2	17.3	125.8	1.4	17.9	104.0
1988-1992	14.2	25.9	110.5	3.0	36.6	106.4	10.8	14.7	128.4	1.2	14.9	102.0
1993-1997	14.6	25.2	104.6	3.6	38.0	103.8	9.0	11.7	119.8	1.4	13.9	107.8
1998-2002	17.8	33.7	88.7	5.6	56.9	101.7	8.6	12.9	102.5	1.4	15.1	100.5
2003-2007	22.6	37.3	105.5	4.6	54.1	96.8	8.6	12.7	104.0	1.4	14.7	107.3
2008-2012	19.0	34.0	97.7	3.4	47.2	97.2	7.8	12.3	99.3	1.2	15.5	104.0
	15-24			55-64			15-24			55-64		
1983-1987	1.8	19.1	136.6	1.8	36.2	85.2	0.4	6.6	96.0	2.4	25.5	129.1
1988-1992	1.8	13.6	137.1	2.0	31.8	89.6	1.0	7.3	138.6	1.4	18.3	102.5
1993-1997	1.4	13.2	116.3	2.2	35.4	88.0	0.4	5.2	100.0	1.0	15.0	94.4
1998-2002	1.0	15.3	96.7	3.8	55.5	87.4	0.0	5.3	75.8	1.4	18.5	98.5
2003-2007	1.4	19.0	108.8	4.8	55.5	96.1	0.2	7.3	81.6	0.8	14.4	86.0
2008-2012	0.4	17.1	80.7	3.2	40.8	84.1	0.0	7.0	72.3	1.4	14.9	100.1
	25-34			65-74			25-34			65-74		
1983-1987	2.8	30.7	120.0	2.8	60.8	138.9	1.2	12.5	116.1	1.2	28.9	92.5
1988-1992	2.0	23.4	122.6	1.4	34.6	98.1	0.8	9.5	109.8	1.8	29.4	112.8
1993-1997	1.8	22.1	113.0	3.0	44.2	138.5	0.6	8.4	101.2	1.6	22.0	112.7
1998-2002	1.0	21.7	80.5	1.6	36.1	79.4	1.4	13.7	117.8	1.0	18.2	86.6
2003-2007	3.6	38.3	123.5	2.6	41.8	97.5	0.8	12.5	95.9	1.6	20.1	107.9
2008-2012	2.6	34.7	106.5	2.6	39.0	98.7	0.4	12.3	85.5	1.4	18.2	102.3
	35-44			>74 y/o			35-44			>74 y/o		
1983-1987	3.0	32.9	103.5	1.6	80.4	102.6	1.6	14.1	119.0	3.0	87.8	152.2
1988-1992	1.8	21.2	89.3	2.2	79.4	123.1	1.2	10.8	111.5	3.4	74.9	156.6
1993-1997	1.0	17.5	71.5	1.6	54.6	104.9	0.8	8.9	105.1	3.2	56.1	162.9
1998-2002	2.6	34.6	98.3	2.2	57.7	109.5	1.2	12.2	117.9	2.2	34.4	110.7
2003-2007	3.0	40.6	101.2	2.6	50.6	112.4	1.2	13.8	109.2	2.6	29.8	133.0
2008-2012	4.0	43.1	115.8	2.8	45.8	110.4	1.0	13.5	99.8	2.4	23.5	122.1

Secondary Medical Zone ID: 078

	Male						Female					
	Suicide			Suicide			Suicide			Suicide		
	Num per year	Rate /100,000	×100	Num per year	Rate /100,000	×100	Num per year	Rate /100,000	×100	Num per year	Rate /100,000	×100
	Total (>10 y/o)			45–54			Total (>10 y/o)			45–54		
1983–1987	12.4	35.7	118.8	3.2	58.5	115.7	9.6	18.3	144.6	1.2	19.3	112.1
1988–1992	10.6	27.0	120.8	1.8	34.4	100.2	9.0	15.5	145.5	1.0	15.7	107.6
1993–1997	8.8	24.6	99.6	1.8	34.1	93.2	7.4	12.3	135.2	1.0	14.0	108.4
1998–2002	14.6	36.4	107.0	3.0	52.6	94.0	7.4	13.4	119.9	0.4	13.2	87.5
2003–2007	15.0	36.6	111.4	2.4	51.1	91.4	5.6	12.6	105.4	0.4	12.5	90.9
2008–2012	14.6	36.3	114.4	2.6	52.6	108.2	3.4	12.0	88.4	0.2	12.8	85.5
	15–24			55–64			15–24			55–64		
1983–1987	0.8	15.2	109.1	2.0	46.4	109.2	0.4	7.2	105.0	1.8	25.8	130.5
1988–1992	0.4	9.5	96.1	1.0	30.2	85.1	0.2	5.0	94.9	0.8	17.7	99.3
1993–1997	1.4	14.6	129.2	1.2	34.6	86.0	0.2	5.0	95.7	1.6	20.9	131.6
1998–2002	0.8	16.4	103.6	3.0	61.6	96.9	0.6	8.2	116.5	1.4	21.0	111.3
2003–2007	1.0	19.5	111.6	3.8	62.9	108.9	0.4	9.4	104.6	0.6	15.4	92.1
2008–2012	0.4	19.5	91.9	3.2	51.2	105.4	0.4	10.3	105.8	0.2	12.4	83.4
	25–34			65–74			25–34			65–74		
1983–1987	0.6	20.9	81.8	1.6	52.6	120.1	1.2	14.3	132.5	0.8	29.2	93.4
1988–1992	1.4	23.4	122.5	1.4	41.1	116.7	0.6	9.7	111.5	2.0	37.9	145.5
1993–1997	0.2	14.9	76.4	1.6	38.2	119.8	0.2	7.6	91.8	2.2	32.0	164.3
1998–2002	1.6	28.5	105.6	2.4	51.6	113.4	0.4	11.0	95.1	2.2	30.4	144.9
2003–2007	2.0	34.8	112.2	2.2	47.3	110.3	0.4	12.3	94.3	1.4	22.2	119.1
2008–2012	1.8	36.1	110.9	2.2	43.5	110.0	0.0	11.9	83.0	0.6	16.7	94.0
	35–44			>74 y/o			35–44			>74 y/o		
1983–1987	2.4	35.9	112.9	1.8	101.3	129.3	2.2	18.2	153.8	2.0	80.7	140.0
1988–1992	2.6	31.7	133.8	2.0	88.6	137.4	1.0	11.2	116.4	3.4	93.9	196.3
1993–1997	1.2	23.8	97.3	1.4	61.8	118.6	0.0	6.6	77.4	2.2	53.2	154.6
1998–2002	1.6	34.8	98.9	2.2	70.8	134.4	0.2	9.1	88.3	2.2	44.0	141.6
2003–2007	0.8	29.7	74.2	2.8	66.7	148.0	0.4	11.9	94.5	2.0	32.4	144.3
2008–2012	2.4	40.7	109.4	1.8	45.5	109.6	0.4	12.6	93.2	1.6	23.4	121.7

Secondary Medical Zone ID: 079

	Male						Female					
	Suicide			Suicide			Suicide			Suicide		
	Num per year	Rate /100,000	×100	Num per year	Rate /100,000	×100	Num per year	Rate /100,000	×100	Num per year	Rate /100,000	×100
	Total (>10 y/o)			45-54			Total (>10 y/o)			45-54		
1983-1987	15.4	36.2	121.5	4.0	61.7	122.0	10.6	18.2	135.3	0.8	16.3	94.7
1988-1992	11.8	26.2	115.5	2.2	36.2	105.5	9.4	14.4	133.0	0.8	14.6	100.0
1993-1997	14.0	28.0	126.6	2.4	37.1	101.5	7.8	12.1	126.6	1.2	14.7	113.5
1998-2002	21.4	41.2	133.0	6.0	73.7	131.8	9.2	13.7	126.2	1.6	17.7	117.5
2003-2007	18.4	39.5	120.2	3.8	61.4	109.7	8.0	13.2	119.5	0.6	13.1	95.4
2008-2012	14.8	35.2	108.8	2.6	50.7	104.3	7.2	13.1	113.9	0.8	15.4	103.0
	15-24			55-64			15-24			55-64		
1983-1987	0.6	13.4	95.8	2.6	45.3	106.5	0.4	7.0	102.5	1.0	18.6	94.4
1988-1992	0.4	9.4	95.3	2.0	34.9	98.2	0.4	5.7	108.1	1.8	21.6	120.8
1993-1997	1.0	13.0	114.9	3.4	49.8	123.8	0.2	4.9	94.8	0.6	14.2	89.6
1998-2002	0.4	14.3	90.6	3.2	61.1	96.2	0.4	7.3	104.5	2.0	23.2	123.2
2003-2007	0.6	17.2	98.5	4.2	63.3	109.7	0.0	7.2	79.8	1.4	18.4	110.0
2008-2012	0.8	22.1	104.0	2.8	46.4	95.5	0.0	7.9	81.4	0.8	14.5	97.1
	25-34			65-74			25-34			65-74		
1983-1987	1.8	28.1	109.9	2.2	56.1	128.1	1.6	15.7	145.7	2.4	43.0	137.7
1988-1992	1.4	22.7	118.8	2.4	48.2	136.8	0.4	8.6	99.3	2.2	35.3	135.3
1993-1997	1.4	23.1	118.2	1.2	29.0	91.0	0.4	8.4	100.9	2.2	28.3	145.1
1998-2002	1.8	30.2	111.9	4.4	63.6	139.8	0.6	11.8	102.2	1.4	22.4	106.6
2003-2007	1.2	29.5	95.3	2.2	44.1	102.8	0.8	14.1	108.4	1.4	21.0	112.6
2008-2012	1.6	35.1	107.8	1.8	38.7	97.9	0.4	13.9	96.9	1.6	21.2	119.0
	35-44			>74 y/o			35-44			>74 y/o		
1983-1987	2.0	31.7	99.6	2.2	99.6	127.2	1.6	15.4	130.8	2.8	86.4	149.7
1988-1992	1.6	23.7	100.0	1.8	74.4	115.4	0.4	9.0	92.7	3.2	76.2	159.3
1993-1997	2.0	28.8	117.5	2.6	78.7	151.1	1.0	10.8	127.0	2.0	42.3	122.9
1998-2002	3.0	46.0	130.6	2.6	67.7	128.3	0.2	8.9	86.5	3.0	46.2	148.8
2003-2007	2.8	50.4	125.7	3.6	66.3	147.2	0.6	12.8	101.0	3.2	38.1	169.6
2008-2012	1.6	36.5	98.2	3.6	57.9	139.7	0.6	13.5	99.8	3.0	30.1	156.7

Secondary Medical Zone ID: 080

	Male						Female					
	Suicide			Suicide			Suicide			Suicide		
	Num per year	Rate /100,000	×100	Num per year	Rate /100,000	×100	Num per year	Rate /100,000	×100	Num per year	Rate /100,000	×100
	Total (>10 y/o)			45-54			Total (>10 y/o)			45-54		
1983-1987	17.0	40.4	146.4	3.8	63.5	125.6	7.6	16.6	118.3	0.6	15.9	92.5
1988-1992	9.6	25.5	111.5	1.8	35.9	104.4	8.4	14.5	134.6	0.6	14.3	97.8
1993-1997	12.4	28.1	130.3	1.8	36.5	99.7	9.0	13.1	153.6	0.2	11.2	86.6
1998-2002	13.4	36.2	107.0	3.2	58.5	104.5	7.8	14.0	125.9	0.6	14.5	96.4
2003-2007	16.4	39.6	127.7	2.0	48.5	86.6	5.2	12.6	105.1	0.8	14.6	106.6
2008-2012	12.0	34.2	108.3	1.4	43.8	90.0	5.0	13.2	105.6	0.2	13.2	88.4
	15-24			55-64			15-24			55-64		
1983-1987	1.2	18.5	132.6	2.6	48.7	114.7	0.8	9.0	131.3	1.4	21.8	110.2
1988-1992	0.4	9.9	100.3	2.0	37.3	105.1	0.2	5.3	100.1	2.0	23.5	131.4
1993-1997	0.2	10.3	91.3	1.4	35.8	89.0	0.2	5.3	100.9	1.8	21.9	137.5
1998-2002	1.2	19.5	123.3	1.8	52.5	82.7	0.4	7.7	109.7	2.0	24.1	128.1
2003-2007	1.2	21.4	122.6	2.4	53.8	93.2	0.4	9.8	109.0	0.4	15.1	90.1
2008-2012	0.2	19.3	90.9	2.6	49.1	101.1	0.2	9.5	98.4	0.8	15.1	101.3
	25-34			65-74			25-34			65-74		
1983-1987	1.2	25.5	99.6	1.8	51.5	117.7	0.2	9.3	86.9	1.2	31.5	100.9
1988-1992	0.8	19.3	100.9	2.0	45.7	129.6	0.8	10.8	124.6	1.2	26.7	102.2
1993-1997	1.2	23.0	117.6	2.8	48.8	153.1	0.6	9.6	115.8	2.2	29.9	153.5
1998-2002	1.4	29.7	109.8	3.0	55.0	120.9	0.6	12.5	107.6	2.2	29.3	139.5
2003-2007	2.6	42.9	138.3	3.2	56.1	130.9	0.4	13.0	100.0	1.4	22.0	118.0
2008-2012	1.0	33.4	102.6	3.0	50.7	128.3	0.8	16.6	116.0	0.8	17.9	100.8
	35-44			>74 y/o			35-44			>74 y/o		
1983-1987	3.0	43.2	135.7	3.4	139.0	177.5	1.2	14.5	123.0	2.2	79.8	138.4
1988-1992	0.6	19.1	80.4	2.0	81.9	126.9	1.2	12.4	128.3	2.4	66.2	138.3
1993-1997	2.0	31.4	128.0	2.6	81.8	157.1	0.6	9.6	112.5	3.4	67.7	196.8
1998-2002	1.2	33.0	93.6	1.6	54.9	104.1	0.6	11.4	109.9	1.4	30.0	96.8
2003-2007	2.0	46.0	114.8	3.0	63.9	142.0	0.0	10.6	84.0	1.8	27.5	122.7
2008-2012	0.6	32.5	87.2	3.0	55.8	134.4	0.6	14.2	104.7	1.6	21.9	114.0

Secondary Medical Zone ID: 081

	Male						Female					
	Suicide			Suicide			Suicide			Suicide		
	Num per year	Rate /100,000	×100	Num per year	Rate /100,000	×100	Num per year	Rate /100,000	×100	Num per year	Rate /100,000	×100
	Total (>10 y/o)			45–54			Total (>10 y/o)			45–54		
1983–1987	18.2	36.4	122.7	5.0	63.5	125.5	12.8	18.6	142.3	1.2	17.2	99.7
1988–1992	14.6	27.2	123.3	1.6	30.1	87.5	10.6	14.6	131.8	1.2	15.6	106.6
1993–1997	21.2	32.0	158.6	4.6	48.8	133.5	9.6	12.1	132.4	1.0	13.4	103.5
1998–2002	23.2	40.4	128.2	6.6	72.3	129.2	9.6	13.4	118.8	1.8	17.8	118.5
2003–2007	25.6	45.8	143.9	5.6	71.6	128.1	10.4	14.4	129.8	1.8	17.3	126.4
2008–2012	26.2	43.1	152.6	5.6	70.5	145.2	8.8	13.9	118.3	0.8	14.7	98.3
	15–24			55–64			15–24			55–64		
1983–1987	1.2	16.4	117.7	3.2	46.4	109.1	1.0	9.1	133.4	1.8	21.6	109.7
1988–1992	0.8	10.8	108.8	2.0	32.1	90.5	0.4	5.6	106.9	2.0	21.0	117.8
1993–1997	1.0	12.7	112.4	4.2	52.2	129.8	0.2	4.9	93.5	1.2	16.5	103.7
1998–2002	1.4	18.7	118.3	4.6	70.7	111.2	0.2	6.5	92.1	1.2	18.8	100.0
2003–2007	1.2	19.7	113.0	5.0	67.4	116.7	0.6	10.2	113.4	1.6	18.6	111.1
2008–2012	1.0	22.6	106.7	6.2	64.0	131.8	0.6	11.2	115.5	1.0	14.8	99.1
	25–34			65–74			25–34			65–74		
1983–1987	1.2	21.6	84.4	2.4	55.6	127.1	0.8	11.1	103.5	3.0	46.8	149.7
1988–1992	2.0	25.2	131.7	3.4	55.9	158.7	0.4	8.2	94.6	2.0	30.9	118.6
1993–1997	2.4	27.9	142.8	3.8	51.4	161.0	0.4	8.1	97.4	2.4	27.3	140.2
1998–2002	2.4	32.4	119.9	2.8	46.4	102.0	0.0	9.1	78.8	0.6	15.7	74.6
2003–2007	4.0	48.1	155.1	1.6	36.2	84.4	1.2	15.6	119.9	1.2	18.6	100.1
2008–2012	3.8	47.6	146.2	1.8	37.8	95.8	0.0	11.7	81.6	1.4	19.4	109.2
	35–44			>74 y/o			35–44			>74 y/o		
1983–1987	2.6	33.8	106.3	2.6	106.7	136.2	2.4	18.0	152.2	2.6	78.6	136.2
1988–1992	2.6	28.3	119.4	2.2	79.2	122.7	0.8	10.0	103.9	3.8	81.3	169.8
1993–1997	2.8	31.7	129.4	2.4	70.4	135.3	0.6	8.6	101.6	3.8	63.0	183.1
1998–2002	2.4	37.6	106.8	2.8	66.9	126.9	1.2	12.9	124.9	4.4	57.2	184.3
2003–2007	5.0	67.8	169.2	3.2	58.1	129.0	1.2	14.9	117.9	2.8	31.0	138.3
2008–2012	3.6	47.1	126.7	4.2	60.6	146.0	1.6	17.2	126.6	3.4	29.9	155.3

Secondary Medical Zone ID: 082

	Male						Female					
	Suicide			Suicide			Suicide			Suicide		
	Num per year	Rate /100,000	×100	Num per year	Rate /100,000	×100	Num per year	Rate /100,000	×100	Num per year	Rate /100,000	×100
	Total (>10 y/o)			45-54			Total (>10 y/o)			45-54		
1983-1987	23.8	31.8	96.4	6.4	52.4	103.6	19.0	18.6	135.0	3.8	24.0	139.1
1988-1992	20.8	24.5	98.7	5.6	38.3	111.5	14.2	13.5	111.1	1.8	14.3	97.6
1993-1997	28.2	27.0	113.7	7.4	41.4	113.0	14.6	11.6	118.7	2.4	13.9	107.9
1998-2002	36.2	35.0	96.1	8.6	51.5	91.9	14.2	12.2	97.9	2.6	15.3	101.9
2003-2007	37.8	35.7	95.3	8.6	55.6	99.4	13.4	11.8	93.7	0.8	10.0	73.1
2008-2012	41.6	35.7	107.6	8.2	53.1	109.2	14.2	12.6	96.2	1.4	12.7	85.1
	15-24			55-64			15-24			55-64		
1983-1987	2.4	17.1	122.6	2.8	35.6	83.8	2.0	10.4	152.7	2.0	19.6	99.3
1988-1992	0.8	8.1	81.9	2.6	27.8	78.3	1.0	6.0	114.5	1.8	16.9	94.4
1993-1997	3.4	16.0	141.5	4.0	35.9	89.1	0.2	3.7	70.3	2.8	19.4	122.1
1998-2002	4.2	22.6	143.1	7.6	57.9	91.1	0.8	6.6	94.3	2.0	17.1	90.6
2003-2007	1.6	15.3	87.6	6.4	44.1	76.4	1.2	9.3	103.6	3.0	17.7	105.7
2008-2012	2.6	21.6	101.9	8.2	48.6	100.1	1.4	10.8	110.8	2.2	14.1	94.7
	25-34			65-74			25-34			65-74		
1983-1987	3.8	27.9	109.3	2.0	40.1	91.6	2.2	13.8	127.9	3.2	38.9	124.3
1988-1992	3.0	21.8	114.2	1.0	24.6	69.7	2.0	11.8	135.8	2.0	24.6	94.3
1993-1997	1.6	14.5	74.2	3.2	36.5	114.4	1.0	7.8	94.0	2.8	24.6	126.1
1998-2002	3.2	22.2	82.2	3.8	41.4	91.0	1.2	9.7	84.0	2.4	21.4	101.7
2003-2007	4.6	28.3	91.1	4.4	41.8	97.4	1.2	10.3	79.1	2.0	17.8	95.5
2008-2012	6.0	36.6	112.4	5.6	43.6	110.3	1.8	13.6	94.7	1.6	15.4	86.6
	35-44			>74 y/o			35-44			>74 y/o		
1983-1987	4.4	28.6	89.9	2.0	71.3	91.0	1.8	11.8	99.8	4.0	80.5	139.5
1988-1992	3.8	23.2	97.8	4.0	91.3	141.6	1.2	8.8	91.0	4.4	66.9	139.7
1993-1997	5.4	31.6	128.8	3.2	65.5	125.7	0.6	6.5	76.8	4.8	56.4	163.9
1998-2002	5.4	35.4	100.5	3.4	59.5	112.8	1.0	8.9	85.9	4.2	40.8	131.2
2003-2007	8.6	49.9	124.6	3.6	48.6	107.9	2.0	12.9	101.9	3.2	25.9	115.5
2008-2012	6.0	34.5	92.8	5.0	51.0	122.9	2.8	14.9	109.7	3.0	20.6	107.0

Secondary Medical Zone ID: 083

	Male						Female					
	Suicide			Suicide			Suicide			Suicide		
	Num per year	Rate /100,000	×100	Num per year	Rate /100,000	×100	Num per year	Rate /100,000	×100	Num per year	Rate /100,000	×100
	Total (>10 y/o)			45-54			Total (>10 y/o)			45-54		
1983-1987	26.4	33.3	103.3	8.0	60.4	119.5	18.4	17.6	124.0	3.0	19.8	114.9
1988-1992	19.8	24.0	96.7	3.6	29.3	85.3	13.2	12.6	101.3	1.6	13.3	90.8
1993-1997	22.0	24.7	100.1	4.4	32.3	88.3	12.4	10.7	105.8	2.0	13.0	100.9
1998-2002	32.6	35.9	101.4	7.6	54.8	98.0	13.0	12.4	97.5	2.2	15.2	101.3
2003-2007	36.4	38.9	114.0	5.4	49.7	88.9	12.6	12.6	100.3	1.0	11.7	85.7
2008-2012	33.8	37.1	115.0	4.6	46.7	96.0	14.0	13.6	110.2	2.6	19.1	127.9
	15-24			55-64			15-24			55-64		
1983-1987	1.2	11.3	81.0	3.6	38.8	91.3	0.6	6.0	88.2	2.4	20.1	101.8
1988-1992	1.4	9.9	99.8	3.4	31.9	89.8	0.2	3.8	71.8	2.2	17.6	98.7
1993-1997	1.6	11.5	101.9	5.0	41.7	103.5	0.2	3.9	74.5	1.0	11.7	73.6
1998-2002	2.6	18.8	119.1	9.0	69.1	108.8	0.6	6.7	94.8	2.4	18.3	97.4
2003-2007	1.8	18.0	103.3	9.4	64.8	112.3	0.6	8.4	93.1	2.4	16.6	99.3
2008-2012	1.6	20.6	97.0	8.2	55.9	115.1	0.8	10.3	106.0	1.8	14.1	94.6
	25-34			65-74			25-34			65-74		
1983-1987	3.2	26.2	102.5	2.6	44.2	100.9	2.0	13.4	124.3	4.6	45.8	146.5
1988-1992	2.8	22.7	118.6	2.0	32.1	91.2	0.4	6.8	78.1	3.2	31.0	118.7
1993-1997	2.2	19.4	99.3	2.6	31.3	98.2	0.6	7.3	87.3	2.4	21.2	108.6
1998-2002	3.2	26.6	98.6	3.6	40.7	89.4	1.4	11.8	101.5	2.0	18.9	89.8
2003-2007	3.8	32.5	104.7	4.8	45.9	106.9	1.8	14.5	111.4	2.2	18.5	99.1
2008-2012	3.6	35.6	109.2	6.0	48.2	122.1	1.0	13.6	94.6	3.2	21.3	119.4
	35-44			>74 y/o			35-44			>74 y/o		
1983-1987	4.6	30.0	94.4	3.0	88.9	113.5	3.0	15.1	128.0	2.8	58.7	101.7
1988-1992	3.0	21.7	91.7	3.6	81.1	125.8	0.8	8.0	82.9	4.8	67.9	141.9
1993-1997	2.8	23.8	97.3	3.4	66.7	128.1	0.8	7.8	91.7	5.4	59.2	172.0
1998-2002	3.4	33.2	94.4	3.2	55.9	106.0	1.2	10.7	103.8	3.2	31.3	100.9
2003-2007	6.0	50.4	125.7	5.2	63.6	141.1	1.4	12.8	101.6	3.2	24.9	111.1
2008-2012	6.6	46.5	125.0	3.2	39.4	94.9	1.4	13.1	97.0	3.2	21.4	111.1

Secondary Medical Zone ID: 084

	Male						Female					
	Suicide			Suicide			Suicide			Suicide		
	Num per year	Rate /100,000	×100	Num per year	Rate /100,000	×100	Num per year	Rate /100,000	×100	Num per year	Rate /100,000	×100
	Total (>10 y/o)			45–54			Total (>10 y/o)			45–54		
1983–1987	50.4	34.4	106.6	8.2	39.8	78.8	29.2	17.6	121.5	5.0	21.5	124.6
1988–1992	42.8	25.8	107.6	7.2	28.2	82.0	29.4	15.2	128.0	6.2	20.6	140.6
1993–1997	45.4	25.5	102.8	10.4	33.2	90.7	22.2	11.1	106.6	3.0	11.1	86.1
1998–2002	60.0	32.4	90.1	14.0	46.0	82.2	28.0	12.7	109.2	3.8	13.5	89.7
2003–2007	68.8	36.1	99.3	12.8	49.4	88.4	26.2	12.6	104.6	3.2	13.1	95.6
2008–2012	65.6	34.8	101.2	11.4	48.7	100.2	25.0	12.7	100.8	2.8	13.8	92.6
	15–24			55–64			15–24			55–64		
1983–1987	4.2	16.0	114.6	7.8	47.6	111.9	1.4	6.4	93.9	5.4	25.9	131.3
1988–1992	4.2	12.5	126.1	8.6	43.2	121.7	0.6	3.6	68.3	4.2	20.2	113.0
1993–1997	2.8	10.3	91.0	8.8	41.2	102.4	1.0	4.4	84.9	3.8	17.5	109.9
1998–2002	4.2	16.5	104.5	13.4	54.8	86.3	0.8	5.2	74.7	5.2	20.4	108.3
2003–2007	5.0	21.3	121.9	17.6	57.9	100.4	1.4	8.1	89.7	3.6	14.0	83.7
2008–2012	5.8	27.1	127.7	13.4	45.0	92.6	1.2	8.4	86.5	5.4	16.6	111.3
	25–34			65–74			25–34			65–74		
1983–1987	6.2	24.2	94.5	5.0	47.2	107.9	2.2	9.6	89.4	4.8	34.5	110.3
1988–1992	4.4	18.3	95.6	4.0	35.6	101.0	3.4	12.3	141.6	5.4	32.6	124.8
1993–1997	6.0	21.7	111.2	3.8	29.0	90.9	2.2	8.9	106.8	4.2	23.0	118.2
1998–2002	8.6	28.1	104.2	7.6	46.6	102.4	1.8	8.9	76.8	3.6	20.2	96.2
2003–2007	10.2	33.4	107.7	9.6	49.9	116.2	3.8	14.0	107.5	5.2	23.6	126.8
2008–2012	9.6	36.1	110.9	7.6	35.7	90.4	3.0	14.0	97.4	4.0	17.7	99.4
	35–44			>74 y/o			35–44			>74 y/o		
1983–1987	11.4	33.7	105.7	7.6	131.7	168.1	3.6	11.7	99.3	6.6	86.7	150.4
1988–1992	9.0	26.8	113.1	5.0	71.7	111.1	3.4	10.5	108.6	6.0	58.1	121.3
1993–1997	7.0	25.7	105.0	6.4	73.4	140.9	2.2	8.6	100.6	5.8	42.0	122.1
1998–2002	4.8	22.9	65.1	7.0	68.5	130.0	2.6	11.0	106.4	10.2	55.7	179.3
2003–2007	9.4	35.9	89.6	3.8	35.3	78.4	2.8	12.2	96.3	6.2	29.0	129.4
2008–2012	9.0	31.4	84.4	8.8	56.8	136.9	3.2	12.6	92.7	5.2	21.6	112.5

Secondary Medical Zone ID: 085

	Male						Female					
	Suicide			Suicide			Suicide			Suicide		
	Num per year	Rate /100,000	×100	Num per year	Rate /100,000	×100	Num per year	Rate /100,000	×100	Num per year	Rate /100,000	×100
	Total (>10 y/o)			45-54			Total (>10 y/o)			45-54		
1983-1987	71.4	30.4	90.7	18.8	42.9	84.9	38.6	16.4	109.3	7.6	18.1	104.8
1988-1992	56.8	21.6	83.7	12.6	25.4	74.0	33.4	12.3	97.1	8.2	16.2	110.8
1993-1997	75.4	24.1	96.4	25.2	43.3	118.3	37.2	12.0	111.2	7.8	14.4	111.1
1998-2002	111.4	33.3	91.9	29.2	55.2	98.6	36.6	11.5	88.7	6.6	14.1	93.7
2003-2007	116.4	33.2	90.2	24.6	52.5	93.9	42.4	12.2	98.2	3.6	10.3	74.9
2008-2012	124.0	33.4	99.2	22.6	46.4	95.4	48.4	13.5	105.1	6.4	15.2	102.2
	15-24			55-64			15-24			55-64		
1983-1987	4.8	10.2	73.4	11.0	46.5	109.4	2.8	6.1	89.9	5.4	21.8	110.7
1988-1992	6.0	10.3	104.2	11.6	34.8	97.9	2.8	5.2	99.3	5.2	16.9	94.4
1993-1997	4.0	8.7	77.2	16.6	39.7	98.6	1.6	3.9	74.6	5.6	14.5	91.0
1998-2002	7.2	16.6	104.9	30.0	62.4	98.2	2.8	7.0	100.3	7.8	17.3	91.9
2003-2007	5.6	15.5	88.7	30.6	57.9	100.4	2.4	7.5	83.3	8.8	17.3	103.3
2008-2012	7.6	20.8	98.1	27.4	53.9	110.8	4.6	12.3	127.1	5.6	12.9	86.4
	25-34			65-74			25-34			65-74		
1983-1987	11.0	23.7	92.6	4.2	39.5	90.3	4.6	11.0	101.8	3.8	26.8	85.7
1988-1992	5.0	10.6	55.4	4.8	34.9	98.9	4.2	9.0	103.5	4.0	23.4	89.6
1993-1997	9.8	16.5	84.2	6.0	31.3	98.1	4.8	8.8	106.0	6.2	26.4	135.3
1998-2002	15.0	23.4	86.8	10.2	38.8	85.2	5.6	10.2	87.6	4.2	16.3	77.5
2003-2007	14.6	24.3	78.5	13.0	37.7	88.0	7.2	13.0	100.2	6.4	17.8	95.4
2008-2012	13.6	26.6	81.7	22.0	51.6	130.7	9.0	17.6	123.0	9.2	20.4	114.4
	35-44			>74 y/o			35-44			>74 y/o		
1983-1987	17.4	29.9	94.0	4.2	85.1	108.7	9.0	15.4	130.4	5.4	70.6	122.3
1988-1992	11.8	21.5	90.5	5.0	72.6	112.5	4.2	8.8	91.4	4.4	40.6	84.8
1993-1997	10.0	20.9	85.3	3.8	47.9	91.9	7.0	14.2	166.5	4.2	29.6	86.1
1998-2002	13.2	26.8	76.2	6.4	57.0	108.1	3.4	8.5	82.7	6.2	33.0	106.3
2003-2007	21.4	35.8	89.3	6.4	41.6	92.3	6.8	13.1	103.4	6.6	26.3	117.2
2008-2012	24.0	34.9	93.9	6.6	31.9	77.0	6.4	11.4	83.9	7.2	21.4	111.4

Secondary Medical Zone ID: 086

	Male						Female					
	Suicide			Suicide			Suicide			Suicide		
	Num per year	Rate /100,000	×100	Num per year	Rate /100,000	×100	Num per year	Rate /100,000	×100	Num per year	Rate /100,000	×100
	Total (>10 y/o)			45–54			Total (>10 y/o)			45–54		
1983–1987	52.8	26.9	77.7	16.0	40.1	79.3	28.0	14.7	94.8	6.8	18.1	105.2
1988–1992	46.8	20.6	79.0	13.0	28.1	81.7	27.0	11.9	90.8	6.4	14.1	96.2
1993–1997	63.2	22.7	90.4	19.0	37.2	101.7	25.4	9.6	87.1	4.6	10.1	78.5
1998–2002	89.2	30.2	81.3	20.4	45.5	81.3	39.2	12.7	100.8	8.6	17.7	117.3
2003–2007	91.4	29.8	78.8	17.8	44.9	80.2	41.6	12.9	102.6	5.0	13.4	97.7
2008–2012	103.8	31.8	93.2	17.4	42.2	86.8	45.6	14.0	105.7	5.4	14.5	97.2
	15–24			55–64			15–24			55–64		
1983–1987	6.0	12.8	92.0	4.4	28.8	67.9	3.4	7.7	112.0	1.6	12.9	65.3
1988–1992	4.2	7.9	79.9	8.8	31.3	88.1	2.2	4.6	86.7	4.0	16.2	90.7
1993–1997	6.6	12.0	105.8	14.6	38.2	94.9	4.2	7.5	144.6	3.8	12.0	75.6
1998–2002	7.0	15.7	99.4	24.6	55.6	87.5	3.2	7.6	107.8	8.2	18.5	98.5
2003–2007	6.4	17.2	98.7	21.4	46.1	79.9	3.6	10.0	111.4	7.2	15.3	91.3
2008–2012	6.4	19.2	90.5	19.8	45.9	94.5	4.0	11.4	117.4	5.6	13.2	88.7
	25–34			65–74			25–34			65–74		
1983–1987	8.4	22.2	86.7	1.6	27.6	63.0	3.8	10.5	97.2	3.6	31.6	101.0
1988–1992	5.8	13.9	72.5	4.2	38.2	108.4	3.0	8.0	91.9	3.2	23.8	91.3
1993–1997	7.6	14.8	75.7	4.6	29.8	93.3	4.0	8.3	100.3	2.8	17.0	87.2
1998–2002	10.0	17.8	66.0	9.0	38.6	84.9	7.4	12.8	110.8	4.6	19.7	93.6
2003–2007	12.6	23.0	74.0	11.0	34.8	81.2	9.0	16.1	123.7	7.2	20.9	112.3
2008–2012	15.2	32.1	98.5	16.2	41.1	104.2	9.2	18.9	132.1	7.2	17.3	96.9
	35–44			>74 y/o			35–44			>74 y/o		
1983–1987	13.2	25.5	80.2	3.2	85.9	109.7	5.0	10.1	85.1	3.4	60.6	105.0
1988–1992	7.6	16.9	71.4	3.0	57.9	89.7	3.8	8.6	89.1	4.4	48.4	101.1
1993–1997	8.2	20.2	82.6	2.2	38.0	73.0	2.2	6.6	77.9	3.6	30.3	88.1
1998–2002	11.6	26.8	76.0	5.8	61.3	116.4	2.8	8.1	78.6	4.4	28.1	90.6
2003–2007	17.4	32.7	81.7	4.6	36.7	81.5	5.6	12.0	95.2	3.8	18.7	83.3
2008–2012	19.4	32.3	86.8	9.4	45.1	108.6	9.6	16.1	118.7	4.4	16.1	83.5

Secondary Medical Zone ID: 087

	Male						Female					
	Suicide			Suicide			Suicide			Suicide		
	Num per year	Rate /100,000	×100	Num per year	Rate /100,000	×100	Num per year	Rate /100,000	×100	Num per year	Rate /100,000	×100
	Total (>10 y/o)			45-54			Total (>10 y/o)			45-54		
1983-1987	88.0	27.6	81.2	25.4	39.6	78.3	43.6	14.4	92.0	10.2	18.4	106.6
1988-1992	77.4	21.1	79.6	18.4	22.7	66.0	49.6	12.7	99.8	10.4	13.7	93.8
1993-1997	105.4	23.0	90.0	28.2	30.7	83.9	49.4	10.7	99.0	9.4	10.7	82.8
1998-2002	166.8	32.9	90.2	43.4	51.6	92.1	64.0	12.7	99.0	11.4	13.6	90.6
2003-2007	170.6	32.8	88.8	31.2	45.8	81.9	69.0	13.0	103.1	7.0	11.0	80.6
2008-2012	172.6	32.6	94.0	31.2	47.4	97.5	72.6	13.6	103.5	10.6	16.9	113.3
	15-24			55-64			15-24			55-64		
1983-1987	9.6	13.6	97.7	10.6	40.3	94.9	5.2	7.5	109.9	3.8	15.5	78.4
1988-1992	7.0	8.0	81.2	11.4	26.7	75.3	4.4	5.1	96.6	6.8	17.4	97.7
1993-1997	8.6	10.0	88.1	25.0	38.9	96.6	7.2	7.8	149.0	7.4	13.6	85.6
1998-2002	11.0	15.3	96.8	43.6	54.4	85.7	6.4	8.7	123.8	10.6	14.8	78.7
2003-2007	12.8	20.5	117.4	42.0	48.0	83.2	6.2	10.4	115.1	13.4	15.3	91.6
2008-2012	12.6	22.4	105.6	36.8	45.9	94.4	6.0	10.9	112.0	12.2	14.5	97.3
	25-34			65-74			25-34			65-74		
1983-1987	10.6	19.2	75.3	5.2	41.1	93.8	4.4	8.1	75.2	4.8	26.9	86.2
1988-1992	10.6	16.7	87.2	6.4	37.9	107.6	6.8	10.5	120.9	4.8	21.8	83.7
1993-1997	14.6	18.3	93.5	6.4	27.1	84.9	4.8	6.9	82.4	5.6	19.8	101.6
1998-2002	23.4	25.6	94.9	15.6	41.2	90.6	10.8	12.5	108.1	6.8	18.7	89.0
2003-2007	23.0	27.1	87.5	24.0	42.8	99.8	11.6	14.3	109.9	10.2	19.0	101.9
2008-2012	17.6	25.8	79.2	25.2	35.6	90.1	9.6	14.4	100.2	12.4	17.2	96.8
	35-44			>74 y/o			35-44			>74 y/o		
1983-1987	23.2	25.4	79.8	3.4	63.6	81.2	8.8	9.9	83.7	6.4	68.1	118.0
1988-1992	16.0	19.2	80.8	7.2	81.7	126.6	7.8	9.2	95.2	8.6	56.4	117.9
1993-1997	15.6	22.2	90.5	6.8	62.8	120.5	6.8	9.6	112.6	8.2	40.1	116.6
1998-2002	21.6	31.7	90.0	8.0	56.7	107.5	8.8	13.1	126.8	9.2	34.8	112.2
2003-2007	27.4	34.5	86.2	9.6	46.1	102.3	10.8	14.5	114.5	9.4	27.2	121.4
2008-2012	33.8	35.2	94.6	15.4	47.7	115.0	12.6	14.4	106.5	9.0	19.9	103.5

Secondary Medical Zone ID: 088

	Male						Female					
	Suicide			Suicide			Suicide			Suicide		
	Num per year	Rate /100,000	×100	Num per year	Rate /100,000	×100	Num per year	Rate /100,000	×100	Num per year	Rate /100,000	×100
	Total (>10 y/o)			45–54			Total (>10 y/o)			45–54		
1983–1987	92.8	26.7	78.4	23.8	37.5	74.1	52.2	14.6	94.3	10.4	17.0	98.7
1988–1992	79.8	19.5	76.9	19.8	26.3	76.5	53.6	12.3	96.7	11.4	15.0	102.6
1993–1997	102.4	21.6	83.9	22.4	26.9	73.6	55.4	10.9	101.5	10.4	12.2	94.5
1998–2002	153.8	29.2	80.1	37.0	44.9	80.2	68.2	12.3	97.4	12.6	15.2	100.9
2003–2007	155.0	28.1	76.0	27.8	37.1	66.3	72.8	12.8	100.0	9.8	13.5	98.5
2008–2012	180.2	31.8	91.5	35.0	44.1	90.7	88.0	14.6	113.6	9.2	12.9	86.2
	15–24			55–64			15–24			55–64		
1983–1987	10.2	13.8	99.0	13.0	35.7	84.0	2.4	4.3	63.0	5.2	14.7	74.3
1988–1992	8.4	9.4	95.4	12.4	25.6	72.2	3.8	4.8	90.7	8.4	17.2	96.2
1993–1997	9.8	11.2	98.8	23.6	37.8	94.0	5.8	6.8	130.4	8.6	14.4	90.8
1998–2002	10.8	14.6	92.5	36.4	50.3	79.1	6.4	8.8	125.2	12.0	16.8	89.0
2003–2007	11.8	17.5	100.5	39.0	48.1	83.3	5.8	9.3	103.9	15.0	17.7	105.6
2008–2012	13.4	20.9	98.5	37.4	47.1	97.0	6.4	10.5	108.5	11.8	14.7	98.5
	25–34			65–74			25–34			65–74		
1983–1987	14.2	22.3	87.1	6.8	36.1	82.5	7.8	12.3	114.6	10.8	40.2	128.7
1988–1992	12.0	16.3	85.4	6.8	29.6	84.1	4.8	7.4	85.2	7.6	25.0	95.9
1993–1997	13.6	15.6	79.7	7.6	24.4	76.5	7.6	9.1	109.4	7.8	20.6	105.5
1998–2002	24.8	25.2	93.3	13.8	33.3	73.1	8.8	9.9	85.8	11.0	23.1	110.0
2003–2007	21.8	24.0	77.5	20.4	37.8	88.1	10.8	12.4	95.6	10.6	18.4	99.0
2008–2012	24.2	30.8	94.5	23.0	36.0	91.2	15.6	19.5	136.2	14.6	20.4	114.9
	35–44			>74 y/o			35–44			>74 y/o		
1983–1987	17.0	21.1	66.2	7.6	78.5	100.2	8.6	10.4	87.8	6.8	50.6	87.7
1988–1992	14.0	17.6	74.2	6.2	49.7	77.0	8.4	10.3	106.2	9.2	45.8	95.7
1993–1997	13.4	18.0	73.3	11.6	69.9	134.2	5.8	8.2	96.3	9.4	35.2	102.4
1998–2002	21.0	26.3	74.8	9.6	46.8	88.7	6.8	9.3	90.2	10.6	31.0	99.8
2003–2007	24.4	27.1	67.7	9.8	35.0	77.8	12.2	13.9	110.1	8.4	18.9	84.4
2008–2012	33.4	33.1	89.0	13.4	35.4	85.4	16.4	16.3	120.4	13.0	22.1	114.8

Secondary Medical Zone ID: 089

	Male						Female					
	Suicide			Suicide			Suicide			Suicide		
	Num per year	Rate /100,000	×100	Num per year	Rate /100,000	×100	Num per year	Rate /100,000	×100	Num per year	Rate /100,000	×100
	Total (>10 y/o)			45–54			Total (>10 y/o)			45–54		
1983–1987	43.8	29.4	85.4	11.6	39.3	77.7	25.0	16.1	106.4	7.8	25.1	145.9
1988–1992	39.0	22.2	84.6	11.2	29.7	86.6	23.4	12.4	97.1	6.4	16.7	114.0
1993–1997	48.4	22.6	87.5	11.8	29.2	79.7	26.4	11.2	106.8	4.8	11.7	90.6
1998–2002	73.0	31.1	83.9	19.8	50.2	89.7	35.4	13.8	109.7	6.6	15.7	104.2
2003–2007	70.2	29.7	77.5	12.2	37.7	67.5	32.4	12.2	99.1	3.0	10.5	76.3
2008–2012	88.4	35.3	101.1	18.2	55.2	113.5	33.8	12.5	99.4	5.6	16.9	113.0
	15–24			55–64			15–24			55–64		
1983–1987	4.0	13.0	93.3	7.0	46.0	108.2	1.2	5.1	75.1	2.4	17.7	89.6
1988–1992	3.6	9.2	93.4	5.8	27.8	78.3	1.8	4.9	92.0	4.8	21.2	118.9
1993–1997	3.8	10.1	89.0	11.4	37.7	93.7	2.6	6.2	118.4	4.8	16.9	106.2
1998–2002	6.0	17.1	108.2	15.0	43.0	67.6	1.4	5.4	76.7	6.6	18.4	97.9
2003–2007	6.2	19.9	114.0	17.6	44.9	77.7	2.8	9.6	107.0	6.0	15.0	89.6
2008–2012	7.0	24.6	115.9	17.4	45.5	93.8	1.6	7.5	77.1	5.8	14.4	96.5
	25–34			65–74			25–34			65–74		
1983–1987	5.4	21.5	83.9	3.4	44.7	102.0	2.4	9.5	88.4	4.2	38.4	123.0
1988–1992	4.2	15.4	80.7	4.8	44.8	127.2	1.8	7.2	83.5	2.0	19.2	73.7
1993–1997	6.4	18.4	94.1	4.6	32.8	102.9	3.6	9.7	117.0	2.8	18.6	95.4
1998–2002	11.6	28.5	105.4	9.0	45.0	98.9	7.6	16.9	146.1	3.6	18.9	90.1
2003–2007	10.8	28.5	92.0	9.6	36.5	85.0	5.2	13.7	105.5	5.4	19.7	105.7
2008–2012	10.2	31.7	97.3	10.4	32.4	82.0	3.4	12.2	84.9	6.2	17.8	100.1
	35–44			>74 y/o			35–44			>74 y/o		
1983–1987	10.2	26.0	81.7	2.2	73.5	93.8	3.2	9.0	76.2	3.4	66.8	115.8
1988–1992	5.4	15.5	65.5	4.0	77.0	119.4	3.0	8.3	85.8	3.6	46.4	97.1
1993–1997	6.8	20.4	83.4	3.6	56.2	108.0	1.8	6.4	74.8	6.0	51.7	150.3
1998–2002	7.8	25.5	72.5	3.8	47.5	90.1	5.0	13.8	133.7	4.4	30.8	99.2
2003–2007	9.4	27.4	68.3	4.2	37.3	82.9	3.4	10.8	85.4	6.4	32.2	143.4
2008–2012	15.6	36.9	99.1	9.2	50.5	121.8	4.4	11.9	87.7	6.8	26.2	136.2

Secondary Medical Zone ID: 090

	Male						Female					
	Suicide			Suicide			Suicide			Suicide		
	Num per year	Rate /100,000	×100	Num per year	Rate /100,000	×100	Num per year	Rate /100,000	×100	Num per year	Rate /100,000	×100
	Total (>10 y/o)			45–54			Total (>10 y/o)			45–54		
1983–1987	72.4	30.0	87.8	16.2	38.5	76.2	40.2	15.8	103.8	7.0	18.1	105.0
1988–1992	65.0	22.2	87.4	14.8	26.9	78.2	40.2	12.7	100.6	6.8	13.5	92.5
1993–1997	84.6	24.0	96.0	23.6	35.5	97.0	43.2	11.3	108.7	8.2	12.7	98.6
1998–2002	124.0	32.9	90.7	31.8	50.1	89.6	50.0	12.5	100.0	8.0	13.1	86.8
2003–2007	125.0	33.2	88.6	22.8	43.6	78.0	53.8	12.9	106.2	6.2	12.2	89.0
2008–2012	131.0	34.1	98.3	24.8	52.0	107.0	58.8	13.8	112.5	6.4	14.2	95.2
	15–24			55–64			15–24			55–64		
1983–1987	6.0	11.8	84.7	9.2	38.6	90.8	3.0	6.6	97.0	7.8	26.4	133.7
1988–1992	7.2	10.2	102.6	11.4	33.3	93.7	2.2	4.0	76.4	6.8	19.7	110.3
1993–1997	8.0	11.1	98.3	16.8	37.7	93.7	4.0	5.9	113.7	8.4	19.3	121.4
1998–2002	8.4	13.9	88.1	28.8	52.7	83.0	4.2	7.3	104.6	9.4	18.1	96.0
2003–2007	9.8	18.1	103.8	29.2	46.5	80.5	4.6	9.6	106.2	8.8	14.4	86.3
2008–2012	8.8	19.4	91.4	30.8	49.0	100.9	4.2	10.0	103.4	12.8	18.6	124.7
	25–34			65–74			25–34			65–74		
1983–1987	9.8	22.6	88.4	7.8	54.1	123.7	2.8	7.5	69.7	5.4	29.6	94.7
1988–1992	6.8	15.5	81.0	7.0	39.7	112.8	3.4	8.1	93.3	6.0	26.2	100.6
1993–1997	10.0	19.0	97.3	7.0	30.5	95.7	2.4	5.9	70.6	4.6	17.3	88.8
1998–2002	16.4	27.3	101.2	14.4	46.0	101.2	5.8	10.8	93.5	8.0	23.7	112.7
2003–2007	18.6	32.1	103.6	14.4	36.5	85.2	6.0	11.7	89.9	10.2	24.0	128.6
2008–2012	14.6	30.3	93.1	17.6	35.5	89.8	6.4	14.0	98.1	9.0	17.8	100.2
	35–44			>74 y/o			35–44			>74 y/o		
1983–1987	16.8	27.0	84.8	6.0	86.6	110.6	6.2	10.5	88.6	7.6	73.2	126.9
1988–1992	11.2	18.5	77.9	6.6	69.6	107.8	6.0	9.6	99.9	9.0	56.8	118.6
1993–1997	12.4	22.9	93.5	6.8	57.8	111.0	3.8	7.5	87.8	11.8	54.9	159.5
1998–2002	15.4	31.9	90.5	8.4	56.5	107.2	4.2	9.3	90.0	10.4	38.5	123.9
2003–2007	21.4	41.3	103.1	8.2	41.3	91.8	7.0	14.0	110.9	10.8	31.4	140.1
2008–2012	23.8	39.2	105.4	10.2	38.3	92.4	9.2	16.0	118.0	10.8	25.6	133.1

Secondary Medical Zone ID: 091

	Male						Female					
	Suicide			Suicide			Suicide			Suicide		
	Num per year	Rate /100,000	×100	Num per year	Rate /100,000	×100	Num per year	Rate /100,000	×100	Num per year	Rate /100,000	×100
	Total (>10 y/o)			45–54			Total (>10 y/o)			45–54		
1983–1987	63.6	27.7	79.7	14.0	33.3	65.9	36.2	15.3	99.2	4.8	13.5	78.1
1988–1992	58.6	20.8	81.6	14.8	27.1	78.8	36.0	12.3	94.5	7.0	13.6	92.9
1993–1997	68.0	20.8	80.1	16.6	26.9	73.5	42.2	11.5	109.8	9.0	13.8	106.8
1998–2002	106.8	29.6	80.4	26.0	43.3	77.3	42.2	11.4	87.7	6.8	11.9	78.7
2003–2007	119.2	32.5	85.9	21.4	42.2	75.4	50.0	13.0	100.6	8.0	14.9	108.8
2008–2012	121.2	32.9	92.5	21.2	44.9	92.4	55.0	14.0	106.6	8.4	17.3	116.0
	15–24			55–64			15–24			55–64		
1983–1987	6.8	13.8	99.3	9.2	39.6	93.2	4.4	8.7	128.0	5.8	22.3	112.8
1988–1992	5.8	9.2	93.2	10.2	30.8	86.7	4.2	6.5	122.6	5.2	16.7	93.3
1993–1997	7.8	11.9	105.2	13.4	31.4	77.9	5.0	7.3	140.0	7.2	17.1	107.8
1998–2002	6.8	13.0	82.5	28.4	53.1	83.6	4.0	7.3	104.4	9.4	18.1	96.1
2003–2007	9.2	18.9	108.1	25.6	42.6	73.8	5.0	10.5	117.0	10.0	16.1	96.4
2008–2012	9.4	22.0	103.8	24.6	42.1	86.6	4.2	10.2	105.4	8.8	14.3	96.1
	25–34			65–74			25–34			65–74		
1983–1987	9.2	21.2	82.9	6.6	53.0	121.2	4.6	10.4	96.4	5.2	33.3	106.7
1988–1992	7.4	15.9	83.4	4.4	29.8	84.6	4.0	8.8	101.6	6.8	32.4	124.0
1993–1997	10.2	18.6	95.1	5.0	24.3	76.3	4.6	8.8	105.8	4.4	18.0	92.2
1998–2002	14.2	24.0	88.7	9.4	33.4	73.4	6.4	11.4	98.8	4.2	15.2	72.2
2003–2007	19.8	33.7	108.8	15.0	38.0	88.7	8.8	15.6	119.9	5.6	15.2	81.4
2008–2012	18.0	35.6	109.4	15.6	32.7	82.8	8.2	16.9	117.9	8.2	16.6	93.3
	35–44			>74 y/o			35–44			>74 y/o		
1983–1987	14.0	22.9	72.0	3.4	67.8	86.6	5.6	9.6	81.1	5.4	64.9	112.5
1988–1992	11.4	19.0	80.3	4.4	57.4	89.0	4.0	7.3	75.9	4.8	37.0	77.3
1993–1997	10.4	20.1	81.8	4.2	42.8	82.2	4.4	8.4	99.0	7.6	40.6	118.1
1998–2002	12.4	26.0	73.8	8.6	59.2	112.4	3.8	8.6	83.1	7.2	29.5	95.1
2003–2007	21.0	39.1	97.5	7.2	37.2	82.7	7.4	14.2	112.7	5.2	17.0	75.9
2008–2012	21.4	35.3	94.8	10.4	38.8	93.5	8.6	14.9	109.9	8.4	20.8	108.0

Secondary Medical Zone ID: 092

	Male						Female					
	Suicide			Suicide			Suicide			Suicide		
	Num per year	Rate /100,000	×100	Num per year	Rate /100,000	×100	Num per year	Rate /100,000	×100	Num per year	Rate /100,000	×100
	Total (>10 y/o)			45–54			Total (>10 y/o)			45–54		
1983–1987	70.4	32.4	98.6	19.2	51.0	100.8	44.6	18.2	125.4	7.8	21.4	124.2
1988–1992	59.4	23.8	94.8	13.4	29.1	84.8	42.2	14.6	119.9	8.0	17.4	118.9
1993–1997	65.6	22.9	88.8	14.2	26.7	73.0	37.4	11.3	109.2	6.2	11.9	91.9
1998–2002	108.4	33.7	93.4	26.6	47.7	85.3	48.0	13.3	110.6	8.0	14.5	96.6
2003–2007	108.0	33.6	90.7	20.6	44.0	78.6	48.6	13.5	112.2	6.8	14.1	103.1
2008–2012	101.8	33.0	91.8	16.2	40.9	84.1	45.4	12.8	104.3	6.0	14.8	99.5
	15–24			55–64			15–24			55–64		
1983–1987	6.4	15.7	112.9	9.8	42.4	99.8	2.8	7.2	105.5	5.6	21.2	107.2
1988–1992	3.4	7.7	78.2	8.4	28.9	81.4	1.4	3.7	69.6	7.6	23.0	128.5
1993–1997	7.0	12.7	112.4	13.0	35.0	86.9	2.0	4.4	83.7	6.2	17.0	106.9
1998–2002	7.4	15.7	99.1	23.6	51.8	81.6	3.0	6.9	97.7	7.8	18.0	95.8
2003–2007	7.4	17.9	102.6	23.8	44.9	77.9	6.0	13.9	154.8	8.4	16.0	95.8
2008–2012	8.0	22.2	104.6	23.2	42.7	88.0	3.0	9.4	96.7	9.0	15.8	106.1
	25–34			65–74			25–34			65–74		
1983–1987	7.6	21.4	83.6	7.6	54.1	123.7	4.2	11.1	102.7	8.0	41.7	133.3
1988–1992	6.4	18.0	94.2	5.2	33.0	93.6	3.4	9.2	106.3	7.8	34.5	132.1
1993–1997	9.2	21.9	111.9	7.2	33.5	105.1	3.2	8.1	97.8	5.8	22.0	113.0
1998–2002	13.8	29.2	108.3	14.6	51.7	113.8	5.4	12.1	104.4	6.8	22.6	107.5
2003–2007	15.2	33.1	106.8	14.0	41.3	96.2	5.0	12.3	94.3	8.0	22.1	118.6
2008–2012	17.0	41.1	126.1	12.2	30.8	78.1	5.0	13.9	97.0	8.4	19.5	109.6
	35–44			>74 y/o			35–44			>74 y/o		
1983–1987	13.6	26.5	83.4	5.8	88.2	112.7	7.6	14.1	119.1	8.2	80.7	139.9
1988–1992	14.2	26.1	110.0	8.2	88.4	137.1	3.8	7.9	81.9	10.2	69.7	145.6
1993–1997	9.2	19.9	81.1	5.6	51.9	99.6	3.4	7.5	88.7	10.6	54.6	158.5
1998–2002	12.6	30.5	86.7	9.6	66.7	126.5	4.6	10.8	104.8	12.2	48.2	155.2
2003–2007	16.4	39.2	97.8	10.4	54.9	121.9	4.0	10.8	85.4	10.4	32.9	146.5
2008–2012	16.2	35.1	94.3	8.6	36.8	88.8	4.4	11.3	83.4	9.4	24.5	127.4

Secondary Medical Zone ID: 093

	Male						Female					
	Suicide			Suicide			Suicide			Suicide		
	Num per year	Rate /100,000	×100	Num per year	Rate /100,000	×100	Num per year	Rate /100,000	×100	Num per year	Rate /100,000	×100
	Total (>10 y/o)			45-54			Total (>10 y/o)			45-54		
1983-1987	60.4	32.4	99.0	12.6	43.2	85.5	32.8	15.8	104.8	4.8	17.0	98.4
1988-1992	47.4	22.9	91.6	9.0	27.1	79.0	41.2	15.6	133.0	5.0	15.1	103.6
1993-1997	60.0	25.4	101.5	11.6	29.5	80.8	33.4	12.0	116.8	5.0	12.9	100.0
1998-2002	88.2	34.8	97.5	22.8	53.5	95.5	39.2	13.3	112.0	8.8	19.5	129.5
2003-2007	86.0	34.5	92.7	17.0	46.3	82.8	34.0	12.7	100.5	6.0	15.6	114.1
2008-2012	89.2	35.7	103.1	17.6	52.4	107.9	34.8	12.9	102.6	5.8	17.2	115.3
	15-24			55-64			15-24			55-64		
1983-1987	5.2	15.2	108.9	8.8	41.2	96.9	2.2	7.0	102.5	5.8	22.2	112.3
1988-1992	4.6	11.0	111.2	7.2	28.9	81.5	3.0	7.2	135.6	6.6	22.1	123.8
1993-1997	4.2	10.7	94.6	13.2	43.7	108.6	1.6	4.7	89.2	5.4	17.7	111.3
1998-2002	4.6	13.6	86.4	18.8	56.6	89.1	2.2	6.8	96.7	6.8	20.2	107.3
2003-2007	7.8	22.4	128.2	19.6	50.5	87.5	2.4	8.6	95.1	5.2	14.7	87.6
2008-2012	7.2	24.8	116.9	18.6	45.5	93.7	2.0	8.5	88.1	4.6	12.5	84.1
	25-34			65-74			25-34			65-74		
1983-1987	10.4	30.2	118.2	8.0	57.1	130.5	4.0	12.0	111.2	6.4	36.1	115.5
1988-1992	5.8	18.5	96.7	7.2	44.3	125.9	2.0	7.4	85.9	7.8	35.5	136.1
1993-1997	9.8	26.8	137.1	7.4	37.2	116.6	1.8	6.7	80.6	7.2	27.7	142.2
1998-2002	11.0	28.9	107.0	12.4	52.3	115.0	3.4	10.5	90.8	6.4	23.6	112.5
2003-2007	12.2	33.1	106.8	8.8	35.6	82.9	4.2	12.9	98.9	4.6	17.1	91.8
2008-2012	9.2	30.7	94.1	12.0	40.0	101.4	3.0	12.1	84.3	6.2	19.4	108.9
	35-44			>74 y/o			35-44			>74 y/o		
1983-1987	10.0	25.9	81.5	5.4	82.5	105.4	3.4	9.9	84.1	6.0	58.6	101.6
1988-1992	8.6	21.0	88.7	4.8	58.2	90.2	5.2	11.8	122.7	11.4	77.4	161.8
1993-1997	7.0	19.4	79.1	6.0	57.4	110.2	4.8	11.7	138.0	7.4	40.9	119.0
1998-2002	11.8	34.4	97.7	6.2	49.9	94.6	3.0	9.6	92.5	8.6	37.1	119.4
2003-2007	12.8	37.5	93.6	7.6	46.0	102.1	4.4	13.3	105.1	7.2	25.4	113.4
2008-2012	16.0	41.5	111.6	8.2	41.1	99.1	5.6	15.3	113.1	7.6	22.5	117.1

Secondary Medical Zone ID: 094

	Male						Female					
	Suicide			Suicide			Suicide			Suicide		
	Num per year	Rate /100,000	×100	Num per year	Rate /100,000	×100	Num per year	Rate /100,000	×100	Num per year	Rate /100,000	×100
	Total (>10 y/o)			45-54			Total (>10 y/o)			45-54		
1983-1987	22.0	37.0	123.8	4.2	50.1	99.0	11.4	16.6	114.7	0.8	14.3	82.9
1988-1992	16.0	26.3	113.9	3.8	41.0	119.5	15.2	16.2	154.6	2.0	17.6	120.3
1993-1997	17.8	27.3	118.1	2.4	32.1	87.8	12.6	13.3	146.1	1.0	12.6	97.7
1998-2002	22.2	36.1	105.7	4.6	53.2	95.1	13.8	14.8	137.9	2.0	17.6	117.0
2003-2007	21.4	36.9	104.7	2.8	41.8	74.7	10.0	13.1	115.0	1.8	16.5	120.7
2008-2012	18.8	35.3	102.8	3.6	50.0	102.9	10.4	13.0	119.1	0.8	14.0	94.0
	15-24			55-64			15-24			55-64		
1983-1987	1.4	15.8	113.4	3.6	45.9	108.0	1.0	8.5	124.7	1.2	17.3	87.4
1988-1992	0.8	10.0	101.4	3.4	39.1	110.0	0.6	5.9	112.5	1.6	17.9	100.3
1993-1997	0.6	10.4	92.2	3.6	42.7	106.0	0.8	6.7	128.1	1.6	17.2	108.2
1998-2002	1.0	15.6	99.0	5.4	67.1	105.7	0.4	6.9	97.9	2.4	22.5	119.5
2003-2007	1.6	20.3	116.6	4.4	55.1	95.4	0.8	10.5	116.4	2.2	19.8	118.0
2008-2012	1.2	22.1	104.1	2.8	40.8	83.9	0.4	9.3	96.3	1.6	16.1	107.7
	25-34			65-74			25-34			65-74		
1983-1987	2.8	29.9	117.1	2.4	51.4	117.4	1.6	14.1	130.6	3.0	43.0	137.7
1988-1992	1.6	20.3	106.3	2.8	45.4	128.9	1.4	11.8	135.9	4.2	47.1	180.4
1993-1997	1.8	21.9	111.9	4.0	49.4	154.8	1.4	11.5	137.9	2.4	25.4	130.2
1998-2002	1.4	24.4	90.5	5.0	59.1	129.9	1.6	14.7	127.2	2.2	23.7	112.7
2003-2007	1.4	27.5	88.6	3.8	49.5	115.3	0.2	10.6	81.2	1.4	18.3	98.4
2008-2012	2.0	34.4	105.7	3.2	42.9	108.7	0.2	12.2	84.8	2.2	21.7	121.8
	35-44			>74 y/o			35-44			>74 y/o		
1983-1987	3.6	36.6	115.1	3.8	123.5	157.7	0.8	11.1	93.6	3.0	75.1	130.1
1988-1992	1.8	21.6	91.0	1.8	65.2	101.0	1.6	12.1	125.7	3.8	74.1	154.9
1993-1997	3.2	31.6	128.9	2.2	61.3	117.7	1.6	12.2	143.0	3.8	59.2	172.0
1998-2002	2.4	33.6	95.4	2.4	56.3	106.7	0.8	10.6	103.0	4.4	53.4	172.1
2003-2007	4.0	50.6	126.2	3.4	56.7	126.0	0.2	10.1	80.0	3.4	33.8	150.6
2008-2012	3.4	42.2	113.5	2.6	42.1	101.4	0.6	12.6	92.9	4.6	35.2	183.2

Secondary Medical Zone ID: 095

	Male						Female					
	Suicide			Suicide			Suicide			Suicide		
	Num per year	Rate /100,000	×100	Num per year	Rate /100,000	×100	Num per year	Rate /100,000	×100	Num per year	Rate /100,000	×100
	Total (>10 y/o)			45–54			Total (>10 y/o)			45–54		
1983–1987	84.0	28.8	86.1	23.2	41.2	81.6	37.8	13.7	86.5	6.0	13.1	75.8
1988–1992	59.4	18.5	71.8	17.0	26.1	75.9	36.6	11.0	84.7	6.2	10.8	73.7
1993–1997	81.6	21.3	83.8	21.6	30.7	83.8	32.4	8.6	77.5	8.4	11.8	91.3
1998–2002	126.6	30.1	83.0	29.6	45.7	81.7	46.0	11.0	84.6	9.4	14.2	94.2
2003–2007	129.0	30.0	80.6	26.0	45.9	82.1	47.8	11.3	84.8	4.6	9.5	69.3
2008–2012	133.6	31.8	89.5	28.4	50.0	102.9	47.8	11.2	83.1	7.2	13.8	92.4
	15–24			55–64			15–24			55–64		
1983–1987	8.0	13.1	94.2	9.6	36.9	86.8	4.8	7.9	115.5	5.4	19.5	98.8
1988–1992	7.0	9.4	95.2	10.8	27.6	77.8	4.4	6.1	115.7	7.2	18.9	105.6
1993–1997	9.0	12.0	106.4	18.6	35.3	87.7	2.2	3.8	71.9	6.2	13.3	83.5
1998–2002	7.6	13.1	82.8	34.4	55.1	86.7	5.0	8.4	119.9	9.2	15.7	83.3
2003–2007	6.6	13.7	78.6	34.6	51.1	88.5	3.6	7.9	87.5	9.6	14.2	84.7
2008–2012	10.0	22.2	104.6	27.2	43.5	89.6	3.6	8.9	91.4	6.6	11.1	74.3
	25–34			65–74			25–34			65–74		
1983–1987	12.2	22.5	88.2	5.4	42.3	96.7	6.4	11.4	105.8	3.2	21.4	68.6
1988–1992	7.4	13.8	72.0	3.6	24.8	70.5	3.6	7.2	82.7	2.6	14.8	56.6
1993–1997	12.0	17.8	91.1	6.0	26.1	81.8	4.0	6.9	82.7	4.4	16.8	86.2
1998–2002	21.0	27.6	102.0	14.0	39.8	87.5	6.6	9.9	85.3	5.8	17.1	81.5
2003–2007	20.4	29.4	94.9	16.0	34.2	79.6	9.0	13.4	102.9	5.8	13.4	72.1
2008–2012	14.6	27.9	85.7	18.6	32.9	83.3	5.8	11.7	81.5	8.6	15.0	84.1
	35–44			>74 y/o			35–44			>74 y/o		
1983–1987	21.4	28.5	89.5	3.8	65.5	83.6	6.0	8.5	71.8	5.6	58.0	100.5
1988–1992	9.6	15.0	63.4	3.8	48.4	75.1	6.6	9.5	98.5	5.4	38.3	80.1
1993–1997	9.4	16.9	69.1	5.0	46.9	90.1	1.8	4.4	52.2	5.4	28.5	82.8
1998–2002	14.0	24.6	70.0	6.0	42.1	80.0	4.8	9.1	87.6	5.2	21.6	69.5
2003–2007	18.4	27.6	68.8	6.6	32.3	71.6	10.0	14.9	117.8	5.2	16.1	71.9
2008–2012	24.8	33.4	89.9	9.8	32.2	77.7	8.6	12.2	90.3	7.4	16.9	88.1

Secondary Medical Zone ID: 096

	Male						Female					
	Suicide			Suicide			Suicide			Suicide		
	Num per year	Rate /100,000	×100	Num per year	Rate /100,000	×100	Num per year	Rate /100,000	×100	Num per year	Rate /100,000	×100
	Total (>10 y/o)			45-54			Total (>10 y/o)			45-54		
1983-1987	126.0	25.4	74.1	32.2	34.7	68.5	61.6	12.9	81.9	10.4	12.8	74.3
1988-1992	106.8	18.6	71.9	28.4	25.5	74.2	70.2	11.9	92.6	15.0	13.9	95.1
1993-1997	138.6	20.8	80.7	34.4	28.5	77.8	61.6	9.2	84.2	12.0	10.2	78.7
1998-2002	208.4	28.2	77.8	51.4	45.6	81.5	78.2	10.7	83.1	13.6	12.3	81.7
2003-2007	225.4	29.2	79.4	38.8	39.7	70.9	91.4	12.0	92.3	10.4	11.3	82.1
2008-2012	222.0	29.0	82.2	38.0	37.6	77.4	108.4	13.1	103.8	10.6	11.9	79.6
	15-24			55-64			15-24			55-64		
1983-1987	12.8	11.5	82.1	17.8	38.4	90.5	6.4	6.4	94.2	6.2	14.1	71.6
1988-1992	9.8	7.4	74.6	18.6	27.3	76.8	4.2	3.7	70.5	10.2	16.0	89.9
1993-1997	12.8	9.7	86.1	31.2	35.3	87.6	4.6	4.1	79.3	13.2	15.8	99.4
1998-2002	15.8	14.4	90.9	51.6	49.9	78.5	7.4	7.5	107.2	14.6	14.8	78.6
2003-2007	17.4	18.6	106.4	56.2	49.5	85.8	7.6	9.2	102.1	15.8	13.9	82.9
2008-2012	18.8	22.6	106.5	42.6	40.7	83.9	6.2	8.4	86.4	21.2	18.4	123.2
	25-34			65-74			25-34			65-74		
1983-1987	14.6	14.9	58.2	7.4	34.7	79.3	8.4	8.7	80.7	9.0	30.0	95.9
1988-1992	14.6	12.8	66.7	5.8	22.6	64.2	8.6	8.3	95.8	7.4	20.8	79.9
1993-1997	19.8	14.6	74.9	8.6	22.3	70.0	7.4	6.5	78.5	10.0	21.4	109.8
1998-2002	32.0	21.4	79.2	21.4	37.2	81.8	16.2	12.0	103.6	10.2	17.5	83.4
2003-2007	35.0	24.7	79.7	23.8	31.8	74.1	17.0	12.9	99.2	12.2	16.0	85.8
2008-2012	33.2	28.3	86.9	28.8	32.0	81.0	17.8	15.6	108.9	19.4	19.6	109.9
	35-44			>74 y/o			35-44			>74 y/o		
1983-1987	32.0	24.9	78.1	8.4	79.6	101.6	11.8	9.5	80.5	8.8	54.7	94.7
1988-1992	19.6	16.6	70.0	9.8	66.3	102.8	11.2	9.4	97.3	13.4	54.2	113.2
1993-1997	21.8	21.2	86.3	9.6	53.0	101.8	6.0	6.4	75.9	8.4	26.8	77.7
1998-2002	27.0	25.2	71.5	8.4	36.8	69.9	7.2	7.8	75.6	9.0	22.4	72.0
2003-2007	40.8	31.4	78.3	13.4	38.8	86.1	17.6	14.7	116.6	10.8	19.9	88.7
2008-2012	44.0	30.1	80.8	16.0	32.7	78.8	19.8	14.3	105.8	13.4	18.9	98.1

Secondary Medical Zone ID: 097

	Male						Female					
	Suicide			Suicide			Suicide			Suicide		
	Num per year	Rate /100,000	×100	Num per year	Rate /100,000	×100	Num per year	Rate /100,000	×100	Num per year	Rate /100,000	×100
	Total (>10 y/o)			45–54			Total (>10 y/o)			45–54		
1983–1987	102.2	26.0	74.9	26.4	34.9	69.0	59.6	15.0	96.2	9.4	14.1	81.6
1988–1992	100.2	21.1	82.9	27.0	28.8	83.8	56.0	11.6	89.1	9.6	11.3	77.2
1993–1997	120.8	21.9	85.8	34.4	32.9	90.0	54.4	9.7	88.0	11.2	10.8	83.9
1998–2002	175.4	29.5	80.5	42.8	45.0	80.4	75.6	11.9	94.9	13.0	13.2	87.6
2003–2007	183.4	30.4	81.0	34.2	43.1	77.0	84.2	12.8	102.7	10.6	13.0	94.6
2008–2012	192.4	31.6	90.0	30.2	40.2	82.6	89.0	13.5	104.8	10.8	14.6	97.5
	15–24			55–64			15–24			55–64		
1983–1987	10.8	12.6	90.2	12.4	32.8	77.3	5.0	6.4	93.3	7.0	17.9	90.5
1988–1992	11.8	10.6	107.0	13.2	23.8	67.1	5.6	5.6	105.3	8.0	15.2	85.3
1993–1997	9.4	9.1	80.4	25.2	34.0	84.4	4.6	4.7	90.7	10.6	15.2	95.3
1998–2002	13.8	15.3	97.0	41.0	46.4	73.0	7.4	8.5	120.6	16.4	18.5	98.4
2003–2007	11.8	16.0	91.9	37.8	38.9	67.3	5.4	8.0	88.7	14.6	14.5	86.5
2008–2012	13.8	21.4	100.7	40.6	44.0	90.7	5.0	8.6	88.9	16.4	16.1	107.7
	25–34			65–74			25–34			65–74		
1983–1987	12.6	17.4	68.1	8.6	45.6	104.1	6.0	8.2	76.6	11.4	42.9	137.2
1988–1992	13.4	16.2	85.0	8.8	35.5	100.8	6.4	8.3	95.7	8.8	27.7	106.0
1993–1997	15.6	16.4	83.9	7.2	21.6	67.7	5.0	6.2	74.2	7.6	19.0	97.5
1998–2002	27.4	25.9	95.9	19.0	38.1	83.7	10.8	11.2	96.2	8.8	17.5	83.2
2003–2007	29.8	29.8	96.0	25.0	37.8	88.0	15.6	16.1	123.6	12.8	18.9	101.8
2008–2012	29.4	34.2	105.0	25.4	32.0	80.9	14.6	17.3	121.1	16.4	19.1	107.5
	35–44			>74 y/o			35–44			>74 y/o		
1983–1987	24.2	23.1	72.7	6.6	75.3	96.2	12.2	11.4	96.4	8.4	62.2	107.7
1988–1992	17.4	17.8	75.1	8.2	65.1	101.0	7.6	7.8	80.8	9.8	46.8	97.8
1993–1997	16.8	20.3	83.0	10.8	65.4	125.6	6.2	7.6	89.2	9.0	32.1	93.3
1998–2002	22.2	27.8	78.9	9.0	42.8	81.3	6.0	8.2	79.5	12.8	34.3	110.3
2003–2007	32.4	35.5	88.5	12.0	38.4	85.3	11.2	13.0	103.3	14.0	27.9	124.4
2008–2012	37.4	35.6	95.8	14.6	33.6	80.9	15.2	15.1	111.7	10.4	16.4	85.4

Secondary Medical Zone ID: 098

	Male						Female					
	Suicide			Suicide			Suicide			Suicide		
	Num per year	Rate /100,000	×100	Num per year	Rate /100,000	×100	Num per year	Rate /100,000	×100	Num per year	Rate /100,000	×100
	Total (>10 y/o)			45–54			Total (>10 y/o)			45–54		
1983–1987	50.4	30.1	88.8	13.4	44.9	88.8	26.2	14.7	96.2	4.8	17.8	103.3
1988–1992	48.8	22.6	89.1	13.0	30.9	89.8	30.0	12.8	99.8	5.8	15.0	102.9
1993–1997	54.6	20.7	79.6	15.2	28.9	78.9	26.0	9.4	84.8	5.4	11.1	86.0
1998–2002	101.4	32.4	89.7	25.8	46.5	83.0	38.4	11.9	93.5	7.2	13.4	89.1
2003–2007	99.0	31.8	82.5	22.2	46.2	82.6	42.0	12.6	97.7	4.4	10.2	74.5
2008–2012	109.0	32.7	94.2	17.4	41.7	85.8	41.0	11.9	92.2	6.0	14.2	95.1
	15–24			55–64			15–24			55–64		
1983–1987	5.6	17.5	125.2	5.8	34.9	82.2	1.4	5.7	82.8	4.6	22.2	112.6
1988–1992	4.2	9.7	97.7	8.2	32.6	91.7	2.2	5.2	99.1	4.6	18.3	102.3
1993–1997	7.8	14.3	126.0	9.8	30.4	75.6	2.6	5.3	101.2	2.0	9.0	56.9
1998–2002	9.4	18.7	118.7	27.0	59.8	94.2	2.2	5.5	77.9	6.6	16.7	88.9
2003–2007	8.8	19.8	113.2	22.4	42.0	72.8	3.2	8.0	89.4	8.0	15.5	92.6
2008–2012	7.4	20.1	94.6	26.2	46.3	95.2	2.8	8.3	85.6	7.8	13.9	93.4
	25–34			65–74			25–34			65–74		
1983–1987	6.0	21.7	84.7	2.8	33.0	75.3	1.6	7.1	66.2	3.6	28.1	89.8
1988–1992	5.8	18.5	96.6	4.8	36.2	102.7	3.8	10.7	123.7	4.4	25.5	97.6
1993–1997	4.0	12.2	62.2	5.4	30.0	94.1	1.4	5.2	62.7	5.2	22.9	117.4
1998–2002	9.4	21.7	80.5	9.0	38.5	84.6	5.0	11.3	97.3	5.6	21.3	101.5
2003–2007	16.4	34.2	110.2	7.0	24.9	58.1	7.2	15.0	115.0	6.2	19.2	103.1
2008–2012	15.2	34.7	106.5	15.0	36.2	91.8	4.4	11.5	80.6	5.6	14.9	83.5
	35–44			>74 y/o			35–44			>74 y/o		
1983–1987	12.0	27.5	86.4	4.8	91.9	117.3	5.4	12.2	103.6	4.8	61.3	106.3
1988–1992	8.4	18.0	75.8	4.4	62.4	96.8	3.6	8.0	83.0	5.2	43.6	91.2
1993–1997	7.4	17.0	69.4	5.0	54.4	104.4	3.6	7.9	92.4	5.4	33.2	96.3
1998–2002	12.4	29.6	84.1	8.0	63.9	121.2	4.2	9.9	95.5	7.6	34.8	112.0
2003–2007	15.8	36.0	89.9	6.0	38.0	84.4	7.2	15.5	122.6	5.6	20.5	91.5
2008–2012	18.2	35.6	95.7	9.4	42.6	102.7	5.8	12.5	91.9	7.8	22.3	116.0

Secondary Medical Zone ID: 099	Male						Female					
	Suicide			Suicide			Suicide			Suicide		
	Num per year	Rate /100,000	×100	Num per year	Rate /100,000	×100	Num per year	Rate /100,000	×100	Num per year	Rate /100,000	×100
	Total (>10 y/o)			45-54			Total (>10 y/o)			45-54		
1983-1987	47.4	33.8	103.4	13.6	58.7	116.0	23.6	14.7	93.3	3.2	15.1	87.8
1988-1992	36.8	24.8	100.0	6.0	28.9	84.1	24.8	13.0	104.1	4.6	17.9	122.4
1993-1997	35.6	23.8	91.8	8.0	33.1	90.6	23.0	11.7	109.3	3.4	13.7	105.7
1998-2002	52.8	34.7	93.3	15.2	57.0	101.8	17.8	10.9	79.3	2.4	12.1	80.5
2003-2007	61.0	38.6	107.7	11.4	50.0	89.4	24.2	13.3	108.0	2.8	13.2	96.6
2008-2012	49.6	34.0	98.0	8.8	45.5	93.7	22.4	13.0	105.5	2.4	13.7	92.1
	15-24			55-64			15-24			55-64		
1983-1987	4.2	18.9	135.7	6.0	34.7	81.7	2.0	8.6	125.4	5.2	22.1	112.0
1988-1992	3.0	12.5	126.2	6.2	30.5	85.9	1.0	5.2	98.0	2.8	14.1	78.8
1993-1997	2.4	11.8	104.5	8.0	38.4	95.4	0.8	4.7	90.6	4.2	17.6	110.7
1998-2002	1.8	13.0	82.0	10.8	54.1	85.1	1.0	6.6	93.4	3.6	17.5	93.1
2003-2007	2.4	16.5	94.3	14.4	60.3	104.4	1.8	10.6	117.5	5.4	20.2	120.7
2008-2012	3.0	21.9	103.2	13.0	49.8	102.6	1.8	11.8	122.0	4.4	16.6	111.2
	25-34			65-74			25-34			65-74		
1983-1987	6.8	28.1	110.0	4.0	37.9	86.6	3.4	13.6	126.7	3.2	23.5	75.3
1988-1992	5.4	24.4	127.6	5.4	40.2	114.1	1.6	8.5	97.8	4.6	25.8	99.0
1993-1997	5.2	24.5	125.3	3.2	23.1	72.4	2.0	9.6	115.6	4.6	21.4	110.0
1998-2002	5.0	26.6	98.5	6.8	39.7	87.2	1.4	9.8	84.8	2.6	14.5	68.9
2003-2007	5.8	32.1	103.5	9.6	50.2	117.0	2.6	14.4	110.8	3.4	16.8	90.4
2008-2012	4.6	31.1	95.6	7.4	40.4	102.3	1.8	13.9	97.1	5.4	22.8	128.1
	35-44			>74 y/o			35-44			>74 y/o		
1983-1987	8.4	32.8	103.2	4.2	71.9	91.8	3.8	14.0	118.9	2.4	28.4	49.2
1988-1992	7.8	27.7	116.8	2.8	43.3	67.1	2.2	9.3	95.8	7.8	57.0	119.1
1993-1997	5.2	22.3	90.9	3.4	41.5	79.6	2.8	10.8	126.9	5.0	31.6	91.7
1998-2002	8.6	39.9	113.4	4.4	44.4	84.3	1.8	9.7	94.1	5.0	26.2	84.5
2003-2007	10.6	53.1	132.4	6.8	49.3	109.5	2.2	12.6	99.4	6.0	24.6	109.5
2008-2012	6.8	37.2	100.0	6.0	38.8	93.6	1.0	10.1	74.5	5.6	20.2	105.0

Secondary Medical Zone ID: 100

	Male						Female					
	Suicide			Suicide			Suicide			Suicide		
	Num per year	Rate /100,000	×100	Num per year	Rate /100,000	×100	Num per year	Rate /100,000	×100	Num per year	Rate /100,000	×100
	Total (>10 y/o)			45–54			Total (>10 y/o)			45–54		
1983–1987	44.8	28.4	82.9	8.8	37.1	73.4	28.4	14.5	93.6	3.2	14.1	82.0
1988–1992	42.0	23.0	90.5	9.6	34.0	99.0	26.4	11.7	90.8	3.4	13.4	91.3
1993–1997	56.2	26.3	105.0	14.2	39.5	107.8	20.8	9.2	78.8	4.6	13.6	105.0
1998–2002	78.0	34.6	95.6	20.2	53.6	95.7	30.4	11.8	92.6	4.2	13.0	86.1
2003–2007	92.4	39.3	109.8	18.2	54.2	96.9	29.8	12.9	94.4	3.8	12.5	91.3
2008–2012	84.2	37.7	108.3	17.2	57.5	118.4	29.6	12.3	96.0	4.4	15.3	102.8
	15–24			55–64			15–24			55–64		
1983–1987	3.8	15.4	110.8	7.2	34.5	81.2	1.2	6.1	89.8	5.0	19.1	96.9
1988–1992	3.0	10.4	104.9	9.0	34.8	98.0	0.4	3.3	61.8	5.4	18.6	104.2
1993–1997	3.4	11.3	99.9	10.6	39.2	97.3	1.8	6.0	114.4	3.2	12.5	78.6
1998–2002	4.4	15.4	97.4	18.0	60.0	94.4	1.4	6.1	87.1	6.2	19.6	104.3
2003–2007	4.8	17.7	101.2	23.8	64.4	111.6	2.8	10.7	119.2	3.6	12.4	74.3
2008–2012	6.0	24.3	114.5	16.8	44.1	90.7	1.0	6.8	70.2	4.8	13.5	90.6
	25–34			65–74			25–34			65–74		
1983–1987	7.0	26.8	105.0	5.6	40.1	91.6	3.6	13.3	123.6	6.6	33.8	108.1
1988–1992	4.6	19.3	100.8	4.6	29.2	83.0	2.0	8.7	100.4	5.0	22.9	87.6
1993–1997	6.0	22.6	115.8	5.2	25.9	81.3	1.0	6.0	72.1	4.2	16.5	84.6
1998–2002	8.0	28.9	106.8	10.2	42.0	92.4	1.8	9.0	77.9	5.6	19.7	93.7
2003–2007	9.0	33.5	108.2	12.0	44.6	104.0	3.4	13.6	104.7	5.2	17.9	96.1
2008–2012	8.4	35.7	109.5	12.8	42.6	107.9	2.0	11.7	81.9	5.4	17.6	99.0
	35–44			>74 y/o			35–44			>74 y/o		
1983–1987	8.6	28.1	88.3	3.6	51.7	66.0	2.4	9.6	81.1	6.4	51.4	89.1
1988–1992	6.8	20.5	86.6	4.4	47.7	73.9	2.2	8.0	82.8	8.0	47.8	100.0
1993–1997	10.0	29.4	119.7	6.8	55.4	106.4	1.4	6.0	70.0	4.6	23.2	67.4
1998–2002	10.0	34.4	97.7	7.0	47.9	90.9	3.4	11.3	109.6	7.6	29.0	93.5
2003–2007	14.0	49.4	123.2	10.4	52.8	117.3	5.2	16.7	132.0	5.6	17.7	79.0
2008–2012	13.2	44.1	118.5	9.4	41.1	99.1	4.6	15.7	115.9	7.2	19.3	100.3

Secondary Medical Zone ID: 101

	Male						Female					
	Suicide			Suicide			Suicide			Suicide		
	Num per year	Rate /100,000	×100	Num per year	Rate /100,000	×100	Num per year	Rate /100,000	×100	Num per year	Rate /100,000	×100
	Total (>10 y/o)			45–54			Total (>10 y/o)			45–54		
1983–1987	25.8	34.7	110.2	4.6	45.1	89.3	17.2	17.1	117.3	3.6	23.6	137.2
1988–1992	19.8	25.5	106.3	6.2	48.9	142.4	15.8	14.0	118.2	2.0	15.9	108.5
1993–1997	21.4	26.5	110.7	5.0	39.9	109.0	11.2	11.1	102.4	2.2	15.1	117.0
1998–2002	28.4	37.4	104.8	6.8	57.9	103.5	9.2	11.7	83.2	2.2	16.7	110.7
2003–2007	24.0	35.7	93.6	5.0	54.1	96.8	8.4	11.8	85.6	0.8	12.0	87.8
2008–2012	20.0	33.2	90.0	4.2	52.0	106.9	9.8	13.0	98.2	1.4	16.2	108.4
	15–24			55–64			15–24			55–64		
1983–1987	1.6	17.3	123.7	6.2	53.7	126.4	1.0	8.5	124.2	2.2	18.8	95.2
1988–1992	1.4	12.0	120.8	2.6	27.9	78.5	0.4	5.2	98.7	4.0	25.0	139.9
1993–1997	1.2	12.6	111.0	5.0	44.6	110.9	0.2	4.6	87.3	2.0	16.7	105.0
1998–2002	2.0	20.4	128.8	8.0	71.9	113.2	0.0	5.4	77.3	1.2	15.6	82.9
2003–2007	1.8	21.9	125.4	4.8	46.2	80.0	0.6	9.7	107.5	1.2	14.1	84.5
2008–2012	1.0	21.7	102.5	3.8	39.3	80.9	0.6	10.7	109.8	1.4	13.7	92.0
	25–34			65–74			25–34			65–74		
1983–1987	2.8	29.0	113.3	3.6	48.5	110.7	2.0	15.2	141.1	3.4	33.8	108.3
1988–1992	0.8	15.4	80.4	2.2	30.9	87.6	1.0	10.1	116.5	2.6	24.8	95.2
1993–1997	2.0	23.3	119.1	3.4	35.4	111.1	1.4	11.4	137.0	2.4	20.2	103.7
1998–2002	1.6	25.2	93.3	4.0	42.6	93.6	0.6	10.8	93.5	1.8	17.9	85.2
2003–2007	2.8	34.9	112.4	4.2	43.0	100.1	0.8	12.7	97.7	2.0	18.3	98.2
2008–2012	1.4	29.8	91.5	5.0	45.0	113.9	1.0	15.1	105.3	1.8	17.2	96.8
	35–44			>74 y/o			35–44			>74 y/o		
1983–1987	3.2	28.6	89.7	3.8	89.5	114.3	1.2	11.1	94.3	3.8	58.8	101.9
1988–1992	1.6	18.0	76.1	4.6	84.3	130.7	0.8	8.8	90.9	5.0	57.5	120.1
1993–1997	2.4	25.0	102.1	2.4	44.8	86.0	0.4	7.1	82.9	2.6	27.5	79.9
1998–2002	3.6	41.2	117.1	2.4	40.8	77.4	1.0	11.2	108.4	2.4	21.9	70.6
2003–2007	2.6	37.9	94.7	2.8	37.5	83.2	1.0	13.1	103.5	2.0	15.9	70.7
2008–2012	1.8	32.0	85.9	2.4	31.3	75.4	0.8	12.9	95.1	2.8	18.2	94.9

Secondary Medical Zone ID: 102

	Male						Female					
	Suicide			Suicide			Suicide			Suicide		
	Num per year	Rate /100,000	×100	Num per year	Rate /100,000	×100	Num per year	Rate /100,000	×100	Num per year	Rate /100,000	×100
	Total (>10 y/o)			45–54			Total (>10 y/o)			45–54		
1983–1987	40.0	32.3	99.3	10.0	48.2	95.3	22.6	16.4	111.2	3.6	18.2	105.6
1988–1992	36.2	25.4	105.8	9.2	36.3	105.7	22.2	13.8	112.9	2.8	13.4	91.5
1993–1997	42.2	26.6	109.0	11.8	40.5	110.7	21.2	11.8	115.5	4.4	15.1	116.6
1998–2002	64.4	38.9	110.3	17.8	63.6	113.7	26.0	14.2	115.9	4.4	16.2	107.5
2003–2007	67.4	40.4	113.8	16.0	66.5	118.9	21.0	12.5	99.5	2.8	13.3	97.0
2008–2012	54.6	33.7	98.2	9.8	47.5	97.8	18.4	12.0	90.3	2.2	13.2	88.7
	15–24			55–64			15–24			55–64		
1983–1987	3.4	16.2	115.8	4.0	33.6	79.1	2.0	8.5	123.9	3.4	21.6	109.6
1988–1992	2.4	9.9	100.3	7.8	42.1	118.6	2.8	8.9	169.3	2.8	16.9	94.7
1993–1997	1.2	7.7	68.4	9.8	46.1	114.5	2.2	7.6	144.9	3.2	16.3	102.7
1998–2002	3.6	16.6	105.4	19.6	79.9	125.7	1.6	7.7	109.0	5.2	21.3	113.4
2003–2007	3.8	19.4	111.4	15.6	59.0	102.2	1.0	7.5	82.9	4.8	17.8	106.3
2008–2012	4.8	25.3	119.4	11.0	43.1	88.7	0.8	7.5	77.6	3.2	13.5	90.7
	25–34			65–74			25–34			65–74		
1983–1987	4.8	22.8	89.3	3.6	47.1	107.5	2.0	10.3	95.7	3.6	33.6	107.5
1988–1992	5.0	23.1	120.8	3.4	36.9	104.9	2.8	11.9	137.3	3.8	29.8	114.2
1993–1997	4.4	20.5	105.1	5.2	39.8	124.9	0.8	6.3	75.3	4.4	27.0	138.6
1998–2002	5.2	24.2	89.8	5.0	36.9	81.2	3.8	15.0	129.5	2.2	16.1	76.6
2003–2007	8.6	36.7	118.3	8.0	44.9	104.6	2.4	12.6	96.7	1.8	13.2	71.1
2008–2012	5.2	29.0	88.9	7.8	38.0	96.2	1.4	11.3	78.8	3.8	17.8	100.1
	35–44			>74 y/o			35–44			>74 y/o		
1983–1987	11.0	37.8	118.6	3.2	76.4	97.5	3.6	12.8	108.2	4.4	65.5	113.5
1988–1992	4.8	19.0	80.3	3.2	62.0	96.1	2.0	8.6	89.1	5.2	55.9	116.9
1993–1997	6.4	26.2	106.8	3.4	54.6	104.8	1.6	7.8	91.8	4.2	36.8	106.8
1998–2002	10.4	46.3	131.4	2.4	37.3	70.7	3.2	13.7	132.2	5.4	37.1	119.4
2003–2007	9.4	44.2	110.3	6.0	54.4	120.7	3.2	14.7	116.1	5.0	27.5	122.5
2008–2012	8.4	35.9	96.6	7.6	53.4	128.8	3.4	14.8	109.4	3.4	17.0	88.6

Secondary Medical Zone ID: 103

	Male						Female					
	Suicide			Suicide			Suicide			Suicide		
	Num per year	Rate /100,000	×100	Num per year	Rate /100,000	×100	Num per year	Rate /100,000	×100	Num per year	Rate /100,000	×100
	Total (>10 y/o)			45-54			Total (>10 y/o)			45-54		
1983-1987	28.6	31.0	93.6	8.4	50.9	100.6	14.4	15.9	103.9	1.6	14.4	83.8
1988-1992	28.4	25.5	105.1	7.0	33.8	98.4	12.2	12.0	91.7	2.4	14.0	95.8
1993-1997	25.2	21.6	81.7	7.6	32.7	89.4	11.8	10.1	91.0	2.0	10.9	84.3
1998-2002	48.4	35.0	98.0	13.0	54.4	97.1	13.0	11.2	82.3	3.8	16.3	108.0
2003-2007	43.8	32.8	86.6	9.2	46.1	82.5	16.6	11.8	97.7	2.2	12.7	92.5
2008-2012	48.4	34.7	101.5	8.6	48.9	100.6	18.8	13.3	105.8	2.0	13.6	91.4
	15-24			55-64			15-24			55-64		
1983-1987	2.8	15.8	113.4	4.2	44.2	104.1	1.0	6.7	98.8	1.8	19.6	99.1
1988-1992	3.0	12.1	121.9	4.6	36.0	101.3	0.4	3.8	71.7	2.4	19.0	106.5
1993-1997	3.0	12.2	108.3	5.2	34.5	85.8	1.2	5.6	107.0	1.4	12.6	79.0
1998-2002	2.8	14.8	94.0	12.4	62.3	98.1	1.6	8.0	113.3	1.8	14.3	76.1
2003-2007	2.4	15.7	89.8	13.2	57.2	99.1	0.8	7.1	78.4	4.2	18.2	108.8
2008-2012	3.2	21.3	100.5	10.8	47.1	96.9	1.2	9.3	96.4	2.4	13.0	87.0
	25-34			65-74			25-34			65-74		
1983-1987	3.4	20.2	78.9	1.2	35.2	80.3	1.8	10.9	100.8	2.0	32.2	102.9
1988-1992	4.2	22.2	116.4	0.8	23.7	67.1	0.4	5.8	67.3	2.6	31.1	119.2
1993-1997	2.4	14.8	75.7	1.8	25.7	80.6	1.8	9.4	112.9	1.4	16.9	86.9
1998-2002	5.8	27.0	99.9	5.8	49.7	109.4	1.0	8.6	74.4	2.4	21.0	99.8
2003-2007	5.6	28.5	91.9	4.0	32.4	75.6	1.2	9.8	75.6	2.6	18.7	100.5
2008-2012	4.8	29.6	90.9	6.0	35.7	90.4	2.0	13.9	96.7	3.2	18.3	103.1
	35-44			>74 y/o			35-44			>74 y/o		
1983-1987	7.0	30.3	95.1	1.6	72.9	93.1	2.6	11.9	100.6	3.6	84.1	145.8
1988-1992	4.8	21.2	89.5	4.0	102.0	158.1	1.6	8.5	88.3	2.4	46.3	96.8
1993-1997	3.4	18.2	74.3	1.6	46.0	88.4	1.0	6.8	79.8	2.8	39.7	115.4
1998-2002	5.6	31.6	89.6	3.0	57.0	108.0	0.8	7.5	73.0	1.6	21.6	69.7
2003-2007	6.6	36.2	90.3	2.8	41.5	92.1	1.6	11.1	88.2	4.0	32.1	143.1
2008-2012	9.8	43.3	116.5	5.2	50.9	122.7	3.6	16.4	121.2	4.2	26.4	137.2

Secondary Medical Zone ID: 104

	Male						Female					
	Suicide			Suicide			Suicide			Suicide		
	Num per year	Rate /100,000	×100	Num per year	Rate /100,000	×100	Num per year	Rate /100,000	×100	Num per year	Rate /100,000	×100
	Total (>10 y/o)			45–54			Total (>10 y/o)			45–54		
1983–1987	79.0	27.8	82.8	20.2	45.4	89.8	50.6	14.9	94.6	8.6	17.3	100.5
1988–1992	62.2	23.1	91.4	12.8	30.3	88.1	42.4	12.9	97.6	5.4	12.6	86.4
1993–1997	68.6	25.1	99.2	17.6	38.6	105.6	35.4	11.2	97.5	6.0	13.1	101.6
1998–2002	108.6	36.0	102.1	25.2	57.0	101.8	51.0	15.0	114.6	9.0	19.1	127.2
2003–2007	110.6	34.0	93.2	19.8	47.4	84.7	59.0	15.9	125.3	6.0	14.5	105.5
2008–2012	113.2	32.6	95.0	16.8	36.7	75.4	56.2	14.7	111.8	9.6	19.4	129.8
	15–24			55–64			15–24			55–64		
1983–1987	5.8	10.7	76.5	13.0	39.5	92.9	4.0	7.4	108.7	6.4	15.8	80.2
1988–1992	5.6	10.9	109.7	10.2	29.7	83.5	4.4	8.1	153.8	7.6	18.3	102.4
1993–1997	5.0	11.7	103.2	18.2	47.8	118.7	2.2	5.5	104.6	5.0	13.1	82.4
1998–2002	6.0	16.3	103.3	28.2	69.9	110.1	4.0	10.0	141.7	7.0	17.3	92.0
2003–2007	5.2	16.2	92.9	32.2	69.5	120.4	6.6	17.2	190.8	8.0	17.3	103.5
2008–2012	5.6	19.4	91.7	24.6	53.7	110.6	3.6	11.5	118.9	9.4	19.4	130.2
	25–34			65–74			25–34			65–74		
1983–1987	12.4	24.7	96.7	8.4	39.1	89.2	8.6	16.8	156.3	5.8	20.5	65.7
1988–1992	9.8	22.7	118.9	6.8	32.8	93.2	5.6	12.7	146.1	4.8	17.1	65.4
1993–1997	9.2	21.8	111.8	5.6	24.7	77.6	5.0	11.3	136.0	5.6	17.4	89.5
1998–2002	16.0	31.4	116.3	15.4	52.4	115.2	9.8	17.8	153.3	7.4	21.0	99.8
2003–2007	12.4	22.7	73.3	14.2	44.7	104.1	13.6	20.9	160.9	7.8	20.4	109.5
2008–2012	20.6	34.4	105.7	17.2	49.6	125.4	11.4	17.8	124.3	7.8	19.5	109.8
	35–44			>74 y/o			35–44			>74 y/o		
1983–1987	14.4	27.2	85.5	4.8	41.2	52.6	10.0	16.4	139.2	7.0	36.7	63.6
1988–1992	11.0	24.6	103.8	5.6	42.3	65.5	6.0	12.2	126.5	8.6	38.2	79.9
1993–1997	8.8	24.5	99.9	4.0	29.9	57.5	5.4	12.9	151.7	6.2	24.5	71.2
1998–2002	11.6	29.7	84.3	6.2	38.4	72.9	7.0	15.4	149.4	6.8	22.6	72.7
2003–2007	20.2	38.1	95.1	6.4	32.5	72.1	9.6	16.6	131.1	7.4	20.2	90.0
2008–2012	20.4	32.2	86.4	7.8	33.1	79.8	8.4	12.6	92.8	5.8	14.4	74.9

Secondary Medical Zone ID: 105

	Male						Female					
	Suicide			Suicide			Suicide			Suicide		
	Num per year	Rate /100,000	×100	Num per year	Rate /100,000	×100	Num per year	Rate /100,000	×100	Num per year	Rate /100,000	×100
	Total (>10 y/o)			45-54			Total (>10 y/o)			45-54		
1983-1987	112.6	27.0	80.7	24.6	38.5	76.1	66.6	14.6	95.9	13.0	18.4	106.6
1988-1992	89.4	20.6	82.0	22.2	31.0	90.4	53.8	11.4	87.3	8.8	12.9	87.9
1993-1997	113.0	24.3	97.2	28.6	36.1	98.6	53.2	10.9	96.7	10.6	14.0	108.7
1998-2002	158.2	32.6	90.8	38.6	51.8	92.5	58.4	12.2	88.7	9.6	14.2	94.5
2003-2007	151.8	30.1	82.8	24.0	37.4	66.9	66.2	13.2	99.8	8.8	14.5	105.9
2008-2012	152.4	30.1	87.0	28.6	42.4	87.3	69.6	13.4	101.1	11.6	18.3	122.6
	15-24			55-64			15-24			55-64		
1983-1987	9.2	10.6	76.2	17.0	37.5	88.3	6.6	8.2	119.7	11.4	20.4	103.4
1988-1992	7.0	8.5	86.1	16.8	31.7	89.4	4.0	5.2	99.2	9.6	16.2	91.0
1993-1997	10.2	13.4	118.1	21.8	37.4	93.0	4.6	6.3	121.4	11.4	17.8	111.9
1998-2002	7.4	12.8	80.8	43.4	66.3	104.3	5.8	9.1	130.2	9.8	15.8	83.7
2003-2007	7.4	14.5	83.3	44.6	61.0	105.7	6.0	11.2	124.8	10.4	15.3	91.6
2008-2012	9.0	19.2	90.7	29.4	42.8	88.2	3.4	7.9	81.8	9.4	14.4	96.7
	25-34			65-74			25-34			65-74		
1983-1987	16.8	19.6	76.5	9.8	37.2	85.0	4.0	6.3	58.6	9.8	28.3	90.4
1988-1992	10.8	13.7	71.8	6.6	24.0	68.0	7.0	9.8	113.6	8.8	22.3	85.4
1993-1997	14.6	17.8	91.2	13.0	35.6	111.5	8.8	11.4	137.3	7.2	15.9	81.7
1998-2002	20.0	22.9	84.7	17.8	42.4	93.2	8.6	10.9	93.7	7.6	15.5	73.7
2003-2007	19.6	22.2	71.5	18.4	39.5	92.0	13.0	15.1	116.2	8.0	15.1	81.0
2008-2012	22.4	27.1	83.3	23.6	44.6	112.8	12.0	15.0	104.9	10.8	18.2	102.3
	35-44			>74 y/o			35-44			>74 y/o		
1983-1987	26.2	29.7	93.2	8.4	62.3	79.5	11.0	13.2	112.2	10.2	48.4	83.9
1988-1992	16.8	21.1	89.1	8.4	50.8	78.7	7.2	9.8	102.0	8.4	31.9	66.7
1993-1997	15.8	23.7	96.7	9.0	45.9	88.2	5.0	8.3	98.0	5.4	17.4	50.6
1998-2002	21.0	31.2	88.5	9.8	41.5	78.8	9.0	13.9	134.3	7.8	19.8	63.6
2003-2007	26.2	32.9	82.2	11.6	39.3	87.2	11.2	15.0	118.8	8.8	17.8	79.5
2008-2012	27.4	30.7	82.4	11.6	33.5	80.8	13.2	15.1	111.0	8.8	15.1	78.4

Secondary Medical Zone ID: 106

	Male						Female					
	Suicide			Suicide			Suicide			Suicide		
	Num per year	Rate /100,000	×100	Num per year	Rate /100,000	×100	Num per year	Rate /100,000	×100	Num per year	Rate /100,000	×100
	Total (>10 y/o)			45–54			Total (>10 y/o)			45–54		
1983–1987	122.8	23.4	70.5	26.2	35.4	70.0	80.2	13.6	88.2	11.2	13.5	78.4
1988–1992	108.6	20.7	82.6	25.6	33.2	96.8	69.8	11.5	87.9	13.4	15.4	105.5
1993–1997	114.8	21.1	83.4	25.2	30.7	83.8	62.8	10.0	88.9	11.0	12.4	96.3
1998–2002	172.0	30.6	84.0	36.2	45.4	81.1	81.6	12.3	94.7	9.4	11.9	79.3
2003–2007	160.2	28.0	74.5	31.6	42.0	75.1	81.6	12.3	93.7	10.4	13.3	97.4
2008–2012	157.4	26.8	75.3	31.8	37.8	77.8	94.0	14.0	101.8	15.4	17.1	114.9
	15–24			55–64			15–24			55–64		
1983–1987	19.0	13.5	96.8	16.0	30.4	71.6	8.2	7.1	104.4	14.0	20.3	102.8
1988–1992	11.0	9.1	92.1	16.0	26.8	75.5	5.6	5.1	97.2	12.0	16.7	93.4
1993–1997	11.8	11.5	101.3	23.0	35.9	89.3	6.2	6.1	116.6	10.0	13.7	86.4
1998–2002	12.4	14.5	91.5	38.6	57.3	90.2	9.4	10.3	146.2	13.6	18.0	95.5
2003–2007	11.8	16.6	95.2	34.6	47.2	81.8	6.8	9.6	106.2	12.6	15.8	94.1
2008–2012	9.8	16.7	78.9	31.4	43.3	89.1	9.0	13.6	140.6	15.0	18.2	122.0
	25–34			65–74			25–34			65–74		
1983–1987	19.6	17.6	68.8	8.8	28.8	65.7	12.2	11.4	105.7	9.8	23.6	75.4
1988–1992	19.2	17.8	93.2	7.2	22.1	62.6	8.2	8.1	93.4	11.6	23.9	91.7
1993–1997	18.8	17.2	88.1	12.8	30.0	94.2	10.0	9.0	108.2	9.2	16.5	84.4
1998–2002	28.8	25.6	94.9	20.2	40.5	89.1	16.8	13.8	119.1	11.2	18.0	85.5
2003–2007	27.4	24.6	79.4	17.8	34.7	80.9	17.0	13.8	106.2	11.2	17.1	92.0
2008–2012	24.6	23.5	72.0	18.2	33.8	85.5	21.2	17.5	122.4	8.8	13.8	77.4
	35–44			>74 y/o			35–44			>74 y/o		
1983–1987	25.0	25.5	80.2	7.8	43.9	56.0	14.2	13.4	113.8	10.4	37.0	64.1
1988–1992	20.4	22.7	95.9	8.8	40.8	63.3	8.2	9.1	94.0	10.6	30.4	63.6
1993–1997	13.8	17.8	72.6	9.4	37.6	72.3	7.4	9.0	105.7	8.8	21.3	61.9
1998–2002	24.8	28.8	81.8	10.8	34.9	66.3	10.2	11.3	109.1	11.0	21.3	68.5
2003–2007	25.8	25.9	64.7	11.0	29.5	65.6	13.6	12.8	101.3	9.6	15.2	67.5
2008–2012	29.2	26.9	72.2	12.0	28.1	67.8	15.6	12.8	94.4	8.8	11.9	62.1

Secondary Medical Zone ID: 107

	Male						Female					
	Suicide			Suicide			Suicide			Suicide		
	Num per year	Rate /100,000	×100	Num per year	Rate /100,000	×100	Num per year	Rate /100,000	×100	Num per year	Rate /100,000	×100
	Total (>10 y/o)			45–54			Total (>10 y/o)			45–54		
1983–1987	132.4	27.0	81.7	27.4	40.3	79.7	72.4	13.3	85.8	13.0	16.3	94.7
1988–1992	95.2	20.0	78.7	20.8	29.8	86.7	65.2	11.6	88.9	10.4	13.6	92.8
1993–1997	112.8	22.5	89.2	27.2	35.2	96.2	62.6	11.0	97.5	12.2	14.9	115.1
1998–2002	174.4	33.3	91.9	40.4	54.3	97.0	78.4	13.6	101.7	13.4	17.6	117.2
2003–2007	167.6	31.1	84.7	26.2	39.6	70.8	84.8	14.6	110.2	8.6	13.2	96.5
2008–2012	171.8	33.7	96.6	25.4	38.9	80.0	88.6	16.0	118.6	11.6	17.2	115.5
	15–24			55–64			15–24			55–64		
1983–1987	16.0	12.3	88.0	17.6	35.4	83.4	8.4	7.7	112.8	10.0	16.1	81.4
1988–1992	9.8	8.9	90.0	16.2	29.3	82.6	8.2	7.7	146.0	11.8	17.4	97.7
1993–1997	13.4	13.7	121.4	21.8	36.9	91.6	6.2	6.7	128.2	10.0	14.8	93.0
1998–2002	12.8	15.4	97.4	41.6	65.9	103.8	7.2	8.8	125.3	13.8	19.9	105.5
2003–2007	14.4	20.2	116.0	29.8	43.7	75.7	11.4	15.8	175.2	14.0	18.8	112.1
2008–2012	14.4	25.9	122.0	30.4	46.3	95.4	8.0	13.9	143.7	13.6	19.0	127.6
	25–34			65–74			25–34			65–74		
1983–1987	25.0	23.1	90.5	9.8	33.0	75.3	8.0	8.8	82.1	11.4	27.7	88.5
1988–1992	15.0	15.3	80.2	6.2	20.7	58.9	8.2	9.2	105.8	6.2	14.5	55.7
1993–1997	19.4	19.5	100.0	9.4	24.5	76.7	10.4	10.9	131.1	7.2	14.0	72.1
1998–2002	28.4	25.8	95.6	17.4	38.5	84.8	14.0	13.5	116.1	8.4	15.1	71.7
2003–2007	34.0	30.9	99.8	21.4	43.0	100.3	20.0	18.5	142.6	8.6	14.7	79.2
2008–2012	36.4	38.6	118.7	17.8	35.3	89.3	20.0	21.8	152.2	9.8	16.3	91.5
	35–44			>74 y/o			35–44			>74 y/o		
1983–1987	27.2	30.2	94.9	8.8	50.5	64.5	12.0	12.8	108.2	9.0	33.9	58.8
1988–1992	19.4	23.6	99.5	7.4	37.1	57.5	9.2	11.0	114.4	11.0	32.7	68.3
1993–1997	14.6	20.7	84.6	7.0	31.0	59.6	7.0	9.9	116.3	9.4	23.7	68.8
1998–2002	23.4	31.1	88.2	10.0	35.8	67.9	11.8	15.4	148.8	9.8	20.4	65.7
2003–2007	28.8	33.8	84.3	12.8	36.0	79.9	14.2	16.5	130.9	8.0	13.8	61.3
2008–2012	34.8	39.7	106.8	12.4	31.2	75.2	16.6	18.5	136.7	9.0	13.5	70.2

Secondary Medical Zone ID: 108

	Male						Female					
	Suicide			Suicide			Suicide			Suicide		
	Num per year	Rate /100,000	×100	Num per year	Rate /100,000	×100	Num per year	Rate /100,000	×100	Num per year	Rate /100,000	×100
	Total (>10 y/o)			45–54			Total (>10 y/o)			45–54		
1983–1987	204.2	28.5	87.2	51.6	45.4	89.7	109.2	14.4	94.9	17.8	14.7	85.4
1988–1992	166.8	21.9	88.9	39.0	32.3	93.9	95.2	11.6	89.7	17.8	14.2	96.9
1993–1997	198.6	24.2	97.8	51.6	39.1	106.8	91.6	10.6	95.0	14.8	11.7	90.3
1998–2002	275.2	32.5	90.7	67.2	53.8	96.1	127.4	14.0	108.1	21.0	17.4	115.7
2003–2007	287.4	34.3	93.2	56.2	51.4	91.8	124.2	14.3	107.3	15.8	15.1	109.9
2008–2012	281.6	32.8	94.0	56.6	49.7	102.3	135.2	14.8	111.7	18.8	16.9	113.4
	15–24			55–64			15–24			55–64		
1983–1987	17.4	11.0	78.8	30.8	39.7	93.4	11.6	8.1	118.2	15.2	17.1	86.8
1988–1992	15.4	9.8	98.9	30.4	32.0	90.2	7.4	5.2	98.3	15.2	14.8	83.0
1993–1997	17.4	12.5	110.8	43.4	41.2	102.3	11.0	8.1	154.8	15.8	14.1	88.5
1998–2002	17.0	14.7	93.0	72.0	64.6	101.7	9.6	8.5	120.4	22.0	18.7	99.3
2003–2007	13.4	14.4	82.4	64.6	53.8	93.2	12.0	12.4	138.2	22.4	18.2	108.6
2008–2012	21.4	24.0	113.0	57.6	49.7	102.3	11.4	12.6	129.4	20.2	17.6	117.7
	25–34			65–74			25–34			65–74		
1983–1987	33.4	23.0	89.9	12.4	30.8	70.3	15.4	12.2	113.2	14.6	26.9	86.1
1988–1992	24.4	17.3	90.7	14.4	29.5	83.6	10.0	8.2	94.1	16.6	25.2	96.6
1993–1997	29.0	19.8	101.2	21.2	32.6	102.2	14.2	10.5	126.7	14.2	17.8	91.5
1998–2002	37.0	24.5	90.6	30.6	38.4	84.4	22.8	15.8	136.2	20.0	21.0	99.8
2003–2007	46.4	33.0	106.4	35.2	39.7	92.4	21.4	16.0	122.7	20.8	19.7	105.8
2008–2012	36.4	25.8	79.2	38.4	42.5	107.5	24.2	18.0	125.7	17.0	16.3	91.5
	35–44			>74 y/o			35–44			>74 y/o		
1983–1987	45.4	31.6	99.2	12.6	60.3	77.0	19.0	13.5	114.0	15.0	46.8	81.1
1988–1992	29.4	21.8	91.8	13.6	50.2	77.8	12.4	9.9	102.5	15.2	35.1	73.3
1993–1997	25.8	22.0	89.6	10.0	31.7	60.8	11.0	10.1	118.3	10.6	19.9	57.7
1998–2002	34.4	29.3	83.3	16.0	37.4	71.0	14.6	13.2	127.2	17.0	24.4	78.4
2003–2007	50.8	39.5	98.5	20.0	35.1	77.9	20.4	16.5	130.8	11.2	12.8	56.8
2008–2012	46.8	30.9	83.1	23.4	35.2	84.8	23.4	16.2	119.4	20.2	18.3	95.2

Secondary Medical Zone ID: 109

	Male						Female					
	Suicide			Suicide			Suicide			Suicide		
	Num per year	Rate /100,000	×100	Num per year	Rate /100,000	×100	Num per year	Rate /100,000	×100	Num per year	Rate /100,000	×100
	Total (>10 y/o)			45-54			Total (>10 y/o)			45-54		
1983-1987	157.8	30.9	94.2	47.6	51.2	101.3	82.8	15.9	103.6	14.0	15.6	90.4
1988-1992	131.8	24.1	97.4	29.2	30.7	89.3	71.2	12.3	96.5	10.2	11.4	78.0
1993-1997	153.4	25.7	104.8	42.4	42.5	116.2	71.2	11.5	105.7	13.0	13.7	106.0
1998-2002	229.8	37.6	105.8	55.0	60.2	107.6	85.2	13.6	105.1	11.0	13.6	90.6
2003-2007	218.2	34.6	97.8	43.2	54.2	96.8	84.6	13.7	105.3	10.8	14.9	108.9
2008-2012	211.4	32.3	96.5	35.2	42.3	87.1	101.4	15.2	118.6	15.8	20.3	136.3
	15-24			55-64			15-24			55-64		
1983-1987	11.6	11.4	81.9	26.2	46.4	109.1	7.6	7.6	111.9	13.6	21.9	111.0
1988-1992	10.2	9.8	98.9	27.8	37.5	105.6	7.4	7.2	135.9	12.0	16.5	92.4
1993-1997	9.6	11.0	97.7	40.8	47.7	118.6	5.8	6.6	127.1	12.8	15.2	95.6
1998-2002	12.4	17.4	110.3	57.8	65.6	103.3	4.6	7.0	99.6	17.2	19.1	101.7
2003-2007	7.0	12.6	72.0	59.2	63.7	110.4	6.6	10.9	121.3	12.6	14.5	86.6
2008-2012	12.4	20.6	97.0	47.0	51.9	106.8	7.6	12.6	129.5	17.8	20.2	135.3
	25-34			65-74			25-34			65-74		
1983-1987	19.4	21.5	84.2	8.4	30.3	69.2	10.0	12.3	114.3	11.4	28.9	92.5
1988-1992	15.8	17.1	89.4	11.2	32.6	92.5	7.6	9.2	106.6	9.8	21.8	83.7
1993-1997	19.2	19.4	99.2	13.8	30.4	95.2	9.6	10.4	124.8	10.0	18.5	94.7
1998-2002	28.4	27.8	103.0	29.0	47.9	105.3	12.6	13.3	114.9	12.8	19.5	92.8
2003-2007	22.8	24.4	78.8	36.2	50.9	118.7	15.2	16.8	129.1	14.4	18.5	99.5
2008-2012	21.8	25.3	77.8	36.0	47.0	118.9	12.6	15.3	106.8	17.2	19.8	111.5
	35-44			>74 y/o			35-44			>74 y/o		
1983-1987	31.6	30.0	94.3	12.8	86.5	110.4	10.0	10.4	87.6	15.8	72.4	125.6
1988-1992	22.6	23.3	98.5	14.6	77.8	120.6	9.4	10.6	110.0	14.2	48.4	101.2
1993-1997	17.4	21.3	86.8	9.8	46.0	88.3	7.4	9.9	116.2	12.2	32.8	95.4
1998-2002	32.4	38.7	110.0	14.4	53.0	100.6	12.8	16.2	156.8	13.8	29.6	95.2
2003-2007	32.8	34.9	87.0	16.8	45.8	101.7	13.0	15.0	118.6	11.8	19.7	87.9
2008-2012	38.8	35.1	94.2	20.0	40.3	97.2	15.2	15.0	111.0	15.0	19.2	99.9

Secondary Medical Zone ID: 110

	Male						Female					
	Suicide			Suicide			Suicide			Suicide		
	Num per year	Rate /100,000	×100	Num per year	Rate /100,000	×100	Num per year	Rate /100,000	×100	Num per year	Rate /100,000	×100
	Total (>10 y/o)			45-54			Total (>10 y/o)			45-54		
1983-1987	148.0	32.0	96.9	41.0	49.3	97.5	67.4	14.5	94.8	11.6	15.0	86.9
1988-1992	113.2	22.5	90.3	30.6	33.3	96.9	67.6	12.8	100.3	12.2	13.9	95.2
1993-1997	146.6	25.9	105.1	37.8	38.1	104.1	57.2	10.2	92.2	14.2	14.8	114.8
1998-2002	218.8	35.5	101.6	51.8	55.5	99.2	84.8	13.7	107.8	14.6	16.6	110.4
2003-2007	212.2	32.8	91.5	36.2	44.0	78.6	83.8	13.1	102.6	10.2	13.6	99.4
2008-2012	218.0	32.8	97.0	36.8	42.6	87.6	99.0	14.6	114.1	14.0	17.8	119.0
	15-24			55-64			15-24			55-64		
1983-1987	11.8	12.7	91.2	20.4	42.8	100.7	5.4	6.4	94.1	10.0	19.1	96.7
1988-1992	8.8	9.0	90.5	19.0	30.3	85.4	5.6	6.0	113.6	8.2	13.6	76.3
1993-1997	11.8	12.8	113.1	33.6	43.8	108.7	5.4	6.3	120.6	10.4	14.1	88.6
1998-2002	10.8	14.6	92.5	66.8	77.6	122.2	6.2	8.5	121.6	17.8	20.7	110.2
2003-2007	11.2	16.9	96.8	58.2	62.2	107.8	5.4	8.7	97.0	17.2	18.7	111.4
2008-2012	10.0	17.1	80.5	48.0	53.3	109.8	7.2	11.8	121.2	18.6	20.6	138.3
	25-34			65-74			25-34			65-74		
1983-1987	18.8	21.8	85.5	12.8	50.0	114.3	9.6	11.9	111.0	7.8	24.0	76.8
1988-1992	14.8	16.8	87.8	8.6	30.0	85.2	8.0	9.8	113.4	8.4	21.6	82.6
1993-1997	23.6	23.5	120.3	11.6	30.1	94.3	6.6	7.7	93.1	7.8	16.8	86.0
1998-2002	24.6	22.7	84.1	27.2	51.5	113.2	14.2	13.8	119.3	12.8	21.7	103.5
2003-2007	29.6	26.7	85.9	29.8	46.6	108.7	14.6	14.0	107.8	11.6	16.8	90.4
2008-2012	29.2	29.2	89.5	31.0	43.4	109.9	14.2	15.0	104.8	15.2	19.1	107.5
	35-44			>74 y/o			35-44			>74 y/o		
1983-1987	32.0	30.4	95.6	11.2	89.1	113.8	12.2	12.3	104.5	9.4	52.4	90.8
1988-1992	20.2	20.9	88.0	11.2	71.2	110.4	13.4	14.0	144.8	11.0	44.1	92.2
1993-1997	19.2	23.3	95.1	8.8	48.8	93.6	6.4	8.6	100.7	6.0	19.9	57.7
1998-2002	25.0	29.4	83.5	12.6	53.7	102.0	9.0	11.6	112.0	9.8	24.4	78.6
2003-2007	34.4	32.1	80.2	12.8	41.0	91.1	14.8	15.1	119.5	10.0	19.2	85.4
2008-2012	43.4	34.8	93.6	19.0	44.7	107.8	16.0	13.9	102.8	13.4	19.8	103.2

Secondary Medical Zone ID: 111

	Male						Female					
	Suicide			Suicide			Suicide			Suicide		
	Num per year	Rate /100,000	×100	Num per year	Rate /100,000	×100	Num per year	Rate /100,000	×100	Num per year	Rate /100,000	×100
	Total (>10 y/o)			45-54			Total (>10 y/o)			45-54		
1983-1987	37.0	30.5	89.1	9.4	44.1	87.2	20.4	15.5	104.7	3.4	17.0	98.5
1988-1992	34.2	22.8	90.2	8.4	31.1	90.7	18.8	12.0	91.1	3.0	13.0	89.0
1993-1997	43.6	24.5	97.8	13.4	41.0	112.1	18.4	10.3	90.9	3.0	11.1	86.0
1998-2002	65.8	34.6	96.0	16.4	54.1	96.7	22.0	11.7	87.5	4.4	15.0	99.6
2003-2007	61.8	32.7	87.8	11.8	46.0	82.3	23.2	12.3	92.8	1.6	9.5	69.5
2008-2012	68.4	35.5	103.0	12.6	49.9	102.6	25.2	13.2	98.9	5.0	19.0	127.6
	15-24			55-64			15-24			55-64		
1983-1987	3.2	13.1	93.6	4.8	38.6	90.8	2.8	9.6	141.3	3.0	20.6	104.4
1988-1992	3.0	9.6	97.2	5.4	31.2	88.0	1.6	5.5	104.3	3.4	18.7	104.8
1993-1997	2.8	9.9	87.4	8.0	36.8	91.4	1.4	5.1	97.6	2.6	13.6	85.9
1998-2002	4.6	16.8	106.1	15.6	59.1	93.0	1.6	6.8	97.4	3.8	16.5	87.4
2003-2007	4.2	17.9	102.5	14.2	48.9	84.8	1.4	7.7	85.7	5.0	16.9	100.8
2008-2012	3.8	19.5	92.2	14.4	49.0	100.9	1.6	9.0	92.7	4.4	15.1	100.9
	25-34			65-74			25-34			65-74		
1983-1987	5.6	24.2	94.8	3.6	48.9	111.6	2.6	11.6	107.5	3.0	32.4	103.7
1988-1992	4.2	16.8	87.9	2.4	29.5	83.6	2.4	9.8	112.5	2.4	22.2	85.1
1993-1997	4.6	16.4	83.7	3.8	30.6	96.0	3.2	10.6	127.8	2.6	18.5	94.9
1998-2002	9.6	29.1	107.9	5.8	38.2	83.9	4.2	13.7	118.2	3.2	19.3	91.9
2003-2007	10.2	33.7	108.6	8.0	40.0	93.2	3.8	14.2	108.8	4.2	19.7	106.0
2008-2012	10.4	39.9	122.6	9.4	38.7	98.1	3.6	16.0	111.8	6.0	21.7	122.2
	35-44			>74 y/o			35-44			>74 y/o		
1983-1987	6.0	22.1	69.3	4.2	104.4	133.3	3.6	12.1	102.4	2.0	44.6	77.4
1988-1992	6.0	20.4	86.3	4.6	77.9	120.8	3.4	10.9	112.5	2.6	29.9	62.5
1993-1997	7.0	25.1	102.2	4.0	57.3	110.1	2.8	10.0	117.9	2.8	22.9	66.5
1998-2002	9.6	36.0	102.1	4.2	49.1	93.2	1.6	8.2	79.6	3.2	20.2	64.9
2003-2007	7.2	27.6	68.9	6.2	50.6	112.4	4.6	15.9	126.2	2.6	13.8	61.4
2008-2012	11.8	38.1	102.4	5.8	39.0	94.0	2.6	11.3	83.6	2.0	10.3	53.6

Secondary Medical Zone ID: 112

	Male						Female					
	Suicide			Suicide			Suicide			Suicide		
	Num per year	Rate /100,000	×100	Num per year	Rate /100,000	×100	Num per year	Rate /100,000	×100	Num per year	Rate /100,000	×100
	Total (>10 y/o)			45–54			Total (>10 y/o)			45–54		
1983–1987	95.0	25.0	71.5	27.8	37.9	75.0	53.6	13.9	88.8	11.0	16.0	92.8
1988–1992	91.2	19.2	76.2	22.2	25.7	74.8	55.8	11.4	88.1	11.6	13.4	91.6
1993–1997	116.0	21.0	82.2	27.6	28.9	79.0	58.6	10.2	93.0	13.4	13.2	102.1
1998–2002	176.2	28.9	79.6	39.4	43.5	77.6	77.6	12.1	94.9	15.0	15.5	102.9
2003–2007	171.8	27.0	72.7	28.8	36.0	64.4	89.4	13.5	103.9	12.8	15.1	110.3
2008–2012	192.8	29.8	83.9	33.6	40.1	82.5	93.0	13.4	102.7	12.2	15.0	100.5
	15–24			55–64			15–24			55–64		
1983–1987	12.4	12.6	90.7	9.4	26.0	61.3	5.0	6.3	91.5	8.6	20.6	104.5
1988–1992	13.0	10.1	102.3	16.6	28.8	81.2	3.8	3.9	74.9	7.2	13.8	77.2
1993–1997	14.2	11.1	98.1	23.6	32.1	79.8	5.0	4.8	92.7	9.8	13.9	87.6
1998–2002	15.0	13.2	83.8	45.0	52.2	82.1	8.8	8.9	127.0	16.0	18.1	96.4
2003–2007	16.2	16.0	91.9	41.0	43.3	75.0	7.2	8.6	95.5	14.0	14.3	85.1
2008–2012	17.4	19.7	93.1	34.8	38.6	79.5	7.0	9.2	94.7	15.0	15.2	101.6
	25–34			65–74			25–34			65–74		
1983–1987	14.6	20.7	80.8	5.8	33.0	75.3	6.6	9.7	89.7	7.6	30.4	97.3
1988–1992	9.4	12.2	63.7	6.6	26.7	75.8	5.6	7.9	91.4	7.2	22.5	86.1
1993–1997	15.8	16.7	85.5	10.2	27.1	85.1	9.8	11.1	134.0	7.2	17.1	87.9
1998–2002	27.6	26.0	96.3	19.4	36.9	81.2	9.8	10.6	91.8	9.2	17.0	81.1
2003–2007	25.2	25.1	81.1	20.0	30.0	69.8	18.0	18.4	141.7	13.0	18.2	97.5
2008–2012	30.6	34.1	104.7	24.8	31.4	79.6	12.6	15.3	106.7	16.2	18.3	102.7
	35–44			>74 y/o			35–44			>74 y/o		
1983–1987	16.8	18.6	58.4	8.2	86.6	110.6	7.8	8.5	72.1	6.6	48.7	84.3
1988–1992	15.2	17.3	73.1	8.0	58.5	90.6	9.4	10.1	104.4	10.4	44.1	92.2
1993–1997	16.0	20.4	83.3	8.2	46.8	89.8	5.8	7.6	89.2	7.4	24.0	69.7
1998–2002	20.6	25.6	72.7	8.6	37.1	70.4	7.6	9.9	95.8	10.8	25.9	83.4
2003–2007	27.0	28.0	69.8	13.6	38.4	85.4	13.6	14.7	116.2	10.8	19.4	86.6
2008–2012	33.6	30.1	80.9	17.6	35.8	86.3	17.2	15.9	117.1	12.2	17.0	88.3

Secondary Medical Zone ID: 113

	Male						Female					
	Suicide			Suicide			Suicide			Suicide		
	Num per year	Rate /100,000	×100	Num per year	Rate /100,000	×100	Num per year	Rate /100,000	×100	Num per year	Rate /100,000	×100
	Total (>10 y/o)			45-54			Total (>10 y/o)			45-54		
1983-1987	63.8	31.1	93.0	16.8	46.4	91.7	27.6	13.8	88.2	4.2	13.1	75.9
1988-1992	57.8	24.4	99.1	14.8	36.5	106.4	28.4	12.0	91.8	3.8	11.0	74.9
1993-1997	56.0	22.0	85.3	13.4	31.5	86.1	28.4	10.4	95.3	5.6	12.7	98.2
1998-2002	85.8	31.1	84.8	15.4	38.8	69.3	39.6	13.1	103.7	7.0	16.2	107.5
2003-2007	92.4	32.1	86.1	18.0	47.4	84.8	36.8	12.4	94.8	6.2	15.7	114.8
2008-2012	90.6	31.2	87.2	18.0	44.8	92.1	38.8	12.8	95.2	6.6	16.7	111.8
	15-24			55-64			15-24			55-64		
1983-1987	4.4	9.9	71.0	9.0	42.3	99.5	2.6	6.3	92.5	4.4	19.3	97.6
1988-1992	5.4	10.0	100.9	9.2	32.1	90.4	3.0	5.9	112.7	5.2	17.7	99.2
1993-1997	5.0	10.4	91.7	11.6	34.8	86.4	2.0	4.6	88.5	5.2	15.1	94.7
1998-2002	8.0	17.7	112.3	22.8	60.7	95.6	4.4	9.5	135.0	7.4	18.6	99.0
2003-2007	7.6	19.2	109.9	21.4	52.1	90.2	3.0	8.5	94.4	5.8	14.3	85.6
2008-2012	6.4	19.5	91.7	16.8	41.9	86.2	3.2	9.7	99.8	5.8	14.1	94.3
	25-34			65-74			25-34			65-74		
1983-1987	10.0	24.3	95.2	5.8	50.5	115.4	4.2	11.3	105.3	3.6	27.3	87.3
1988-1992	7.2	16.6	86.9	5.4	38.9	110.4	4.4	10.7	123.2	3.4	20.9	80.2
1993-1997	9.6	19.3	98.8	3.6	21.7	68.1	5.4	11.3	136.3	3.2	15.8	81.0
1998-2002	14.4	27.3	101.0	8.4	35.3	77.6	6.4	13.2	113.9	4.2	16.6	79.1
2003-2007	13.2	27.8	89.7	11.8	38.5	89.7	7.2	15.4	118.2	4.6	14.9	79.9
2008-2012	15.6	34.6	106.4	11.4	34.6	87.7	6.0	14.8	103.5	6.8	17.5	98.5
	35-44			>74 y/o			35-44			>74 y/o		
1983-1987	13.8	30.3	95.3	3.8	76.2	97.3	5.4	11.8	100.3	3.2	45.5	78.9
1988-1992	10.4	23.7	100.1	5.2	69.6	107.9	3.8	9.3	96.1	4.8	43.3	90.6
1993-1997	7.6	20.0	81.6	5.2	56.2	107.8	3.4	9.1	106.6	3.6	25.2	73.3
1998-2002	12.2	30.2	85.7	4.6	41.3	78.4	3.8	10.3	99.4	6.0	30.4	97.7
2003-2007	15.4	33.1	82.6	4.8	31.2	69.3	4.8	11.6	92.1	4.8	18.8	84.0
2008-2012	16.8	32.7	87.9	5.4	26.7	64.5	6.2	13.0	96.2	3.8	12.5	65.2

Secondary Medical Zone ID: 114

	Male						Female					
	Suicide			Suicide			Suicide			Suicide		
	Num per year	Rate /100,000	×100	Num per year	Rate /100,000	×100	Num per year	Rate /100,000	×100	Num per year	Rate /100,000	×100
	Total (>10 y/o)			45-54			Total (>10 y/o)			45-54		
1983-1987	88.6	25.9	76.9	21.2	40.4	79.8	49.4	14.2	92.6	9.0	16.1	93.2
1988-1992	73.0	20.0	77.8	17.6	31.1	90.5	51.0	12.7	100.4	9.0	14.8	101.4
1993-1997	83.2	20.7	80.5	20.8	33.3	91.0	48.0	11.0	100.8	10.8	16.0	123.7
1998-2002	115.4	27.2	73.9	26.4	43.7	78.0	56.4	12.3	95.1	8.2	14.0	92.9
2003-2007	110.6	25.9	66.9	22.2	39.1	69.8	58.6	12.7	96.3	8.0	14.3	104.5
2008-2012	126.2	28.0	78.8	20.0	32.8	67.4	60.4	12.6	95.0	9.6	15.8	106.1
	15-24			55-64			15-24			55-64		
1983-1987	11.8	12.6	90.4	11.6	33.5	78.8	4.4	6.4	93.4	8.6	20.9	106.2
1988-1992	6.2	7.2	72.4	11.2	25.9	72.8	7.0	8.4	159.3	7.4	15.9	88.8
1993-1997	7.6	9.4	83.2	16.2	33.4	82.9	4.4	5.9	113.1	9.2	17.0	106.9
1998-2002	12.0	16.0	101.6	24.2	47.7	75.1	5.6	8.2	116.9	12.6	21.4	113.7
2003-2007	10.2	16.1	92.3	22.2	39.8	69.0	8.8	13.9	155.1	9.2	15.5	92.7
2008-2012	10.4	18.8	88.8	26.8	46.4	95.5	4.6	9.0	92.4	7.8	13.8	92.7
	25-34			65-74			25-34			65-74		
1983-1987	13.6	17.9	70.0	6.0	33.5	76.5	6.6	10.4	96.7	8.2	33.3	106.6
1988-1992	12.0	15.1	78.8	6.0	27.5	78.1	6.2	9.3	107.2	5.8	20.5	78.7
1993-1997	14.0	16.3	83.5	6.8	23.4	73.3	6.4	8.7	104.6	4.6	13.6	69.6
1998-2002	20.0	22.6	83.8	9.4	27.2	59.8	9.0	11.5	99.3	8.0	18.5	88.0
2003-2007	19.2	23.3	75.1	10.4	26.6	62.1	10.0	13.0	99.9	7.2	15.3	82.0
2008-2012	19.8	26.7	82.1	14.4	33.8	85.4	9.2	13.2	91.9	8.4	16.4	91.9
	35-44			>74 y/o			35-44			>74 y/o		
1983-1987	19.4	28.3	89.1	4.8	53.7	68.5	7.2	10.8	91.8	5.0	37.6	65.2
1988-1992	13.2	20.1	84.9	6.6	52.6	81.6	8.8	13.0	134.2	6.4	33.6	70.3
1993-1997	11.2	18.8	76.6	6.2	40.8	78.3	5.8	10.0	118.2	6.8	27.1	78.8
1998-2002	14.4	22.7	64.5	8.8	43.3	82.1	5.6	9.7	93.5	7.2	22.4	72.1
2003-2007	19.8	26.5	66.2	6.6	25.9	57.6	9.0	12.8	101.7	6.2	15.0	66.8
2008-2012	26.2	31.4	84.4	8.4	26.2	63.1	11.4	14.2	104.4	8.8	16.5	85.6

Secondary Medical Zone ID: 115

	Male						Female					
	Suicide			Suicide			Suicide			Suicide		
	Num per year	Rate /100,000	×100	Num per year	Rate /100,000	×100	Num per year	Rate /100,000	×100	Num per year	Rate /100,000	×100
	Total (>10 y/o)			45-54			Total (>10 y/o)			45-54		
1983-1987	60.8	26.4	77.6	15.8	37.9	74.9	35.2	14.8	95.8	6.8	15.6	90.8
1988-1992	52.6	20.1	79.0	12.4	28.3	82.5	33.4	11.8	90.9	7.2	14.8	101.3
1993-1997	62.2	21.4	82.8	14.4	31.2	85.2	30.2	9.8	86.6	6.2	12.5	96.8
1998-2002	98.4	31.5	85.7	22.2	48.7	87.1	44.4	12.7	99.2	6.8	14.7	97.3
2003-2007	90.6	28.6	75.1	14.8	35.8	64.1	45.0	12.9	98.5	4.2	11.0	80.4
2008-2012	95.0	29.7	82.0	17.8	39.6	81.5	43.0	12.5	91.5	4.6	11.5	77.3
	15-24			55-64			15-24			55-64		
1983-1987	7.0	12.8	91.9	9.2	35.6	83.7	4.0	7.7	113.0	4.4	17.1	86.7
1988-1992	6.6	10.7	107.6	8.8	25.7	72.3	3.4	6.0	112.9	7.2	19.1	107.1
1993-1997	5.4	10.2	90.2	14.0	34.7	86.2	2.8	5.4	103.5	5.4	13.2	82.9
1998-2002	9.6	19.0	120.3	20.4	49.3	77.6	2.8	6.5	92.0	10.0	20.6	109.2
2003-2007	7.8	17.9	102.6	19.8	44.8	77.5	5.4	12.0	133.4	8.4	16.9	100.7
2008-2012	8.0	20.4	96.2	20.6	46.1	95.0	3.4	9.0	92.6	7.6	15.8	106.0
	25-34			65-74			25-34			65-74		
1983-1987	11.0	23.6	92.5	4.4	38.0	86.7	4.6	10.9	101.1	7.0	42.0	134.3
1988-1992	8.6	17.4	90.9	4.4	28.4	80.6	4.2	9.2	106.2	4.4	22.6	86.7
1993-1997	11.6	20.6	105.6	3.6	17.8	55.8	5.4	10.2	122.7	4.8	18.2	93.2
1998-2002	17.0	29.2	108.0	11.4	36.9	81.2	9.0	15.7	135.6	6.6	19.3	91.7
2003-2007	14.4	27.9	89.9	11.6	32.8	76.5	9.0	17.0	130.4	5.8	14.9	79.8
2008-2012	12.6	28.4	87.1	11.4	31.5	79.8	6.6	14.9	104.1	4.8	12.4	69.6
	35-44			>74 y/o			35-44			>74 y/o		
1983-1987	10.8	23.0	72.1	2.6	53.9	68.8	4.6	9.8	83.1	3.8	46.4	80.3
1988-1992	8.8	19.1	80.4	3.0	41.0	63.5	3.2	7.8	80.3	3.6	28.4	59.3
1993-1997	8.6	20.2	82.5	4.6	44.4	85.2	3.4	8.3	97.8	2.2	14.5	42.3
1998-2002	12.6	28.0	79.4	5.0	37.0	70.2	4.2	9.9	95.6	5.0	21.6	69.6
2003-2007	16.0	31.1	77.5	6.2	30.6	68.0	6.2	12.6	99.7	5.6	17.7	79.0
2008-2012	17.4	31.3	84.0	7.0	26.4	63.6	9.2	15.9	117.4	6.4	15.4	80.1

Secondary Medical Zone ID: 116

	Male						Female					
	Suicide			Suicide			Suicide			Suicide		
	Num per year	Rate /100,000	×100	Num per year	Rate /100,000	×100	Num per year	Rate /100,000	×100	Num per year	Rate /100,000	×100
	Total (>10 y/o)			45-54			Total (>10 y/o)			45-54		
1983-1987	7.6	36.1	129.5	1.4	53.5	105.7	4.8	16.9	130.2	0.6	18.5	107.4
1988-1992	7.0	28.0	140.1	1.8	45.2	131.7	2.6	12.9	103.3	0.2	14.2	97.0
1993-1997	8.6	29.6	153.0	1.8	42.8	117.0	3.0	11.4	116.9	0.6	14.5	111.9
1998-2002	7.8	38.5	120.4	3.2	73.5	131.3	1.4	12.2	90.8	0.2	14.6	97.0
2003-2007	10.8	42.3	151.3	3.4	86.4	154.6	2.0	12.4	100.5	0.2	13.5	98.6
2008-2012	5.8	34.1	104.8	0.8	46.0	94.6	2.6	13.1	107.8	0.4	15.9	106.9
	15-24			55-64			15-24			55-64		
1983-1987	0.4	15.9	114.0	1.0	43.7	102.8	0.4	8.3	120.9	1.0	23.6	119.4
1988-1992	0.6	12.0	121.3	1.4	42.5	119.7	0.0	5.0	94.5	0.4	17.7	99.0
1993-1997	0.4	12.5	110.8	1.8	50.2	124.7	0.4	6.7	128.3	0.0	13.5	85.1
1998-2002	0.2	16.1	101.6	1.0	59.6	93.9	0.2	7.6	108.4	0.0	16.9	90.0
2003-2007	0.4	19.0	109.1	2.2	66.2	114.6	0.0	8.5	94.8	0.2	16.0	95.7
2008-2012	0.0	20.0	94.1	1.6	50.5	103.9	0.0	9.3	95.4	0.8	17.0	113.8
	25-34			65-74			25-34			65-74		
1983-1987	1.0	29.3	114.7	1.4	57.9	132.2	0.6	13.1	121.6	0.6	31.8	101.8
1988-1992	0.6	21.2	111.0	0.2	30.1	85.5	0.0	8.0	91.8	0.4	24.4	93.6
1993-1997	0.8	23.1	118.1	1.2	41.2	129.1	0.2	8.7	104.1	0.8	23.6	121.1
1998-2002	0.2	25.1	92.8	0.8	45.7	100.6	0.0	10.8	93.3	0.4	20.8	99.2
2003-2007	0.2	28.2	91.0	2.2	61.0	142.1	0.0	12.0	92.4	0.2	17.3	93.0
2008-2012	0.2	30.8	94.6	1.2	44.2	111.9	0.2	14.5	101.1	0.6	19.3	108.4
	35-44			>74 y/o			35-44			>74 y/o		
1983-1987	1.0	33.0	103.6	1.4	108.4	138.3	0.6	13.4	113.6	1.0	69.0	119.5
1988-1992	1.0	26.4	111.2	1.4	90.1	139.7	0.4	10.4	108.0	1.2	61.9	129.3
1993-1997	1.4	32.3	131.7	1.2	71.3	137.0	0.0	7.7	90.1	1.0	43.6	126.6
1998-2002	1.6	45.4	128.9	0.8	57.6	109.2	0.4	11.6	112.6	0.2	22.9	73.8
2003-2007	1.2	46.1	115.0	1.2	55.7	123.7	0.2	12.7	100.7	1.2	32.6	145.1
2008-2012	0.8	37.4	100.6	1.2	49.3	118.8	0.0	12.6	93.2	0.6	19.9	103.6

Secondary Medical Zone ID: 117

	Male						Female					
	Suicide			Suicide			Suicide			Suicide		
	Num per year	Rate /100,000	×100	Num per year	Rate /100,000	×100	Num per year	Rate /100,000	×100	Num per year	Rate /100,000	×100
	Total (>10 y/o)			45-54			Total (>10 y/o)			45-54		
1983-1987	110.6	26.9	77.5	23.0	33.7	66.7	60.6	14.5	95.8	9.2	14.3	82.8
1988-1992	96.6	20.1	78.3	18.4	23.1	67.2	57.8	11.9	92.4	9.2	12.2	83.2
1993-1997	123.2	21.8	86.0	29.2	30.8	84.2	56.0	10.1	91.7	10.4	11.6	89.9
1998-2002	174.8	28.1	77.3	42.8	44.6	79.7	80.6	12.8	100.0	11.2	12.7	84.5
2003-2007	169.0	25.8	68.2	36.0	39.2	70.2	70.8	10.8	83.1	7.6	9.7	70.5
2008-2012	192.0	27.5	78.2	34.6	34.3	70.5	88.8	12.5	95.5	12.6	13.7	91.5
	15-24			55-64			15-24			55-64		
1983-1987	10.8	11.1	79.2	19.8	44.8	105.3	7.0	8.5	124.9	9.4	20.2	102.4
1988-1992	8.0	7.6	76.9	20.4	35.6	100.3	5.2	5.7	107.9	9.4	16.7	93.8
1993-1997	12.2	11.6	102.4	27.6	40.5	100.7	5.8	6.3	120.4	8.4	13.1	82.5
1998-2002	12.6	13.9	87.7	45.6	58.1	91.4	8.2	9.6	136.0	14.2	18.3	97.5
2003-2007	12.4	14.9	85.7	36.4	41.0	71.0	5.8	8.0	89.4	12.6	14.5	86.8
2008-2012	16.4	19.9	93.6	36.0	39.7	81.6	6.4	8.9	92.0	14.2	15.6	104.6
	25-34			65-74			25-34			65-74		
1983-1987	17.2	18.3	71.5	9.0	40.5	92.6	7.6	9.6	88.7	7.6	28.2	90.2
1988-1992	16.6	15.6	81.4	9.6	35.0	99.3	6.4	7.4	85.2	8.8	25.8	98.8
1993-1997	17.0	14.2	72.6	12.0	32.4	101.5	7.8	7.7	92.0	8.2	19.2	98.5
1998-2002	29.2	22.6	83.7	14.6	32.2	70.8	15.8	13.2	114.1	8.4	16.7	79.4
2003-2007	29.0	23.9	77.0	16.8	30.3	70.6	12.0	10.8	82.8	10.6	17.2	92.2
2008-2012	27.4	25.8	79.2	20.8	31.5	79.7	15.0	14.7	102.5	13.4	18.2	102.2
	35-44			>74 y/o			35-44			>74 y/o		
1983-1987	21.4	22.6	70.9	8.6	83.2	106.2	11.4	12.4	105.2	8.0	52.1	90.3
1988-1992	15.2	16.0	67.5	8.4	58.9	91.3	8.2	9.2	95.6	10.4	46.6	97.5
1993-1997	17.6	19.2	78.2	7.2	41.3	79.2	7.2	8.6	101.1	7.6	26.7	77.7
1998-2002	22.2	21.9	62.3	7.2	32.7	61.9	11.8	12.4	119.8	11.0	28.7	92.3
2003-2007	30.4	24.8	61.8	7.8	25.8	57.2	13.4	12.0	94.8	8.6	16.8	75.1
2008-2012	39.8	29.5	79.4	16.4	37.5	90.5	16.2	12.6	93.1	10.6	15.7	81.9

Secondary Medical Zone ID: 118

	Male						Female					
	Suicide			Suicide			Suicide			Suicide		
	Num per year	Rate /100,000	×100	Num per year	Rate /100,000	×100	Num per year	Rate /100,000	×100	Num per year	Rate /100,000	×100
	Total (>10 y/o)			45-54			Total (>10 y/o)			45-54		
1983-1987	101.0	25.8	74.2	27.2	35.8	70.9	49.2	13.1	81.0	7.2	11.0	63.8
1988-1992	80.2	17.9	68.9	19.4	23.2	67.6	52.2	11.1	85.3	10.0	12.1	82.8
1993-1997	101.8	19.8	76.9	28.4	31.4	85.9	49.4	9.3	83.6	9.0	10.1	78.1
1998-2002	166.6	30.3	82.4	35.8	43.1	77.0	74.6	12.5	98.6	15.8	17.6	116.8
2003-2007	166.0	29.4	79.1	30.8	42.1	75.3	68.8	12.3	89.8	9.8	13.4	97.6
2008-2012	166.4	29.3	83.1	31.0	41.4	85.1	75.2	12.8	93.9	10.4	14.5	97.0
	15-24			55-64			15-24			55-64		
1983-1987	8.4	9.8	70.2	13.2	31.1	73.1	4.2	5.6	82.5	7.8	18.1	91.6
1988-1992	10.8	10.5	106.1	12.4	21.5	60.6	4.6	5.1	97.0	11.4	19.0	106.5
1993-1997	9.8	10.7	94.6	21.6	30.2	74.9	4.6	5.4	103.2	10.8	15.1	94.8
1998-2002	12.4	16.1	102.2	39.6	49.7	78.3	7.2	9.4	134.3	15.4	18.5	98.1
2003-2007	11.8	18.0	103.0	39.8	47.2	81.7	8.0	12.4	137.8	13.6	15.3	91.2
2008-2012	10.6	18.2	86.0	32.2	40.7	83.7	8.2	13.6	140.5	10.4	12.8	85.5
	25-34			65-74			25-34			65-74		
1983-1987	15.2	20.5	80.3	7.0	35.7	81.5	6.2	9.2	85.6	7.4	29.1	93.2
1988-1992	6.6	8.9	46.7	9.2	33.8	95.9	5.2	7.3	84.6	8.0	24.5	94.0
1993-1997	12.4	13.8	70.8	8.2	22.2	69.6	7.8	9.1	109.8	5.2	13.2	67.6
1998-2002	22.8	23.3	86.4	18.8	36.5	80.2	10.4	11.5	99.0	10.0	18.4	87.7
2003-2007	24.2	27.0	87.1	23.0	36.1	84.0	12.4	14.2	109.2	9.8	14.9	80.1
2008-2012	20.6	27.7	85.2	24.8	34.8	88.0	9.4	13.2	92.1	14.8	18.4	103.3
	35-44			>74 y/o			35-44			>74 y/o		
1983-1987	21.0	22.9	72.0	8.4	90.6	115.7	8.4	9.3	78.5	8.0	57.5	99.7
1988-1992	15.2	17.5	73.7	6.6	51.8	80.4	7.6	8.8	91.0	5.4	27.4	57.3
1993-1997	13.0	17.2	70.3	7.2	43.1	82.7	4.4	6.4	75.0	7.6	27.2	79.1
1998-2002	26.6	33.7	95.7	10.2	44.0	83.4	6.0	8.6	83.2	9.8	26.0	83.8
2003-2007	25.8	28.9	72.2	10.6	31.7	70.4	9.0	11.1	87.6	6.0	12.2	54.4
2008-2012	32.8	32.5	87.3	14.4	30.9	74.4	13.6	14.0	103.0	8.4	12.5	65.1

Secondary Medical Zone ID: 119

	Male						Female					
	Suicide			Suicide			Suicide			Suicide		
	Num per year	Rate /100,000	×100	Num per year	Rate /100,000	×100	Num per year	Rate /100,000	×100	Num per year	Rate /100,000	×100
	Total (>10 y/o)			45–54			Total (>10 y/o)			45–54		
1983–1987	102.0	30.1	89.9	29.2	48.1	95.2	47.8	14.0	90.4	8.0	14.2	82.5
1988–1992	84.2	22.0	88.5	18.2	27.6	80.5	52.8	13.0	102.9	10.2	15.3	104.6
1993–1997	107.0	25.1	100.4	26.4	35.6	97.2	46.4	10.6	97.3	7.2	10.6	82.4
1998–2002	154.6	34.6	96.3	35.6	51.1	91.3	60.6	13.0	102.3	11.8	16.9	112.5
2003–2007	145.8	32.0	88.3	30.6	50.4	90.1	63.4	13.4	106.4	6.0	11.2	81.4
2008–2012	151.0	33.6	97.3	30.0	49.2	101.3	62.2	14.0	103.3	11.0	18.5	123.9
	15–24			55–64			15–24			55–64		
1983–1987	7.4	11.7	83.8	16.8	42.3	99.5	5.2	8.3	122.2	7.8	19.2	97.1
1988–1992	7.2	9.9	99.7	18.6	36.3	102.1	5.4	7.5	141.3	7.0	14.7	82.2
1993–1997	9.8	13.8	122.2	21.8	37.9	94.2	6.4	9.0	171.8	8.8	15.5	97.4
1998–2002	10.0	17.6	111.4	41.4	65.1	102.4	3.4	6.6	94.1	8.8	15.1	80.0
2003–2007	7.2	15.7	90.0	43.4	61.8	107.1	5.2	10.8	120.5	13.4	18.9	113.1
2008–2012	7.8	18.8	88.8	38.0	55.2	113.6	6.2	13.9	143.0	11.4	16.9	113.0
	25–34			65–74			25–34			65–74		
1983–1987	13.2	21.3	83.2	8.0	42.0	95.9	7.2	12.0	111.1	4.8	21.8	69.7
1988–1992	12.8	19.4	101.7	5.8	25.7	73.1	6.0	9.8	113.1	6.6	22.6	86.5
1993–1997	14.6	20.5	104.8	12.4	36.1	113.2	6.6	9.9	119.3	6.4	17.4	89.1
1998–2002	18.8	25.9	95.8	18.0	42.0	92.4	10.6	14.8	127.5	9.0	19.8	94.1
2003–2007	16.0	24.8	79.8	20.2	40.7	95.0	12.2	17.8	137.1	9.0	17.3	92.9
2008–2012	15.6	28.4	87.1	19.8	36.7	92.9	7.6	14.2	99.2	7.0	13.3	74.6
	35–44			>74 y/o			35–44			>74 y/o		
1983–1987	19.2	25.4	79.9	7.8	84.4	107.8	7.4	10.3	87.6	7.4	52.7	91.4
1988–1992	14.2	19.6	82.5	7.2	60.7	94.0	8.4	11.4	118.6	9.0	47.0	98.3
1993–1997	15.4	24.3	99.2	6.6	45.3	87.1	3.6	6.6	77.9	7.4	30.6	89.0
1998–2002	21.6	34.9	99.2	9.2	46.7	88.5	7.0	11.8	113.9	9.8	30.8	99.3
2003–2007	19.0	28.5	71.1	9.2	33.9	75.2	8.0	12.6	99.4	9.4	22.4	99.6
2008–2012	26.4	35.7	95.9	12.6	35.0	84.4	10.0	14.2	104.9	9.0	17.1	88.7

Secondary Medical Zone ID: 120

	Male						Female					
	Suicide			Suicide			Suicide			Suicide		
	Num per year	Rate /100,000	×100	Num per year	Rate /100,000	×100	Num per year	Rate /100,000	×100	Num per year	Rate /100,000	×100
	Total (>10 y/o)			45–54			Total (>10 y/o)			45–54		
1983–1987	50.0	25.3	69.8	14.8	41.3	81.7	25.2	13.3	83.5	5.6	16.2	94.1
1988–1992	49.0	19.7	76.2	13.2	30.9	89.9	27.8	11.4	89.1	4.8	12.2	83.6
1993–1997	62.2	21.4	82.5	16.0	33.0	90.3	26.4	9.4	85.3	5.8	12.1	93.9
1998–2002	93.8	29.3	78.6	18.6	40.0	71.4	37.6	11.7	90.4	7.4	15.3	101.5
2003–2007	88.4	26.1	67.1	18.4	40.1	71.7	41.0	11.7	90.3	3.8	10.0	73.2
2008–2012	106.8	29.2	82.0	18.2	35.8	73.7	47.8	12.2	95.9	7.2	14.8	99.5
	15–24			55–64			15–24			55–64		
1983–1987	4.4	8.7	62.5	5.4	30.3	71.3	2.4	5.8	85.0	3.0	16.5	83.8
1988–1992	6.0	9.2	93.0	6.0	23.5	66.1	3.4	6.1	114.7	6.8	22.3	124.8
1993–1997	8.4	12.9	113.9	11.0	32.7	81.3	3.2	5.8	111.8	3.4	11.4	71.7
1998–2002	7.8	14.4	91.0	22.8	56.9	89.6	3.6	7.4	105.7	8.2	19.6	104.0
2003–2007	5.2	11.9	68.2	21.4	47.5	82.2	4.0	9.1	100.9	6.4	14.5	86.3
2008–2012	9.4	20.5	96.6	21.2	46.1	94.9	1.2	4.5	46.6	8.4	16.9	113.1
	25–34			65–74			25–34			65–74		
1983–1987	8.6	17.7	69.3	4.6	47.6	108.6	3.0	7.7	71.9	2.8	26.9	86.0
1988–1992	8.8	14.7	77.0	2.6	25.2	71.4	3.8	7.8	89.8	2.0	16.9	64.9
1993–1997	10.0	14.5	74.0	4.8	28.2	88.3	5.4	8.9	106.7	3.8	19.5	100.1
1998–2002	15.2	20.6	76.3	9.2	39.3	86.4	4.8	8.2	70.7	4.6	18.6	88.5
2003–2007	12.8	18.8	60.7	9.0	31.7	73.9	9.4	14.0	107.6	6.2	18.8	100.9
2008–2012	17.6	28.1	86.4	11.6	34.2	86.5	8.2	13.9	96.9	6.4	16.8	94.3
	35–44			>74 y/o			35–44			>74 y/o		
1983–1987	9.4	19.8	62.1	2.6	68.1	87.0	5.0	10.6	89.4	3.0	50.9	88.3
1988–1992	9.4	18.5	78.2	3.0	53.6	83.1	3.6	8.0	83.4	3.4	38.7	80.8
1993–1997	6.8	15.0	61.1	5.2	63.3	121.6	2.4	6.2	73.2	2.4	21.7	63.2
1998–2002	13.6	25.1	71.2	6.0	54.9	104.1	5.0	10.2	98.7	4.0	24.5	79.1
2003–2007	15.8	24.2	60.3	5.4	35.8	79.6	7.8	12.9	102.0	3.2	14.4	64.2
2008–2012	21.0	29.2	78.4	7.0	32.8	79.0	10.4	14.6	107.7	6.0	17.9	93.1

Secondary Medical Zone ID: 121

	Male						Female					
	Suicide			Suicide			Suicide			Suicide		
	Num per year	Rate /100,000	×100	Num per year	Rate /100,000	×100	Num per year	Rate /100,000	×100	Num per year	Rate /100,000	×100
	Total (>10 y/o)			45-54			Total (>10 y/o)			45-54		
1983-1987	71.8	32.7	99.5	14.2	42.0	83.0	29.8	14.8	96.4	5.4	16.6	96.4
1988-1992	58.8	24.7	99.4	15.4	38.9	113.3	23.2	11.1	82.5	3.8	12.4	84.6
1993-1997	64.6	25.2	100.5	16.2	38.0	103.9	24.6	10.4	94.3	4.2	12.1	94.1
1998-2002	99.8	36.2	103.1	24.4	59.2	105.8	31.6	12.6	98.0	6.4	17.6	116.9
2003-2007	92.0	32.5	89.1	19.0	51.3	91.7	33.0	12.2	98.8	3.8	13.0	94.9
2008-2012	100.8	33.1	98.6	14.8	39.6	81.5	37.8	13.5	104.7	6.4	18.5	123.9
	15-24			55-64			15-24			55-64		
1983-1987	8.2	17.0	122.0	14.0	55.5	130.7	2.4	6.5	95.7	4.8	19.3	97.8
1988-1992	3.4	7.9	80.1	10.2	35.7	100.6	1.2	3.8	72.9	3.4	13.9	77.6
1993-1997	4.4	10.8	95.4	13.6	42.8	106.3	2.4	6.2	119.2	3.4	12.6	79.5
1998-2002	5.2	15.4	97.4	26.8	75.6	119.0	1.8	6.5	91.9	6.8	20.7	110.0
2003-2007	3.8	14.1	80.5	26.2	66.8	115.7	2.4	8.9	99.5	6.4	18.2	108.8
2008-2012	6.6	22.0	103.6	22.6	57.2	117.7	3.0	11.0	113.3	5.0	15.1	101.0
	25-34			65-74			25-34			65-74		
1983-1987	10.2	21.6	84.5	8.2	57.4	131.0	5.0	12.9	119.8	4.4	29.1	93.2
1988-1992	7.8	15.9	83.3	4.8	33.1	93.8	2.8	7.9	90.6	4.6	25.2	96.6
1993-1997	10.0	18.7	95.6	5.4	29.9	93.7	4.0	9.3	112.2	4.0	18.9	96.9
1998-2002	14.6	25.8	95.5	10.8	48.3	106.1	4.2	10.0	86.5	4.4	18.7	89.2
2003-2007	12.4	22.5	72.5	12.2	48.1	112.2	4.0	9.7	74.7	5.6	20.3	108.8
2008-2012	13.6	26.2	80.5	13.2	45.7	115.8	5.6	12.9	90.2	5.4	18.7	104.8
	35-44			>74 y/o			35-44			>74 y/o		
1983-1987	12.6	27.8	87.2	4.2	79.3	101.3	3.6	10.1	85.4	4.2	55.7	96.6
1988-1992	11.0	24.2	102.1	6.0	76.3	118.2	3.4	9.4	97.7	4.0	39.5	82.5
1993-1997	10.4	25.8	105.2	4.4	48.5	93.2	3.4	9.9	117.0	3.0	23.5	68.4
1998-2002	11.2	28.7	81.4	6.8	57.7	109.4	3.8	11.3	109.8	4.0	23.7	76.3
2003-2007	12.6	27.3	68.2	5.4	38.4	85.3	4.4	11.8	93.5	6.4	26.9	119.9
2008-2012	19.2	34.1	91.5	10.8	56.0	134.9	6.0	12.9	95.2	6.2	21.1	109.9

Secondary Medical Zone ID: 122

	Male						Female					
	Suicide			Suicide			Suicide			Suicide		
	Num per year	Rate /100,000	×100	Num per year	Rate /100,000	×100	Num per year	Rate /100,000	×100	Num per year	Rate /100,000	×100
	Total (>10 y/o)			45–54			Total (>10 y/o)			45–54		
1983–1987	80.4	27.7	82.6	21.2	41.4	81.8	39.0	12.6	78.9	6.6	13.5	78.1
1988–1992	66.4	20.8	81.8	11.6	22.2	64.7	42.4	11.8	89.2	6.6	12.2	83.3
1993–1997	71.8	21.5	81.4	15.2	27.2	74.2	36.2	9.6	84.0	4.4	8.4	65.4
1998–2002	120.8	33.6	92.2	29.6	54.7	97.7	54.2	13.8	103.1	9.6	16.6	110.3
2003–2007	108.4	31.6	82.7	19.0	42.7	76.4	45.0	12.0	90.1	7.0	14.9	109.0
2008–2012	101.2	30.7	84.1	18.8	43.2	88.9	49.6	13.2	98.5	7.2	16.4	109.7
	15–24			55–64			15–24			55–64		
1983–1987	6.8	12.7	91.0	14.8	40.8	96.1	3.6	7.0	102.2	5.2	14.3	72.7
1988–1992	6.6	10.1	102.1	14.6	32.9	92.7	2.0	4.0	75.9	7.2	15.9	89.2
1993–1997	8.2	13.4	118.1	13.8	29.4	73.0	4.0	6.9	133.2	7.4	14.7	92.3
1998–2002	8.0	16.9	106.9	30.2	57.4	90.3	4.8	10.1	143.0	10.6	18.7	99.4
2003–2007	8.8	21.0	120.3	24.8	45.2	78.3	3.8	10.4	116.0	8.0	14.0	83.5
2008–2012	7.6	21.5	101.5	20.6	41.0	84.3	2.2	7.9	81.3	8.6	15.3	102.3
	25–34			65–74			25–34			65–74		
1983–1987	11.6	25.9	101.5	4.2	24.0	54.9	3.4	8.7	81.2	6.4	25.2	80.6
1988–1992	10.0	20.7	108.4	6.8	29.0	82.2	6.4	13.4	154.6	6.2	20.5	78.5
1993–1997	8.2	16.5	84.5	7.4	24.7	77.3	3.4	7.7	93.1	5.4	15.1	77.6
1998–2002	15.8	28.7	106.2	13.0	35.1	77.3	6.4	12.6	108.6	6.6	16.1	76.7
2003–2007	12.0	25.3	81.6	16.6	38.4	89.4	6.2	13.4	102.8	6.4	14.2	76.2
2008–2012	11.2	29.2	89.6	13.0	28.9	73.2	6.8	17.1	119.5	9.2	17.2	96.4
	35–44			>74 y/o			35–44			>74 y/o		
1983–1987	13.2	22.9	71.9	8.4	80.2	102.4	6.6	10.6	89.8	7.2	45.6	79.0
1988–1992	10.6	19.7	83.1	6.2	46.7	72.4	4.2	8.1	83.9	9.6	43.1	90.1
1993–1997	12.6	26.6	108.6	6.0	37.2	71.5	4.4	9.4	110.8	7.2	26.2	76.2
1998–2002	13.8	31.0	88.0	10.2	49.0	93.0	7.6	15.8	152.4	8.4	24.4	78.7
2003–2007	19.4	38.9	97.2	7.6	29.6	65.6	5.6	12.1	96.0	8.0	18.3	81.8
2008–2012	19.6	36.0	96.7	10.0	29.8	71.8	7.2	13.6	100.6	8.2	15.4	80.2

Secondary Medical Zone ID: 123

	Male						Female					
	Suicide			Suicide			Suicide			Suicide		
	Num per year	Rate /100,000	×100	Num per year	Rate /100,000	×100	Num per year	Rate /100,000	×100	Num per year	Rate /100,000	×100
	Total (>10 y/o)			45–54			Total (>10 y/o)			45–54		
1983–1987	59.2	27.9	83.3	16.8	44.2	87.3	25.4	12.8	78.6	4.0	12.7	73.8
1988–1992	49.0	20.4	79.8	11.2	26.0	75.7	29.2	11.6	88.4	5.8	13.5	92.5
1993–1997	52.6	19.7	74.6	14.0	29.3	80.1	25.8	9.3	81.3	5.0	10.6	82.4
1998–2002	79.0	27.5	72.5	18.2	40.7	72.8	36.2	11.9	88.9	7.0	14.6	97.1
2003–2007	92.8	30.1	79.7	18.2	44.7	79.9	38.2	11.7	90.9	3.8	10.6	77.1
2008–2012	85.6	27.7	77.2	14.2	34.6	71.3	37.2	11.3	85.7	5.6	13.9	93.3
	15–24			55–64			15–24			55–64		
1983–1987	7.8	16.0	114.9	6.6	32.7	76.9	1.8	5.3	77.9	4.2	18.6	94.4
1988–1992	5.2	9.7	97.5	5.6	21.4	60.2	2.8	5.7	108.3	5.6	18.6	103.9
1993–1997	4.0	8.6	76.2	11.6	32.6	80.9	2.0	4.5	87.0	4.2	12.6	79.3
1998–2002	6.2	14.4	91.0	19.0	46.6	73.3	2.8	6.9	98.6	7.6	17.8	94.5
2003–2007	4.2	12.8	73.1	21.2	46.5	80.5	3.8	10.3	114.0	6.8	14.6	87.0
2008–2012	6.2	18.4	86.6	15.4	36.8	75.7	2.8	8.8	91.1	5.6	12.7	84.9
	25–34			65–74			25–34			65–74		
1983–1987	7.2	19.8	77.6	4.0	37.3	85.3	2.8	8.6	80.1	2.6	21.0	67.1
1988–1992	8.0	18.5	96.7	5.2	35.8	101.5	4.2	10.2	118.1	3.2	19.3	74.0
1993–1997	9.2	18.2	93.2	2.6	17.0	53.2	3.2	7.5	90.1	4.8	20.6	105.8
1998–2002	11.8	22.3	82.6	6.6	29.0	63.7	5.6	11.3	97.4	3.6	14.7	69.9
2003–2007	17.6	33.3	107.5	10.4	33.3	77.6	5.6	11.9	91.3	7.0	19.6	105.4
2008–2012	9.4	23.4	71.8	11.6	31.9	80.7	4.0	10.9	76.0	6.2	15.6	87.7
	35–44			>74 y/o			35–44			>74 y/o		
1983–1987	12.4	25.4	79.7	3.8	65.5	83.6	4.2	9.2	77.8	5.4	59.1	102.4
1988–1992	10.6	22.3	94.1	3.2	43.6	67.7	3.0	7.3	76.0	4.6	36.1	75.5
1993–1997	8.2	20.0	81.4	2.6	30.5	58.6	3.6	8.7	102.1	3.0	19.5	56.8
1998–2002	11.4	26.5	75.3	5.2	42.3	80.3	5.4	12.3	118.8	4.0	19.9	64.1
2003–2007	15.0	28.8	72.0	6.0	35.4	78.6	5.0	11.0	86.8	6.0	21.4	95.2
2008–2012	21.0	35.9	96.4	7.8	33.9	81.7	5.8	11.2	82.7	7.0	19.3	100.4

Secondary Medical Zone ID: 124

	Male						Female					
	Suicide			Suicide			Suicide			Suicide		
	Num per year	Rate /100,000	×100	Num per year	Rate /100,000	×100	Num per year	Rate /100,000	×100	Num per year	Rate /100,000	×100
	Total (>10 y/o)			45-54			Total (>10 y/o)			45-54		
1983-1987	52.0	27.7	80.7	13.0	41.0	81.0	33.4	16.4	110.5	4.6	16.0	92.6
1988-1992	47.2	21.3	83.2	9.2	23.9	69.7	26.6	11.8	89.7	4.8	13.2	90.5
1993-1997	52.2	20.8	80.0	14.2	30.7	84.0	23.2	9.3	81.8	5.4	12.1	93.7
1998-2002	82.6	30.4	82.1	19.4	44.2	79.0	36.2	12.5	98.8	6.2	14.2	94.4
2003-2007	90.4	32.5	86.2	17.8	47.4	84.7	31.6	11.7	87.6	4.8	13.2	96.0
2008-2012	89.0	31.0	89.8	14.2	40.6	83.5	33.4	12.1	90.4	3.4	11.6	77.7
	15-24			55-64			15-24			55-64		
1983-1987	5.4	11.9	85.7	5.6	32.1	75.6	2.4	6.8	100.1	3.8	18.8	95.4
1988-1992	5.6	10.0	100.6	7.8	30.8	86.9	2.2	5.1	97.0	3.2	14.3	80.3
1993-1997	6.2	11.4	100.4	10.6	34.0	84.4	1.4	3.8	73.1	4.2	14.2	89.1
1998-2002	5.6	12.8	80.9	19.4	50.8	80.0	2.6	6.8	97.4	7.0	18.5	98.2
2003-2007	7.4	17.6	101.0	22.0	49.6	85.9	2.0	7.0	77.7	5.2	13.1	78.5
2008-2012	6.0	18.1	85.3	23.0	51.2	105.4	2.6	9.2	94.7	6.0	13.9	93.0
	25-34			65-74			25-34			65-74		
1983-1987	6.6	19.6	76.6	4.6	43.3	98.9	4.8	13.2	122.6	5.8	39.0	124.7
1988-1992	6.4	17.0	88.8	4.8	35.9	101.8	2.8	8.4	97.4	4.8	27.6	105.7
1993-1997	6.4	15.4	78.7	4.4	26.3	82.4	2.8	7.7	92.5	4.0	19.3	98.9
1998-2002	11.0	24.2	89.6	9.2	40.7	89.5	6.0	13.6	117.5	5.4	21.4	102.0
2003-2007	11.6	27.3	88.1	9.2	33.1	77.1	6.4	15.3	117.8	6.2	20.0	107.4
2008-2012	9.6	27.2	83.6	13.6	38.0	96.2	5.4	15.5	108.3	6.4	17.6	98.7
	35-44			>74 y/o			35-44			>74 y/o		
1983-1987	12.8	27.4	86.1	3.8	72.2	92.1	6.6	13.6	115.6	5.4	63.9	110.7
1988-1992	8.6	19.4	81.7	4.6	61.7	95.6	3.8	8.9	92.2	4.6	39.0	81.5
1993-1997	8.0	20.7	84.3	2.4	31.3	60.0	2.2	6.6	78.2	3.2	22.1	64.2
1998-2002	12.0	32.0	90.8	6.0	51.1	96.9	3.0	9.1	88.4	6.0	29.8	96.0
2003-2007	16.0	38.8	96.7	6.4	40.7	90.4	4.2	11.8	93.1	2.6	11.8	52.5
2008-2012	14.8	33.1	88.9	7.8	37.8	91.2	5.4	13.3	97.8	4.2	14.2	73.9

Secondary Medical Zone ID: 125

	Male						Female					
	Suicide			Suicide			Suicide			Suicide		
	Num per year	Rate /100,000	×100	Num per year	Rate /100,000	×100	Num per year	Rate /100,000	×100	Num per year	Rate /100,000	×100
	Total (>10 y/o)			45–54			Total (>10 y/o)			45–54		
1983–1987	67.8	28.7	82.8	17.0	38.4	76.0	30.6	14.5	89.3	4.8	13.6	78.9
1988–1992	65.8	21.8	89.1	20.6	35.3	102.7	31.4	11.5	88.2	6.6	13.0	88.9
1993–1997	79.6	22.9	91.1	20.2	31.7	86.5	31.8	9.7	88.8	7.8	12.4	96.2
1998–2002	117.0	31.5	85.4	26.4	44.3	79.1	46.4	12.5	98.2	8.4	14.2	94.2
2003–2007	124.8	31.7	86.0	21.6	41.7	74.5	46.4	12.1	94.9	6.4	13.0	95.2
2008–2012	127.6	32.1	92.5	27.0	51.6	106.2	49.0	12.9	96.5	7.0	15.1	100.9
	15–24			55–64			15–24			55–64		
1983–1987	6.4	11.5	82.6	7.6	36.1	84.9	2.0	5.0	73.2	4.8	20.9	105.8
1988–1992	7.8	10.4	105.0	7.6	25.1	70.7	3.2	5.2	98.7	5.2	17.5	98.2
1993–1997	10.6	14.2	125.5	15.2	35.2	87.4	3.4	5.4	104.3	5.6	14.5	91.4
1998–2002	9.2	15.7	99.2	27.8	51.8	81.6	3.8	7.2	102.9	11.0	20.5	108.9
2003–2007	7.2	15.1	86.7	30.2	49.8	86.2	2.8	7.1	79.0	9.0	15.0	89.8
2008–2012	5.4	14.2	66.8	23.4	41.2	84.8	4.4	10.9	111.9	7.6	13.3	89.1
	25–34			65–74			25–34			65–74		
1983–1987	10.6	22.4	87.6	4.8	45.8	104.5	2.4	6.9	64.1	5.2	36.2	115.8
1988–1992	8.4	15.6	81.5	2.8	24.6	69.7	3.4	7.8	89.9	3.2	20.2	77.3
1993–1997	13.4	20.0	102.4	4.8	26.0	81.5	2.6	5.7	68.4	4.4	19.9	101.9
1998–2002	18.0	24.5	90.8	9.6	36.3	79.8	8.8	13.4	115.2	3.4	14.2	67.5
2003–2007	19.6	28.6	92.2	15.8	40.9	95.4	7.4	12.4	95.3	7.4	19.3	103.6
2008–2012	16.8	30.1	92.5	17.6	36.5	92.4	8.6	16.4	114.3	7.4	15.6	87.9
	35–44			>74 y/o			35–44			>74 y/o		
1983–1987	16.2	25.5	80.1	4.6	90.5	115.5	6.2	10.2	86.5	5.2	71.9	124.5
1988–1992	13.8	22.2	93.8	4.4	63.2	98.0	4.8	8.4	87.4	4.8	43.1	90.2
1993–1997	10.0	18.9	77.2	5.0	55.5	106.5	3.6	7.5	88.5	4.4	29.3	85.3
1998–2002	17.8	32.5	92.3	8.0	64.3	122.0	6.0	11.9	115.2	4.6	23.8	76.5
2003–2007	23.4	37.1	92.6	7.0	41.2	91.5	7.4	13.2	104.8	5.4	20.4	91.1
2008–2012	26.0	36.1	96.9	10.8	44.3	106.7	8.4	13.3	98.3	5.6	16.6	86.1

Secondary Medical Zone ID: 126

	Male						Female					
	Suicide			Suicide			Suicide			Suicide		
	Num per year	Rate /100,000	×100	Num per year	Rate /100,000	×100	Num per year	Rate /100,000	×100	Num per year	Rate /100,000	×100
	Total (>10 y/o)			45–54			Total (>10 y/o)			45–54		
1983–1987	59.2	30.4	87.2	12.6	34.8	68.9	24.4	13.4	84.3	4.2	14.0	81.0
1988–1992	47.0	20.2	77.9	13.6	29.2	85.1	26.2	11.5	86.4	5.8	13.6	92.8
1993–1997	61.2	21.8	84.7	15.2	30.1	82.2	26.4	9.5	86.3	6.4	12.4	96.2
1998–2002	100.2	31.5	87.0	21.2	44.0	78.6	43.8	13.2	105.8	7.6	15.2	100.8
2003–2007	104.6	31.8	85.6	19.4	44.9	80.3	39.0	12.3	92.0	6.2	14.4	104.9
2008–2012	106.6	32.6	91.7	20.4	46.4	95.4	45.0	13.0	101.0	6.4	15.5	103.9
	15–24			55–64			15–24			55–64		
1983–1987	7.0	14.5	104.0	5.2	30.8	72.5	2.0	5.4	79.7	2.2	14.4	72.9
1988–1992	5.0	8.6	87.0	7.4	28.2	79.3	1.8	3.9	73.8	3.2	14.2	79.8
1993–1997	7.4	12.2	107.8	10.8	30.9	76.8	3.0	5.4	104.0	3.8	12.5	78.5
1998–2002	7.0	14.4	91.1	26.4	58.7	92.3	3.0	6.5	92.6	8.2	18.8	99.7
2003–2007	6.8	15.9	91.0	25.4	51.4	89.1	4.2	9.7	107.5	5.4	12.2	73.1
2008–2012	8.6	21.5	101.4	17.8	39.1	80.6	4.6	11.3	116.8	8.4	16.3	109.6
	25–34			65–74			25–34			65–74		
1983–1987	7.8	21.5	83.9	7.8	71.9	164.1	3.2	9.5	88.3	4.2	33.9	108.3
1988–1992	6.6	15.5	80.9	3.2	29.1	82.5	2.2	6.7	77.0	4.6	29.2	111.7
1993–1997	10.6	19.0	97.5	5.2	30.6	95.8	3.4	7.5	89.9	3.4	18.2	93.3
1998–2002	17.2	27.2	100.8	11.8	47.7	104.9	8.4	14.3	123.4	6.0	23.3	110.7
2003–2007	21.0	34.9	112.5	9.4	30.8	71.8	7.8	14.4	110.4	6.4	19.4	104.0
2008–2012	15.6	32.5	99.9	12.4	31.8	80.6	6.2	14.2	99.1	6.6	16.3	91.6
	35–44			>74 y/o			35–44			>74 y/o		
1983–1987	13.8	26.8	84.3	4.8	104.6	133.5	6.2	11.8	100.1	2.4	43.5	75.4
1988–1992	8.2	17.6	74.1	3.0	53.2	82.5	3.8	8.4	86.7	4.8	47.0	98.3
1993–1997	8.0	18.7	76.4	3.8	49.2	94.4	2.2	6.2	73.3	4.0	29.5	85.6
1998–2002	12.2	26.8	76.2	4.4	43.8	83.0	4.4	10.4	100.8	6.2	33.0	106.2
2003–2007	17.2	32.3	80.7	5.0	34.8	77.3	6.6	13.4	106.5	2.4	11.6	51.8
2008–2012	24.2	39.8	107.0	7.4	35.5	85.6	6.8	12.9	95.0	6.0	19.1	99.2

Secondary Medical Zone ID: 127

	Male						Female					
	Suicide			Suicide			Suicide			Suicide		
	Num per year	Rate /100,000	×100	Num per year	Rate /100,000	×100	Num per year	Rate /100,000	×100	Num per year	Rate /100,000	×100
	Total (>10 y/o)			45–54			Total (>10 y/o)			45–54		
1983–1987	39.8	29.7	88.8	10.0	43.6	86.2	18.2	13.0	81.1	4.0	16.9	98.1
1988–1992	35.4	23.1	93.5	8.0	31.1	90.7	19.6	11.7	88.0	2.8	12.2	83.3
1993–1997	37.8	22.9	90.3	12.0	40.3	110.0	16.8	9.4	83.0	4.0	13.3	102.8
1998–2002	58.6	34.1	93.3	13.0	48.8	87.2	20.6	11.4	84.7	4.0	14.6	97.2
2003–2007	57.8	34.2	91.3	13.2	55.8	99.8	25.0	13.2	101.9	3.8	15.1	110.1
2008–2012	63.2	35.6	106.3	12.0	52.7	108.4	22.6	12.2	94.6	4.2	17.7	118.4
	15–24			55–64			15–24			55–64		
1983–1987	3.2	13.1	94.2	4.8	33.3	78.3	0.8	4.9	72.2	3.8	20.3	103.1
1988–1992	3.2	10.9	110.4	8.6	40.4	113.7	1.2	4.8	91.7	3.4	16.6	92.9
1993–1997	2.0	9.3	81.8	9.2	39.8	98.9	0.6	3.7	70.2	2.4	12.0	75.3
1998–2002	5.0	20.1	127.0	16.2	64.0	100.7	1.6	7.3	103.3	3.8	16.1	85.3
2003–2007	3.0	16.7	95.5	12.6	47.6	82.5	1.8	9.4	105.0	5.2	17.4	104.2
2008–2012	4.0	22.6	106.7	12.0	45.5	93.7	0.6	6.4	65.8	4.0	14.6	97.7
	25–34			65–74			25–34			65–74		
1983–1987	7.6	30.9	120.9	4.6	49.2	112.4	1.0	7.0	65.0	3.4	28.8	92.1
1988–1992	4.0	17.7	92.5	3.6	33.7	95.8	2.4	10.0	115.3	3.2	22.9	87.8
1993–1997	3.2	14.5	74.3	4.8	33.4	104.6	2.0	8.5	101.6	3.2	18.9	96.8
1998–2002	4.6	20.3	75.2	6.8	40.8	89.6	2.2	10.1	87.3	3.8	19.7	93.9
2003–2007	8.8	35.0	112.8	9.8	47.9	111.5	3.8	15.2	116.6	2.8	14.6	78.4
2008–2012	6.0	31.1	95.5	10.8	46.0	116.4	2.0	12.4	86.2	4.2	17.1	96.2
	35–44			>74 y/o			35–44			>74 y/o		
1983–1987	7.0	25.7	80.7	2.4	58.5	74.7	2.2	9.2	77.5	2.8	41.3	71.7
1988–1992	5.2	19.9	83.9	2.8	50.0	77.6	1.6	7.5	77.6	5.0	46.8	97.9
1993–1997	4.0	18.3	74.8	2.6	39.5	75.9	1.6	7.5	88.2	3.0	24.1	70.1
1998–2002	8.0	34.6	98.4	5.0	54.2	102.9	1.6	8.6	83.4	3.4	22.1	71.0
2003–2007	6.6	29.3	73.1	3.6	33.6	74.6	3.8	14.8	117.5	3.8	19.1	85.1
2008–2012	10.8	40.2	108.0	7.4	46.2	111.5	2.8	12.4	91.7	4.6	18.6	96.8

Secondary Medical Zone ID: 128

	Male						Female					
	Suicide			Suicide			Suicide			Suicide		
	Num per year	Rate /100,000	×100	Num per year	Rate /100,000	×100	Num per year	Rate /100,000	×100	Num per year	Rate /100,000	×100
	Total (>10 y/o)			45–54			Total (>10 y/o)			45–54		
1983–1987	49.2	42.8	146.2	12.2	71.1	140.5	21.4	16.2	110.3	3.0	17.3	100.3
1988–1992	40.2	32.6	148.0	8.2	48.3	140.5	25.0	15.3	134.9	2.0	13.7	93.8
1993–1997	43.6	33.4	150.1	9.8	50.3	137.4	28.8	14.9	170.5	3.4	16.4	127.3
1998–2002	60.4	45.1	143.5	15.0	75.3	134.6	30.4	15.6	155.6	2.8	15.5	103.2
2003–2007	64.2	49.2	154.4	13.4	76.9	137.6	22.8	13.4	128.9	3.2	16.7	121.9
2008–2012	53.4	43.5	139.8	12.0	73.7	151.6	17.4	13.5	107.5	3.2	18.9	126.9
	15–24			55–64			15–24			55–64		
1983–1987	3.8	22.6	161.8	11.0	68.4	160.9	0.8	6.6	96.3	2.8	17.8	90.2
1988–1992	1.6	10.7	108.2	10.2	57.0	160.7	0.6	5.0	95.3	3.8	19.8	110.7
1993–1997	3.0	15.6	137.8	8.8	52.3	129.9	0.6	5.0	95.5	6.2	27.8	175.2
1998–2002	2.0	15.7	99.4	13.8	86.4	136.0	1.0	7.5	106.3	5.2	25.7	136.5
2003–2007	1.8	16.7	95.5	12.8	73.3	127.0	1.0	9.1	101.5	3.0	17.4	104.1
2008–2012	4.0	29.4	138.7	11.4	58.8	121.0	1.6	12.7	131.0	1.2	11.2	75.4
	25–34			65–74			25–34			65–74		
1983–1987	6.0	33.4	130.5	4.8	52.6	120.2	2.2	12.5	116.3	6.2	45.9	146.8
1988–1992	4.8	29.0	151.8	4.0	39.6	112.4	1.6	10.1	116.5	6.8	43.1	165.2
1993–1997	4.8	29.4	150.3	6.2	45.6	143.0	0.8	7.6	91.1	5.4	29.5	151.3
1998–2002	5.4	34.4	127.3	11.0	68.9	151.5	2.0	13.3	115.1	6.4	31.7	151.1
2003–2007	4.8	34.6	111.6	10.2	65.9	153.6	1.0	11.2	85.8	4.4	23.5	126.5
2008–2012	5.6	40.5	124.5	7.6	51.9	131.3	1.0	12.6	87.6	3.0	19.0	106.7
	35–44			>74 y/o			35–44			>74 y/o		
1983–1987	6.6	36.3	114.1	4.8	97.6	124.6	1.6	10.8	91.4	4.8	61.1	106.0
1988–1992	6.6	32.2	135.9	4.8	78.3	121.4	2.4	11.5	118.9	7.8	71.5	149.5
1993–1997	5.8	31.8	129.8	5.2	72.0	138.3	1.8	9.9	115.9	10.2	74.1	215.3
1998–2002	6.2	40.4	114.6	6.8	76.4	145.0	1.0	8.9	86.4	12.0	70.0	225.3
2003–2007	10.8	70.1	175.1	10.2	84.6	187.9	0.4	8.8	69.3	9.8	46.2	205.7
2008–2012	6.4	43.6	117.2	6.4	49.4	119.0	1.6	13.3	98.3	5.8	24.5	127.4

Secondary Medical Zone ID: 129

	Male						Female					
	Suicide			Suicide			Suicide			Suicide		
	Num per year	Rate /100,000	×100	Num per year	Rate /100,000	×100	Num per year	Rate /100,000	×100	Num per year	Rate /100,000	×100
	Total (>10 y/o)			45-54			Total (>10 y/o)			45-54		
1983-1987	124.6	35.0	108.3	36.2	62.8	124.2	64.6	15.5	102.6	9.0	15.4	89.6
1988-1992	103.6	26.6	109.5	22.0	36.5	106.4	71.4	14.4	118.3	10.4	16.4	111.9
1993-1997	113.4	26.9	108.7	25.6	37.2	101.7	70.0	12.8	126.4	9.2	13.3	102.9
1998-2002	181.6	41.0	115.4	49.4	68.2	121.8	77.2	13.3	116.0	11.0	15.3	101.9
2003-2007	183.4	40.9	114.2	40.2	63.3	113.2	66.6	12.6	103.6	7.8	12.6	91.8
2008-2012	159.6	36.9	106.7	31.8	55.7	114.5	67.0	13.0	104.5	10.0	17.0	113.7
	15-24			55-64			15-24			55-64		
1983-1987	6.4	11.6	83.0	19.8	44.5	104.8	4.2	7.1	104.4	9.6	18.7	94.7
1988-1992	6.8	10.7	107.8	19.4	37.2	104.9	2.8	4.7	88.7	12.8	21.5	120.6
1993-1997	7.2	11.2	98.9	21.4	39.5	98.2	3.6	5.6	106.4	11.0	18.2	114.4
1998-2002	9.8	16.3	103.4	41.0	71.2	112.1	4.2	7.2	102.5	13.4	21.4	113.8
2003-2007	9.2	17.4	100.0	45.4	68.9	119.4	4.6	8.9	99.2	12.8	18.5	110.5
2008-2012	10.8	23.0	108.2	39.0	55.3	113.9	4.2	9.3	96.2	11.2	15.5	103.8
	25-34			65-74			25-34			65-74		
1983-1987	17.4	27.8	108.7	9.6	38.8	88.6	7.6	11.8	109.9	14.0	38.7	123.8
1988-1992	11.4	20.3	106.4	11.6	37.9	107.5	5.6	9.5	110.0	10.0	24.1	92.5
1993-1997	11.8	20.6	105.6	12.6	32.6	102.1	5.8	9.6	115.7	13.2	25.9	132.7
1998-2002	21.4	33.8	125.2	18.2	41.0	90.1	5.4	9.5	82.1	16.4	28.9	137.5
2003-2007	20.4	33.3	107.2	24.6	50.6	117.9	7.4	12.4	95.5	11.2	19.7	105.7
2008-2012	16.6	31.1	95.6	19.8	39.7	100.4	7.8	14.5	101.3	12.0	20.3	114.3
	35-44			>74 y/o			35-44			>74 y/o		
1983-1987	24.2	34.4	108.2	10.6	80.5	102.8	6.8	10.2	86.0	13.4	62.8	108.8
1988-1992	20.4	27.9	117.6	11.8	68.9	106.9	6.8	9.5	98.2	22.8	74.4	155.5
1993-1997	19.4	29.6	120.7	15.0	71.1	136.6	5.6	8.7	101.9	21.4	55.0	159.9
1998-2002	26.0	43.7	124.0	15.8	60.6	114.9	4.2	7.9	76.4	22.4	45.4	146.1
2003-2007	27.6	46.9	117.1	15.8	46.6	103.5	6.6	11.6	92.2	16.2	26.5	117.9
2008-2012	26.2	41.2	110.7	15.0	37.0	89.1	5.6	10.1	74.9	16.2	22.3	116.0

Secondary Medical Zone ID: 130

	Male						Female					
	Suicide			Suicide			Suicide			Suicide		
	Num per year	Rate /100,000	×100	Num per year	Rate /100,000	×100	Num per year	Rate /100,000	×100	Num per year	Rate /100,000	×100
	Total (>10 y/o)			45–54			Total (>10 y/o)			45–54		
1983–1987	39.0	36.1	115.0	12.4	67.5	133.5	20.8	16.5	112.5	3.6	19.1	110.6
1988–1992	34.4	28.6	124.9	8.6	45.6	132.8	29.0	17.4	160.4	3.0	15.9	108.6
1993–1997	35.6	28.2	120.5	7.4	38.7	105.8	19.2	12.1	122.9	2.4	13.0	100.4
1998–2002	58.0	43.3	132.2	14.6	71.0	126.8	21.4	13.3	116.2	3.0	15.5	103.1
2003–2007	52.4	42.3	121.4	13.0	72.4	129.5	19.6	13.0	112.7	2.2	13.5	98.6
2008–2012	44.6	36.5	112.7	8.2	53.2	109.5	18.4	13.2	108.8	2.8	17.0	114.1
	15–24			55–64			15–24			55–64		
1983–1987	2.4	14.9	107.2	7.6	52.7	123.9	1.2	7.2	105.5	3.6	21.8	110.5
1988–1992	1.0	8.4	84.8	7.4	44.9	126.3	1.2	6.2	118.0	4.6	23.4	131.0
1993–1997	2.0	12.0	105.8	4.8	33.3	82.8	1.8	7.9	151.3	2.6	15.6	98.3
1998–2002	3.4	20.0	126.3	14.0	80.7	127.0	0.8	6.6	94.1	3.2	18.9	100.2
2003–2007	2.6	19.4	111.3	11.2	62.3	108.0	0.6	7.3	81.3	3.2	17.3	103.1
2008–2012	1.6	18.6	87.6	7.6	43.4	89.4	1.0	9.8	100.5	2.4	14.0	93.6
	25–34			65–74			25–34			65–74		
1983–1987	4.6	26.2	102.3	3.4	43.7	99.9	1.4	9.4	87.6	4.6	39.7	127.0
1988–1992	3.4	21.2	111.1	4.0	40.8	115.9	2.2	11.4	131.5	5.6	39.6	152.0
1993–1997	3.6	22.3	114.2	6.0	46.6	146.1	1.4	8.9	106.4	3.4	22.4	115.0
1998–2002	7.0	37.6	139.1	8.6	59.2	130.2	1.0	9.5	81.7	5.6	30.6	145.7
2003–2007	5.6	35.2	113.5	5.0	38.1	88.9	1.8	12.8	98.6	4.0	22.4	120.3
2008–2012	5.8	39.3	120.6	8.2	51.2	129.7	1.8	14.5	101.4	3.6	20.3	114.3
	35–44			>74 y/o			35–44			>74 y/o		
1983–1987	5.2	28.4	89.1	3.4	84.3	107.7	1.2	9.1	76.8	5.2	79.1	137.0
1988–1992	6.2	29.1	122.8	3.8	72.2	112.0	2.8	12.0	124.4	9.6	102.8	214.8
1993–1997	5.2	28.1	114.8	6.6	93.1	178.7	1.0	7.3	85.4	6.4	55.8	162.1
1998–2002	5.8	36.8	104.5	4.2	55.4	105.1	1.4	9.9	95.7	6.2	42.8	138.0
2003–2007	7.6	48.4	120.7	7.4	68.6	152.3	1.8	12.5	99.2	5.8	32.4	144.2
2008–2012	5.6	36.8	99.0	7.6	58.5	140.9	1.2	11.2	82.8	5.6	26.2	136.2

Secondary Medical Zone ID: 131

	Male						Female					
	Suicide			Suicide			Suicide			Suicide		
	Num per year	Rate /100,000	×100	Num per year	Rate /100,000	×100	Num per year	Rate /100,000	×100	Num per year	Rate /100,000	×100
	Total (>10 y/o)			45–54			Total (>10 y/o)			45–54		
1983–1987	87.6	40.8	130.1	21.8	66.2	130.9	59.8	21.0	154.3	6.8	19.5	113.3
1988–1992	80.8	33.7	146.7	14.2	42.8	124.6	57.8	17.4	160.7	6.6	18.3	125.4
1993–1997	80.2	32.1	136.3	14.0	38.6	105.5	49.0	14.0	154.2	5.2	14.3	110.8
1998–2002	109.6	42.9	128.3	25.6	67.1	119.9	53.4	15.2	144.5	5.6	15.5	102.7
2003–2007	103.4	41.6	121.4	22.8	67.2	120.2	43.8	13.6	128.0	4.0	12.9	94.1
2008–2012	88.2	38.1	113.0	18.4	60.4	124.3	42.0	14.4	125.8	5.2	17.2	115.5
	15–24			55–64			15–24			55–64		
1983–1987	4.4	15.5	111.0	13.8	48.2	113.5	1.4	5.9	86.9	10.2	28.5	144.5
1988–1992	4.8	13.4	135.5	17.2	52.6	148.2	1.0	4.3	81.0	9.2	24.8	138.6
1993–1997	5.0	13.9	122.7	14.8	47.4	117.7	1.0	4.2	80.7	8.8	24.1	151.5
1998–2002	5.2	17.0	107.4	23.2	75.0	118.1	2.6	8.7	123.6	7.4	21.9	116.3
2003–2007	4.6	18.1	104.0	22.6	65.2	113.0	2.2	9.6	107.0	6.8	18.9	112.9
2008–2012	3.8	19.1	90.0	17.0	47.1	96.9	3.2	13.9	143.6	6.0	16.1	107.9
	25–34			65–74			25–34			65–74		
1983–1987	11.6	33.2	130.1	12.0	64.5	147.3	2.4	8.5	78.7	12.2	48.8	156.3
1988–1992	7.8	24.8	129.8	9.2	44.4	126.0	3.0	9.8	112.7	12.0	42.1	161.2
1993–1997	10.0	30.5	156.1	13.2	50.9	159.7	3.0	9.8	117.9	9.6	29.6	151.9
1998–2002	9.2	29.5	109.1	16.4	58.5	128.6	3.0	11.0	95.1	11.4	32.8	156.0
2003–2007	10.2	33.4	107.7	14.8	53.8	125.4	2.4	10.4	80.1	7.6	23.1	124.3
2008–2012	9.8	35.7	109.7	12.4	45.4	115.0	2.8	12.8	89.3	7.4	22.6	127.2
	35–44			>74 y/o			35–44			>74 y/o		
1983–1987	11.0	30.3	95.2	12.8	130.8	167.0	4.8	13.0	110.4	22.0	140.9	244.2
1988–1992	15.0	36.4	153.7	12.4	100.1	155.2	3.0	8.8	91.1	23.0	110.8	231.5
1993–1997	12.0	33.3	135.7	11.0	75.7	145.4	2.0	6.9	81.7	19.4	75.8	220.2
1998–2002	13.6	43.4	123.2	16.4	90.6	172.0	2.8	9.9	96.0	20.4	64.6	207.9
2003–2007	12.8	43.4	108.3	15.6	69.5	154.4	3.6	12.9	102.3	17.0	44.5	198.3
2008–2012	13.8	43.0	115.6	12.8	50.2	121.0	2.4	10.6	78.3	14.8	33.5	174.3

Secondary Medical Zone ID: 132

	Male						Female					
	Suicide			Suicide			Suicide			Suicide		
	Num per year	Rate /100,000	×100	Num per year	Rate /100,000	×100	Num per year	Rate /100,000	×100	Num per year	Rate /100,000	×100
	Total (>10 y/o)			45–54			Total (>10 y/o)			45–54		
1983–1987	52.0	46.5	165.1	9.2	61.6	121.9	31.6	21.6	169.3	4.0	22.8	132.2
1988–1992	41.8	34.5	165.8	7.6	49.5	144.3	33.0	18.7	192.5	2.4	16.3	111.3
1993–1997	45.2	35.4	172.2	6.2	40.6	111.1	30.2	15.9	200.5	3.4	18.1	140.4
1998–2002	57.0	47.7	155.0	12.4	72.7	130.0	34.0	18.0	194.7	6.0	26.5	176.2
2003–2007	53.0	46.5	149.4	10.2	68.2	121.9	27.0	15.4	169.7	3.8	20.1	146.6
2008–2012	47.2	41.5	144.6	9.0	66.9	137.6	20.2	14.5	137.1	1.8	15.7	105.5
	15–24			55–64			15–24			55–64		
1983–1987	3.2	22.3	160.0	10.0	64.0	150.6	0.8	7.3	107.0	3.4	21.1	106.8
1988–1992	1.4	11.0	111.6	7.8	49.1	138.4	0.0	3.6	68.2	3.4	20.4	114.1
1993–1997	1.2	11.2	99.4	9.4	60.7	150.7	0.6	5.4	103.2	4.0	23.0	144.7
1998–2002	2.8	20.7	131.3	10.8	80.2	126.2	0.6	6.8	96.8	3.2	21.7	115.5
2003–2007	2.8	23.1	132.7	8.8	62.8	108.8	0.6	8.5	94.3	3.4	20.6	122.8
2008–2012	2.0	23.7	111.9	11.4	64.8	133.5	1.0	11.4	117.3	3.4	18.6	124.5
	25–34			65–74			25–34			65–74		
1983–1987	5.2	31.7	124.2	6.4	68.1	155.5	0.6	7.7	71.7	5.0	42.3	135.4
1988–1992	3.8	26.3	137.7	7.4	63.5	180.3	1.2	9.5	109.5	8.0	53.2	204.0
1993–1997	3.0	23.9	122.4	7.2	53.1	166.4	1.2	9.6	115.2	6.0	34.7	178.3
1998–2002	5.8	40.4	149.6	9.2	65.0	142.9	1.2	11.8	101.9	6.0	33.9	161.6
2003–2007	4.6	38.6	124.3	8.8	65.0	151.6	1.0	12.2	93.6	3.6	23.5	126.2
2008–2012	3.4	34.7	106.4	5.0	43.4	110.0	1.2	14.4	100.2	3.8	24.2	135.9
	35–44			>74 y/o			35–44			>74 y/o		
1983–1987	7.6	44.9	141.2	10.2	175.5	224.0	2.2	13.8	116.5	15.6	178.0	308.6
1988–1992	5.0	28.5	120.2	8.6	126.2	195.6	1.6	10.2	105.9	16.4	149.4	312.2
1993–1997	7.2	40.9	167.0	11.0	136.1	261.3	1.6	10.0	118.1	13.4	101.9	296.2
1998–2002	7.2	51.1	145.2	8.8	92.3	175.0	2.4	14.9	143.8	14.6	88.2	284.1
2003–2007	6.6	55.4	138.2	11.2	91.9	204.0	1.0	11.8	93.7	13.6	67.3	299.8
2008–2012	5.0	42.8	115.0	11.4	81.0	195.3	1.2	13.3	98.1	7.8	34.6	180.0

Secondary Medical Zone ID: 133

	Male						Female					
	Suicide			Suicide			Suicide			Suicide		
	Num per year	Rate /100,000	×100	Num per year	Rate /100,000	×100	Num per year	Rate /100,000	×100	Num per year	Rate /100,000	×100
	Total (>10 y/o)			45–54			Total (>10 y/o)			45–54		
1983–1987	70.4	46.8	155.6	18.0	76.9	152.0	48.8	23.8	182.2	4.6	19.4	112.9
1988–1992	61.0	35.8	169.2	13.0	55.9	162.8	49.0	20.4	199.3	4.4	18.0	123.3
1993–1997	51.2	30.8	136.0	11.8	47.4	129.5	41.6	15.9	192.7	4.8	17.8	137.9
1998–2002	70.8	42.2	130.0	17.2	69.2	123.6	43.2	16.0	173.1	3.0	14.3	94.8
2003–2007	69.8	43.8	131.4	13.6	64.6	115.5	32.6	13.7	143.6	3.2	15.1	110.1
2008–2012	62.0	39.7	127.0	12.2	63.0	129.6	24.6	13.2	117.0	1.4	11.6	77.4
	15–24			55–64			15–24			55–64		
1983–1987	4.0	19.8	142.0	10.0	49.5	116.4	1.4	7.6	111.1	6.6	25.9	131.1
1988–1992	1.0	8.0	80.3	14.8	63.0	177.5	1.8	7.7	145.2	6.4	24.4	136.5
1993–1997	2.2	12.0	106.2	8.2	40.7	101.1	0.2	3.5	66.9	6.2	24.1	151.9
1998–2002	2.2	15.0	95.1	14.2	70.4	110.8	0.4	5.3	74.9	6.2	25.2	133.7
2003–2007	3.8	22.8	130.5	16.2	71.6	124.1	1.4	10.0	110.9	3.6	16.8	100.5
2008–2012	4.2	28.2	133.1	14.0	57.5	118.3	1.8	12.6	130.3	2.6	13.3	89.3
	25–34			65–74			25–34			65–74		
1983–1987	8.0	35.3	138.0	9.6	70.0	160.0	2.0	10.5	97.1	11.0	58.5	187.3
1988–1992	3.8	20.7	108.1	10.8	67.8	192.6	1.8	9.6	111.3	10.4	49.3	188.9
1993–1997	3.8	20.9	107.2	8.6	48.3	151.5	1.2	7.9	95.1	9.4	38.7	198.5
1998–2002	5.6	29.3	108.7	11.8	61.3	134.8	1.6	10.7	92.2	9.8	38.6	183.6
2003–2007	3.6	24.5	78.9	12.0	62.7	146.1	0.6	8.6	65.9	6.6	27.5	147.7
2008–2012	4.8	32.1	98.5	8.8	48.0	121.4	2.0	14.4	100.8	5.0	22.5	126.4
	35–44			>74 y/o			35–44			>74 y/o		
1983–1987	8.0	33.4	104.9	12.6	189.2	241.5	2.8	12.3	103.7	20.4	188.9	327.4
1988–1992	6.2	25.4	107.3	11.4	135.5	210.1	3.6	12.9	133.7	20.6	143.2	299.3
1993–1997	7.2	31.6	129.1	9.0	91.6	175.9	2.4	10.4	122.5	17.4	98.1	284.9
1998–2002	8.0	40.8	115.9	11.6	94.8	179.9	1.6	9.8	94.5	20.6	91.3	294.1
2003–2007	10.6	55.5	138.4	9.6	65.1	144.6	1.4	10.7	84.3	15.8	57.9	258.1
2008–2012	6.6	36.8	98.8	11.4	64.5	155.5	1.4	11.2	82.6	10.4	33.7	175.0

Secondary Medical Zone ID: 134

	Male						Female					
	Suicide			Suicide			Suicide			Suicide		
	Num per year	Rate /100,000	×100	Num per year	Rate /100,000	×100	Num per year	Rate /100,000	×100	Num per year	Rate /100,000	×100
	Total (>10 y/o)			45–54			Total (>10 y/o)			45–54		
1983–1987	17.6	37.5	131.9	4.0	60.0	118.6	9.2	16.0	111.1	1.8	20.4	118.3
1988–1992	11.0	25.6	110.0	1.2	30.0	87.5	10.0	14.2	127.1	1.8	18.7	128.0
1993–1997	15.6	29.7	143.4	2.6	41.5	113.5	7.0	11.4	112.7	1.6	16.9	131.0
1998–2002	14.8	37.2	108.5	2.8	54.6	97.5	12.0	15.1	151.0	1.8	19.6	130.2
2003–2007	19.0	43.2	140.5	5.4	88.6	158.4	6.8	13.2	114.0	1.2	16.6	121.0
2008–2012	13.8	37.0	117.2	2.2	51.1	105.2	5.4	12.5	105.5	0.2	13.3	89.0
	15–24			55–64			15–24			55–64		
1983–1987	0.2	12.0	86.3	3.0	47.0	110.5	0.4	7.5	110.2	1.2	18.6	94.4
1988–1992	0.4	10.3	104.1	2.2	35.7	100.4	0.6	7.1	134.0	0.8	15.6	87.3
1993–1997	1.0	14.2	125.5	4.0	54.0	134.1	0.2	5.4	103.8	0.2	11.5	72.6
1998–2002	1.0	18.9	119.4	2.8	61.0	96.0	0.0	6.2	88.9	2.0	23.3	123.9
2003–2007	0.4	17.4	99.5	4.4	72.1	124.8	0.0	7.8	86.6	2.0	21.6	128.8
2008–2012	0.6	22.4	105.8	2.6	49.1	101.1	0.0	8.5	87.8	0.8	15.3	102.5
	25–34			65–74			25–34			65–74		
1983–1987	1.6	30.0	117.3	2.4	51.6	117.9	0.4	10.6	98.8	2.0	33.3	106.6
1988–1992	1.0	21.9	114.8	1.4	33.6	95.4	0.4	9.2	105.7	2.0	29.4	112.6
1993–1997	0.8	20.8	106.6	2.0	35.5	111.4	0.6	9.8	118.3	1.0	17.3	88.7
1998–2002	0.8	26.6	98.4	3.0	49.8	109.5	1.0	14.5	125.2	2.6	28.4	135.3
2003–2007	2.0	39.5	127.5	3.0	50.8	118.5	0.2	12.2	94.0	0.6	15.6	84.0
2008–2012	1.2	34.7	106.5	2.6	47.2	119.5	0.2	13.6	94.7	0.8	17.4	97.7
	35–44			>74 y/o			35–44			>74 y/o		
1983–1987	2.0	34.6	108.8	4.4	131.3	167.6	0.4	11.0	93.2	3.0	70.7	122.6
1988–1992	1.8	27.9	117.6	3.0	83.2	128.9	0.8	11.0	113.4	3.6	64.2	134.2
1993–1997	1.8	30.3	123.8	3.4	80.6	154.7	0.4	8.8	102.9	3.0	45.6	132.4
1998–2002	2.2	42.7	121.2	2.2	55.3	104.9	0.4	10.5	101.6	4.2	51.4	165.6
2003–2007	1.4	40.4	100.7	2.4	49.5	109.9	0.2	11.8	93.0	2.6	29.4	131.0
2008–2012	1.8	41.5	111.4	2.8	48.0	115.8	0.2	12.8	94.4	3.2	28.8	149.9

Secondary Medical Zone ID: 135

	Male						Female					
	Suicide			Suicide			Suicide			Suicide		
	Num per year	Rate /100,000	×100	Num per year	Rate /100,000	×100	Num per year	Rate /100,000	×100	Num per year	Rate /100,000	×100
	Total (>10 y/o)			45-54			Total (>10 y/o)			45-54		
1983-1987	29.0	41.2	144.0	6.0	59.5	117.6	14.6	17.3	123.1	1.4	15.9	92.2
1988-1992	21.2	28.5	131.3	3.4	35.1	102.2	15.6	15.2	137.1	2.2	17.1	117.1
1993-1997	24.8	31.0	142.1	4.2	38.2	104.4	15.2	13.5	147.9	2.4	16.2	125.5
1998-2002	27.8	37.8	115.0	5.8	56.6	101.2	19.0	15.6	155.5	2.0	16.5	109.8
2003-2007	28.4	39.3	119.1	4.8	56.6	101.2	18.6	15.1	158.8	1.6	15.4	112.2
2008-2012	26.0	37.3	117.7	5.0	57.4	118.0	11.8	13.7	116.4	1.2	15.3	102.8
	15-24			55-64			15-24			55-64		
1983-1987	3.2	25.1	179.8	4.8	53.5	126.0	1.0	8.2	119.4	1.6	18.7	94.6
1988-1992	1.6	12.4	124.9	4.8	46.9	132.1	0.8	6.3	119.6	3.4	24.9	139.2
1993-1997	1.0	11.5	102.1	5.6	53.2	132.1	1.4	8.4	161.7	2.2	18.9	118.7
1998-2002	1.2	16.3	103.3	7.2	73.8	116.2	0.4	6.8	96.9	3.0	23.2	123.2
2003-2007	0.4	14.5	83.2	7.0	65.3	113.2	0.8	10.6	117.3	3.2	21.4	128.0
2008-2012	1.2	21.9	103.1	4.6	47.5	97.8	0.8	11.5	118.5	1.2	13.8	92.7
	25-34			65-74			25-34			65-74		
1983-1987	2.4	26.6	104.2	2.8	50.9	116.3	0.6	9.4	87.7	3.4	42.0	134.4
1988-1992	1.4	18.2	95.5	3.8	52.8	149.9	0.4	7.6	87.5	2.0	26.5	101.7
1993-1997	2.0	21.8	111.5	3.6	44.4	139.2	1.0	9.6	115.0	3.6	31.8	163.2
1998-2002	2.4	27.3	101.1	3.6	46.7	102.7	1.0	11.8	101.9	4.2	33.2	158.0
2003-2007	3.0	33.3	107.5	5.4	58.7	136.8	1.0	13.1	100.8	3.0	24.8	133.3
2008-2012	2.6	34.5	106.0	4.0	45.2	114.3	1.0	15.0	104.5	0.8	14.5	81.4
	35-44			>74 y/o			35-44			>74 y/o		
1983-1987	6.2	46.0	144.4	3.6	119.5	152.5	2.4	15.4	130.0	4.0	82.4	142.9
1988-1992	3.6	29.6	124.7	2.6	73.8	114.5	1.2	10.2	105.7	5.6	80.4	168.0
1993-1997	4.6	39.9	162.9	3.8	80.7	155.0	0.4	7.2	85.1	4.2	51.2	148.8
1998-2002	2.2	31.4	89.1	5.4	90.7	172.1	1.4	12.8	124.1	7.0	66.2	213.3
2003-2007	4.0	46.5	116.1	3.8	57.2	127.0	1.2	13.5	107.2	7.8	58.7	261.7
2008-2012	4.4	43.2	116.0	4.2	52.8	127.3	1.0	13.2	97.6	5.6	36.2	188.0

Secondary Medical Zone ID: 136

	Male						Female					
	Suicide			Suicide			Suicide			Suicide		
	Num per year	Rate /100,000	×100	Num per year	Rate /100,000	×100	Num per year	Rate /100,000	×100	Num per year	Rate /100,000	×100
	Total (>10 y/o)			45–54			Total (>10 y/o)			45–54		
1983–1987	69.6	34.9	108.7	20.2	62.5	123.5	36.8	15.7	103.9	6.0	17.8	103.2
1988–1992	56.4	25.8	106.2	13.4	38.3	111.5	36.6	13.3	107.2	5.2	14.6	99.9
1993–1997	60.8	25.7	104.3	16.6	41.2	112.5	32.2	10.9	104.0	4.2	11.3	87.1
1998–2002	92.6	37.0	106.2	23.0	59.4	106.1	36.8	12.6	100.3	4.4	12.6	83.5
2003–2007	90.6	36.8	102.9	15.6	49.5	88.5	35.6	12.5	101.4	4.2	13.2	96.3
2008–2012	76.6	33.4	94.7	12.0	42.8	88.0	38.6	13.0	109.6	2.2	10.1	67.8
	15–24			55–64			15–24			55–64		
1983–1987	4.6	15.1	108.5	10.8	43.1	101.4	3.0	8.7	126.8	5.4	19.0	96.2
1988–1992	4.2	11.4	115.5	9.4	33.1	93.1	2.2	6.0	114.3	7.2	21.1	118.2
1993–1997	3.2	10.0	88.2	11.6	38.7	96.0	1.4	4.6	88.3	7.8	21.4	134.9
1998–2002	5.2	16.8	106.1	20.8	63.3	99.7	2.6	8.3	118.0	5.6	16.8	89.4
2003–2007	4.6	17.6	100.9	22.2	59.0	102.3	1.2	6.6	73.0	6.8	17.0	101.7
2008–2012	5.0	21.5	101.6	16.6	45.4	93.4	1.2	7.2	74.6	7.2	17.2	115.3
	25–34			65–74			25–34			65–74		
1983–1987	8.2	27.1	105.9	4.6	32.5	74.3	3.0	10.1	93.4	4.8	25.0	80.0
1988–1992	5.6	19.6	102.6	8.0	43.0	122.2	1.2	5.9	68.0	7.4	30.4	116.7
1993–1997	7.2	22.2	113.5	7.4	33.7	105.8	2.8	8.8	105.4	4.8	18.0	92.2
1998–2002	10.6	29.1	107.9	10.8	43.8	96.3	4.0	11.6	100.4	6.8	22.1	105.1
2003–2007	13.4	36.9	119.0	12.8	47.4	110.5	5.8	16.0	123.0	7.2	21.6	116.2
2008–2012	9.2	31.9	98.1	9.6	34.6	87.7	5.8	18.6	130.0	8.6	23.3	130.7
	35–44			>74 y/o			35–44			>74 y/o		
1983–1987	15.0	36.8	115.7	6.2	80.7	103.0	5.2	12.4	105.0	9.2	72.6	125.8
1988–1992	9.8	25.3	106.9	6.0	58.1	90.1	3.2	8.8	90.6	9.6	52.3	109.2
1993–1997	6.8	21.7	88.4	8.0	61.7	118.5	1.8	6.6	77.1	9.0	39.1	113.7
1998–2002	10.6	35.2	99.8	11.2	70.4	133.6	3.2	10.5	101.8	10.0	35.7	114.9
2003–2007	12.4	38.6	96.4	9.6	49.5	109.8	2.8	10.3	81.3	7.6	22.6	100.9
2008–2012	16.0	41.9	112.6	8.2	37.2	89.6	3.8	11.8	87.0	9.8	24.0	124.8

Secondary Medical Zone ID: 137

	Male						Female					
	Suicide			Suicide			Suicide			Suicide		
	Num per year	Rate /100,000	×100	Num per year	Rate /100,000	×100	Num per year	Rate /100,000	×100	Num per year	Rate /100,000	×100
	Total (>10 y/o)			45-54			Total (>10 y/o)			45-54		
1983-1987	49.4	34.4	108.0	13.0	57.7	114.1	30.6	16.8	115.8	5.4	20.6	119.8
1988-1992	39.0	25.3	105.2	8.8	36.5	106.1	28.8	14.3	116.9	4.4	16.4	112.1
1993-1997	42.8	26.0	107.3	10.4	37.3	101.9	23.8	11.2	109.6	2.8	11.2	86.4
1998-2002	66.0	37.9	112.0	18.4	67.0	119.7	28.0	13.2	109.7	4.4	15.7	104.4
2003-2007	66.6	39.0	114.4	10.8	52.4	93.7	27.2	13.4	112.9	4.0	16.2	118.4
2008-2012	55.4	36.2	105.6	6.2	38.2	78.7	23.8	13.5	104.3	1.6	11.5	77.2
	15-24			55-64			15-24			55-64		
1983-1987	3.4	16.5	118.3	10.6	54.2	127.5	1.8	7.8	113.5	4.8	21.6	109.6
1988-1992	0.8	6.7	67.3	10.0	46.0	129.4	2.0	7.1	134.6	6.6	24.7	138.2
1993-1997	1.8	9.7	85.8	10.2	46.8	116.2	1.0	5.0	95.0	3.6	15.6	98.4
1998-2002	3.6	18.0	114.0	17.2	73.8	116.2	1.8	8.5	121.4	5.2	20.4	108.6
2003-2007	2.8	18.2	104.5	20.0	72.9	126.4	1.4	9.5	105.5	4.4	16.4	97.7
2008-2012	3.4	24.1	113.4	11.2	44.9	92.4	1.4	10.6	108.8	4.2	15.3	102.4
	25-34			65-74			25-34			65-74		
1983-1987	6.4	29.8	116.5	2.8	29.0	66.3	1.4	8.2	76.3	6.0	36.5	116.7
1988-1992	3.6	19.4	101.3	4.0	31.7	90.0	1.8	9.0	103.7	4.0	23.6	90.5
1993-1997	3.2	17.7	90.6	5.6	34.9	109.5	1.4	7.7	93.1	5.8	26.7	137.2
1998-2002	4.4	22.6	83.7	10.6	56.7	124.6	2.0	10.5	90.6	5.2	23.2	110.6
2003-2007	7.2	32.9	106.1	9.4	49.7	115.8	3.0	14.0	108.0	5.0	21.4	114.9
2008-2012	7.8	40.3	123.7	7.8	39.8	100.7	3.0	16.4	114.7	5.6	22.1	124.0
	35-44			>74 y/o			35-44			>74 y/o		
1983-1987	9.2	31.7	99.7	4.0	71.9	91.8	4.4	13.8	116.5	6.8	70.3	121.8
1988-1992	7.6	27.1	114.4	4.0	55.3	85.8	3.4	11.1	115.3	6.6	50.3	105.1
1993-1997	5.6	25.8	105.3	6.0	63.3	121.6	1.6	7.8	91.4	7.6	45.5	132.1
1998-2002	4.8	27.9	79.3	6.8	59.7	113.2	1.4	8.7	83.8	8.0	39.0	125.7
2003-2007	7.8	40.3	100.7	8.6	59.2	131.4	1.8	10.9	86.7	7.6	30.2	134.5
2008-2012	11.0	45.5	122.3	7.6	46.3	111.6	3.6	15.4	113.4	4.4	16.5	85.6

Secondary Medical Zone ID: 138

	Male						Female					
	Suicide			Suicide			Suicide			Suicide		
	Num per year	Rate /100,000	×100	Num per year	Rate /100,000	×100	Num per year	Rate /100,000	×100	Num per year	Rate /100,000	×100
	Total (>10 y/o)			45-54			Total (>10 y/o)			45-54		
1983-1987	28.8	39.2	135.3	7.2	68.6	135.7	17.2	18.1	132.1	3.0	22.1	128.2
1988-1992	24.2	29.5	139.4	3.4	35.6	103.7	17.6	15.7	144.5	2.4	17.9	122.2
1993-1997	22.6	28.2	125.0	4.4	38.0	104.0	12.0	11.8	118.6	1.6	13.6	105.4
1998-2002	29.6	38.2	115.1	6.0	53.9	96.3	12.2	13.0	107.7	1.8	15.6	103.5
2003-2007	31.0	39.2	121.5	5.6	58.7	105.0	15.2	14.1	131.5	1.2	13.4	97.8
2008-2012	27.6	37.3	117.6	4.0	49.7	102.3	9.6	12.7	99.9	0.6	12.4	83.3
	15-24			55-64			15-24			55-64		
1983-1987	1.0	13.6	97.8	6.6	60.7	142.9	1.6	10.5	153.9	2.0	19.8	100.3
1988-1992	1.0	10.5	106.4	5.8	50.8	143.0	0.4	5.1	96.9	2.8	21.5	120.2
1993-1997	1.0	11.5	101.7	5.2	50.1	124.4	1.0	7.1	136.1	2.6	20.2	127.3
1998-2002	1.0	15.0	95.2	6.0	65.8	103.6	0.8	8.1	114.8	1.4	17.2	91.4
2003-2007	1.0	17.0	97.7	5.4	53.0	91.8	1.0	11.2	124.8	3.2	21.4	128.0
2008-2012	1.2	21.7	102.4	5.4	48.2	99.3	0.6	10.4	107.0	1.6	14.8	99.2
	25-34			65-74			25-34			65-74		
1983-1987	3.4	32.0	125.1	3.4	53.1	121.3	2.0	15.0	139.4	3.0	36.2	115.8
1988-1992	1.2	17.4	91.2	3.6	46.6	132.2	1.2	10.6	122.5	4.0	39.9	152.8
1993-1997	2.0	22.6	115.8	3.0	36.1	113.2	0.2	6.7	80.9	2.4	23.0	117.8
1998-2002	3.6	34.0	125.9	4.8	52.8	116.0	0.4	9.7	83.5	3.4	27.6	131.2
2003-2007	2.8	32.1	103.4	5.8	60.9	141.9	1.0	12.9	99.1	2.6	22.4	120.3
2008-2012	4.0	41.0	125.8	4.6	49.3	124.9	0.6	12.9	90.0	2.6	21.8	122.7
	35-44			>74 y/o			35-44			>74 y/o		
1983-1987	3.6	31.9	100.3	3.4	98.1	125.2	1.4	12.2	103.4	4.2	75.6	131.0
1988-1992	4.4	32.2	135.9	4.8	102.0	158.1	1.0	9.5	98.5	5.8	76.8	160.4
1993-1997	2.6	26.2	106.9	4.4	80.9	155.3	0.6	7.8	91.5	3.6	42.4	123.0
1998-2002	3.2	37.6	106.7	4.8	73.1	138.7	0.6	9.4	90.5	3.6	35.6	114.6
2003-2007	3.6	44.0	109.7	6.8	78.1	173.4	1.0	12.7	100.7	5.2	38.0	169.4
2008-2012	3.8	40.4	108.7	4.4	49.4	119.2	0.8	12.5	92.2	2.8	20.1	104.3

Secondary Medical Zone ID: 139

	Male						Female					
	Suicide			Suicide			Suicide			Suicide		
	Num per year	Rate /100,000	×100	Num per year	Rate /100,000	×100	Num per year	Rate /100,000	×100	Num per year	Rate /100,000	×100
	Total (>10 y/o)			45-54			Total (>10 y/o)			45-54		
1983-1987	27.8	32.1	96.2	6.2	46.5	92.0	14.8	14.3	91.1	1.8	14.3	83.2
1988-1992	23.4	24.2	97.7	6.6	39.6	115.4	16.0	12.6	101.1	2.6	15.0	102.5
1993-1997	28.6	25.7	107.2	8.2	41.9	114.6	13.8	10.7	97.1	3.0	14.2	109.9
1998-2002	44.8	38.3	111.9	8.8	52.1	93.1	18.4	13.1	106.2	4.0	18.3	121.4
2003-2007	44.6	39.0	110.5	8.8	60.3	107.7	12.0	11.1	81.0	2.0	13.4	98.0
2008-2012	36.6	34.6	99.4	6.6	49.3	101.5	15.2	13.0	97.1	2.0	14.6	97.9
	15-24			55-64			15-24			55-64		
1983-1987	2.8	18.0	129.1	4.4	40.2	94.6	1.0	6.8	100.1	1.8	16.2	82.1
1988-1992	1.2	9.0	91.3	5.4	40.4	113.7	0.4	4.1	77.5	2.0	15.7	87.8
1993-1997	2.0	12.1	106.7	7.0	47.8	118.9	1.2	6.3	120.6	2.4	15.9	99.8
1998-2002	2.2	16.3	103.5	14.8	88.5	139.2	1.8	9.7	138.2	4.0	21.6	114.8
2003-2007	2.4	19.2	109.8	12.8	69.4	120.2	0.6	7.6	84.4	3.0	16.4	97.9
2008-2012	2.6	23.4	110.2	9.0	51.8	106.5	1.2	10.8	111.5	2.0	13.1	87.8
	25-34			65-74			25-34			65-74		
1983-1987	2.2	19.8	77.6	3.4	46.1	105.3	1.6	10.9	101.3	4.6	41.3	132.1
1988-1992	2.6	19.5	101.8	2.0	28.6	81.1	1.0	8.0	92.6	2.2	21.8	83.7
1993-1997	2.2	17.1	87.7	3.8	36.4	114.1	0.6	6.5	78.2	2.0	17.2	88.5
1998-2002	4.6	27.7	102.6	5.2	46.6	102.6	1.6	10.8	93.3	3.4	23.5	111.7
2003-2007	4.0	27.1	87.5	4.8	41.5	96.6	1.4	11.2	85.9	2.2	17.0	91.3
2008-2012	4.6	33.6	103.3	5.2	39.1	99.0	1.6	13.8	96.0	4.0	22.1	124.1
	35-44			>74 y/o			35-44			>74 y/o		
1983-1987	4.8	27.3	85.7	4.0	98.3	125.5	1.8	10.4	88.4	2.2	42.2	73.2
1988-1992	3.2	20.1	84.9	2.4	53.9	83.6	1.6	9.2	95.4	6.2	69.5	145.2
1993-1997	2.4	19.2	78.4	2.8	49.6	95.2	1.6	9.4	110.2	3.0	29.7	86.3
1998-2002	5.0	36.0	102.2	4.2	57.6	109.2	0.6	7.5	72.3	3.0	24.8	79.8
2003-2007	6.8	44.7	111.5	4.8	52.0	115.4	0.6	8.7	69.0	2.2	15.9	70.9
2008-2012	5.0	32.9	88.5	3.6	37.5	90.4	2.4	14.0	103.4	2.0	13.5	70.0

Secondary Medical Zone ID: 140

	Male						Female					
	Suicide			Suicide			Suicide			Suicide		
	Num per year	Rate /100,000	×100	Num per year	Rate /100,000	×100	Num per year	Rate /100,000	×100	Num per year	Rate /100,000	×100
	Total (>10 y/o)			45–54			Total (>10 y/o)			45–54		
1983–1987	78.4	32.5	99.4	20.4	56.4	111.4	38.4	14.2	91.7	6.6	17.4	101.2
1988–1992	58.2	22.0	87.6	11.8	28.7	83.6	37.8	12.1	92.5	7.6	16.7	114.4
1993–1997	64.2	22.1	85.8	15.6	30.7	83.9	25.4	8.2	69.3	4.4	9.6	74.6
1998–2002	101.2	32.4	88.7	25.6	49.7	88.8	39.2	11.6	85.3	7.0	13.8	91.8
2003–2007	109.0	34.0	91.6	24.4	54.8	97.9	41.6	11.5	90.4	6.2	13.6	99.5
2008–2012	103.4	32.0	92.0	17.6	43.0	88.4	39.2	11.6	85.1	4.0	10.8	72.3
	15–24			55–64			15–24			55–64		
1983–1987	6.0	12.9	92.3	11.4	41.7	98.1	2.2	5.4	79.7	5.4	17.6	89.2
1988–1992	5.4	9.6	96.5	11.2	35.0	98.6	2.6	5.0	94.2	6.2	17.5	98.0
1993–1997	5.0	9.0	79.9	13.4	39.0	96.8	1.8	3.8	73.3	4.0	11.9	74.9
1998–2002	7.2	14.0	88.6	25.2	62.9	99.0	3.8	7.8	110.5	6.2	15.9	84.3
2003–2007	7.6	16.5	94.7	28.0	56.9	98.5	3.4	8.5	94.7	7.8	15.7	93.6
2008–2012	6.8	17.5	82.5	25.2	49.8	102.6	2.4	7.5	77.1	6.0	12.4	83.4
	25–34			65–74			25–34			65–74		
1983–1987	9.8	22.8	89.1	7.8	45.7	104.4	5.4	11.6	107.9	7.6	32.7	104.7
1988–1992	7.4	17.6	92.2	6.4	33.0	93.7	3.4	8.0	91.9	7.6	28.2	108.2
1993–1997	7.0	16.0	81.8	8.4	34.3	107.6	3.8	8.1	97.2	3.6	13.2	67.9
1998–2002	13.4	26.1	96.6	11.0	40.6	89.2	6.2	11.7	101.2	4.8	15.7	74.5
2003–2007	15.8	30.8	99.2	10.4	35.5	82.7	7.0	13.4	103.4	5.4	15.9	85.3
2008–2012	13.8	31.7	97.3	14.2	40.1	101.5	6.6	14.8	103.1	6.0	15.8	89.0
	35–44			>74 y/o			35–44			>74 y/o		
1983–1987	15.8	29.5	92.7	6.8	81.3	103.8	6.2	11.5	97.7	4.8	38.0	65.9
1988–1992	10.0	19.5	82.2	5.6	51.3	79.5	4.6	8.9	92.4	5.8	30.4	63.4
1993–1997	9.8	21.7	88.4	4.8	37.2	71.5	3.0	7.0	82.3	4.8	20.3	59.1
1998–2002	13.0	30.6	87.0	5.0	33.2	63.1	4.6	10.3	100.0	6.4	21.5	69.1
2003–2007	16.2	35.8	89.3	6.6	32.7	72.6	3.4	8.8	69.3	8.2	21.4	95.4
2008–2012	17.8	34.4	92.4	8.0	32.4	78.0	8.0	14.7	108.1	6.0	13.9	72.3

Secondary Medical Zone ID: 141

	Male						Female					
	Suicide			Suicide			Suicide			Suicide		
	Num per year	Rate /100,000	×100	Num per year	Rate /100,000	×100	Num per year	Rate /100,000	×100	Num per year	Rate /100,000	×100
	Total (>10 y/o)			45-54			Total (>10 y/o)			45-54		
1983-1987	25.4	35.0	111.3	5.6	52.8	104.5	14.6	15.8	107.3	2.2	18.0	104.7
1988-1992	23.0	28.1	123.8	3.8	35.0	101.9	12.4	12.9	101.4	1.6	14.5	99.1
1993-1997	18.2	24.9	98.2	4.8	37.8	103.2	12.0	11.4	110.5	1.0	11.0	85.4
1998-2002	25.2	35.0	95.6	4.2	41.9	74.9	10.0	11.8	89.3	1.4	13.7	91.0
2003-2007	25.2	35.5	98.1	4.0	45.2	80.9	12.2	13.5	109.0	1.8	15.2	110.9
2008-2012	22.2	34.7	98.8	3.8	47.9	98.6	10.4	13.0	102.7	1.4	15.7	105.0
	15-24			55-64			15-24			55-64		
1983-1987	1.8	17.0	122.0	5.4	51.5	121.2	0.2	5.4	79.2	3.0	22.9	116.0
1988-1992	1.2	10.9	109.8	4.0	38.1	107.2	1.0	6.9	130.8	2.6	19.7	110.3
1993-1997	1.0	11.3	99.8	3.8	39.3	97.6	0.2	4.4	84.0	2.8	20.2	126.9
1998-2002	2.0	19.6	124.3	8.0	73.3	115.4	0.2	6.0	85.5	1.4	16.4	87.2
2003-2007	0.8	16.5	94.4	5.8	51.9	89.9	1.0	11.4	126.2	2.6	18.6	110.9
2008-2012	1.4	23.3	109.7	5.0	44.6	91.7	0.0	7.2	74.4	2.4	16.6	111.6
	25-34			65-74			25-34			65-74		
1983-1987	2.8	27.4	107.3	3.4	49.2	112.4	2.4	16.0	148.8	3.0	33.3	106.5
1988-1992	3.2	28.9	151.4	4.6	52.2	148.1	1.0	9.7	111.5	1.4	18.8	72.0
1993-1997	2.0	22.3	114.0	2.6	32.1	100.6	0.4	7.4	89.3	3.0	24.7	126.8
1998-2002	2.8	30.3	112.2	3.6	43.4	95.3	0.4	9.8	84.6	2.6	22.3	106.3
2003-2007	3.0	34.7	112.0	5.0	51.7	120.4	1.2	14.1	108.3	1.4	16.0	86.2
2008-2012	3.0	38.4	117.9	3.0	36.7	92.9	0.8	14.3	99.9	1.6	17.0	95.6
	35-44			>74 y/o			35-44			>74 y/o		
1983-1987	3.8	30.3	95.1	2.4	73.9	94.3	2.2	14.0	118.8	1.6	36.1	62.7
1988-1992	3.0	23.8	100.5	3.2	72.5	112.4	1.6	10.8	112.2	3.2	44.8	93.5
1993-1997	2.2	22.9	93.3	1.6	38.5	74.0	1.6	11.1	131.1	3.0	33.8	98.3
1998-2002	2.6	32.8	93.1	2.0	40.3	76.5	1.0	10.8	104.8	3.0	28.8	92.6
2003-2007	3.4	42.5	106.1	3.2	44.9	99.6	1.2	13.5	107.1	3.0	23.0	102.5
2008-2012	2.2	33.3	89.5	3.8	45.3	109.1	1.4	14.9	109.6	2.8	19.3	100.2

Secondary Medical Zone ID: 142

	Male						Female					
	Suicide			Suicide			Suicide			Suicide		
	Num per year	Rate /100,000	×100	Num per year	Rate /100,000	×100	Num per year	Rate /100,000	×100	Num per year	Rate /100,000	×100
	Total (>10 y/o)			45–54			Total (>10 y/o)			45–54		
1983–1987	15.2	31.6	95.8	2.8	42.1	83.2	11.6	16.1	111.4	2.2	20.1	116.6
1988–1992	14.2	26.3	112.6	2.2	33.8	98.4	8.6	12.7	98.7	0.4	12.0	82.0
1993–1997	15.2	27.9	119.5	4.0	45.5	124.2	7.4	11.0	101.6	1.4	14.6	112.7
1998–2002	20.2	39.7	117.3	3.4	52.4	93.6	7.0	12.4	93.7	1.0	14.8	98.6
2003–2007	21.0	42.6	130.1	6.4	88.9	158.9	6.4	12.4	97.7	0.6	13.0	94.8
2008–2012	17.2	38.2	121.9	3.4	59.5	122.3	7.0	13.0	107.6	1.2	17.6	118.3
	15–24			55–64			15–24			55–64		
1983–1987	1.0	15.8	113.5	3.4	43.2	101.7	0.2	6.3	92.3	2.8	24.5	124.1
1988–1992	0.2	8.8	89.0	4.4	46.8	131.9	0.4	5.9	111.8	1.4	16.6	93.0
1993–1997	0.8	12.6	111.8	2.6	38.8	96.5	0.6	6.8	129.8	0.8	13.3	83.5
1998–2002	1.2	19.2	121.6	4.8	70.6	111.1	0.8	9.4	133.1	1.2	18.1	96.0
2003–2007	1.0	19.9	114.1	3.4	54.0	93.5	0.2	8.9	98.8	0.8	15.2	90.8
2008–2012	1.0	24.4	114.8	3.8	51.3	105.7	0.0	8.5	87.9	1.2	15.5	103.8
	25–34			65–74			25–34			65–74		
1983–1987	2.6	32.7	127.8	2.8	50.2	114.7	0.4	9.6	89.1	2.0	31.0	99.2
1988–1992	1.2	21.6	112.8	2.0	35.1	99.5	0.8	10.4	119.9	2.2	28.5	109.4
1993–1997	1.4	24.4	125.1	2.4	34.3	107.4	0.2	7.9	94.7	1.2	16.6	85.3
1998–2002	2.0	34.3	127.0	3.2	45.8	100.7	0.0	10.0	86.2	1.0	16.2	77.3
2003–2007	1.6	36.0	116.0	3.8	52.1	121.3	0.2	12.2	93.5	1.2	17.1	91.8
2008–2012	1.8	39.1	120.0	2.4	41.2	104.2	0.0	12.7	88.8	1.6	19.5	109.6
	35–44			>74 y/o			35–44			>74 y/o		
1983–1987	1.2	23.8	74.8	1.4	63.7	81.4	1.2	12.8	108.6	2.8	60.0	103.9
1988–1992	2.6	29.1	122.6	1.6	56.0	86.8	0.8	10.0	103.3	2.6	46.4	96.9
1993–1997	2.4	31.6	128.9	1.6	46.1	88.5	0.8	9.8	115.1	2.4	35.9	104.3
1998–2002	3.2	49.1	139.5	2.4	52.5	99.6	0.8	11.9	114.8	2.2	28.6	92.2
2003–2007	1.8	43.6	108.8	2.8	48.0	106.5	0.8	14.2	112.1	2.6	25.6	113.9
2008–2012	1.4	38.3	103.1	3.2	46.8	112.9	0.2	12.6	92.7	2.8	23.0	119.5

Secondary Medical Zone ID: 143

	Male						Female					
	Suicide			Suicide			Suicide			Suicide		
	Num per year	Rate /100,000	×100	Num per year	Rate /100,000	×100	Num per year	Rate /100,000	×100	Num per year	Rate /100,000	×100
	Total (>10 y/o)			45–54			Total (>10 y/o)			45–54		
1983–1987	57.6	35.3	110.1	14.4	57.4	113.6	27.8	14.7	96.2	3.8	15.5	90.1
1988–1992	43.6	24.8	102.1	9.8	37.1	108.1	27.8	12.6	101.3	3.4	13.4	91.9
1993–1997	43.4	23.8	94.4	10.2	34.8	95.0	23.0	10.1	94.4	3.4	11.9	92.0
1998–2002	68.4	35.5	99.7	16.8	55.8	99.7	27.2	11.5	94.3	2.8	11.5	76.2
2003–2007	66.0	34.3	95.3	14.2	53.7	96.0	27.6	12.2	99.2	3.8	13.8	101.0
2008–2012	60.6	34.5	95.5	11.0	46.0	94.6	23.8	11.9	89.8	3.8	14.9	100.1
	15–24			55–64			15–24			55–64		
1983–1987	4.4	16.3	117.0	7.0	36.5	85.9	1.0	5.2	75.6	5.2	21.3	107.9
1988–1992	2.0	8.3	83.4	8.4	35.9	101.0	1.6	5.6	106.9	4.8	18.5	103.6
1993–1997	4.2	13.1	116.1	7.6	34.3	85.1	1.4	5.3	101.4	3.2	13.6	85.3
1998–2002	3.6	14.6	92.2	16.6	66.5	104.7	1.0	5.6	80.3	5.8	20.7	110.1
2003–2007	3.2	15.5	88.9	16.6	58.4	101.2	2.4	10.6	117.8	4.0	14.8	88.5
2008–2012	6.8	29.3	138.1	12.2	43.7	90.0	1.2	8.0	82.1	4.6	15.1	101.3
	25–34			65–74			25–34			65–74		
1983–1987	5.8	23.1	90.3	6.8	53.0	121.1	2.2	9.0	83.8	6.0	34.3	109.7
1988–1992	3.2	14.5	76.0	5.4	37.8	107.2	1.2	6.3	72.8	6.4	31.5	120.7
1993–1997	3.0	14.2	72.5	6.6	36.2	113.3	1.2	6.2	74.8	4.4	19.8	101.6
1998–2002	6.0	23.9	88.4	9.8	47.6	104.6	2.6	10.5	90.3	6.4	25.1	119.3
2003–2007	5.4	23.4	75.3	7.2	36.6	85.3	2.4	10.8	82.9	4.4	18.3	98.2
2008–2012	5.4	27.4	84.2	8.2	38.3	96.9	2.2	12.0	83.7	3.6	15.8	88.9
	35–44			>74 y/o			35–44			>74 y/o		
1983–1987	11.4	36.1	113.3	7.8	102.5	130.9	3.2	10.9	92.0	6.4	60.0	103.9
1988–1992	7.2	23.6	99.6	7.4	79.9	123.9	3.0	9.7	100.3	7.2	48.4	101.1
1993–1997	7.2	26.0	106.2	4.6	46.8	89.8	1.2	5.9	69.8	7.6	40.5	117.8
1998–2002	8.8	34.7	98.6	6.8	55.3	105.0	1.2	6.8	66.2	7.4	32.6	104.9
2003–2007	9.8	38.9	97.1	9.6	58.2	129.3	2.4	10.6	84.2	8.2	29.0	129.2
2008–2012	10.4	38.6	103.6	6.2	34.5	83.1	3.4	13.1	96.3	5.0	16.3	84.8

Secondary Medical Zone ID: 144

	Male						Female					
	Suicide			Suicide			Suicide			Suicide		
	Num per year	Rate /100,000	×100	Num per year	Rate /100,000	×100	Num per year	Rate /100,000	×100	Num per year	Rate /100,000	×100
	Total (>10 y/o)			45–54			Total (>10 y/o)			45–54		
1983–1987	10.0	32.1	99.3	3.0	55.9	110.6	5.8	15.2	99.9	1.2	18.7	108.7
1988–1992	8.8	24.9	107.5	1.2	31.3	91.1	2.8	11.1	72.7	0.6	14.2	97.2
1993–1997	10.6	26.6	119.2	1.2	32.2	88.1	4.8	10.8	102.5	0.8	13.6	105.5
1998–2002	12.6	36.6	105.4	3.2	58.0	103.7	4.2	12.0	90.4	0.4	13.6	90.3
2003–2007	12.8	37.0	109.4	3.2	63.1	112.9	4.2	12.2	95.3	0.6	13.6	99.2
2008–2012	11.6	34.7	109.1	1.4	45.0	92.7	3.2	12.4	90.2	0.6	15.0	100.5
	15–24			55–64			15–24			55–64		
1983–1987	0.2	11.7	83.6	2.6	48.9	115.2	0.4	7.4	107.9	0.4	15.9	80.3
1988–1992	0.2	9.2	93.2	2.8	45.0	126.8	0.0	4.5	84.9	0.2	13.8	77.2
1993–1997	1.2	14.6	129.3	2.6	46.9	116.5	0.2	5.2	100.4	0.6	14.7	92.7
1998–2002	0.8	17.2	109.0	2.8	64.6	101.8	0.2	6.9	98.3	0.8	18.5	98.2
2003–2007	0.2	15.7	90.0	2.4	54.3	94.1	0.0	7.6	84.0	0.6	16.0	95.3
2008–2012	0.6	21.7	102.3	2.4	47.7	98.2	0.0	8.3	85.5	0.4	13.6	91.4
	25–34			65–74			25–34			65–74		
1983–1987	1.4	26.9	105.3	0.4	33.3	76.1	0.4	10.2	95.1	1.8	38.9	124.3
1988–1992	0.4	16.3	85.2	2.6	53.9	153.0	0.2	8.0	92.2	0.0	14.6	56.0
1993–1997	1.0	22.1	113.3	1.8	38.2	119.7	0.6	9.6	115.5	1.0	19.9	102.1
1998–2002	1.0	27.8	102.8	2.2	48.0	105.5	0.2	10.8	93.2	1.2	22.0	104.5
2003–2007	1.4	34.1	109.9	1.4	40.0	93.1	0.4	13.0	100.0	1.2	20.6	110.7
2008–2012	0.6	30.3	93.0	2.4	47.6	120.5	0.2	13.4	93.3	0.0	13.9	78.4
	35–44			>74 y/o			35–44			>74 y/o		
1983–1987	0.8	25.4	79.6	1.6	89.1	113.8	0.6	11.9	100.4	1.0	48.7	84.4
1988–1992	0.2	16.0	67.6	1.4	69.4	107.6	0.4	9.3	96.4	1.4	46.4	96.9
1993–1997	1.0	23.2	94.8	1.6	62.5	120.1	0.2	7.6	89.5	1.4	35.8	104.1
1998–2002	1.4	35.7	101.2	1.2	49.7	94.4	0.0	8.4	80.8	1.4	30.1	96.8
2003–2007	1.0	36.5	91.2	3.2	68.5	152.2	0.2	11.5	91.2	1.2	21.3	94.9
2008–2012	1.2	37.4	100.6	3.0	57.2	137.9	0.6	14.3	105.4	1.4	20.3	105.4

Secondary Medical Zone ID: 145

	Male						Female					
	Suicide			Suicide			Suicide			Suicide		
	Num per year	Rate /100,000	×100	Num per year	Rate /100,000	×100	Num per year	Rate /100,000	×100	Num per year	Rate /100,000	×100
	Total (>10 y/o)			45–54			Total (>10 y/o)			45–54		
1983–1987	22.0	30.8	89.5	5.6	48.7	96.3	12.6	14.3	91.9	1.6	15.3	88.8
1988–1992	20.8	24.6	101.4	4.6	36.7	106.9	12.2	12.1	94.0	1.0	12.1	83.0
1993–1997	20.8	24.6	96.3	5.8	39.8	108.8	13.4	11.3	110.6	1.6	12.5	96.5
1998–2002	30.2	34.2	94.4	7.4	53.5	95.6	12.6	11.9	93.6	1.4	13.0	86.2
2003–2007	38.2	38.9	115.0	10.0	74.7	133.7	13.8	12.6	103.7	0.8	10.8	78.6
2008–2012	30.6	34.9	101.7	3.8	40.2	82.7	11.6	12.0	94.3	1.6	14.6	97.8
	15–24			55–64			15–24			55–64		
1983–1987	2.8	19.6	140.9	2.6	32.0	75.3	0.4	5.6	81.7	1.6	16.5	83.7
1988–1992	1.0	9.3	93.7	4.0	34.9	98.2	1.2	6.9	131.0	1.4	14.7	82.3
1993–1997	1.4	11.5	101.3	3.6	35.2	87.3	0.4	4.6	87.4	2.6	18.7	117.4
1998–2002	1.0	13.1	83.2	7.4	63.6	100.1	0.6	6.6	93.6	3.0	21.3	113.2
2003–2007	1.6	17.3	98.9	8.4	61.7	106.8	0.8	8.8	97.8	2.0	16.0	95.6
2008–2012	2.8	25.8	121.5	5.6	43.7	89.9	0.8	9.6	99.4	2.2	15.2	101.9
	25–34			65–74			25–34			65–74		
1983–1987	2.0	20.3	79.4	3.6	51.8	118.4	0.6	8.2	75.9	3.4	36.5	116.8
1988–1992	1.8	17.4	91.0	3.8	45.9	130.4	1.2	9.4	108.6	2.6	26.4	101.1
1993–1997	2.2	19.6	100.5	1.0	18.9	59.4	0.6	7.3	87.4	2.4	20.7	106.4
1998–2002	2.8	24.7	91.5	3.4	38.3	84.2	1.0	10.5	90.3	2.6	21.6	102.7
2003–2007	3.2	27.9	90.0	5.0	47.3	110.2	2.0	14.7	112.7	2.4	19.7	105.6
2008–2012	3.8	34.2	105.0	4.8	43.5	110.1	0.6	11.2	78.4	1.8	17.0	95.3
	35–44			>74 y/o			35–44			>74 y/o		
1983–1987	2.4	22.2	69.7	3.0	80.4	102.6	2.2	13.6	115.3	2.6	49.5	85.9
1988–1992	2.0	17.6	74.1	3.6	74.1	114.9	0.4	7.0	72.8	4.4	55.6	116.2
1993–1997	3.0	24.1	98.1	3.6	63.4	121.8	1.6	10.3	121.3	4.2	42.3	122.9
1998–2002	3.6	32.8	93.0	4.6	66.3	125.8	0.2	6.9	66.4	3.8	32.2	103.6
2003–2007	3.8	35.1	87.5	6.2	66.4	147.4	1.6	13.2	104.3	4.2	28.3	126.2
2008–2012	5.8	42.3	113.6	4.0	41.7	100.6	0.8	11.1	81.7	3.8	22.5	117.0

Secondary Medical Zone ID: 146

	Male						Female					
	Suicide			Suicide			Suicide			Suicide		
	Num per year	Rate /100,000	×100	Num per year	Rate /100,000	×100	Num per year	Rate /100,000	×100	Num per year	Rate /100,000	×100
	Total (>10 y/o)			45–54			Total (>10 y/o)			45–54		
1983–1987	18.2	30.2	88.2	3.6	41.1	81.2	7.0	12.7	71.3	0.6	12.5	72.5
1988–1992	14.0	22.3	84.3	2.6	28.9	84.2	10.0	12.4	95.5	1.0	13.1	89.9
1993–1997	15.8	23.9	90.7	3.2	32.2	88.0	9.0	10.5	97.6	1.6	13.7	106.2
1998–2002	25.4	34.7	97.8	6.4	55.2	98.7	7.8	11.1	81.1	1.2	13.7	91.2
2003–2007	23.0	34.9	89.8	6.2	57.6	103.0	9.8	12.3	97.9	1.2	13.3	97.0
2008–2012	24.0	34.2	100.4	4.4	46.4	95.6	7.0	12.2	84.4	0.4	11.3	75.8
	15–24			55–64			15–24			55–64		
1983–1987	1.0	12.8	91.8	3.6	41.3	97.1	0.2	5.7	83.3	1.0	15.1	76.7
1988–1992	0.2	7.6	77.2	3.4	34.4	96.8	1.2	7.8	148.1	2.2	19.2	107.5
1993–1997	0.4	9.3	81.8	3.6	39.5	98.0	0.2	4.4	85.1	1.6	15.9	100.1
1998–2002	1.0	14.8	93.7	9.2	86.7	136.5	0.0	5.2	74.7	2.0	19.5	103.4
2003–2007	0.8	15.7	90.1	6.0	57.7	99.9	0.2	7.2	79.9	1.4	15.6	93.1
2008–2012	1.8	23.6	111.3	4.8	45.5	93.7	1.0	12.0	124.2	0.8	12.5	83.6
	25–34			65–74			25–34			65–74		
1983–1987	2.2	22.4	87.7	2.6	47.8	109.1	0.8	9.7	90.4	1.6	26.4	84.5
1988–1992	1.8	18.0	94.2	1.0	25.6	72.6	0.4	7.2	83.4	1.6	22.1	84.6
1993–1997	2.4	22.2	113.9	2.0	28.8	90.3	0.4	7.2	86.6	2.0	20.5	105.3
1998–2002	2.2	25.3	93.6	3.4	42.5	93.5	0.4	9.4	81.4	1.6	18.5	88.2
2003–2007	2.2	27.4	88.5	2.0	32.8	76.4	1.2	13.4	103.1	2.0	19.7	105.8
2008–2012	2.6	32.4	99.6	3.8	44.1	111.7	0.6	12.6	87.7	1.2	16.1	90.7
	35–44			>74 y/o			35–44			>74 y/o		
1983–1987	3.0	28.1	88.2	2.2	76.2	97.4	0.8	10.2	86.3	2.0	46.6	80.7
1988–1992	2.8	22.9	96.6	2.2	63.1	97.8	1.4	10.7	111.1	2.2	39.2	81.9
1993–1997	2.6	24.0	97.8	1.6	44.2	84.8	0.8	8.4	98.5	2.4	33.1	96.2
1998–2002	1.0	19.9	56.6	2.2	47.8	90.6	0.4	8.3	80.1	2.2	25.7	82.7
2003–2007	4.0	41.9	104.6	1.8	34.0	75.5	1.0	12.3	97.5	2.8	24.2	107.8
2008–2012	3.0	35.0	94.1	3.6	45.0	108.6	1.2	13.6	100.4	1.8	15.8	82.2

Secondary Medical Zone ID: 147

	Male						Female					
	Suicide			Suicide			Suicide			Suicide		
	Num per year	Rate /100,000	×100	Num per year	Rate /100,000	×100	Num per year	Rate /100,000	×100	Num per year	Rate /100,000	×100
	Total (>10 y/o)			45–54			Total (>10 y/o)			45–54		
1983–1987	56.2	32.7	99.7	14.2	52.5	103.9	23.4	12.9	79.6	3.4	14.1	81.7
1988–1992	48.8	25.2	102.7	11.8	38.9	113.2	21.2	10.7	75.9	6.0	18.2	124.4
1993–1997	53.6	25.4	101.9	16.8	47.0	128.4	24.6	10.3	93.5	4.8	14.0	108.5
1998–2002	78.6	35.5	99.7	21.4	61.7	110.3	22.0	10.2	73.4	3.8	12.8	84.9
2003–2007	90.0	40.0	110.3	21.8	69.1	123.5	25.6	11.6	85.4	2.6	10.6	77.2
2008–2012	78.4	36.0	103.0	15.4	51.5	106.0	29.6	13.0	96.7	3.4	13.1	87.6
	15–24			55–64			15–24			55–64		
1983–1987	5.4	17.1	122.5	8.8	41.1	96.7	0.8	4.7	68.4	3.2	15.1	76.6
1988–1992	2.6	8.5	85.9	8.6	34.8	98.0	1.6	5.3	100.8	2.4	12.0	67.3
1993–1997	4.2	11.9	105.1	10.0	38.9	96.5	1.4	4.9	94.0	2.8	12.0	75.3
1998–2002	4.0	14.1	89.3	20.4	71.0	111.7	1.6	6.5	92.3	3.2	13.9	73.6
2003–2007	4.6	17.4	99.8	21.4	65.3	113.2	1.8	8.2	91.0	5.2	16.2	96.8
2008–2012	4.6	20.1	95.0	17.2	51.4	105.8	2.8	11.8	122.2	5.4	15.6	104.4
	25–34			65–74			25–34			65–74		
1983–1987	8.6	29.5	115.4	4.8	38.0	86.7	3.2	11.2	104.1	3.8	23.5	75.1
1988–1992	5.6	19.5	101.9	3.8	27.3	77.5	1.8	7.4	85.1	3.2	17.6	67.3
1993–1997	6.4	20.1	102.9	4.4	25.0	78.5	2.4	8.2	98.9	4.2	18.2	93.6
1998–2002	7.0	22.4	83.0	8.8	41.5	91.2	2.8	10.1	87.0	4.2	17.7	84.3
2003–2007	12.0	35.9	115.8	8.8	38.8	90.5	4.2	13.8	105.9	3.0	13.6	72.9
2008–2012	9.8	35.0	107.5	10.8	41.7	105.6	3.4	13.8	96.3	4.0	15.5	87.2
	35–44			>74 y/o			35–44			>74 y/o		
1983–1987	11.4	33.8	106.1	3.0	48.8	62.3	4.4	12.8	108.4	4.6	42.7	73.9
1988–1992	9.8	27.7	117.0	6.6	69.4	107.6	2.4	8.2	84.8	3.8	27.1	56.6
1993–1997	6.2	20.6	84.1	5.6	50.6	97.1	3.0	9.4	110.6	6.0	31.0	90.1
1998–2002	9.6	32.0	91.0	7.2	52.8	100.2	1.6	7.3	70.5	4.8	21.1	67.8
2003–2007	14.6	44.6	111.2	6.2	37.2	82.6	4.4	13.9	110.1	4.4	15.8	70.3
2008–2012	15.8	43.9	118.0	4.8	26.6	64.1	5.2	15.3	112.9	5.4	16.3	84.7

Secondary Medical Zone ID: 148

	Male						Female					
	Suicide			Suicide			Suicide			Suicide		
	Num per year	Rate /100,000	×100	Num per year	Rate /100,000	×100	Num per year	Rate /100,000	×100	Num per year	Rate /100,000	×100
	Total (>10 y/o)			45–54			Total (>10 y/o)			45–54		
1983–1987	22.0	35.3	114.8	5.4	58.7	116.1	13.6	16.9	117.6	0.8	14.2	82.4
1988–1992	18.4	26.8	114.4	3.6	37.4	108.9	12.4	13.9	114.9	2.8	19.8	135.2
1993–1997	18.0	26.0	104.9	4.2	38.7	105.9	11.0	12.0	114.2	2.2	15.9	123.4
1998–2002	25.2	35.9	100.5	7.2	63.2	113.0	13.2	13.6	115.1	2.2	17.2	114.1
2003–2007	26.0	37.9	102.9	7.6	72.2	129.1	9.8	12.8	97.9	1.4	14.1	102.7
2008–2012	22.4	34.0	98.5	3.0	41.0	84.3	10.2	13.0	102.5	2.0	17.7	118.9
	15–24			55–64			15–24			55–64		
1983–1987	1.0	13.4	96.4	3.0	40.4	95.1	0.8	7.6	111.0	1.6	19.1	97.0
1988–1992	0.8	9.8	98.8	2.4	31.0	87.2	1.4	8.4	158.4	1.6	17.8	99.6
1993–1997	0.6	10.0	88.4	3.8	41.8	103.8	1.2	7.7	147.1	2.2	19.4	122.0
1998–2002	2.0	19.3	121.9	4.2	52.6	82.8	0.6	7.2	102.4	2.2	20.4	108.2
2003–2007	1.6	19.4	111.2	5.8	57.3	99.3	1.2	11.6	129.2	0.8	13.6	81.1
2008–2012	0.8	18.6	87.8	4.6	46.0	94.8	0.6	9.7	99.9	1.2	13.7	91.8
	25–34			65–74			25–34			65–74		
1983–1987	3.2	31.9	125.0	2.2	43.2	98.6	3.0	19.8	184.1	2.6	34.8	111.4
1988–1992	1.6	19.7	103.0	2.0	34.4	97.7	0.6	8.3	95.4	2.2	27.2	104.4
1993–1997	2.0	21.3	109.2	2.2	31.5	98.8	1.4	10.7	128.4	2.0	21.6	111.0
1998–2002	2.8	28.3	104.8	2.2	36.2	79.6	0.6	9.9	85.6	2.6	25.1	119.3
2003–2007	3.0	32.9	106.1	3.2	41.5	96.6	2.0	16.4	126.0	2.2	21.2	113.7
2008–2012	2.8	35.5	108.9	4.2	45.1	114.3	0.6	12.7	88.3	2.2	20.1	113.0
	35–44			>74 y/o			35–44			>74 y/o		
1983–1987	5.2	44.1	138.5	2.0	69.0	88.1	1.6	13.4	113.6	3.2	62.9	109.1
1988–1992	4.6	35.4	149.4	3.2	75.2	116.6	0.6	8.6	88.6	3.2	49.0	102.4
1993–1997	3.0	28.4	115.9	2.2	49.8	95.6	0.6	7.8	91.9	1.4	21.7	63.1
1998–2002	3.0	34.2	97.0	3.8	63.9	121.1	1.6	13.0	125.7	3.4	34.6	111.4
2003–2007	3.0	35.5	88.6	1.8	33.8	75.1	0.4	9.9	78.6	1.6	16.6	74.1
2008–2012	4.2	40.5	108.7	2.8	39.0	94.1	1.2	13.3	98.4	2.4	19.5	101.5

Secondary Medical Zone ID: 149

	Male						Female					
	Suicide			Suicide			Suicide			Suicide		
	Num per year	Rate /100,000	×100	Num per year	Rate /100,000	×100	Num per year	Rate /100,000	×100	Num per year	Rate /100,000	×100
	Total (>10 y/o)			45–54			Total (>10 y/o)			45–54		
1983–1987	16.0	37.7	130.8	3.2	57.4	113.6	7.4	15.8	105.2	1.8	21.9	127.4
1988–1992	10.4	26.3	113.6	1.6	34.5	100.4	6.2	12.8	101.6	1.2	16.8	114.9
1993–1997	11.0	27.0	117.8	2.6	42.7	116.8	4.8	10.6	97.5	0.2	11.3	87.3
1998–2002	14.8	37.8	116.1	3.6	64.0	114.3	3.2	11.3	79.0	0.4	14.0	92.7
2003–2007	11.0	36.4	97.1	2.0	50.9	91.0	6.6	13.7	117.3	0.8	14.8	108.3
2008–2012	13.0	36.5	118.0	1.8	48.5	99.8	4.0	12.6	97.8	0.4	14.3	95.8
	15–24			55–64			15–24			55–64		
1983–1987	1.2	17.6	126.2	2.0	40.5	95.2	0.0	5.6	82.2	1.0	18.4	93.4
1988–1992	0.2	9.0	90.6	1.6	33.2	93.5	0.0	4.4	84.0	1.6	20.9	117.0
1993–1997	0.6	11.9	105.6	2.2	43.1	107.0	0.2	5.3	101.0	0.6	14.8	93.3
1998–2002	0.6	16.1	101.9	3.6	72.4	113.9	0.0	6.2	87.5	0.2	15.8	83.8
2003–2007	0.6	17.9	102.8	2.8	59.9	103.7	0.2	8.7	97.2	1.4	19.5	116.2
2008–2012	1.0	24.3	114.7	1.8	45.3	93.3	0.0	8.3	85.5	1.2	17.1	114.7
	25–34			65–74			25–34			65–74		
1983–1987	3.0	39.2	153.3	1.6	45.6	104.3	1.2	14.7	136.2	1.6	32.9	105.2
1988–1992	1.8	27.4	143.2	1.4	36.1	102.5	0.0	7.2	83.0	1.2	24.6	94.3
1993–1997	1.4	24.4	124.9	2.0	38.0	119.0	0.8	10.5	126.7	0.8	17.2	88.0
1998–2002	0.8	26.1	96.7	2.8	52.3	114.9	0.2	10.9	93.9	0.6	17.5	83.2
2003–2007	1.4	34.4	111.0	1.8	43.6	101.6	0.8	15.2	117.2	0.6	17.0	91.3
2008–2012	0.8	32.6	99.9	2.4	47.2	119.5	0.2	13.8	96.1	0.2	15.0	84.4
	35–44			>74 y/o			35–44			>74 y/o		
1983–1987	2.6	40.0	125.8	2.4	97.9	125.0	0.4	11.1	94.2	1.4	47.2	81.9
1988–1992	2.0	29.8	125.7	1.8	70.2	108.8	0.4	9.5	98.5	1.8	44.8	93.7
1993–1997	1.0	23.7	96.8	1.2	48.1	92.4	0.4	8.8	103.0	1.8	35.8	104.0
1998–2002	1.2	33.6	95.4	2.2	60.3	114.4	0.0	8.5	82.5	1.8	30.5	98.1
2003–2007	1.4	40.1	100.1	1.0	34.7	77.0	1.0	15.3	121.4	1.8	24.7	109.9
2008–2012	2.2	44.1	118.5	3.0	54.4	131.1	0.6	14.4	106.1	1.4	19.2	100.1

Secondary Medical Zone ID: 150

	Male						Female					
	Suicide			Suicide			Suicide			Suicide		
	Num per year	Rate /100,000	×100	Num per year	Rate /100,000	×100	Num per year	Rate /100,000	×100	Num per year	Rate /100,000	×100
	Total (>10 y/o)			45–54			Total (>10 y/o)			45–54		
1983–1987	24.2	31.5	96.3	4.2	39.1	77.4	16.6	16.9	116.8	3.8	23.5	136.4
1988–1992	26.6	28.3	123.5	6.0	43.2	125.7	18.2	15.2	134.1	2.6	17.1	117.1
1993–1997	26.8	27.0	114.1	6.4	41.5	113.5	15.8	12.6	128.7	1.8	13.2	102.1
1998–2002	35.8	36.7	104.8	9.8	62.1	111.0	15.0	13.0	107.4	1.8	14.2	94.5
2003–2007	43.4	42.3	124.0	9.4	64.4	115.2	16.2	13.6	116.5	2.2	15.0	109.4
2008–2012	39.2	38.9	120.5	8.8	61.4	126.3	17.2	14.1	122.0	1.6	14.0	94.2
	15–24			55–64			15–24			55–64		
1983–1987	2.2	15.0	107.5	5.0	46.4	109.1	1.2	7.3	106.3	2.0	18.7	94.6
1988–1992	1.0	8.7	87.9	5.4	42.8	120.5	0.8	5.3	100.1	4.2	25.7	144.0
1993–1997	2.0	12.6	111.2	5.8	46.0	114.2	0.6	4.8	92.2	3.2	21.0	131.9
1998–2002	1.8	15.1	95.8	10.2	78.5	123.5	1.4	8.5	120.4	2.6	19.8	105.0
2003–2007	2.2	17.9	102.6	9.0	64.7	112.0	0.8	7.9	88.1	3.2	19.8	118.4
2008–2012	3.4	26.0	122.7	6.8	47.7	98.3	1.4	11.0	113.0	3.2	18.3	122.4
	25–34			65–74			25–34			65–74		
1983–1987	3.4	25.2	98.4	3.6	55.4	126.4	2.2	13.6	125.9	2.4	31.1	99.4
1988–1992	4.4	27.6	144.4	3.0	40.0	113.5	2.4	12.8	147.4	2.8	29.0	111.2
1993–1997	3.0	20.8	106.5	4.8	46.3	145.1	1.4	9.2	111.0	3.0	24.7	126.9
1998–2002	3.8	28.0	103.7	3.2	37.4	82.2	1.8	12.6	109.1	2.2	19.9	94.8
2003–2007	4.8	37.0	119.4	5.6	50.5	117.7	1.6	13.6	104.9	2.0	17.9	95.9
2008–2012	4.6	39.3	120.5	6.4	51.7	130.8	1.2	14.0	98.0	2.6	19.7	111.0
	35–44			>74 y/o			35–44			>74 y/o		
1983–1987	3.8	28.9	90.7	1.8	66.9	85.4	1.6	12.0	101.4	3.4	68.3	118.4
1988–1992	4.6	28.1	118.4	2.2	60.9	94.4	2.0	11.6	119.7	3.4	53.3	111.4
1993–1997	3.8	25.6	104.4	1.0	32.6	62.6	2.0	11.3	133.2	3.8	45.2	131.3
1998–2002	4.0	31.3	88.8	3.0	52.3	99.2	0.6	7.9	76.4	4.6	42.6	137.2
2003–2007	7.6	53.1	132.5	4.8	57.3	127.2	1.8	13.4	105.7	4.4	32.1	142.9
2008–2012	5.6	41.4	111.2	3.6	40.5	97.6	2.4	16.1	118.9	4.8	28.6	148.7

Secondary Medical Zone ID: 151

	Male						Female					
	Suicide			Suicide			Suicide			Suicide		
	Num per year	Rate /100,000	×100	Num per year	Rate /100,000	×100	Num per year	Rate /100,000	×100	Num per year	Rate /100,000	×100
	Total (>10 y/o)			45-54			Total (>10 y/o)			45-54		
1983-1987	25.6	31.3	91.2	5.8	47.0	93.0	14.0	14.2	89.3	2.0	16.3	94.7
1988-1992	21.6	23.8	93.5	4.0	32.0	93.2	12.6	11.6	86.7	1.4	13.2	90.3
1993-1997	22.4	24.2	91.6	4.2	31.6	86.4	14.4	11.5	106.9	2.0	13.6	105.0
1998-2002	34.6	35.4	94.7	7.8	51.3	91.6	15.2	12.2	99.0	2.8	16.9	112.2
2003-2007	36.0	36.6	96.9	7.8	54.2	97.0	12.8	11.7	91.2	1.8	13.5	98.4
2008-2012	30.2	33.0	88.8	5.2	42.2	86.9	17.2	14.1	112.6	2.8	18.0	121.0
	15-24			55-64			15-24			55-64		
1983-1987	2.4	17.8	127.4	3.2	31.4	73.8	0.8	7.0	102.1	1.6	15.0	76.1
1988-1992	2.0	12.4	125.2	4.4	33.9	95.4	0.4	4.6	87.6	1.8	15.2	85.2
1993-1997	2.6	15.1	133.9	4.2	35.8	89.0	0.0	3.4	64.9	2.8	18.6	117.2
1998-2002	2.4	18.2	115.1	6.6	55.0	86.6	0.6	6.5	93.2	2.4	18.7	99.1
2003-2007	2.4	20.2	115.6	8.0	55.3	95.8	0.6	7.9	88.0	2.4	16.7	99.9
2008-2012	2.8	25.4	119.8	6.2	42.4	87.2	1.0	10.2	105.5	2.6	15.8	105.7
	25-34			65-74			25-34			65-74		
1983-1987	5.8	36.4	142.4	1.8	30.9	70.5	1.0	9.4	87.3	2.4	26.2	83.9
1988-1992	3.0	21.9	114.8	1.8	25.5	72.5	0.6	7.2	83.4	2.2	20.6	78.9
1993-1997	3.2	22.7	116.1	1.8	21.3	66.9	2.0	11.6	138.9	2.8	20.3	104.2
1998-2002	5.8	35.6	131.7	4.6	40.9	90.0	1.6	12.1	104.5	2.8	20.6	98.2
2003-2007	3.8	30.1	96.9	3.2	32.7	76.2	1.2	11.7	90.2	2.4	18.5	99.3
2008-2012	3.4	31.2	95.7	4.4	37.7	95.4	2.0	16.0	111.4	2.4	18.1	101.9
	35-44			>74 y/o			35-44			>74 y/o		
1983-1987	4.0	29.0	91.0	2.6	61.7	78.8	1.6	11.7	99.3	4.6	63.1	109.3
1988-1992	3.2	21.7	91.5	3.2	58.1	90.1	1.4	9.7	100.7	4.8	50.8	106.1
1993-1997	3.8	25.7	104.9	2.6	42.7	82.1	1.6	10.0	117.6	3.0	27.7	80.5
1998-2002	5.0	36.6	103.9	2.4	36.0	68.3	0.2	6.6	64.0	4.8	33.4	107.6
2003-2007	6.4	46.2	115.4	4.2	41.9	93.0	0.6	9.5	74.8	3.8	22.2	99.0
2008-2012	4.8	35.8	96.3	3.4	32.3	77.8	2.2	14.8	109.3	4.2	21.2	110.1

Secondary Medical Zone ID: 152

	Male						Female					
	Suicide			Suicide			Suicide			Suicide		
	Num per year	Rate /100,000	×100	Num per year	Rate /100,000	×100	Num per year	Rate /100,000	×100	Num per year	Rate /100,000	×100
	Total (>10 y/o)			45–54			Total (>10 y/o)			45–54		
1983–1987	25.0	31.4	93.7	5.0	43.7	86.3	16.8	15.9	108.5	1.8	15.7	91.0
1988–1992	25.2	26.6	111.4	7.0	46.7	135.9	18.8	14.5	125.6	2.6	16.7	114.3
1993–1997	22.4	24.6	93.9	6.4	40.4	110.4	13.2	10.8	103.3	2.0	13.3	102.8
1998–2002	35.0	35.7	98.6	8.2	55.4	99.0	20.8	15.2	129.4	5.4	24.5	162.6
2003–2007	30.6	33.5	87.9	5.0	42.7	76.3	16.6	13.6	113.0	0.8	10.6	77.4
2008–2012	31.6	34.7	97.7	5.8	49.0	100.7	13.4	13.7	99.2	2.6	17.8	119.5
	15–24			55–64			15–24			55–64		
1983–1987	2.2	16.1	115.6	3.0	31.6	74.3	1.4	8.5	125.1	2.4	18.7	94.8
1988–1992	2.4	13.0	131.6	3.2	28.8	81.1	1.0	6.2	117.4	4.4	25.0	140.3
1993–1997	1.6	11.5	101.6	2.8	29.1	72.4	0.0	3.3	63.3	2.6	17.9	112.9
1998–2002	3.8	22.7	143.9	7.0	56.8	89.4	1.8	10.1	144.4	3.6	22.5	119.3
2003–2007	1.6	16.6	95.1	7.4	53.1	92.0	0.8	8.7	96.7	3.4	19.6	116.9
2008–2012	1.8	20.5	96.5	5.2	40.7	83.9	1.0	10.5	108.6	2.8	16.6	111.1
	25–34			65–74			25–34			65–74		
1983–1987	2.6	23.0	89.8	2.8	41.4	94.4	0.6	8.2	76.0	3.4	34.3	109.9
1988–1992	1.6	15.8	82.8	2.6	32.5	92.2	1.2	9.3	107.5	3.6	30.3	116.0
1993–1997	3.2	22.9	117.3	2.8	28.7	89.9	0.6	7.1	84.9	1.8	16.1	82.7
1998–2002	5.0	32.4	120.1	3.2	34.7	76.3	1.6	12.1	104.2	3.0	22.2	105.5
2003–2007	4.6	34.0	109.5	3.0	32.5	75.8	2.2	15.3	117.7	2.6	19.7	105.6
2008–2012	3.8	34.3	105.3	3.6	34.0	86.2	2.0	16.4	114.6	1.0	13.3	74.9
	35–44			>74 y/o			35–44			>74 y/o		
1983–1987	5.8	36.8	115.5	3.4	82.7	105.5	2.2	13.3	113.0	5.0	78.0	135.2
1988–1992	4.4	27.6	116.4	3.8	73.3	113.6	1.2	9.1	94.7	4.8	58.1	121.5
1993–1997	4.0	27.9	114.0	1.4	32.1	61.6	1.0	8.2	96.2	5.0	46.8	136.1
1998–2002	3.8	32.1	91.2	4.0	53.2	101.0	1.4	10.8	104.8	4.0	31.4	101.0
2003–2007	4.4	36.9	92.0	4.6	47.7	106.0	2.6	16.1	127.6	4.2	26.1	116.2
2008–2012	7.2	46.3	124.4	4.2	39.5	95.2	1.8	13.8	102.1	2.2	14.5	75.3

Secondary Medical Zone ID: 153

	Male						Female					
	Suicide			Suicide			Suicide			Suicide		
	Num per year	Rate /100,000	×100	Num per year	Rate /100,000	×100	Num per year	Rate /100,000	×100	Num per year	Rate /100,000	×100
	Total (>10 y/o)			45-54			Total (>10 y/o)			45-54		
1983-1987	31.0	34.4	109.2	8.8	60.8	120.2	15.6	15.2	99.4	2.2	16.6	96.1
1988-1992	21.2	23.8	93.8	5.4	35.7	104.1	15.8	13.1	106.7	2.0	14.1	96.2
1993-1997	21.0	22.8	87.4	3.4	27.0	73.9	12.6	10.8	98.3	2.8	15.0	115.8
1998-2002	29.0	32.0	82.1	6.2	45.9	82.0	15.6	12.8	102.7	2.6	16.1	106.7
2003-2007	36.6	38.0	99.8	8.4	64.0	114.4	12.4	11.9	91.1	0.8	10.7	78.2
2008-2012	28.8	33.1	88.9	5.6	48.1	99.1	14.6	13.0	103.9	0.8	11.7	78.7
	15-24			55-64			15-24			55-64		
1983-1987	2.2	16.2	116.3	7.0	55.3	130.0	0.8	6.6	97.2	1.6	15.9	80.5
1988-1992	1.8	11.2	113.1	3.6	31.1	87.6	1.2	6.7	126.4	2.0	16.3	91.2
1993-1997	2.8	15.3	134.9	4.2	35.1	87.1	1.0	6.2	118.3	2.0	15.2	95.5
1998-2002	1.2	13.6	85.9	6.8	52.3	82.4	1.0	7.8	111.7	3.2	20.1	106.9
2003-2007	2.6	21.1	120.7	8.2	54.9	95.1	0.8	9.1	101.1	2.8	17.1	102.1
2008-2012	2.8	26.3	124.0	5.6	43.0	88.5	1.4	12.7	131.4	1.6	13.2	88.2
	25-34			65-74			25-34			65-74		
1983-1987	3.6	26.4	103.2	2.2	38.7	88.4	2.2	13.4	125.0	3.6	37.8	121.0
1988-1992	2.6	19.7	102.8	2.6	34.3	97.3	1.0	8.4	97.4	2.2	23.2	88.8
1993-1997	3.0	20.6	105.7	2.8	29.8	93.3	1.2	8.8	105.4	2.0	17.6	90.5
1998-2002	5.4	31.6	117.0	3.2	35.3	77.5	1.6	11.6	100.4	2.8	21.4	101.8
2003-2007	4.4	30.1	97.1	4.4	39.3	91.6	1.0	10.7	81.9	2.0	16.8	90.2
2008-2012	4.0	33.5	103.0	3.2	30.5	77.3	1.4	14.0	97.4	2.0	16.1	90.2
	35-44			>74 y/o			35-44			>74 y/o		
1983-1987	5.0	30.5	95.9	1.8	55.7	71.1	2.6	13.6	114.9	2.6	43.3	75.1
1988-1992	4.0	25.3	106.6	1.0	34.7	53.8	2.4	12.2	125.9	5.0	57.9	120.9
1993-1997	2.2	19.5	79.7	2.6	48.0	92.3	1.2	8.8	104.0	2.4	26.5	77.0
1998-2002	4.0	32.9	93.5	2.2	37.9	71.8	0.6	8.2	79.1	3.8	31.7	102.2
2003-2007	7.0	48.6	121.3	1.6	25.9	57.5	2.2	14.8	117.4	2.8	19.8	88.5
2008-2012	4.2	32.2	86.4	3.2	33.6	81.0	1.4	12.3	91.0	6.0	30.7	159.6

Secondary Medical Zone ID: 154

	Male						Female					
	Suicide			Suicide			Suicide			Suicide		
	Num per year	Rate /100,000	×100	Num per year	Rate /100,000	×100	Num per year	Rate /100,000	×100	Num per year	Rate /100,000	×100
	Total (>10 y/o)			45–54			Total (>10 y/o)			45–54		
1983–1987	23.2	30.9	93.3	4.4	41.9	82.8	16.0	16.3	109.6	3.8	23.8	138.2
1988–1992	22.0	25.5	105.7	4.0	33.4	97.3	13.0	12.5	98.7	1.4	13.3	91.3
1993–1997	22.0	25.3	99.7	7.0	45.8	125.2	14.6	12.3	118.7	2.8	16.2	125.5
1998–2002	33.6	36.4	103.3	8.6	61.7	110.3	14.8	12.8	106.1	1.8	14.5	96.4
2003–2007	37.4	39.3	112.6	6.8	56.1	100.3	11.6	11.7	93.4	1.8	14.3	104.7
2008–2012	38.2	39.2	119.8	6.2	52.2	107.4	11.0	12.3	92.1	1.8	15.6	104.4
	15–24			55–64			15–24			55–64		
1983–1987	1.0	11.8	84.7	4.2	40.1	94.4	0.8	7.2	105.1	2.2	18.8	95.1
1988–1992	1.6	11.4	115.5	5.6	43.8	123.2	0.2	4.1	77.2	2.2	17.7	98.9
1993–1997	0.6	9.1	80.8	3.4	34.0	84.4	1.0	6.5	125.4	1.8	15.4	96.6
1998–2002	1.6	16.0	101.6	7.2	61.7	97.1	0.8	7.5	106.1	2.6	19.7	104.7
2003–2007	2.2	20.6	118.0	10.0	70.8	122.6	0.4	7.5	83.6	1.2	13.5	80.8
2008–2012	2.4	24.7	116.6	7.6	53.6	110.3	0.6	9.0	92.9	2.0	14.9	99.9
	25–34			65–74			25–34			65–74		
1983–1987	3.8	29.9	117.1	1.2	28.1	64.2	1.6	12.3	114.5	2.2	27.4	87.6
1988–1992	2.0	18.5	97.0	2.4	32.2	91.3	0.8	8.3	95.7	3.6	32.4	124.1
1993–1997	3.0	23.1	118.0	1.6	22.0	68.9	2.0	12.0	143.9	2.4	20.2	103.8
1998–2002	4.0	29.8	110.4	4.4	43.6	95.9	0.4	8.7	74.8	4.0	27.8	132.5
2003–2007	3.4	29.2	94.3	5.6	49.8	116.1	1.0	11.5	88.2	1.6	16.1	86.6
2008–2012	4.4	36.8	113.0	4.4	39.9	100.9	1.0	13.0	91.0	1.4	15.1	84.9
	35–44			>74 y/o			35–44			>74 y/o		
1983–1987	5.2	36.3	114.1	3.4	83.3	106.3	1.8	12.6	106.3	3.6	59.4	103.0
1988–1992	3.4	24.8	104.7	2.8	60.0	93.0	1.2	9.5	98.5	3.6	46.5	97.1
1993–1997	3.2	25.6	104.3	3.0	52.3	100.5	1.0	8.5	100.4	3.4	35.3	102.5
1998–2002	4.0	35.0	99.3	3.8	53.2	101.0	1.4	11.3	109.4	3.8	32.1	103.3
2003–2007	5.6	45.4	113.4	3.8	43.3	96.2	1.0	11.3	89.4	4.6	30.0	133.8
2008–2012	8.4	51.8	139.2	4.4	42.1	101.4	1.4	12.9	95.5	2.8	17.8	92.5

Secondary Medical Zone ID: 155

	Male						Female					
	Suicide			Suicide			Suicide			Suicide		
	Num per year	Rate /100,000	×100	Num per year	Rate /100,000	×100	Num per year	Rate /100,000	×100	Num per year	Rate /100,000	×100
	Total (>10 y/o)			45–54			Total (>10 y/o)			45–54		
1983–1987	29.4	35.0	112.3	6.2	52.5	103.9	21.8	18.3	133.3	3.2	21.2	122.8
1988–1992	21.2	24.8	102.3	4.6	38.0	110.7	19.8	15.1	132.3	2.8	18.0	123.2
1993–1997	21.6	24.6	101.0	3.2	30.7	84.0	16.8	12.6	130.0	2.8	16.9	130.7
1998–2002	32.6	36.3	105.7	6.4	53.3	95.3	12.8	12.0	95.4	1.0	12.5	83.2
2003–2007	33.2	39.4	109.4	7.2	62.7	112.1	15.0	13.5	113.7	2.0	15.4	112.6
2008–2012	31.0	37.1	111.3	6.2	57.1	117.4	11.4	12.3	97.9	1.2	13.8	92.8
	15–24			55–64			15–24			55–64		
1983–1987	1.4	14.8	106.0	4.4	38.9	91.5	1.2	8.6	125.8	3.4	22.5	113.8
1988–1992	0.8	9.6	96.5	3.0	28.4	80.0	0.6	5.5	104.5	3.4	21.5	120.3
1993–1997	0.8	10.6	93.6	4.0	37.4	92.8	0.8	6.2	118.7	2.0	16.0	100.7
1998–2002	3.2	24.1	152.8	4.8	49.9	78.5	0.2	5.7	81.1	2.2	18.7	99.3
2003–2007	1.6	19.2	110.3	7.8	63.0	109.2	0.6	8.7	96.4	0.6	12.2	72.9
2008–2012	2.2	25.8	121.7	5.8	48.0	98.9	0.2	7.5	77.6	3.0	18.5	123.9
	25–34			65–74			25–34			65–74		
1983–1987	2.6	25.1	98.1	4.6	55.4	126.4	1.4	11.7	108.7	4.4	39.6	126.6
1988–1992	2.0	20.1	105.1	3.4	37.5	106.4	1.0	9.2	105.9	5.2	39.3	150.8
1993–1997	2.6	23.5	120.1	3.0	29.8	93.3	1.4	10.4	125.4	3.4	24.0	122.9
1998–2002	2.8	27.7	102.7	4.4	42.3	93.1	1.0	11.2	96.3	3.0	22.3	106.3
2003–2007	2.6	29.1	93.8	3.4	36.8	85.8	2.6	18.2	139.9	2.4	19.4	104.1
2008–2012	2.8	32.9	101.0	3.0	34.1	86.4	0.6	12.1	84.6	1.8	17.0	95.7
	35–44			>74 y/o			35–44			>74 y/o		
1983–1987	5.8	41.1	129.2	4.2	86.2	110.0	1.6	12.3	104.5	6.6	89.0	154.3
1988–1992	3.4	26.0	109.5	4.0	69.2	107.3	1.8	11.6	119.9	5.0	54.2	113.2
1993–1997	2.0	20.6	84.2	6.0	80.9	155.3	1.0	8.8	103.0	5.4	46.9	136.1
1998–2002	3.4	34.2	97.1	7.4	81.4	154.5	1.4	11.7	113.0	4.0	30.2	97.3
2003–2007	5.0	47.2	117.7	5.2	50.6	112.3	1.2	12.6	99.8	5.4	31.4	139.8
2008–2012	4.8	42.1	113.1	6.2	52.0	125.4	1.2	13.2	97.1	3.4	19.3	100.3

Secondary Medical Zone ID: 156

	Male						Female					
	Suicide			Suicide			Suicide			Suicide		
	Num per year	Rate /100,000	×100	Num per year	Rate /100,000	×100	Num per year	Rate /100,000	×100	Num per year	Rate /100,000	×100
	Total (>10 y/o)			45-54			Total (>10 y/o)			45-54		
1983-1987	5.4	31.4	90.7	1.2	44.5	88.1	4.0	15.2	105.2	1.4	22.1	128.2
1988-1992	3.4	23.1	83.8	0.6	30.7	89.5	3.8	13.1	108.7	0.8	16.4	111.9
1993-1997	5.8	26.3	111.6	1.0	36.7	100.4	1.8	10.3	86.3	0.6	14.1	109.4
1998-2002	6.4	35.9	98.3	1.2	52.7	94.1	2.2	12.1	90.5	0.0	13.4	89.1
2003-2007	5.2	34.8	90.8	1.4	57.6	103.1	2.0	12.4	93.4	0.4	14.3	104.3
2008-2012	5.8	34.7	104.1	1.2	51.1	105.1	3.2	13.4	108.9	0.4	15.7	105.4
	15-24			55-64			15-24			55-64		
1983-1987	0.6	16.4	117.7	0.8	36.9	86.9	0.4	8.0	117.0	0.8	20.6	104.5
1988-1992	0.2	9.9	100.3	1.8	42.8	120.4	0.2	5.7	108.6	0.8	19.1	106.9
1993-1997	0.2	11.3	99.9	1.6	44.7	111.1	0.0	4.9	93.1	0.2	14.1	88.4
1998-2002	0.4	16.7	106.0	0.8	53.7	84.5	0.0	6.6	93.8	0.8	20.3	107.6
2003-2007	0.2	17.3	99.1	1.0	52.2	90.5	0.4	10.8	120.4	0.2	15.7	93.7
2008-2012	0.2	21.0	99.0	1.4	49.9	102.6	0.2	10.4	107.2	0.6	15.9	106.9
	25-34			65-74			25-34			65-74		
1983-1987	1.0	28.7	112.3	0.4	38.0	86.7	0.6	12.9	119.5	0.4	26.7	85.4
1988-1992	0.2	17.1	89.5	0.0	25.3	71.8	0.2	8.9	102.1	0.6	24.5	94.1
1993-1997	0.8	22.9	117.3	0.2	24.5	76.7	0.0	7.6	91.8	0.4	17.5	89.9
1998-2002	0.6	27.7	102.5	1.2	45.7	100.6	0.0	10.7	92.5	0.8	22.2	105.9
2003-2007	0.2	28.4	91.5	0.8	40.1	93.5	0.0	12.0	92.6	0.2	16.3	87.7
2008-2012	0.6	34.0	104.3	1.2	43.0	108.9	0.2	14.5	101.3	0.8	19.7	110.5
	35-44			>74 y/o			35-44			>74 y/o		
1983-1987	1.0	32.5	102.0	0.4	66.9	85.4	0.4	12.2	103.3	0.0	29.0	50.3
1988-1992	0.4	21.5	90.5	0.2	48.7	75.4	0.4	10.3	106.9	0.8	43.9	91.7
1993-1997	1.0	28.9	117.8	0.8	54.7	105.0	0.4	9.6	113.4	0.2	21.0	60.9
1998-2002	1.6	45.2	128.4	0.6	46.9	89.0	0.2	10.4	100.6	0.4	21.9	70.5
2003-2007	0.2	32.3	80.6	1.4	53.1	118.0	0.0	11.6	91.8	0.8	22.1	98.5
2008-2012	0.4	35.6	95.8	0.8	39.2	94.5	0.2	13.6	100.1	0.8	19.4	101.1

Secondary Medical Zone ID: 157

	Male						Female					
	Suicide			Suicide			Suicide			Suicide		
	Num per year	Rate /100,000	×100	Num per year	Rate /100,000	×100	Num per year	Rate /100,000	×100	Num per year	Rate /100,000	×100
	Total (>10 y/o)			45-54			Total (>10 y/o)			45-54		
1983-1987	50.4	31.3	94.8	9.8	41.0	81.2	29.8	15.2	99.9	5.0	18.5	107.2
1988-1992	42.6	24.1	96.7	9.4	34.7	100.9	28.0	12.9	100.3	3.0	12.5	85.6
1993-1997	43.8	23.2	91.4	12.6	40.3	110.2	24.0	10.7	96.3	4.6	14.4	111.3
1998-2002	68.4	34.2	94.9	15.4	52.4	93.6	27.6	11.7	93.4	5.2	16.6	110.3
2003-2007	68.0	34.0	92.5	12.8	49.4	88.3	25.8	11.9	91.5	2.0	10.2	74.2
2008-2012	73.2	36.8	105.3	12.6	49.2	101.3	29.2	13.2	101.6	3.2	13.7	91.6
	15-24			55-64			15-24			55-64		
1983-1987	3.6	14.4	103.4	9.0	41.6	97.9	2.8	9.4	137.7	5.2	20.4	103.2
1988-1992	2.2	8.7	87.5	8.8	36.3	102.2	2.0	6.6	124.5	4.6	17.5	98.2
1993-1997	4.2	13.4	118.4	8.6	36.2	89.9	2.0	6.6	125.7	5.2	18.5	116.7
1998-2002	5.6	20.0	126.8	13.0	51.9	81.7	1.6	7.0	99.6	4.0	16.2	86.2
2003-2007	3.6	16.9	96.8	19.2	64.6	111.9	2.0	9.6	106.9	5.0	16.7	99.7
2008-2012	6.0	26.5	124.8	14.0	48.6	100.0	2.4	11.6	119.4	6.8	19.5	130.9
	25-34			65-74			25-34			65-74		
1983-1987	7.0	27.3	106.8	6.0	45.3	103.4	2.0	9.0	84.0	4.4	26.4	84.3
1988-1992	4.6	18.5	96.7	6.4	39.7	112.8	2.2	8.9	102.5	7.2	33.1	126.9
1993-1997	4.4	16.7	85.4	4.6	25.6	80.3	1.2	6.2	74.6	3.4	15.5	79.4
1998-2002	7.2	24.1	89.1	12.0	53.2	116.9	1.6	8.0	69.4	5.4	21.3	101.4
2003-2007	8.2	27.9	90.1	8.4	38.8	90.5	3.4	12.5	95.9	3.8	16.1	86.7
2008-2012	10.4	38.2	117.4	8.4	36.3	91.8	4.0	15.8	110.1	3.4	14.7	82.8
	35-44			>74 y/o			35-44			>74 y/o		
1983-1987	9.4	30.8	96.9	5.6	74.3	94.9	2.8	10.2	86.3	7.6	63.6	110.2
1988-1992	7.8	25.6	108.0	3.4	41.4	64.2	3.6	11.1	115.4	5.4	36.4	76.2
1993-1997	4.2	17.5	71.3	5.0	46.4	89.1	2.4	8.9	104.6	5.0	27.3	79.2
1998-2002	8.4	32.3	91.7	6.4	47.6	90.3	2.0	8.9	85.6	7.6	31.9	102.7
2003-2007	9.4	33.9	84.7	6.4	38.0	84.3	3.6	13.0	103.1	6.0	20.8	92.8
2008-2012	14.2	42.8	115.1	7.4	36.2	87.2	3.4	12.3	90.5	6.0	18.1	93.9

Secondary Medical Zone ID: 158

	Male						Female					
	Suicide			Suicide			Suicide			Suicide		
	Num per year	Rate /100,000	×100	Num per year	Rate /100,000	×100	Num per year	Rate /100,000	×100	Num per year	Rate /100,000	×100
	Total (>10 y/o)			45–54			Total (>10 y/o)			45–54		
1983–1987	11.8	35.1	117.9	1.6	43.4	85.8	7.8	17.3	125.4	0.2	14.1	81.6
1988–1992	9.2	25.9	115.0	1.2	31.3	91.2	5.6	13.1	108.3	0.4	13.5	92.7
1993–1997	11.8	28.1	130.5	2.4	40.3	110.1	5.8	11.7	118.7	1.0	14.4	111.9
1998–2002	12.6	36.1	104.3	3.8	64.1	114.5	2.8	11.3	78.4	0.4	13.7	90.9
2003–2007	13.8	39.2	113.1	3.2	65.0	116.2	6.2	13.2	114.5	1.0	15.6	113.9
2008–2012	11.0	35.0	103.1	1.8	48.2	99.2	3.6	12.2	93.5	0.0	12.2	81.5
	15–24			55–64			15–24			55–64		
1983–1987	1.0	17.5	125.2	2.4	48.2	113.4	0.4	7.4	108.7	1.6	23.7	120.1
1988–1992	1.0	12.4	125.7	1.0	30.0	84.4	0.4	6.1	115.4	1.2	19.6	109.7
1993–1997	1.2	14.6	129.0	3.2	52.7	131.0	0.4	6.1	116.2	0.4	13.9	87.6
1998–2002	0.6	16.3	103.1	2.4	59.3	93.3	0.2	7.0	99.1	0.4	16.7	88.5
2003–2007	0.2	15.9	91.2	2.6	55.1	95.4	0.2	8.8	98.0	1.4	19.0	113.5
2008–2012	0.4	20.5	96.6	1.4	41.3	85.1	0.4	10.9	112.1	0.4	13.6	91.2
	25–34			65–74			25–34			65–74		
1983–1987	1.6	30.3	118.7	2.4	64.1	146.4	0.4	10.7	99.0	1.0	30.9	98.8
1988–1992	1.2	23.4	122.3	2.2	49.6	140.6	0.6	10.0	115.3	1.0	25.9	99.2
1993–1997	0.8	19.9	101.9	1.0	30.0	94.0	0.2	7.9	94.7	1.8	27.5	140.9
1998–2002	0.6	23.8	88.1	2.0	47.2	103.8	0.0	9.7	83.6	0.4	16.8	80.0
2003–2007	1.2	31.1	100.3	2.6	52.2	121.6	0.2	11.7	90.2	0.6	17.2	92.2
2008–2012	1.4	35.3	108.4	1.4	38.1	96.5	0.0	12.3	86.2	0.6	17.0	95.3
	35–44			>74 y/o			35–44			>74 y/o		
1983–1987	1.4	29.8	93.8	1.4	87.4	111.6	1.0	13.5	114.6	3.2	108.9	188.7
1988–1992	1.2	23.9	100.9	1.4	72.1	111.8	0.6	10.2	105.3	1.4	49.0	102.4
1993–1997	1.4	27.5	112.3	1.8	69.6	133.7	0.6	9.7	113.6	1.4	37.9	110.0
1998–2002	1.0	31.4	89.2	2.2	67.6	128.3	0.0	8.4	81.6	1.4	31.5	101.6
2003–2007	2.8	53.3	133.0	1.2	41.2	91.5	0.4	12.3	97.1	2.4	34.8	155.0
2008–2012	2.4	42.9	115.4	2.2	48.7	117.4	0.2	12.2	90.3	2.0	25.3	131.8

Secondary Medical Zone ID: 159

	Male						Female					
	Suicide			Suicide			Suicide			Suicide		
	Num per year	Rate /100,000	×100	Num per year	Rate /100,000	×100	Num per year	Rate /100,000	×100	Num per year	Rate /100,000	×100
	Total (>10 y/o)			45-54			Total (>10 y/o)			45-54		
1983-1987	61.4	28.6	85.4	12.0	36.6	72.3	38.8	14.7	95.9	5.4	15.4	89.6
1988-1992	58.2	24.0	96.9	12.2	32.6	95.0	39.4	12.8	102.8	4.4	12.5	85.8
1993-1997	58.6	22.9	89.7	13.8	33.1	90.4	35.6	11.1	103.2	6.0	13.9	107.6
1998-2002	88.0	32.7	91.0	17.4	43.9	78.4	46.0	13.7	111.6	5.2	13.4	89.2
2003-2007	92.4	36.2	94.7	22.2	60.7	108.5	40.0	12.4	102.8	4.8	13.3	97.4
2008-2012	84.0	33.1	94.2	15.6	46.9	96.6	37.4	12.9	99.0	6.2	17.2	115.2
	15-24			55-64			15-24			55-64		
1983-1987	3.6	11.9	85.2	9.4	33.0	77.7	2.4	6.9	101.7	5.8	17.5	88.8
1988-1992	5.2	12.9	130.0	10.6	32.2	90.6	1.8	5.0	95.2	5.8	16.4	91.9
1993-1997	3.2	9.8	86.4	9.4	29.8	73.9	2.0	5.4	104.2	5.6	15.5	97.6
1998-2002	6.8	20.0	126.7	22.2	61.7	97.1	2.8	8.3	117.5	6.2	17.2	91.3
2003-2007	7.2	23.8	136.7	20.0	50.7	87.9	3.2	11.1	123.9	6.2	15.6	93.2
2008-2012	5.6	23.2	109.3	20.6	51.2	105.4	1.6	8.1	83.1	4.8	12.8	86.0
	25-34			65-74			25-34			65-74		
1983-1987	7.8	23.5	92.1	7.6	42.8	97.7	3.8	10.7	99.8	6.6	28.2	90.1
1988-1992	5.4	17.0	89.2	7.6	35.4	100.6	3.8	10.3	119.1	8.6	29.9	114.7
1993-1997	6.8	19.4	99.3	6.8	26.6	83.3	3.0	8.4	101.4	6.2	19.2	98.5
1998-2002	8.0	22.3	82.5	13.2	44.6	98.0	6.2	14.8	127.8	9.6	26.3	125.3
2003-2007	11.0	30.4	98.0	8.6	30.6	71.4	3.6	11.0	84.9	9.0	24.1	129.5
2008-2012	9.4	31.9	98.1	10.2	33.2	83.9	3.6	13.1	91.2	6.2	17.5	98.2
	35-44			>74 y/o			35-44			>74 y/o		
1983-1987	11.0	26.6	83.5	9.4	93.2	118.9	3.0	8.4	71.5	11.6	75.2	130.3
1988-1992	9.2	22.3	94.0	8.0	63.7	98.7	3.6	9.0	93.1	11.2	54.1	113.1
1993-1997	9.2	24.5	99.7	8.6	56.9	109.2	4.6	11.2	131.3	8.2	32.2	93.6
1998-2002	9.8	29.0	82.3	10.4	55.0	104.3	3.4	9.9	96.0	12.2	37.6	121.0
2003-2007	14.2	39.4	98.4	9.2	38.9	86.4	2.8	9.5	75.1	10.4	25.9	115.3
2008-2012	12.2	32.8	88.2	10.4	36.8	88.6	6.0	15.1	111.2	9.0	19.6	102.0

Secondary Medical Zone ID: 160

	Male						Female					
	Suicide			Suicide			Suicide			Suicide		
	Num per year	Rate /100,000	×100	Num per year	Rate /100,000	×100	Num per year	Rate /100,000	×100	Num per year	Rate /100,000	×100
	Total (>10 y/o)			45–54			Total (>10 y/o)			45–54		
1983–1987	16.6	34.2	108.3	3.8	53.1	105.1	12.4	17.8	127.5	3.0	25.0	145.2
1988–1992	13.8	26.1	112.4	2.4	35.5	103.3	12.8	15.1	141.0	1.4	16.1	110.4
1993–1997	14.8	27.0	114.5	2.2	33.6	91.9	9.4	11.5	123.7	0.8	12.5	96.8
1998–2002	19.8	36.9	109.1	3.6	50.9	91.0	12.0	14.0	132.4	1.0	14.7	98.0
2003–2007	20.8	39.0	116.3	5.4	70.8	126.5	7.6	12.3	104.4	0.4	11.7	85.6
2008–2012	20.2	37.6	120.5	3.0	49.6	102.1	6.0	12.3	95.8	0.6	13.7	92.0
	15–24			55–64			15–24			55–64		
1983–1987	1.4	17.5	125.3	2.0	35.2	82.8	0.2	6.1	89.5	1.2	17.7	89.9
1988–1992	0.4	9.2	93.2	3.2	40.4	113.8	0.0	4.1	78.6	1.8	19.6	109.9
1993–1997	0.6	11.1	98.5	3.4	45.7	113.5	0.6	6.3	121.1	1.4	17.1	107.6
1998–2002	0.8	15.9	100.3	4.0	63.8	100.5	0.6	8.0	113.4	1.6	20.2	107.5
2003–2007	0.2	14.6	83.8	5.0	65.1	112.8	0.2	8.1	89.7	1.0	16.1	96.4
2008–2012	1.4	25.1	118.1	3.8	49.0	100.9	0.4	10.0	102.9	0.8	14.0	93.6
	25–34			65–74			25–34			65–74		
1983–1987	1.6	24.7	96.8	2.6	51.8	118.4	1.6	15.0	138.9	1.4	27.8	89.0
1988–1992	1.4	21.0	110.0	1.2	29.6	84.1	1.0	10.7	123.7	2.2	30.2	115.9
1993–1997	2.6	28.8	147.3	1.8	31.0	97.2	0.2	7.3	87.3	1.0	16.5	84.6
1998–2002	1.4	26.3	97.5	4.0	54.5	119.8	1.4	14.6	125.6	1.6	21.2	101.0
2003–2007	1.4	29.4	94.9	2.8	45.4	105.9	1.0	14.5	111.4	0.8	16.0	85.9
2008–2012	1.4	32.2	98.8	2.8	44.1	111.7	0.2	12.4	86.4	0.8	16.3	91.9
	35–44			>74 y/o			35–44			>74 y/o		
1983–1987	3.0	36.0	113.0	2.2	88.8	113.4	1.2	13.2	112.2	3.8	88.0	152.6
1988–1992	2.6	28.4	119.6	2.6	80.3	124.5	0.4	8.7	89.6	6.0	102.3	213.8
1993–1997	2.0	26.6	108.6	2.0	56.4	108.4	0.4	7.8	92.2	5.0	70.3	204.4
1998–2002	2.8	40.3	114.4	3.2	66.5	126.1	0.2	8.5	82.3	5.6	63.4	204.1
2003–2007	2.4	41.4	103.3	3.6	59.1	131.2	0.4	11.4	90.2	3.8	37.0	165.0
2008–2012	4.0	48.5	130.3	3.8	53.7	129.4	0.4	12.3	91.0	2.8	24.9	129.3

Secondary Medical Zone ID: 161

	Male						Female					
	Suicide			Suicide			Suicide			Suicide		
	Num per year	Rate /100,000	×100	Num per year	Rate /100,000	×100	Num per year	Rate /100,000	×100	Num per year	Rate /100,000	×100
	Total (>10 y/o)			45–54			Total (>10 y/o)			45–54		
1983–1987	82.4	28.6	85.2	20.4	41.1	81.3	48.4	14.8	96.4	8.2	16.1	93.5
1988–1992	66.2	20.8	83.0	14.4	26.6	77.3	44.6	11.9	93.8	6.6	12.3	83.9
1993–1997	75.2	21.7	85.7	17.4	29.4	80.4	44.8	10.8	101.2	6.0	10.3	79.5
1998–2002	122.8	33.9	93.1	28.8	50.7	90.6	58.2	13.1	107.5	7.6	13.2	87.5
2003–2007	129.2	34.7	96.2	24.6	51.1	91.3	55.6	12.6	104.5	6.8	13.4	97.5
2008–2012	117.0	32.5	93.3	20.0	43.8	90.1	46.8	11.4	89.5	4.4	10.6	71.0
	15–24			55–64			15–24			55–64		
1983–1987	9.0	16.5	118.3	11.0	33.7	79.2	3.0	5.5	81.2	8.4	21.4	108.2
1988–1992	5.0	8.8	88.5	16.8	38.6	108.8	1.6	3.2	60.3	10.6	22.3	124.7
1993–1997	5.8	10.4	91.9	17.8	37.6	93.3	3.4	5.4	104.3	8.8	17.3	108.6
1998–2002	9.8	19.1	121.0	32.2	61.4	96.6	3.8	7.4	104.8	10.0	18.2	96.9
2003–2007	7.4	17.5	100.4	34.6	59.8	103.6	2.8	7.2	79.5	13.6	20.9	125.0
2008–2012	5.8	16.6	78.0	24.8	45.5	93.7	3.2	8.6	88.7	8.6	14.6	97.7
	25–34			65–74			25–34			65–74		
1983–1987	10.4	21.9	85.5	6.2	34.2	78.0	6.2	11.5	106.8	7.6	30.3	96.9
1988–1992	5.2	11.9	62.5	7.4	33.1	94.0	2.4	5.7	66.2	8.0	26.6	101.9
1993–1997	8.6	16.9	86.7	9.8	33.2	104.0	4.0	7.6	91.3	7.0	19.6	100.7
1998–2002	16.2	28.3	104.6	14.0	39.6	87.2	7.0	11.9	102.5	10.2	24.1	114.5
2003–2007	14.6	27.6	88.8	17.8	43.0	100.2	7.2	13.1	100.5	9.4	19.9	106.8
2008–2012	17.0	36.2	111.2	16.6	36.3	91.9	5.0	11.9	83.2	7.8	15.7	88.2
	35–44			>74 y/o			35–44			>74 y/o		
1983–1987	16.0	25.7	80.7	9.2	86.6	110.6	6.6	10.4	88.1	8.4	58.3	101.0
1988–1992	9.2	16.5	69.5	8.2	62.3	96.5	5.6	9.2	95.2	9.6	47.6	99.5
1993–1997	8.6	18.1	73.8	7.0	46.4	89.1	4.2	8.1	95.6	11.4	43.6	126.7
1998–2002	15.4	32.5	92.3	6.0	34.9	66.2	6.0	11.7	112.9	13.6	41.1	132.4
2003–2007	17.6	34.5	86.1	12.4	48.9	108.5	5.0	10.3	81.4	10.6	25.6	113.9
2008–2012	20.4	35.6	95.6	12.0	38.1	91.8	7.0	12.4	91.5	10.8	21.3	110.9

Secondary Medical Zone ID: 162

	Male						Female					
	Suicide			Suicide			Suicide			Suicide		
	Num per year	Rate /100,000	×100	Num per year	Rate /100,000	×100	Num per year	Rate /100,000	×100	Num per year	Rate /100,000	×100
	Total (>10 y/o)			45-54			Total (>10 y/o)			45-54		
1983-1987	42.8	29.7	87.1	9.4	40.5	80.1	28.6	16.1	106.7	5.8	21.2	122.9
1988-1992	27.8	19.1	71.0	3.8	18.5	53.8	23.6	12.2	96.0	3.6	14.1	96.1
1993-1997	33.6	21.0	78.0	8.6	30.7	83.8	19.4	9.7	88.6	2.0	9.2	71.1
1998-2002	50.0	29.5	77.6	12.6	45.1	80.6	20.6	10.3	80.8	2.8	11.7	77.5
2003-2007	55.2	31.5	83.8	10.6	44.1	78.8	26.8	12.7	103.3	3.2	12.9	94.0
2008-2012	48.0	30.1	80.4	9.0	42.4	87.3	22.2	11.7	90.4	2.8	13.1	88.0
	15-24			55-64			15-24			55-64		
1983-1987	4.4	16.5	118.4	6.4	35.6	83.9	1.2	5.2	76.8	3.2	16.8	85.1
1988-1992	1.4	7.0	71.1	7.2	32.8	92.3	1.6	5.2	99.3	5.0	20.2	113.0
1993-1997	4.0	13.0	114.8	6.0	29.1	72.3	2.0	6.2	118.6	2.8	13.0	81.8
1998-2002	3.6	15.2	96.2	9.8	43.4	68.3	0.6	4.6	65.8	5.2	19.7	104.4
2003-2007	3.6	17.3	99.3	14.2	51.1	88.6	2.0	9.4	104.8	5.6	18.4	109.6
2008-2012	3.8	20.7	97.8	10.0	38.3	78.8	1.4	8.6	88.6	3.6	13.4	89.6
	25-34			65-74			25-34			65-74		
1983-1987	4.8	21.2	82.8	5.6	48.6	111.1	2.8	10.9	101.2	4.6	31.1	99.7
1988-1992	4.0	18.0	94.1	3.6	30.4	86.2	0.6	5.2	59.8	4.8	28.0	107.3
1993-1997	3.4	16.0	81.8	3.6	24.3	76.2	1.2	6.5	77.6	2.6	15.0	76.8
1998-2002	4.6	20.7	76.7	7.6	40.9	89.9	1.2	7.7	66.6	3.6	18.0	85.5
2003-2007	8.2	31.9	102.9	5.4	30.2	70.4	3.2	13.1	100.8	4.6	19.7	105.8
2008-2012	6.8	31.7	97.3	6.0	30.4	77.1	2.2	12.4	86.6	4.4	18.1	101.9
	35-44			>74 y/o			35-44			>74 y/o		
1983-1987	7.2	25.4	79.9	5.0	79.5	101.5	3.0	10.7	90.7	7.8	82.8	143.5
1988-1992	3.4	14.2	60.0	4.4	58.8	91.1	2.0	7.9	82.3	6.0	49.6	103.6
1993-1997	4.6	19.5	79.5	3.2	39.7	76.3	2.4	8.9	104.2	6.4	42.0	122.1
1998-2002	6.2	28.6	81.2	5.6	52.5	99.6	1.0	6.7	64.7	6.0	32.4	104.5
2003-2007	6.8	30.9	77.1	6.2	44.4	98.6	2.6	11.6	92.1	5.6	24.4	108.9
2008-2012	7.0	30.2	81.2	5.2	33.0	79.6	2.2	10.9	80.7	5.6	20.7	107.6

Secondary Medical Zone ID: 163

	Male						Female					
	Suicide			Suicide			Suicide			Suicide		
	Num per year	Rate /100,000	×100	Num per year	Rate /100,000	×100	Num per year	Rate /100,000	×100	Num per year	Rate /100,000	×100
	Total (>10 y/o)			45–54			Total (>10 y/o)			45–54		
1983–1987	46.2	31.9	96.9	12.4	54.5	107.8	29.6	16.8	114.2	5.0	20.1	116.6
1988–1992	39.0	24.4	98.3	8.2	33.6	97.9	29.8	14.3	121.0	3.2	13.9	95.1
1993–1997	36.6	22.0	85.4	7.4	28.2	77.0	27.8	12.0	123.1	2.8	11.3	87.1
1998–2002	58.2	33.1	89.9	13.2	46.6	83.4	24.6	11.7	94.3	2.8	11.8	78.3
2003–2007	61.6	34.1	93.4	10.0	41.7	74.5	28.2	12.7	108.8	4.6	16.0	116.7
2008–2012	59.6	33.7	97.9	10.6	47.9	98.6	24.8	11.9	99.1	1.6	10.1	67.9
	15–24			55–64			15–24			55–64		
1983–1987	3.0	14.0	100.2	6.6	35.8	84.4	0.6	4.8	70.1	3.8	18.4	93.3
1988–1992	3.2	11.6	117.0	6.8	31.9	89.9	0.8	4.2	79.9	3.4	16.0	89.4
1993–1997	2.6	10.6	94.0	8.2	37.7	93.5	1.0	4.6	87.4	4.4	17.9	112.4
1998–2002	4.2	17.0	107.4	14.4	60.7	95.6	2.2	8.4	119.2	4.2	17.9	95.3
2003–2007	3.2	16.2	92.8	16.8	59.5	103.1	0.8	6.2	68.6	4.4	16.3	97.1
2008–2012	4.6	23.7	111.9	13.2	46.1	95.0	2.0	10.9	112.1	5.0	16.2	108.3
	25–34			65–74			25–34			65–74		
1983–1987	4.2	21.0	82.3	4.4	40.4	92.2	3.0	12.1	112.8	6.0	38.6	123.6
1988–1992	4.8	22.2	116.1	4.6	34.1	96.8	2.2	9.5	109.4	7.2	38.0	145.8
1993–1997	2.4	14.0	71.9	4.6	27.6	86.5	1.8	8.3	99.7	4.8	22.5	115.5
1998–2002	4.0	20.4	75.5	7.8	41.0	90.2	2.6	11.5	98.9	4.0	19.0	90.4
2003–2007	6.6	28.7	92.6	8.0	40.6	94.6	3.8	15.3	117.3	4.2	18.8	100.8
2008–2012	6.6	32.1	98.5	6.8	33.8	85.6	1.8	11.6	81.1	3.6	16.6	93.4
	35–44			>74 y/o			35–44			>74 y/o		
1983–1987	8.8	31.3	98.3	6.8	91.2	116.4	2.2	9.7	81.8	9.0	89.8	155.7
1988–1992	5.6	20.3	85.4	5.6	66.3	102.8	2.2	8.6	88.7	10.6	80.6	168.5
1993–1997	5.4	21.8	89.1	6.0	59.3	113.9	2.2	8.5	99.4	10.8	65.7	190.8
1998–2002	8.0	35.1	99.7	6.6	53.7	101.8	0.8	6.3	60.5	8.0	39.3	126.5
2003–2007	9.4	41.5	103.7	7.6	47.2	104.9	1.8	10.0	79.3	8.4	32.6	145.4
2008–2012	8.2	34.9	93.9	9.4	48.4	116.6	2.0	10.9	80.5	8.8	28.5	148.1

Secondary Medical Zone ID: 164

	Male						Female					
	Suicide			Suicide			Suicide			Suicide		
	Num per year	Rate /100,000	×100	Num per year	Rate /100,000	×100	Num per year	Rate /100,000	×100	Num per year	Rate /100,000	×100
	Total (>10 y/o)			45–54			Total (>10 y/o)			45–54		
1983–1987	47.8	33.7	103.9	10.2	44.5	87.9	38.4	20.3	146.4	5.2	19.9	115.7
1988–1992	42.8	26.5	112.6	9.8	39.1	113.7	32.0	14.8	131.4	3.2	13.6	93.2
1993–1997	47.2	27.3	113.2	10.6	38.5	105.3	31.2	13.2	139.1	4.4	14.8	114.9
1998–2002	69.4	38.8	112.2	17.8	64.1	114.5	32.6	13.8	122.9	4.6	16.2	107.8
2003–2007	63.0	36.3	102.4	9.8	44.2	79.1	28.2	12.9	112.4	2.4	11.7	85.0
2008–2012	49.8	31.5	90.4	8.2	41.6	85.6	27.0	12.6	110.1	3.0	14.3	95.8
	15–24			55–64			15–24			55–64		
1983–1987	3.0	14.2	101.8	8.4	43.9	103.3	1.6	7.0	103.0	6.2	25.8	130.8
1988–1992	2.4	10.2	102.6	9.6	41.8	117.9	0.2	2.9	54.8	6.2	23.3	130.6
1993–1997	2.8	11.7	103.1	10.2	43.8	108.9	1.0	4.7	89.2	5.2	19.7	124.0
1998–2002	3.0	15.0	94.7	17.6	72.8	114.5	1.8	7.8	111.3	4.6	18.6	99.0
2003–2007	3.4	18.0	103.1	18.2	68.2	118.1	1.0	7.1	78.4	4.6	17.0	101.4
2008–2012	3.2	20.6	97.1	12.2	47.3	97.4	1.6	9.9	101.6	3.2	13.3	89.1
	25–34			65–74			25–34			65–74		
1983–1987	6.8	30.7	120.0	5.4	49.1	112.1	3.2	13.1	121.7	7.0	44.9	143.7
1988–1992	4.8	22.7	118.6	5.0	38.0	107.9	0.6	5.6	64.2	5.0	29.0	111.3
1993–1997	3.2	16.7	85.5	6.6	38.0	119.2	1.8	8.3	100.2	6.4	28.6	147.0
1998–2002	6.4	28.3	104.8	8.2	43.5	95.6	2.4	11.2	96.3	5.4	23.4	111.5
2003–2007	5.6	27.8	89.5	9.4	45.9	107.0	3.4	14.8	114.1	5.4	22.0	118.1
2008–2012	5.2	30.2	92.7	5.6	30.5	77.1	2.4	14.1	98.5	5.6	21.5	120.8
	35–44			>74 y/o			35–44			>74 y/o		
1983–1987	6.2	25.1	79.0	7.4	115.6	147.6	4.0	13.6	115.4	11.2	117.7	204.0
1988–1992	4.2	17.8	74.9	6.8	87.8	136.0	2.4	9.3	96.6	14.4	112.7	235.6
1993–1997	8.6	33.8	137.9	5.2	59.0	113.2	2.0	8.4	99.0	10.4	66.2	192.4
1998–2002	8.8	39.2	111.4	7.2	63.3	120.1	1.6	8.5	82.7	11.8	58.5	188.3
2003–2007	9.2	42.0	104.9	7.2	49.3	109.4	3.0	13.3	104.9	8.2	32.7	145.8
2008–2012	6.4	31.7	85.2	9.0	49.8	120.0	1.2	9.4	69.4	10.0	32.3	167.9

Secondary Medical Zone ID: 165

	Male						Female					
	Suicide			Suicide			Suicide			Suicide		
	Num per year	Rate /100,000	×100	Num per year	Rate /100,000	×100	Num per year	Rate /100,000	×100	Num per year	Rate /100,000	×100
	Total (>10 y/o)			45–54			Total (>10 y/o)			45–54		
1983–1987	36.8	41.2	144.3	8.4	62.5	123.6	19.8	18.4	133.3	2.2	17.0	98.7
1988–1992	32.6	32.4	157.8	6.4	46.8	136.4	20.4	15.9	146.7	2.0	15.0	102.5
1993–1997	27.4	28.4	129.0	5.4	40.2	109.9	17.2	12.8	141.1	3.0	17.0	131.3
1998–2002	31.0	35.9	104.4	7.2	57.5	102.7	20.0	14.9	139.9	2.6	17.4	115.5
2003–2007	37.2	41.0	125.8	6.8	62.7	112.0	16.0	13.8	123.8	2.8	18.4	134.5
2008–2012	42.0	41.7	150.8	7.0	63.4	130.4	12.8	13.1	108.7	1.4	14.9	99.8
	15–24			55–64			15–24			55–64		
1983–1987	3.0	22.6	162.0	7.4	60.5	142.3	1.4	9.4	137.3	4.0	26.8	135.9
1988–1992	0.6	8.7	88.0	6.4	47.5	133.8	0.0	3.7	69.3	3.2	21.4	119.8
1993–1997	2.0	14.4	127.2	6.4	49.0	121.8	0.6	5.6	108.0	3.0	19.7	124.1
1998–2002	1.8	17.9	113.3	9.2	75.3	118.5	0.8	7.9	112.5	4.6	26.4	140.0
2003–2007	1.6	19.1	109.3	8.8	68.5	118.6	0.6	9.1	100.7	3.0	19.3	115.5
2008–2012	1.6	23.3	109.8	8.6	62.0	127.7	0.6	10.0	103.2	1.8	14.8	99.3
	25–34			65–74			25–34			65–74		
1983–1987	3.0	28.1	110.1	5.0	66.0	150.8	1.8	13.5	125.1	3.0	34.5	110.3
1988–1992	2.0	20.6	107.8	6.4	66.5	188.9	1.0	9.3	107.0	4.4	38.8	148.8
1993–1997	1.6	18.5	94.5	4.4	42.7	133.9	0.4	7.0	83.9	3.4	26.4	135.5
1998–2002	1.8	22.9	84.9	5.2	48.5	106.6	0.6	9.8	84.7	4.0	28.2	134.3
2003–2007	3.2	32.2	103.8	7.0	58.7	136.9	1.2	13.1	100.4	5.2	31.2	167.5
2008–2012	3.2	36.1	110.8	8.0	62.4	158.0	0.6	12.5	87.2	3.0	21.1	118.8
	35–44			>74 y/o			35–44			>74 y/o		
1983–1987	5.4	38.3	120.2	4.6	108.6	138.6	2.0	13.1	111.0	5.2	85.2	147.7
1988–1992	4.0	29.0	122.2	6.8	123.0	190.6	2.6	13.7	142.0	7.2	88.6	185.2
1993–1997	2.4	23.6	96.1	5.0	83.6	160.5	0.8	8.1	95.8	6.0	61.4	178.4
1998–2002	1.6	23.9	67.9	4.2	63.0	119.5	1.2	11.1	106.9	6.2	51.2	165.0
2003–2007	4.4	43.8	109.3	5.4	60.7	134.9	0.2	9.0	71.5	3.0	22.7	101.0
2008–2012	4.0	38.9	104.5	9.6	80.3	193.6	1.2	13.3	97.8	4.2	24.9	129.5

Secondary Medical Zone ID: 166

	Male						Female					
	Suicide			Suicide			Suicide			Suicide		
	Num per year	Rate /100,000	×100	Num per year	Rate /100,000	×100	Num per year	Rate /100,000	×100	Num per year	Rate /100,000	×100
	Total (>10 y/o)			45-54			Total (>10 y/o)			45-54		
1983-1987	15.2	35.1	112.7	2.8	45.9	90.8	6.2	14.4	87.5	0.6	14.5	84.4
1988-1992	12.4	26.3	113.2	3.2	41.2	119.9	4.6	11.3	77.5	0.6	13.0	89.3
1993-1997	11.2	25.7	101.7	1.8	31.6	86.4	6.2	11.1	101.2	1.6	15.4	119.1
1998-2002	17.0	37.5	107.6	4.0	58.2	104.0	7.0	12.7	101.5	1.2	15.8	104.8
2003-2007	19.2	40.3	124.5	4.2	69.9	125.0	8.8	14.0	122.9	1.4	16.6	121.2
2008-2012	19.0	38.2	132.0	3.4	59.9	123.3	7.8	13.8	117.0	0.8	15.9	106.6
	15-24			55-64			15-24			55-64		
1983-1987	0.8	15.6	111.8	1.2	32.0	75.3	0.4	7.2	106.1	0.2	13.5	68.3
1988-1992	1.0	12.2	123.4	1.6	29.9	84.2	0.0	4.4	83.0	0.8	15.7	87.8
1993-1997	0.4	11.2	98.8	1.8	34.4	85.5	0.0	4.4	84.6	0.8	14.4	90.5
1998-2002	0.4	15.5	97.9	3.2	55.8	87.9	0.2	7.0	99.1	0.8	16.6	88.4
2003-2007	0.2	16.2	93.0	4.8	63.8	110.4	0.2	8.9	98.9	1.4	17.4	103.7
2008-2012	0.2	19.6	92.6	5.6	63.3	130.2	0.2	9.7	100.5	2.0	18.4	123.2
	25-34			65-74			25-34			65-74		
1983-1987	2.2	32.6	127.5	1.8	47.6	108.8	1.0	13.0	120.5	1.2	28.1	90.0
1988-1992	0.8	19.0	99.2	2.2	43.4	123.2	0.2	7.9	91.1	0.8	20.0	76.5
1993-1997	1.6	24.8	127.2	1.8	33.6	105.4	0.8	10.2	122.8	1.4	20.8	106.5
1998-2002	2.2	33.7	124.7	3.0	48.7	107.1	0.4	11.3	97.2	1.8	23.6	112.4
2003-2007	2.0	37.7	121.5	3.4	50.8	118.4	1.2	16.6	127.6	2.8	27.3	146.7
2008-2012	1.0	33.4	102.7	4.4	54.3	137.5	0.8	16.4	114.7	1.2	17.9	100.3
	35-44			>74 y/o			35-44			>74 y/o		
1983-1987	4.4	46.1	144.9	1.6	77.1	98.5	1.0	12.3	103.8	1.8	55.1	95.5
1988-1992	2.4	29.0	122.6	1.2	55.0	85.3	0.2	8.1	83.8	2.0	47.1	98.4
1993-1997	2.0	30.1	122.8	1.8	57.3	110.1	0.8	10.0	118.0	0.8	21.4	62.3
1998-2002	2.4	43.1	122.4	1.8	52.0	98.7	0.8	12.0	116.3	1.8	29.4	94.7
2003-2007	1.6	39.3	98.0	3.0	56.9	126.5	0.2	11.2	89.1	1.6	21.6	96.4
2008-2012	2.0	39.8	107.1	2.4	44.3	106.9	0.4	13.0	95.6	2.4	24.4	127.1

Secondary Medical Zone ID: 167

	Male						Female					
	Suicide			Suicide			Suicide			Suicide		
	Num per year	Rate /100,000	×100	Num per year	Rate /100,000	×100	Num per year	Rate /100,000	×100	Num per year	Rate /100,000	×100
	Total (>10 y/o)			45-54			Total (>10 y/o)			45-54		
1983-1987	19.2	34.8	115.6	5.8	61.4	121.5	10.2	15.5	104.1	1.6	16.8	97.4
1988-1992	16.4	27.2	119.7	4.0	40.6	118.1	10.2	13.4	107.8	1.0	12.9	88.0
1993-1997	16.6	26.7	114.2	4.2	41.1	112.4	8.4	11.0	101.7	1.6	13.7	105.8
1998-2002	26.2	39.9	123.3	5.2	60.2	107.5	14.2	14.7	131.6	3.0	20.7	137.7
2003-2007	22.6	40.0	111.6	5.4	72.1	128.9	8.4	12.8	97.3	1.2	14.7	107.1
2008-2012	22.4	37.0	117.6	4.2	58.7	120.8	11.2	13.9	116.3	1.2	16.3	109.2
	15-24			55-64			15-24			55-64		
1983-1987	0.6	12.0	85.7	3.8	51.2	120.5	0.2	5.7	83.4	1.6	19.3	97.8
1988-1992	0.8	10.3	103.7	3.6	41.6	117.0	0.6	5.9	111.8	1.4	16.6	93.2
1993-1997	0.8	11.7	103.1	4.2	45.5	113.1	1.0	7.4	142.2	2.4	19.4	122.3
1998-2002	0.8	16.1	101.9	9.6	90.7	142.7	0.4	7.1	101.5	2.8	21.5	114.2
2003-2007	1.2	20.2	115.7	5.6	58.4	101.1	0.2	8.1	89.6	1.6	15.7	93.8
2008-2012	0.8	21.6	101.9	4.6	50.6	104.1	0.4	9.9	102.3	2.2	17.0	114.3
	25-34			65-74			25-34			65-74		
1983-1987	1.8	26.4	103.4	1.4	40.6	92.8	1.4	13.8	128.1	1.4	27.8	88.9
1988-1992	1.4	20.8	108.9	2.0	39.4	111.7	1.0	10.6	121.7	2.4	30.7	117.6
1993-1997	1.6	21.6	110.8	2.2	35.0	109.6	0.6	8.5	102.3	1.2	16.9	86.9
1998-2002	1.8	27.3	101.2	3.8	49.8	109.6	1.4	13.9	120.0	2.2	22.6	107.4
2003-2007	3.2	40.8	131.7	3.2	41.0	95.6	1.0	14.1	108.6	2.2	20.1	107.9
2008-2012	1.4	32.9	101.1	4.6	44.9	113.6	0.8	15.2	106.3	3.2	22.1	124.4
	35-44			>74 y/o			35-44			>74 y/o		
1983-1987	4.2	39.0	122.6	1.2	65.1	83.1	1.6	12.9	108.9	2.2	64.0	110.8
1988-1992	2.2	25.3	106.6	2.4	74.5	115.6	2.4	14.6	151.0	1.4	34.7	72.5
1993-1997	1.8	25.8	105.1	1.8	52.8	101.5	0.4	7.7	91.0	1.2	23.9	69.5
1998-2002	2.6	39.2	111.2	2.2	53.6	101.7	1.0	11.9	115.5	3.4	40.6	130.9
2003-2007	2.4	39.6	98.7	1.6	36.6	81.2	1.2	14.2	112.7	1.0	14.1	63.0
2008-2012	3.0	40.3	108.4	3.4	47.5	114.6	0.8	13.2	97.3	2.6	21.3	111.0

Secondary Medical Zone ID: 168

	Male						Female					
	Suicide			Suicide			Suicide			Suicide		
	Num per year	Rate /100,000	×100	Num per year	Rate /100,000	×100	Num per year	Rate /100,000	×100	Num per year	Rate /100,000	×100
	Total (>10 y/o)			45–54			Total (>10 y/o)			45–54		
1983–1987	72.4	29.3	88.7	19.2	45.4	89.7	35.0	13.5	86.3	7.4	17.5	101.7
1988–1992	62.0	22.5	90.0	15.2	32.0	93.2	29.8	10.4	77.6	4.8	11.5	78.8
1993–1997	67.0	22.3	87.8	17.2	33.0	90.3	29.4	9.2	81.9	5.8	11.6	90.2
1998–2002	106.4	33.3	92.1	25.0	49.0	87.6	37.4	10.9	85.1	6.4	13.3	88.3
2003–2007	113.8	35.3	96.0	27.4	59.9	107.1	39.4	11.3	90.8	6.2	14.0	102.0
2008–2012	114.4	35.1	103.0	22.2	51.9	106.7	39.4	11.7	91.0	4.8	12.8	86.0
	15–24			55–64			15–24			55–64		
1983–1987	4.0	10.5	75.1	11.0	38.4	90.4	2.8	6.8	99.9	4.6	15.8	80.2
1988–1992	6.0	11.8	118.7	11.2	31.3	88.1	1.4	3.8	71.7	5.4	15.3	85.6
1993–1997	6.2	12.8	113.3	15.6	38.7	96.0	1.2	3.6	68.4	6.8	16.2	102.1
1998–2002	3.8	11.5	72.7	28.2	62.9	98.9	1.8	5.7	81.5	7.8	17.5	92.8
2003–2007	4.6	14.8	84.6	26.0	52.8	91.6	2.8	9.0	100.2	8.8	17.2	102.9
2008–2012	6.2	20.6	97.0	24.0	48.6	100.0	1.6	7.0	72.1	6.0	12.8	86.1
	25–34			65–74			25–34			65–74		
1983–1987	8.2	19.3	75.4	6.2	41.3	94.2	4.8	10.8	100.7	5.4	27.3	87.5
1988–1992	7.2	16.4	85.8	5.0	28.0	79.6	2.4	6.6	76.4	4.8	20.5	78.5
1993–1997	7.8	16.9	86.5	6.4	26.3	82.6	2.2	6.1	73.0	4.0	14.3	73.5
1998–2002	12.2	24.5	90.9	14.8	47.1	103.7	3.8	9.2	79.3	5.0	15.5	73.8
2003–2007	12.4	26.5	85.5	16.8	47.0	109.6	3.8	9.8	75.7	7.6	19.1	102.7
2008–2012	14.2	33.9	103.9	18.8	46.1	116.7	4.8	13.5	94.2	6.6	15.9	89.3
	35–44			>74 y/o			35–44			>74 y/o		
1983–1987	17.8	32.6	102.6	6.0	73.3	93.6	4.4	9.2	77.9	5.6	45.2	78.4
1988–1992	11.8	22.2	93.6	5.6	55.3	85.7	5.4	10.2	105.7	5.4	32.7	68.3
1993–1997	9.0	19.6	80.2	4.8	40.2	77.2	3.0	7.1	84.1	5.8	26.9	78.1
1998–2002	16.2	36.0	102.3	5.8	38.9	73.7	4.0	9.8	94.4	8.6	30.5	98.1
2003–2007	19.6	41.5	103.6	7.0	34.2	76.1	2.6	7.9	62.9	7.6	21.1	94.2
2008–2012	17.8	35.4	95.2	11.0	40.3	97.2	6.2	13.3	97.9	9.4	21.1	109.6

Secondary Medical Zone ID: 169

	Male						Female					
	Suicide			Suicide			Suicide			Suicide		
	Num per year	Rate /100,000	×100	Num per year	Rate /100,000	×100	Num per year	Rate /100,000	×100	Num per year	Rate /100,000	×100
	Total (>10 y/o)			45–54			Total (>10 y/o)			45–54		
1983–1987	50.2	34.7	109.8	14.6	58.8	116.2	19.8	14.1	89.1	3.2	15.1	87.6
1988–1992	35.4	23.3	93.9	9.2	33.9	98.8	15.2	10.4	74.2	2.6	11.9	81.6
1993–1997	39.8	23.7	94.2	9.0	31.5	86.2	17.8	9.7	89.4	3.4	12.1	93.8
1998–2002	65.8	36.0	101.3	16.6	55.3	98.9	21.6	11.4	88.7	2.4	11.0	72.9
2003–2007	69.4	37.6	103.6	15.0	56.1	100.3	24.0	12.3	97.5	3.6	13.8	100.7
2008–2012	76.6	39.2	120.1	15.4	59.6	122.7	21.8	11.4	90.0	2.2	11.5	76.8
	15–24			55–64			15–24			55–64		
1983–1987	2.6	12.3	88.2	6.8	42.5	100.1	1.2	5.9	86.4	3.0	18.1	91.8
1988–1992	1.6	7.9	79.9	7.8	38.2	107.5	1.2	5.0	94.0	3.6	17.6	98.6
1993–1997	2.0	9.6	84.5	10.6	45.0	111.7	0.8	4.2	80.6	3.0	14.0	88.4
1998–2002	3.6	16.5	104.4	14.8	59.0	92.8	1.4	6.9	98.7	5.4	20.1	107.0
2003–2007	3.6	18.5	106.0	17.2	60.5	104.7	1.2	7.7	85.6	4.6	16.3	97.5
2008–2012	3.4	20.8	98.1	18.6	60.9	125.3	1.0	7.7	79.7	6.2	18.7	125.7
	25–34			65–74			25–34			65–74		
1983–1987	5.4	22.2	87.0	5.4	57.9	132.3	1.6	8.0	74.6	4.2	34.7	111.0
1988–1992	3.2	14.6	76.6	3.2	32.6	92.5	0.6	5.2	59.8	2.0	17.7	68.0
1993–1997	4.8	18.7	95.6	5.0	35.5	111.4	0.6	5.0	60.4	4.6	25.2	129.5
1998–2002	11.0	35.4	131.2	8.8	50.8	111.7	1.4	8.0	69.1	3.0	17.3	82.1
2003–2007	8.4	31.2	100.6	8.6	44.2	103.1	3.8	14.4	111.0	5.2	22.1	118.6
2008–2012	10.0	39.8	122.1	10.6	45.5	115.2	2.0	11.8	82.1	4.8	19.0	106.9
	35–44			>74 y/o			35–44			>74 y/o		
1983–1987	11.8	37.4	117.4	3.0	73.1	93.4	2.8	10.3	87.4	3.6	55.1	95.5
1988–1992	6.0	20.6	87.0	4.0	70.8	109.8	1.6	7.3	75.2	3.6	40.2	84.0
1993–1997	5.6	21.4	87.3	2.8	45.6	87.5	1.6	7.1	83.0	3.8	32.4	94.0
1998–2002	7.0	29.1	82.6	4.0	49.2	93.3	2.4	10.0	96.3	5.4	35.0	112.6
2003–2007	11.8	43.4	108.3	4.6	41.7	92.6	2.2	10.4	82.0	3.4	18.4	81.9
2008–2012	11.6	39.0	104.7	6.8	45.8	110.5	2.2	10.5	77.2	3.4	15.3	79.7

Secondary Medical Zone ID: 170

	Male						Female					
	Suicide			Suicide			Suicide			Suicide		
	Num per year	Rate /100,000	×100	Num per year	Rate /100,000	×100	Num per year	Rate /100,000	×100	Num per year	Rate /100,000	×100
	Total (>10 y/o)			45-54			Total (>10 y/o)			45-54		
1983-1987	80.8	28.3	84.7	20.2	40.5	80.0	38.0	12.4	78.0	7.2	14.9	86.7
1988-1992	68.2	22.0	87.7	16.2	30.0	87.5	39.4	11.2	85.6	7.6	14.1	96.7
1993-1997	80.6	23.7	94.8	21.2	36.2	98.9	34.4	9.3	82.5	5.8	10.6	82.4
1998-2002	113.2	32.2	90.1	29.6	53.7	96.0	38.0	10.2	77.0	8.2	14.9	98.9
2003-2007	111.4	33.6	88.3	22.8	48.6	86.9	35.2	10.4	74.6	3.8	9.6	70.1
2008-2012	104.8	31.8	90.5	18.6	43.2	88.9	44.2	12.3	92.0	6.0	14.1	94.2
	15-24			55-64			15-24			55-64		
1983-1987	6.4	13.4	95.9	13.2	38.7	91.1	2.6	5.6	81.7	6.4	17.2	87.4
1988-1992	4.6	8.9	89.9	14.8	34.6	97.5	2.6	4.9	93.0	8.0	18.0	100.9
1993-1997	4.6	9.6	84.5	20.0	42.5	105.6	2.4	4.9	93.0	6.6	14.2	89.3
1998-2002	6.4	15.1	95.3	30.6	61.3	96.4	2.6	6.4	91.5	9.0	17.7	94.1
2003-2007	7.0	18.6	106.8	29.0	54.0	93.6	3.8	10.1	112.6	7.8	14.6	87.4
2008-2012	6.4	20.3	95.7	20.4	40.6	83.5	3.2	10.1	103.7	7.8	14.6	97.9
	25-34			65-74			25-34			65-74		
1983-1987	11.4	23.6	92.2	5.2	31.3	71.6	4.4	9.2	85.2	5.2	22.2	70.9
1988-1992	7.0	15.7	81.9	9.0	39.8	113.1	3.2	7.3	84.3	5.4	19.0	73.0
1993-1997	9.2	19.0	97.1	7.6	26.8	84.1	4.0	8.3	99.2	6.0	17.2	88.3
1998-2002	9.4	20.0	74.1	15.2	42.8	94.1	3.4	8.1	69.8	4.0	11.9	56.4
2003-2007	14.8	31.1	100.4	12.0	31.8	74.1	5.0	11.3	86.7	5.8	14.1	75.7
2008-2012	13.2	32.8	100.6	17.6	40.1	101.6	5.4	14.0	97.5	6.6	14.6	81.9
	35-44			>74 y/o			35-44			>74 y/o		
1983-1987	17.2	28.1	88.4	7.0	69.3	88.4	6.4	10.6	89.7	5.6	37.5	65.0
1988-1992	9.4	17.2	72.6	7.2	57.4	88.9	4.8	8.7	89.7	7.6	36.7	76.6
1993-1997	9.8	20.4	83.0	8.2	54.4	104.4	3.4	7.3	86.2	6.2	23.9	69.4
1998-2002	12.2	27.4	77.9	9.6	51.7	98.0	2.8	7.3	70.4	8.0	24.2	77.9
2003-2007	19.0	39.5	98.5	6.6	28.7	63.6	4.0	9.6	76.3	5.0	12.7	56.5
2008-2012	17.4	34.3	92.1	11.2	36.3	87.4	7.0	13.8	102.0	8.2	16.3	84.9

Secondary Medical Zone ID: 171

	Male						Female					
	Suicide		×100	Suicide		×100	Suicide		×100	Suicide		×100
	Num per year	Rate /100,000		Num per year	Rate /100,000		Num per year	Rate /100,000		Num per year	Rate /100,000	
	Total (>10 y/o)			45-54			Total (>10 y/o)			45-54		
1983-1987	49.4	29.0	85.4	11.2	39.2	77.6	26.0	13.5	85.2	5.0	17.2	99.8
1988-1992	43.8	22.7	90.8	7.8	25.8	75.0	25.2	11.3	87.0	3.6	12.6	86.3
1993-1997	46.6	22.6	87.7	10.6	30.6	83.7	23.6	9.6	88.8	3.0	9.9	76.9
1998-2002	75.8	33.7	93.6	17.2	48.7	87.0	25.0	10.5	79.8	4.6	13.5	89.4
2003-2007	79.6	35.5	96.4	15.6	49.6	88.7	24.6	10.7	81.0	3.4	11.7	85.4
2008-2012	71.2	32.8	93.2	12.2	43.0	88.5	24.6	10.8	82.3	2.0	9.8	65.5
	15-24			55-64			15-24			55-64		
1983-1987	4.0	15.2	108.6	7.0	33.2	78.0	1.6	6.2	90.2	2.2	12.1	61.4
1988-1992	2.0	8.3	83.5	9.0	33.2	93.5	1.0	4.1	77.6	3.8	15.0	84.1
1993-1997	3.0	10.8	95.3	11.6	39.7	98.6	1.2	4.5	86.4	4.2	14.8	93.1
1998-2002	3.4	14.2	89.7	18.6	59.6	93.9	1.0	5.2	74.5	4.6	16.0	85.3
2003-2007	4.6	19.2	110.3	19.8	56.4	97.8	1.0	6.1	68.1	4.8	14.7	87.5
2008-2012	4.0	20.4	96.2	17.0	47.9	98.7	1.6	8.6	88.5	4.6	13.4	90.1
	25-34			65-74			25-34			65-74		
1983-1987	4.6	17.9	70.0	4.4	37.5	85.5	2.2	8.4	78.2	4.4	27.4	87.6
1988-1992	4.6	17.0	89.1	7.0	43.2	122.6	2.4	8.6	98.7	4.4	22.6	86.5
1993-1997	3.4	13.8	70.8	5.2	27.0	84.6	1.0	5.5	65.8	5.0	20.4	104.9
1998-2002	7.8	25.6	94.8	11.4	46.7	102.6	2.8	10.2	88.0	3.8	16.0	76.2
2003-2007	10.0	32.2	103.8	11.2	43.0	100.2	3.0	11.2	86.5	4.4	16.5	88.9
2008-2012	7.4	29.1	89.5	9.6	35.4	89.7	2.2	10.9	76.2	4.2	15.4	86.3
	35-44			>74 y/o			35-44			>74 y/o		
1983-1987	11.4	31.6	99.2	6.8	92.8	118.5	4.0	11.4	96.7	6.6	58.6	101.5
1988-1992	9.0	24.8	104.7	4.4	53.3	82.6	1.6	6.4	66.4	8.2	54.9	114.8
1993-1997	8.4	25.8	105.2	4.4	44.3	85.0	2.2	7.4	87.1	6.8	36.8	106.8
1998-2002	10.2	34.3	97.4	6.8	51.6	97.9	1.4	6.8	65.8	6.6	28.7	92.3
2003-2007	11.6	39.1	97.6	6.6	38.2	84.9	3.0	11.2	89.1	5.0	17.7	79.1
2008-2012	13.2	40.0	107.6	7.8	36.3	87.4	3.8	12.9	95.5	6.2	18.2	94.7

Secondary Medical Zone ID: 172

	Male						Female					
	Suicide			Suicide			Suicide			Suicide		
	Num per year	Rate /100,000	×100	Num per year	Rate /100,000	×100	Num per year	Rate /100,000	×100	Num per year	Rate /100,000	×100
	Total (>10 y/o)			45-54			Total (>10 y/o)			45-54		
1983-1987	51.2	31.5	94.6	12.0	47.7	94.4	28.2	14.9	98.7	2.8	13.4	77.6
1988-1992	39.4	22.0	87.2	5.8	23.2	67.6	26.4	12.2	97.6	3.8	14.5	98.9
1993-1997	40.2	21.2	80.5	10.0	30.7	84.0	23.0	10.0	93.3	3.8	12.4	96.2
1998-2002	56.8	28.3	74.2	13.8	39.8	71.1	27.8	11.8	93.0	4.8	14.4	95.7
2003-2007	62.4	30.3	77.3	13.6	42.1	75.3	23.0	10.8	81.1	3.6	12.2	89.2
2008-2012	65.0	31.2	86.1	12.4	43.5	89.6	22.0	10.8	79.4	2.4	10.8	72.6
	15-24			55-64			15-24			55-64		
1983-1987	3.6	15.3	109.4	7.2	36.3	85.4	0.4	4.2	61.2	4.8	20.3	102.8
1988-1992	2.8	10.2	102.8	7.6	32.2	90.7	1.0	4.5	84.9	3.8	16.0	89.8
1993-1997	2.8	10.2	90.5	7.4	31.9	79.1	1.4	5.1	97.9	4.0	15.7	98.9
1998-2002	2.6	11.7	74.0	12.6	48.6	76.5	2.0	7.4	105.0	5.4	19.7	104.5
2003-2007	5.0	19.2	109.8	12.4	40.2	69.7	2.2	9.3	103.0	4.6	15.5	92.5
2008-2012	5.0	22.7	107.1	14.6	41.8	86.0	1.4	8.1	83.8	4.4	13.6	91.2
	25-34			65-74			25-34			65-74		
1983-1987	8.0	25.9	101.3	5.6	46.0	105.0	2.4	9.1	84.5	4.6	29.2	93.3
1988-1992	5.6	19.9	104.2	4.8	34.0	96.5	2.0	8.0	92.1	3.2	18.9	72.3
1993-1997	6.2	21.5	109.9	5.6	30.5	95.6	0.8	5.4	64.4	4.6	20.3	104.3
1998-2002	6.2	21.8	80.6	9.8	45.6	100.2	2.2	9.4	81.1	3.6	16.6	78.8
2003-2007	9.6	29.4	94.8	7.8	36.5	85.0	2.0	9.1	70.3	2.2	12.1	64.8
2008-2012	8.8	30.1	92.4	8.0	34.7	87.8	3.0	12.5	87.1	3.6	15.6	87.8
	35-44			>74 y/o			35-44			>74 y/o		
1983-1987	8.6	26.7	83.8	6.0	83.4	106.5	3.4	11.4	96.2	9.8	87.0	150.7
1988-1992	7.2	20.5	86.4	5.6	63.5	98.5	2.6	8.5	88.2	10.0	67.4	140.9
1993-1997	5.0	16.7	68.2	3.0	33.3	63.9	2.0	7.1	83.6	6.4	35.8	104.0
1998-2002	7.6	27.1	76.8	4.2	36.5	69.2	2.6	9.6	92.6	7.2	32.0	103.1
2003-2007	9.4	33.0	82.4	4.6	30.3	67.3	3.0	11.7	92.7	5.4	19.9	88.5
2008-2012	9.6	32.1	86.2	6.6	34.1	82.1	2.2	10.2	75.1	5.0	16.2	84.2

Secondary Medical Zone ID: 173

	Male						Female					
	Suicide			Suicide			Suicide			Suicide		
	Num per year	Rate /100,000	×100	Num per year	Rate /100,000	×100	Num per year	Rate /100,000	×100	Num per year	Rate /100,000	×100
	Total (>10 y/o)			45–54			Total (>10 y/o)			45–54		
1983–1987	75.0	25.3	73.9	16.6	33.9	67.1	45.8	13.5	87.2	6.2	13.2	76.7
1988–1992	61.4	18.9	72.8	13.6	25.4	73.8	37.0	10.1	74.9	5.4	11.1	75.8
1993–1997	68.4	19.2	73.6	15.6	26.5	72.4	36.0	8.7	79.2	5.0	9.4	72.4
1998–2002	102.8	27.2	72.8	28.4	46.2	82.6	39.0	10.0	71.8	6.8	12.2	80.8
2003–2007	109.6	28.3	74.7	24.0	43.2	77.2	39.8	10.1	74.9	5.0	10.3	75.4
2008–2012	112.0	29.8	81.8	20.8	40.0	82.4	47.8	11.7	88.4	5.8	12.3	82.2
	15–24			55–64			15–24			55–64		
1983–1987	4.0	8.9	63.9	9.8	28.7	67.4	2.2	5.0	73.3	7.6	18.7	94.7
1988–1992	4.8	8.9	90.3	10.4	24.5	69.1	2.2	4.3	82.1	6.0	13.9	77.9
1993–1997	5.4	10.2	90.6	12.8	27.9	69.3	2.6	4.9	94.8	5.8	12.4	77.9
1998–2002	6.4	13.7	86.7	22.8	44.8	70.6	4.2	8.3	118.7	6.4	13.5	71.9
2003–2007	5.8	14.3	82.1	26.0	44.8	77.7	4.4	10.2	113.3	9.6	16.3	97.1
2008–2012	7.2	19.3	91.1	20.8	36.2	74.5	3.6	9.9	102.3	8.4	14.2	95.3
	25–34			65–74			25–34			65–74		
1983–1987	10.8	19.7	77.1	8.8	42.6	97.3	6.0	10.9	101.2	8.2	29.7	94.9
1988–1992	6.4	12.3	64.4	7.8	31.7	90.1	4.2	8.2	94.0	6.8	21.3	81.7
1993–1997	8.0	14.7	75.1	10.2	31.3	98.1	2.0	5.0	59.7	7.4	19.0	97.6
1998–2002	14.4	23.8	88.3	12.4	33.3	73.2	4.6	8.9	77.1	7.4	17.4	82.8
2003–2007	11.8	21.2	68.3	13.4	32.1	74.9	3.6	8.0	61.5	5.2	12.4	66.5
2008–2012	15.0	29.5	90.6	15.2	33.2	84.1	4.6	10.8	75.6	7.0	14.7	82.7
	35–44			>74 y/o			35–44			>74 y/o		
1983–1987	16.6	26.5	83.1	8.2	69.1	88.2	6.2	10.4	87.7	9.4	52.4	90.8
1988–1992	10.0	16.6	69.8	8.4	56.6	87.7	3.4	6.6	68.5	9.0	36.7	76.8
1993–1997	9.2	16.8	68.5	6.8	39.5	75.8	4.2	7.8	92.1	9.0	28.9	83.9
1998–2002	10.6	20.7	58.7	7.6	36.0	68.3	4.2	8.6	83.2	5.4	15.0	48.2
2003–2007	18.4	32.4	80.9	10.0	34.8	77.2	5.0	10.2	80.6	7.0	15.0	66.7
2008–2012	22.2	36.0	96.7	10.6	30.1	72.5	8.6	14.5	107.1	9.6	16.9	87.8

Secondary Medical Zone ID: 174

	Male						Female					
	Suicide			Suicide			Suicide			Suicide		
	Num per year	Rate /100,000	×100	Num per year	Rate /100,000	×100	Num per year	Rate /100,000	×100	Num per year	Rate /100,000	×100
	Total (>10 y/o)			45–54			Total (>10 y/o)			45–54		
1983–1987	246.4	29.7	89.6	57.0	40.2	79.6	135.0	15.3	99.8	22.2	15.7	91.1
1988–1992	199.6	22.1	89.1	47.4	30.7	89.4	129.2	12.9	102.0	18.6	12.5	85.6
1993–1997	216.8	22.3	88.9	56.6	33.9	92.7	108.0	10.3	93.6	19.0	11.9	92.4
1998–2002	331.6	32.7	90.8	76.2	48.8	87.3	144.4	13.0	102.3	24.2	16.0	106.6
2003–2007	310.6	29.9	82.3	56.6	41.6	74.3	146.0	13.0	103.4	19.2	14.6	106.2
2008–2012	323.4	31.5	91.1	57.4	42.2	86.9	133.6	12.1	93.6	17.4	13.5	90.4
	15–24			55–64			15–24			55–64		
1983–1987	22.8	13.6	97.5	34.8	38.5	90.6	10.8	6.9	100.5	21.8	20.9	106.1
1988–1992	15.8	8.9	89.8	38.0	33.1	93.1	10.0	5.9	112.3	21.0	17.4	97.6
1993–1997	16.8	10.2	89.8	46.8	36.6	90.8	8.4	5.3	102.3	19.4	14.7	92.4
1998–2002	22.4	15.7	99.3	83.4	59.8	94.1	9.2	6.9	97.9	26.0	18.3	97.4
2003–2007	17.6	14.3	82.1	74.2	48.7	84.4	11.8	9.8	109.1	27.0	17.6	105.0
2008–2012	22.0	19.9	94.0	67.8	46.5	95.7	8.8	8.3	85.4	19.6	13.7	91.9
	25–34			65–74			25–34			65–74		
1983–1987	36.6	23.5	92.1	23.8	47.2	107.9	15.6	10.6	98.8	16.6	25.3	81.1
1988–1992	25.8	16.6	86.7	16.0	28.3	80.2	14.6	9.8	112.5	20.6	26.1	100.2
1993–1997	22.6	14.0	71.9	24.8	32.7	102.5	14.4	9.2	110.2	11.2	12.5	63.9
1998–2002	40.2	23.6	87.3	40.0	42.0	92.4	20.6	12.4	107.3	22.2	20.2	96.1
2003–2007	44.6	27.1	87.3	44.0	40.4	94.1	22.2	13.9	106.6	21.6	17.5	94.2
2008–2012	46.4	31.5	96.7	44.6	37.8	95.8	19.4	13.9	97.1	24.8	18.6	104.7
	35–44			>74 y/o			35–44			>74 y/o		
1983–1987	49.6	27.7	87.1	20.8	81.1	103.5	19.0	11.0	92.9	28.4	74.9	129.9
1988–1992	34.2	20.6	86.9	22.2	67.6	104.8	17.4	10.7	111.0	26.4	50.7	106.0
1993–1997	31.4	22.1	90.1	17.6	46.6	89.6	14.2	10.2	120.2	21.4	32.9	95.6
1998–2002	45.0	32.0	91.0	23.2	49.1	93.2	17.2	12.5	120.9	24.8	30.0	96.5
2003–2007	47.6	30.7	76.6	25.0	39.4	87.5	19.0	12.8	101.0	24.6	23.2	103.2
2008–2012	56.8	33.1	89.0	27.4	34.3	82.7	20.8	12.8	94.1	22.6	17.4	90.4

Secondary Medical Zone ID: 175

	Male						Female					
	Suicide			Suicide			Suicide			Suicide		
	Num per year	Rate /100,000	×100	Num per year	Rate /100,000	×100	Num per year	Rate /100,000	×100	Num per year	Rate /100,000	×100
	Total (>10 y/o)			45–54			Total (>10 y/o)			45–54		
1983–1987	26.6	27.2	74.4	4.8	27.8	55.0	14.8	13.4	85.0	2.4	14.5	84.1
1988–1992	21.4	19.8	71.9	5.4	24.6	71.7	16.6	12.3	95.3	2.6	12.7	86.8
1993–1997	29.4	22.2	84.1	7.0	29.2	79.8	17.8	11.0	104.8	2.4	10.6	82.3
1998–2002	40.0	29.8	75.0	11.4	50.3	89.9	21.4	12.6	101.6	3.2	14.0	92.7
2003–2007	46.8	32.6	84.6	10.2	53.7	96.0	21.6	12.1	102.8	1.6	10.8	79.0
2008–2012	43.4	30.2	84.4	7.2	40.7	83.7	15.4	10.9	80.1	1.4	11.0	73.5
	15–24			55–64			15–24			55–64		
1983–1987	1.4	8.9	63.8	5.0	42.1	99.2	1.4	6.5	95.0	2.2	18.4	93.5
1988–1992	2.2	9.4	94.6	4.6	30.0	84.4	1.2	4.9	93.8	2.2	15.8	88.7
1993–1997	2.8	11.6	102.2	7.4	37.2	92.4	1.0	4.6	88.8	5.0	22.1	139.1
1998–2002	3.2	16.2	102.3	9.0	42.7	67.2	1.6	7.6	108.0	5.6	22.0	116.7
2003–2007	2.0	14.7	84.5	9.0	39.9	69.1	0.6	6.2	69.1	6.4	21.3	127.0
2008–2012	1.4	14.6	69.0	7.6	38.3	78.8	1.0	8.0	82.9	3.0	13.6	91.2
	25–34			65–74			25–34			65–74		
1983–1987	3.4	21.9	85.6	3.6	51.0	116.5	3.2	14.5	135.1	1.4	21.7	69.4
1988–1992	3.4	18.9	98.7	2.0	29.9	84.7	2.2	10.3	119.0	3.0	28.2	108.1
1993–1997	2.8	15.0	76.6	2.6	27.3	85.7	1.8	8.3	99.8	2.8	21.8	112.0
1998–2002	6.2	25.2	93.2	3.4	31.0	68.1	2.8	11.4	98.3	3.0	20.7	98.6
2003–2007	6.6	28.4	91.6	7.0	40.2	93.8	2.6	11.8	91.1	3.4	18.9	101.4
2008–2012	5.6	30.6	93.8	8.0	38.4	97.2	2.0	12.4	86.8	3.0	15.5	87.2
	35–44			>74 y/o			35–44			>74 y/o		
1983–1987	5.4	22.9	72.1	3.0	85.4	109.0	3.2	11.8	100.1	0.8	26.1	45.3
1988–1992	2.0	12.8	54.0	1.8	49.5	76.7	1.6	8.1	83.6	3.8	51.8	108.3
1993–1997	4.4	23.2	94.7	2.4	48.4	92.9	1.4	7.7	91.1	3.4	36.6	106.3
1998–2002	4.6	26.2	74.5	2.2	40.6	77.0	1.6	9.3	89.5	3.6	31.5	101.3
2003–2007	8.2	36.7	91.5	3.8	44.4	98.5	2.2	11.3	89.2	4.6	30.3	135.2
2008–2012	8.2	32.6	87.7	5.4	44.7	107.6	2.2	10.9	80.5	2.8	17.0	88.4

Secondary Medical Zone ID: 176

	Male						Female					
	Suicide			Suicide			Suicide			Suicide		
	Num per year	Rate /100,000	×100	Num per year	Rate /100,000	×100	Num per year	Rate /100,000	×100	Num per year	Rate /100,000	×100
	Total (>10 y/o)			45-54			Total (>10 y/o)			45-54		
1983-1987	17.0	31.2	94.3	4.6	46.1	91.2	7.2	14.6	89.9	1.0	14.6	84.9
1988-1992	11.4	21.8	80.4	3.2	29.3	85.2	5.6	11.2	79.6	1.2	13.1	89.5
1993-1997	14.6	23.4	88.3	3.0	29.4	80.2	7.4	10.7	98.0	1.8	13.5	104.2
1998-2002	21.4	32.4	85.6	4.4	46.3	82.7	8.2	12.2	90.9	1.0	12.8	85.1
2003-2007	23.2	33.0	88.8	4.4	51.7	92.5	10.0	12.7	102.3	1.4	14.4	105.0
2008-2012	20.8	31.2	84.7	3.4	43.1	88.7	11.0	13.1	105.4	1.0	14.0	93.7
	15-24			55-64			15-24			55-64		
1983-1987	0.8	10.2	73.2	3.0	48.9	115.1	0.4	5.7	83.1	1.8	23.9	121.1
1988-1992	1.2	9.5	96.0	2.2	32.1	90.4	0.2	3.9	73.5	1.6	19.5	109.3
1993-1997	1.8	12.4	109.6	2.8	33.9	84.2	0.2	3.9	75.2	0.6	12.0	75.5
1998-2002	0.4	11.0	69.9	6.4	59.6	93.7	0.4	6.1	87.5	1.0	15.1	80.0
2003-2007	1.4	17.0	97.6	5.6	51.5	89.2	0.4	7.6	84.7	1.4	14.8	88.5
2008-2012	1.0	18.1	85.1	4.2	44.6	91.8	0.6	9.2	95.2	2.4	17.3	116.0
	25-34			65-74			25-34			65-74		
1983-1987	2.8	27.3	106.8	1.0	41.0	93.6	0.8	9.8	90.7	0.6	25.0	80.0
1988-1992	0.6	12.0	62.7	0.6	28.9	82.1	1.2	10.1	116.3	0.4	18.6	71.2
1993-1997	2.6	20.9	107.2	0.8	25.4	79.5	1.2	9.4	112.5	1.6	24.5	125.7
1998-2002	4.2	29.4	108.9	2.8	45.2	99.3	1.6	12.1	104.7	1.4	21.4	102.0
2003-2007	2.8	25.5	82.1	4.8	52.4	122.2	1.6	13.0	100.1	2.2	22.1	118.5
2008-2012	2.8	28.3	87.0	3.8	39.3	99.5	1.6	14.5	101.0	1.8	17.8	100.2
	35-44			>74 y/o			35-44			>74 y/o		
1983-1987	4.0	30.4	95.6	0.8	70.1	89.5	1.2	10.6	89.6	1.4	63.5	110.1
1988-1992	1.8	19.1	80.6	1.6	76.1	118.1	0.8	8.7	89.7	0.2	23.9	50.0
1993-1997	2.4	25.1	102.4	1.2	55.0	105.6	0.8	8.6	100.6	1.2	33.7	98.0
1998-2002	1.6	24.8	70.5	1.6	57.0	108.2	1.8	13.9	134.0	1.0	25.5	82.1
2003-2007	2.6	28.8	71.9	1.6	44.6	99.1	1.8	14.5	115.0	1.2	21.3	95.1
2008-2012	4.0	32.7	88.0	1.6	36.4	87.8	1.6	13.3	98.3	2.0	23.3	121.1

Secondary Medical Zone ID: 177

	Male						Female					
	Suicide			Suicide			Suicide			Suicide		
	Num per year	Rate /100,000	×100	Num per year	Rate /100,000	×100	Num per year	Rate /100,000	×100	Num per year	Rate /100,000	×100
	Total (>10 y/o)			45-54			Total (>10 y/o)			45-54		
1983-1987	31.6	27.7	77.1	7.2	34.1	67.3	23.2	17.4	113.1	4.6	19.8	114.7
1988-1992	30.0	21.8	82.2	7.6	28.0	81.6	22.4	13.4	107.8	4.4	15.5	106.0
1993-1997	36.2	22.0	83.6	8.2	28.3	77.3	19.0	10.2	94.5	4.2	13.0	100.4
1998-2002	54.6	30.4	79.3	10.0	38.1	68.1	26.8	12.4	101.2	4.2	14.1	93.7
2003-2007	56.2	28.9	77.0	7.2	32.0	57.2	26.4	11.9	97.4	3.8	14.1	103.1
2008-2012	58.6	29.8	83.2	10.2	40.6	83.5	27.6	12.5	97.4	3.6	14.3	95.8
	15-24			55-64			15-24			55-64		
1983-1987	2.8	10.9	78.5	5.2	39.5	93.1	1.2	5.6	82.4	2.6	19.0	96.1
1988-1992	1.8	6.9	69.8	4.4	26.1	73.6	1.2	4.3	82.1	3.0	17.1	95.8
1993-1997	3.8	11.0	96.8	7.0	31.9	79.3	1.2	4.3	81.6	2.0	11.5	72.1
1998-2002	5.0	15.8	100.2	12.0	45.5	71.6	1.4	5.8	82.7	4.6	17.5	92.9
2003-2007	3.0	13.1	75.0	12.8	43.5	75.4	1.0	5.9	65.4	4.6	15.2	90.5
2008-2012	5.0	20.2	95.3	11.2	41.4	85.1	2.2	9.5	98.3	4.0	13.6	91.5
	25-34			65-74			25-34			65-74		
1983-1987	4.6	23.9	93.4	2.2	36.3	83.0	1.8	9.4	87.2	4.4	41.4	132.6
1988-1992	4.6	20.0	104.4	3.6	38.5	109.2	2.0	8.7	100.2	2.8	25.2	96.5
1993-1997	4.8	17.6	90.1	4.2	33.1	103.8	0.8	5.1	61.6	2.0	16.0	82.1
1998-2002	8.8	26.4	97.7	5.2	34.9	76.8	1.6	7.4	63.5	5.4	27.5	131.1
2003-2007	9.4	29.2	94.2	8.4	40.2	93.8	3.8	12.2	93.9	6.8	27.3	146.4
2008-2012	7.8	29.1	89.3	8.0	32.9	83.4	3.2	12.7	88.8	5.6	20.0	112.1
	35-44			>74 y/o			35-44			>74 y/o		
1983-1987	5.8	21.6	67.9	3.6	98.9	126.3	2.2	8.8	74.8	6.4	112.3	194.6
1988-1992	3.4	15.0	63.2	4.4	84.1	130.4	2.4	8.8	91.3	6.6	76.8	160.6
1993-1997	4.6	19.9	81.2	3.6	58.7	112.8	2.2	8.6	101.4	6.6	57.7	167.6
1998-2002	9.4	34.8	98.8	4.0	52.4	99.3	3.4	12.0	116.3	6.2	43.0	138.4
2003-2007	9.2	30.3	75.7	6.2	54.0	119.9	2.0	8.9	70.4	4.4	24.7	110.0
2008-2012	8.6	26.4	71.0	7.0	45.9	110.7	4.4	12.8	94.2	4.4	19.8	103.0

Secondary Medical Zone ID: 178

	Male						Female					
	Suicide			Suicide			Suicide			Suicide		
	Num per year	Rate /100,000	×100	Num per year	Rate /100,000	×100	Num per year	Rate /100,000	×100	Num per year	Rate /100,000	×100
	Total (>10 y/o)			45–54			Total (>10 y/o)			45–54		
1983–1987	46.0	27.2	78.5	12.0	37.6	74.4	31.6	15.9	106.2	5.0	16.4	95.1
1988–1992	39.4	21.1	81.4	9.4	26.5	77.3	27.6	12.5	98.7	6.0	15.8	108.4
1993–1997	36.2	18.8	68.0	8.6	24.8	67.7	27.8	11.1	104.9	5.8	13.9	108.0
1998–2002	67.2	30.5	80.7	17.2	49.1	87.7	32.8	12.4	100.5	6.0	15.7	104.1
2003–2007	69.2	30.6	80.7	11.4	39.4	70.4	27.4	11.3	86.5	4.4	13.9	101.1
2008–2012	63.6	28.8	79.6	11.6	40.6	83.6	29.2	11.5	89.9	3.6	13.0	86.9
	15–24			55–64			15–24			55–64		
1983–1987	3.2	10.1	72.7	7.2	38.6	90.9	2.2	6.1	88.9	5.4	23.8	120.4
1988–1992	5.6	12.9	129.9	4.6	21.2	59.7	2.2	5.3	99.9	3.6	15.5	86.9
1993–1997	4.2	11.5	101.3	7.4	26.7	66.3	2.2	5.6	106.7	6.8	20.6	129.4
1998–2002	3.8	13.7	86.6	14.4	43.7	68.8	2.2	7.1	100.9	5.4	16.4	87.2
2003–2007	4.8	17.8	102.3	14.8	42.1	73.0	1.6	7.1	79.1	6.4	16.5	98.4
2008–2012	4.0	18.0	84.9	11.0	35.3	72.6	0.0	3.5	35.9	2.4	9.6	64.1
	25–34			65–74			25–34			65–74		
1983–1987	5.8	20.7	80.9	3.2	34.4	78.5	3.4	10.9	101.3	6.6	41.5	132.6
1988–1992	5.2	17.7	92.4	4.4	35.6	101.1	2.2	7.7	89.2	3.4	21.8	83.7
1993–1997	4.0	13.4	68.8	3.0	21.4	67.1	3.4	9.3	112.0	2.8	15.8	81.2
1998–2002	10.0	26.1	96.8	8.0	38.4	84.3	4.0	10.9	93.7	6.8	26.8	127.6
2003–2007	11.0	30.5	98.4	10.0	37.7	87.8	2.4	8.7	67.3	4.4	16.5	88.4
2008–2012	7.4	27.1	83.1	11.0	35.8	90.7	6.2	18.6	129.9	6.6	19.1	107.5
	35–44			>74 y/o			35–44			>74 y/o		
1983–1987	10.4	26.6	83.6	3.8	71.9	91.8	5.4	12.6	106.8	3.6	48.2	83.6
1988–1992	5.0	15.6	65.8	5.2	72.3	112.1	3.8	9.9	102.7	6.2	54.5	113.9
1993–1997	5.6	19.2	78.2	3.0	40.4	77.6	2.4	7.9	93.0	4.4	31.2	90.6
1998–2002	8.6	28.8	81.7	5.2	51.7	98.1	2.6	9.1	88.0	5.8	32.4	104.2
2003–2007	12.0	34.5	86.2	5.0	37.9	84.1	3.8	11.5	91.4	4.0	18.6	82.8
2008–2012	11.4	30.3	81.4	7.2	38.9	93.9	4.2	11.7	86.1	6.2	21.6	112.1

Secondary Medical Zone ID: 179

	Male						Female					
	Suicide			Suicide			Suicide			Suicide		
	Num per year	Rate /100,000	×100	Num per year	Rate /100,000	×100	Num per year	Rate /100,000	×100	Num per year	Rate /100,000	×100
	Total (>10 y/o)			45–54			Total (>10 y/o)			45–54		
1983–1987	65.4	29.7	85.5	16.4	38.8	76.8	41.0	17.1	112.4	6.0	16.0	92.6
1988–1992	55.8	22.3	85.6	13.2	26.2	76.4	36.8	13.0	103.9	7.0	14.4	98.6
1993–1997	57.8	20.2	77.1	17.8	32.2	88.1	30.4	9.8	89.7	5.2	10.1	78.3
1998–2002	97.2	30.3	82.8	23.0	46.5	83.1	44.6	12.5	101.7	5.8	12.1	80.2
2003–2007	107.4	32.4	88.1	20.6	49.6	88.7	46.0	12.2	103.3	4.6	11.6	84.8
2008–2012	111.6	32.9	96.3	19.8	48.2	99.1	47.8	12.8	104.0	5.4	13.9	93.3
	15–24			55–64			15–24			55–64		
1983–1987	4.8	10.8	77.5	9.0	39.3	92.5	3.4	7.1	104.1	5.8	22.6	114.5
1988–1992	3.6	7.4	74.2	9.6	30.2	85.1	4.2	7.0	133.4	4.8	16.3	91.1
1993–1997	4.8	9.4	83.4	11.8	29.9	74.2	1.8	3.9	75.4	4.6	12.9	81.0
1998–2002	6.2	14.2	89.6	25.6	52.3	82.4	2.8	6.7	94.9	8.8	18.4	97.9
2003–2007	6.2	16.4	94.2	24.4	46.6	80.8	2.4	7.3	81.7	11.2	19.3	115.4
2008–2012	7.4	20.9	98.6	21.4	44.9	92.5	4.0	11.3	116.2	5.8	12.2	81.9
	25–34			65–74			25–34			65–74		
1983–1987	8.2	21.7	85.1	6.0	48.3	110.4	2.8	7.9	73.3	8.4	46.5	148.9
1988–1992	5.0	13.2	68.8	6.2	40.1	113.9	2.0	6.1	70.2	4.6	24.4	93.4
1993–1997	5.6	12.5	64.1	6.2	30.4	95.2	3.8	8.1	97.1	4.6	19.6	100.8
1998–2002	10.8	19.9	73.8	12.6	44.5	97.8	5.8	10.8	93.4	7.8	25.5	121.2
2003–2007	15.4	28.1	90.5	16.0	43.3	100.9	6.8	12.9	99.4	8.4	22.0	118.4
2008–2012	12.4	28.0	85.8	18.0	40.1	101.6	4.6	11.7	81.5	10.4	21.6	121.3
	35–44			>74 y/o			35–44			>74 y/o		
1983–1987	13.2	23.9	75.2	7.2	109.4	139.7	6.6	11.4	96.9	7.6	83.7	145.1
1988–1992	9.4	19.1	80.8	8.4	96.6	149.8	4.4	8.7	90.2	9.8	73.0	152.5
1993–1997	6.2	15.9	64.8	5.4	54.8	105.1	2.2	6.1	71.2	7.8	44.7	129.8
1998–2002	11.6	28.0	79.6	7.0	55.2	104.6	3.2	8.5	82.2	9.8	43.0	138.5
2003–2007	14.8	30.5	76.1	9.8	53.2	118.2	3.8	9.4	74.1	8.8	30.3	135.0
2008–2012	21.0	35.7	95.9	11.4	46.0	111.0	7.2	13.2	97.1	10.2	27.3	142.2

Secondary Medical Zone ID: 180

	Male						Female					
	Suicide			Suicide			Suicide			Suicide		
	Num per year	Rate /100,000	×100	Num per year	Rate /100,000	×100	Num per year	Rate /100,000	×100	Num per year	Rate /100,000	×100
	Total (>10 y/o)			45–54			Total (>10 y/o)			45–54		
1983–1987	56.4	28.6	84.4	15.0	42.4	83.9	34.0	15.8	103.7	3.8	13.1	75.8
1988–1992	46.0	21.1	82.3	11.2	27.8	81.0	33.2	13.1	105.5	6.0	15.2	103.7
1993–1997	55.2	22.3	87.1	13.6	31.0	84.8	36.4	12.3	121.0	5.8	13.1	101.7
1998–2002	86.6	32.2	88.8	18.4	44.8	80.1	40.4	13.1	109.3	5.6	13.9	92.5
2003–2007	99.4	34.7	96.7	17.8	48.6	87.0	39.4	12.8	106.0	3.0	10.2	74.5
2008–2012	87.4	32.2	88.9	18.4	50.3	103.5	32.6	11.0	88.0	4.6	13.9	93.2
	15–24			55–64			15–24			55–64		
1983–1987	5.6	13.6	97.8	8.0	37.1	87.2	3.0	7.2	105.0	3.2	15.6	78.9
1988–1992	4.0	9.1	91.5	9.8	33.8	95.2	2.2	5.0	94.8	5.8	19.5	109.4
1993–1997	3.8	9.4	82.8	12.2	36.2	90.0	1.6	4.2	80.6	4.6	14.4	90.9
1998–2002	5.4	14.7	93.1	20.4	53.0	83.5	2.4	6.7	95.8	7.2	18.9	100.5
2003–2007	5.8	16.9	96.8	21.4	50.4	87.3	4.6	12.4	138.1	7.8	17.8	106.3
2008–2012	8.6	24.2	114.3	13.4	35.6	73.3	2.2	8.0	82.4	5.2	13.3	89.3
	25–34			65–74			25–34			65–74		
1983–1987	9.4	26.2	102.6	4.6	41.4	94.6	5.2	13.9	129.4	6.0	36.8	117.7
1988–1992	5.8	16.4	85.8	3.8	29.3	83.1	1.4	5.7	65.8	5.6	29.7	114.0
1993–1997	7.8	19.1	97.7	5.4	28.8	90.1	4.4	10.5	126.7	6.2	25.9	132.7
1998–2002	10.0	22.9	84.8	12.8	50.2	110.5	5.4	12.5	108.0	6.8	24.4	116.1
2003–2007	13.8	30.0	96.8	15.6	50.8	118.4	4.6	11.7	89.7	6.0	19.2	103.0
2008–2012	14.6	34.7	106.5	11.0	33.1	83.8	2.6	9.4	65.8	5.4	15.9	89.5
	35–44			>74 y/o			35–44			>74 y/o		
1983–1987	9.6	22.4	70.4	4.0	68.5	87.5	5.2	11.7	98.9	7.4	74.3	128.7
1988–1992	6.0	15.6	65.7	5.0	64.1	99.4	2.6	7.4	76.4	9.4	68.4	142.9
1993–1997	6.2	17.9	72.8	6.2	64.9	124.7	3.6	9.5	112.1	10.0	57.5	167.2
1998–2002	12.4	33.7	95.6	7.2	60.1	114.1	3.2	9.6	92.7	9.8	45.5	146.7
2003–2007	15.0	36.3	90.6	9.6	57.1	126.9	4.6	12.2	97.0	8.8	32.4	144.4
2008–2012	15.0	32.2	86.7	6.2	31.7	76.5	3.8	10.2	75.3	8.6	25.6	132.9

Secondary Medical Zone ID: 181

	Male						Female					
	Suicide			Suicide			Suicide			Suicide		
	Num per year	Rate /100,000	×100	Num per year	Rate /100,000	×100	Num per year	Rate /100,000	×100	Num per year	Rate /100,000	×100
	Total (>10 y/o)			45–54			Total (>10 y/o)			45–54		
1983–1987	33.8	27.1	75.3	8.8	43.0	85.0	17.8	14.7	91.3	3.2	17.4	100.7
1988–1992	32.8	21.9	82.6	8.6	31.1	90.6	22.6	14.0	113.4	3.6	14.8	101.5
1993–1997	41.2	22.7	88.7	9.8	30.5	83.3	23.4	12.1	118.8	3.0	11.0	85.2
1998–2002	59.8	30.7	81.8	13.2	42.3	75.6	26.4	12.7	105.8	3.2	12.0	80.0
2003–2007	63.8	30.5	80.0	13.4	47.0	84.0	23.4	11.6	92.5	3.6	13.3	97.2
2008–2012	59.2	28.0	77.4	6.2	26.6	54.8	22.6	11.0	86.9	3.4	13.9	92.9
	15–24			55–64			15–24			55–64		
1983–1987	3.2	10.1	72.3	3.8	34.3	80.7	1.6	6.6	96.4	1.4	14.9	75.7
1988–1992	2.8	7.7	77.7	4.0	27.1	76.4	1.8	5.5	105.2	1.8	14.5	81.0
1993–1997	5.6	12.9	114.3	7.2	35.8	89.0	3.0	7.6	145.7	4.8	21.8	137.3
1998–2002	5.2	14.9	94.0	12.8	50.5	79.4	2.6	8.2	117.0	5.6	21.6	114.8
2003–2007	6.6	18.8	108.0	14.0	45.9	79.5	2.0	8.3	92.0	6.6	20.0	119.3
2008–2012	4.6	16.7	78.9	12.8	42.4	87.4	1.0	6.2	64.2	2.8	11.4	76.4
	25–34			65–74			25–34			65–74		
1983–1987	5.6	19.7	77.1	2.0	35.4	80.9	0.8	5.7	53.2	2.8	31.6	101.3
1988–1992	3.2	11.4	59.9	3.0	36.4	103.4	2.0	8.1	92.9	3.4	30.6	117.2
1993–1997	6.6	18.0	92.0	2.8	27.5	86.1	2.2	7.9	94.6	2.4	19.2	98.7
1998–2002	9.2	23.1	85.4	6.4	44.6	98.2	3.4	10.8	92.8	3.4	22.1	105.2
2003–2007	9.6	24.1	77.6	5.6	33.4	77.8	2.0	8.5	65.0	2.6	16.2	87.1
2008–2012	11.2	29.7	91.1	6.8	31.6	80.0	2.2	10.1	70.6	3.4	16.3	91.6
	35–44			>74 y/o			35–44			>74 y/o		
1983–1987	6.6	21.3	66.9	3.4	87.7	111.9	2.8	10.0	84.7	5.0	86.1	149.2
1988–1992	5.6	17.8	75.2	5.2	96.3	149.3	2.6	8.8	91.3	7.4	90.4	188.8
1993–1997	5.8	20.2	82.5	3.4	57.9	111.3	2.2	8.1	95.5	5.8	56.9	165.3
1998–2002	7.2	25.3	71.8	5.2	67.0	127.0	2.4	9.5	92.0	5.8	45.3	145.9
2003–2007	10.4	30.8	76.8	4.2	43.5	96.7	2.6	10.3	81.8	3.6	23.6	105.1
2008–2012	12.4	32.6	87.6	5.2	41.2	99.3	4.0	12.4	91.8	5.8	28.0	145.8

Secondary Medical Zone ID: 182

	Male						Female					
	Suicide			Suicide			Suicide			Suicide		
	Num per year	Rate /100,000	×100	Num per year	Rate /100,000	×100	Num per year	Rate /100,000	×100	Num per year	Rate /100,000	×100
	Total (>10 y/o)			45–54			Total (>10 y/o)			45–54		
1983–1987	30.2	27.8	78.0	8.4	44.7	88.4	21.6	16.2	107.2	3.4	17.7	103.0
1988–1992	27.6	21.3	82.1	5.8	26.8	78.0	16.2	11.6	86.9	3.6	15.6	107.0
1993–1997	34.0	22.5	87.3	9.6	34.7	94.9	20.4	11.5	109.6	4.0	14.2	110.1
1998–2002	51.2	31.3	83.6	14.0	50.1	89.5	23.6	12.6	101.3	4.2	15.1	100.5
2003–2007	54.8	31.0	82.2	10.6	42.6	76.2	18.8	11.2	82.9	2.2	10.8	79.0
2008–2012	52.8	29.9	82.5	11.2	44.8	92.2	22.2	12.4	91.6	2.6	12.4	83.2
	15–24			55–64			15–24			55–64		
1983–1987	2.2	11.4	81.8	3.0	29.4	69.3	1.2	6.0	88.3	2.6	18.6	94.3
1988–1992	2.6	9.8	98.6	3.2	23.8	67.1	0.2	2.8	52.2	2.6	16.4	91.6
1993–1997	2.8	10.6	93.5	5.6	32.6	81.0	1.0	4.3	83.3	2.8	15.3	96.1
1998–2002	2.8	13.0	82.2	12.2	56.3	88.6	1.8	7.2	102.8	3.4	17.2	91.2
2003–2007	4.2	17.8	102.2	11.2	44.4	76.9	1.4	7.4	82.4	3.0	13.8	82.4
2008–2012	3.4	18.1	85.3	8.8	35.9	73.8	2.4	10.9	112.1	3.8	14.3	95.6
	25–34			65–74			25–34			65–74		
1983–1987	4.4	20.2	79.0	2.6	39.2	89.4	1.6	8.3	77.3	4.0	37.4	119.8
1988–1992	3.8	16.1	84.2	2.0	28.3	80.3	2.2	9.0	103.9	2.8	24.9	95.4
1993–1997	4.6	17.4	89.0	3.6	31.9	99.9	2.4	8.9	107.3	3.8	24.7	126.7
1998–2002	6.0	21.5	79.5	4.0	33.4	73.4	3.2	11.4	98.8	2.4	17.0	80.8
2003–2007	9.4	29.6	95.3	8.6	49.3	115.0	3.8	13.2	101.3	3.2	18.1	97.4
2008–2012	9.2	31.9	97.8	5.0	29.4	74.5	4.4	16.0	111.6	2.2	13.9	78.3
	35–44			>74 y/o			35–44			>74 y/o		
1983–1987	5.4	21.6	67.8	4.2	98.5	125.7	3.0	11.5	97.0	5.6	87.4	151.5
1988–1992	6.8	23.4	98.8	3.2	65.3	101.3	1.0	6.3	65.0	3.6	45.5	95.0
1993–1997	4.6	18.9	77.1	3.0	53.1	102.0	2.0	8.1	95.7	4.4	42.0	121.9
1998–2002	7.4	29.6	84.0	4.6	60.8	115.4	3.2	11.9	115.1	5.2	38.3	123.3
2003–2007	7.8	28.8	71.9	3.0	34.5	76.6	2.8	11.5	90.8	2.2	15.1	67.2
2008–2012	8.6	29.1	78.2	6.4	48.0	115.6	3.4	12.4	91.3	3.4	17.4	90.4

Secondary Medical Zone ID: 183

	Male						Female					
	Suicide		×100	Suicide		×100	Suicide		×100	Suicide		×100
	Num per year	Rate /100,000		Num per year	Rate /100,000		Num per year	Rate /100,000		Num per year	Rate /100,000	
	Total (>10 y/o)			45–54			Total (>10 y/o)			45–54		
1983–1987	58.0	28.6	84.7	13.0	40.1	79.2	33.8	15.3	100.4	5.4	16.8	97.2
1988–1992	44.0	20.0	76.7	10.8	29.1	84.7	31.0	12.3	97.6	4.6	13.4	91.3
1993–1997	54.6	21.7	83.7	10.4	25.7	70.3	33.2	11.4	109.7	5.0	12.3	95.1
1998–2002	83.2	30.2	82.1	16.2	38.6	69.0	37.4	12.5	99.8	6.6	15.5	102.9
2003–2007	84.2	29.3	77.0	18.0	45.2	80.9	32.8	11.4	87.6	5.4	14.1	102.9
2008–2012	94.0	30.8	87.9	18.0	44.3	91.2	34.8	11.6	89.6	5.2	14.2	95.4
	15–24			55–64			15–24			55–64		
1983–1987	5.2	11.6	83.0	9.0	40.5	95.2	2.2	5.9	85.7	6.4	24.3	123.1
1988–1992	3.6	7.9	79.6	7.2	26.9	75.9	2.8	5.9	112.6	5.0	17.6	98.4
1993–1997	5.8	11.9	105.1	10.4	33.8	84.1	3.0	6.4	122.0	7.2	20.7	130.4
1998–2002	7.0	16.8	106.6	17.0	49.1	77.3	3.0	7.7	109.0	6.4	18.3	97.1
2003–2007	5.6	15.4	88.4	14.6	37.4	64.8	1.4	5.6	61.8	4.2	12.4	74.1
2008–2012	6.2	17.6	83.2	16.8	40.7	83.9	1.4	5.9	60.6	4.2	11.7	78.6
	25–34			65–74			25–34			65–74		
1983–1987	8.4	21.1	82.5	3.8	35.0	79.8	2.4	7.6	70.9	5.2	31.2	99.7
1988–1992	7.8	17.7	92.6	4.0	30.0	85.2	2.0	6.3	73.1	3.8	21.5	82.3
1993–1997	9.2	18.4	94.4	4.6	25.4	79.7	3.0	7.4	89.0	4.0	18.1	93.0
1998–2002	15.0	26.9	99.5	10.2	42.4	93.3	6.0	12.3	106.0	5.2	19.8	94.4
2003–2007	12.6	23.8	76.7	10.4	38.2	89.1	5.4	11.9	91.7	5.6	18.7	100.3
2008–2012	13.4	27.8	85.4	11.0	35.7	90.4	4.6	12.1	84.8	7.0	20.0	112.4
	35–44			>74 y/o			35–44			>74 y/o		
1983–1987	13.8	30.3	95.1	4.8	73.8	94.2	4.6	11.1	93.7	7.4	69.7	120.8
1988–1992	5.6	13.9	58.8	4.8	59.7	92.6	4.8	10.8	111.5	8.0	56.2	117.4
1993–1997	8.0	20.0	81.5	5.8	59.2	113.7	3.0	8.1	95.0	7.8	43.9	127.7
1998–2002	10.0	24.8	70.4	7.6	61.4	116.4	3.4	9.3	90.3	6.8	31.8	102.5
2003–2007	15.0	31.2	77.8	8.0	47.8	106.1	4.6	11.3	89.4	5.4	20.4	90.9
2008–2012	19.2	35.1	94.5	9.0	41.9	100.9	6.2	13.0	96.2	6.0	18.4	95.7

Secondary Medical Zone ID: 184

	Male						Female					
	Suicide			Suicide			Suicide			Suicide		
	Num per year	Rate /100,000	×100	Num per year	Rate /100,000	×100	Num per year	Rate /100,000	×100	Num per year	Rate /100,000	×100
	Total (>10 y/o)			45–54			Total (>10 y/o)			45–54		
1983–1987	10.8	33.3	103.0	2.2	48.6	96.0	6.2	15.4	101.9	0.2	13.8	80.0
1988–1992	6.0	22.6	81.5	1.2	31.4	91.4	5.8	13.1	104.8	0.6	14.4	98.2
1993–1997	8.0	24.7	96.9	2.6	42.0	114.9	3.6	10.1	88.3	0.2	11.2	86.8
1998–2002	9.0	32.8	83.3	1.8	46.8	83.6	5.6	12.7	104.3	1.6	18.6	123.8
2003–2007	9.6	34.5	87.5	1.2	41.2	73.8	5.2	12.7	105.4	0.6	13.7	100.0
2008–2012	9.8	34.5	96.9	2.2	52.6	108.3	3.4	12.6	92.6	0.2	13.1	88.0
	15–24			55–64			15–24			55–64		
1983–1987	1.4	20.6	147.6	1.0	33.5	78.9	0.2	6.6	96.9	1.0	19.3	97.9
1988–1992	0.6	10.9	109.8	1.4	32.4	91.2	0.0	4.5	85.1	1.2	19.2	107.3
1993–1997	0.0	9.5	83.8	1.2	35.0	86.8	0.4	6.0	115.7	0.8	16.0	100.7
1998–2002	0.4	14.9	94.5	1.8	54.1	85.2	0.4	7.7	109.2	0.8	18.6	98.9
2003–2007	0.8	18.8	108.0	1.6	46.3	80.2	0.4	9.8	109.0	0.4	15.2	90.8
2008–2012	0.6	21.6	102.0	1.8	43.8	90.2	0.4	10.8	111.5	0.6	14.4	96.9
	25–34			65–74			25–34			65–74		
1983–1987	1.2	26.6	103.9	1.8	50.3	115.0	0.4	10.5	97.4	1.0	29.1	93.1
1988–1992	0.8	20.2	105.4	0.8	30.5	86.5	0.4	9.1	104.5	2.4	39.0	149.4
1993–1997	0.0	14.8	75.8	1.0	28.8	90.2	0.2	8.0	96.3	0.2	13.1	67.3
1998–2002	0.8	26.4	97.6	1.0	36.5	80.3	0.2	10.9	93.9	1.6	24.8	117.9
2003–2007	1.0	31.0	100.1	1.6	41.5	96.7	0.2	12.1	93.0	1.6	23.1	123.8
2008–2012	1.2	34.1	104.6	0.8	33.5	84.8	0.2	13.5	94.4	0.6	17.0	95.6
	35–44			>74 y/o			35–44			>74 y/o		
1983–1987	1.8	33.5	105.2	1.4	75.4	96.3	1.4	15.4	130.7	2.0	67.9	117.7
1988–1992	0.4	17.7	74.8	0.8	50.3	78.0	0.2	8.7	89.8	1.0	36.6	76.4
1993–1997	1.2	25.6	104.4	2.0	65.5	125.8	0.4	8.6	101.4	1.4	34.6	100.6
1998–2002	1.0	32.3	91.7	2.2	62.5	118.5	0.0	8.4	81.7	1.0	24.5	78.8
2003–2007	1.8	45.6	113.8	1.6	43.9	97.4	0.2	11.6	92.2	1.8	27.2	121.4
2008–2012	1.6	39.8	107.0	1.6	39.6	95.5	0.4	13.5	99.8	1.0	17.3	90.0

Secondary Medical Zone ID: 185

	Male						Female					
	Suicide			Suicide			Suicide			Suicide		
	Num per year	Rate /100,000	×100	Num per year	Rate /100,000	×100	Num per year	Rate /100,000	×100	Num per year	Rate /100,000	×100
	Total (>10 y/o)			45–54			Total (>10 y/o)			45–54		
1983–1987	63.8	27.0	80.2	16.6	42.3	83.6	39.2	14.6	94.4	5.2	14.1	82.1
1988–1992	56.0	21.2	83.6	12.0	27.3	79.6	34.2	11.4	86.9	6.2	14.1	96.6
1993–1997	64.6	22.1	86.4	18.0	34.8	95.1	30.6	9.5	84.8	5.8	11.8	91.7
1998–2002	90.2	29.5	79.6	17.6	37.0	66.2	40.0	11.4	90.6	6.0	12.9	85.9
2003–2007	109.0	33.6	91.7	22.0	49.6	88.7	35.4	10.7	83.1	5.0	12.2	88.8
2008–2012	94.8	30.2	85.4	17.8	42.7	87.8	40.4	12.1	92.9	7.6	17.4	116.8
	15–24			55–64			15–24			55–64		
1983–1987	6.6	14.7	105.4	8.6	33.1	78.0	2.4	5.8	85.0	5.0	16.8	85.1
1988–1992	7.4	13.3	134.6	6.8	22.0	62.0	2.8	5.6	106.3	4.0	13.0	72.6
1993–1997	5.8	11.7	103.5	13.8	36.4	90.4	2.2	4.9	93.1	5.2	13.9	87.1
1998–2002	5.6	13.7	86.8	22.2	52.7	82.9	1.8	5.3	76.1	7.4	17.5	92.8
2003–2007	5.0	14.0	80.5	29.4	59.4	102.8	2.2	7.0	77.9	6.0	13.5	80.4
2008–2012	9.2	24.5	115.6	21.2	44.2	91.0	3.2	9.8	101.2	5.6	12.5	83.6
	25–34			65–74			25–34			65–74		
1983–1987	8.2	20.2	79.1	4.2	29.8	68.1	6.0	13.3	123.1	7.6	34.0	108.7
1988–1992	8.6	19.8	103.4	5.0	28.6	81.2	3.2	8.0	91.9	5.8	23.2	88.8
1993–1997	9.8	20.9	107.1	5.4	23.8	74.7	3.8	8.6	103.0	3.4	12.9	66.2
1998–2002	11.8	23.9	88.4	11.2	38.8	85.2	5.4	11.6	99.8	7.2	21.2	101.1
2003–2007	15.8	31.3	101.0	12.2	37.2	86.6	5.0	11.6	89.1	6.8	18.4	99.0
2008–2012	10.6	26.0	79.8	12.6	34.4	87.2	4.0	11.5	80.1	7.8	18.8	105.5
	35–44			>74 y/o			35–44			>74 y/o		
1983–1987	12.6	24.7	77.7	6.4	72.1	92.0	4.6	9.6	81.6	8.4	61.2	106.1
1988–1992	9.0	18.4	77.7	7.2	62.1	96.3	4.4	9.0	93.7	7.8	41.4	86.5
1993–1997	6.6	15.9	64.8	4.8	38.2	73.4	3.2	7.6	89.7	7.0	29.8	86.6
1998–2002	13.0	30.2	85.8	8.4	52.1	98.8	3.0	8.0	77.7	9.2	31.3	100.7
2003–2007	16.6	35.8	89.3	8.0	38.6	85.8	3.4	9.2	73.1	6.8	19.3	86.0
2008–2012	14.2	29.0	78.1	9.0	34.9	84.2	3.8	9.7	71.5	8.0	19.1	99.3

Secondary Medical Zone ID: 186

	Male						Female					
	Suicide			Suicide			Suicide			Suicide		
	Num per year	Rate /100,000	×100	Num per year	Rate /100,000	×100	Num per year	Rate /100,000	×100	Num per year	Rate /100,000	×100
	Total (>10 y/o)			45–54			Total (>10 y/o)			45–54		
1983–1987	75.2	27.6	82.1	17.8	39.2	77.6	44.8	14.4	93.8	6.4	14.8	85.9
1988–1992	62.6	20.6	81.1	15.4	29.4	85.6	41.6	11.6	90.7	5.8	12.2	83.4
1993–1997	66.0	19.9	76.5	19.2	32.3	88.4	39.4	10.0	92.3	7.8	13.1	101.7
1998–2002	113.0	31.5	85.0	26.4	46.3	82.7	54.6	12.4	103.2	9.0	15.3	101.5
2003–2007	114.0	31.3	82.4	21.0	42.3	75.6	48.4	11.7	93.5	6.6	13.1	95.4
2008–2012	118.4	31.2	89.6	17.6	36.4	74.9	37.0	10.1	73.8	6.0	12.8	85.9
	15–24			55–64			15–24			55–64		
1983–1987	7.0	14.1	101.3	10.0	32.3	76.1	4.6	8.5	123.8	6.6	18.4	93.0
1988–1992	4.8	8.9	89.4	11.2	28.1	79.2	1.6	3.5	66.5	7.0	17.0	95.2
1993–1997	5.0	9.5	84.3	12.0	28.0	69.7	1.2	3.0	57.7	6.6	14.7	92.6
1998–2002	7.0	14.8	93.8	25.2	50.1	78.8	3.2	6.8	96.9	7.2	15.2	81.0
2003–2007	7.0	16.5	94.5	28.4	49.6	86.0	4.8	10.9	120.9	11.0	18.3	109.5
2008–2012	9.4	22.6	106.7	23.2	42.2	86.8	1.6	5.6	57.6	5.2	10.5	70.5
	25–34			65–74			25–34			65–74		
1983–1987	11.2	24.2	94.5	7.0	39.4	90.0	5.2	10.9	101.4	6.4	26.0	83.3
1988–1992	5.8	12.8	67.0	5.4	27.3	77.4	3.2	7.2	82.9	9.8	31.9	122.2
1993–1997	6.6	13.2	67.7	8.2	28.9	90.5	3.2	6.7	80.4	5.4	16.1	82.6
1998–2002	15.6	26.0	96.3	13.4	38.5	84.6	4.6	8.9	76.7	11.6	27.7	131.8
2003–2007	16.6	28.4	91.7	12.6	32.7	76.1	4.6	9.4	72.0	6.8	16.3	87.3
2008–2012	13.6	27.4	84.1	18.2	39.7	100.4	5.2	11.8	82.6	7.4	16.0	89.6
	35–44			>74 y/o			35–44			>74 y/o		
1983–1987	15.8	26.7	83.8	5.8	58.7	75.0	5.8	10.3	86.9	9.8	61.0	105.7
1988–1992	9.6	17.4	73.6	10.0	74.4	115.3	3.8	7.4	76.4	10.4	47.0	98.2
1993–1997	9.4	19.0	77.7	5.6	39.7	76.3	4.8	9.1	107.0	10.4	37.5	108.9
1998–2002	18.8	36.4	103.3	6.2	36.7	69.7	4.2	8.9	85.7	14.6	42.0	135.2
2003–2007	20.6	36.6	91.3	7.8	33.2	73.8	4.8	9.9	78.1	9.8	23.4	104.3
2008–2012	23.8	37.0	99.3	12.2	39.5	95.3	5.8	10.7	78.8	5.6	12.4	64.4

Secondary Medical Zone ID: 187

	Male						Female					
	Suicide			Suicide			Suicide			Suicide		
	Num per year	Rate /100,000	×100	Num per year	Rate /100,000	×100	Num per year	Rate /100,000	×100	Num per year	Rate /100,000	×100
	Total (>10 y/o)			45-54			Total (>10 y/o)			45-54		
1983-1987	51.8	30.6	91.7	12.2	44.0	87.1	29.6	14.7	93.7	4.2	15.4	89.1
1988-1992	43.4	23.2	91.5	9.2	31.0	90.1	29.0	12.4	95.9	4.4	14.4	98.5
1993-1997	45.4	22.7	86.9	10.6	31.8	86.8	25.4	10.0	92.7	3.4	10.9	84.2
1998-2002	64.6	30.8	82.9	16.6	49.0	87.6	36.0	13.3	107.3	5.2	14.9	98.9
2003-2007	68.4	32.1	86.4	13.0	44.4	79.4	25.4	11.1	83.0	2.4	10.0	72.7
2008-2012	64.4	31.2	88.9	12.2	46.0	94.6	28.2	12.3	92.3	4.2	14.9	99.7
	15-24			55-64			15-24			55-64		
1983-1987	3.8	14.7	105.4	6.6	32.2	75.8	2.2	7.7	112.6	4.0	16.7	84.5
1988-1992	4.0	11.7	118.1	6.2	25.0	70.3	1.2	4.5	85.6	5.6	18.9	105.8
1993-1997	3.6	11.2	99.3	9.4	34.4	85.4	1.0	4.0	77.4	5.2	16.9	106.2
1998-2002	4.0	14.6	92.6	15.8	54.2	85.4	2.8	8.7	124.3	6.6	20.2	107.1
2003-2007	4.0	16.4	94.0	16.0	49.3	85.5	2.0	8.5	94.6	4.0	13.4	79.9
2008-2012	2.8	15.7	74.1	15.0	45.5	93.7	2.6	11.2	115.2	6.0	16.3	109.1
	25-34			65-74			25-34			65-74		
1983-1987	7.2	27.4	107.2	2.8	27.1	62.0	4.4	14.3	132.6	4.2	24.7	78.9
1988-1992	5.6	21.2	111.1	4.8	32.7	92.7	2.4	8.8	102.0	5.6	26.2	100.4
1993-1997	7.2	24.7	126.6	4.2	22.9	71.9	2.2	8.1	96.8	4.8	19.1	98.2
1998-2002	6.0	22.4	83.1	9.4	39.7	87.2	4.0	12.8	110.7	6.2	21.9	104.2
2003-2007	7.8	27.9	90.1	10.6	41.5	96.7	2.8	11.1	85.1	3.4	13.7	73.7
2008-2012	6.8	28.7	88.2	8.4	33.2	84.1	2.8	12.7	88.7	4.4	15.7	88.4
	35-44			>74 y/o			35-44			>74 y/o		
1983-1987	11.6	34.4	108.2	7.6	90.9	116.1	4.0	11.8	100.2	6.4	52.6	91.1
1988-1992	8.4	24.6	103.7	5.0	54.4	84.4	3.2	9.5	97.9	6.4	39.9	83.3
1993-1997	5.8	19.9	81.3	4.4	42.8	82.1	2.0	7.1	83.9	6.8	34.3	99.8
1998-2002	8.0	29.6	84.2	4.8	39.8	75.6	2.6	9.4	91.1	8.4	33.8	108.9
2003-2007	9.6	35.4	88.5	7.4	42.1	93.5	4.0	13.5	106.9	6.8	22.4	99.8
2008-2012	9.6	33.9	91.1	9.6	43.2	104.1	2.8	11.2	82.8	5.4	15.9	82.8

Secondary Medical Zone ID: 188

	Male						Female					
	Suicide			Suicide			Suicide			Suicide		
	Num per year	Rate /100,000	×100	Num per year	Rate /100,000	×100	Num per year	Rate /100,000	×100	Num per year	Rate /100,000	×100
	Total (>10 y/o)			45–54			Total (>10 y/o)			45–54		
1983–1987	59.4	29.9	88.7	13.2	40.4	79.9	33.6	13.6	86.9	4.4	13.6	78.9
1988–1992	45.4	21.9	85.5	9.2	29.0	84.4	36.6	12.8	101.9	6.8	17.6	120.0
1993–1997	48.0	21.9	85.4	10.4	30.8	84.2	33.8	10.8	106.7	5.2	13.7	106.0
1998–2002	79.2	34.6	95.5	18.8	53.6	95.7	34.6	11.4	95.3	3.8	12.1	80.5
2003–2007	78.2	34.8	94.4	18.0	57.4	102.6	32.8	11.9	96.0	5.6	16.0	116.7
2008–2012	74.6	35.7	99.1	14.6	50.6	104.2	32.2	12.9	97.5	3.4	12.6	84.3
	15–24			55–64			15–24			55–64		
1983–1987	3.6	12.9	92.1	8.0	31.4	73.8	1.8	6.2	90.3	4.8	15.9	80.8
1988–1992	2.4	8.8	88.8	8.4	27.2	76.7	1.8	5.5	104.6	3.6	12.3	68.9
1993–1997	3.2	11.1	97.9	10.8	34.6	86.1	2.0	6.0	115.8	5.6	15.8	99.6
1998–2002	3.0	13.2	83.4	20.0	63.0	99.2	1.2	5.7	81.3	6.8	19.2	102.1
2003–2007	3.4	15.9	91.3	16.6	49.8	86.3	1.6	7.8	86.5	6.2	16.9	100.9
2008–2012	6.8	29.0	136.9	15.2	45.1	92.8	2.4	11.2	115.0	4.6	13.6	90.9
	25–34			65–74			25–34			65–74		
1983–1987	9.8	31.2	122.1	5.8	37.8	86.4	3.2	10.3	95.3	5.6	25.8	82.4
1988–1992	4.8	17.7	92.8	3.8	24.2	68.6	2.2	7.9	90.6	7.4	28.0	107.4
1993–1997	5.2	18.9	96.7	6.8	28.9	90.6	1.8	7.0	83.5	6.2	20.2	103.4
1998–2002	8.4	28.5	105.6	11.4	40.9	89.9	3.0	10.6	91.3	6.2	19.1	91.1
2003–2007	8.0	29.2	94.1	9.2	33.9	79.1	2.6	10.6	81.8	7.2	20.7	111.4
2008–2012	8.0	33.8	103.7	9.6	35.3	89.3	2.6	12.5	87.3	4.6	15.2	85.3
	35–44			>74 y/o			35–44			>74 y/o		
1983–1987	11.6	32.0	100.6	7.2	70.8	90.4	4.4	11.8	99.7	9.4	58.4	101.3
1988–1992	8.6	24.2	102.0	8.0	67.0	103.8	3.0	8.8	91.2	11.8	57.6	120.3
1993–1997	5.2	18.0	73.4	6.4	50.0	96.0	1.6	6.2	72.4	11.4	46.7	135.8
1998–2002	10.2	34.9	99.2	7.4	48.9	92.8	2.0	7.9	76.2	11.6	39.1	126.0
2003–2007	11.2	38.8	96.8	11.8	55.0	122.2	2.4	9.7	76.9	7.2	20.3	90.6
2008–2012	12.2	40.1	107.7	8.2	33.3	80.4	5.2	15.6	115.3	9.2	21.6	112.4

Secondary Medical Zone ID: 189

	Male						Female					
	Suicide			Suicide			Suicide			Suicide		
	Num per year	Rate /100,000	×100	Num per year	Rate /100,000	×100	Num per year	Rate /100,000	×100	Num per year	Rate /100,000	×100
	Total (>10 y/o)			45–54			Total (>10 y/o)			45–54		
1983–1987	12.6	30.6	88.4	3.2	46.1	91.1	5.8	13.1	75.9	1.4	17.6	102.0
1988–1992	11.0	24.3	97.2	2.4	34.9	101.7	4.6	10.8	70.6	0.8	13.6	92.9
1993–1997	11.6	25.1	100.7	2.0	33.7	92.0	6.8	10.8	99.7	1.4	14.6	113.0
1998–2002	19.0	37.8	113.9	3.2	52.5	93.9	6.0	12.0	88.0	1.0	15.0	99.6
2003–2007	20.0	39.6	125.3	3.6	60.9	108.9	7.8	13.3	109.5	1.2	15.5	113.2
2008–2012	16.8	37.6	118.2	4.2	64.5	132.7	5.2	12.6	94.3	1.0	16.3	109.1
	15–24			55–64			15–24			55–64		
1983–1987	0.6	13.4	96.1	2.6	41.0	96.5	0.0	5.4	79.1	1.4	19.0	96.1
1988–1992	0.0	8.0	80.7	3.0	38.4	108.2	0.0	4.3	81.6	0.8	15.0	84.1
1993–1997	0.2	10.1	89.0	3.4	44.7	111.0	0.2	5.1	98.7	1.6	17.9	112.7
1998–2002	0.6	15.9	100.6	5.2	72.7	114.4	0.2	6.9	97.7	1.6	19.8	105.0
2003–2007	0.4	16.6	95.1	5.2	69.4	120.2	0.2	8.7	96.4	2.0	19.6	117.3
2008–2012	1.0	24.2	113.9	3.0	47.9	98.5	0.2	9.5	98.4	0.6	13.5	90.3
	25–34			65–74			25–34			65–74		
1983–1987	1.8	28.2	110.4	1.0	33.7	77.0	0.2	8.6	80.2	1.2	25.3	81.0
1988–1992	0.8	18.5	96.6	1.4	33.0	93.6	0.2	7.6	87.7	0.8	18.2	69.6
1993–1997	0.8	19.3	99.0	1.8	31.9	100.0	0.0	6.7	80.7	1.2	17.9	91.9
1998–2002	1.0	26.3	97.4	3.8	52.5	115.3	0.6	11.9	103.0	1.6	21.3	101.2
2003–2007	0.4	25.1	81.0	5.4	65.7	153.2	1.0	15.4	118.1	1.0	16.8	90.3
2008–2012	1.0	32.7	100.5	2.6	41.8	105.8	0.0	12.2	85.5	1.8	20.4	114.4
	35–44			>74 y/o			35–44			>74 y/o		
1983–1987	2.2	31.3	98.2	1.2	60.8	77.6	0.2	9.1	77.3	1.2	38.0	65.8
1988–1992	1.8	25.2	106.3	1.6	57.6	89.2	0.2	8.0	83.2	1.8	38.4	80.3
1993–1997	1.6	26.2	106.9	1.8	52.7	101.3	0.4	8.1	95.5	2.0	32.9	95.6
1998–2002	2.4	41.3	117.2	2.8	63.8	121.0	0.2	8.9	85.6	0.8	16.8	54.2
2003–2007	2.4	46.7	116.5	2.6	50.0	111.0	0.6	12.7	100.3	1.8	21.5	95.9
2008–2012	2.0	39.8	107.1	3.0	47.7	115.0	0.6	13.6	100.7	1.0	14.3	74.2

Secondary Medical Zone ID: 190

	Male						Female					
	Suicide			Suicide			Suicide			Suicide		
	Num per year	Rate /100,000	×100	Num per year	Rate /100,000	×100	Num per year	Rate /100,000	×100	Num per year	Rate /100,000	×100
	Total (>10 y/o)			45-54			Total (>10 y/o)			45-54		
1983-1987	25.6	28.9	84.1	5.2	37.3	73.8	17.4	15.9	105.8	3.4	19.6	113.9
1988-1992	25.0	23.2	91.9	6.4	34.2	99.5	16.4	12.6	100.2	2.8	14.8	101.6
1993-1997	26.8	22.0	85.7	7.0	32.6	89.1	14.2	10.4	91.6	2.6	12.1	93.6
1998-2002	39.4	30.5	80.4	8.8	42.2	75.5	19.6	12.2	97.5	3.4	14.7	97.6
2003-2007	49.2	33.6	92.0	10.2	50.3	90.0	18.8	12.1	92.0	2.6	12.8	93.4
2008-2012	49.0	33.6	94.6	9.0	45.3	93.1	23.4	13.8	104.7	3.4	15.5	103.6
	15-24			55-64			15-24			55-64		
1983-1987	2.0	12.4	88.8	3.2	33.9	79.8	2.2	9.4	137.4	1.8	17.2	87.2
1988-1992	3.2	12.7	127.9	4.0	30.1	84.8	1.0	5.0	94.8	3.4	20.6	115.1
1993-1997	3.4	13.5	119.3	7.6	44.9	111.6	0.8	4.4	84.3	3.6	19.2	120.9
1998-2002	3.2	15.7	99.4	9.2	52.4	82.4	1.2	6.4	91.8	2.2	14.9	79.1
2003-2007	3.0	16.4	94.1	14.0	61.6	106.7	2.2	10.5	116.6	4.0	17.0	101.3
2008-2012	3.4	20.1	94.9	10.0	44.9	92.4	2.8	13.3	136.9	3.6	14.8	99.4
	25-34			65-74			25-34			65-74		
1983-1987	3.0	20.9	81.6	3.6	54.3	123.9	1.2	8.9	82.5	3.0	34.4	110.2
1988-1992	2.8	16.9	88.7	2.8	37.3	106.0	2.0	9.8	113.3	1.8	20.9	80.1
1993-1997	2.8	16.4	83.8	3.0	30.0	94.0	2.8	11.1	133.9	1.6	15.3	78.6
1998-2002	4.4	23.0	85.1	5.2	41.5	91.3	2.8	12.1	104.6	3.4	22.4	106.5
2003-2007	6.0	28.8	92.9	5.6	39.1	91.0	2.8	12.7	97.9	2.2	15.5	83.4
2008-2012	6.6	34.0	104.3	6.6	38.4	97.3	2.8	14.2	98.9	3.8	19.1	107.1
	35-44			>74 y/o			35-44			>74 y/o		
1983-1987	6.8	32.0	100.5	1.6	59.5	75.9	1.8	9.9	84.1	4.0	73.0	126.6
1988-1992	3.2	17.1	72.3	2.6	61.5	95.3	2.2	9.7	100.7	3.2	44.3	92.6
1993-1997	1.2	10.9	44.5	1.8	40.7	78.1	1.4	7.6	88.8	1.2	17.2	50.1
1998-2002	4.8	26.8	76.0	3.8	56.2	106.7	2.2	10.5	101.1	4.4	35.4	113.9
2003-2007	5.8	29.6	73.8	4.6	47.9	106.3	2.2	11.1	87.7	2.8	18.8	83.7
2008-2012	9.2	37.7	101.4	4.0	35.0	84.3	4.4	15.7	115.6	2.6	14.9	77.4

Secondary Medical Zone ID: 191

	Male						Female					
	Suicide			Suicide			Suicide			Suicide		
	Num per year	Rate /100,000	×100	Num per year	Rate /100,000	×100	Num per year	Rate /100,000	×100	Num per year	Rate /100,000	×100
	Total (>10 y/o)			45-54			Total (>10 y/o)			45-54		
1983-1987	21.8	29.0	81.5	3.8	33.3	65.8	12.0	14.6	92.1	1.4	14.4	83.8
1988-1992	20.4	22.5	87.2	4.2	26.8	78.0	12.6	12.5	96.4	2.8	16.0	109.5
1993-1997	21.6	21.7	79.4	5.6	29.9	81.7	11.8	10.3	93.6	2.6	13.0	100.3
1998-2002	35.6	30.5	79.4	7.4	40.9	73.0	16.2	12.2	96.5	2.2	12.8	84.8
2003-2007	40.4	31.8	81.8	10.2	57.0	101.9	16.4	12.0	93.2	3.4	16.2	118.4
2008-2012	39.4	29.9	81.9	7.0	42.2	86.8	15.4	11.5	86.0	2.0	13.2	88.5
	15-24			55-64			15-24			55-64		
1983-1987	1.6	11.8	84.7	2.8	36.8	86.6	0.6	5.7	83.1	1.6	18.6	94.3
1988-1992	1.4	8.6	87.1	3.6	33.0	92.8	0.6	4.3	81.0	1.4	15.5	87.0
1993-1997	1.2	8.1	71.8	4.8	36.8	91.3	0.6	4.1	78.4	2.2	16.6	104.3
1998-2002	2.4	12.3	78.1	8.4	52.8	83.1	2.2	9.0	128.3	2.4	17.0	90.6
2003-2007	3.2	15.5	89.0	7.0	39.9	69.1	1.2	7.9	87.9	1.8	12.8	76.6
2008-2012	3.4	18.3	86.1	7.2	40.1	82.5	1.0	7.8	80.9	3.0	14.6	97.7
	25-34			65-74			25-34			65-74		
1983-1987	3.4	22.5	87.9	2.0	44.1	100.7	0.6	7.1	65.6	2.8	38.1	122.0
1988-1992	2.8	17.7	92.9	2.2	37.8	107.2	0.6	6.4	73.6	2.0	26.4	101.3
1993-1997	2.8	16.3	83.4	2.2	30.0	94.0	1.0	7.0	83.9	2.4	23.4	120.3
1998-2002	4.2	20.6	76.4	5.2	49.3	108.4	2.8	12.0	103.2	2.8	23.8	113.3
2003-2007	5.4	24.2	78.2	5.0	41.4	96.4	2.0	10.3	79.0	2.6	19.8	106.4
2008-2012	4.8	25.8	79.1	3.0	26.5	67.1	2.4	12.6	87.6	2.6	17.5	98.4
	35-44			>74 y/o			35-44			>74 y/o		
1983-1987	5.6	27.9	87.7	2.6	95.2	121.6	2.4	11.8	100.3	2.6	64.0	110.9
1988-1992	4.6	22.9	96.7	1.2	49.2	76.3	2.0	9.7	100.7	3.2	55.8	116.7
1993-1997	3.8	22.3	91.1	1.2	40.2	77.2	0.8	6.5	76.3	2.2	32.4	94.3
1998-2002	5.4	31.4	89.2	2.6	54.2	102.8	0.6	6.8	66.2	3.2	35.0	112.6
2003-2007	7.0	34.3	85.6	2.6	40.8	90.6	2.2	11.7	92.6	3.2	26.6	118.4
2008-2012	8.4	33.8	91.0	5.6	54.8	132.1	1.8	10.0	74.0	2.4	18.0	93.7

Secondary Medical Zone ID: 192

	Male						Female					
	Suicide			Suicide			Suicide			Suicide		
	Num per year	Rate /100,000	×100	Num per year	Rate /100,000	×100	Num per year	Rate /100,000	×100	Num per year	Rate /100,000	×100
	Total (>10 y/o)			45-54			Total (>10 y/o)			45-54		
1983-1987	18.0	35.0	114.3	4.4	56.2	111.2	9.8	16.7	114.8	1.2	17.2	100.0
1988-1992	13.4	24.4	101.5	2.0	28.2	82.1	8.0	13.0	102.4	1.2	14.8	101.1
1993-1997	14.8	24.5	97.1	4.2	38.0	104.0	9.2	11.5	116.5	1.8	14.8	114.8
1998-2002	21.8	34.3	93.2	5.8	53.1	94.9	8.6	12.1	96.3	0.8	12.5	82.9
2003-2007	21.4	32.8	87.5	3.4	40.0	71.5	9.8	13.0	104.5	0.8	11.8	86.1
2008-2012	26.8	35.7	111.2	4.8	51.3	105.5	11.2	13.8	112.1	0.8	12.7	85.4
	15-24			55-64			15-24			55-64		
1983-1987	0.8	12.8	91.6	3.8	54.3	127.9	0.4	6.6	96.3	1.8	22.9	116.2
1988-1992	1.0	10.2	103.3	2.6	36.5	102.8	0.6	5.8	110.2	0.6	14.7	82.3
1993-1997	0.8	10.1	89.7	3.8	45.4	112.8	0.4	4.9	94.3	1.2	16.0	100.7
1998-2002	1.6	16.3	103.1	4.8	59.4	93.4	0.2	5.6	79.7	1.8	19.9	105.7
2003-2007	2.2	20.2	116.1	4.8	50.6	87.7	1.6	12.6	139.8	1.8	17.4	103.6
2008-2012	1.6	20.7	97.5	6.2	53.0	109.1	1.2	11.9	122.6	2.0	16.4	109.9
	25-34			65-74			25-34			65-74		
1983-1987	1.6	22.6	88.2	1.4	43.8	100.2	0.2	7.9	73.0	2.2	39.7	127.1
1988-1992	1.2	16.8	88.1	1.6	38.5	109.3	0.8	9.1	104.4	1.4	26.6	102.0
1993-1997	1.0	15.4	78.9	1.6	31.7	99.3	0.6	7.9	94.8	2.6	30.1	154.3
1998-2002	2.8	26.9	99.6	2.2	40.8	89.8	0.6	9.9	85.7	1.2	19.5	92.9
2003-2007	2.4	26.5	85.6	3.2	45.8	106.7	1.4	13.6	104.9	1.8	21.1	113.5
2008-2012	3.0	32.7	100.3	4.2	48.0	121.4	1.0	13.6	95.1	1.8	19.8	111.0
	35-44			>74 y/o			35-44			>74 y/o		
1983-1987	4.0	36.6	115.0	2.0	93.8	119.7	0.6	10.1	85.4	3.4	94.5	163.8
1988-1992	2.6	24.0	101.1	2.2	82.3	127.6	0.6	8.5	88.1	2.8	64.2	134.1
1993-1997	2.2	22.8	92.9	1.2	49.5	95.1	0.4	6.9	81.4	2.2	41.7	121.1
1998-2002	3.2	34.4	97.6	1.4	48.7	92.3	1.2	11.3	108.9	2.8	40.7	131.2
2003-2007	2.8	33.2	82.8	2.6	53.2	118.0	0.8	11.4	90.4	1.6	21.5	96.0
2008-2012	4.0	38.1	102.5	3.0	49.0	118.1	2.0	16.1	118.6	2.4	23.5	122.4

Secondary Medical Zone ID: 193

	Male						Female					
	Suicide			Suicide			Suicide			Suicide		
	Num per year	Rate /100,000	×100	Num per year	Rate /100,000	×100	Num per year	Rate /100,000	×100	Num per year	Rate /100,000	×100
	Total (>10 y/o)			45-54			Total (>10 y/o)			45-54		
1983-1987	23.6	30.2	88.1	5.4	44.7	88.3	14.8	15.2	98.5	2.2	17.3	100.5
1988-1992	22.0	23.9	97.2	3.4	27.4	79.8	12.6	12.1	91.2	2.6	16.5	113.1
1993-1997	27.8	26.2	109.0	6.8	39.1	106.8	14.0	10.8	106.9	2.4	13.9	108.0
1998-2002	30.6	31.7	82.5	6.4	42.0	75.0	13.6	11.7	91.0	2.2	14.2	94.2
2003-2007	40.0	36.6	101.3	9.0	58.3	104.3	16.4	12.4	106.4	2.6	15.3	111.4
2008-2012	41.2	36.4	109.6	7.0	50.6	104.1	16.0	12.6	104.5	2.6	16.8	112.3
	15-24			55-64			15-24			55-64		
1983-1987	1.4	12.1	86.6	3.0	33.8	79.5	0.4	5.3	78.3	2.4	19.7	100.0
1988-1992	1.6	10.1	102.3	5.6	42.2	119.0	1.2	6.4	121.9	2.4	18.4	102.8
1993-1997	2.6	13.4	118.6	6.4	47.2	117.2	1.2	6.2	118.9	1.6	14.2	89.3
1998-2002	2.0	14.5	91.6	8.0	60.1	94.7	1.0	7.0	99.1	1.4	15.1	80.3
2003-2007	3.2	20.3	116.2	7.0	47.3	81.9	0.2	5.5	61.6	1.6	13.9	83.0
2008-2012	2.8	21.9	103.2	7.4	45.5	93.6	1.0	9.3	95.8	1.8	13.1	87.9
	25-34			65-74			25-34			65-74		
1983-1987	4.8	30.8	120.6	2.8	44.1	100.8	1.2	9.6	89.2	3.0	32.3	103.4
1988-1992	3.6	23.7	124.1	3.2	41.0	116.4	0.8	7.5	86.5	2.2	22.9	87.9
1993-1997	4.4	25.9	132.4	2.2	26.8	84.1	0.8	7.3	87.4	2.6	21.4	109.7
1998-2002	3.2	22.9	84.9	3.0	34.8	76.5	1.0	9.5	82.3	2.8	22.2	105.7
2003-2007	4.6	29.0	93.6	7.4	58.8	137.1	1.4	11.3	86.6	5.4	31.8	170.9
2008-2012	4.4	30.9	94.9	7.0	51.0	129.0	1.2	11.9	82.9	2.0	17.0	95.3
	35-44			>74 y/o			35-44			>74 y/o		
1983-1987	3.4	23.8	74.9	2.8	77.7	99.2	0.8	8.7	73.7	4.8	78.1	135.3
1988-1992	3.0	19.4	81.9	1.2	40.1	62.1	0.8	7.6	78.3	2.6	36.8	76.8
1993-1997	2.8	20.1	82.1	2.2	47.3	90.7	0.6	6.4	74.7	4.8	47.6	138.3
1998-2002	4.6	33.0	93.8	3.4	55.5	105.3	1.0	8.8	85.5	4.2	34.8	112.0
2003-2007	5.8	39.9	99.5	3.0	40.6	90.1	1.2	10.9	86.2	4.0	26.9	119.8
2008-2012	8.6	47.3	127.2	4.0	41.9	101.1	1.4	11.9	87.5	6.0	31.9	165.7

Secondary Medical Zone ID: 194

	Male						Female					
	Suicide			Suicide			Suicide			Suicide		
	Num per year	Rate /100,000	×100	Num per year	Rate /100,000	×100	Num per year	Rate /100,000	×100	Num per year	Rate /100,000	×100
	Total (>10 y/o)			45–54			Total (>10 y/o)			45–54		
1983–1987	14.0	29.3	83.2	3.8	47.6	94.2	9.4	14.9	96.3	1.6	17.8	103.6
1988–1992	11.4	22.2	82.9	2.0	27.4	79.8	9.0	12.4	98.1	0.8	12.9	87.9
1993–1997	12.8	22.8	84.3	2.0	27.3	74.7	8.0	10.6	97.6	0.8	11.2	87.0
1998–2002	19.4	32.4	83.3	5.0	50.1	89.5	7.0	11.4	80.5	1.4	14.6	96.7
2003–2007	23.4	35.7	93.4	4.8	51.8	92.7	8.4	11.8	91.1	1.2	13.3	97.1
2008–2012	20.0	32.8	87.3	4.8	51.6	106.2	9.2	12.8	96.1	1.0	13.5	90.6
	15–24			55–64			15–24			55–64		
1983–1987	0.4	9.4	67.2	2.4	38.8	91.2	0.6	6.6	96.9	1.4	18.8	95.3
1988–1992	1.4	11.1	111.8	1.6	27.1	76.2	0.2	4.2	78.9	1.8	19.3	108.0
1993–1997	1.6	12.5	110.6	3.2	40.2	99.8	1.2	7.2	138.8	1.2	15.2	95.9
1998–2002	1.6	15.6	98.9	4.0	53.1	83.5	0.6	6.7	96.1	1.4	17.8	94.6
2003–2007	1.6	17.1	98.0	4.8	51.5	89.3	0.8	9.0	99.7	1.6	16.6	98.9
2008–2012	2.2	23.1	109.0	3.2	39.3	80.8	1.0	10.7	109.8	2.2	17.0	114.2
	25–34			65–74			25–34			65–74		
1983–1987	2.8	28.5	111.7	1.0	34.3	78.4	1.2	11.7	108.3	1.8	30.7	98.3
1988–1992	1.0	15.3	79.8	0.6	24.7	70.1	0.6	8.0	92.3	1.8	26.6	101.8
1993–1997	1.6	18.5	94.5	1.4	27.7	86.9	0.4	7.0	84.5	1.0	16.3	83.8
1998–2002	1.4	20.5	75.8	3.0	44.9	98.7	1.2	11.7	100.7	1.0	17.2	81.8
2003–2007	3.0	29.4	94.8	2.4	38.2	89.1	0.6	10.4	79.6	1.8	20.2	108.3
2008–2012	3.8	36.1	110.7	1.8	32.1	81.2	1.4	14.8	103.0	2.0	19.9	112.1
	35–44			>74 y/o			35–44			>74 y/o		
1983–1987	2.2	25.6	80.4	1.2	64.1	81.9	0.6	9.8	82.6	2.2	58.6	101.6
1988–1992	2.6	24.2	102.0	2.2	72.4	112.2	1.6	11.5	119.5	2.2	45.3	94.7
1993–1997	2.2	23.6	96.1	0.8	36.8	70.7	0.6	7.7	90.5	2.8	43.4	126.2
1998–2002	3.0	33.7	95.6	1.4	43.9	83.3	0.4	8.2	79.3	1.0	18.9	60.9
2003–2007	5.0	47.8	119.4	1.8	39.7	88.1	0.6	10.5	83.0	1.8	20.9	93.2
2008–2012	2.6	30.8	82.8	1.6	32.7	78.9	0.8	11.6	85.7	0.8	12.3	64.0

Secondary Medical Zone ID: 195

	Male						Female					
	Suicide			Suicide			Suicide			Suicide		
	Num per year	Rate /100,000	×100	Num per year	Rate /100,000	×100	Num per year	Rate /100,000	×100	Num per year	Rate /100,000	×100
	Total (>10 y/o)			45-54			Total (>10 y/o)			45-54		
1983-1987	18.2	30.0	86.9	3.8	40.7	80.5	13.4	15.8	106.2	1.8	17.3	100.6
1988-1992	17.6	24.8	101.9	3.4	33.3	97.0	8.4	11.3	80.7	1.4	14.2	97.0
1993-1997	15.6	23.3	87.6	3.2	31.8	86.9	8.6	10.0	90.1	1.4	12.8	98.9
1998-2002	28.4	36.3	103.8	5.8	52.2	93.3	8.8	11.6	83.4	2.2	16.8	111.7
2003-2007	25.0	33.9	92.2	4.4	46.5	83.1	9.0	11.6	88.5	0.6	11.0	80.0
2008-2012	24.4	34.1	96.7	4.2	46.3	95.3	10.6	12.5	99.5	0.6	11.7	78.3
	15-24			55-64			15-24			55-64		
1983-1987	1.6	15.2	109.1	3.4	39.7	93.5	0.4	5.8	85.4	1.6	17.9	90.5
1988-1992	1.2	10.5	106.5	3.6	35.3	99.5	0.4	4.8	90.1	1.6	16.9	94.6
1993-1997	1.6	12.8	113.0	3.4	36.8	91.3	0.2	4.1	79.4	1.8	16.8	105.6
1998-2002	1.8	17.1	108.3	7.0	67.9	106.9	0.4	6.2	88.6	1.8	18.3	97.1
2003-2007	1.8	18.7	107.4	7.0	62.0	107.4	0.6	8.3	92.7	1.4	15.3	91.3
2008-2012	1.8	21.9	103.1	5.6	49.6	102.0	0.6	9.1	93.5	1.4	14.2	95.4
	25-34			65-74			25-34			65-74		
1983-1987	2.8	26.8	104.9	2.0	40.1	91.5	1.0	10.5	97.3	3.4	37.8	121.0
1988-1992	1.6	18.3	95.7	1.8	32.2	91.4	0.0	5.7	65.5	1.8	22.7	87.0
1993-1997	1.6	18.3	93.6	1.0	20.7	64.8	0.6	7.6	91.3	1.0	14.4	74.1
1998-2002	2.8	26.8	99.2	4.0	45.7	100.5	0.8	10.3	88.5	1.6	18.8	89.5
2003-2007	3.6	32.5	104.9	3.2	39.4	91.9	1.2	12.5	96.5	2.2	20.6	110.5
2008-2012	5.0	41.7	127.9	2.8	35.8	90.7	1.0	13.3	92.7	1.8	18.0	101.3
	35-44			>74 y/o			35-44			>74 y/o		
1983-1987	2.8	27.2	85.4	1.8	65.2	83.2	2.0	14.0	118.2	3.2	62.3	108.1
1988-1992	2.2	21.2	89.4	3.6	84.4	130.8	1.2	10.1	104.2	2.0	33.9	70.9
1993-1997	2.4	23.7	96.9	2.0	48.7	93.5	0.4	6.8	80.3	3.2	37.8	109.8
1998-2002	3.6	36.4	103.3	3.4	61.2	116.2	0.6	8.8	85.0	1.4	18.2	58.7
2003-2007	2.2	28.4	70.8	2.6	41.5	92.2	0.4	9.6	76.2	2.4	21.3	95.0
2008-2012	2.6	31.0	83.3	2.4	34.7	83.7	1.6	14.2	104.8	3.4	24.0	125.0

Secondary Medical Zone ID: 196

	Male						Female					
	Suicide			Suicide			Suicide			Suicide		
	Num per year	Rate /100,000	×100	Num per year	Rate /100,000	×100	Num per year	Rate /100,000	×100	Num per year	Rate /100,000	×100
	Total (>10 y/o)			45–54			Total (>10 y/o)			45–54		
1983–1987	8.2	34.0	110.2	1.8	52.1	103.0	4.0	15.4	99.7	0.6	17.4	100.8
1988–1992	7.6	26.4	119.8	2.0	43.7	127.2	4.0	13.0	103.9	0.4	14.3	97.9
1993–1997	4.2	23.2	80.6	1.6	38.6	105.4	2.4	10.1	87.3	0.0	10.9	84.4
1998–2002	10.0	36.3	103.5	2.8	61.1	109.2	3.2	12.4	92.0	0.4	14.3	95.3
2003–2007	11.6	37.5	116.0	1.0	42.7	76.4	4.2	12.7	105.0	1.0	16.0	117.1
2008–2012	7.8	33.3	94.3	0.8	41.1	84.6	3.6	12.9	99.8	0.2	13.4	90.1
	15–24			55–64			15–24			55–64		
1983–1987	1.2	20.2	144.7	1.6	45.5	107.2	0.0	6.0	87.7	0.2	16.5	83.6
1988–1992	1.0	12.7	128.5	1.6	39.2	110.4	0.0	4.6	87.8	0.6	17.6	98.6
1993–1997	0.0	9.7	85.4	1.0	36.9	91.6	0.0	4.6	87.7	0.2	13.7	85.9
1998–2002	1.0	18.5	117.1	2.4	65.4	103.0	0.2	7.0	99.4	0.2	16.6	88.0
2003–2007	0.6	18.0	103.4	3.2	67.6	117.0	0.2	8.8	97.5	0.6	16.6	98.9
2008–2012	0.2	19.1	90.0	2.6	52.8	108.6	0.0	8.3	85.3	0.2	13.4	89.6
	25–34			65–74			25–34			65–74		
1983–1987	0.8	25.3	99.0	1.0	46.9	107.1	0.4	11.1	102.7	1.0	34.1	109.1
1988–1992	0.4	17.6	91.9	1.6	47.0	133.5	0.4	9.4	107.9	0.8	26.7	102.4
1993–1997	0.4	18.1	92.7	0.4	25.6	80.1	0.4	9.0	107.8	1.0	22.9	117.3
1998–2002	1.4	30.8	113.9	1.0	40.1	88.1	0.2	10.9	94.4	0.6	19.8	94.3
2003–2007	1.8	38.1	122.8	1.4	42.8	99.7	0.0	11.2	86.0	1.0	20.9	112.1
2008–2012	0.6	30.7	94.2	1.0	37.2	94.1	0.4	14.5	101.4	1.0	19.7	111.0
	35–44			>74 y/o			35–44			>74 y/o		
1983–1987	0.2	23.5	74.0	1.6	101.1	129.0	0.4	11.8	99.7	1.4	69.0	119.6
1988–1992	0.6	21.4	90.1	0.4	51.1	79.2	0.2	9.1	94.2	1.6	60.4	126.2
1993–1997	0.6	22.0	89.6	0.2	37.1	71.3	0.0	7.1	83.4	0.8	31.8	92.3
1998–2002	0.6	29.6	84.0	0.8	49.2	93.3	0.8	12.6	121.7	0.8	27.2	87.7
2003–2007	1.4	42.3	105.6	2.2	62.1	137.9	0.4	12.6	100.0	1.0	23.3	104.0
2008–2012	1.2	37.7	101.5	1.4	43.1	104.0	1.0	16.1	118.4	0.8	18.3	95.1

Secondary Medical Zone ID: 197

	Male						Female					
	Suicide			Suicide			Suicide			Suicide		
	Num per year	Rate /100,000	×100	Num per year	Rate /100,000	×100	Num per year	Rate /100,000	×100	Num per year	Rate /100,000	×100
	Total (>10 y/o)			45-54			Total (>10 y/o)			45-54		
1983-1987	22.8	36.5	120.5	5.6	59.2	117.1	12.2	15.9	106.1	2.2	19.8	114.7
1988-1992	18.2	28.1	122.0	3.0	35.8	104.2	11.6	13.4	111.3	1.2	14.3	98.1
1993-1997	22.6	30.5	143.0	5.0	46.7	127.6	8.6	10.8	101.6	1.4	13.8	106.8
1998-2002	28.2	41.1	130.5	6.2	65.3	116.7	9.0	12.1	97.9	0.8	13.4	89.1
2003-2007	25.0	41.0	123.2	4.8	61.7	110.3	10.4	12.9	115.4	0.8	13.0	95.2
2008-2012	21.8	37.1	119.2	4.2	55.7	114.6	7.8	12.7	101.5	1.0	15.1	101.3
	15-24			55-64			15-24			55-64		
1983-1987	0.6	12.3	88.0	4.2	48.4	114.0	1.0	8.9	129.8	1.2	16.6	84.0
1988-1992	1.2	12.1	122.1	3.4	37.4	105.3	0.4	5.5	104.6	1.6	17.5	97.9
1993-1997	1.6	15.1	133.2	4.8	50.1	124.5	0.6	6.3	121.3	2.2	19.5	122.8
1998-2002	2.0	21.8	138.1	7.8	86.1	135.5	0.2	6.5	92.6	1.8	19.6	104.0
2003-2007	1.0	18.8	107.9	8.4	84.0	145.5	0.2	8.2	90.7	2.2	19.4	115.6
2008-2012	0.6	20.1	94.8	5.0	54.1	111.4	0.2	8.9	91.6	1.0	14.1	94.5
	25-34			65-74			25-34			65-74		
1983-1987	2.2	27.9	109.1	2.4	45.9	104.8	0.8	11.0	101.9	2.4	31.9	102.1
1988-1992	3.2	32.6	170.4	1.6	32.1	91.1	0.8	9.7	111.8	2.6	29.8	114.4
1993-1997	2.6	28.2	144.1	2.6	35.5	111.3	0.0	6.4	76.7	1.0	15.1	77.7
1998-2002	2.0	29.1	107.9	6.0	63.5	139.7	0.6	11.3	97.3	1.4	18.6	88.4
2003-2007	1.8	31.7	102.2	2.8	41.1	95.7	0.0	10.0	77.1	2.0	20.8	111.5
2008-2012	1.8	34.9	107.1	3.6	46.1	116.8	0.8	15.4	107.5	0.6	14.4	80.6
	35-44			>74 y/o			35-44			>74 y/o		
1983-1987	5.4	46.8	147.2	2.4	73.8	94.2	1.2	12.2	103.4	3.4	61.3	106.2
1988-1992	2.2	24.4	103.0	3.6	82.7	128.2	1.0	10.2	106.0	4.0	56.7	118.5
1993-1997	2.2	26.8	109.2	3.8	76.7	147.3	0.6	8.5	99.4	2.8	35.3	102.7
1998-2002	1.8	30.8	87.3	2.2	48.9	92.9	0.4	9.2	88.6	3.8	38.4	123.7
2003-2007	3.4	47.8	119.3	2.8	46.4	103.1	1.0	13.5	106.9	4.0	33.2	148.0
2008-2012	2.6	39.2	105.3	3.8	49.4	119.1	0.6	12.8	94.2	3.6	26.4	137.5

Secondary Medical Zone ID: 198

	Male						Female					
	Suicide			Suicide			Suicide			Suicide		
	Num per year	Rate /100,000	×100	Num per year	Rate /100,000	×100	Num per year	Rate /100,000	×100	Num per year	Rate /100,000	×100
	Total (>10 y/o)			45–54			Total (>10 y/o)			45–54		
1983–1987	33.8	35.4	110.3	7.4	51.0	100.9	19.4	15.9	107.5	3.8	21.3	123.5
1988–1992	26.8	26.1	108.5	4.8	33.8	98.3	18.8	13.3	113.0	2.2	14.7	100.7
1993–1997	26.6	25.3	103.1	5.8	36.4	99.4	18.8	12.4	126.9	4.0	18.4	142.6
1998–2002	40.0	37.0	106.4	9.6	59.7	106.7	20.8	14.4	122.8	3.6	18.8	125.0
2003–2007	32.6	33.3	89.8	5.6	45.5	81.3	16.0	12.9	106.9	1.2	12.0	87.4
2008–2012	32.6	34.6	98.1	5.6	47.6	98.0	13.0	12.9	96.2	2.4	17.5	117.6
	15–24			55–64			15–24			55–64		
1983–1987	2.0	16.1	115.2	4.6	37.5	88.2	0.6	6.3	91.6	2.6	18.0	91.5
1988–1992	2.2	12.4	124.7	6.4	41.8	117.8	0.2	3.9	74.2	4.0	21.9	122.9
1993–1997	1.4	11.0	96.9	6.4	44.6	110.7	0.2	4.0	76.1	3.8	21.3	133.8
1998–2002	1.0	12.6	79.7	11.0	75.7	119.1	0.4	6.1	86.6	4.2	23.5	124.9
2003–2007	2.4	19.7	112.8	8.2	55.2	95.7	0.8	9.2	102.4	3.8	20.0	119.7
2008–2012	2.2	22.1	104.1	6.0	43.7	89.9	0.8	10.1	103.9	2.8	16.4	109.9
	25–34			65–74			25–34			65–74		
1983–1987	4.4	31.8	124.5	3.0	39.0	89.0	1.2	10.2	95.1	4.0	34.0	108.8
1988–1992	2.6	20.9	109.6	2.8	32.3	91.7	0.6	7.3	84.4	3.4	26.5	101.7
1993–1997	2.0	17.9	91.4	4.0	34.5	108.1	1.0	8.4	101.3	4.2	26.2	134.3
1998–2002	2.2	20.9	77.5	4.8	40.9	89.9	2.0	13.3	114.6	4.4	26.5	126.2
2003–2007	3.0	25.6	82.7	4.8	41.0	95.6	1.8	13.8	106.1	3.8	23.4	125.5
2008–2012	4.4	34.8	106.9	4.4	38.1	96.4	1.0	12.9	89.7	0.8	12.3	69.1
	35–44			>74 y/o			35–44			>74 y/o		
1983–1987	8.0	43.5	136.8	4.4	84.5	107.9	1.4	10.2	86.4	5.8	72.9	126.3
1988–1992	4.2	25.9	109.2	3.8	62.8	97.4	0.6	7.3	75.2	7.8	74.0	154.6
1993–1997	3.4	25.1	102.3	3.6	52.4	100.6	1.0	8.2	96.0	4.6	38.0	110.3
1998–2002	5.2	39.6	112.3	6.2	71.3	135.3	1.2	10.3	99.2	5.0	34.3	110.5
2003–2007	4.0	34.4	85.8	4.6	46.2	102.6	1.2	11.7	92.8	3.4	20.5	91.2
2008–2012	5.8	40.0	107.4	4.0	37.2	89.6	1.8	13.9	102.5	3.4	18.2	94.9

Secondary Medical Zone ID: 199

	Male						Female					
	Suicide			Suicide			Suicide			Suicide		
	Num per year	Rate /100,000	×100	Num per year	Rate /100,000	×100	Num per year	Rate /100,000	×100	Num per year	Rate /100,000	×100
	Total (>10 y/o)			45–54			Total (>10 y/o)			45–54		
1983–1987	16.8	32.0	96.7	2.2	35.3	69.8	9.4	14.9	97.0	1.8	18.8	109.2
1988–1992	15.0	24.7	101.5	3.0	33.3	96.9	9.6	12.7	101.9	1.0	13.4	91.8
1993–1997	17.0	25.5	102.5	5.0	41.3	112.9	8.0	10.3	95.1	1.0	11.5	89.3
1998–2002	26.2	36.7	104.8	6.2	55.3	98.8	9.2	11.9	92.7	1.8	15.3	101.9
2003–2007	26.0	37.5	103.6	5.8	59.0	105.5	8.4	12.1	90.3	1.2	13.1	95.8
2008–2012	20.8	33.7	92.7	4.2	49.9	102.6	9.2	12.6	97.1	1.4	15.2	102.2
	15–24			55–64			15–24			55–64		
1983–1987	1.0	13.3	95.4	2.4	39.0	91.7	0.2	5.7	83.1	2.0	22.2	112.6
1988–1992	1.2	10.4	105.5	3.0	36.4	102.4	0.6	5.6	106.9	1.6	18.3	102.5
1993–1997	1.0	10.2	90.3	2.4	33.9	84.3	0.8	6.0	115.8	1.4	16.0	100.7
1998–2002	1.2	13.6	86.1	5.8	63.8	100.4	0.6	6.7	95.5	1.8	19.0	100.9
2003–2007	1.4	15.9	91.3	6.6	60.5	104.9	1.2	10.7	119.0	0.8	13.4	79.8
2008–2012	1.8	21.1	99.5	4.2	42.7	88.0	0.6	9.1	94.0	1.4	14.0	93.7
	25–34			65–74			25–34			65–74		
1983–1987	3.0	31.7	124.1	1.8	44.0	100.6	0.8	10.4	96.4	1.6	29.3	93.6
1988–1992	1.4	19.1	100.0	1.8	36.9	104.6	0.4	7.6	87.3	2.0	28.4	108.8
1993–1997	1.0	16.7	85.6	0.8	21.7	68.0	0.6	8.0	95.6	1.2	17.6	90.2
1998–2002	2.6	28.5	105.6	4.4	53.2	116.9	1.0	11.5	99.5	0.8	15.7	74.8
2003–2007	3.4	35.5	114.4	3.8	46.7	108.8	1.2	13.4	103.1	0.4	12.9	69.1
2008–2012	2.8	34.6	106.1	2.4	34.3	86.7	0.4	11.7	81.6	1.6	17.9	100.4
	35–44			>74 y/o			35–44			>74 y/o		
1983–1987	3.8	34.2	107.5	2.6	88.5	113.0	1.2	11.6	98.6	1.8	48.1	83.4
1988–1992	2.6	23.8	100.4	2.0	64.6	100.1	1.2	10.1	104.5	2.8	51.7	108.1
1993–1997	3.4	30.4	123.9	3.2	75.8	145.5	0.2	6.1	72.1	2.8	40.8	118.5
1998–2002	3.6	39.0	110.7	2.2	52.8	100.3	0.2	7.5	72.3	3.0	35.9	115.5
2003–2007	3.2	39.7	99.1	1.8	37.9	84.1	0.8	11.5	91.2	2.8	27.0	120.2
2008–2012	3.0	36.4	97.8	2.2	36.7	88.4	1.4	14.3	105.5	2.4	20.9	108.7

Secondary Medical Zone ID: 200

	Male						Female					
	Suicide			Suicide			Suicide			Suicide		
	Num per year	Rate /100,000	×100	Num per year	Rate /100,000	×100	Num per year	Rate /100,000	×100	Num per year	Rate /100,000	×100
	Total (>10 y/o)			45–54			Total (>10 y/o)			45–54		
1983–1987	188.0	30.1	91.1	44.4	45.4	89.9	111.2	15.1	98.4	22.0	20.8	120.6
1988–1992	141.4	21.4	86.2	33.8	32.2	93.9	106.6	13.2	101.9	18.8	16.4	112.0
1993–1997	159.4	22.8	90.2	43.0	37.0	101.1	82.0	9.9	87.8	15.4	12.4	96.4
1998–2002	251.4	33.8	96.0	63.8	59.2	105.8	105.0	12.5	93.7	16.8	14.7	97.5
2003–2007	224.6	31.5	85.1	40.6	46.4	83.0	111.0	13.0	101.4	14.2	14.9	108.4
2008–2012	223.6	31.9	91.7	38.6	45.3	93.3	108.2	13.2	100.1	15.4	16.7	112.1
	15–24			55–64			15–24			55–64		
1983–1987	15.8	11.0	78.5	32.0	47.6	112.0	8.8	6.9	100.6	19.4	23.0	116.4
1988–1992	12.6	8.5	85.6	25.6	31.3	88.2	10.0	7.0	131.9	16.2	17.7	99.3
1993–1997	13.6	9.6	85.0	33.4	36.9	91.7	4.8	3.8	72.6	16.2	16.4	102.9
1998–2002	15.2	12.7	80.6	64.0	64.5	101.5	8.4	7.1	101.2	18.8	17.5	93.0
2003–2007	14.4	14.0	80.2	56.0	51.4	89.0	8.4	8.2	90.8	21.4	17.6	105.1
2008–2012	15.8	17.9	84.3	51.6	49.9	102.7	7.0	8.0	82.8	18.6	16.1	108.2
	25–34			65–74			25–34			65–74		
1983–1987	22.0	21.3	83.3	15.8	38.9	88.8	11.0	10.5	97.6	16.2	26.3	84.2
1988–1992	16.2	16.1	84.4	11.4	26.8	76.1	10.0	9.6	110.9	16.0	24.0	91.9
1993–1997	21.0	19.1	97.5	16.2	29.1	91.1	10.6	9.3	112.1	13.6	18.2	93.4
1998–2002	26.2	22.4	82.9	36.0	50.4	110.8	15.6	12.6	109.0	16.6	19.8	94.1
2003–2007	30.4	27.0	87.0	27.4	35.0	81.6	18.8	15.6	119.7	21.4	22.6	121.2
2008–2012	29.0	30.8	94.4	28.2	33.2	84.2	13.4	13.5	94.5	19.6	19.1	107.3
	35–44			>74 y/o			35–44			>74 y/o		
1983–1987	38.6	30.6	96.1	19.0	71.9	91.8	15.0	11.4	96.2	18.2	46.2	80.1
1988–1992	25.0	22.2	93.6	16.6	53.6	83.1	14.0	11.5	119.1	20.6	38.4	80.3
1993–1997	19.8	21.6	88.0	12.4	38.1	73.1	8.2	8.5	100.2	13.2	20.9	60.8
1998–2002	27.0	30.2	85.8	18.8	48.3	91.6	12.2	12.7	122.4	16.2	21.4	69.0
2003–2007	37.2	37.6	93.8	18.0	36.7	81.6	12.2	12.0	94.7	14.4	16.0	71.4
2008–2012	39.4	35.9	96.4	20.6	34.0	82.0	18.6	15.8	116.4	15.4	15.0	77.8

Secondary Medical Zone ID: 201

	Male						Female					
	Suicide			Suicide			Suicide			Suicide		
	Num per year	Rate /100,000	×100	Num per year	Rate /100,000	×100	Num per year	Rate /100,000	×100	Num per year	Rate /100,000	×100
	Total (>10 y/o)			45–54			Total (>10 y/o)			45–54		
1983–1987	39.4	29.2	82.9	11.8	46.0	90.9	19.2	13.5	84.5	2.4	12.8	74.3
1988–1992	33.2	21.2	81.5	7.4	24.7	72.0	21.4	12.0	92.0	5.4	16.4	111.9
1993–1997	36.0	20.7	76.8	8.2	24.8	67.8	19.6	10.0	87.8	5.4	13.7	105.8
1998–2002	69.8	33.9	94.1	19.4	56.9	101.6	26.4	12.1	92.6	5.8	15.4	102.4
2003–2007	66.2	33.2	88.1	13.4	51.6	92.3	24.6	11.7	87.3	3.4	12.5	90.9
2008–2012	54.4	29.4	79.4	8.8	39.9	82.1	23.0	11.2	83.0	2.6	12.1	81.0
	15–24			55–64			15–24			55–64		
1983–1987	2.0	9.3	66.4	5.2	37.6	88.5	1.8	6.7	98.2	3.0	19.4	98.5
1988–1992	3.4	9.4	94.9	6.2	31.4	88.4	1.0	3.8	71.3	3.6	18.0	100.7
1993–1997	3.8	10.2	90.4	8.2	33.9	84.2	1.4	4.3	83.0	3.6	15.1	95.1
1998–2002	6.0	17.1	108.5	16.4	54.2	85.2	3.4	9.3	132.1	6.0	19.3	102.5
2003–2007	3.4	13.8	79.2	17.2	49.8	86.4	2.2	8.6	96.0	5.4	15.2	90.8
2008–2012	4.2	18.1	85.2	10.2	35.3	72.6	2.0	9.2	94.8	2.8	10.3	68.9
	25–34			65–74			25–34			65–74		
1983–1987	4.4	20.0	78.1	4.6	55.3	126.2	2.4	9.5	87.9	2.6	26.9	86.1
1988–1992	4.0	17.7	92.5	2.0	26.4	75.1	1.6	7.4	84.8	3.6	27.5	105.6
1993–1997	4.0	16.1	82.5	3.6	28.4	88.9	1.4	6.4	77.0	2.4	16.6	85.4
1998–2002	7.2	23.8	88.0	7.4	42.1	92.7	2.4	9.0	78.0	3.0	17.4	82.9
2003–2007	11.4	35.4	114.2	6.4	31.4	73.3	4.8	14.5	111.3	2.2	12.5	67.4
2008–2012	8.4	33.2	102.0	8.0	31.1	78.7	2.6	11.9	82.8	4.2	15.7	88.3
	35–44			>74 y/o			35–44			>74 y/o		
1983–1987	8.6	23.6	74.1	2.8	72.2	92.2	4.0	10.7	90.7	3.0	49.7	86.1
1988–1992	7.0	21.1	88.9	3.2	60.3	93.5	2.6	8.0	83.2	3.6	39.4	82.3
1993–1997	4.6	18.7	76.3	3.4	52.5	100.8	3.2	10.1	118.5	1.8	18.0	52.4
1998–2002	7.0	29.9	84.9	6.0	67.3	127.7	3.2	11.6	111.8	2.6	19.3	62.0
2003–2007	9.8	36.6	91.2	4.6	40.9	90.8	3.4	12.3	97.4	3.2	17.5	77.9
2008–2012	9.6	31.8	85.5	5.0	34.3	82.6	3.8	12.4	91.5	5.0	20.5	106.8

Secondary Medical Zone ID: 202

	Male						Female					
	Suicide			Suicide			Suicide			Suicide		
	Num per year	Rate /100,000	×100	Num per year	Rate /100,000	×100	Num per year	Rate /100,000	×100	Num per year	Rate /100,000	×100
	Total (>10 y/o)			45-54			Total (>10 y/o)			45-54		
1983-1987	5.6	28.9	73.9	2.6	54.6	108.0	5.4	15.8	105.0	0.8	17.6	102.4
1988-1992	7.2	23.7	91.9	1.6	31.6	92.1	4.6	12.4	95.3	0.4	13.1	89.7
1993-1997	8.0	23.7	87.8	1.4	28.8	78.7	6.2	11.6	114.7	1.0	13.2	101.9
1998-2002	11.4	31.7	78.2	2.8	45.1	80.6	6.2	12.5	97.7	1.2	14.9	99.2
2003-2007	11.4	30.6	70.4	2.4	42.4	75.8	6.4	12.2	95.5	0.6	12.0	87.7
2008-2012	14.4	32.4	87.4	2.8	45.9	94.4	5.0	11.7	83.8	0.6	12.9	86.5
	15-24			55-64			15-24			55-64		
1983-1987	0.0	10.0	71.6	0.0	27.6	64.8	0.0	5.7	83.5	0.6	18.8	95.5
1988-1992	0.4	9.3	94.2	1.2	32.8	92.2	0.2	4.9	92.8	1.0	19.2	107.4
1993-1997	0.4	10.0	88.3	2.2	42.2	104.7	0.4	5.4	103.5	0.8	16.1	101.4
1998-2002	0.6	13.8	87.4	3.0	55.2	86.8	0.6	7.5	107.2	1.2	19.1	101.5
2003-2007	1.4	19.2	109.9	2.4	41.5	71.8	0.6	9.3	102.9	1.0	15.5	92.3
2008-2012	1.2	21.7	102.1	2.6	40.8	83.9	0.2	7.9	81.7	1.0	13.8	92.3
	25-34			65-74			25-34			65-74		
1983-1987	1.0	25.5	99.6	0.2	33.6	76.8	1.0	13.5	125.6	0.6	27.5	87.9
1988-1992	1.6	26.0	136.2	0.6	32.1	91.2	0.2	7.8	90.3	0.6	23.0	88.1
1993-1997	0.2	14.9	76.3	1.0	31.7	99.3	0.2	7.4	89.1	1.8	29.0	148.8
1998-2002	0.8	22.4	83.0	2.0	46.9	103.1	0.6	11.0	94.6	1.0	21.5	102.3
2003-2007	1.6	27.7	89.2	0.8	31.1	72.4	1.0	13.0	100.1	2.2	26.2	140.6
2008-2012	1.4	29.0	89.1	2.4	39.3	99.4	1.0	14.3	99.8	1.0	17.5	98.1
	35-44			>74 y/o			35-44			>74 y/o		
1983-1987	1.2	26.6	83.4	0.6	64.3	82.1	0.6	11.4	96.4	1.8	77.7	134.7
1988-1992	1.0	19.9	84.0	0.8	59.2	91.7	0.2	8.0	83.2	2.0	64.4	134.7
1993-1997	1.8	27.0	110.3	1.0	54.5	104.7	1.0	10.4	122.0	1.0	32.3	93.7
1998-2002	1.2	28.3	80.4	1.0	50.4	95.6	0.8	10.9	105.6	0.8	24.7	79.5
2003-2007	1.4	28.6	71.5	1.4	45.9	102.0	0.4	10.5	83.2	0.6	16.4	73.3
2008-2012	3.0	36.4	97.9	1.0	34.9	84.2	0.4	10.8	79.3	0.8	16.4	85.1

Secondary Medical Zone ID: 203

	Male						Female					
	Suicide			Suicide			Suicide			Suicide		
	Num per year	Rate /100,000	×100	Num per year	Rate /100,000	×100	Num per year	Rate /100,000	×100	Num per year	Rate /100,000	×100
	Total (>10 y/o)			45-54			Total (>10 y/o)			45-54		
1983-1987	93.2	25.9	75.6	24.6	37.7	74.5	52.4	13.5	86.8	9.0	14.2	82.7
1988-1992	82.6	20.8	82.3	20.4	27.9	81.2	51.2	11.6	89.6	10.2	13.6	92.9
1993-1997	90.2	20.6	81.2	26.6	33.2	90.7	44.8	9.4	84.4	11.2	13.2	101.9
1998-2002	155.6	33.3	93.4	36.8	50.8	90.8	60.6	12.0	92.4	10.0	13.3	88.6
2003-2007	143.8	31.7	85.6	29.0	48.6	86.9	65.0	12.5	98.6	8.8	13.7	99.8
2008-2012	127.4	28.8	81.4	23.2	39.9	82.0	65.4	13.1	96.7	10.6	16.5	110.4
	15-24			55-64			15-24			55-64		
1983-1987	8.4	10.5	75.0	13.2	36.3	85.5	4.6	6.2	90.2	6.4	16.6	83.9
1988-1992	7.0	8.1	81.9	14.2	29.2	82.1	4.8	5.6	106.7	8.4	16.6	93.0
1993-1997	9.6	11.3	100.0	17.8	30.9	76.7	4.0	5.1	97.2	5.8	10.5	66.0
1998-2002	13.0	17.7	112.3	43.4	65.6	103.3	4.8	7.2	101.9	10.2	15.4	81.6
2003-2007	11.8	18.8	107.7	34.4	49.3	85.4	4.4	8.0	89.0	13.4	17.2	102.8
2008-2012	7.2	14.8	69.7	23.8	37.5	77.3	5.2	10.2	104.9	12.2	16.2	108.7
	25-34			65-74			25-34			65-74		
1983-1987	13.4	19.6	76.5	6.8	38.9	89.0	6.4	9.1	84.4	7.0	28.1	90.0
1988-1992	13.8	19.9	103.9	6.4	30.1	85.4	5.2	7.7	88.5	6.6	22.6	86.5
1993-1997	11.6	16.2	83.0	8.0	26.6	83.4	6.2	8.3	100.2	6.2	16.9	86.8
1998-2002	20.0	25.9	95.7	17.0	42.1	92.5	7.4	9.8	84.7	12.8	26.4	125.7
2003-2007	21.4	30.7	99.0	16.4	34.3	80.0	11.2	14.9	114.7	8.8	16.3	87.4
2008-2012	17.2	30.2	92.8	19.6	35.7	90.4	7.4	12.6	87.7	9.2	14.8	83.3
	35-44			>74 y/o			35-44			>74 y/o		
1983-1987	20.4	23.5	74.0	6.2	64.4	82.2	10.0	11.0	92.8	8.8	54.8	95.1
1988-1992	14.8	19.0	80.3	5.6	47.5	73.6	9.2	10.7	110.8	6.6	31.5	65.8
1993-1997	10.6	17.0	69.5	5.8	41.4	79.5	4.6	7.1	82.9	6.4	24.5	71.3
1998-2002	18.0	28.9	82.1	7.4	41.3	78.3	8.8	12.7	123.2	6.4	20.1	64.6
2003-2007	19.4	28.4	71.0	11.2	42.6	94.6	9.0	12.4	98.0	9.2	21.1	94.1
2008-2012	24.0	31.7	85.3	12.2	35.0	84.3	14.2	16.5	122.0	6.6	12.4	64.6

Secondary Medical Zone ID: 204

	Male						Female					
	Suicide			Suicide			Suicide			Suicide		
	Num per year	Rate /100,000	×100	Num per year	Rate /100,000	×100	Num per year	Rate /100,000	×100	Num per year	Rate /100,000	×100
	Total (>10 y/o)			45–54			Total (>10 y/o)			45–54		
1983–1987	65.6	26.4	76.1	19.2	41.2	81.5	33.6	13.2	84.1	6.2	14.8	85.9
1988–1992	55.8	20.0	77.7	14.8	27.1	79.0	35.6	11.6	90.2	7.6	14.0	95.5
1993–1997	66.4	21.1	81.5	19.6	32.9	89.8	34.4	9.9	91.1	9.0	13.9	107.6
1998–2002	111.4	32.6	90.2	22.6	43.9	78.4	47.0	12.7	99.0	9.2	15.7	104.6
2003–2007	94.6	28.7	76.4	15.6	38.3	68.5	35.0	10.1	76.1	4.8	11.4	83.2
2008–2012	96.6	29.7	83.5	15.4	39.6	81.5	43.6	12.5	90.3	5.4	13.5	90.3
	15–24			55–64			15–24			55–64		
1983–1987	4.8	9.1	65.4	8.6	35.3	83.0	2.2	4.9	71.9	5.6	20.5	104.1
1988–1992	4.4	7.5	75.4	10.6	30.8	86.6	3.2	5.2	98.7	6.2	17.9	100.3
1993–1997	5.6	9.5	84.4	14.2	33.1	82.3	3.4	5.6	107.2	7.6	17.4	109.2
1998–2002	7.0	14.4	91.0	31.4	61.1	96.2	4.4	8.5	120.7	10.0	19.1	101.6
2003–2007	6.6	16.6	95.0	24.2	45.3	78.4	2.6	7.2	80.2	7.6	13.7	82.1
2008–2012	7.4	20.8	98.1	18.2	38.7	79.7	3.2	9.3	96.3	9.4	16.3	109.5
	25–34			65–74			25–34			65–74		
1983–1987	6.0	13.7	53.7	5.2	43.0	98.3	4.4	9.1	84.4	3.6	24.0	76.7
1988–1992	7.4	15.1	79.2	4.4	30.0	85.2	5.0	9.8	112.9	2.6	15.6	59.6
1993–1997	9.6	17.2	87.8	4.2	21.6	67.6	4.6	8.4	101.4	1.8	9.8	50.2
1998–2002	17.4	27.8	103.0	13.6	45.7	100.5	7.4	12.1	104.7	6.2	19.8	94.4
2003–2007	13.4	24.5	79.1	14.4	38.9	90.7	5.4	10.3	78.8	6.2	16.4	88.3
2008–2012	10.0	23.8	73.0	13.2	30.9	78.2	8.8	17.4	121.2	5.8	13.4	75.0
	35–44			>74 y/o			35–44			>74 y/o		
1983–1987	17.6	27.2	85.5	4.0	70.7	90.2	6.8	10.5	88.6	4.4	47.6	82.6
1988–1992	9.8	17.8	75.3	4.2	54.1	83.9	4.8	8.4	87.2	6.0	42.4	88.6
1993–1997	9.2	20.5	83.5	4.0	42.3	81.3	3.2	7.2	84.6	4.8	27.0	78.3
1998–2002	13.6	31.3	89.0	5.4	43.1	81.8	5.2	11.4	110.6	4.6	21.1	67.9
2003–2007	15.2	31.2	77.8	5.2	30.8	68.4	3.6	8.7	69.0	4.8	16.6	74.0
2008–2012	22.4	37.7	101.4	9.6	38.2	92.1	7.2	12.5	92.5	3.4	10.3	53.7

Secondary Medical Zone ID: 205

	Male						Female					
	Suicide		×100	Suicide		×100	Suicide		×100	Suicide		×100
	Num per year	Rate /100,000		Num per year	Rate /100,000		Num per year	Rate /100,000		Num per year	Rate /100,000	
	Total (>10 y/o)			45-54			Total (>10 y/o)			45-54		
1983-1987	127.8	29.4	89.2	39.8	49.2	97.2	73.2	16.4	108.9	14.4	18.8	109.2
1988-1992	105.2	22.3	89.4	30.2	31.9	93.0	64.6	12.8	100.0	12.2	13.2	90.6
1993-1997	120.6	22.7	90.0	36.6	36.2	99.0	58.0	10.1	93.8	13.2	12.6	97.2
1998-2002	219.0	37.9	108.4	46.2	53.1	94.8	77.6	12.8	100.5	15.6	16.4	109.0
2003-2007	193.6	34.6	96.5	35.0	51.0	91.2	73.0	12.4	95.8	8.8	12.5	91.4
2008-2012	178.6	33.2	97.0	30.2	46.6	95.9	73.6	12.8	95.9	12.0	17.4	116.9
	15-24			55-64			15-24			55-64		
1983-1987	9.6	10.2	73.1	15.4	39.1	91.9	6.8	7.3	106.5	9.0	20.9	105.8
1988-1992	11.2	10.2	102.6	20.2	35.2	99.1	5.0	4.8	92.0	8.4	15.6	87.5
1993-1997	10.4	10.4	91.7	26.6	36.0	89.5	4.0	4.2	81.4	11.6	16.1	101.2
1998-2002	13.8	16.9	107.0	72.0	81.6	128.4	4.6	6.1	87.0	17.4	19.4	103.3
2003-2007	11.0	16.8	96.5	55.8	60.8	105.4	7.0	10.5	117.0	16.2	16.4	98.0
2008-2012	12.8	21.8	102.7	35.4	44.0	90.5	5.8	10.1	104.3	12.6	14.2	94.9
	25-34			65-74			25-34			65-74		
1983-1987	17.6	22.4	87.5	7.6	40.3	92.2	9.0	11.3	104.7	8.6	30.9	98.9
1988-1992	11.6	15.2	79.7	7.6	32.9	93.3	8.4	10.6	122.3	10.8	32.4	124.0
1993-1997	13.2	15.0	76.6	10.6	31.6	98.9	6.2	7.2	87.0	7.8	19.6	100.7
1998-2002	25.4	26.1	96.7	25.4	52.5	115.4	11.6	12.0	103.1	10.4	20.6	97.9
2003-2007	25.2	29.7	95.6	27.6	44.1	102.8	10.0	11.8	91.1	11.6	17.8	95.6
2008-2012	18.0	28.7	88.2	31.0	40.5	102.6	8.6	13.3	93.1	14.2	17.2	96.6
	35-44			>74 y/o			35-44			>74 y/o		
1983-1987	31.2	28.4	89.3	6.2	68.2	87.1	14.6	12.7	107.5	10.8	71.5	123.9
1988-1992	16.0	17.6	74.2	8.2	64.5	100.0	8.2	8.6	89.3	11.6	51.2	106.9
1993-1997	17.6	23.4	95.6	5.2	37.1	71.3	5.8	7.8	91.7	9.4	32.5	94.3
1998-2002	25.8	35.7	101.5	10.0	52.1	98.9	9.8	13.1	126.8	8.2	23.3	75.0
2003-2007	28.0	35.1	87.6	10.8	40.1	89.1	11.6	14.1	111.5	7.8	17.3	77.0
2008-2012	33.2	36.9	99.2	17.6	44.8	108.0	11.4	12.7	93.9	9.0	15.5	80.5

Secondary Medical Zone ID: 206

	Male						Female					
	Suicide			Suicide			Suicide			Suicide		
	Num per year	Rate /100,000	×100	Num per year	Rate /100,000	×100	Num per year	Rate /100,000	×100	Num per year	Rate /100,000	×100
	Total (>10 y/o)			45–54			Total (>10 y/o)			45–54		
1983–1987	96.4	30.0	89.2	25.2	42.3	83.7	51.6	14.7	96.6	9.8	16.8	97.3
1988–1992	81.6	23.6	93.5	21.4	32.2	93.9	39.4	10.7	79.8	7.2	11.5	78.8
1993–1997	89.2	23.0	92.6	23.8	34.6	94.5	45.0	10.6	97.0	7.6	11.0	85.5
1998–2002	147.6	36.0	102.4	37.6	61.9	110.6	55.4	12.4	98.0	8.6	14.0	92.7
2003–2007	141.0	35.6	98.4	28.6	59.1	105.6	50.6	12.0	92.1	6.4	13.1	95.6
2008–2012	131.0	34.8	102.2	21.6	47.3	97.2	54.8	13.7	101.7	9.0	18.3	122.7
	15–24			55–64			15–24			55–64		
1983–1987	7.4	10.4	74.3	15.4	45.6	107.4	4.0	6.2	90.6	8.4	21.8	110.6
1988–1992	5.4	7.5	75.8	13.0	29.6	83.3	3.4	4.9	92.3	7.0	15.8	88.6
1993–1997	5.8	8.7	77.3	24.2	43.6	108.2	4.0	5.9	112.7	8.2	15.0	94.2
1998–2002	6.8	12.6	79.4	46.4	74.6	117.4	3.2	6.3	89.9	12.2	19.0	100.7
2003–2007	7.4	15.7	90.2	39.4	61.4	106.4	2.8	6.9	76.6	10.2	15.3	91.2
2008–2012	8.6	20.7	97.5	27.0	48.5	99.8	3.8	9.7	99.7	11.0	17.4	116.4
	25–34			65–74			25–34			65–74		
1983–1987	12.0	22.4	87.8	7.4	42.8	97.8	5.2	9.9	91.8	8.8	33.8	108.1
1988–1992	10.6	19.0	99.6	8.4	40.5	115.0	5.2	9.3	107.8	6.2	22.3	85.3
1993–1997	10.6	17.1	87.7	9.4	34.1	106.8	6.2	9.6	115.6	5.4	16.6	84.9
1998–2002	15.6	23.7	87.7	17.6	47.2	103.8	8.6	12.6	108.9	9.6	22.8	108.7
2003–2007	15.2	26.1	84.3	19.0	41.4	96.4	9.0	14.6	112.6	8.6	17.3	92.9
2008–2012	15.0	34.0	104.3	24.0	44.9	113.6	6.6	14.9	103.8	8.6	15.3	85.8
	35–44			>74 y/o			35–44			>74 y/o		
1983–1987	21.2	28.6	89.7	7.4	80.1	102.3	7.8	10.5	88.9	7.4	50.6	87.7
1988–1992	16.0	24.8	104.7	6.8	60.9	94.4	4.8	7.9	81.9	5.6	29.3	61.2
1993–1997	10.6	21.1	85.9	4.8	40.2	77.2	5.4	9.9	116.9	8.2	33.0	95.8
1998–2002	17.4	34.4	97.7	6.2	41.6	78.9	5.8	11.3	109.0	7.4	24.8	80.0
2003–2007	20.4	36.1	90.1	11.0	49.9	110.8	8.0	13.7	108.7	5.6	15.4	68.7
2008–2012	25.0	40.5	109.0	9.6	34.2	82.4	9.4	15.0	110.3	6.2	14.1	73.3

Secondary Medical Zone ID: 207

	Male						Female					
	Suicide			Suicide			Suicide			Suicide		
	Num per year	Rate /100,000	×100	Num per year	Rate /100,000	×100	Num per year	Rate /100,000	×100	Num per year	Rate /100,000	×100
	Total (>10 y/o)			45–54			Total (>10 y/o)			45–54		
1983–1987	61.6	29.1	85.0	16.2	41.0	81.2	28.8	12.8	80.4	6.0	15.8	91.5
1988–1992	55.6	22.3	89.6	15.0	31.9	92.9	32.6	11.6	89.7	6.8	14.3	97.5
1993–1997	63.0	22.6	88.9	19.2	37.1	101.4	36.8	11.1	103.5	6.4	12.0	92.8
1998–2002	92.2	31.7	85.6	24.0	51.6	92.2	43.4	12.3	98.4	6.8	13.8	91.5
2003–2007	100.2	33.5	92.7	19.2	50.1	89.6	38.0	11.6	88.9	5.2	12.8	93.1
2008–2012	100.2	35.1	100.8	19.0	51.8	106.6	42.0	12.4	97.2	6.8	16.5	110.9
	15–24			55–64			15–24			55–64		
1983–1987	6.2	13.5	97.0	10.2	42.5	100.1	2.2	5.3	78.3	3.2	14.6	73.8
1988–1992	5.6	10.2	103.5	10.4	32.4	91.3	2.0	4.1	78.2	5.2	16.3	91.5
1993–1997	4.4	9.3	81.9	14.6	37.4	93.0	2.6	5.0	95.3	4.6	12.5	78.8
1998–2002	6.6	15.4	97.2	25.0	55.7	87.6	4.0	8.4	119.1	10.2	20.7	110.2
2003–2007	4.8	14.1	80.6	27.2	56.1	97.2	4.0	10.1	112.2	9.2	17.1	102.4
2008–2012	6.2	19.0	89.6	20.4	46.4	95.4	2.6	8.2	84.8	8.2	16.0	107.2
	25–34			65–74			25–34			65–74		
1983–1987	5.2	16.8	65.7	4.8	40.4	92.4	2.0	6.8	63.2	5.0	29.9	95.8
1988–1992	6.6	18.2	95.0	5.6	36.0	102.1	2.6	7.2	83.4	4.4	22.3	85.4
1993–1997	8.0	18.9	96.9	3.2	18.1	56.8	4.0	8.6	103.7	5.8	21.9	112.6
1998–2002	9.0	21.0	77.8	9.4	35.4	77.9	4.2	9.4	80.9	6.8	21.4	102.1
2003–2007	11.8	29.2	94.2	16.2	46.4	108.2	4.4	10.9	83.5	4.2	12.9	69.4
2008–2012	11.0	33.9	104.0	14.0	35.4	89.6	3.6	11.8	82.6	8.4	18.5	103.9
	35–44			>74 y/o			35–44			>74 y/o		
1983–1987	12.0	24.5	76.9	6.8	101.3	129.3	5.6	10.6	90.1	4.6	47.3	82.0
1988–1992	7.8	17.7	74.7	4.4	54.3	84.2	5.8	10.9	113.3	5.8	39.8	83.3
1993–1997	7.4	19.3	78.7	6.2	58.9	113.0	5.2	11.1	130.8	8.0	40.8	118.7
1998–2002	12.2	32.0	90.7	5.8	45.9	87.0	3.2	8.5	82.2	8.0	32.6	104.8
2003–2007	14.2	35.4	88.3	6.8	38.4	85.3	5.0	11.7	92.5	5.8	19.1	85.2
2008–2012	19.2	42.5	114.3	10.0	41.2	99.3	5.2	11.8	87.0	7.2	18.8	97.6

Secondary Medical Zone ID: 208

	Male						Female					
	Suicide			Suicide			Suicide			Suicide		
	Num per year	Rate /100,000	×100	Num per year	Rate /100,000	×100	Num per year	Rate /100,000	×100	Num per year	Rate /100,000	×100
	Total (>10 y/o)			45–54			Total (>10 y/o)			45–54		
1983–1987	104.0	33.1	99.9	30.8	53.1	104.9	51.0	15.3	99.8	9.4	17.0	98.9
1988–1992	84.2	24.4	99.0	22.2	33.6	97.9	49.0	12.7	100.4	12.4	17.9	122.2
1993–1997	93.0	24.5	98.9	24.2	34.9	95.5	46.8	11.1	102.4	9.2	12.6	97.5
1998–2002	154.4	38.7	109.9	34.6	56.9	101.6	58.4	13.3	103.9	9.0	14.0	92.7
2003–2007	132.6	33.8	95.1	22.2	47.5	84.9	60.4	13.8	108.3	7.4	14.2	103.7
2008–2012	126.2	33.5	96.6	21.8	48.2	99.3	60.0	13.8	106.0	8.2	16.7	112.1
	15–24			55–64			15–24			55–64		
1983–1987	7.4	11.8	84.6	13.6	42.6	100.2	3.4	5.7	84.0	8.4	22.9	116.1
1988–1992	7.8	10.6	107.0	14.6	33.7	94.8	4.4	6.0	114.3	7.2	16.7	93.4
1993–1997	9.4	13.4	118.8	22.6	42.4	105.4	3.6	5.4	103.9	9.8	18.1	113.9
1998–2002	8.8	16.6	105.2	44.0	72.5	114.0	3.8	7.2	102.9	12.0	19.2	102.2
2003–2007	4.2	11.8	67.5	37.8	59.2	102.6	4.8	10.8	120.0	13.0	18.3	109.4
2008–2012	9.2	22.7	107.1	25.4	44.9	92.5	5.2	12.6	129.7	12.0	17.7	118.7
	25–34			65–74			25–34			65–74		
1983–1987	11.8	21.6	84.7	9.2	55.5	126.8	7.6	12.5	116.1	7.8	34.1	109.0
1988–1992	8.8	16.8	88.1	9.0	44.1	125.2	3.6	7.1	82.3	8.0	29.6	113.3
1993–1997	9.4	16.5	84.5	8.8	32.6	102.1	7.0	11.0	131.9	6.8	20.5	105.3
1998–2002	18.4	28.5	105.7	19.2	51.9	114.1	8.0	12.2	105.4	7.6	19.2	91.5
2003–2007	17.8	30.4	98.1	21.2	47.0	109.5	9.4	14.8	114.1	10.2	20.5	110.1
2008–2012	13.8	29.3	90.1	21.0	40.4	102.2	7.8	15.1	105.4	11.6	19.6	110.2
	35–44			>74 y/o			35–44			>74 y/o		
1983–1987	23.2	30.2	94.7	7.4	90.5	115.6	7.6	9.9	83.6	6.2	49.2	85.2
1988–1992	14.8	22.5	94.9	6.8	65.2	101.1	5.8	8.6	89.3	7.2	38.8	81.1
1993–1997	10.4	20.7	84.4	7.4	58.8	112.9	4.2	8.0	93.9	6.2	26.6	77.4
1998–2002	19.2	39.5	112.1	9.8	60.0	113.9	8.0	14.7	142.4	9.6	31.7	101.9
2003–2007	19.8	36.9	92.2	9.6	43.8	97.2	9.2	15.6	123.3	6.2	16.6	74.1
2008–2012	23.2	36.1	97.0	11.4	38.9	93.8	7.2	11.6	85.7	7.8	16.5	86.0

Secondary Medical Zone ID: 209

	Male						Female					
	Suicide			Suicide			Suicide			Suicide		
	Num per year	Rate /100,000	×100	Num per year	Rate /100,000	×100	Num per year	Rate /100,000	×100	Num per year	Rate /100,000	×100
	Total (>10 y/o)			45–54			Total (>10 y/o)			45–54		
1983–1987	108.4	34.8	108.2	36.0	65.1	128.8	51.8	15.4	99.8	8.2	15.5	89.9
1988–1992	87.4	25.5	104.8	19.0	31.3	91.1	58.0	14.1	114.2	10.4	16.2	110.8
1993–1997	95.2	24.9	100.7	22.4	33.4	91.2	50.2	11.2	105.3	8.6	12.5	96.6
1998–2002	153.6	37.3	104.8	36.4	56.6	101.2	62.8	13.3	105.2	12.4	17.7	117.6
2003–2007	154.2	36.7	102.4	31.0	56.9	101.8	67.6	13.6	112.2	9.8	16.2	118.6
2008–2012	137.2	34.2	97.4	28.2	53.0	109.1	64.8	13.4	106.3	10.4	18.0	120.9
	15–24			55–64			15–24			55–64		
1983–1987	8.0	13.4	96.3	15.0	43.7	102.8	3.4	5.8	85.5	6.2	16.9	85.9
1988–1992	7.8	11.2	113.6	19.6	43.2	121.6	4.0	5.8	109.3	10.6	21.8	121.8
1993–1997	9.0	13.1	116.0	22.4	43.2	107.2	4.2	6.1	117.5	8.2	15.5	97.6
1998–2002	9.2	16.1	101.7	40.0	67.7	106.5	5.8	9.5	134.7	12.6	20.2	107.2
2003–2007	6.4	13.8	79.4	38.8	59.7	103.4	3.6	7.7	85.9	14.6	20.2	120.7
2008–2012	6.8	15.8	74.3	27.4	44.9	92.5	2.4	6.3	64.5	10.0	14.9	100.1
	25–34			65–74			25–34			65–74		
1983–1987	9.6	19.7	77.0	9.8	54.8	125.2	5.4	10.1	93.8	8.8	34.1	109.0
1988–1992	7.6	15.6	81.4	8.2	37.9	107.5	5.6	10.1	116.2	9.0	29.7	113.7
1993–1997	12.0	20.3	103.8	12.2	40.3	126.3	4.6	7.7	92.9	7.6	20.8	106.6
1998–2002	19.6	28.9	107.2	17.0	45.2	99.5	6.6	9.9	85.1	7.4	17.7	84.3
2003–2007	18.0	29.2	94.2	22.4	49.0	114.1	9.4	14.0	107.7	10.4	20.2	108.5
2008–2012	19.8	38.0	116.8	18.0	35.2	89.2	8.6	15.8	110.4	11.0	18.7	105.1
	35–44			>74 y/o			35–44			>74 y/o		
1983–1987	22.8	32.6	102.6	6.6	78.1	99.8	8.4	11.8	100.0	11.2	74.7	129.5
1988–1992	15.6	24.2	102.3	9.4	80.3	124.4	6.0	9.2	95.0	12.2	54.8	114.4
1993–1997	11.0	20.6	84.0	6.2	47.3	90.9	5.6	9.5	111.9	11.4	40.9	119.0
1998–2002	21.0	37.8	107.3	9.8	56.3	106.8	6.8	11.6	112.6	11.2	32.8	105.7
2003–2007	23.8	38.8	96.9	13.8	55.7	123.6	8.6	13.3	105.4	11.2	25.7	114.5
2008–2012	25.4	37.4	100.4	11.6	37.5	90.4	11.4	15.4	113.7	10.8	20.3	105.6

Secondary Medical Zone ID: 210

	Male						Female					
	Suicide			Suicide			Suicide			Suicide		
	Num per year	Rate /100,000	×100	Num per year	Rate /100,000	×100	Num per year	Rate /100,000	×100	Num per year	Rate /100,000	×100
	Total (>10 y/o)			45–54			Total (>10 y/o)			45–54		
1983–1987	385.4	36.0	110.9	109.0	58.9	116.5	191.6	16.1	105.6	34.4	18.1	104.8
1988–1992	325.6	28.6	118.0	85.4	44.5	129.5	157.0	12.5	96.0	29.8	15.5	106.2
1993–1997	347.8	28.8	117.1	88.4	43.7	119.6	146.6	11.3	100.2	29.0	14.8	114.6
1998–2002	589.4	46.1	133.8	149.0	79.9	142.8	201.6	14.8	114.7	32.8	18.5	122.8
2003–2007	532.0	42.1	120.2	91.6	59.8	106.9	202.0	15.5	117.3	24.8	16.6	121.2
2008–2012	486.4	38.9	117.0	85.2	56.8	116.8	220.0	17.0	125.8	31.4	20.9	139.8
	15–24			55–64			15–24			55–64		
1983–1987	20.0	10.1	72.2	68.4	54.5	128.1	16.0	8.0	117.4	30.2	20.7	105.1
1988–1992	18.8	9.3	93.8	69.0	44.9	126.4	13.2	6.5	123.5	25.4	15.9	88.8
1993–1997	19.6	10.5	93.1	87.8	51.9	129.0	10.6	5.7	109.9	26.4	15.2	95.5
1998–2002	24.8	15.6	98.8	181.0	101.6	159.9	13.2	8.2	117.2	39.6	21.8	115.6
2003–2007	25.4	18.6	106.4	153.6	81.4	141.1	17.2	12.2	136.1	38.2	20.3	121.5
2008–2012	26.4	21.9	103.3	116.6	65.0	133.8	17.2	13.5	139.4	30.6	17.8	119.3
	25–34			65–74			25–34			65–74		
1983–1987	40.8	22.9	89.4	37.2	54.2	123.9	19.8	11.7	108.7	29.8	30.7	98.2
1988–1992	34.0	19.0	99.6	31.4	40.9	116.0	18.8	10.7	123.4	26.4	24.6	94.4
1993–1997	37.0	19.2	98.2	41.6	42.0	131.7	22.0	11.3	135.5	21.4	17.3	88.8
1998–2002	59.2	28.7	106.1	80.4	64.1	140.9	33.2	15.8	136.0	33.6	23.2	110.5
2003–2007	63.6	32.7	105.4	87.4	61.4	143.1	34.6	17.1	131.2	30.8	19.3	103.8
2008–2012	56.0	32.0	98.4	80.2	53.5	135.3	37.2	20.0	139.6	35.0	20.9	117.4
	35–44			>74 y/o			35–44			>74 y/o		
1983–1987	76.8	36.3	114.0	31.6	88.2	112.6	31.6	15.0	127.1	29.0	51.3	89.0
1988–1992	56.4	29.1	123.0	29.0	67.8	105.1	17.6	9.7	100.5	25.0	34.5	72.1
1993–1997	46.8	29.1	118.9	25.4	53.4	102.5	14.8	9.7	114.0	21.4	24.5	71.1
1998–2002	59.6	38.4	109.0	33.8	57.8	109.7	20.4	13.3	128.2	28.4	26.3	84.6
2003–2007	75.4	44.0	109.9	34.4	44.4	98.7	32.8	18.6	147.0	23.2	17.2	76.8
2008–2012	76.4	39.0	104.8	44.2	44.9	108.3	39.4	19.6	144.2	28.8	17.6	91.3

Secondary Medical Zone ID: 211

	Male						Female					
	Suicide			Suicide			Suicide			Suicide		
	Num per year	Rate /100,000	×100	Num per year	Rate /100,000	×100	Num per year	Rate /100,000	×100	Num per year	Rate /100,000	×100
	Total (>10 y/o)			45–54			Total (>10 y/o)			45–54		
1983–1987	191.8	34.5	107.0	54.4	58.1	114.8	102.0	15.9	104.8	21.6	21.2	123.0
1988–1992	150.4	25.0	101.9	36.4	36.2	105.5	91.6	12.9	99.9	16.2	15.2	103.8
1993–1997	154.0	24.5	98.0	38.2	35.2	96.1	82.0	11.0	101.3	14.4	12.7	98.4
1998–2002	248.0	36.2	101.8	66.4	61.9	110.6	103.8	13.1	101.6	18.0	15.9	105.8
2003–2007	243.8	35.3	97.1	44.6	48.9	87.5	105.2	13.4	102.1	16.6	16.3	118.8
2008–2012	225.6	33.5	96.7	40.8	47.2	97.0	111.6	14.2	106.9	16.8	17.2	115.6
	15–24			55–64			15–24			55–64		
1983–1987	15.0	15.2	109.1	26.2	39.7	93.3	7.6	7.4	108.5	15.0	19.7	99.8
1988–1992	12.6	11.3	113.7	28.0	34.3	96.6	7.2	6.2	117.8	14.6	16.5	92.6
1993–1997	14.2	13.3	118.0	33.0	38.7	96.1	6.2	5.7	108.6	14.2	15.3	96.2
1998–2002	17.0	17.6	111.6	63.2	66.5	104.7	8.6	8.4	119.9	18.4	18.1	95.9
2003–2007	16.0	18.6	106.9	62.0	58.0	100.5	7.4	8.6	95.2	18.2	16.0	95.5
2008–2012	17.8	23.6	111.5	50.8	48.6	100.0	9.2	11.7	120.8	20.2	17.5	117.3
	25–34			65–74			25–34			65–74		
1983–1987	25.6	28.5	111.6	16.4	45.7	104.3	11.6	12.2	113.5	16.0	32.2	103.1
1988–1992	17.8	20.2	105.7	12.8	31.0	87.9	10.6	10.8	125.1	14.8	25.6	97.9
1993–1997	17.4	19.4	99.3	16.2	30.1	94.5	11.0	10.9	130.4	11.4	17.2	88.4
1998–2002	27.8	27.6	102.0	31.2	45.1	99.2	13.8	12.7	109.3	15.8	19.4	92.5
2003–2007	31.6	32.2	103.9	32.2	41.5	96.7	17.8	16.2	124.4	18.0	19.5	104.9
2008–2012	27.4	33.0	101.2	32.0	38.2	96.6	12.4	13.6	95.0	18.6	18.8	105.8
	35–44			>74 y/o			35–44			>74 y/o		
1983–1987	40.6	35.8	112.5	12.8	68.4	87.3	13.6	11.6	98.1	16.2	51.5	89.3
1988–1992	27.4	25.2	106.1	14.8	62.0	96.1	13.2	11.4	117.8	14.8	36.3	75.9
1993–1997	22.0	24.0	98.1	12.2	47.7	91.7	8.8	9.0	105.4	15.2	32.5	94.5
1998–2002	27.8	31.8	90.2	14.6	43.2	81.9	11.0	11.4	110.0	17.6	28.6	92.1
2003–2007	35.8	38.2	95.3	21.2	43.9	97.5	14.6	14.1	111.6	12.4	15.6	69.6
2008–2012	33.6	32.8	88.3	22.8	37.8	91.1	18.8	16.3	120.4	15.0	15.2	78.9

Secondary Medical Zone ID: 212

	Male						Female					
	Suicide			Suicide			Suicide			Suicide		
	Num per year	Rate /100,000	×100	Num per year	Rate /100,000	×100	Num per year	Rate /100,000	×100	Num per year	Rate /100,000	×100
	Total (>10 y/o)			45–54			Total (>10 y/o)			45–54		
1983–1987	123.8	31.3	95.5	34.4	51.0	100.8	63.6	14.6	96.1	9.4	14.2	82.6
1988–1992	91.2	22.1	88.9	19.2	27.4	79.9	58.2	12.3	95.8	10.0	13.8	94.6
1993–1997	103.0	24.1	95.5	26.8	35.8	97.8	48.2	10.0	90.9	9.6	12.6	97.6
1998–2002	162.2	35.3	99.2	40.6	57.9	103.5	67.6	12.8	101.4	10.4	14.5	96.3
2003–2007	148.8	32.5	88.7	28.6	48.7	87.0	64.8	12.8	96.7	9.6	15.0	109.7
2008–2012	144.4	31.8	91.8	25.4	43.5	89.6	65.4	12.7	95.9	10.2	16.2	108.8
	15–24			55–64			15–24			55–64		
1983–1987	9.6	12.8	91.7	20.4	46.1	108.5	6.2	7.9	115.4	10.2	20.3	102.8
1988–1992	7.0	9.1	91.7	19.0	35.3	99.3	7.4	8.4	158.9	8.6	15.4	86.4
1993–1997	5.6	8.6	76.4	22.0	38.1	94.6	2.6	3.9	75.5	10.8	17.2	108.5
1998–2002	11.8	19.0	120.1	46.4	71.6	112.7	5.2	8.0	113.3	14.2	20.5	108.9
2003–2007	8.0	16.0	91.9	41.8	59.4	102.9	4.4	8.3	92.4	12.4	16.5	98.7
2008–2012	8.8	19.4	91.4	30.2	45.6	93.9	4.2	8.9	91.2	10.8	14.9	99.9
	25–34			65–74			25–34			65–74		
1983–1987	16.6	23.3	91.2	9.6	40.8	93.3	7.2	10.2	94.6	9.2	29.0	92.6
1988–1992	10.8	15.8	82.8	9.2	34.4	97.5	5.8	8.5	97.5	10.0	26.9	103.2
1993–1997	12.6	17.7	90.8	13.0	37.7	118.1	6.0	8.4	101.4	6.6	16.2	82.9
1998–2002	18.0	23.5	87.0	20.6	47.8	105.0	11.6	13.9	120.0	11.4	22.3	106.0
2003–2007	16.6	23.8	76.6	17.2	35.5	82.7	11.6	14.5	111.7	8.8	15.8	84.9
2008–2012	15.8	27.4	84.2	20.2	36.7	93.0	9.8	14.9	103.8	12.0	18.6	104.7
	35–44			>74 y/o			35–44			>74 y/o		
1983–1987	24.0	28.4	89.1	8.6	70.4	89.9	11.2	12.8	108.3	9.8	51.1	88.6
1988–1992	18.2	23.8	100.5	7.2	48.6	75.3	6.8	9.0	92.9	9.0	36.0	75.2
1993–1997	14.4	23.6	96.3	8.0	48.4	92.9	5.2	8.4	98.8	7.4	25.5	74.1
1998–2002	18.2	30.0	85.3	6.2	32.2	61.1	5.8	9.6	92.4	9.0	24.0	77.4
2003–2007	22.0	31.5	78.5	13.8	47.8	106.1	11.6	15.3	121.0	6.4	13.8	61.6
2008–2012	31.6	39.0	104.9	12.2	34.5	83.1	10.0	12.3	90.6	8.4	14.5	75.4

Secondary Medical Zone ID: 213

	Male						Female					
	Suicide			Suicide			Suicide			Suicide		
	Num per year	Rate /100,000	×100	Num per year	Rate /100,000	×100	Num per year	Rate /100,000	×100	Num per year	Rate /100,000	×100
	Total (>10 y/o)			45–54			Total (>10 y/o)			45–54		
1983–1987	55.0	27.3	78.7	15.4	40.8	80.7	31.6	14.0	89.7	4.4	13.1	76.1
1988–1992	43.4	18.9	72.1	9.4	22.8	66.4	33.4	12.0	93.4	4.8	11.7	80.2
1993–1997	55.2	20.6	78.2	14.6	30.0	81.9	27.8	9.3	80.9	5.2	10.6	81.9
1998–2002	94.8	31.3	84.6	23.4	48.1	85.9	40.0	11.6	89.0	5.8	12.0	79.9
2003–2007	98.4	31.7	84.3	18.6	43.9	78.5	43.0	12.5	92.9	5.0	11.4	83.3
2008–2012	91.8	29.5	83.0	12.6	31.9	65.7	41.8	11.6	88.1	5.0	11.7	78.4
	15–24			55–64			15–24			55–64		
1983–1987	4.2	11.1	79.4	7.4	33.0	77.6	2.8	6.7	97.6	5.4	20.5	103.7
1988–1992	4.0	8.9	89.7	8.8	28.6	80.5	3.6	6.7	127.4	6.8	19.8	110.9
1993–1997	4.4	10.0	88.2	10.2	28.7	71.2	2.4	5.0	95.6	4.4	12.3	77.4
1998–2002	7.8	17.9	113.6	23.2	53.3	83.9	3.8	8.1	115.7	5.4	13.4	71.3
2003–2007	7.6	19.3	110.6	23.2	47.9	83.1	3.8	9.6	106.3	6.8	13.7	81.7
2008–2012	7.2	20.5	96.7	18.6	40.2	82.7	2.8	8.4	86.1	8.4	15.4	103.6
	25–34			65–74			25–34			65–74		
1983–1987	7.8	22.5	87.8	4.0	35.4	80.9	4.2	10.8	100.7	5.4	32.5	104.0
1988–1992	4.4	12.7	66.2	4.0	27.7	78.7	2.2	6.3	72.9	5.2	25.8	99.0
1993–1997	8.2	18.3	93.8	5.4	25.9	81.3	3.2	7.1	85.6	3.4	14.5	74.6
1998–2002	11.0	22.8	84.6	10.8	38.7	85.1	6.2	11.5	99.4	7.4	22.4	106.8
2003–2007	13.4	29.4	94.9	11.2	33.3	77.6	9.2	17.0	130.4	5.8	15.9	85.2
2008–2012	12.2	32.0	98.1	15.8	38.6	97.8	6.8	15.7	109.3	8.2	17.9	100.8
	35–44			>74 y/o			35–44			>74 y/o		
1983–1987	10.8	23.6	74.1	5.2	79.7	101.8	5.0	10.3	87.6	4.4	46.0	79.7
1988–1992	9.0	19.7	83.1	3.6	46.0	71.3	4.4	9.1	93.8	6.4	44.0	91.9
1993–1997	8.8	20.9	85.1	3.6	37.2	71.5	4.4	9.3	109.1	4.8	26.5	77.0
1998–2002	13.2	30.0	85.0	4.8	37.6	71.3	4.0	8.9	86.4	7.2	29.6	95.5
2003–2007	16.8	34.8	86.8	7.6	39.3	87.2	8.0	14.6	115.7	4.2	14.0	62.3
2008–2012	15.8	30.7	82.5	9.0	35.0	84.3	5.2	10.2	75.1	5.4	14.0	72.9

Secondary Medical Zone ID: 214

	Male						Female					
	Suicide			Suicide			Suicide			Suicide		
	Num per year	Rate /100,000	×100	Num per year	Rate /100,000	×100	Num per year	Rate /100,000	×100	Num per year	Rate /100,000	×100
	Total (>10 y/o)			45–54			Total (>10 y/o)			45–54		
1983–1987	72.4	30.6	92.1	20.8	50.1	99.1	37.8	14.6	95.8	5.4	14.6	84.6
1988–1992	67.0	24.6	100.4	16.4	33.1	96.2	37.8	12.5	98.2	7.6	15.5	106.0
1993–1997	71.0	23.3	91.5	17.2	30.6	83.6	32.0	9.5	86.6	6.0	11.0	85.3
1998–2002	118.8	35.7	99.3	29.8	54.6	97.6	43.8	11.8	94.4	8.6	15.3	101.4
2003–2007	114.8	34.4	94.7	22.0	49.4	88.3	47.4	13.0	102.1	7.0	14.6	106.6
2008–2012	108.8	33.3	96.2	18.2	45.0	92.5	45.0	12.3	96.9	6.6	15.2	101.8
	15–24			55–64			15–24			55–64		
1983–1987	5.8	13.6	97.6	8.6	34.7	81.6	2.8	6.4	94.2	4.2	16.3	82.6
1988–1992	7.2	12.9	130.3	11.6	34.3	96.6	1.2	3.1	59.4	5.4	16.3	91.5
1993–1997	6.2	11.8	104.0	15.2	37.6	93.4	3.8	6.5	125.3	5.0	13.2	82.7
1998–2002	8.4	18.0	113.8	28.8	60.2	94.8	2.0	5.3	74.8	9.2	19.1	101.6
2003–2007	7.0	17.9	102.5	26.0	48.9	84.8	3.6	9.3	103.0	9.2	16.6	98.9
2008–2012	8.0	22.2	104.8	19.4	39.1	80.5	1.8	6.5	67.4	9.0	16.0	107.0
	25–34			65–74			25–34			65–74		
1983–1987	9.4	23.2	90.9	5.0	38.0	86.8	4.0	9.6	89.2	6.0	31.4	100.5
1988–1992	9.0	21.7	113.7	6.0	35.8	101.5	4.8	10.5	121.2	5.0	22.9	88.0
1993–1997	7.2	15.8	80.7	6.6	29.0	90.8	3.8	7.9	95.2	4.2	15.9	81.6
1998–2002	14.8	27.3	101.2	12.4	42.5	93.6	6.6	11.9	103.0	5.4	17.4	82.8
2003–2007	17.4	33.9	109.3	16.8	47.2	109.9	6.4	12.7	97.3	8.0	20.6	110.4
2008–2012	15.0	34.4	105.5	17.0	40.2	101.9	5.8	13.9	97.1	7.8	17.6	99.0
	35–44			>74 y/o			35–44			>74 y/o		
1983–1987	17.0	29.4	92.5	5.6	79.3	101.3	8.6	14.0	118.7	6.8	59.1	102.4
1988–1992	10.6	20.0	84.4	6.2	66.4	102.9	6.4	11.0	113.8	7.4	46.2	96.6
1993–1997	12.0	25.3	103.1	5.8	52.3	100.5	2.8	6.6	77.1	6.2	30.3	88.0
1998–2002	15.6	35.0	99.3	8.6	60.0	113.8	3.2	8.0	76.9	8.8	33.8	109.0
2003–2007	15.0	32.5	81.0	9.8	50.3	111.6	6.8	13.7	108.6	5.8	18.5	82.3
2008–2012	19.0	36.1	97.0	11.8	46.9	113.2	5.6	11.4	84.2	8.0	20.4	105.9

Secondary Medical Zone ID: 215

	Male						Female					
	Suicide			Suicide			Suicide			Suicide		
	Num per year	Rate /100,000	×100	Num per year	Rate /100,000	×100	Num per year	Rate /100,000	×100	Num per year	Rate /100,000	×100
	Total (>10 y/o)			45–54			Total (>10 y/o)			45–54		
1983–1987	40.0	33.9	105.3	10.0	52.9	104.6	25.8	17.3	119.6	4.2	20.5	118.8
1988–1992	31.4	24.9	101.1	4.8	26.4	76.8	26.2	15.1	127.8	3.8	16.8	115.2
1993–1997	39.4	27.4	114.8	8.6	37.4	102.2	21.8	11.5	118.7	2.6	12.1	94.0
1998–2002	47.0	34.6	94.2	9.2	44.4	79.4	26.0	13.9	119.0	2.8	13.7	90.9
2003–2007	51.8	37.8	103.4	11.2	58.5	104.6	20.0	12.7	100.5	3.4	15.7	114.3
2008–2012	53.2	38.1	113.9	9.4	54.0	111.1	18.4	11.7	96.4	0.8	9.7	64.7
	15–24			55–64			15–24			55–64		
1983–1987	2.2	13.3	95.2	7.4	47.9	112.7	0.8	5.6	82.5	3.4	20.3	102.8
1988–1992	2.6	11.6	116.7	8.8	46.8	131.7	1.8	6.9	131.2	3.8	19.9	111.4
1993–1997	2.4	11.8	104.1	9.4	48.4	120.3	0.4	3.6	69.1	3.8	18.8	118.2
1998–2002	2.8	15.9	100.9	10.8	57.0	89.7	1.4	7.4	105.1	7.2	28.2	149.8
2003–2007	3.2	19.3	110.7	11.2	52.8	91.4	1.6	9.8	109.4	3.6	16.5	98.3
2008–2012	2.4	19.6	92.2	9.6	45.6	93.9	1.4	10.1	104.3	2.6	13.3	89.0
	25–34			65–74			25–34			65–74		
1983–1987	4.6	26.2	102.5	3.6	42.8	97.7	2.2	11.4	106.3	4.4	35.2	112.6
1988–1992	4.8	26.3	137.7	2.8	30.4	86.4	1.4	8.5	98.0	5.2	34.6	132.6
1993–1997	3.2	19.2	98.3	5.2	37.8	118.7	2.0	9.8	117.6	4.0	23.6	120.9
1998–2002	4.6	25.8	95.4	6.0	41.5	91.2	3.4	14.7	127.1	3.4	20.5	97.5
2003–2007	6.2	33.7	108.6	6.8	42.6	99.4	1.0	9.3	71.5	2.8	17.2	92.2
2008–2012	7.6	42.3	130.0	8.6	46.5	117.6	1.0	10.9	76.3	4.0	19.8	111.1
	35–44			>74 y/o			35–44			>74 y/o		
1983–1987	9.4	38.7	121.5	2.8	59.6	76.1	4.0	14.9	126.1	6.6	77.2	133.9
1988–1992	4.8	22.2	93.8	2.8	48.9	75.9	4.0	13.8	143.2	6.2	55.4	115.8
1993–1997	6.2	30.1	122.9	4.2	57.2	109.8	0.4	5.1	59.7	8.4	58.9	171.0
1998–2002	7.8	42.3	120.1	5.6	61.8	117.1	1.6	9.7	93.5	6.2	37.3	120.0
2003–2007	7.4	42.7	106.5	5.4	47.5	105.4	3.2	15.4	121.8	4.4	22.6	100.6
2008–2012	9.0	44.4	119.4	6.6	46.5	112.0	2.0	12.3	90.7	6.6	27.2	141.6

Secondary Medical Zone ID: 216

	Male						Female					
	Suicide			Suicide			Suicide			Suicide		
	Num per year	Rate /100,000	×100	Num per year	Rate /100,000	×100	Num per year	Rate /100,000	×100	Num per year	Rate /100,000	×100
	Total (>10 y/o)			45–54			Total (>10 y/o)			45–54		
1983–1987	81.0	36.8	116.0	26.0	70.6	139.7	36.0	14.7	96.3	5.4	15.7	90.8
1988–1992	52.8	23.2	93.9	16.6	41.6	121.0	31.8	11.9	91.6	6.4	15.6	106.9
1993–1997	62.6	24.7	99.8	14.4	33.6	92.0	30.2	10.2	94.0	5.0	11.7	90.8
1998–2002	112.0	41.0	117.5	26.6	62.4	111.5	36.4	12.3	93.7	6.6	15.4	102.0
2003–2007	99.4	37.5	103.1	20.2	56.5	101.1	39.0	13.0	101.8	3.8	11.5	84.1
2008–2012	97.6	36.6	108.1	16.4	48.9	100.6	37.4	12.6	98.5	6.0	16.6	111.2
	15–24			55–64			15–24			55–64		
1983–1987	5.0	14.1	101.1	11.6	45.8	107.8	2.6	6.8	99.2	5.2	18.6	94.5
1988–1992	4.2	10.2	103.5	8.0	27.9	78.5	1.4	3.9	74.5	5.6	17.5	98.0
1993–1997	5.0	12.1	106.8	15.2	44.8	111.2	1.0	3.4	64.7	6.4	17.6	110.7
1998–2002	8.0	20.8	131.8	30.0	79.8	125.6	1.6	5.5	78.3	7.6	19.4	102.9
2003–2007	7.6	22.0	125.9	22.4	55.1	95.4	4.0	11.7	130.4	7.6	17.3	103.3
2008–2012	8.0	25.7	121.3	19.4	48.6	100.0	3.0	10.4	107.5	6.8	15.8	105.9
	25–34			65–74			25–34			65–74		
1983–1987	10.4	28.6	111.9	6.2	43.4	99.0	3.4	9.7	90.5	4.6	24.4	78.2
1988–1992	4.4	14.2	74.1	4.8	31.1	88.1	3.8	10.0	115.5	5.0	22.5	86.2
1993–1997	5.2	15.1	77.3	7.8	36.2	113.5	3.0	7.9	94.5	4.0	15.9	81.7
1998–2002	12.2	28.8	106.6	14.0	52.9	116.3	4.2	10.4	89.6	5.8	19.7	93.7
2003–2007	15.0	36.4	117.4	12.4	43.2	100.6	5.6	13.7	105.0	6.0	18.3	98.5
2008–2012	11.2	33.2	101.9	14.4	44.0	111.3	3.8	12.4	86.5	6.0	17.0	95.2
	35–44			>74 y/o			35–44			>74 y/o		
1983–1987	15.4	33.6	105.7	6.2	80.9	103.3	5.0	11.1	93.8	9.6	74.3	128.8
1988–1992	10.2	24.0	101.1	4.4	49.3	76.5	4.0	9.4	97.7	5.6	35.2	73.5
1993–1997	8.8	23.9	97.4	5.8	53.1	101.9	3.2	8.6	101.0	7.6	36.4	105.7
1998–2002	15.0	41.2	117.1	6.2	48.3	91.6	4.8	12.4	119.8	5.2	21.6	69.5
2003–2007	14.0	38.1	95.0	7.4	43.1	95.7	4.4	11.8	93.8	7.0	22.6	100.6
2008–2012	18.4	42.9	115.3	9.4	43.1	103.9	3.4	9.9	73.0	8.4	22.5	116.8

Secondary Medical Zone ID: 217

	Male						Female					
	Suicide			Suicide			Suicide			Suicide		
	Num per year	Rate /100,000	×100	Num per year	Rate /100,000	×100	Num per year	Rate /100,000	×100	Num per year	Rate /100,000	×100
	Total (>10 y/o)			45–54			Total (>10 y/o)			45–54		
1983–1987	33.8	30.1	88.6	8.2	45.1	89.3	22.6	15.5	102.0	3.2	17.1	99.0
1988–1992	29.2	24.0	95.1	5.0	27.7	80.7	19.0	12.2	94.8	2.6	13.8	94.6
1993–1997	30.6	23.6	93.2	7.4	34.0	92.9	13.0	9.4	77.2	2.8	12.7	98.4
1998–2002	45.0	34.5	93.4	12.4	56.1	100.3	19.2	12.2	93.5	2.0	11.9	78.8
2003–2007	51.2	37.3	106.3	10.8	58.7	104.9	20.6	13.3	104.0	2.6	13.8	100.6
2008–2012	42.2	34.3	97.1	8.2	51.4	105.8	17.8	12.5	95.9	2.4	14.9	100.0
	15–24			55–64			15–24			55–64		
1983–1987	3.0	16.2	116.0	4.6	34.0	80.0	1.2	6.6	97.2	3.2	18.7	95.0
1988–1992	2.4	11.3	113.9	4.8	29.8	83.8	1.2	5.6	106.6	2.6	15.6	87.3
1993–1997	1.8	10.3	91.3	5.0	31.8	78.9	0.6	4.2	79.9	2.0	12.7	80.1
1998–2002	2.6	15.6	98.4	11.6	62.4	98.2	1.0	6.5	93.0	3.2	17.5	92.8
2003–2007	2.6	17.9	102.4	13.4	61.9	107.3	3.0	14.9	165.7	2.8	14.7	87.5
2008–2012	2.8	22.0	103.7	9.0	44.0	90.6	1.0	9.0	93.0	2.4	12.8	86.2
	25–34			65–74			25–34			65–74		
1983–1987	3.6	21.7	84.8	2.2	30.3	69.3	3.0	13.7	127.4	2.8	24.3	77.7
1988–1992	4.2	24.2	126.7	3.6	35.1	99.6	1.2	8.0	92.6	3.0	22.0	84.3
1993–1997	3.4	20.9	106.8	3.6	29.0	90.9	0.4	5.7	68.1	2.0	14.3	73.4
1998–2002	3.4	22.6	83.8	6.0	42.0	92.2	1.8	11.0	94.8	3.6	20.7	98.5
2003–2007	5.4	31.5	101.5	8.4	51.4	119.8	2.6	14.2	109.3	3.4	19.0	101.9
2008–2012	6.4	38.7	118.8	6.4	39.2	99.2	1.6	13.0	90.6	2.4	15.3	85.7
	35–44			>74 y/o			35–44			>74 y/o		
1983–1987	7.2	31.0	97.5	4.8	85.3	109.0	2.6	11.5	97.7	6.6	71.3	123.5
1988–1992	5.4	24.0	101.5	3.6	56.9	88.3	2.2	9.8	101.1	6.0	51.4	107.3
1993–1997	3.6	20.7	84.6	5.4	68.5	131.6	2.6	11.2	131.9	2.6	20.9	60.7
1998–2002	5.6	34.8	98.8	3.2	41.7	79.1	2.2	11.8	113.8	5.2	31.0	99.8
2003–2007	5.4	35.8	89.3	5.0	46.0	102.2	1.8	11.9	94.2	4.4	22.2	98.7
2008–2012	4.8	31.8	85.4	4.6	37.2	89.6	2.8	14.7	108.7	5.2	22.1	115.1

Secondary Medical Zone ID: 218

	Male						Female					
	Suicide			Suicide			Suicide			Suicide		
	Num per year	Rate /100,000	×100	Num per year	Rate /100,000	×100	Num per year	Rate /100,000	×100	Num per year	Rate /100,000	×100
	Total (>10 y/o)			45-54			Total (>10 y/o)			45-54		
1983-1987	35.0	35.4	114.5	8.4	58.1	114.8	17.2	14.6	95.9	2.4	16.8	97.3
1988-1992	20.8	22.6	89.7	4.0	32.0	93.1	18.0	13.2	108.5	1.6	13.4	91.9
1993-1997	27.2	26.9	110.0	4.4	33.1	90.4	13.0	10.1	95.1	1.0	10.4	80.7
1998-2002	34.8	36.1	100.6	9.0	59.9	107.0	12.0	11.0	83.1	2.0	14.7	97.4
2003-2007	38.8	40.6	114.5	7.8	58.8	105.2	14.4	12.7	102.7	1.0	11.3	82.6
2008-2012	34.6	38.3	113.7	7.4	59.3	122.0	14.4	14.1	107.2	1.4	13.9	92.9
	15-24			55-64			15-24			55-64		
1983-1987	0.4	8.9	64.0	6.2	44.6	105.1	0.8	7.1	103.6	2.4	17.2	87.3
1988-1992	1.0	9.8	99.1	5.2	35.9	101.2	1.0	6.7	126.3	4.0	22.0	123.0
1993-1997	1.8	13.3	117.7	6.0	43.8	108.7	0.6	5.4	103.8	1.8	14.0	88.3
1998-2002	1.8	17.0	107.4	7.4	60.9	95.9	0.0	5.0	70.5	1.0	13.8	73.2
2003-2007	2.8	23.6	135.2	8.0	58.7	101.6	0.8	9.5	105.1	1.2	13.3	79.7
2008-2012	1.8	23.4	110.4	7.4	50.8	104.6	1.4	13.5	138.7	2.0	14.6	98.1
	25-34			65-74			25-34			65-74		
1983-1987	5.8	37.5	146.6	4.2	48.8	111.5	0.8	8.7	80.8	3.6	30.9	99.0
1988-1992	1.6	16.5	86.5	3.2	34.5	98.0	0.8	8.1	93.3	2.2	19.5	74.9
1993-1997	2.2	20.5	105.1	4.6	37.1	116.3	0.4	6.8	81.5	3.8	24.1	123.7
1998-2002	2.6	25.8	95.4	5.2	42.8	94.1	0.2	8.3	71.8	3.8	23.9	113.9
2003-2007	3.2	31.2	100.8	6.2	50.2	116.9	1.4	13.6	104.9	2.4	18.1	97.3
2008-2012	3.0	33.4	102.7	4.8	42.6	107.7	1.6	16.1	112.7	2.6	19.2	107.6
	35-44			>74 y/o			35-44			>74 y/o		
1983-1987	5.0	33.5	105.3	5.0	87.2	111.3	0.6	8.5	71.8	6.6	73.5	127.4
1988-1992	2.0	17.3	72.9	3.8	61.0	94.6	1.6	10.3	106.4	6.8	61.1	127.6
1993-1997	4.8	31.9	130.0	3.4	49.7	95.5	0.8	7.5	88.6	4.6	35.9	104.3
1998-2002	4.2	35.7	101.3	4.2	51.1	96.9	0.8	8.9	86.5	4.2	28.3	91.0
2003-2007	6.0	51.6	128.7	4.8	46.2	102.5	2.4	16.3	129.2	5.2	28.0	124.9
2008-2012	5.2	43.1	115.9	5.0	42.1	101.5	2.0	15.6	115.3	3.4	17.8	92.7

Secondary Medical Zone ID: 219

	Male						Female					
	Suicide			Suicide			Suicide			Suicide		
	Num per year	Rate /100,000	×100	Num per year	Rate /100,000	×100	Num per year	Rate /100,000	×100	Num per year	Rate /100,000	×100
	Total (>10 y/o)			45-54			Total (>10 y/o)			45-54		
1983-1987	19.2	35.0	114.0	3.8	51.7	102.3	11.4	16.0	109.4	1.4	17.3	100.5
1988-1992	16.2	27.0	119.1	3.2	40.2	117.1	10.8	13.5	112.8	1.6	16.5	113.2
1993-1997	14.8	25.7	105.9	2.0	31.0	84.8	8.4	11.2	104.0	1.2	13.5	104.6
1998-2002	23.6	37.6	114.2	5.6	60.9	108.8	12.4	13.8	123.0	1.2	14.8	98.6
2003-2007	24.0	40.9	117.9	7.4	82.4	147.3	8.2	12.3	99.2	1.2	14.3	104.0
2008-2012	22.0	37.5	116.7	5.2	63.4	130.5	9.4	13.3	109.9	1.2	15.6	104.8
	15-24			55-64			15-24			55-64		
1983-1987	1.2	16.8	120.2	3.0	40.5	95.2	0.4	6.9	101.1	1.8	19.9	101.1
1988-1992	0.8	10.7	108.3	3.2	37.8	106.6	0.4	5.5	105.2	2.0	19.7	110.1
1993-1997	0.6	10.9	96.0	5.4	57.0	141.7	0.2	4.7	90.5	2.0	19.3	121.3
1998-2002	1.2	16.9	107.3	4.8	65.6	103.2	0.2	6.2	88.4	2.0	21.0	111.6
2003-2007	0.6	15.8	90.7	5.0	59.6	103.3	0.0	6.6	73.4	0.8	14.6	87.3
2008-2012	0.8	20.0	94.5	4.8	51.7	106.4	0.4	9.4	97.3	0.6	12.6	84.7
	25-34			65-74			25-34			65-74		
1983-1987	1.2	22.5	88.2	2.8	52.2	119.2	0.6	10.3	95.4	1.8	29.2	93.4
1988-1992	1.6	22.8	119.2	2.4	39.7	112.8	0.6	9.0	103.7	1.8	25.1	96.2
1993-1997	0.6	16.4	83.9	1.4	25.5	80.0	0.6	8.7	104.6	1.6	19.2	98.4
1998-2002	1.6	26.7	99.0	5.4	60.8	133.8	0.6	11.1	95.8	3.4	29.9	142.4
2003-2007	2.6	35.6	114.8	3.8	50.4	117.6	0.4	11.4	87.4	1.4	18.3	98.0
2008-2012	1.8	32.6	100.2	2.8	41.1	104.1	1.2	16.4	114.3	2.2	21.7	122.2
	35-44			>74 y/o			35-44			>74 y/o		
1983-1987	4.2	43.3	136.0	3.0	88.5	112.9	1.8	15.2	129.0	3.6	69.9	121.2
1988-1992	2.0	24.1	101.8	3.0	77.9	120.7	0.6	9.1	94.4	3.8	59.3	124.0
1993-1997	2.0	25.5	103.9	2.6	62.0	119.0	1.2	10.7	126.4	1.6	25.7	74.6
1998-2002	2.2	34.0	96.7	2.8	57.4	108.8	1.4	13.2	127.2	3.6	38.9	125.4
2003-2007	2.6	41.5	103.7	2.0	38.6	85.8	1.4	15.0	118.4	3.0	27.3	121.7
2008-2012	2.8	40.1	107.7	3.6	48.8	117.7	0.6	12.6	92.9	3.2	24.7	128.7

Secondary Medical Zone ID: 220

	Male						Female					
	Suicide			Suicide			Suicide			Suicide		
	Num per year	Rate /100,000	×100	Num per year	Rate /100,000	×100	Num per year	Rate /100,000	×100	Num per year	Rate /100,000	×100
	Total (>10 y/o)			45-54			Total (>10 y/o)			45-54		
1983-1987	28.6	36.2	115.8	6.8	58.9	116.5	17.0	16.4	112.8	1.6	15.8	91.7
1988-1992	23.0	27.4	117.3	5.0	42.8	124.7	14.8	13.4	110.7	2.2	16.7	114.4
1993-1997	20.8	26.2	105.2	4.4	36.9	101.0	11.4	11.1	103.3	1.6	13.3	102.6
1998-2002	30.0	37.6	106.7	5.8	50.6	90.5	15.0	13.4	115.3	2.0	15.8	104.9
2003-2007	30.8	39.2	113.1	5.8	56.7	101.5	14.6	13.9	120.7	2.2	16.6	120.8
2008-2012	28.0	37.3	114.1	5.6	58.5	120.4	13.4	13.8	116.6	1.2	14.5	97.5
	15-24			55-64			15-24			55-64		
1983-1987	1.0	13.0	93.2	5.8	50.6	119.0	0.6	6.8	99.2	4.8	30.0	152.0
1988-1992	1.6	12.4	124.8	4.8	40.1	112.9	0.0	3.7	70.2	2.6	19.5	109.2
1993-1997	1.0	11.4	100.6	3.8	38.1	94.7	0.6	5.7	109.2	2.8	20.2	127.1
1998-2002	2.4	21.0	132.9	7.0	68.2	107.3	0.4	6.5	92.7	1.6	17.4	92.3
2003-2007	0.8	15.9	91.3	7.0	60.8	105.4	0.4	8.1	89.7	3.0	20.2	120.7
2008-2012	1.6	23.7	111.5	5.6	48.0	98.8	0.8	11.1	114.1	2.0	15.6	104.8
	25-34			65-74			25-34			65-74		
1983-1987	4.2	34.5	135.0	2.8	42.1	96.2	2.0	14.5	134.3	2.4	27.4	87.5
1988-1992	1.8	19.8	103.8	2.6	35.2	100.0	1.0	9.5	109.2	2.2	23.1	88.7
1993-1997	2.2	22.3	114.3	2.4	28.6	89.7	0.6	7.9	94.7	1.6	16.3	83.8
1998-2002	2.0	25.0	92.7	4.6	46.3	101.9	1.2	12.2	105.5	2.8	22.9	109.2
2003-2007	3.0	33.5	108.0	6.0	56.6	131.8	1.0	12.8	98.3	3.6	25.9	139.3
2008-2012	2.6	34.7	106.4	4.8	47.7	120.6	0.6	12.9	89.8	1.4	16.4	92.3
	35-44			>74 y/o			35-44			>74 y/o		
1983-1987	5.0	37.8	118.8	3.0	68.3	87.2	1.6	12.5	105.6	4.0	56.6	98.1
1988-1992	3.8	27.9	117.9	3.4	64.2	99.5	1.4	10.4	107.9	5.4	59.8	125.0
1993-1997	3.6	30.1	123.0	3.4	58.1	111.5	0.8	8.2	96.8	3.4	34.7	100.9
1998-2002	4.8	45.5	129.3	3.4	52.2	99.0	1.4	12.1	116.9	5.6	44.8	144.3
2003-2007	4.8	51.1	127.5	3.4	43.1	95.6	1.4	13.9	109.7	3.0	22.2	98.9
2008-2012	4.2	42.5	114.1	3.4	38.1	91.8	2.2	17.2	126.8	5.2	29.5	153.3

Secondary Medical Zone ID: 221

	Male						Female					
	Suicide			Suicide			Suicide			Suicide		
	Num per year	Rate /100,000	×100	Num per year	Rate /100,000	×100	Num per year	Rate /100,000	×100	Num per year	Rate /100,000	×100
	Total (>10 y/o)			45–54			Total (>10 y/o)			45–54		
1983–1987	31.2	27.2	77.6	11.4	51.9	102.6	21.0	14.9	96.2	3.0	15.2	88.5
1988–1992	24.6	19.7	72.1	6.0	26.0	75.7	18.8	11.7	88.7	4.0	15.0	102.7
1993–1997	30.4	20.7	77.7	7.8	29.7	81.2	18.6	10.1	92.0	3.6	12.2	94.5
1998–2002	47.8	30.2	79.8	14.0	52.5	93.9	22.8	11.9	90.5	3.8	13.6	90.2
2003–2007	46.6	29.7	77.0	7.2	35.4	63.2	21.4	11.5	86.9	2.6	11.6	84.9
2008–2012	40.8	28.6	75.1	6.0	33.8	69.6	19.2	10.8	80.7	2.6	12.6	84.4
	15–24			55–64			15–24			55–64		
1983–1987	1.4	8.7	62.2	2.8	26.7	62.7	2.6	8.8	129.3	2.6	17.9	90.9
1988–1992	2.0	8.5	85.6	4.8	28.1	79.1	1.6	5.3	100.6	1.6	12.2	68.4
1993–1997	3.2	11.5	101.5	7.8	36.8	91.3	2.6	7.2	137.9	3.8	16.7	105.1
1998–2002	3.0	13.6	85.9	10.8	47.9	75.4	1.8	6.9	98.3	4.6	18.1	96.1
2003–2007	4.2	18.7	107.4	11.0	44.0	76.2	1.6	7.8	87.0	3.8	14.2	85.0
2008–2012	3.2	18.8	88.6	9.4	39.7	81.7	1.2	7.6	77.9	4.4	14.9	99.6
	25–34			65–74			25–34			65–74		
1983–1987	4.4	22.0	85.9	2.6	38.5	87.9	1.0	6.7	62.3	3.8	34.7	111.1
1988–1992	3.0	15.5	81.0	2.8	31.8	90.1	1.4	7.1	82.2	2.4	21.1	81.1
1993–1997	3.0	15.0	76.9	2.8	24.3	76.2	1.4	6.7	80.6	1.6	13.1	67.3
1998–2002	5.0	22.4	82.8	5.8	37.7	82.9	4.0	13.2	114.2	3.6	19.9	94.6
2003–2007	6.2	28.0	90.5	8.4	43.2	100.8	1.8	9.5	73.0	4.0	18.7	100.5
2008–2012	6.4	33.3	102.2	6.2	31.6	80.0	2.6	13.4	93.3	3.2	14.8	83.2
	35–44			>74 y/o			35–44			>74 y/o		
1983–1987	6.2	23.8	74.7	2.2	60.1	76.7	3.2	11.0	92.9	4.8	67.4	116.8
1988–1992	3.6	15.7	66.0	2.0	44.4	68.9	3.4	10.6	110.2	4.4	46.8	97.7
1993–1997	3.4	16.8	68.5	2.2	38.9	74.8	1.8	7.6	89.8	3.8	32.5	94.5
1998–2002	5.4	26.7	75.9	3.8	47.1	89.3	2.0	9.1	88.2	3.0	21.6	69.7
2003–2007	6.0	29.1	72.6	3.2	31.9	70.9	4.2	14.9	118.2	3.4	18.3	81.4
2008–2012	5.8	28.7	77.0	3.6	28.3	68.2	1.4	8.8	65.1	3.8	16.5	85.8

Secondary Medical Zone ID: 222

	Male						Female					
	Suicide			Suicide			Suicide			Suicide		
	Num per year	Rate /100,000	×100	Num per year	Rate /100,000	×100	Num per year	Rate /100,000	×100	Num per year	Rate /100,000	×100
	Total (>10 y/o)			45–54			Total (>10 y/o)			45–54		
1983–1987	26.8	30.6	90.5	6.0	43.4	85.8	15.8	14.9	96.7	2.2	16.3	94.3
1988–1992	20.0	22.1	84.0	5.2	33.7	98.1	13.4	12.0	89.8	2.8	16.1	110.4
1993–1997	21.0	21.7	81.0	4.6	30.0	82.0	11.4	9.8	85.4	2.0	12.2	94.3
1998–2002	37.8	34.4	96.4	9.6	56.2	100.4	15.0	12.2	93.0	2.6	15.0	99.8
2003–2007	33.2	33.4	87.0	6.0	44.7	79.9	15.0	12.2	97.0	2.4	14.7	107.5
2008–2012	34.6	34.2	99.4	6.8	52.7	108.5	17.0	14.0	108.2	1.8	14.3	96.0
	15–24			55–64			15–24			55–64		
1983–1987	1.4	10.6	76.1	5.2	44.1	103.9	0.6	5.5	80.5	3.0	21.5	109.0
1988–1992	1.2	8.1	82.2	3.6	29.6	83.3	0.8	4.8	90.9	2.2	16.8	94.1
1993–1997	1.4	9.3	82.2	4.8	36.0	89.5	0.8	4.7	90.6	1.0	11.0	69.4
1998–2002	2.4	15.1	95.5	9.8	64.8	101.9	1.4	7.6	108.7	3.4	20.3	108.0
2003–2007	2.4	17.0	97.4	6.8	45.5	78.8	0.8	7.3	81.4	2.4	15.5	92.4
2008–2012	3.0	22.4	105.6	5.4	38.8	79.9	1.6	11.2	115.2	2.6	15.0	100.8
	25–34			65–74			25–34			65–74		
1983–1987	4.8	30.2	118.2	2.8	42.8	97.8	2.4	13.7	127.2	3.4	34.2	109.6
1988–1992	2.6	19.1	100.1	1.6	26.3	74.7	1.0	8.1	93.1	2.6	24.6	94.4
1993–1997	1.6	14.5	74.1	3.0	30.6	95.8	0.6	6.5	78.2	2.8	21.5	110.1
1998–2002	2.8	22.2	82.2	5.8	48.3	106.3	2.2	12.8	110.3	2.4	19.4	92.1
2003–2007	7.0	42.5	137.1	4.0	35.8	83.4	1.8	12.8	98.5	2.0	16.5	88.6
2008–2012	3.6	31.9	97.9	5.8	42.4	107.4	2.2	15.9	110.9	2.4	17.3	97.4
	35–44			>74 y/o			35–44			>74 y/o		
1983–1987	4.0	25.8	80.9	2.4	65.6	83.8	1.6	10.5	88.8	2.6	44.1	76.4
1988–1992	3.6	22.1	93.4	2.2	51.6	80.0	1.8	10.0	103.7	2.2	31.1	65.1
1993–1997	3.0	21.3	87.0	2.6	48.8	93.8	1.6	9.4	110.6	2.6	27.7	80.6
1998–2002	3.8	30.5	86.7	3.6	53.3	101.2	1.0	8.9	86.1	2.0	19.3	62.2
2003–2007	4.0	33.6	83.7	3.0	38.1	84.7	1.6	12.3	97.3	4.0	26.2	116.9
2008–2012	4.8	36.7	98.7	5.2	47.5	114.5	3.2	17.5	128.7	3.2	19.2	100.0

Secondary Medical Zone ID: 223

	Male						Female					
	Suicide			Suicide			Suicide			Suicide		
	Num per year	Rate /100,000	×100	Num per year	Rate /100,000	×100	Num per year	Rate /100,000	×100	Num per year	Rate /100,000	×100
	Total (>10 y/o)			45–54			Total (>10 y/o)			45–54		
1983–1987	28.6	27.4	77.1	7.2	38.0	75.1	17.0	14.3	88.8	2.6	15.0	87.1
1988–1992	28.6	21.9	86.5	7.2	29.2	85.1	15.4	11.1	80.5	2.6	12.2	83.6
1993–1997	26.8	19.7	71.9	6.2	24.9	68.1	15.4	9.4	82.5	4.2	13.4	103.4
1998–2002	50.4	31.8	85.9	11.6	46.0	82.1	21.4	11.8	90.0	3.8	13.6	90.3
2003–2007	42.2	29.2	72.7	7.6	38.6	69.0	20.8	11.7	89.1	2.8	12.4	90.5
2008–2012	39.8	28.7	75.6	6.4	37.4	76.9	20.8	11.9	89.5	3.6	15.9	106.7
	15–24			55–64			15–24			55–64		
1983–1987	1.2	8.4	60.2	5.6	42.8	100.6	1.4	6.6	97.0	1.8	15.9	80.4
1988–1992	2.6	9.8	98.7	6.2	35.8	100.7	1.0	4.4	83.0	2.4	15.4	86.2
1993–1997	1.6	8.1	71.5	7.4	36.5	90.7	1.0	4.3	83.0	2.0	11.9	75.1
1998–2002	4.2	17.0	107.8	13.2	54.2	85.2	1.6	6.9	98.1	4.0	17.0	90.5
2003–2007	2.4	14.5	83.3	10.2	40.1	69.6	2.2	10.3	114.1	4.6	15.8	94.5
2008–2012	2.6	18.0	85.1	7.6	34.8	71.7	1.0	7.7	79.4	3.4	12.9	86.4
	25–34			65–74			25–34			65–74		
1983–1987	2.4	17.2	67.2	4.2	55.2	126.0	2.2	10.7	99.4	2.8	30.1	96.3
1988–1992	2.8	16.2	84.6	2.0	27.4	77.9	2.2	9.7	111.7	3.0	26.0	99.6
1993–1997	2.4	13.9	71.4	3.6	30.4	95.2	1.8	8.0	96.3	1.6	13.8	71.0
1998–2002	5.2	23.5	87.1	6.6	44.0	96.7	2.8	11.2	96.3	3.8	21.7	103.4
2003–2007	5.8	27.8	89.5	5.4	32.5	75.8	2.4	11.2	86.2	3.4	17.7	95.1
2008–2012	4.0	26.3	80.9	7.4	34.1	86.3	2.2	12.6	88.1	3.8	16.7	93.8
	35–44			>74 y/o			35–44			>74 y/o		
1983–1987	5.8	23.1	72.6	2.0	63.6	81.2	1.8	8.3	70.5	4.4	71.7	124.2
1988–1992	4.8	19.7	83.2	3.0	63.1	97.8	1.6	7.3	75.6	2.6	33.3	69.6
1993–1997	2.2	13.6	55.5	3.4	55.9	107.4	1.4	6.9	80.9	3.4	31.1	90.3
1998–2002	5.4	28.5	80.8	4.0	52.5	99.5	2.0	9.5	92.2	3.4	25.3	81.6
2003–2007	7.2	34.8	86.9	3.4	35.9	79.8	2.6	11.8	93.3	2.6	15.9	71.1
2008–2012	7.8	34.2	91.8	3.8	31.7	76.3	2.8	12.0	88.4	4.0	18.5	96.2

Secondary Medical Zone ID: 224

	Male						Female					
	Suicide			Suicide			Suicide			Suicide		
	Num per year	Rate /100,000	×100	Num per year	Rate /100,000	×100	Num per year	Rate /100,000	×100	Num per year	Rate /100,000	×100
	Total (>10 y/o)			45–54			Total (>10 y/o)			45–54		
1983–1987	31.2	27.5	77.8	8.4	40.6	80.3	18.8	13.9	87.8	2.8	15.0	87.3
1988–1992	25.8	20.4	75.8	6.4	27.0	78.6	20.2	12.3	94.1	4.4	16.2	110.5
1993–1997	26.6	19.4	69.5	7.4	28.5	77.9	20.4	10.8	99.3	3.6	12.3	95.4
1998–2002	50.0	31.3	82.3	12.2	46.3	82.7	20.0	11.0	82.2	3.8	13.7	91.0
2003–2007	49.6	31.1	79.7	12.0	51.4	92.0	22.6	11.8	91.2	3.2	13.0	95.1
2008–2012	50.8	31.6	87.6	10.2	46.9	96.5	18.6	10.8	78.5	1.8	10.4	69.7
	15–24			55–64			15–24			55–64		
1983–1987	1.2	8.0	57.1	5.6	40.6	95.6	1.6	6.8	98.8	2.4	17.0	86.2
1988–1992	1.8	8.2	82.8	3.6	23.7	66.7	1.4	5.1	97.2	3.0	16.8	93.8
1993–1997	2.0	9.2	81.4	5.4	29.0	72.0	1.4	5.1	97.5	2.8	14.1	88.5
1998–2002	3.2	14.6	92.5	13.6	56.3	88.7	1.0	5.5	78.1	4.8	18.8	99.8
2003–2007	3.4	16.9	96.8	11.4	44.3	76.7	1.8	8.6	95.9	4.2	15.2	90.6
2008–2012	3.6	19.9	93.6	8.0	34.9	71.8	1.0	7.0	71.7	2.4	11.0	73.7
	25–34			65–74			25–34			65–74		
1983–1987	4.6	23.5	92.1	2.6	37.0	84.4	2.0	9.8	90.8	4.0	33.3	106.4
1988–1992	3.8	18.7	98.0	3.4	35.4	100.6	1.4	7.3	84.4	4.2	29.7	114.0
1993–1997	3.6	16.6	84.8	2.8	25.0	78.3	3.0	10.2	122.1	1.8	13.9	71.5
1998–2002	6.2	24.9	92.1	4.8	34.4	75.5	3.0	11.1	95.6	2.4	15.9	75.6
2003–2007	5.6	25.1	80.8	7.0	38.1	88.8	3.0	12.1	92.7	4.6	21.0	112.6
2008–2012	5.8	30.0	92.1	8.2	37.4	94.7	2.6	12.9	90.0	3.4	15.4	86.8
	35–44			>74 y/o			35–44			>74 y/o		
1983–1987	5.8	23.0	72.3	3.0	72.1	92.0	2.6	10.0	84.3	3.2	47.0	81.5
1988–1992	4.0	16.9	71.4	2.8	54.3	84.2	2.4	8.9	92.2	3.2	34.0	71.2
1993–1997	2.8	14.7	59.8	2.4	40.9	78.5	2.6	9.5	112.1	5.0	38.8	112.7
1998–2002	7.6	33.3	94.4	2.2	34.1	64.7	1.2	7.1	68.5	3.6	24.6	79.3
2003–2007	7.2	31.3	78.0	3.0	31.9	70.9	2.4	10.5	83.4	3.4	18.6	82.8
2008–2012	9.4	36.0	96.8	5.4	38.8	93.6	3.4	12.6	93.1	4.0	17.8	92.6

Secondary Medical Zone ID: 225

	Male						Female					
	Suicide			Suicide			Suicide			Suicide		
	Num per year	Rate /100,000	×100	Num per year	Rate /100,000	×100	Num per year	Rate /100,000	×100	Num per year	Rate /100,000	×100
	Total (>10 y/o)			45-54			Total (>10 y/o)			45-54		
1983-1987	20.4	37.7	129.3	5.4	64.6	127.8	9.2	15.4	103.8	0.8	15.0	87.3
1988-1992	14.2	26.5	117.3	1.8	31.4	91.4	10.8	14.3	125.4	1.0	14.6	100.1
1993-1997	14.0	26.9	112.9	2.8	38.6	105.4	7.6	11.3	108.5	1.6	15.6	121.2
1998-2002	18.8	37.3	109.2	3.0	48.7	86.9	7.4	12.3	99.4	0.4	12.7	84.1
2003-2007	18.8	39.6	115.0	4.2	63.2	113.1	8.8	13.5	117.1	1.4	15.9	116.4
2008-2012	15.2	35.6	107.3	2.8	51.7	106.3	6.4	13.3	102.5	1.2	16.8	112.3
	15-24			55-64			15-24			55-64		
1983-1987	1.0	15.0	107.8	2.8	41.6	97.9	0.0	5.3	77.3	2.4	24.5	123.9
1988-1992	0.6	10.0	100.6	4.2	47.0	132.3	0.2	4.9	92.5	1.4	17.9	100.1
1993-1997	0.4	10.3	91.1	3.6	46.2	114.9	0.2	4.8	92.8	1.4	17.1	107.3
1998-2002	0.4	13.7	86.5	4.8	71.2	112.1	0.4	7.2	101.9	0.2	14.3	75.8
2003-2007	1.2	19.4	111.1	3.2	53.7	93.0	0.4	9.0	100.1	1.4	17.8	106.3
2008-2012	1.0	22.3	105.3	4.0	52.9	108.9	0.4	10.0	102.9	1.0	14.9	100.2
	25-34			65-74			25-34			65-74		
1983-1987	1.8	26.8	104.7	3.2	61.5	140.5	0.4	9.5	88.5	2.8	40.3	129.0
1988-1992	2.2	27.6	144.5	2.4	43.0	122.1	0.6	9.1	105.0	3.2	39.3	150.7
1993-1997	2.4	28.9	147.8	1.6	29.7	93.0	0.6	8.9	107.4	1.6	21.1	108.0
1998-2002	2.2	32.6	120.7	3.6	51.0	112.2	0.8	12.5	108.1	2.8	28.5	135.9
2003-2007	1.4	31.7	102.3	2.6	43.6	101.5	0.4	12.3	94.4	1.4	19.3	103.5
2008-2012	0.8	30.2	92.7	2.6	43.6	110.5	0.6	14.9	103.8	0.8	16.4	92.0
	35-44			>74 y/o			35-44			>74 y/o		
1983-1987	3.0	37.8	118.9	3.2	108.3	138.3	1.2	13.4	113.1	1.6	46.5	80.7
1988-1992	1.6	23.2	98.0	1.4	56.7	87.9	1.2	11.6	119.7	3.2	61.2	127.8
1993-1997	2.0	27.5	112.3	1.2	44.3	85.1	0.8	9.6	112.8	1.4	27.5	80.0
1998-2002	3.0	44.2	125.5	1.8	49.6	94.1	0.4	9.5	91.7	2.4	34.1	109.8
2003-2007	3.2	53.7	134.0	3.0	54.3	120.6	1.0	14.2	112.5	2.8	30.6	136.4
2008-2012	1.8	38.7	104.1	2.2	40.4	97.3	0.8	14.5	106.7	1.6	18.4	95.9

Secondary Medical Zone ID: 226

	Male						Female					
	Suicide			Suicide			Suicide			Suicide		
	Num per year	Rate /100,000	×100	Num per year	Rate /100,000	×100	Num per year	Rate /100,000	×100	Num per year	Rate /100,000	×100
	Total (>10 y/o)			45-54			Total (>10 y/o)			45-54		
1983-1987	80.2	38.8	125.6	29.4	85.4	168.8	41.2	17.0	114.5	9.4	24.7	143.4
1988-1992	52.6	25.2	104.7	12.8	37.0	107.6	33.0	12.5	100.6	6.4	16.7	114.5
1993-1997	57.4	26.2	107.1	13.6	37.2	101.7	30.2	11.0	103.5	6.8	16.1	124.7
1998-2002	84.4	37.7	108.4	20.8	61.5	109.9	34.6	12.7	101.6	6.4	17.0	112.6
2003-2007	71.2	33.9	94.6	12.0	45.3	80.9	33.0	13.0	103.1	6.2	18.2	132.6
2008-2012	67.8	33.4	99.5	9.6	40.8	84.0	28.0	12.3	92.6	2.6	11.5	77.4
	15-24			55-64			15-24			55-64		
1983-1987	3.2	11.2	80.4	13.8	54.1	127.4	0.6	3.8	55.3	4.2	16.4	83.0
1988-1992	2.2	8.1	81.8	11.6	39.7	111.7	1.2	4.2	80.0	4.4	15.3	85.4
1993-1997	3.6	11.7	103.3	14.2	46.2	114.8	2.2	6.3	121.4	6.4	18.7	117.4
1998-2002	4.2	16.3	103.1	23.8	73.8	116.2	1.2	5.8	82.2	5.6	17.2	91.4
2003-2007	3.2	15.9	90.9	23.0	67.4	116.7	2.0	9.1	101.3	6.6	17.6	105.0
2008-2012	3.2	18.3	86.5	18.2	56.3	115.8	2.2	10.8	111.7	4.2	13.2	88.7
	25-34			65-74			25-34			65-74		
1983-1987	7.0	24.0	94.0	6.6	47.9	109.5	3.6	11.3	104.7	6.6	33.0	105.8
1988-1992	4.0	15.5	81.2	6.0	37.6	106.8	1.4	6.2	71.4	10.0	41.4	158.6
1993-1997	6.0	20.9	107.0	5.4	27.7	86.9	1.8	6.8	81.9	4.8	18.9	96.7
1998-2002	8.6	28.9	107.2	11.8	48.5	106.6	3.8	11.9	103.1	7.0	23.5	112.0
2003-2007	6.6	26.6	85.7	11.8	45.9	106.9	3.4	12.5	96.1	4.8	16.7	89.6
2008-2012	6.4	30.3	93.1	12.0	43.3	109.7	2.6	12.6	88.3	5.2	16.9	95.2
	35-44			>74 y/o			35-44			>74 y/o		
1983-1987	15.8	39.1	123.0	4.4	61.4	78.3	7.2	15.8	133.8	9.6	72.8	126.2
1988-1992	10.2	28.2	118.9	5.6	61.5	95.4	2.2	7.3	75.9	7.4	43.9	91.8
1993-1997	6.8	23.6	96.1	7.8	70.7	135.7	1.4	6.0	70.2	6.8	33.5	97.5
1998-2002	8.6	32.9	93.5	6.2	49.3	93.6	3.0	10.4	100.7	7.6	30.8	99.3
2003-2007	7.8	31.4	78.4	6.6	40.1	89.1	3.2	11.6	92.1	6.8	22.6	100.9
2008-2012	10.0	36.0	96.9	8.2	40.4	97.5	4.8	15.0	110.4	6.4	18.3	95.3

Secondary Medical Zone ID: 227

	Male						Female					
	Suicide			Suicide			Suicide			Suicide		
	Num per year	Rate /100,000	×100	Num per year	Rate /100,000	×100	Num per year	Rate /100,000	×100	Num per year	Rate /100,000	×100
	Total (>10 y/o)			45–54			Total (>10 y/o)			45–54		
1983–1987	12.6	32.9	102.0	3.4	54.7	108.1	7.4	15.3	103.9	1.0	17.2	99.6
1988–1992	11.6	25.4	108.9	1.2	26.5	77.1	7.0	12.8	102.9	1.6	17.1	116.8
1993–1997	13.4	26.1	108.6	2.8	35.7	97.5	8.0	11.6	116.1	1.4	14.2	110.2
1998–2002	18.4	35.3	98.0	3.8	49.1	87.8	6.4	11.6	88.2	0.4	11.8	78.6
2003–2007	19.8	37.0	101.1	5.2	62.9	112.4	7.8	12.1	99.4	0.6	11.8	86.0
2008–2012	17.8	33.9	98.6	3.2	46.4	95.5	9.8	13.4	112.1	0.8	13.3	89.2
	15–24			55–64			15–24			55–64		
1983–1987	0.4	11.9	85.0	2.6	47.4	111.5	0.8	8.4	123.5	1.4	21.6	109.5
1988–1992	1.0	11.4	115.6	2.6	40.7	114.5	0.0	4.1	77.0	0.6	15.4	86.3
1993–1997	1.0	12.2	107.6	3.0	44.6	110.9	0.0	3.9	75.4	1.8	20.1	126.5
1998–2002	1.0	15.3	97.1	5.4	72.7	114.4	0.0	5.4	76.3	1.0	17.4	92.4
2003–2007	1.0	16.8	96.5	3.8	51.7	89.5	0.6	9.2	102.1	2.2	19.6	116.9
2008–2012	1.2	20.8	98.0	4.4	50.2	103.3	0.4	8.9	91.5	2.6	19.2	128.5
	25–34			65–74			25–34			65–74		
1983–1987	1.0	22.4	87.5	1.2	43.0	98.1	1.2	13.2	122.6	1.4	32.2	103.2
1988–1992	1.0	18.7	97.6	1.8	42.1	119.5	0.4	8.0	92.7	1.6	29.9	114.6
1993–1997	1.4	20.2	103.3	2.2	40.1	125.8	0.4	7.5	89.8	1.4	22.1	113.6
1998–2002	2.0	27.0	99.9	2.2	44.2	97.1	0.2	8.9	76.4	1.2	20.5	97.7
2003–2007	2.0	29.7	95.9	2.6	44.9	104.6	0.6	11.3	87.2	1.2	18.7	100.5
2008–2012	2.4	35.1	107.8	2.0	36.5	92.4	1.2	15.6	108.9	1.6	19.5	109.6
	35–44			>74 y/o			35–44			>74 y/o		
1983–1987	2.6	33.0	103.8	1.4	78.4	100.1	1.0	12.2	103.3	0.6	35.2	61.0
1988–1992	2.6	27.9	117.9	1.4	65.3	101.3	0.8	9.8	101.0	2.0	52.0	108.7
1993–1997	2.2	26.4	107.8	0.8	42.8	82.2	0.6	8.2	96.6	2.4	46.9	136.3
1998–2002	2.6	34.3	97.4	1.4	49.6	94.1	1.2	11.7	113.5	2.4	38.9	125.3
2003–2007	3.6	44.2	110.4	1.6	43.0	95.6	0.4	10.1	80.0	2.2	28.7	127.8
2008–2012	1.8	31.5	84.6	2.8	50.9	122.7	1.0	12.9	95.4	2.2	23.8	123.6

Secondary Medical Zone ID: 228

	Male						Female					
	Suicide			Suicide			Suicide			Suicide		
	Num per year	Rate /100,000	×100	Num per year	Rate /100,000	×100	Num per year	Rate /100,000	×100	Num per year	Rate /100,000	×100
	Total (>10 y/o)			45-54			Total (>10 y/o)			45-54		
1983-1987	13.8	33.7	105.5	4.6	63.3	125.2	9.0	16.2	113.1	0.8	15.7	91.2
1988-1992	11.6	25.2	107.3	2.2	33.8	98.5	9.0	13.8	118.7	0.8	13.9	95.3
1993-1997	14.4	26.5	117.3	1.8	30.7	83.9	8.2	11.6	118.5	1.2	13.7	106.0
1998-2002	17.0	36.0	99.3	4.0	53.4	95.4	8.2	12.7	106.0	1.0	14.4	95.4
2003-2007	11.8	31.2	76.0	2.8	47.2	84.4	7.0	12.2	100.2	1.0	13.8	100.8
2008-2012	14.2	32.7	96.2	1.6	39.7	81.8	8.6	13.5	113.2	1.2	16.0	107.1
	15-24			55-64			15-24			55-64		
1983-1987	0.6	12.4	88.6	2.4	43.9	103.3	0.4	6.8	99.6	2.4	26.5	134.1
1988-1992	1.2	11.6	117.6	2.6	39.3	110.6	0.6	6.2	117.4	1.8	20.9	117.0
1993-1997	1.0	11.8	104.6	3.6	48.6	120.8	0.2	4.7	89.6	1.0	15.5	97.8
1998-2002	1.0	15.5	98.3	4.0	62.3	98.1	0.4	6.9	98.1	1.2	18.4	97.8
2003-2007	0.2	13.6	77.7	3.6	53.0	91.9	0.4	8.5	94.6	1.0	15.7	93.9
2008-2012	1.0	21.0	99.0	2.2	40.3	82.9	0.4	9.4	96.9	1.0	14.4	96.5
	25-34			65-74			25-34			65-74		
1983-1987	2.0	30.0	117.5	0.6	32.9	75.2	0.6	10.7	99.5	1.0	27.0	86.3
1988-1992	0.4	14.8	77.3	1.8	40.9	116.1	0.8	9.9	114.4	1.8	30.5	116.7
1993-1997	0.8	18.1	92.7	3.4	51.5	161.5	0.2	7.2	86.5	2.0	26.0	133.3
1998-2002	1.6	27.8	103.1	1.8	40.3	88.5	0.8	12.1	104.4	1.6	22.6	107.5
2003-2007	0.4	22.8	73.4	2.6	45.1	105.2	0.4	11.7	89.8	1.0	17.6	94.3
2008-2012	2.0	36.2	111.3	3.0	44.7	113.1	1.0	15.9	110.9	2.0	21.7	121.8
	35-44			>74 y/o			35-44			>74 y/o		
1983-1987	2.0	30.1	94.7	1.6	81.1	103.6	0.8	11.6	98.3	2.8	79.2	137.2
1988-1992	1.2	20.4	86.2	2.2	81.1	125.7	0.2	7.9	81.9	3.0	66.5	139.1
1993-1997	1.4	22.6	92.1	2.2	69.1	132.6	0.6	8.5	99.7	3.0	53.3	154.9
1998-2002	3.0	42.4	120.3	1.6	52.0	98.6	0.4	9.1	88.1	2.8	42.3	136.4
2003-2007	0.8	27.4	68.5	1.4	39.9	88.6	0.6	12.0	95.2	2.6	31.7	141.1
2008-2012	1.0	31.6	85.0	3.4	56.2	135.4	0.8	13.7	101.3	2.2	23.5	122.5

Secondary Medical Zone ID: 229

	Male						Female					
	Suicide			Suicide			Suicide			Suicide		
	Num per year	Rate /100,000	×100	Num per year	Rate /100,000	×100	Num per year	Rate /100,000	×100	Num per year	Rate /100,000	×100
	Total (>10 y/o)			45–54			Total (>10 y/o)			45–54		
1983–1987	16.4	36.4	123.5	5.0	68.2	134.8	8.0	15.7	105.6	1.6	19.7	114.4
1988–1992	12.0	26.6	116.3	2.8	41.1	119.7	10.0	14.7	131.6	2.0	19.0	130.1
1993–1997	11.6	26.1	109.9	1.0	28.4	77.6	8.0	12.0	123.0	1.2	14.4	111.5
1998–2002	16.2	37.1	108.3	3.0	52.0	92.9	6.6	12.5	100.1	0.6	13.7	90.9
2003–2007	14.4	35.6	101.5	2.8	54.0	96.5	6.4	12.9	103.1	0.8	13.8	100.7
2008–2012	14.0	35.2	107.7	2.0	46.6	95.9	6.0	13.0	102.6	1.2	17.0	113.7
	15–24			55–64			15–24			55–64		
1983–1987	2.0	22.2	159.4	3.2	50.8	119.5	0.2	6.1	89.5	1.2	19.6	99.3
1988–1992	1.0	11.8	118.8	2.2	36.3	102.1	0.0	4.2	79.2	1.2	18.2	101.8
1993–1997	0.6	11.4	100.8	3.0	46.0	114.2	0.6	6.4	123.2	2.0	21.5	135.2
1998–2002	0.2	13.1	83.2	5.2	80.2	126.2	0.2	6.5	93.1	1.4	20.1	106.9
2003–2007	0.0	13.8	79.2	3.4	57.9	100.3	0.0	7.1	78.7	1.0	16.6	99.1
2008–2012	0.8	21.8	102.7	4.2	56.9	117.0	0.2	9.0	92.8	1.0	15.2	101.8
	25–34			65–74			25–34			65–74		
1983–1987	0.6	19.8	77.6	1.6	45.9	104.9	0.2	8.7	80.9	2.0	37.4	119.8
1988–1992	0.8	18.0	94.4	1.2	33.9	96.3	0.6	9.2	106.6	1.8	30.5	116.9
1993–1997	1.0	20.0	102.6	2.2	39.8	124.7	0.4	8.2	98.6	0.8	17.0	87.2
1998–2002	0.8	23.9	88.7	2.0	43.1	94.7	0.6	11.6	100.3	1.4	22.0	104.9
2003–2007	1.0	28.1	90.6	3.0	51.4	119.8	0.6	13.0	99.8	1.8	22.8	122.5
2008–2012	1.0	31.2	95.8	1.8	39.4	99.6	0.6	14.6	101.8	0.6	16.0	90.1
	35–44			>74 y/o			35–44			>74 y/o		
1983–1987	3.2	40.1	125.9	0.8	58.5	74.7	1.2	13.5	114.3	1.6	52.9	91.7
1988–1992	1.8	25.6	108.0	2.2	81.6	126.5	1.0	11.0	113.8	3.4	73.0	152.6
1993–1997	2.0	29.4	119.9	1.8	61.5	118.1	0.4	8.1	95.2	2.6	47.5	138.1
1998–2002	2.6	42.8	121.4	2.4	65.4	124.1	0.4	9.7	93.4	2.0	33.2	107.0
2003–2007	1.6	37.6	93.8	2.6	56.1	124.5	1.4	15.9	125.8	0.8	15.7	70.1
2008–2012	2.2	40.1	107.8	2.0	43.0	103.7	0.6	13.3	98.4	1.8	21.6	112.1

Secondary Medical Zone ID: 230

	Male						Female					
	Suicide			Suicide			Suicide			Suicide		
	Num per year	Rate /100,000	×100	Num per year	Rate /100,000	×100	Num per year	Rate /100,000	×100	Num per year	Rate /100,000	×100
	Total (>10 y/o)			45–54			Total (>10 y/o)			45–54		
1983–1987	16.2	38.3	137.4	5.2	76.0	150.4	8.4	16.4	118.7	0.6	15.6	90.6
1988–1992	14.6	29.9	151.8	3.4	50.1	145.9	7.8	14.1	121.7	2.0	19.8	135.8
1993–1997	11.0	27.8	118.8	2.2	39.3	107.4	9.0	13.3	147.3	0.4	11.8	91.6
1998–2002	18.6	41.8	135.8	5.0	74.2	132.6	9.2	14.3	133.6	2.2	20.8	138.3
2003–2007	15.2	39.6	120.6	2.6	56.4	100.8	7.0	13.6	117.3	1.6	17.9	130.3
2008–2012	15.6	38.3	127.9	3.4	61.5	126.6	5.8	13.0	107.9	0.2	12.8	85.9
	15–24			55–64			15–24			55–64		
1983–1987	0.8	15.6	112.1	1.2	34.5	81.2	0.0	5.6	82.3	3.2	32.1	162.7
1988–1992	0.8	11.3	114.1	4.4	57.7	162.6	0.2	5.2	97.9	0.8	16.7	93.8
1993–1997	0.6	11.7	103.6	2.8	47.4	117.8	0.2	5.2	99.2	1.8	21.4	134.7
1998–2002	0.6	15.6	98.6	4.6	81.3	127.9	0.2	6.8	96.8	2.0	23.7	126.0
2003–2007	0.6	17.3	98.9	4.6	75.7	131.1	0.0	7.4	82.8	1.6	19.7	117.8
2008–2012	0.6	20.8	98.0	3.4	55.3	113.9	0.4	10.6	109.1	0.6	14.2	95.4
	25–34			65–74			25–34			65–74		
1983–1987	2.0	31.9	124.8	2.0	54.1	123.5	0.8	12.2	113.3	1.4	32.7	104.7
1988–1992	0.6	17.9	93.5	2.4	49.6	140.7	0.2	7.9	91.3	1.4	28.1	107.6
1993–1997	1.8	27.2	139.5	0.8	26.5	83.0	1.0	11.1	132.9	1.4	22.7	116.3
1998–2002	1.4	29.2	108.3	4.0	63.1	138.8	0.4	11.3	97.5	2.0	27.0	128.5
2003–2007	0.8	28.3	91.4	3.4	58.5	136.3	0.8	14.5	111.4	1.2	20.2	108.7
2008–2012	2.4	41.6	127.7	2.2	45.0	113.9	0.6	15.0	104.8	1.0	18.6	104.7
	35–44			>74 y/o			35–44			>74 y/o		
1983–1987	3.4	45.9	144.2	1.2	73.2	93.4	0.6	11.8	99.5	1.8	61.0	105.7
1988–1992	1.6	26.4	111.3	1.4	66.1	102.5	0.8	10.8	111.4	2.4	61.5	128.4
1993–1997	2.2	33.3	135.9	0.6	39.5	75.9	1.8	14.8	174.2	2.4	49.8	144.7
1998–2002	2.4	44.1	125.1	0.6	38.4	72.9	0.4	10.1	97.8	2.0	36.5	117.4
2003–2007	2.4	49.8	124.3	0.8	34.9	77.4	0.6	13.0	102.7	1.2	20.8	92.8
2008–2012	1.2	35.5	95.5	2.4	50.4	121.5	0.4	13.0	95.6	2.6	28.6	148.8

Secondary Medical Zone ID: 231

	Male						Female					
	Suicide			Suicide			Suicide			Suicide		
	Num per year	Rate /100,000	×100	Num per year	Rate /100,000	×100	Num per year	Rate /100,000	×100	Num per year	Rate /100,000	×100
	Total (>10 y/o)			45-54			Total (>10 y/o)			45-54		
1983-1987	29.8	40.8	140.4	7.6	68.6	135.7	16.2	17.7	126.7	3.2	23.1	134.1
1988-1992	24.2	30.3	142.1	4.8	44.5	129.5	12.0	13.2	107.9	1.0	13.0	89.1
1993-1997	24.0	29.5	134.1	3.0	33.4	91.2	12.6	12.1	122.9	2.4	16.4	126.7
1998-2002	31.2	39.9	122.9	4.8	51.1	91.3	13.0	13.5	112.2	2.0	16.5	109.7
2003-2007	34.0	43.0	135.8	6.2	64.7	115.7	10.0	12.7	99.2	0.6	11.3	82.4
2008-2012	22.8	35.5	104.8	4.0	48.5	99.8	13.4	13.8	121.4	1.4	15.3	102.7
	15-24			55-64			15-24			55-64		
1983-1987	2.6	22.3	160.2	4.8	49.9	117.3	0.8	7.5	109.8	2.2	20.6	104.5
1988-1992	1.2	11.4	114.8	6.4	54.0	152.0	0.8	6.4	122.1	1.4	15.9	89.1
1993-1997	0.6	10.3	90.7	6.4	56.1	139.3	0.4	5.1	98.6	2.4	19.3	121.7
1998-2002	0.6	13.5	85.2	8.2	80.6	126.8	0.4	6.7	94.8	2.2	19.9	105.8
2003-2007	0.8	16.3	93.5	9.0	80.4	139.2	0.6	9.2	102.7	2.8	20.1	120.2
2008-2012	1.4	23.1	108.7	6.4	57.1	117.6	0.4	9.1	93.9	1.8	15.7	105.3
	25-34			65-74			25-34			65-74		
1983-1987	3.0	30.2	118.2	3.4	55.3	126.2	1.4	12.4	115.6	3.8	43.2	138.3
1988-1992	2.0	22.1	115.8	3.0	42.7	121.1	0.8	8.8	101.6	3.2	33.4	127.9
1993-1997	3.0	27.5	140.7	4.6	48.7	152.6	0.6	7.9	94.6	2.4	22.7	116.4
1998-2002	3.0	30.8	114.1	5.6	56.5	124.3	1.2	12.2	105.6	2.2	21.5	102.4
2003-2007	2.2	29.5	95.2	5.4	55.4	129.1	0.8	12.0	92.6	1.2	15.7	84.5
2008-2012	2.6	35.1	107.8	2.2	33.6	85.1	1.2	15.4	107.4	3.0	22.9	128.7
	35-44			>74 y/o			35-44			>74 y/o		
1983-1987	3.6	34.2	107.4	4.8	121.0	154.5	1.4	12.2	103.5	3.4	65.5	113.5
1988-1992	3.8	31.4	132.5	3.0	73.2	113.6	1.0	9.7	100.0	3.8	56.7	118.5
1993-1997	3.0	29.0	118.4	3.4	68.6	131.8	1.0	9.3	108.9	3.4	41.4	120.4
1998-2002	4.8	46.8	132.9	4.2	69.4	131.6	1.8	13.7	132.5	3.2	32.9	105.8
2003-2007	5.2	54.5	135.9	5.2	65.7	145.9	1.6	14.4	114.3	2.2	20.2	89.9
2008-2012	2.6	34.9	93.7	3.6	44.5	107.2	1.0	13.0	95.9	4.6	30.0	155.7

Secondary Medical Zone ID: 232

	Male						Female					
	Suicide			Suicide			Suicide			Suicide		
	Num per year	Rate /100,000	×100	Num per year	Rate /100,000	×100	Num per year	Rate /100,000	×100	Num per year	Rate /100,000	×100
	Total (>10 y/o)			45-54			Total (>10 y/o)			45-54		
1983-1987	18.6	37.6	128.8	5.6	69.8	138.1	6.8	14.1	87.0	1.6	18.9	109.6
1988-1992	13.8	27.7	123.6	3.6	45.9	133.7	10.0	13.9	120.2	1.2	15.4	105.6
1993-1997	13.0	26.9	117.1	4.6	51.2	140.1	7.6	11.4	111.3	0.8	12.5	97.1
1998-2002	19.4	39.4	123.4	3.8	59.1	105.6	8.8	13.6	113.6	0.8	14.4	95.6
2003-2007	15.8	37.9	110.9	3.2	59.6	106.6	9.4	14.0	126.1	1.2	15.7	114.6
2008-2012	13.8	35.6	109.6	1.8	46.6	95.8	5.6	13.1	99.4	0.2	12.6	84.6
	15-24			55-64			15-24			55-64		
1983-1987	0.8	15.2	109.1	3.2	47.5	111.8	0.4	7.1	103.7	0.4	13.9	70.6
1988-1992	0.6	10.6	107.1	3.2	42.3	119.1	0.2	5.1	97.1	1.8	20.0	111.9
1993-1997	1.2	14.5	127.8	2.4	39.9	99.0	0.2	5.2	99.0	2.2	21.5	135.2
1998-2002	0.8	17.1	108.4	4.8	73.1	115.1	0.2	6.8	97.2	3.0	26.2	139.0
2003-2007	0.6	18.0	103.3	5.4	73.8	127.8	0.4	9.8	108.7	1.6	18.4	110.0
2008-2012	0.4	20.6	96.9	4.0	55.6	114.4	0.6	12.0	123.6	1.6	17.2	115.4
	25-34			65-74			25-34			65-74		
1983-1987	2.0	30.7	120.0	2.0	47.6	108.7	0.2	8.8	81.8	1.6	29.9	95.8
1988-1992	1.0	20.8	108.7	1.4	33.9	96.1	0.8	10.4	119.6	1.4	24.0	91.9
1993-1997	0.6	18.5	94.6	1.4	29.4	92.3	0.4	8.5	102.2	1.8	22.6	116.0
1998-2002	1.4	29.3	108.4	2.8	47.3	103.9	0.4	11.3	97.4	1.6	21.7	103.1
2003-2007	1.0	30.2	97.5	2.6	45.3	105.7	0.2	11.7	89.9	1.2	18.4	98.9
2008-2012	0.8	31.7	97.4	3.0	46.7	118.3	0.6	15.2	106.4	1.6	20.0	112.4
	35-44			>74 y/o			35-44			>74 y/o		
1983-1987	2.8	36.5	114.7	2.2	86.1	109.9	1.4	13.9	117.3	1.2	39.8	68.9
1988-1992	2.6	31.2	131.6	1.4	56.7	87.8	1.0	10.9	113.0	3.6	65.6	137.0
1993-1997	1.4	25.6	104.5	1.4	48.3	92.8	0.4	8.2	97.0	1.8	31.8	92.5
1998-2002	2.4	43.1	122.5	3.4	74.6	141.5	1.0	12.7	123.1	1.8	27.4	88.2
2003-2007	1.2	35.9	89.5	1.8	42.6	94.6	1.4	16.4	130.2	3.4	35.4	157.6
2008-2012	2.4	43.4	116.6	1.4	34.4	82.9	0.2	12.2	89.9	0.8	13.4	69.5

Secondary Medical Zone ID: 233

	Male						Female					
	Suicide			Suicide			Suicide			Suicide		
	Num per year	Rate /100,000	×100	Num per year	Rate /100,000	×100	Num per year	Rate /100,000	×100	Num per year	Rate /100,000	×100
	Total (>10 y/o)			45-54			Total (>10 y/o)			45-54		
1983-1987	31.0	31.8	96.3	7.8	52.2	103.3	13.6	12.8	77.3	3.2	18.4	106.9
1988-1992	30.4	26.9	114.3	4.6	32.5	94.5	14.2	11.5	85.1	2.8	16.2	110.6
1993-1997	24.4	23.3	89.6	5.8	35.0	95.7	12.0	9.7	82.9	2.0	12.5	96.8
1998-2002	35.0	32.4	86.5	8.6	48.2	86.1	12.8	11.1	78.0	2.0	13.2	87.6
2003-2007	41.0	36.6	97.3	10.8	60.4	108.0	13.4	11.5	84.6	2.2	13.3	96.9
2008-2012	46.0	38.7	116.0	8.6	54.2	111.6	15.0	13.3	94.2	2.2	14.6	98.1
	15-24			55-64			15-24			55-64		
1983-1987	2.0	13.4	96.4	4.8	37.7	88.6	0.2	4.6	67.8	2.0	15.3	77.6
1988-1992	1.4	9.3	94.1	7.4	45.9	129.2	0.4	4.2	80.2	2.8	17.2	96.1
1993-1997	1.8	11.0	96.9	5.2	38.2	95.0	0.6	4.7	90.1	2.0	14.0	87.9
1998-2002	2.0	13.9	88.2	8.6	62.4	98.2	0.6	5.9	83.7	1.6	14.9	79.2
2003-2007	2.2	15.8	90.6	8.8	55.1	95.5	1.0	8.4	93.3	2.2	15.2	91.0
2008-2012	3.8	25.7	121.1	10.0	52.1	107.3	1.6	11.9	122.8	2.4	14.2	95.4
	25-34			65-74			25-34			65-74		
1983-1987	4.6	26.8	104.9	2.6	36.9	84.3	0.4	6.3	58.5	3.4	30.7	98.3
1988-1992	4.2	25.1	131.4	2.8	32.1	91.2	1.4	8.9	102.3	2.0	18.3	70.3
1993-1997	3.0	20.8	106.7	2.8	26.9	84.4	1.4	9.0	107.9	2.0	15.2	77.9
1998-2002	3.2	24.1	89.3	4.8	40.7	89.4	1.6	11.4	98.5	3.2	20.4	97.1
2003-2007	4.4	29.7	95.7	6.6	50.5	117.6	1.8	12.8	98.2	1.2	12.6	67.5
2008-2012	7.8	45.3	139.2	4.4	38.3	97.0	2.4	16.1	112.1	1.6	14.7	82.7
	35-44			>74 y/o			35-44			>74 y/o		
1983-1987	6.2	34.8	109.5	3.0	67.5	86.2	0.8	8.5	71.9	3.6	48.6	84.2
1988-1992	5.8	28.3	119.5	4.2	71.4	110.7	1.2	8.3	85.8	3.6	38.3	79.9
1993-1997	3.2	20.0	81.4	2.4	41.5	79.8	1.0	7.1	84.1	3.0	26.4	76.8
1998-2002	4.6	31.1	88.4	3.2	44.3	84.1	1.6	10.3	99.2	2.0	16.3	52.5
2003-2007	6.2	42.3	105.5	2.0	27.2	60.3	1.4	11.4	89.9	3.6	20.1	89.4
2008-2012	6.0	39.3	105.6	5.4	45.2	109.0	2.4	14.9	109.9	2.4	13.4	69.6

Secondary Medical Zone ID: 234

	Male						Female					
	Suicide			Suicide			Suicide			Suicide		
	Num per year	Rate /100,000	×100	Num per year	Rate /100,000	×100	Num per year	Rate /100,000	×100	Num per year	Rate /100,000	×100
	Total (>10 y/o)			45-54			Total (>10 y/o)			45-54		
1983-1987	18.6	34.8	110.3	5.8	66.5	131.5	9.2	14.6	93.0	1.8	18.8	109.3
1988-1992	17.4	27.8	124.7	3.8	43.6	127.1	13.6	14.7	131.8	1.2	14.9	102.2
1993-1997	19.6	28.9	132.4	4.2	43.2	118.1	10.6	11.8	121.4	1.4	14.2	109.8
1998-2002	22.4	37.9	110.5	6.0	62.6	111.8	10.0	12.7	106.2	0.4	11.9	78.7
2003-2007	24.0	38.8	119.3	6.0	69.2	123.8	8.8	12.6	103.6	2.0	17.2	125.2
2008-2012	21.4	37.2	116.1	3.2	48.8	100.3	9.4	13.0	109.9	0.4	12.2	81.8
	15-24			55-64			15-24			55-64		
1983-1987	0.4	11.4	82.0	2.0	34.2	80.4	0.6	7.4	108.8	1.8	19.8	100.5
1988-1992	0.8	10.6	107.0	3.2	38.6	108.8	0.4	5.5	103.6	2.4	21.3	119.5
1993-1997	1.4	13.8	122.1	3.8	46.3	115.1	0.4	5.4	103.5	1.2	15.4	96.9
1998-2002	0.0	11.3	71.7	6.2	77.8	122.4	0.2	6.2	88.2	2.8	24.4	129.9
2003-2007	0.8	16.8	96.4	4.8	59.2	102.5	0.4	8.7	96.3	1.8	18.3	109.5
2008-2012	1.8	26.3	123.8	5.0	52.7	108.6	0.6	10.7	110.5	1.0	13.9	93.1
	25-34			65-74			25-34			65-74		
1983-1987	2.6	29.3	114.6	2.0	43.6	99.5	0.4	8.8	81.3	1.6	27.4	87.8
1988-1992	1.0	17.5	91.5	2.4	40.3	114.4	1.0	10.3	118.5	2.8	32.5	124.4
1993-1997	0.6	16.2	83.2	3.0	40.7	127.6	0.6	8.7	104.2	3.4	30.8	158.0
1998-2002	1.8	28.0	103.6	3.6	49.8	109.4	0.8	12.0	103.2	1.4	18.8	89.6
2003-2007	2.6	35.4	114.3	3.4	48.2	112.4	0.2	10.5	80.6	1.6	19.4	104.2
2008-2012	1.8	32.5	99.9	3.4	46.4	117.5	0.0	10.9	75.9	2.8	24.6	138.4
	35-44			>74 y/o			35-44			>74 y/o		
1983-1987	3.0	33.7	106.0	2.8	94.6	120.8	0.6	10.4	88.3	2.4	54.2	94.0
1988-1992	4.2	35.3	149.1	2.0	62.0	96.2	0.8	9.5	98.7	5.0	74.8	156.3
1993-1997	3.2	32.4	132.3	3.4	74.9	143.8	0.2	6.6	77.8	3.4	43.8	127.4
1998-2002	3.0	39.8	113.0	1.8	45.0	85.4	0.4	9.0	87.1	4.0	41.7	134.3
2003-2007	1.6	33.2	82.8	4.8	69.8	154.9	0.4	11.1	87.8	2.4	22.8	101.6
2008-2012	3.6	44.9	120.7	2.4	39.8	96.0	1.0	14.2	105.0	3.6	26.5	138.1

Secondary Medical Zone ID: 235

	Male						Female					
	Suicide			Suicide			Suicide			Suicide		
	Num per year	Rate /100,000	×100	Num per year	Rate /100,000	×100	Num per year	Rate /100,000	×100	Num per year	Rate /100,000	×100
	Total (>10 y/o)			45-54			Total (>10 y/o)			45-54		
1983-1987	37.2	35.5	112.1	10.2	61.1	120.7	16.8	14.1	88.5	2.8	16.7	97.1
1988-1992	30.8	27.2	114.8	8.4	46.9	136.6	16.2	12.0	91.6	2.8	15.5	106.0
1993-1997	36.6	29.3	126.0	10.2	49.5	135.3	16.6	10.9	103.5	3.2	15.1	116.7
1998-2002	46.6	39.3	110.7	13.4	68.0	121.6	15.8	11.6	88.2	3.6	17.2	114.1
2003-2007	51.0	41.5	120.0	13.6	77.8	139.1	13.8	11.2	84.1	1.6	11.9	86.7
2008-2012	41.0	36.6	107.7	7.6	52.9	108.9	16.4	12.9	98.3	2.2	15.1	101.4
	15-24			55-64			15-24			55-64		
1983-1987	2.0	15.1	108.0	6.4	45.1	106.1	1.8	9.4	137.8	2.6	17.0	86.2
1988-1992	1.2	9.3	94.4	7.4	44.7	125.8	0.2	3.7	71.0	2.8	16.8	94.2
1993-1997	1.0	9.4	83.3	8.2	50.0	124.1	0.8	5.4	103.6	3.4	18.3	115.2
1998-2002	2.6	17.6	111.1	9.2	60.8	95.7	0.4	5.6	79.3	3.0	18.3	97.4
2003-2007	2.2	18.0	103.1	12.8	69.6	120.6	0.6	7.4	82.2	1.8	13.4	79.9
2008-2012	2.6	23.3	109.9	9.4	51.3	105.6	1.4	11.7	120.8	2.2	13.4	90.1
	25-34			65-74			25-34			65-74		
1983-1987	4.6	27.9	109.3	3.6	44.4	101.3	1.0	8.4	78.4	2.6	24.9	79.7
1988-1992	3.6	23.5	122.8	3.4	35.5	100.7	1.0	8.0	91.7	2.8	21.6	82.8
1993-1997	3.6	23.6	120.7	5.4	41.3	129.6	0.6	6.7	80.5	3.4	20.7	106.2
1998-2002	5.8	33.9	125.4	5.6	43.2	95.1	1.0	9.6	82.5	3.2	19.8	94.5
2003-2007	5.8	35.7	115.0	6.8	48.6	113.3	1.4	11.4	87.6	2.4	16.4	88.0
2008-2012	5.6	38.1	116.9	6.2	43.6	110.5	1.6	13.5	94.6	1.6	13.9	78.2
	35-44			>74 y/o			35-44			>74 y/o		
1983-1987	5.4	29.8	93.7	5.0	99.8	127.4	3.0	13.8	117.0	3.0	41.1	71.3
1988-1992	4.0	22.1	93.4	2.8	53.9	83.6	2.0	10.2	105.2	4.6	45.0	94.1
1993-1997	4.8	27.7	113.0	3.4	52.3	100.5	1.0	7.3	86.5	4.2	33.0	95.9
1998-2002	6.6	42.3	120.2	3.4	45.0	85.3	1.0	8.7	84.0	3.6	23.8	76.6
2003-2007	5.6	40.3	100.6	4.2	41.7	92.7	1.4	11.4	90.4	4.6	23.2	103.3
2008-2012	5.2	36.4	97.8	4.4	37.7	90.9	1.8	13.0	95.7	5.6	23.7	123.2

Secondary Medical Zone ID: 236

	Male						Female					
	Suicide			Suicide			Suicide			Suicide		
	Num per year	Rate /100,000	×100	Num per year	Rate /100,000	×100	Num per year	Rate /100,000	×100	Num per year	Rate /100,000	×100
	Total (>10 y/o)			45-54			Total (>10 y/o)			45-54		
1983-1987	40.8	37.7	121.4	9.8	58.4	115.4	16.2	14.0	88.3	2.8	16.7	97.0
1988-1992	31.6	27.3	116.1	6.4	38.1	111.0	14.4	11.5	84.2	2.0	13.3	90.7
1993-1997	31.8	26.7	109.2	9.4	46.4	126.8	15.8	10.8	99.6	3.6	16.1	124.3
1998-2002	50.4	39.9	115.6	12.8	64.5	115.3	14.6	11.4	82.9	1.2	10.8	72.0
2003-2007	46.4	38.6	106.7	9.6	57.1	102.2	16.4	12.0	94.5	1.8	12.4	90.5
2008-2012	45.2	37.3	112.2	6.2	44.0	90.5	11.4	11.4	77.1	1.6	12.8	86.1
	15-24			55-64			15-24			55-64		
1983-1987	1.4	11.0	79.0	7.6	52.3	123.0	1.0	6.7	98.1	2.2	16.4	83.3
1988-1992	2.0	10.7	108.5	6.6	40.9	115.2	0.0	3.0	56.6	2.6	16.4	92.0
1993-1997	1.8	10.9	96.1	6.4	41.3	102.6	0.4	4.0	77.0	2.4	14.9	93.8
1998-2002	3.8	19.6	123.9	12.4	76.0	119.7	0.4	5.2	74.5	2.6	17.1	90.9
2003-2007	3.2	19.6	112.6	9.2	53.8	93.2	0.8	7.6	84.2	4.6	20.7	123.9
2008-2012	4.4	28.2	132.9	11.6	57.9	119.2	1.8	12.5	129.0	1.8	12.5	83.9
	25-34			65-74			25-34			65-74		
1983-1987	2.8	20.2	79.2	4.4	53.3	121.8	1.8	11.0	101.9	2.2	23.9	76.6
1988-1992	3.0	20.4	106.8	3.4	36.3	103.1	1.4	9.0	103.4	3.4	26.6	101.8
1993-1997	2.6	18.8	96.5	4.0	33.8	106.1	1.2	8.3	99.1	2.6	18.2	93.2
1998-2002	5.4	31.4	116.3	6.2	46.4	101.9	3.2	15.6	134.3	2.8	18.7	89.1
2003-2007	6.6	38.2	123.3	6.2	45.2	105.3	2.4	14.3	110.0	2.6	17.2	92.2
2008-2012	5.6	37.5	115.1	5.4	40.2	101.8	1.4	12.8	89.3	1.8	14.6	81.9
	35-44			>74 y/o			35-44			>74 y/o		
1983-1987	12.0	54.5	171.3	2.8	67.7	86.4	2.6	12.7	107.8	3.4	47.1	81.7
1988-1992	5.8	28.3	119.5	4.2	72.9	113.0	2.4	11.1	115.0	2.6	29.0	60.5
1993-1997	5.0	28.1	114.6	2.6	43.6	83.7	1.6	9.0	106.4	4.0	32.3	93.8
1998-2002	6.2	38.9	110.4	3.4	45.2	85.7	0.8	7.9	76.0	3.4	23.6	75.9
2003-2007	7.0	45.2	112.8	4.6	44.7	99.3	1.0	9.9	78.5	3.2	17.9	79.9
2008-2012	6.8	40.4	108.7	5.2	42.3	101.9	0.6	9.4	69.1	2.4	13.0	67.8

Secondary Medical Zone ID: 237

	Male						Female					
	Suicide			Suicide			Suicide			Suicide		
	Num per year	Rate /100,000	×100	Num per year	Rate /100,000	×100	Num per year	Rate /100,000	×100	Num per year	Rate /100,000	×100
	Total (>10 y/o)			45–54			Total (>10 y/o)			45–54		
1983–1987	14.4	35.4	118.5	4.0	62.8	124.2	9.4	17.2	124.4	1.4	19.2	111.4
1988–1992	17.0	32.1	163.6	3.4	50.7	147.6	8.2	14.1	120.9	1.6	18.3	125.3
1993–1997	15.4	30.3	148.2	3.2	46.4	126.7	6.8	11.8	118.1	0.4	12.1	93.3
1998–2002	17.0	38.8	124.9	4.0	63.9	114.2	7.6	13.3	117.4	1.4	17.9	118.6
2003–2007	18.8	42.0	142.6	3.6	67.1	120.0	5.8	12.5	107.3	0.4	12.9	94.2
2008–2012	19.0	40.7	148.3	3.0	60.3	124.0	4.4	12.8	98.6	0.6	15.3	102.8
	15–24			55–64			15–24			55–64		
1983–1987	0.6	14.9	107.0	2.6	44.6	105.1	0.4	7.7	112.2	1.6	21.5	109.0
1988–1992	0.8	11.9	120.1	3.4	47.0	132.5	0.6	7.0	133.4	0.6	15.1	84.7
1993–1997	0.2	10.5	92.9	3.8	54.7	135.9	0.6	7.0	133.3	1.0	16.5	103.9
1998–2002	1.2	19.5	123.5	2.2	58.1	91.5	0.4	7.8	111.2	1.0	19.4	102.9
2003–2007	1.0	20.4	116.8	4.4	73.8	127.8	0.4	10.0	111.1	0.8	16.9	100.7
2008–2012	1.4	27.4	129.2	3.6	55.1	113.4	0.0	8.4	86.6	0.4	13.7	91.7
	25–34			65–74			25–34			65–74		
1983–1987	0.6	21.1	82.5	1.4	43.0	98.3	0.8	12.6	116.6	1.4	31.3	100.2
1988–1992	1.4	25.0	130.9	2.0	41.6	118.0	0.6	10.0	115.7	1.6	28.1	107.5
1993–1997	0.6	19.6	100.1	4.0	57.0	178.6	0.4	9.0	107.6	1.2	19.7	101.3
1998–2002	2.4	37.6	139.4	2.4	46.8	102.9	0.4	11.9	102.3	1.2	20.6	98.1
2003–2007	1.8	37.4	120.6	2.4	47.7	111.1	0.4	13.2	101.2	0.6	16.4	87.8
2008–2012	2.4	42.3	130.0	2.8	50.7	128.5	0.4	14.5	101.5	1.0	18.9	106.4
	35–44			>74 y/o			35–44			>74 y/o		
1983–1987	3.0	43.0	135.2	2.0	85.6	109.3	0.4	11.1	94.3	3.4	92.5	160.4
1988–1992	3.2	37.4	157.6	2.8	89.3	138.4	0.8	10.9	112.9	2.4	56.1	117.2
1993–1997	2.2	33.2	135.5	1.4	49.8	95.6	0.8	10.5	123.6	2.4	43.7	127.1
1998–2002	1.2	34.2	97.1	3.6	77.9	147.8	0.0	8.5	82.6	3.2	44.9	144.8
2003–2007	1.4	42.0	104.7	4.2	72.6	161.2	0.0	10.8	85.8	3.2	35.8	159.3
2008–2012	1.8	41.6	111.9	4.0	61.4	147.9	0.4	13.6	100.3	1.6	19.3	100.1

Secondary Medical Zone ID: 238

	Male						Female					
	Suicide			Suicide			Suicide			Suicide		
	Num per year	Rate /100,000	×100	Num per year	Rate /100,000	×100	Num per year	Rate /100,000	×100	Num per year	Rate /100,000	×100
	Total (>10 y/o)			45–54			Total (>10 y/o)			45–54		
1983–1987	26.2	35.7	111.8	7.6	63.6	125.8	13.8	15.4	101.8	2.4	18.5	107.6
1988–1992	23.0	27.3	119.8	3.8	35.2	102.5	11.4	12.2	93.4	1.6	14.5	99.5
1993–1997	19.2	24.5	97.7	3.2	30.5	83.3	11.4	11.0	102.9	1.6	13.0	100.8
1998–2002	33.6	38.5	112.8	8.8	63.4	113.3	11.2	11.4	91.1	1.6	14.1	93.5
2003–2007	37.0	41.9	121.9	8.6	69.6	124.5	13.8	12.5	109.3	1.0	11.8	85.9
2008–2012	33.0	37.8	116.6	7.4	63.7	131.1	12.0	12.6	100.8	1.0	12.9	86.7
	15–24			55–64			15–24			55–64		
1983–1987	1.0	12.8	91.6	4.0	41.6	97.8	0.0	4.7	68.3	3.4	24.7	125.2
1988–1992	0.8	9.4	94.7	7.6	57.3	161.5	0.2	4.2	79.8	2.4	18.6	104.4
1993–1997	0.8	10.2	90.1	4.4	41.1	102.0	1.2	7.3	140.7	2.4	18.1	113.6
1998–2002	1.4	15.6	98.8	6.0	59.9	94.3	0.8	7.5	107.1	1.8	17.8	94.3
2003–2007	2.6	22.5	128.8	8.6	66.7	115.6	0.0	5.8	64.2	2.4	17.9	106.8
2008–2012	1.2	19.5	91.9	6.6	49.5	101.9	0.6	9.1	93.8	2.2	15.7	105.2
	25–34			65–74			25–34			65–74		
1983–1987	4.0	31.8	124.4	2.0	38.4	87.7	1.2	10.8	100.8	1.4	22.7	72.5
1988–1992	2.4	22.5	117.6	3.4	43.3	122.8	0.4	7.1	81.5	3.0	29.8	114.1
1993–1997	1.2	16.4	84.2	2.8	32.7	102.4	0.2	6.3	76.0	1.4	15.5	79.7
1998–2002	3.8	31.7	117.5	3.6	40.8	89.7	0.2	8.4	72.3	1.6	16.9	80.2
2003–2007	4.0	34.4	111.0	5.0	48.7	113.5	0.6	10.4	79.6	3.0	22.5	120.7
2008–2012	3.6	34.8	106.9	4.8	46.4	117.4	1.0	13.1	91.6	2.6	20.7	116.1
	35–44			>74 y/o			35–44			>74 y/o		
1983–1987	4.6	34.8	109.4	2.8	79.0	100.8	2.4	15.0	126.8	3.0	53.9	93.5
1988–1992	3.0	23.1	97.6	2.0	53.3	82.7	1.4	10.2	105.3	2.4	35.7	74.6
1993–1997	3.6	28.5	116.3	3.0	58.5	112.4	1.2	9.3	109.4	3.2	35.0	101.8
1998–2002	4.6	41.3	117.2	5.4	77.8	147.7	0.2	7.2	69.4	5.0	42.1	135.6
2003–2007	4.6	44.5	111.0	3.4	45.4	100.9	1.6	13.9	109.9	5.2	34.7	154.7
2008–2012	4.8	40.9	109.8	4.6	47.3	114.0	1.4	13.5	99.9	3.2	19.9	103.6

Secondary Medical Zone ID: 239

	Male						Female					
	Suicide			Suicide			Suicide			Suicide		
	Num per year	Rate /100,000	×100	Num per year	Rate /100,000	×100	Num per year	Rate /100,000	×100	Num per year	Rate /100,000	×100
	Total (>10 y/o)			45–54			Total (>10 y/o)			45–54		
1983–1987	20.0	40.4	150.9	3.8	63.6	125.8	11.4	17.8	132.2	1.6	20.3	117.5
1988–1992	16.0	30.4	152.9	2.0	39.2	114.2	13.0	15.8	158.1	1.4	17.5	119.8
1993–1997	16.4	31.2	155.8	5.0	59.6	162.8	8.8	12.4	133.8	1.0	14.6	113.0
1998–2002	19.0	41.1	138.7	4.6	71.1	127.1	6.6	12.7	105.4	0.8	15.5	103.2
2003–2007	18.6	42.6	145.9	4.0	74.7	133.6	5.4	12.8	102.6	1.0	15.7	114.4
2008–2012	14.4	39.0	126.1	3.8	69.0	141.9	6.4	13.1	114.9	0.6	15.4	103.4
	15–24			55–64			15–24			55–64		
1983–1987	0.4	13.7	98.1	4.2	57.5	135.4	0.4	7.6	111.5	1.2	18.6	94.3
1988–1992	0.2	9.5	95.9	2.6	40.8	114.8	0.0	4.6	87.3	2.2	22.7	127.4
1993–1997	0.6	12.5	110.4	1.2	34.5	85.7	0.4	6.2	119.5	2.0	22.0	138.5
1998–2002	0.6	16.7	105.7	4.4	82.2	129.4	0.0	6.3	89.4	0.2	15.6	82.9
2003–2007	0.6	18.5	106.0	5.0	80.0	138.7	0.2	9.0	100.2	0.8	16.8	100.2
2008–2012	0.4	21.3	100.4	2.4	48.0	98.8	0.0	8.6	88.7	0.8	15.3	102.5
	25–34			65–74			25–34			65–74		
1983–1987	1.8	31.0	121.3	2.0	46.2	105.6	1.6	16.9	157.0	3.6	48.7	155.9
1988–1992	1.4	25.6	134.1	2.4	43.3	123.0	0.4	9.2	105.6	3.4	42.0	161.1
1993–1997	0.8	21.5	109.9	3.0	45.9	144.0	0.4	9.0	108.5	1.4	20.3	104.2
1998–2002	1.0	28.9	106.8	3.6	57.4	126.2	0.8	13.7	118.6	1.8	24.0	114.1
2003–2007	1.0	32.2	103.7	2.6	50.5	117.6	0.0	11.3	86.9	1.2	19.8	106.1
2008–2012	1.4	36.6	112.3	1.6	40.7	103.2	0.0	12.7	88.6	1.8	22.8	128.1
	35–44			>74 y/o			35–44			>74 y/o		
1983–1987	3.0	43.5	136.8	4.6	134.2	171.3	0.8	12.9	109.0	2.2	57.3	99.3
1988–1992	3.0	36.8	155.4	4.4	110.3	171.1	0.4	9.5	98.4	5.2	88.3	184.5
1993–1997	2.4	36.0	146.9	3.4	78.4	150.5	0.6	9.7	114.0	3.0	45.7	132.9
1998–2002	1.4	37.1	105.2	3.4	70.0	132.8	0.2	9.6	92.8	2.8	36.8	118.6
2003–2007	1.4	43.4	108.3	4.0	68.0	151.0	0.4	12.8	101.7	1.8	22.0	97.9
2008–2012	2.4	46.7	125.5	2.4	45.1	108.8	0.4	13.7	101.2	2.8	26.2	136.5

Secondary Medical Zone ID: 240

	Male						Female					
	Suicide			Suicide			Suicide			Suicide		
	Num per year	Rate /100,000	×100	Num per year	Rate /100,000	×100	Num per year	Rate /100,000	×100	Num per year	Rate /100,000	×100
	Total (>10 y/o)			45-54			Total (>10 y/o)			45-54		
1983-1987	21.8	39.0	136.3	6.0	71.6	141.6	15.2	19.0	146.9	1.8	19.4	112.5
1988-1992	17.4	29.4	138.4	4.2	49.7	144.7	11.2	14.2	125.7	1.0	14.7	100.2
1993-1997	20.0	31.1	150.7	4.6	48.5	132.7	7.8	11.2	108.4	1.6	15.7	121.3
1998-2002	21.4	39.9	122.1	7.0	77.3	138.0	10.8	13.9	124.2	1.6	17.3	114.8
2003-2007	24.6	43.2	142.7	4.6	66.3	118.5	8.0	12.7	109.4	0.8	13.7	100.2
2008-2012	19.0	36.9	119.6	3.0	50.6	104.1	7.0	13.4	105.1	1.0	16.2	108.6
	15-24			55-64			15-24			55-64		
1983-1987	1.0	16.1	115.3	3.2	44.9	105.8	0.2	6.4	93.3	3.2	27.6	139.7
1988-1992	0.8	11.1	111.7	3.8	45.5	128.1	0.2	5.1	96.4	1.6	18.3	102.6
1993-1997	0.8	12.2	107.9	4.6	55.0	136.6	0.2	5.1	96.8	1.8	18.9	118.9
1998-2002	1.8	21.1	133.7	3.0	56.6	89.1	0.4	7.4	105.3	1.8	21.1	112.1
2003-2007	1.6	21.9	125.5	7.8	89.5	155.0	0.4	9.3	103.5	1.6	18.5	110.4
2008-2012	1.6	26.3	124.2	4.8	56.2	115.6	0.2	9.1	94.0	1.2	15.5	104.0
	25-34			65-74			25-34			65-74		
1983-1987	1.8	26.7	104.4	2.2	47.3	108.1	1.2	13.4	124.7	4.0	50.1	160.3
1988-1992	1.8	25.0	130.9	2.6	43.9	124.7	0.6	9.3	107.5	3.0	36.0	138.1
1993-1997	1.0	20.2	103.2	2.4	37.2	116.6	0.2	7.5	90.6	1.8	21.4	109.9
1998-2002	1.4	27.8	103.1	3.2	49.4	108.6	0.2	10.2	88.4	3.2	30.0	142.9
2003-2007	1.6	32.3	104.1	2.6	45.1	105.2	0.8	14.1	108.7	1.2	17.9	96.3
2008-2012	0.8	28.6	87.8	2.4	42.4	107.3	0.6	14.6	102.1	1.4	19.1	107.5
	35-44			>74 y/o			35-44			>74 y/o		
1983-1987	3.8	41.4	130.1	3.8	119.5	152.6	0.8	11.7	99.0	4.0	88.2	152.9
1988-1992	1.8	24.1	101.5	2.2	69.1	107.1	0.4	8.7	90.4	4.4	75.0	156.8
1993-1997	3.2	36.2	147.5	3.4	79.7	153.0	0.6	8.9	104.7	1.6	28.1	81.6
1998-2002	2.6	40.9	116.1	2.4	56.4	107.0	0.8	11.6	111.8	2.6	33.2	106.8
2003-2007	2.2	43.0	107.3	4.2	68.1	151.3	0.0	10.1	80.0	3.2	31.2	138.9
2008-2012	2.6	41.1	110.4	3.8	56.6	136.5	1.0	15.1	111.5	1.6	17.1	89.1

Secondary Medical Zone ID: 241

	Male						Female					
	Suicide			Suicide			Suicide			Suicide		
	Num per year	Rate /100,000	×100	Num per year	Rate /100,000	×100	Num per year	Rate /100,000	×100	Num per year	Rate /100,000	×100
	Total (>10 y/o)			45-54			Total (>10 y/o)			45-54		
1983-1987	18.4	39.0	142.7	4.8	69.4	137.2	11.2	18.5	139.6	1.4	19.0	110.3
1988-1992	14.8	28.9	145.2	1.4	32.4	94.4	7.6	13.5	113.0	1.2	16.3	111.2
1993-1997	15.4	30.2	145.9	3.8	49.0	134.0	5.8	11.0	104.7	1.2	14.9	115.2
1998-2002	24.2	44.9	162.8	5.0	71.0	126.9	7.6	13.1	114.2	1.0	15.8	104.7
2003-2007	19.8	43.5	146.9	3.8	68.3	122.1	6.2	13.0	108.0	0.8	14.3	104.7
2008-2012	11.0	33.9	101.8	2.2	51.9	106.8	5.8	13.4	107.3	0.6	15.0	100.3
	15-24			55-64			15-24			55-64		
1983-1987	0.6	14.7	105.6	4.6	63.2	148.8	0.2	6.7	98.1	2.4	26.3	133.5
1988-1992	0.6	11.0	110.7	3.8	50.5	142.4	0.0	4.5	86.0	1.2	18.2	101.7
1993-1997	0.6	12.1	107.3	4.2	57.5	142.7	0.2	5.3	101.2	1.0	16.3	102.3
1998-2002	0.6	16.2	102.7	4.4	78.4	123.4	0.4	7.7	109.9	1.0	18.7	99.6
2003-2007	0.8	19.3	110.6	5.4	80.5	139.4	0.0	7.7	85.2	1.0	17.0	101.7
2008-2012	0.0	18.1	85.4	2.2	45.2	93.0	0.4	10.9	112.6	1.4	17.1	114.4
	25-34			65-74			25-34			65-74		
1983-1987	2.2	32.5	127.1	2.2	53.7	122.6	1.2	14.2	131.8	1.6	33.8	108.1
1988-1992	1.2	23.0	120.6	2.2	45.1	127.9	0.2	8.0	92.2	1.8	30.6	117.4
1993-1997	1.2	23.7	121.1	1.6	33.5	105.1	0.0	7.1	85.2	1.8	24.6	126.1
1998-2002	2.6	38.6	142.8	3.2	53.9	118.4	0.0	9.9	85.7	2.2	27.1	128.9
2003-2007	2.4	42.0	135.3	2.6	49.1	114.4	1.2	16.8	129.1	0.8	17.2	92.4
2008-2012	0.8	31.7	97.4	1.4	38.0	96.3	0.4	14.4	100.4	0.6	16.3	91.6
	35-44			>74 y/o			35-44			>74 y/o		
1983-1987	2.0	33.5	105.3	2.0	87.8	112.1	0.8	12.3	104.3	3.6	99.5	172.4
1988-1992	1.4	23.9	101.0	4.0	113.4	175.8	0.2	8.4	87.5	3.0	65.6	137.0
1993-1997	1.6	27.5	112.3	2.4	69.7	133.8	0.4	8.5	99.5	1.2	27.9	81.1
1998-2002	3.2	52.3	148.4	5.0	103.4	196.1	0.4	10.2	98.9	2.6	39.4	126.8
2003-2007	2.0	48.3	120.5	2.8	57.9	128.5	0.2	11.6	91.6	2.2	28.0	124.7
2008-2012	1.0	35.7	95.9	3.4	57.2	137.8	0.6	14.3	105.2	1.8	21.1	109.5

Secondary Medical Zone ID: 242

	Male						Female					
	Suicide			Suicide			Suicide			Suicide		
	Num per year	Rate /100,000	×100	Num per year	Rate /100,000	×100	Num per year	Rate /100,000	×100	Num per year	Rate /100,000	×100
	Total (>10 y/o)			45–54			Total (>10 y/o)			45–54		
1983–1987	6.8	35.7	131.3	0.8	48.9	96.7	2.4	15.2	96.0	0.2	16.4	95.1
1988–1992	6.0	27.5	139.5	1.6	46.7	135.8	2.2	12.5	96.7	0.0	13.4	91.9
1993–1997	5.4	27.3	128.2	2.4	51.5	140.8	2.0	10.6	100.4	0.2	12.8	99.0
1998–2002	6.4	37.3	117.5	0.4	46.6	83.3	2.0	12.5	98.1	0.4	15.6	103.9
2003–2007	6.2	38.9	121.0	1.6	65.8	117.6	2.0	12.8	102.0	0.2	13.6	99.4
2008–2012	3.6	34.2	98.4	0.2	43.2	89.0	0.8	12.4	91.0	0.0	13.9	93.2
	15–24			55–64			15–24			55–64		
1983–1987	0.6	18.1	129.7	1.8	55.1	129.7	0.0	6.5	95.8	0.2	17.8	90.4
1988–1992	0.8	13.1	132.7	0.6	34.4	97.0	0.2	5.9	112.6	0.4	17.8	100.0
1993–1997	0.0	10.8	95.1	1.2	45.9	114.0	0.0	5.0	95.9	0.0	13.7	86.3
1998–2002	0.2	16.0	101.5	2.0	77.0	121.1	0.2	7.6	108.8	0.0	17.1	90.7
2003–2007	0.4	19.0	109.0	1.2	58.8	101.8	0.0	8.6	95.2	0.4	17.2	102.7
2008–2012	0.4	23.2	109.2	1.4	51.7	106.5	0.0	9.3	96.0	0.4	15.4	103.4
	25–34			65–74			25–34			65–74		
1983–1987	1.0	31.1	121.6	0.6	44.1	100.6	0.0	9.7	90.1	0.6	31.5	100.7
1988–1992	0.2	18.7	97.6	1.0	42.7	121.3	0.2	9.1	105.5	0.4	24.2	92.7
1993–1997	0.2	19.5	99.9	0.6	33.2	104.1	0.0	7.9	95.2	0.4	19.2	98.5
1998–2002	0.4	28.0	103.5	1.0	48.3	106.2	0.0	11.1	95.4	0.4	20.6	97.9
2003–2007	0.6	33.6	108.3	1.0	47.4	110.5	0.2	13.4	103.2	0.2	17.5	93.9
2008–2012	0.2	31.7	97.4	0.2	35.6	90.1	0.0	13.7	95.4	0.2	17.3	96.9
	35–44			>74 y/o			35–44			>74 y/o		
1983–1987	1.0	35.6	111.9	1.0	89.2	113.9	0.0	10.8	91.3	1.4	76.6	132.7
1988–1992	0.6	24.7	104.2	1.2	80.9	125.4	0.4	10.6	109.7	0.6	41.0	85.7
1993–1997	0.4	24.1	98.3	0.6	52.2	100.2	0.2	8.8	103.9	1.2	44.7	130.0
1998–2002	0.8	38.9	110.4	1.6	73.3	139.1	0.0	9.6	93.0	1.0	35.1	113.2
2003–2007	0.6	42.5	106.2	0.8	47.5	105.6	0.2	13.0	103.0	0.6	21.9	97.5
2008–2012	0.6	39.1	105.0	0.6	40.5	97.6	0.0	13.0	95.7	0.2	15.4	80.2

Secondary Medical Zone ID: 243

	Male						Female					
	Suicide			Suicide			Suicide			Suicide		
	Num per year	Rate /100,000	×100	Num per year	Rate /100,000	×100	Num per year	Rate /100,000	×100	Num per year	Rate /100,000	×100
	Total (>10 y/o)			45–54			Total (>10 y/o)			45–54		
1983–1987	105.2	30.7	94.4	26.0	49.1	97.2	50.6	13.0	82.4	9.8	17.4	101.1
1988–1992	82.2	22.5	90.0	19.6	34.0	99.0	47.8	11.0	82.4	7.0	12.3	84.5
1993–1997	80.4	20.9	81.0	23.8	35.7	97.6	42.2	9.4	80.6	6.4	10.1	78.4
1998–2002	123.6	30.7	83.4	31.6	49.5	88.4	53.8	11.6	85.5	10.4	15.5	103.2
2003–2007	129.0	31.3	84.6	25.2	45.6	81.6	42.8	9.7	71.4	4.6	9.3	68.1
2008–2012	129.4	33.1	90.8	26.0	50.0	102.8	50.6	11.1	83.4	6.8	13.1	87.9
	15–24			55–64			15–24			55–64		
1983–1987	7.6	13.7	98.2	16.4	39.6	93.2	4.4	7.6	111.1	7.8	16.8	85.2
1988–1992	6.6	10.2	103.2	14.6	30.2	85.1	2.8	4.6	87.3	8.2	15.7	88.0
1993–1997	8.4	12.3	109.1	13.8	28.5	70.8	3.0	4.8	91.9	7.2	13.4	84.3
1998–2002	7.0	12.6	79.7	31.2	57.0	89.7	3.0	5.7	81.8	11.0	18.5	98.3
2003–2007	8.4	16.2	93.1	32.2	50.9	88.2	2.4	5.6	62.6	8.0	12.9	77.0
2008–2012	11.0	23.0	108.6	26.4	42.6	87.7	4.2	9.2	94.9	8.4	13.2	88.3
	25–34			65–74			25–34			65–74		
1983–1987	11.0	20.9	81.7	8.4	35.4	80.9	5.6	10.0	93.0	6.2	19.9	63.8
1988–1992	10.0	19.3	100.8	7.2	26.8	76.0	5.8	10.1	116.8	6.6	17.9	68.6
1993–1997	9.0	17.2	88.1	10.0	27.9	87.5	4.8	8.4	100.9	9.2	19.9	102.1
1998–2002	13.6	23.4	86.6	15.0	36.2	79.6	7.6	12.0	103.5	9.6	19.0	90.4
2003–2007	16.0	26.9	86.6	16.0	35.9	83.6	6.6	11.1	85.7	7.4	14.6	78.6
2008–2012	16.8	31.4	96.5	12.6	27.4	69.4	5.0	10.4	72.8	9.0	16.3	91.5
	35–44			>74 y/o			35–44			>74 y/o		
1983–1987	23.8	34.5	108.3	11.2	72.7	92.8	6.4	9.7	81.9	9.8	41.8	72.4
1988–1992	14.4	22.1	93.4	9.8	52.7	81.7	6.8	10.0	104.0	10.2	32.9	68.7
1993–1997	9.8	18.0	73.3	5.4	28.4	54.5	4.6	8.0	94.6	6.4	17.7	51.4
1998–2002	15.6	30.1	85.4	9.2	38.3	72.6	5.8	10.5	101.5	6.4	14.8	47.6
2003–2007	17.6	32.8	81.9	13.4	41.2	91.6	5.8	10.7	85.0	8.0	14.5	64.8
2008–2012	24.6	39.1	105.1	12.0	31.6	76.2	7.6	12.4	91.1	9.6	14.7	76.5

Secondary Medical Zone ID: 244

	Male						Female					
	Suicide			Suicide			Suicide			Suicide		
	Num per year	Rate /100,000	×100	Num per year	Rate /100,000	×100	Num per year	Rate /100,000	×100	Num per year	Rate /100,000	×100
	Total (>10 y/o)			45-54			Total (>10 y/o)			45-54		
1983-1987	79.6	29.4	88.0	21.4	47.4	93.7	36.4	12.4	74.8	6.6	15.2	88.4
1988-1992	63.4	22.0	86.5	16.6	33.7	98.2	35.4	10.5	77.1	6.4	13.4	91.8
1993-1997	70.0	23.0	87.7	19.4	35.5	96.9	34.2	9.3	82.3	6.8	12.4	95.9
1998-2002	95.2	30.3	80.5	26.2	50.8	90.7	36.8	10.5	75.5	7.0	13.7	90.8
2003-2007	101.6	32.9	84.7	21.8	50.1	89.6	37.0	10.8	79.0	5.8	13.2	96.1
2008-2012	94.2	31.5	85.5	15.8	41.8	86.0	39.0	11.2	84.0	5.8	14.6	97.7
	15-24			55-64			15-24			55-64		
1983-1987	7.2	16.9	121.5	11.4	35.9	84.5	3.0	6.7	98.4	4.8	14.4	73.1
1988-1992	5.2	10.8	109.3	11.6	29.9	84.2	1.8	4.0	76.4	6.0	15.2	84.9
1993-1997	4.4	9.9	87.8	12.2	30.0	74.6	2.0	4.3	82.8	7.4	16.5	103.7
1998-2002	4.6	12.3	78.2	23.4	51.3	80.7	1.6	4.7	67.2	6.0	14.0	74.6
2003-2007	8.4	21.3	122.3	22.4	44.1	76.5	3.6	9.2	102.6	7.4	14.4	85.7
2008-2012	7.6	22.5	106.0	21.0	42.5	87.6	2.8	8.6	88.4	7.0	13.7	91.8
	25-34			65-74			25-34			65-74		
1983-1987	11.4	25.9	101.3	3.8	23.7	54.1	5.6	12.2	113.0	4.8	19.6	62.7
1988-1992	7.8	19.6	102.6	4.4	22.6	64.0	2.6	7.1	81.5	6.2	20.9	80.1
1993-1997	8.4	20.0	102.6	6.0	22.6	70.9	3.2	7.7	93.0	4.8	14.5	74.4
1998-2002	10.0	22.5	83.2	11.6	35.2	77.5	5.8	12.0	103.3	5.0	14.1	67.3
2003-2007	12.2	27.0	87.0	12.4	33.8	78.9	4.0	9.8	75.1	5.2	13.7	73.3
2008-2012	13.6	34.0	104.2	9.4	25.3	64.0	4.0	11.4	79.7	7.0	15.9	89.4
	35-44			>74 y/o			35-44			>74 y/o		
1983-1987	13.2	24.0	75.4	10.2	79.8	101.9	5.6	10.2	86.6	6.0	32.3	56.0
1988-1992	10.8	20.7	87.5	6.8	46.4	71.9	4.2	8.4	86.7	8.2	32.3	67.4
1993-1997	12.4	27.1	110.6	6.8	41.2	79.1	2.2	5.9	69.1	7.8	25.4	73.8
1998-2002	12.6	31.6	89.7	6.4	34.5	65.4	3.8	9.5	92.2	7.4	20.4	65.9
2003-2007	16.4	39.0	97.4	7.4	30.7	68.3	4.2	10.7	84.9	6.8	15.6	69.7
2008-2012	16.8	35.5	95.4	9.8	32.1	77.3	4.8	11.1	81.7	7.6	14.9	77.6

Secondary Medical Zone ID: 245

	Male						Female					
	Suicide			Suicide			Suicide			Suicide		
	Num per year	Rate /100,000	×100	Num per year	Rate /100,000	×100	Num per year	Rate /100,000	×100	Num per year	Rate /100,000	×100
	Total (>10 y/o)			45–54			Total (>10 y/o)			45–54		
1983–1987	12.4	31.7	94.5	2.4	44.3	87.7	12.0	17.9	132.0	1.0	16.4	95.3
1988–1992	6.4	21.0	71.6	1.6	33.1	96.3	8.4	13.4	110.6	0.8	14.5	99.3
1993–1997	11.2	26.5	106.4	1.0	30.4	83.0	5.2	10.4	91.9	0.6	12.6	97.6
1998–2002	15.0	37.1	104.8	3.4	58.4	104.4	7.2	12.7	105.4	0.6	14.4	95.6
2003–2007	13.0	37.0	99.0	2.2	49.9	89.2	4.4	11.8	88.9	0.0	11.0	80.2
2008–2012	14.2	36.5	115.1	2.2	51.0	104.8	5.4	13.2	102.2	0.8	16.0	107.4
	15–24			55–64			15–24			55–64		
1983–1987	0.6	14.2	101.5	2.6	41.0	96.5	0.2	6.4	94.0	2.0	22.4	113.7
1988–1992	0.4	9.7	98.3	2.0	32.5	91.4	0.2	5.1	96.4	2.0	21.1	117.9
1993–1997	1.0	13.1	116.0	1.6	34.9	86.6	0.0	4.3	82.0	1.8	19.9	125.4
1998–2002	0.6	15.0	94.8	3.0	62.9	99.0	0.0	5.8	82.0	1.6	21.3	113.4
2003–2007	1.4	20.6	118.1	2.8	56.9	98.5	0.0	7.0	77.6	0.6	15.7	93.6
2008–2012	1.0	23.1	108.9	3.6	55.0	113.2	0.2	9.0	93.2	0.6	14.3	95.9
	25–34			65–74			25–34			65–74		
1983–1987	1.0	23.6	92.1	1.6	41.4	94.5	1.0	13.2	123.1	1.8	32.3	103.3
1988–1992	0.0	12.7	66.6	0.8	26.5	75.2	0.4	8.9	102.6	1.4	24.4	93.4
1993–1997	1.2	23.3	119.5	1.6	30.1	94.4	0.2	8.0	95.9	1.2	18.5	94.7
1998–2002	1.2	29.1	107.8	2.6	45.1	99.1	0.0	10.0	86.7	0.8	16.9	80.7
2003–2007	1.2	32.7	105.3	2.0	41.5	96.8	0.4	13.1	100.8	0.6	15.5	83.4
2008–2012	1.0	33.1	101.7	1.8	40.7	103.1	0.4	14.6	101.6	1.2	19.1	107.5
	35–44			>74 y/o			35–44			>74 y/o		
1983–1987	1.8	32.6	102.4	2.4	84.8	108.2	1.0	13.3	112.7	5.0	109.7	190.2
1988–1992	0.6	18.6	78.5	1.0	45.3	70.3	0.6	10.0	103.7	3.0	57.3	119.8
1993–1997	2.8	37.3	152.3	2.0	55.2	106.1	0.2	7.6	89.7	1.2	24.6	71.5
1998–2002	2.2	42.4	120.5	1.8	47.3	89.7	1.0	13.2	127.5	3.2	40.8	131.4
2003–2007	2.0	48.0	119.9	1.4	35.3	78.4	0.6	13.5	107.1	2.2	25.1	111.8
2008–2012	1.8	41.6	111.9	2.8	46.1	111.1	0.6	14.4	106.1	1.6	18.2	94.4

Secondary Medical Zone ID: 246

	Male						Female					
	Suicide			Suicide			Suicide			Suicide		
	Num per year	Rate /100,000	×100	Num per year	Rate /100,000	×100	Num per year	Rate /100,000	×100	Num per year	Rate /100,000	×100
	Total (>10 y/o)			45–54			Total (>10 y/o)			45–54		
1983–1987	11.2	35.5	116.3	3.2	62.8	124.2	5.6	15.2	101.1	0.8	17.3	100.6
1988–1992	10.2	27.4	128.1	1.8	39.9	116.2	6.6	13.7	118.9	1.2	17.3	118.5
1993–1997	6.6	24.5	95.6	1.4	36.5	99.7	4.8	11.1	108.0	1.0	15.1	117.1
1998–2002	13.4	39.0	123.0	4.0	71.4	127.5	4.0	12.1	94.0	0.4	14.2	94.5
2003–2007	11.0	36.3	111.9	1.6	50.2	89.7	4.2	12.9	101.8	0.6	14.2	103.7
2008–2012	11.4	37.6	120.4	2.2	56.2	115.6	1.4	12.0	79.9	0.4	14.7	98.6
	15–24			55–64			15–24			55–64		
1983–1987	1.0	19.0	136.3	1.8	42.3	99.6	0.2	7.0	101.8	0.8	18.5	94.0
1988–1992	0.0	8.8	88.7	3.0	48.2	135.8	0.0	4.7	89.5	1.6	21.7	121.4
1993–1997	0.0	9.9	88.0	1.6	40.4	100.3	0.0	4.7	89.5	1.0	17.7	111.2
1998–2002	0.4	15.8	100.2	2.6	68.4	107.7	0.0	6.3	90.0	0.6	18.2	96.8
2003–2007	0.2	16.5	94.5	2.6	62.3	107.8	0.8	12.6	140.6	1.0	18.3	109.2
2008–2012	1.2	27.0	127.2	1.8	47.0	96.8	0.0	8.7	89.4	0.2	13.3	89.5
	25–34			65–74			25–34			65–74		
1983–1987	1.6	31.3	122.3	0.6	35.6	81.3	0.2	9.8	90.9	1.8	38.7	124.0
1988–1992	1.0	23.0	120.5	1.4	39.4	111.8	0.2	8.4	97.2	1.4	29.6	113.4
1993–1997	0.2	17.3	88.6	1.2	32.0	100.2	0.4	9.2	110.5	0.8	18.5	94.7
1998–2002	0.4	25.1	93.0	2.0	47.1	103.7	0.0	10.4	89.3	0.8	19.6	93.2
2003–2007	1.4	36.2	116.7	2.2	50.2	116.9	0.2	12.5	96.1	0.2	14.9	80.2
2008–2012	1.4	37.1	113.8	2.6	52.2	132.1	0.0	12.8	89.2	0.2	15.5	87.0
	35–44			>74 y/o			35–44			>74 y/o		
1983–1987	2.2	40.3	126.5	0.8	60.8	77.6	0.6	12.5	105.6	1.2	49.4	85.7
1988–1992	1.0	23.7	100.0	2.0	78.3	121.4	0.6	10.5	108.8	1.6	47.4	99.1
1993–1997	0.8	23.6	96.2	1.4	56.2	107.9	0.2	8.0	94.1	1.4	34.7	100.8
1998–2002	1.6	40.7	115.5	2.4	69.1	131.1	0.4	10.8	104.4	1.8	34.6	111.6
2003–2007	0.2	29.4	73.5	2.8	63.4	140.7	0.2	12.1	95.6	1.2	21.5	95.6
2008–2012	1.4	40.6	109.1	0.8	32.8	79.2	0.4	13.9	102.5	0.2	11.5	59.7

Secondary Medical Zone ID: 247

	Male						Female					
	Suicide			Suicide			Suicide			Suicide		
	Num per year	Rate /100,000	×100	Num per year	Rate /100,000	×100	Num per year	Rate /100,000	×100	Num per year	Rate /100,000	×100
	Total (>10 y/o)			45–54			Total (>10 y/o)			45–54		
1983–1987	36.0	37.3	118.0	8.0	56.7	112.1	17.6	15.0	97.2	5.0	25.6	148.5
1988–1992	25.4	25.9	105.7	5.2	38.8	112.9	15.8	12.4	95.8	2.4	16.0	109.3
1993–1997	26.4	26.7	106.5	6.2	40.8	111.6	13.2	10.4	94.4	1.6	12.3	95.4
1998–2002	42.8	40.4	119.6	10.8	67.8	121.1	15.6	12.5	98.5	4.2	21.2	140.7
2003–2007	37.0	39.0	107.9	9.4	69.1	123.5	14.8	12.5	101.8	1.6	13.2	96.5
2008–2012	31.0	35.1	101.6	5.8	51.5	105.9	9.8	11.3	82.3	1.0	12.5	84.0
	15–24			55–64			15–24			55–64		
1983–1987	2.8	20.7	148.7	4.8	37.7	88.6	0.4	5.7	83.0	2.2	16.1	81.6
1988–1992	1.2	10.0	100.6	6.6	42.7	120.2	0.0	3.3	63.2	2.2	15.5	86.7
1993–1997	1.4	11.4	100.9	5.2	40.1	99.5	0.0	3.3	63.1	1.4	12.4	78.1
1998–2002	3.2	21.3	135.0	6.8	58.0	91.3	0.8	7.1	101.7	2.0	17.1	90.7
2003–2007	2.4	20.3	116.1	8.0	58.1	100.6	0.8	8.8	97.9	2.8	18.1	108.1
2008–2012	1.4	19.5	92.1	8.6	55.1	113.5	0.6	8.8	91.2	1.4	12.8	85.7
	25–34			65–74			25–34			65–74		
1983–1987	5.6	37.4	146.3	3.6	42.8	97.7	1.0	9.5	88.3	4.4	35.5	113.6
1988–1992	2.0	18.5	96.8	2.6	29.9	84.7	2.0	12.1	139.8	3.0	23.5	90.0
1993–1997	2.4	21.2	108.7	3.2	28.7	90.0	1.4	10.0	120.4	3.6	22.3	114.7
1998–2002	4.0	31.2	115.6	6.4	49.1	107.9	0.4	8.8	75.8	2.6	18.4	87.4
2003–2007	3.0	28.7	92.5	3.8	36.2	84.4	1.0	11.6	89.0	3.0	20.1	107.8
2008–2012	4.0	36.4	111.7	2.2	28.7	72.6	0.8	12.3	86.2	1.4	14.8	83.2
	35–44			>74 y/o			35–44			>74 y/o		
1983–1987	7.0	42.9	134.8	4.2	75.8	96.8	1.2	10.4	88.4	3.4	42.5	73.7
1988–1992	4.8	29.3	123.4	3.0	50.9	79.0	1.6	10.3	106.4	4.4	41.6	87.0
1993–1997	5.2	34.1	139.3	2.6	40.3	77.4	1.2	8.8	103.5	4.0	31.8	92.4
1998–2002	5.6	44.0	124.8	6.0	66.7	126.6	1.0	9.7	93.4	4.4	29.2	94.1
2003–2007	4.8	44.3	110.5	5.6	51.9	115.2	1.2	12.2	96.4	4.4	23.7	105.8
2008–2012	4.0	37.1	99.8	5.0	42.5	102.4	0.8	11.5	84.7	3.8	18.8	97.6

Secondary Medical Zone ID: 248

	Male						Female					
	Suicide			Suicide			Suicide			Suicide		
	Num per year	Rate /100,000	×100	Num per year	Rate /100,000	×100	Num per year	Rate /100,000	×100	Num per year	Rate /100,000	×100
	Total (>10 y/o)			45–54			Total (>10 y/o)			45–54		
1983–1987	139.8	29.9	90.3	36.6	48.8	96.5	67.8	13.3	85.6	11.8	15.8	91.8
1988–1992	116.2	22.5	90.5	23.6	27.5	80.2	69.0	11.7	90.8	14.2	16.0	109.3
1993–1997	133.4	23.4	93.4	36.4	35.8	97.8	59.6	9.4	84.6	13.6	13.3	103.2
1998–2002	195.0	32.3	90.2	50.6	51.5	92.0	73.0	10.9	84.1	15.2	15.3	101.7
2003–2007	204.8	34.0	92.1	46.4	55.2	98.6	75.8	11.1	87.8	11.6	13.6	99.4
2008–2012	187.0	31.4	90.2	31.4	40.6	83.5	80.4	12.4	91.9	11.8	14.7	98.9
	15–24			55–64			15–24			55–64		
1983–1987	8.0	9.5	67.9	21.8	42.0	98.8	5.8	6.7	97.8	10.6	18.1	91.5
1988–1992	8.2	8.3	84.2	21.4	34.5	97.2	5.0	5.0	94.5	9.8	15.1	84.7
1993–1997	11.0	11.1	98.0	27.0	38.9	96.6	4.0	4.2	80.6	9.2	13.1	82.2
1998–2002	10.8	13.3	84.1	47.2	59.1	93.0	5.2	6.3	89.8	15.8	18.9	100.4
2003–2007	11.2	15.8	90.7	45.8	49.4	85.6	3.4	5.4	60.5	14.4	15.1	90.3
2008–2012	13.8	21.3	100.6	40.2	44.0	90.5	5.8	9.1	94.0	13.8	14.5	96.9
	25–34			65–74			25–34			65–74		
1983–1987	22.0	25.1	98.1	9.8	34.6	79.1	7.2	8.5	78.7	11.6	29.7	95.0
1988–1992	17.2	20.1	104.9	11.4	33.1	93.8	4.6	5.9	68.4	12.4	26.4	101.4
1993–1997	17.2	19.0	97.0	13.0	29.9	93.9	6.8	7.5	90.7	8.4	15.7	80.3
1998–2002	22.0	22.5	83.3	22.8	43.7	96.0	9.2	9.4	80.9	11.0	18.0	85.7
2003–2007	25.2	26.7	86.2	22.8	38.5	89.8	8.4	9.1	70.2	12.6	18.4	98.6
2008–2012	24.6	30.8	94.5	24.0	35.3	89.3	11.6	14.0	97.4	11.0	14.7	82.9
	35–44			>74 y/o			35–44			>74 y/o		
1983–1987	28.6	26.8	84.3	12.2	77.5	99.0	10.2	9.8	83.0	10.4	43.5	75.3
1988–1992	22.2	21.7	91.3	12.2	60.1	93.2	10.6	10.2	105.4	12.0	35.8	74.9
1993–1997	18.6	21.4	87.3	10.2	42.8	82.3	6.6	7.6	89.8	11.0	25.8	75.1
1998–2002	24.8	30.1	85.3	16.4	54.2	102.9	6.2	7.9	76.1	10.4	19.6	63.2
2003–2007	36.6	41.0	102.2	16.6	42.9	95.2	11.8	13.0	103.0	13.6	20.0	89.3
2008–2012	33.0	33.2	89.1	20.0	42.4	102.1	14.8	14.3	105.4	11.6	14.7	76.5

Secondary Medical Zone ID: 249

	Male						Female					
	Suicide			Suicide			Suicide			Suicide		
	Num per year	Rate /100,000	×100	Num per year	Rate /100,000	×100	Num per year	Rate /100,000	×100	Num per year	Rate /100,000	×100
	Total (>10 y/o)			45–54			Total (>10 y/o)			45–54		
1983–1987	15.8	32.5	98.5	3.8	50.1	99.0	7.0	14.1	87.1	1.0	15.4	89.6
1988–1992	14.0	24.5	100.2	2.6	31.7	92.3	9.6	13.1	107.1	2.2	17.5	120.0
1993–1997	15.6	25.0	98.3	3.4	33.6	91.9	7.4	10.4	92.2	1.6	13.5	104.2
1998–2002	21.6	34.0	91.0	6.2	54.8	97.9	9.6	12.4	95.6	1.6	14.5	96.5
2003–2007	18.2	31.2	77.3	4.6	49.2	88.0	7.6	11.4	84.6	1.2	12.8	93.7
2008–2012	21.4	33.4	95.0	3.6	45.5	93.7	8.0	12.2	88.9	1.2	14.2	95.4
	15–24			55–64			15–24			55–64		
1983–1987	1.4	16.3	116.8	2.2	38.2	90.0	0.0	5.0	73.3	1.8	21.7	110.1
1988–1992	1.0	10.4	105.4	2.4	33.4	94.0	1.0	6.9	130.5	1.0	15.6	87.5
1993–1997	1.0	11.1	98.2	4.0	45.4	112.7	0.8	6.1	116.5	2.0	18.7	117.5
1998–2002	2.4	20.2	127.6	4.0	51.8	81.5	1.2	8.8	125.4	1.8	18.8	99.6
2003–2007	1.0	16.0	91.8	4.2	45.2	78.3	0.2	6.7	74.4	1.8	16.5	98.3
2008–2012	1.6	22.4	105.5	4.6	43.9	90.5	0.2	7.4	76.8	1.0	12.6	84.2
	25–34			65–74			25–34			65–74		
1983–1987	1.6	23.0	89.8	2.0	48.7	111.2	0.6	9.4	87.7	1.0	26.7	85.4
1988–1992	1.4	18.8	98.5	1.4	32.8	93.2	0.8	8.9	103.2	1.8	28.4	108.9
1993–1997	1.2	17.5	89.5	1.0	23.7	74.4	0.4	7.1	85.8	0.8	15.1	77.7
1998–2002	2.4	27.2	100.8	2.8	42.8	94.1	0.4	9.2	79.6	1.8	21.4	101.7
2003–2007	2.4	29.7	95.7	2.4	37.4	87.1	1.2	13.1	100.4	1.2	16.6	89.3
2008–2012	2.2	31.2	95.9	3.6	41.7	105.4	1.0	13.9	97.2	1.8	18.5	103.9
	35–44			>74 y/o			35–44			>74 y/o		
1983–1987	3.0	30.4	95.5	1.8	83.4	106.4	1.0	11.1	93.9	1.6	54.7	94.8
1988–1992	2.4	22.7	95.6	2.8	86.3	133.8	1.0	9.5	98.2	1.8	44.3	92.6
1993–1997	3.4	30.0	122.3	1.6	49.9	95.8	0.6	7.5	87.8	1.2	25.5	74.1
1998–2002	2.2	29.6	84.1	1.6	44.5	84.3	1.0	10.4	101.0	1.8	26.7	86.1
2003–2007	1.4	25.6	63.9	2.2	42.3	93.9	0.6	10.7	84.4	1.4	17.5	77.8
2008–2012	3.0	35.5	95.4	2.8	42.1	101.6	1.8	15.3	113.0	1.0	13.1	68.0

Secondary Medical Zone ID: 250

	Male						Female					
	Suicide			Suicide			Suicide			Suicide		
	Num per year	Rate /100,000	×100	Num per year	Rate /100,000	×100	Num per year	Rate /100,000	×100	Num per year	Rate /100,000	×100
	Total (>10 y/o)			45–54			Total (>10 y/o)			45–54		
1983–1987	43.6	32.1	97.2	11.0	50.2	99.3	24.8	14.6	94.8	3.6	16.1	93.2
1988–1992	34.8	24.8	97.6	8.4	36.3	105.6	17.8	11.1	78.2	3.4	14.5	99.1
1993–1997	35.6	25.1	96.9	11.6	44.7	122.2	16.2	10.1	82.9	3.4	13.4	103.9
1998–2002	50.4	35.7	97.4	13.0	58.9	105.3	17.6	11.4	81.6	3.4	15.4	102.3
2003–2007	50.2	37.6	101.2	10.2	59.1	105.7	18.2	12.7	91.6	3.4	16.8	122.3
2008–2012	41.0	33.3	94.1	7.8	52.0	107.1	15.0	12.2	84.7	2.8	17.0	113.7
	15–24			55–64			15–24			55–64		
1983–1987	2.8	14.2	101.8	7.8	43.0	101.3	1.2	6.3	92.3	4.4	20.1	101.6
1988–1992	2.0	9.5	95.8	6.2	32.0	90.2	1.0	5.0	94.7	3.8	17.2	96.1
1993–1997	2.6	12.1	107.4	6.2	33.4	83.0	1.0	5.3	100.9	2.4	12.6	79.0
1998–2002	1.6	12.8	81.1	12.0	58.9	92.6	1.0	6.9	98.7	4.0	18.2	96.5
2003–2007	2.4	17.5	100.1	14.6	64.1	111.0	1.2	9.3	103.5	3.0	14.5	86.7
2008–2012	2.0	18.9	89.1	9.8	48.4	99.6	0.6	7.8	80.5	2.4	13.1	88.0
	25–34			65–74			25–34			65–74		
1983–1987	6.2	31.4	122.8	4.0	35.9	81.9	1.8	10.1	93.6	6.2	36.4	116.3
1988–1992	3.8	21.5	112.5	3.0	26.4	75.0	1.4	8.5	98.1	2.8	17.9	68.6
1993–1997	4.2	22.5	115.0	3.0	23.0	72.0	1.0	7.3	87.2	1.8	11.8	60.5
1998–2002	4.2	24.4	90.3	6.4	40.5	89.1	1.8	11.1	95.5	3.0	16.4	78.1
2003–2007	5.0	29.6	95.5	6.0	37.1	86.5	1.4	11.1	85.2	2.8	15.4	82.5
2008–2012	4.0	30.0	92.2	5.2	32.0	81.1	1.6	13.7	95.8	2.0	13.1	73.4
	35–44			>74 y/o			35–44			>74 y/o		
1983–1987	9.0	34.2	107.3	2.8	49.6	63.3	3.2	11.9	100.9	4.2	40.9	70.9
1988–1992	8.0	31.8	134.3	3.4	45.7	70.8	2.4	10.1	104.6	3.0	24.5	51.3
1993–1997	4.4	24.3	99.1	3.4	39.4	75.6	3.2	13.3	156.8	3.4	22.2	64.4
1998–2002	7.4	43.7	124.2	5.8	53.3	101.1	0.8	7.9	76.3	3.6	20.0	64.3
2003–2007	7.6	46.6	116.4	4.4	36.7	81.5	2.8	15.3	120.8	3.6	16.7	74.4
2008–2012	6.2	36.7	98.5	6.0	40.9	98.6	2.4	14.2	104.7	3.2	13.8	71.8

Secondary Medical Zone ID: 251

	Male						Female					
	Suicide			Suicide			Suicide			Suicide		
	Num per year	Rate /100,000	×100	Num per year	Rate /100,000	×100	Num per year	Rate /100,000	×100	Num per year	Rate /100,000	×100
	Total (>10 y/o)			45-54			Total (>10 y/o)			45-54		
1983-1987	26.0	34.2	107.2	6.8	57.7	114.1	12.0	14.5	91.5	1.4	15.0	87.2
1988-1992	23.6	26.7	112.7	4.4	34.4	100.1	15.4	13.5	113.8	1.2	12.6	86.2
1993-1997	25.6	26.0	107.8	6.0	38.1	104.1	13.6	11.4	109.4	2.0	13.1	101.2
1998-2002	33.4	34.1	94.1	9.4	59.6	106.5	14.0	12.2	96.7	2.2	14.8	98.1
2003-2007	34.0	34.6	91.0	7.6	56.5	101.0	12.0	11.7	88.5	0.8	10.5	76.4
2008-2012	34.2	33.9	95.8	5.8	46.8	96.4	12.6	12.5	92.4	2.2	16.0	107.5
	15-24			55-64			15-24			55-64		
1983-1987	2.0	16.4	117.5	4.8	48.0	113.0	0.4	5.9	86.6	2.0	18.9	95.8
1988-1992	1.8	10.9	110.6	5.4	44.8	126.3	0.8	5.7	107.6	2.8	20.6	115.6
1993-1997	2.6	12.4	110.1	5.6	45.8	113.7	1.0	5.8	111.1	2.8	19.7	123.6
1998-2002	1.4	11.1	70.5	8.2	63.7	100.2	0.6	5.8	82.8	3.0	20.6	109.5
2003-2007	2.4	14.7	84.2	7.0	49.1	85.1	1.2	8.8	97.9	2.2	15.6	93.4
2008-2012	3.0	18.9	89.3	6.2	43.6	89.8	1.2	9.7	99.7	2.2	14.6	97.8
	25-34			65-74			25-34			65-74		
1983-1987	2.6	25.2	98.5	2.4	41.3	94.4	0.4	7.8	72.4	1.8	25.5	81.7
1988-1992	2.2	20.0	104.8	2.0	31.7	89.9	1.0	8.9	102.6	2.2	24.4	93.4
1993-1997	2.8	21.3	109.0	3.2	36.1	113.3	0.2	5.8	69.7	3.0	24.8	127.2
1998-2002	2.6	22.3	82.6	4.2	45.0	99.0	1.6	11.7	101.3	2.2	20.2	96.4
2003-2007	4.6	30.9	99.5	4.6	44.4	103.5	2.0	13.9	106.7	1.0	13.7	73.7
2008-2012	5.2	35.6	109.2	4.4	38.7	97.9	2.0	15.4	107.5	1.6	15.6	87.6
	35-44			>74 y/o			35-44			>74 y/o		
1983-1987	5.2	34.1	107.2	1.8	60.0	76.6	2.8	15.0	127.0	3.2	58.4	101.2
1988-1992	5.0	30.5	128.8	2.8	62.7	97.3	2.4	12.4	128.6	4.8	60.1	125.6
1993-1997	2.6	21.6	88.2	2.8	53.6	102.9	1.6	10.1	119.1	3.0	32.4	94.1
1998-2002	3.6	30.8	87.5	3.8	58.9	111.7	0.8	8.6	83.5	3.6	31.4	101.1
2003-2007	5.0	37.6	93.8	2.8	39.9	88.6	1.6	12.4	98.4	3.2	23.2	103.3
2008-2012	6.8	41.2	110.9	2.6	33.7	81.3	0.8	10.3	75.8	2.4	16.7	87.1

Secondary Medical Zone ID: 252

	Male						Female					
	Suicide			Suicide			Suicide			Suicide		
	Num per year	Rate /100,000	×100	Num per year	Rate /100,000	×100	Num per year	Rate /100,000	×100	Num per year	Rate /100,000	×100
	Total (>10 y/o)			45–54			Total (>10 y/o)			45–54		
1983–1987	49.6	36.7	115.0	10.8	52.8	104.4	30.4	17.2	118.4	4.8	20.1	116.4
1988–1992	41.0	28.6	119.8	7.6	36.9	107.4	25.6	13.6	110.4	5.2	19.8	135.2
1993–1997	40.4	28.0	114.9	10.8	45.3	123.8	20.0	10.9	101.6	3.0	13.2	102.1
1998–2002	54.4	38.3	109.1	13.8	62.1	111.0	25.6	12.9	112.1	2.4	13.0	86.4
2003–2007	52.0	38.6	107.4	11.0	59.6	106.6	23.0	13.2	110.9	2.6	14.0	102.2
2008–2012	48.8	37.8	111.9	8.0	51.8	106.6	19.4	12.6	101.8	3.4	18.4	123.3
	15–24			55–64			15–24			55–64		
1983–1987	3.8	20.8	149.2	8.6	45.7	107.6	1.4	7.3	107.4	5.2	22.3	113.0
1988–1992	2.0	11.2	112.8	9.2	44.0	123.8	1.4	6.4	121.9	4.4	18.9	105.6
1993–1997	2.6	13.8	122.2	8.2	42.6	105.7	1.0	5.5	106.2	3.6	16.5	103.8
1998–2002	3.4	20.3	128.6	12.8	66.5	104.7	0.8	6.5	92.3	4.6	20.7	109.9
2003–2007	2.6	19.4	111.3	13.2	61.4	106.4	1.2	9.4	104.8	4.2	18.0	107.5
2008–2012	3.0	24.6	116.1	10.0	47.6	97.9	0.4	7.2	73.9	3.2	14.9	100.0
	25–34			65–74			25–34			65–74		
1983–1987	5.4	28.2	110.4	4.2	38.3	87.5	2.8	13.1	121.4	4.8	31.6	101.3
1988–1992	6.0	32.7	171.3	3.6	29.2	82.9	1.2	8.1	93.6	4.4	25.1	96.1
1993–1997	4.8	28.3	144.9	4.6	30.0	93.9	1.4	8.8	105.4	4.8	22.6	116.0
1998–2002	6.4	36.0	133.5	9.0	51.1	112.3	2.2	12.8	110.1	5.4	24.2	115.2
2003–2007	2.4	22.1	71.3	8.4	48.8	113.8	1.2	10.9	83.7	4.0	19.4	104.4
2008–2012	4.4	33.6	103.3	7.2	42.1	106.7	1.2	12.6	88.0	4.4	20.6	115.5
	35–44			>74 y/o			35–44			>74 y/o		
1983–1987	10.0	40.0	125.6	6.8	96.3	122.9	2.8	11.9	100.5	8.4	77.4	134.1
1988–1992	5.8	25.4	107.2	6.4	74.3	115.2	1.0	7.1	73.6	7.8	56.2	117.4
1993–1997	4.4	24.0	98.0	5.0	52.0	99.9	1.2	7.5	88.0	5.0	31.0	90.1
1998–2002	4.0	28.7	81.6	5.0	45.5	86.3	1.4	9.6	92.8	8.8	42.1	135.7
2003–2007	7.8	50.9	127.0	6.6	46.8	104.0	2.6	14.8	117.1	7.2	29.0	129.0
2008–2012	8.4	47.4	127.4	7.8	47.3	114.0	1.2	11.1	82.0	5.6	20.5	106.8

Secondary Medical Zone ID: 253

	Male						Female					
	Suicide			Suicide			Suicide			Suicide		
	Num per year	Rate /100,000	×100	Num per year	Rate /100,000	×100	Num per year	Rate /100,000	×100	Num per year	Rate /100,000	×100
	Total (>10 y/o)			45–54			Total (>10 y/o)			45–54		
1983–1987	64.6	32.2	98.2	20.4	61.6	121.8	39.2	16.2	108.6	5.8	17.3	100.6
1988–1992	48.4	22.9	90.9	11.2	32.0	93.2	29.0	11.7	88.3	5.2	14.5	98.9
1993–1997	53.4	23.6	91.6	16.2	39.5	108.0	28.6	10.4	95.2	5.8	13.8	107.2
1998–2002	81.4	34.0	93.3	22.2	56.0	100.1	32.0	11.6	89.7	5.4	14.0	93.2
2003–2007	85.6	35.5	97.2	19.4	57.3	102.4	30.8	11.5	89.8	4.6	13.5	98.3
2008–2012	78.2	35.0	97.6	16.0	53.8	110.7	33.8	13.0	98.9	4.0	13.9	93.2
	15–24			55–64			15–24			55–64		
1983–1987	1.6	7.6	54.1	9.2	38.5	90.6	2.2	6.9	101.2	11.2	34.3	174.0
1988–1992	2.8	8.9	89.9	8.6	31.2	87.9	2.0	5.6	105.4	3.2	12.6	70.6
1993–1997	1.8	7.3	64.6	10.0	34.3	85.1	1.4	4.5	86.7	4.0	13.2	83.3
1998–2002	5.0	16.2	102.3	20.2	60.7	95.6	2.4	7.7	110.3	3.8	13.4	71.0
2003–2007	2.8	13.4	77.1	19.6	52.3	90.6	1.8	8.0	88.8	5.4	14.7	87.6
2008–2012	4.4	20.7	97.7	15.0	41.2	84.9	2.4	10.8	111.6	6.4	15.7	105.3
	25–34			65–74			25–34			65–74		
1983–1987	8.2	24.0	93.7	5.0	37.3	85.2	3.4	10.0	92.5	4.8	26.7	85.6
1988–1992	7.2	22.7	118.9	2.8	20.8	59.1	2.6	8.4	96.6	5.6	25.5	97.7
1993–1997	7.2	22.5	115.0	4.2	22.2	69.7	2.2	7.4	89.0	4.4	17.4	89.4
1998–2002	9.8	28.1	104.2	10.8	44.5	97.8	3.2	10.0	86.5	7.6	24.9	118.5
2003–2007	10.8	31.6	101.8	12.0	44.7	104.1	4.0	12.2	94.0	6.6	20.7	110.9
2008–2012	8.8	31.4	96.4	11.2	38.4	97.1	3.8	13.9	96.9	5.4	16.8	94.6
	35–44			>74 y/o			35–44			>74 y/o		
1983–1987	12.8	30.5	95.8	7.4	88.6	113.1	4.0	10.2	86.0	7.8	59.6	103.4
1988–1992	11.6	27.5	115.9	4.0	44.1	68.4	4.2	10.1	104.1	5.8	35.6	74.3
1993–1997	8.8	24.9	101.7	5.2	45.2	86.9	3.6	9.6	113.3	7.2	34.7	100.9
1998–2002	8.8	29.6	84.0	4.4	33.8	64.1	2.8	9.4	90.8	6.6	26.1	83.9
2003–2007	12.2	39.7	99.1	8.6	45.5	101.1	2.2	9.1	71.7	6.2	19.6	87.2
2008–2012	15.8	44.2	118.8	6.6	31.4	75.8	5.0	14.2	104.6	6.6	17.7	92.3

Secondary Medical Zone ID: 254

	Male						Female					
	Suicide			Suicide			Suicide			Suicide		
	Num per year	Rate /100,000	×100	Num per year	Rate /100,000	×100	Num per year	Rate /100,000	×100	Num per year	Rate /100,000	×100
	Total (>10 y/o)			45–54			Total (>10 y/o)			45–54		
1983–1987	25.8	40.2	138.9	6.8	74.3	146.9	16.4	18.3	137.2	2.0	19.4	112.4
1988–1992	22.4	31.3	149.0	3.0	39.8	115.7	14.8	15.0	136.4	1.2	15.0	102.8
1993–1997	21.6	30.3	142.9	4.0	44.6	122.0	9.8	11.2	113.2	1.2	13.9	107.2
1998–2002	24.4	39.1	121.9	5.2	61.9	110.6	10.0	12.9	107.1	0.2	11.7	77.8
2003–2007	25.6	42.1	133.3	5.8	74.3	132.8	12.0	13.2	129.9	0.8	13.4	97.9
2008–2012	18.8	36.5	112.2	4.2	61.1	125.6	9.2	14.0	113.5	1.4	17.7	118.8
	15–24			55–64			15–24			55–64		
1983–1987	0.6	14.0	100.4	4.0	45.2	106.3	0.4	7.3	106.6	3.8	28.3	143.4
1988–1992	1.0	11.9	119.8	6.2	57.3	161.4	0.4	5.9	111.6	2.8	22.3	124.9
1993–1997	1.4	14.5	128.6	3.4	43.5	108.1	0.0	4.3	81.9	1.4	16.0	100.9
1998–2002	1.6	20.1	127.3	4.6	68.0	107.1	0.0	5.8	83.1	1.4	18.8	99.9
2003–2007	0.4	15.8	90.3	4.8	63.2	109.4	0.2	8.2	91.1	1.6	18.1	107.9
2008–2012	0.4	18.9	89.3	4.8	54.5	112.2	0.0	7.8	80.3	0.4	12.5	83.5
	25–34			65–74			25–34			65–74		
1983–1987	3.2	36.0	140.8	4.2	61.6	140.6	1.4	14.2	132.0	2.8	35.7	114.3
1988–1992	2.8	32.0	167.6	2.0	32.9	93.5	0.2	7.5	86.9	4.6	44.2	169.6
1993–1997	0.8	18.5	94.8	5.0	53.3	167.0	0.2	7.5	90.2	2.0	20.7	106.3
1998–2002	1.4	26.9	99.5	4.8	56.0	123.1	0.0	9.4	80.7	2.4	23.4	111.4
2003–2007	2.4	36.4	117.3	4.2	54.5	127.1	0.6	13.0	99.9	2.4	23.0	123.4
2008–2012	1.4	32.4	99.3	3.2	46.7	118.3	0.6	14.5	101.1	2.4	23.0	129.0
	35–44			>74 y/o			35–44			>74 y/o		
1983–1987	3.8	41.5	130.3	3.2	82.7	105.6	1.4	13.8	117.0	4.6	77.9	135.1
1988–1992	3.2	31.9	134.6	4.2	84.9	131.6	0.8	10.0	103.2	4.8	64.2	134.2
1993–1997	3.2	35.2	143.4	3.8	68.1	130.7	0.6	8.7	102.4	4.4	49.2	143.0
1998–2002	2.4	38.5	109.3	4.4	66.9	127.0	1.6	14.9	144.2	4.2	40.0	128.8
2003–2007	3.0	50.1	125.0	5.0	62.8	139.5	0.2	10.9	86.0	6.2	45.2	201.4
2008–2012	2.2	38.6	103.8	2.6	37.7	90.9	1.6	17.3	127.9	2.6	19.8	102.9

Secondary Medical Zone ID: 255

	Male						Female					
	Suicide			Suicide			Suicide			Suicide		
	Num per year	Rate /100,000	×100	Num per year	Rate /100,000	×100	Num per year	Rate /100,000	×100	Num per year	Rate /100,000	×100
	Total (>10 y/o)			45–54			Total (>10 y/o)			45–54		
1983–1987	22.8	32.2	99.5	4.6	42.5	84.1	12.4	15.1	98.2	2.6	18.7	108.8
1988–1992	23.2	27.6	122.7	5.0	40.6	118.1	11.2	12.3	96.1	1.8	14.6	99.6
1993–1997	21.8	26.7	111.5	3.6	32.6	89.1	12.8	11.8	116.1	2.6	15.8	122.5
1998–2002	29.8	37.3	106.7	7.2	58.2	104.0	12.0	12.5	99.0	2.6	17.5	116.3
2003–2007	29.2	38.3	107.3	7.0	64.9	116.0	14.2	14.1	117.1	1.4	13.7	99.9
2008–2012	28.4	36.7	114.2	6.4	62.8	129.2	10.0	12.9	95.9	0.6	12.1	80.8
	15–24			55–64			15–24			55–64		
1983–1987	0.8	11.7	84.0	3.8	40.5	95.4	0.8	7.3	106.2	1.8	18.1	91.5
1988–1992	0.8	9.5	96.0	5.0	41.8	117.6	0.0	3.6	67.9	2.4	18.8	105.3
1993–1997	1.8	14.0	123.9	4.0	38.2	94.8	0.2	4.3	82.2	2.2	16.9	106.4
1998–2002	0.2	11.4	72.4	6.0	58.8	92.6	0.4	6.5	92.2	2.2	18.5	98.5
2003–2007	1.4	18.7	106.9	6.4	56.2	97.4	0.0	6.2	68.8	1.8	15.8	94.4
2008–2012	0.8	19.0	89.6	7.0	54.8	112.8	0.8	10.9	111.9	1.4	13.8	92.3
	25–34			65–74			25–34			65–74		
1983–1987	3.4	29.8	116.4	2.6	45.0	102.9	1.4	11.9	110.4	1.8	27.6	88.3
1988–1992	1.4	17.3	90.7	4.0	49.6	140.8	0.8	8.7	100.1	2.6	28.4	108.8
1993–1997	2.8	25.5	130.6	3.8	40.9	128.2	0.8	8.5	101.8	2.0	19.6	100.6
1998–2002	3.8	33.4	123.8	4.8	48.8	107.3	1.2	11.9	102.9	2.2	20.2	96.2
2003–2007	2.8	31.4	101.3	4.8	47.9	111.7	1.8	15.5	119.1	3.4	24.1	129.4
2008–2012	1.8	29.5	90.6	3.2	37.2	94.1	1.0	14.1	98.4	2.4	19.3	108.6
	35–44			>74 y/o			35–44			>74 y/o		
1983–1987	4.8	35.6	111.8	2.8	83.8	106.9	0.8	9.4	79.5	3.2	65.8	114.0
1988–1992	3.2	25.0	105.3	3.6	81.2	125.8	0.4	7.3	76.1	3.2	50.3	105.1
1993–1997	3.0	26.9	109.5	2.8	57.7	110.8	1.6	11.0	129.9	3.4	41.0	119.2
1998–2002	4.4	42.9	121.7	3.4	58.0	110.0	0.8	9.7	94.2	2.6	27.4	88.4
2003–2007	3.4	39.9	99.6	3.4	46.7	103.6	2.8	18.8	148.4	2.8	22.9	102.3
2008–2012	4.2	41.3	110.9	5.0	52.5	126.5	1.8	15.4	113.3	2.0	15.7	81.5

Secondary Medical Zone ID: 256

	Male						Female					
	Suicide			Suicide			Suicide			Suicide		
	Num per year	Rate /100,000	×100	Num per year	Rate /100,000	×100	Num per year	Rate /100,000	×100	Num per year	Rate /100,000	×100
	Total (>10 y/o)			45–54			Total (>10 y/o)			45–54		
1983–1987	16.2	33.3	103.5	3.0	46.8	92.6	9.8	15.0	96.3	2.4	21.8	126.5
1988–1992	13.4	26.0	106.2	1.8	30.9	89.9	5.2	10.7	67.5	0.2	11.3	77.4
1993–1997	14.8	27.2	114.8	4.0	45.2	123.4	7.4	10.7	95.7	1.6	15.2	118.0
1998–2002	17.8	37.4	103.5	4.4	60.5	108.0	7.4	12.5	91.7	1.0	14.9	98.8
2003–2007	16.8	37.5	103.2	3.6	60.5	108.2	7.6	12.7	101.6	1.0	14.6	106.4
2008–2012	16.8	36.7	112.4	2.6	51.7	106.5	5.6	12.6	92.2	0.6	14.4	96.3
	15–24			55–64			15–24			55–64		
1983–1987	0.6	13.6	97.9	2.0	34.1	80.2	0.4	7.0	102.1	1.2	16.8	85.2
1988–1992	0.6	10.3	104.1	3.0	37.8	106.5	0.2	5.0	94.2	0.6	13.4	75.0
1993–1997	0.2	9.9	88.0	3.0	42.1	104.6	0.0	4.3	81.9	1.6	17.4	109.4
1998–2002	1.2	18.8	119.0	2.2	47.0	74.0	0.2	6.7	95.2	1.8	20.3	107.7
2003–2007	0.2	15.4	88.3	3.8	54.9	95.1	0.4	9.5	106.0	1.4	17.0	101.8
2008–2012	1.0	23.7	111.9	5.0	58.2	119.8	0.2	9.3	95.7	1.8	17.4	116.5
	25–34			65–74			25–34			65–74		
1983–1987	2.2	31.7	124.1	2.4	45.8	104.7	0.6	10.8	100.2	2.0	29.7	95.0
1988–1992	2.2	30.0	156.9	1.8	33.0	93.7	0.2	7.7	88.9	0.4	13.5	51.8
1993–1997	1.2	22.5	115.1	2.4	34.4	107.9	0.2	7.6	91.4	1.4	17.8	91.3
1998–2002	2.6	36.0	133.2	3.6	49.5	108.9	0.8	12.7	109.5	1.2	17.7	84.1
2003–2007	2.0	35.8	115.6	2.4	40.6	94.6	0.8	14.1	108.4	1.4	18.3	98.4
2008–2012	1.8	37.0	113.5	2.6	40.6	102.8	0.2	12.9	89.8	0.8	15.5	87.3
	35–44			>74 y/o			35–44			>74 y/o		
1983–1987	3.0	36.9	115.9	3.0	88.3	112.7	1.2	13.0	109.8	2.0	42.2	73.2
1988–1992	2.0	26.3	111.0	2.0	56.6	87.7	1.8	13.6	140.7	1.8	30.6	63.9
1993–1997	1.6	26.3	107.5	2.4	54.7	105.0	0.4	8.2	96.0	2.2	28.8	83.6
1998–2002	2.6	44.1	125.2	1.2	35.7	67.6	0.8	11.7	113.5	1.6	20.7	66.5
2003–2007	2.2	45.6	113.7	2.6	45.2	100.4	0.2	11.0	87.5	2.4	22.7	101.0
2008–2012	2.0	38.9	104.7	1.8	34.3	82.7	0.6	13.5	99.2	1.4	15.0	77.8

Secondary Medical Zone ID: 257

	Male						Female					
	Suicide			Suicide			Suicide			Suicide		
	Num per year	Rate /100,000	×100	Num per year	Rate /100,000	×100	Num per year	Rate /100,000	×100	Num per year	Rate /100,000	×100
	Total (>10 y/o)			45–54			Total (>10 y/o)			45–54		
1983–1987	41.0	35.1	111.6	8.4	47.2	93.4	23.2	16.7	115.1	5.6	24.4	141.5
1988–1992	32.4	25.9	109.6	9.8	44.7	130.2	19.0	12.9	102.1	4.4	18.1	123.8
1993–1997	36.0	27.2	112.7	7.0	32.9	89.8	17.6	11.1	104.8	5.4	18.7	144.5
1998–2002	49.8	36.6	107.3	9.6	48.9	87.4	17.0	11.7	89.8	1.8	11.5	76.7
2003–2007	46.4	36.8	102.2	9.2	55.1	98.5	20.8	13.0	110.0	2.6	14.3	104.4
2008–2012	43.0	34.5	103.4	5.8	43.5	89.4	16.4	12.7	94.2	2.2	14.9	99.7
	15–24			55–64			15–24			55–64		
1983–1987	2.6	15.3	109.6	7.8	52.8	124.1	1.4	7.6	111.7	4.2	23.7	120.4
1988–1992	1.2	8.4	85.0	7.2	42.7	120.2	0.6	4.4	84.3	3.0	17.5	97.9
1993–1997	2.2	12.0	105.8	8.4	46.6	115.9	0.8	5.1	97.2	2.4	14.2	89.6
1998–2002	1.8	14.3	90.8	13.4	69.0	108.5	0.2	4.9	70.3	3.0	16.7	89.0
2003–2007	1.8	16.6	95.2	11.6	56.0	97.0	0.6	7.6	84.3	4.4	18.3	109.3
2008–2012	1.2	16.7	78.5	9.8	49.4	101.7	0.4	7.5	77.6	2.6	13.5	90.8
	25–34			65–74			25–34			65–74		
1983–1987	5.8	30.5	119.3	2.8	37.9	86.5	3.0	13.8	128.3	3.8	33.9	108.4
1988–1992	1.4	12.8	67.1	3.6	37.2	105.6	1.6	9.3	107.1	3.4	26.6	101.9
1993–1997	3.0	19.8	101.3	3.8	32.5	101.9	1.2	7.9	95.3	1.4	12.7	65.4
1998–2002	5.0	28.5	105.7	10.0	65.1	143.1	2.2	12.1	104.1	4.2	24.1	114.8
2003–2007	5.8	33.8	109.1	7.4	48.4	112.9	2.2	13.2	101.6	3.6	20.1	107.9
2008–2012	5.6	36.7	112.7	7.0	41.1	104.0	2.0	14.6	101.8	2.8	16.3	91.5
	35–44			>74 y/o			35–44			>74 y/o		
1983–1987	8.6	35.2	110.5	5.0	102.7	131.1	2.8	11.5	97.8	2.4	40.3	69.8
1988–1992	4.2	20.8	87.9	4.8	78.5	121.7	1.8	8.9	91.7	4.2	44.6	93.1
1993–1997	7.0	35.4	144.5	4.4	62.3	119.7	2.2	10.4	122.0	4.2	35.3	102.5
1998–2002	4.6	32.0	90.8	5.4	63.8	121.0	1.8	10.9	105.4	3.8	27.2	87.5
2003–2007	6.4	41.8	104.3	4.2	43.0	95.6	2.4	13.8	109.6	5.0	27.3	121.5
2008–2012	7.0	39.0	104.8	6.6	51.5	124.2	3.6	16.8	123.6	2.8	15.0	78.2

Secondary Medical Zone ID: 258

	Male						Female					
	Suicide			Suicide			Suicide			Suicide		
	Num per year	Rate /100,000	×100	Num per year	Rate /100,000	×100	Num per year	Rate /100,000	×100	Num per year	Rate /100,000	×100
	Total (>10 y/o)			45-54			Total (>10 y/o)			45-54		
1983-1987	47.4	36.9	119.1	11.8	59.8	118.3	23.2	15.6	102.6	6.2	24.9	144.7
1988-1992	31.4	24.0	97.6	5.6	30.2	87.9	19.2	12.0	90.9	3.0	14.4	98.5
1993-1997	38.6	26.5	109.1	10.8	44.3	121.0	17.8	10.5	93.4	4.2	15.6	120.7
1998-2002	57.0	38.1	108.3	14.8	61.8	110.4	26.0	13.1	110.9	4.2	16.5	109.9
2003-2007	54.4	36.9	102.0	9.4	48.1	86.0	24.4	13.1	108.7	3.2	14.6	106.6
2008-2012	51.8	35.2	104.4	8.6	48.0	98.7	19.4	12.2	92.3	2.2	13.2	88.4
	15-24			55-64			15-24			55-64		
1983-1987	3.2	16.3	116.8	9.6	56.1	132.0	2.2	9.1	133.7	3.8	20.2	102.4
1988-1992	1.4	8.2	82.8	5.2	30.5	86.0	2.0	7.3	137.8	3.4	17.3	96.9
1993-1997	3.0	12.4	110.0	9.0	46.9	116.5	1.2	5.3	102.3	2.6	13.9	87.7
1998-2002	2.2	13.0	82.5	12.6	66.3	104.4	0.4	4.6	65.7	4.4	20.0	106.1
2003-2007	4.8	22.1	126.7	13.4	60.5	104.8	0.8	6.5	72.0	5.4	20.1	120.2
2008-2012	3.4	20.9	98.7	11.8	50.6	104.2	1.2	8.4	86.9	5.0	18.1	121.3
	25-34			65-74			25-34			65-74		
1983-1987	4.0	23.2	90.9	4.4	45.8	104.5	1.8	9.9	92.0	3.6	28.3	90.6
1988-1992	3.2	19.0	99.6	4.0	36.1	102.5	1.0	7.1	82.4	2.2	17.4	66.7
1993-1997	3.4	19.5	100.0	2.4	21.5	67.3	2.2	10.2	122.2	2.6	16.1	82.9
1998-2002	5.8	28.5	105.4	8.0	49.2	108.3	1.8	10.4	89.5	5.2	25.2	120.0
2003-2007	8.4	38.3	123.5	6.0	38.8	90.5	2.2	12.1	93.3	5.8	25.6	137.5
2008-2012	7.4	38.4	118.0	6.0	37.4	94.7	2.6	14.8	103.1	2.8	15.7	88.3
	35-44			>74 y/o			35-44			>74 y/o		
1983-1987	8.4	35.5	111.6	5.8	99.4	126.9	2.4	10.8	91.7	3.2	41.1	71.2
1988-1992	5.4	23.6	99.4	6.6	87.9	136.3	2.4	10.0	103.2	5.2	43.8	91.5
1993-1997	4.8	24.0	97.9	5.2	62.6	120.2	1.4	7.6	88.8	3.6	25.7	74.5
1998-2002	8.2	42.0	119.2	5.2	54.4	103.1	2.4	11.5	111.4	7.4	39.3	126.4
2003-2007	6.4	35.8	89.4	5.8	47.5	105.5	2.8	13.7	108.4	4.2	19.6	87.5
2008-2012	7.4	36.4	98.0	7.2	47.8	115.3	1.8	11.1	82.2	3.8	15.9	82.9

Secondary Medical Zone ID: 259

	Male						Female					
	Suicide			Suicide			Suicide			Suicide		
	Num per year	Rate /100,000	×100	Num per year	Rate /100,000	×100	Num per year	Rate /100,000	×100	Num per year	Rate /100,000	×100
	Total (>10 y/o)			45–54			Total (>10 y/o)			45–54		
1983–1987	50.0	39.6	131.0	13.2	68.5	135.4	25.0	16.7	114.9	5.0	21.7	125.8
1988–1992	38.0	28.5	122.9	8.4	42.6	124.0	18.8	12.2	94.0	3.8	16.9	115.4
1993–1997	32.0	25.3	99.3	7.0	34.0	92.9	17.2	10.5	96.4	3.0	13.3	102.8
1998–2002	54.4	39.1	113.5	13.8	62.6	111.9	19.0	12.2	92.2	4.0	16.7	111.0
2003–2007	52.0	39.1	110.1	9.8	56.1	100.3	16.6	11.5	87.9	1.2	10.1	74.0
2008–2012	47.8	37.2	110.8	8.4	54.9	112.9	22.0	14.2	111.2	3.6	19.1	127.8
	15–24			55–64			15–24			55–64		
1983–1987	2.2	14.0	100.2	9.0	54.1	127.3	1.4	7.5	110.1	4.0	20.8	105.6
1988–1992	2.0	10.5	105.9	8.0	43.6	122.9	1.0	5.3	101.2	3.4	17.4	97.5
1993–1997	2.0	11.0	97.1	6.4	38.3	95.2	0.6	4.3	83.2	4.0	18.5	116.7
1998–2002	2.8	16.1	101.7	11.2	63.2	99.5	1.2	7.3	103.3	3.0	16.8	89.4
2003–2007	2.6	17.4	100.0	14.0	66.0	114.3	0.4	6.2	68.5	5.2	20.4	121.7
2008–2012	2.6	20.7	97.6	9.8	47.0	96.8	1.6	11.7	120.8	4.0	16.4	110.1
	25–34			65–74			25–34			65–74		
1983–1987	7.0	35.2	137.6	6.2	58.2	132.9	1.0	7.8	72.7	4.8	35.6	113.9
1988–1992	3.6	22.4	117.0	3.6	33.4	94.9	1.0	7.6	87.4	3.2	22.2	85.1
1993–1997	3.2	20.8	106.4	4.4	33.0	103.4	1.0	7.5	90.0	2.6	16.3	83.5
1998–2002	5.8	31.9	118.2	7.2	47.8	105.2	0.8	8.5	73.3	3.8	20.6	97.8
2003–2007	6.0	34.7	111.8	4.8	35.1	81.7	1.8	12.1	92.9	3.0	16.9	90.9
2008–2012	4.6	32.6	100.1	6.4	40.6	102.7	2.4	15.5	108.2	3.2	17.5	98.2
	35–44			>74 y/o			35–44			>74 y/o		
1983–1987	7.4	33.1	103.9	4.8	94.2	120.2	3.8	14.5	123.1	4.8	63.8	110.6
1988–1992	5.4	24.7	104.3	7.0	96.1	149.0	1.2	7.5	78.1	5.2	47.4	99.1
1993–1997	6.0	31.1	126.7	3.0	42.2	81.1	1.4	8.0	94.5	4.6	33.5	97.2
1998–2002	6.6	41.1	116.6	7.0	70.6	133.9	2.4	12.7	122.6	3.8	23.4	75.5
2003–2007	7.6	48.8	121.8	7.2	58.9	130.9	1.8	12.2	96.4	3.0	15.7	70.0
2008–2012	9.2	48.8	131.2	6.6	47.8	115.3	2.2	13.5	99.3	5.0	20.4	106.0

Secondary Medical Zone ID: 260

	Male						Female					
	Suicide			Suicide			Suicide			Suicide		
	Num per year	Rate /100,000	×100	Num per year	Rate /100,000	×100	Num per year	Rate /100,000	×100	Num per year	Rate /100,000	×100
	Total (>10 y/o)			45-54			Total (>10 y/o)			45-54		
1983-1987	56.2	40.5	133.3	15.0	67.6	133.7	22.6	14.5	94.5	4.0	16.9	97.8
1988-1992	33.8	25.1	102.8	7.4	35.9	104.5	19.2	11.5	86.9	3.2	14.0	96.0
1993-1997	39.6	26.9	112.5	9.8	41.9	114.5	16.4	9.9	84.7	4.2	15.2	117.3
1998-2002	56.4	38.6	111.1	13.4	59.8	106.8	20.0	11.6	88.9	4.6	17.4	115.5
2003-2007	52.8	39.5	108.2	12.2	65.5	117.1	19.6	12.5	93.4	3.0	14.5	105.6
2008-2012	50.2	37.4	112.9	9.0	56.4	116.1	19.6	13.1	96.9	2.4	14.6	98.0
	15-24			55-64			15-24			55-64		
1983-1987	2.8	15.0	107.7	9.4	50.9	119.7	1.8	7.8	113.7	3.8	18.9	95.7
1988-1992	1.4	8.4	84.8	8.0	39.2	110.5	1.0	5.0	94.9	3.4	16.1	89.9
1993-1997	2.8	12.3	108.7	10.0	48.1	119.5	1.4	6.1	116.5	3.6	15.7	98.5
1998-2002	2.6	14.9	94.4	15.6	76.7	120.7	0.4	5.0	71.1	4.6	19.5	103.5
2003-2007	1.8	14.8	84.7	13.0	60.5	104.9	1.6	10.2	113.1	4.8	18.4	109.8
2008-2012	3.0	22.5	106.2	12.8	56.3	115.8	1.4	10.7	110.8	2.8	13.2	88.7
	25-34			65-74			25-34			65-74		
1983-1987	7.2	34.3	134.1	5.8	54.6	124.8	2.2	10.6	98.8	4.2	30.7	98.2
1988-1992	3.0	19.3	101.1	4.4	36.8	104.5	1.8	9.3	107.5	1.6	13.7	52.7
1993-1997	3.6	22.3	114.2	5.4	35.4	111.0	2.4	10.9	130.7	1.0	9.1	46.7
1998-2002	6.4	34.5	127.9	8.8	50.2	110.4	1.4	9.9	85.0	2.8	15.6	74.5
2003-2007	4.8	30.9	99.8	8.2	47.0	109.4	2.0	12.5	96.1	3.2	16.1	86.6
2008-2012	4.6	33.1	101.7	8.2	45.8	115.9	3.0	17.3	120.8	4.0	18.3	103.1
	35-44			>74 y/o			35-44			>74 y/o		
1983-1987	12.4	47.5	149.2	3.4	68.9	88.0	3.8	13.5	114.0	2.6	35.1	60.8
1988-1992	6.2	26.5	112.0	3.2	51.9	80.4	2.6	10.2	105.8	5.6	46.4	96.9
1993-1997	4.2	23.1	94.3	3.8	49.0	94.0	0.6	5.6	65.3	3.2	23.0	67.0
1998-2002	5.8	36.4	103.4	3.4	39.4	74.8	1.6	9.8	94.6	4.6	25.8	83.2
2003-2007	8.6	53.4	133.4	4.2	36.9	81.8	2.0	12.3	97.7	3.0	14.9	66.2
2008-2012	7.0	41.0	110.2	5.6	38.7	93.3	2.2	13.2	97.6	3.8	15.4	80.2

Secondary Medical Zone ID: 261

	Male						Female					
	Suicide			Suicide			Suicide			Suicide		
	Num per year	Rate /100,000	×100	Num per year	Rate /100,000	×100	Num per year	Rate /100,000	×100	Num per year	Rate /100,000	×100
	Total (>10 y/o)			45–54			Total (>10 y/o)			45–54		
1983–1987	7.2	33.2	103.1	2.4	58.9	116.4	5.4	16.0	111.7	0.6	16.9	98.0
1988–1992	5.0	24.4	96.1	1.6	40.1	116.8	2.4	11.6	79.7	0.0	12.4	84.6
1993–1997	7.2	26.8	118.6	2.2	44.5	121.7	1.8	9.8	78.1	0.2	11.7	90.8
1998–2002	9.4	37.8	114.1	3.8	74.9	133.8	4.2	12.9	105.2	1.0	17.0	113.2
2003–2007	8.8	38.7	114.0	1.6	56.0	100.1	4.0	12.8	107.8	0.0	11.8	86.2
2008–2012	5.6	34.2	95.5	0.8	46.3	95.3	3.2	13.4	101.9	0.6	16.3	109.5
	15–24			55–64			15–24			55–64		
1983–1987	0.2	13.2	94.4	1.6	45.3	106.7	0.2	7.0	102.6	1.6	25.2	127.7
1988–1992	0.2	9.9	99.8	0.4	27.0	76.1	0.0	4.8	90.4	0.6	17.2	96.4
1993–1997	0.4	12.1	106.7	1.8	45.2	112.4	0.0	4.7	90.9	0.4	14.6	92.0
1998–2002	0.2	15.2	96.3	2.2	67.8	106.7	0.0	6.5	92.0	1.0	20.5	108.8
2003–2007	0.4	18.2	104.6	2.6	66.1	114.6	0.2	9.4	104.3	0.6	16.7	99.7
2008–2012	0.2	20.9	98.7	1.8	49.4	101.7	0.8	14.3	147.4	0.4	14.3	95.9
	25–34			65–74			25–34			65–74		
1983–1987	1.2	29.9	116.8	0.8	42.8	97.7	0.4	11.2	104.5	0.8	30.2	96.7
1988–1992	0.6	21.2	110.9	0.2	27.0	76.6	0.0	7.7	88.8	0.4	20.9	80.0
1993–1997	0.4	20.1	103.1	0.4	26.0	81.6	0.4	9.4	112.8	0.0	12.9	66.3
1998–2002	1.2	32.4	120.1	0.6	37.7	82.9	0.2	11.4	98.8	0.4	18.1	86.0
2003–2007	0.2	28.0	90.2	1.2	43.0	100.1	0.2	12.8	98.3	1.6	24.7	132.8
2008–2012	0.4	31.8	97.6	1.2	41.4	104.7	0.0	13.2	91.8	0.6	17.9	100.5
	35–44			>74 y/o			35–44			>74 y/o		
1983–1987	0.6	27.7	87.1	0.4	61.8	78.9	0.6	12.5	106.2	1.2	59.3	102.7
1988–1992	1.2	26.8	113.3	0.8	62.2	96.4	0.4	9.9	102.1	1.0	42.7	89.2
1993–1997	1.0	27.4	111.9	1.0	56.6	108.7	0.0	7.3	85.4	0.8	29.5	85.8
1998–2002	0.4	31.2	88.6	1.0	53.2	100.8	0.4	11.2	107.9	1.2	32.0	103.0
2003–2007	1.6	52.8	131.7	1.2	47.6	105.7	0.4	13.3	105.7	1.0	23.0	102.5
2008–2012	0.8	38.4	103.1	0.4	32.4	78.0	0.0	12.4	91.4	0.8	18.2	94.6

Secondary Medical Zone ID: 262

	Male						Female					
	Suicide			Suicide			Suicide			Suicide		
	Num per year	Rate /100,000	×100	Num per year	Rate /100,000	×100	Num per year	Rate /100,000	×100	Num per year	Rate /100,000	×100
	Total (>10 y/o)			45–54			Total (>10 y/o)			45–54		
1983–1987	16.6	38.1	137.9	4.4	67.3	133.0	8.0	15.7	109.0	1.4	19.0	110.1
1988–1992	13.6	28.9	143.5	2.8	44.7	130.0	7.8	13.5	115.2	0.4	13.0	88.8
1993–1997	10.0	26.1	113.0	2.4	40.7	111.3	6.4	11.3	113.1	1.4	15.7	121.8
1998–2002	13.4	37.0	110.6	3.8	64.1	114.6	7.0	13.0	111.8	0.8	15.1	100.2
2003–2007	13.0	38.2	113.9	1.8	48.8	87.3	5.8	12.8	107.7	0.2	12.0	87.5
2008–2012	12.4	36.8	118.4	2.6	58.7	120.7	3.4	12.8	90.9	0.2	13.4	89.6
	15–24			55–64			15–24			55–64		
1983–1987	0.2	12.0	85.9	2.6	46.7	109.9	0.0	5.7	84.2	1.6	22.0	111.6
1988–1992	0.4	10.3	103.6	4.0	53.5	150.6	0.4	6.0	114.4	1.6	20.3	113.4
1993–1997	0.4	11.5	102.1	2.4	43.5	108.1	0.0	4.5	85.5	1.6	19.5	122.9
1998–2002	0.6	16.7	105.9	3.4	69.2	109.0	0.4	7.8	111.4	1.4	20.5	108.9
2003–2007	0.2	16.4	94.1	3.0	60.4	104.6	0.4	10.1	112.7	0.8	16.3	97.4
2008–2012	0.0	18.8	88.5	3.0	52.4	107.9	0.2	9.9	101.8	0.2	12.7	84.8
	25–34			65–74			25–34			65–74		
1983–1987	2.0	32.1	125.7	2.8	62.9	143.7	0.8	12.3	114.3	2.2	39.2	125.5
1988–1992	0.0	13.6	71.0	1.8	41.6	118.1	0.2	8.1	93.9	3.2	44.5	170.5
1993–1997	1.0	23.1	118.1	2.4	43.1	135.2	0.2	8.0	96.6	1.8	25.0	128.4
1998–2002	0.6	25.9	96.1	2.8	51.8	113.8	0.2	10.9	94.2	1.0	19.5	92.7
2003–2007	0.8	30.5	98.3	2.2	46.0	107.2	0.6	14.1	108.6	1.0	18.4	99.0
2008–2012	1.0	34.4	105.5	1.6	39.9	101.1	0.2	13.7	95.6	0.6	16.3	91.6
	35–44			>74 y/o			35–44			>74 y/o		
1983–1987	1.8	33.0	103.8	2.8	113.6	145.1	0.8	12.4	104.7	1.2	44.0	76.3
1988–1992	2.2	30.7	129.4	2.4	87.0	134.9	0.2	8.5	87.9	1.8	45.3	94.6
1993–1997	0.6	20.5	83.6	0.8	42.5	81.6	0.0	6.8	79.8	1.4	31.2	90.6
1998–2002	0.6	29.4	83.4	1.6	54.5	103.3	0.2	9.5	92.2	3.0	45.7	147.2
2003–2007	2.2	54.1	135.1	2.8	61.5	136.6	0.2	11.8	93.1	2.6	33.2	148.2
2008–2012	1.6	41.2	110.8	2.6	51.5	124.0	1.2	17.0	125.4	0.8	14.9	77.7

Secondary Medical Zone ID: 263

	Male						Female					
	Suicide			Suicide			Suicide			Suicide		
	Num per year	Rate /100,000	×100	Num per year	Rate /100,000	×100	Num per year	Rate /100,000	×100	Num per year	Rate /100,000	×100
	Total (>10 y/o)			45–54			Total (>10 y/o)			45–54		
1983–1987	68.4	32.6	99.2	16.6	50.0	98.9	37.6	14.9	96.7	7.8	20.4	118.3
1988–1992	46.4	21.4	82.6	11.8	34.0	99.1	33.0	11.8	89.3	3.8	11.9	81.6
1993–1997	50.8	21.8	83.2	11.4	29.3	80.2	27.8	9.6	83.8	5.6	13.4	103.6
1998–2002	73.4	30.0	80.6	18.0	43.8	78.2	28.0	10.4	73.2	5.0	12.7	84.3
2003–2007	74.0	30.1	79.6	16.4	45.4	81.2	25.2	9.8	70.1	4.0	11.5	83.7
2008–2012	69.8	30.3	82.7	14.0	45.0	92.7	29.4	11.6	81.9	3.4	11.6	77.6
	15–24			55–64			15–24			55–64		
1983–1987	5.4	16.9	121.0	7.0	28.6	67.3	4.0	9.8	144.0	6.2	19.1	96.6
1988–1992	2.0	7.5	75.5	7.8	26.1	73.4	1.8	4.9	93.4	5.2	15.3	85.5
1993–1997	4.0	11.4	100.9	8.8	29.6	73.6	2.2	5.6	106.4	5.6	15.7	98.7
1998–2002	4.4	14.2	89.6	14.2	45.5	71.7	0.8	4.2	59.4	7.0	19.3	102.3
2003–2007	3.6	13.9	79.8	20.0	51.4	89.1	1.0	5.3	58.4	4.8	13.4	80.0
2008–2012	4.4	18.9	89.3	13.8	35.7	73.4	1.8	8.2	84.7	4.0	11.1	74.6
	25–34			65–74			25–34			65–74		
1983–1987	13.2	34.7	135.7	4.8	33.7	76.9	3.6	9.9	91.7	6.4	29.6	94.7
1988–1992	6.0	19.0	99.4	5.0	28.4	80.7	3.4	9.5	109.2	6.6	25.5	97.6
1993–1997	5.4	17.8	91.2	6.2	26.8	84.0	1.8	6.4	76.8	4.6	15.6	79.9
1998–2002	5.6	19.3	71.5	11.6	42.4	93.2	4.0	11.3	97.2	4.6	15.0	71.3
2003–2007	7.2	23.4	75.4	8.8	33.1	77.0	2.4	8.9	68.2	4.0	13.5	72.4
2008–2012	10.0	33.4	102.6	8.8	32.3	81.8	3.8	13.3	92.8	6.4	18.7	104.9
	35–44			>74 y/o			35–44			>74 y/o		
1983–1987	12.6	30.6	96.1	8.2	89.2	113.8	5.0	12.0	101.4	4.6	35.6	61.7
1988–1992	9.2	21.7	91.6	4.4	43.2	66.9	5.0	10.9	113.1	7.2	37.2	77.7
1993–1997	8.6	23.1	94.1	6.0	46.9	90.1	3.6	9.0	106.1	4.4	19.3	55.9
1998–2002	11.2	33.9	96.3	8.2	51.6	98.0	2.4	8.0	77.0	4.0	14.9	48.1
2003–2007	10.6	33.9	84.7	7.2	36.2	80.5	4.0	12.0	95.0	4.8	13.7	61.2
2008–2012	10.0	31.3	84.2	8.6	35.0	84.4	5.2	14.1	104.3	4.6	11.6	60.3

Secondary Medical Zone ID: 264

	Male						Female					
	Suicide			Suicide			Suicide			Suicide		
	Num per year	Rate /100,000	×100	Num per year	Rate /100,000	×100	Num per year	Rate /100,000	×100	Num per year	Rate /100,000	×100
	Total (>10 y/o)			45-54			Total (>10 y/o)			45-54		
1983-1987	20.0	29.6	81.9	3.4	33.6	66.5	14.2	15.0	97.1	2.2	17.0	98.7
1988-1992	15.4	21.6	80.8	3.0	28.6	83.3	12.0	12.1	91.7	1.6	14.0	95.4
1993-1997	15.6	22.4	79.5	2.8	27.9	76.1	9.8	10.4	88.3	2.8	16.4	126.7
1998-2002	21.0	30.7	74.9	6.2	50.0	89.4	11.4	11.9	90.3	2.8	17.7	117.2
2003-2007	21.0	31.3	75.7	5.2	49.4	88.3	9.2	11.9	84.4	1.0	11.9	87.2
2008-2012	22.4	32.5	89.9	3.6	43.6	89.7	8.6	12.5	85.9	1.6	15.7	105.4
	15-24			55-64			15-24			55-64		
1983-1987	2.2	18.8	134.6	3.0	33.0	77.7	0.2	5.2	76.9	1.6	16.0	81.2
1988-1992	0.8	9.4	94.8	2.2	24.0	67.5	0.4	4.9	92.2	1.4	14.3	80.1
1993-1997	1.4	12.4	109.6	2.6	29.0	72.0	0.2	4.3	81.6	0.4	9.4	59.4
1998-2002	1.4	15.9	100.6	3.8	43.2	68.0	0.0	5.1	71.9	2.0	17.8	94.6
2003-2007	0.8	15.2	87.0	5.2	46.8	81.1	0.4	7.9	87.6	2.6	18.1	108.1
2008-2012	1.0	19.4	91.3	4.4	40.3	82.9	0.4	8.8	91.0	1.8	14.5	97.3
	25-34			65-74			25-34			65-74		
1983-1987	2.2	22.3	87.2	2.2	38.8	88.7	1.4	11.4	106.0	2.4	28.0	89.5
1988-1992	1.4	17.1	89.3	2.6	35.3	100.1	1.0	9.2	106.7	1.8	20.4	78.2
1993-1997	1.4	18.2	93.3	2.6	29.4	92.0	1.4	10.6	127.2	1.4	14.8	75.9
1998-2002	1.2	21.1	78.2	3.6	38.5	84.7	0.4	9.3	80.5	3.4	24.7	117.7
2003-2007	3.6	35.3	114.0	2.4	30.3	70.7	1.6	14.8	113.9	1.6	16.0	85.7
2008-2012	3.0	34.9	107.2	3.4	36.6	92.5	1.0	14.1	98.8	0.8	13.3	74.6
	35-44			>74 y/o			35-44			>74 y/o		
1983-1987	5.4	37.2	117.0	1.6	51.1	65.3	2.4	14.3	121.1	4.0	63.2	109.5
1988-1992	1.4	15.7	66.3	4.0	78.1	121.0	2.2	12.3	127.3	3.6	45.0	94.0
1993-1997	3.4	27.9	114.0	1.4	34.4	66.1	1.4	10.0	117.9	2.2	24.8	72.0
1998-2002	3.2	34.3	97.3	1.6	34.3	65.0	1.2	11.0	106.8	1.6	17.3	55.6
2003-2007	1.6	26.6	66.3	2.2	32.9	72.9	0.8	11.4	90.4	1.2	11.6	51.7
2008-2012	3.2	36.9	99.3	3.8	40.4	97.3	1.6	14.9	110.0	1.4	11.9	61.8

Secondary Medical Zone ID: 265

	Male						Female					
	Suicide			Suicide			Suicide			Suicide		
	Num per year	Rate /100,000	×100	Num per year	Rate /100,000	×100	Num per year	Rate /100,000	×100	Num per year	Rate /100,000	×100
	Total (>10 y/o)			45–54			Total (>10 y/o)			45–54		
1983–1987	27.6	42.7	149.7	4.6	54.3	107.4	14.0	17.4	124.9	1.4	16.5	95.8
1988–1992	22.2	31.4	153.5	5.0	54.8	159.5	15.4	15.7	146.4	2.0	18.2	124.2
1993–1997	19.4	29.8	135.5	3.6	42.8	116.9	12.2	12.5	137.7	0.0	9.5	73.8
1998–2002	18.6	34.9	102.0	2.6	43.3	77.3	10.0	13.1	110.6	2.0	18.5	123.1
2003–2007	22.2	40.3	123.9	4.8	63.1	112.9	8.2	12.9	105.9	2.0	18.0	131.4
2008–2012	18.4	37.3	116.8	2.2	44.9	92.4	8.4	13.2	111.8	0.8	14.8	99.0
	15–24			55–64			15–24			55–64		
1983–1987	1.6	19.2	137.7	5.4	54.5	128.4	0.6	7.6	112.0	3.0	24.2	122.8
1988–1992	0.8	11.0	111.2	5.6	51.3	144.5	0.4	5.8	109.3	3.2	23.6	132.0
1993–1997	0.6	11.4	100.9	3.6	43.6	108.3	0.2	5.0	96.0	2.4	20.6	129.8
1998–2002	1.0	16.9	107.1	2.6	51.9	81.7	0.0	5.8	82.7	1.6	19.9	105.9
2003–2007	0.8	17.9	102.5	3.8	57.1	98.9	0.0	7.1	79.4	0.8	15.5	92.8
2008–2012	1.2	24.5	115.6	5.2	57.1	117.6	0.2	9.2	94.9	1.4	16.1	108.1
	25–34			65–74			25–34			65–74		
1983–1987	4.6	42.2	165.0	1.2	33.3	76.1	1.2	12.7	118.0	2.2	32.0	102.3
1988–1992	1.8	24.1	125.8	3.2	45.6	129.4	1.2	11.5	133.0	2.8	31.7	121.5
1993–1997	1.2	21.4	109.8	3.8	44.1	138.3	0.6	9.1	109.1	4.0	32.8	168.5
1998–2002	1.4	28.4	105.2	5.0	56.6	124.4	0.6	11.9	102.8	3.2	27.4	130.5
2003–2007	2.8	42.0	135.4	4.4	55.7	129.8	0.8	14.3	109.9	1.6	19.0	101.9
2008–2012	3.2	44.7	137.3	0.8	30.0	75.9	0.8	15.8	109.9	2.4	23.5	132.1
	35–44			>74 y/o			35–44			>74 y/o		
1983–1987	5.0	50.9	160.0	5.2	139.1	177.6	2.4	18.1	153.5	3.2	66.9	116.0
1988–1992	3.4	32.7	137.8	2.4	69.2	107.3	1.2	11.4	117.7	4.6	70.1	146.5
1993–1997	4.2	40.7	166.2	2.4	58.7	112.8	0.8	9.4	110.4	4.2	52.9	153.6
1998–2002	2.0	35.0	99.3	4.0	72.2	137.0	0.2	8.6	83.1	2.4	28.5	91.7
2003–2007	2.0	42.0	104.9	3.6	55.1	122.3	0.4	11.8	93.3	2.6	24.0	106.8
2008–2012	1.8	39.0	105.0	4.0	52.6	126.7	0.0	11.3	83.2	2.8	21.9	113.7

Secondary Medical Zone ID: 266

	Male						Female					
	Suicide			Suicide			Suicide			Suicide		
	Num per year	Rate /100,000	×100	Num per year	Rate /100,000	×100	Num per year	Rate /100,000	×100	Num per year	Rate /100,000	×100
	Total (>10 y/o)			45–54			Total (>10 y/o)			45–54		
1983–1987	11.8	30.5	89.0	2.4	42.0	83.1	8.4	15.4	103.3	1.6	18.9	109.7
1988–1992	10.2	24.2	94.1	1.8	30.7	89.4	9.0	13.4	113.7	1.4	16.0	109.3
1993–1997	8.4	22.9	79.6	2.4	34.9	95.4	6.8	10.9	103.1	1.8	16.0	123.9
1998–2002	16.4	35.4	97.8	4.2	57.2	102.2	6.0	11.9	88.8	1.0	14.7	97.9
2003–2007	17.2	37.4	104.5	3.2	54.8	98.1	5.6	11.9	90.9	1.2	15.2	111.2
2008–2012	15.0	35.4	103.1	2.0	45.3	93.3	5.2	12.9	92.1	0.8	15.1	101.0
	15–24			55–64			15–24			55–64		
1983–1987	0.4	11.6	83.0	2.0	38.4	90.5	0.4	6.7	98.5	0.6	15.7	79.7
1988–1992	1.4	12.7	128.8	1.2	26.7	75.1	0.2	4.7	89.8	1.4	18.4	103.1
1993–1997	0.0	8.4	74.1	1.6	33.0	82.0	0.2	4.7	91.0	1.0	15.3	96.0
1998–2002	1.4	17.8	112.7	4.2	64.3	101.3	0.0	5.7	80.6	0.8	16.6	88.4
2003–2007	0.8	17.4	99.8	5.4	67.9	117.7	0.2	8.1	89.5	1.2	16.5	98.6
2008–2012	2.2	30.2	142.2	4.8	56.6	116.5	0.2	9.0	92.5	0.4	12.5	84.0
	25–34			65–74			25–34			65–74		
1983–1987	0.8	20.8	81.3	1.2	39.8	90.8	0.2	8.7	80.8	1.2	28.2	90.3
1988–1992	1.6	23.9	125.3	1.0	30.7	87.2	0.6	9.3	107.1	1.8	29.4	112.7
1993–1997	0.8	18.7	95.8	1.0	25.8	81.0	0.4	8.2	98.8	0.8	16.3	83.4
1998–2002	2.0	30.7	113.6	2.4	43.7	96.0	0.4	10.8	92.8	1.2	19.6	93.2
2003–2007	2.4	37.1	119.7	2.0	38.7	90.2	0.0	10.1	77.8	0.8	16.2	86.8
2008–2012	1.0	30.9	94.8	2.2	38.9	98.6	1.2	17.4	121.4	0.6	15.2	85.3
	35–44			>74 y/o			35–44			>74 y/o		
1983–1987	3.2	37.2	117.0	1.6	75.6	96.5	1.8	15.2	128.6	2.4	67.4	116.9
1988–1992	2.0	25.5	107.7	1.2	53.9	83.6	0.6	9.3	96.7	3.0	60.9	127.2
1993–1997	1.8	27.0	110.1	0.8	38.3	73.6	0.4	8.0	94.3	2.0	36.5	106.0
1998–2002	0.8	26.7	75.8	1.2	42.0	79.7	0.6	10.6	102.3	2.0	30.8	99.2
2003–2007	1.8	39.1	97.6	1.6	39.2	87.1	0.4	11.6	92.1	1.8	22.9	102.0
2008–2012	1.2	33.2	89.4	1.4	33.2	79.9	1.0	14.8	109.0	1.0	14.8	76.8

Secondary Medical Zone ID: 267

	Male						Female					
	Suicide			Suicide			Suicide			Suicide		
	Num per year	Rate /100,000	×100	Num per year	Rate /100,000	×100	Num per year	Rate /100,000	×100	Num per year	Rate /100,000	×100
	Total (>10 y/o)			45–54			Total (>10 y/o)			45–54		
1983–1987	7.2	34.0	113.1	1.8	57.0	112.7	4.8	16.0	112.8	1.2	21.3	123.7
1988–1992	6.2	26.7	119.1	0.6	31.9	92.9	3.0	12.4	94.6	0.6	15.6	106.8
1993–1997	4.2	25.0	95.1	0.8	34.8	95.2	2.0	10.3	87.3	0.0	11.3	87.4
1998–2002	5.0	34.7	88.3	1.6	56.1	100.3	2.0	11.9	87.2	0.4	15.0	99.5
2003–2007	4.4	34.2	83.6	0.6	44.9	80.2	2.0	12.3	92.5	0.2	13.1	95.6
2008–2012	3.4	32.6	84.9	0.6	45.3	93.2	1.6	12.5	92.0	0.2	14.4	96.8
	15–24			55–64			15–24			55–64		
1983–1987	0.4	15.1	108.2	1.0	40.7	95.9	0.2	7.0	103.2	0.6	19.3	97.9
1988–1992	0.2	10.0	100.6	0.8	33.3	93.9	0.2	5.7	107.2	0.4	16.7	93.8
1993–1997	0.4	12.2	107.9	0.8	38.3	95.2	0.0	4.8	92.0	0.4	15.5	97.7
1998–2002	0.2	15.6	98.8	0.6	52.6	82.9	0.0	6.6	93.7	0.4	18.4	97.7
2003–2007	0.2	17.4	99.9	0.4	44.0	76.2	0.0	8.4	92.9	0.4	16.5	98.3
2008–2012	0.0	19.8	93.3	0.8	43.9	90.3	0.0	9.1	93.7	0.2	14.0	93.8
	25–34			65–74			25–34			65–74		
1983–1987	1.0	29.2	114.1	1.4	54.1	123.6	0.8	13.8	128.3	0.2	23.5	75.3
1988–1992	0.8	23.3	121.8	0.8	36.5	103.7	0.0	7.8	89.7	0.6	24.3	93.0
1993–1997	0.2	18.4	94.3	0.4	27.1	85.1	0.2	8.5	102.7	0.8	21.4	109.6
1998–2002	0.4	26.7	99.0	1.0	44.2	97.3	0.0	10.7	92.3	0.6	20.6	98.1
2003–2007	0.6	32.2	103.9	0.8	41.1	95.9	0.4	14.1	108.7	0.0	15.1	81.1
2008–2012	0.4	32.4	99.3	0.6	37.5	95.0	0.0	13.4	93.7	0.2	16.5	92.6
	35–44			>74 y/o			35–44			>74 y/o		
1983–1987	1.4	36.5	114.8	0.0	49.1	62.6	0.8	14.0	118.3	1.0	57.2	99.1
1988–1992	2.6	40.2	169.7	0.4	52.3	81.0	0.2	9.4	97.0	1.0	45.7	95.5
1993–1997	1.2	30.8	125.5	0.4	43.0	82.6	0.2	8.5	99.7	0.4	23.6	68.6
1998–2002	0.8	36.6	104.0	0.4	42.4	80.4	0.0	9.3	89.5	0.6	24.6	79.2
2003–2007	0.8	42.3	105.5	1.0	46.0	102.2	0.4	13.6	107.3	0.6	19.3	86.1
2008–2012	0.0	32.9	88.3	1.0	42.0	101.2	0.2	13.5	99.6	0.8	19.3	100.6

Secondary Medical Zone ID: 268

	Male						Female					
	Suicide			Suicide			Suicide			Suicide		
	Num per year	Rate /100,000	×100	Num per year	Rate /100,000	×100	Num per year	Rate /100,000	×100	Num per year	Rate /100,000	×100
	Total (>10 y/o)			45–54			Total (>10 y/o)			45–54		
1983–1987	53.4	31.8	95.8	14.8	53.6	106.0	28.0	14.4	92.6	5.0	17.5	101.4
1988–1992	45.4	24.4	98.7	11.4	38.0	110.6	25.6	11.9	90.3	4.8	15.5	106.2
1993–1997	47.2	23.9	94.1	14.2	39.7	108.6	24.0	10.4	93.0	3.4	10.9	84.8
1998–2002	60.6	31.0	81.4	17.2	50.0	89.3	29.0	12.2	93.5	4.8	14.3	94.7
2003–2007	70.4	34.9	92.0	18.2	60.6	108.4	29.0	12.2	96.0	3.8	13.1	95.6
2008–2012	67.2	34.1	96.8	10.2	41.4	85.1	28.2	13.1	95.4	5.8	19.2	128.4
	15–24			55–64			15–24			55–64		
1983–1987	4.6	17.0	122.0	7.4	36.8	86.7	1.8	6.8	99.7	3.6	16.2	82.1
1988–1992	3.2	10.6	106.9	7.8	32.4	91.4	2.4	6.9	131.1	3.6	14.8	82.7
1993–1997	3.4	11.4	100.7	8.0	33.4	83.1	2.8	7.6	146.2	2.6	11.4	71.9
1998–2002	5.0	17.9	113.4	12.0	47.4	74.5	1.8	7.0	99.6	5.8	19.6	104.4
2003–2007	3.4	16.1	92.3	14.6	47.1	81.7	1.4	7.4	82.8	7.8	21.0	125.1
2008–2012	4.4	22.3	105.2	13.2	42.0	86.5	2.6	12.2	125.8	4.2	13.3	88.9
	25–34			65–74			25–34			65–74		
1983–1987	7.8	26.6	104.0	3.6	33.9	77.5	4.6	13.6	126.7	2.8	20.5	65.7
1988–1992	5.8	20.9	109.3	5.8	39.3	111.6	2.4	8.5	98.3	3.8	21.1	80.8
1993–1997	6.2	21.4	109.7	4.6	26.9	84.4	3.0	9.5	114.3	3.0	14.6	74.8
1998–2002	7.0	23.6	87.6	6.2	33.4	73.4	4.0	12.1	104.6	4.4	18.4	87.4
2003–2007	9.8	31.7	102.4	6.6	32.8	76.3	4.0	12.8	98.4	4.8	18.6	99.7
2008–2012	8.6	33.9	104.1	8.8	38.2	96.8	3.2	13.1	91.3	4.4	17.0	95.6
	35–44			>74 y/o			35–44			>74 y/o		
1983–1987	9.6	27.4	86.1	5.2	75.2	96.0	5.6	14.1	119.7	4.2	41.8	72.4
1988–1992	7.6	21.6	91.3	3.6	46.4	71.9	3.2	9.2	94.9	5.4	38.5	80.5
1993–1997	6.8	22.6	92.1	4.0	42.4	81.4	3.0	9.2	108.6	6.0	33.8	98.3
1998–2002	9.0	32.6	92.4	4.2	37.9	72.0	3.0	10.4	101.0	5.0	23.7	76.3
2003–2007	11.4	40.0	99.8	6.0	38.9	86.4	3.0	11.2	88.9	4.2	16.2	72.2
2008–2012	14.4	43.7	117.4	7.2	38.3	92.4	3.8	12.6	92.7	4.2	14.2	73.9

Secondary Medical Zone ID: 269

	Male						Female					
	Suicide			Suicide			Suicide			Suicide		
	Num per year	Rate /100,000	×100	Num per year	Rate /100,000	×100	Num per year	Rate /100,000	×100	Num per year	Rate /100,000	×100
	Total (>10 y/o)			45–54			Total (>10 y/o)			45–54		
1983–1987	47.8	37.1	116.8	10.8	56.1	110.9	19.8	13.9	87.4	1.6	11.8	68.4
1988–1992	37.2	26.6	111.7	6.6	34.0	99.1	20.4	12.5	94.1	3.0	14.8	101.0
1993–1997	36.8	25.8	104.0	11.2	44.9	122.8	18.0	10.2	94.1	3.0	12.9	100.1
1998–2002	44.4	33.1	87.2	13.4	57.0	101.8	17.8	11.4	82.9	3.0	13.8	91.7
2003–2007	44.4	32.3	86.8	7.6	42.2	75.4	17.0	11.6	84.9	2.2	12.3	90.0
2008–2012	46.0	34.0	98.0	7.6	47.1	96.9	16.0	11.6	84.7	2.4	14.5	97.2
	15–24			55–64			15–24			55–64		
1983–1987	3.8	20.3	145.2	6.8	41.3	97.2	1.4	7.4	108.7	3.8	19.5	99.0
1988–1992	2.2	10.7	108.1	7.6	39.5	111.3	2.2	8.1	153.7	2.4	14.1	79.0
1993–1997	2.2	11.2	99.4	7.2	39.7	98.5	0.8	4.6	89.0	2.8	14.7	92.2
1998–2002	3.0	16.7	105.8	10.6	57.8	91.0	1.0	6.5	93.0	2.8	16.2	85.9
2003–2007	2.0	15.5	88.9	10.6	49.6	85.9	0.8	7.4	82.7	2.6	13.8	82.4
2008–2012	2.8	21.4	100.8	11.8	50.5	103.9	0.8	8.3	85.6	2.0	11.5	77.2
	25–34			65–74			25–34			65–74		
1983–1987	6.4	32.0	125.0	3.6	37.8	86.3	2.4	11.9	110.2	3.0	23.8	76.2
1988–1992	4.2	23.8	124.3	5.2	41.8	118.5	1.6	9.1	105.0	3.4	22.2	85.1
1993–1997	2.2	15.8	80.9	4.0	28.8	90.3	1.2	7.8	93.5	2.0	13.3	68.1
1998–2002	5.8	29.5	109.3	4.0	30.6	67.4	1.8	10.6	91.7	2.2	14.4	68.6
2003–2007	6.4	32.6	105.2	7.4	45.0	105.0	2.6	13.4	103.2	3.4	18.1	97.2
2008–2012	6.2	35.9	110.1	6.4	39.1	99.0	1.6	12.3	86.1	3.6	18.7	104.8
	35–44			>74 y/o			35–44			>74 y/o		
1983–1987	10.0	39.8	125.2	6.2	93.5	119.4	1.8	9.5	80.8	5.6	59.2	102.6
1988–1992	5.6	23.9	100.9	5.8	72.1	111.7	3.0	11.3	117.5	4.8	38.3	79.9
1993–1997	6.4	30.4	124.2	3.4	42.2	80.9	1.8	8.8	103.5	6.4	39.9	115.9
1998–2002	4.4	28.6	81.1	3.2	36.2	68.7	1.8	10.3	99.8	5.0	27.1	87.3
2003–2007	4.8	31.3	78.2	5.4	42.3	93.9	2.0	12.2	96.9	3.4	16.2	72.1
2008–2012	6.6	35.8	96.3	4.6	33.0	79.6	2.0	12.2	90.0	3.6	15.2	78.9

Secondary Medical Zone ID: 270

	Male						Female					
	Suicide			Suicide			Suicide			Suicide		
	Num per year	Rate /100,000	×100	Num per year	Rate /100,000	×100	Num per year	Rate /100,000	×100	Num per year	Rate /100,000	×100
	Total (>10 y/o)			45–54			Total (>10 y/o)			45–54		
1983–1987	20.0	32.6	97.7	4.8	50.9	100.6	10.6	14.2	90.0	1.4	15.8	91.5
1988–1992	14.2	23.2	88.7	2.8	32.4	94.5	10.0	12.2	91.9	0.8	12.5	85.8
1993–1997	14.4	23.9	87.7	4.0	37.6	102.8	9.0	10.4	95.3	1.0	11.8	91.4
1998–2002	21.6	35.5	90.8	5.6	54.2	96.8	7.4	11.0	78.7	1.8	15.8	105.1
2003–2007	20.0	34.4	86.0	3.4	43.6	78.0	5.4	10.6	71.7	0.8	12.1	88.0
2008–2012	16.0	31.7	80.9	4.2	51.7	106.3	7.8	12.5	90.8	2.0	18.3	122.6
	15–24			55–64			15–24			55–64		
1983–1987	1.2	14.8	105.9	2.4	33.5	78.7	0.8	7.7	112.3	2.6	22.0	111.7
1988–1992	1.4	11.9	120.3	3.6	36.6	103.1	0.4	5.1	96.8	1.6	16.5	92.5
1993–1997	0.2	8.7	76.7	3.2	37.6	93.4	0.0	3.8	72.2	1.4	15.0	94.3
1998–2002	1.0	15.1	95.4	6.0	66.7	105.0	0.2	6.0	85.3	0.8	15.1	80.1
2003–2007	1.4	18.9	108.2	5.0	52.7	91.2	0.4	8.3	92.6	1.6	16.3	97.5
2008–2012	0.8	19.2	90.6	2.6	36.2	74.5	0.2	8.1	83.9	2.8	18.7	125.7
	25–34			65–74			25–34			65–74		
1983–1987	2.8	28.9	113.1	1.4	32.7	74.7	0.8	10.1	93.7	1.6	24.7	79.2
1988–1992	1.6	19.9	104.3	0.6	20.7	58.8	1.2	10.6	122.5	2.2	25.3	96.9
1993–1997	1.6	20.6	105.5	1.6	25.2	79.0	0.6	8.3	99.4	2.4	22.2	114.1
1998–2002	3.0	32.0	118.4	1.4	29.2	64.1	1.2	12.8	110.5	1.2	16.2	77.0
2003–2007	2.6	32.4	104.5	2.0	32.4	75.5	0.0	9.1	69.8	1.2	15.9	85.3
2008–2012	1.4	28.5	87.4	1.6	30.4	76.9	0.8	13.8	96.1	0.4	12.9	72.7
	35–44			>74 y/o			35–44			>74 y/o		
1983–1987	3.8	34.0	107.0	3.6	91.6	117.0	1.4	12.4	104.9	1.8	38.6	67.0
1988–1992	2.4	23.1	97.4	1.8	48.7	75.5	1.2	10.3	106.2	2.4	36.9	77.1
1993–1997	2.8	28.1	114.6	1.0	30.8	59.2	0.8	8.6	101.0	2.8	33.4	97.1
1998–2002	3.8	42.4	120.4	0.6	25.4	48.1	0.0	7.0	67.7	2.2	24.1	77.5
2003–2007	3.4	43.9	109.5	2.2	35.8	79.6	0.2	9.8	77.4	1.2	13.2	58.9
2008–2012	2.8	36.9	99.1	2.6	35.7	86.0	0.4	11.4	83.9	1.2	12.5	65.1

Secondary Medical Zone ID: 271

	Male						Female					
	Suicide			Suicide			Suicide			Suicide		
	Num per year	Rate /100,000	×100	Num per year	Rate /100,000	×100	Num per year	Rate /100,000	×100	Num per year	Rate /100,000	×100
	Total (>10 y/o)			45–54			Total (>10 y/o)			45–54		
1983–1987	15.6	35.0	115.2	3.6	54.4	107.6	6.2	14.5	90.7	1.2	17.6	102.1
1988–1992	14.0	27.9	124.5	3.0	40.8	118.6	6.4	12.4	96.9	0.8	14.1	96.2
1993–1997	13.6	27.2	116.1	4.6	48.8	133.5	5.4	10.5	94.6	0.4	11.0	85.0
1998–2002	20.2	38.2	118.2	5.8	67.8	121.2	5.4	11.8	87.6	0.6	13.3	88.0
2003–2007	18.6	37.5	111.4	2.2	42.4	75.8	6.4	12.7	99.6	1.4	15.8	115.4
2008–2012	15.4	34.8	102.8	3.0	50.8	104.6	6.2	13.1	99.9	1.0	15.7	105.3
	15–24			55–64			15–24			55–64		
1983–1987	1.0	15.6	112.2	1.6	36.7	86.5	0.2	6.1	89.8	1.4	20.6	104.6
1988–1992	0.6	10.0	100.7	3.0	43.0	121.1	0.2	4.9	93.3	1.6	20.0	112.2
1993–1997	0.6	11.2	99.1	2.4	40.4	100.3	0.4	5.7	109.0	1.0	15.8	99.6
1998–2002	1.2	18.0	114.0	4.2	67.5	106.2	0.4	7.3	104.5	0.6	16.3	86.4
2003–2007	0.6	16.6	95.3	3.8	57.1	98.8	0.2	8.2	91.1	0.6	14.7	87.5
2008–2012	0.6	20.1	94.7	5.8	62.3	128.2	0.2	8.9	92.0	0.4	12.5	84.1
	25–34			65–74			25–34			65–74		
1983–1987	3.2	35.7	139.5	2.4	58.6	133.8	0.4	9.3	86.7	0.8	26.1	83.6
1988–1992	2.0	25.3	132.3	1.2	34.3	97.4	0.4	8.2	94.8	0.8	21.3	81.8
1993–1997	2.4	27.6	141.1	1.0	27.4	86.0	0.2	7.3	87.4	1.2	20.1	102.9
1998–2002	1.8	27.9	103.3	3.2	53.3	117.2	0.8	12.1	104.7	1.4	21.8	103.7
2003–2007	3.0	38.5	124.1	3.6	55.6	129.5	1.0	14.3	109.9	0.8	16.8	90.4
2008–2012	1.8	33.4	102.5	0.8	30.5	77.1	1.8	19.6	136.6	1.2	18.5	103.9
	35–44			>74 y/o			35–44			>74 y/o		
1983–1987	2.2	30.8	96.8	1.6	80.8	103.1	0.4	10.1	85.3	1.8	60.7	105.3
1988–1992	3.0	30.9	130.5	1.2	59.0	91.5	0.8	10.0	103.2	1.8	47.8	99.9
1993–1997	1.4	22.8	93.0	1.2	49.7	95.5	0.8	9.5	112.1	1.4	32.0	92.9
1998–2002	1.6	31.6	89.7	2.4	64.8	122.9	0.2	8.6	83.1	1.4	26.9	86.7
2003–2007	2.8	45.6	113.9	2.6	56.2	124.9	0.8	13.1	103.6	1.6	22.9	102.0
2008–2012	1.4	32.9	88.4	2.0	42.7	103.0	0.2	11.4	84.4	1.4	18.5	96.2

Secondary Medical Zone ID: 272

	Male						Female					
	Suicide			Suicide			Suicide			Suicide		
	Num per year	Rate /100,000	×100	Num per year	Rate /100,000	×100	Num per year	Rate /100,000	×100	Num per year	Rate /100,000	×100
	Total (>10 y/o)			45–54			Total (>10 y/o)			45–54		
1983–1987	32.8	33.1	99.2	8.8	54.6	108.0	18.0	15.1	96.6	3.0	17.0	98.9
1988–1992	29.6	26.2	109.1	7.4	42.5	123.7	13.2	11.2	79.8	2.2	13.7	93.8
1993–1997	25.8	24.2	92.0	7.4	39.1	106.8	14.4	10.5	94.2	3.2	15.0	115.9
1998–2002	37.2	33.5	91.1	10.4	56.9	101.6	15.8	12.4	90.2	3.8	17.6	117.0
2003–2007	42.8	37.7	103.8	8.4	54.9	98.1	15.2	12.6	92.5	1.4	11.4	83.1
2008–2012	36.8	34.2	98.6	5.0	40.3	83.0	14.6	12.7	92.7	1.4	12.6	84.3
	15–24			55–64			15–24			55–64		
1983–1987	2.0	14.6	104.6	3.4	29.8	70.0	1.4	8.2	119.3	1.6	13.8	69.9
1988–1992	1.8	11.1	112.2	6.8	41.6	117.2	0.6	4.9	92.5	2.6	16.1	90.4
1993–1997	2.2	13.2	116.9	5.6	38.2	95.0	0.6	4.9	94.7	1.2	10.7	67.0
1998–2002	2.6	18.5	117.4	8.4	57.1	89.9	0.8	7.0	100.0	3.6	20.0	106.1
2003–2007	2.2	19.2	110.0	9.4	54.6	94.6	1.8	12.8	142.2	3.2	17.0	101.5
2008–2012	1.6	19.7	92.8	9.8	53.0	109.2	1.0	10.6	108.8	3.6	16.9	113.3
	25–34			65–74			25–34			65–74		
1983–1987	5.8	33.8	132.2	1.6	27.4	62.6	2.4	12.8	118.9	4.4	37.7	120.6
1988–1992	2.0	16.2	84.8	3.0	31.7	90.0	1.0	7.9	90.8	1.4	15.1	58.0
1993–1997	3.0	20.8	106.6	1.6	18.8	59.0	1.4	9.0	108.7	2.0	15.1	77.5
1998–2002	3.8	26.4	97.9	5.0	40.1	88.2	1.6	11.4	98.3	2.2	16.1	76.7
2003–2007	4.2	29.8	96.2	6.2	45.7	106.6	1.0	10.4	79.9	2.6	17.0	91.5
2008–2012	5.0	36.0	110.4	6.0	42.8	108.4	1.6	13.9	96.8	1.8	14.5	81.7
	35–44			>74 y/o			35–44			>74 y/o		
1983–1987	9.0	42.6	133.8	2.0	52.9	67.6	2.2	11.5	97.8	3.0	45.5	78.9
1988–1992	5.2	26.5	112.0	3.4	58.8	91.2	1.8	9.6	99.0	3.6	39.7	83.0
1993–1997	4.2	26.0	106.0	1.8	32.1	61.6	2.0	10.3	121.0	4.0	33.8	98.3
1998–2002	3.0	25.6	72.6	4.0	48.2	91.4	2.0	11.9	115.0	1.8	15.4	49.7
2003–2007	7.2	48.2	120.3	5.0	46.5	103.3	2.6	15.2	120.2	2.4	14.9	66.3
2008–2012	6.2	39.4	106.0	3.2	30.7	73.9	2.4	14.7	108.3	2.8	14.7	76.6

Secondary Medical Zone ID: 273

	Male						Female					
	Suicide			Suicide			Suicide			Suicide		
	Num per year	Rate /100,000	×100	Num per year	Rate /100,000	×100	Num per year	Rate /100,000	×100	Num per year	Rate /100,000	×100
	Total (>10 y/o)			45–54			Total (>10 y/o)			45–54		
1983–1987	31.4	35.1	111.7	9.0	63.2	125.0	21.2	17.3	120.8	3.6	21.2	122.9
1988–1992	24.6	27.1	110.7	6.0	40.9	119.0	14.2	12.6	94.5	2.4	15.4	105.0
1993–1997	24.0	26.0	104.9	6.2	39.2	107.1	12.8	10.8	98.0	1.6	11.6	89.6
1998–2002	29.4	33.8	91.8	6.8	49.7	88.7	13.4	11.8	92.3	2.4	15.3	101.8
2003–2007	31.2	37.2	99.6	8.8	70.9	126.8	13.2	13.0	98.2	1.8	14.0	102.5
2008–2012	22.4	32.0	84.7	4.8	49.6	102.0	12.4	13.3	98.6	1.8	15.9	106.5
	15–24			55–64			15–24			55–64		
1983–1987	1.0	11.7	83.9	4.4	40.6	95.5	1.2	7.8	114.0	2.6	19.2	97.5
1988–1992	2.6	14.7	148.1	3.8	32.5	91.5	0.8	5.6	106.6	3.4	20.9	117.2
1993–1997	2.0	14.0	123.5	5.2	41.4	102.9	0.4	4.6	88.0	2.4	16.6	104.3
1998–2002	1.4	15.7	99.5	8.4	65.1	102.5	0.2	5.5	78.8	4.2	23.6	125.4
2003–2007	1.0	16.2	93.1	7.2	52.5	90.9	0.4	7.8	86.4	2.8	17.2	103.0
2008–2012	1.4	21.5	101.1	6.2	46.0	94.8	1.4	13.7	141.7	2.8	16.4	109.9
	25–34			65–74			25–34			65–74		
1983–1987	3.2	26.2	102.6	3.2	45.4	103.6	2.4	14.1	131.3	3.4	33.1	105.9
1988–1992	3.8	29.3	153.1	1.8	27.5	78.2	1.6	10.7	122.9	1.8	19.1	73.3
1993–1997	2.2	21.0	107.6	2.6	28.9	90.5	1.4	9.9	118.5	2.4	19.0	97.6
1998–2002	4.0	32.2	119.1	4.2	42.0	92.4	0.4	8.6	74.2	2.2	18.4	87.5
2003–2007	2.8	29.1	94.0	4.4	41.7	97.3	1.8	14.5	111.3	2.2	17.7	94.9
2008–2012	2.4	30.9	94.8	1.6	25.0	63.4	0.8	12.8	89.0	2.0	16.5	92.8
	35–44			>74 y/o			35–44			>74 y/o		
1983–1987	7.4	41.0	128.8	3.2	73.1	93.3	4.0	17.4	147.4	3.6	51.9	90.0
1988–1992	4.4	27.1	114.5	2.2	48.3	74.9	1.6	9.9	102.0	2.2	28.6	59.7
1993–1997	3.4	26.9	109.6	2.2	42.0	80.7	1.6	10.2	120.0	3.0	29.2	85.0
1998–2002	2.0	25.8	73.2	2.6	42.9	81.4	1.0	9.9	95.5	3.0	24.9	80.1
2003–2007	3.6	38.2	95.2	3.2	40.8	90.7	1.8	14.3	113.0	2.0	15.2	67.9
2008–2012	2.6	31.4	84.5	3.2	36.5	87.9	1.4	13.3	98.0	2.2	14.9	77.7

Secondary Medical Zone ID: 274

	Male						Female					
	Suicide			Suicide			Suicide			Suicide		
	Num per year	Rate /100,000	×100	Num per year	Rate /100,000	×100	Num per year	Rate /100,000	×100	Num per year	Rate /100,000	×100
	Total (>10 y/o)			45–54			Total (>10 y/o)			45–54		
1983–1987	88.2	37.1	116.2	22.6	62.4	123.4	49.6	16.9	114.5	8.8	20.8	120.5
1988–1992	72.2	27.6	114.9	17.0	42.8	124.8	43.2	13.4	105.3	7.8	17.3	118.1
1993–1997	67.4	24.7	98.0	20.0	43.0	117.4	35.0	10.6	93.8	6.0	12.6	97.3
1998–2002	102.6	35.2	98.3	27.4	58.1	103.8	42.4	11.9	93.1	7.8	15.4	102.5
2003–2007	120.2	40.4	110.9	27.0	63.5	113.5	44.4	12.6	98.2	5.8	12.9	94.4
2008–2012	100.6	34.9	100.7	19.2	50.7	104.2	43.4	12.7	96.3	5.2	13.0	86.8
	15–24			55–64			15–24			55–64		
1983–1987	4.8	12.5	89.9	10.2	36.6	86.1	2.8	6.7	97.4	10.0	26.5	134.4
1988–1992	5.0	10.9	109.8	14.0	40.9	115.2	3.2	6.3	119.5	7.2	18.3	102.3
1993–1997	3.0	7.9	70.2	13.8	40.0	99.5	2.4	5.0	96.7	5.4	14.0	88.4
1998–2002	7.6	17.4	110.4	20.2	55.2	86.9	2.4	6.0	85.3	7.4	17.9	95.0
2003–2007	7.4	19.0	108.8	28.2	63.7	110.3	5.0	11.9	132.0	8.8	17.9	106.7
2008–2012	9.4	26.5	125.0	20.8	46.0	94.7	2.4	7.9	81.4	7.4	14.8	99.2
	25–34			65–74			25–34			65–74		
1983–1987	12.2	29.7	116.3	9.0	54.4	124.3	4.4	10.2	94.4	8.0	35.8	114.5
1988–1992	7.4	20.0	104.6	7.0	35.4	100.6	5.0	10.9	126.2	7.2	26.8	102.9
1993–1997	7.4	19.7	100.7	6.8	27.6	86.4	4.8	10.3	123.9	4.6	14.9	76.4
1998–2002	13.2	30.7	113.7	10.8	38.3	84.2	4.8	10.8	93.4	7.4	20.1	95.7
2003–2007	12.6	30.2	97.5	14.6	46.1	107.5	6.6	14.1	108.8	6.6	17.5	93.9
2008–2012	12.0	33.0	101.4	13.8	41.1	103.9	6.8	16.3	113.8	8.2	19.8	111.2
	35–44			>74 y/o			35–44			>74 y/o		
1983–1987	21.2	44.1	138.5	7.8	78.5	100.3	8.6	16.0	135.4	6.8	46.9	81.4
1988–1992	15.8	32.1	135.3	5.8	51.0	79.1	5.6	10.7	111.2	7.2	37.0	77.3
1993–1997	10.6	24.8	101.3	5.8	43.6	83.7	5.4	10.9	128.2	6.2	26.1	75.8
1998–2002	12.4	32.0	91.0	10.8	60.9	115.5	4.2	9.9	95.8	8.2	26.8	86.3
2003–2007	21.0	51.8	129.3	9.2	41.3	91.6	3.8	9.9	78.7	7.8	20.0	88.9
2008–2012	15.0	35.9	96.5	10.2	38.0	91.7	7.0	14.8	109.2	6.4	14.2	73.7

Secondary Medical Zone ID: 275

	Male						Female					
	Suicide			Suicide			Suicide			Suicide		
	Num per year	Rate /100,000	×100	Num per year	Rate /100,000	×100	Num per year	Rate /100,000	×100	Num per year	Rate /100,000	×100
	Total (>10 y/o)			45-54			Total (>10 y/o)			45-54		
1983-1987	44.2	42.7	147.4	10.0	68.0	134.5	25.0	18.6	133.1	3.6	20.5	119.1
1988-1992	29.6	29.1	128.1	5.6	42.6	123.9	16.4	13.1	102.4	3.0	17.8	121.6
1993-1997	27.8	28.7	120.6	4.2	34.5	94.4	14.0	11.0	104.1	1.4	12.0	92.9
1998-2002	37.2	39.5	116.6	9.2	65.1	116.3	19.0	14.2	121.9	3.2	18.9	125.6
2003-2007	32.8	38.3	109.1	7.4	61.6	110.1	15.0	13.3	111.2	2.0	15.2	111.0
2008-2012	26.0	34.1	100.6	5.4	52.6	108.3	9.8	12.2	89.9	1.0	13.3	89.2
	15-24			55-64			15-24			55-64		
1983-1987	2.0	17.0	122.0	7.2	50.3	118.5	1.0	7.7	113.3	4.4	23.9	121.2
1988-1992	0.6	8.8	89.2	6.2	41.7	117.4	0.4	4.9	93.0	2.8	17.4	97.6
1993-1997	1.6	13.5	119.1	6.8	49.2	122.2	0.0	3.7	71.5	1.6	13.2	82.8
1998-2002	1.4	16.6	104.9	8.4	71.0	111.8	0.6	7.2	103.1	2.6	19.3	102.3
2003-2007	1.2	17.8	102.2	6.2	53.2	92.1	1.0	11.0	122.4	2.4	17.5	104.4
2008-2012	0.8	19.6	92.2	6.2	48.2	99.1	0.8	11.3	116.0	1.4	13.3	89.5
	25-34			65-74			25-34			65-74		
1983-1987	5.6	38.0	148.5	6.0	63.4	144.7	2.6	15.6	144.7	3.4	30.4	97.4
1988-1992	2.8	24.1	126.3	5.0	46.4	131.6	1.6	11.2	129.1	2.8	22.8	87.3
1993-1997	2.8	25.7	131.5	3.4	30.5	95.5	1.0	9.2	110.8	3.2	20.8	106.8
1998-2002	2.4	28.0	103.8	5.4	44.9	98.7	1.2	12.4	107.0	3.2	21.0	99.9
2003-2007	3.4	36.8	118.5	5.2	45.5	106.0	0.8	12.2	93.9	2.8	19.5	104.6
2008-2012	1.8	30.8	94.6	3.0	34.4	87.2	0.2	11.4	79.5	2.6	19.3	108.6
	35-44			>74 y/o			35-44			>74 y/o		
1983-1987	6.0	40.3	126.7	7.2	123.3	157.5	2.6	14.9	126.3	7.4	86.5	149.9
1988-1992	4.6	29.9	126.3	4.8	75.6	117.2	1.4	10.0	103.3	4.4	43.9	91.8
1993-1997	4.8	34.4	140.5	4.2	59.6	114.5	2.0	12.0	140.9	4.6	37.6	109.4
1998-2002	4.6	41.7	118.5	5.4	62.8	119.2	1.8	13.2	128.0	6.4	41.9	135.0
2003-2007	3.6	41.2	102.8	5.8	54.6	121.1	1.0	12.2	96.8	4.8	26.5	118.0
2008-2012	2.4	34.4	92.4	6.4	52.3	126.0	0.6	11.8	86.7	3.2	17.2	89.6

Secondary Medical Zone ID: 276

	Male						Female					
	Suicide			Suicide			Suicide			Suicide		
	Num per year	Rate /100,000	×100	Num per year	Rate /100,000	×100	Num per year	Rate /100,000	×100	Num per year	Rate /100,000	×100
	Total (>10 y/o)			45-54			Total (>10 y/o)			45-54		
1983-1987	30.2	39.0	129.9	7.6	65.9	130.3	16.4	16.5	113.7	2.8	20.1	116.4
1988-1992	20.8	27.4	116.8	4.8	43.7	127.3	12.2	12.8	98.5	2.0	16.0	109.4
1993-1997	20.4	27.1	113.0	6.2	47.5	129.8	10.8	11.0	102.3	1.4	12.7	98.2
1998-2002	31.2	41.1	121.9	9.6	74.5	133.0	9.8	12.1	89.2	1.6	14.7	97.4
2003-2007	29.2	41.6	121.3	7.8	75.3	134.6	9.4	12.5	94.7	1.4	13.9	101.7
2008-2012	22.6	35.1	108.4	2.2	39.0	80.3	8.6	12.5	95.0	0.8	13.3	89.4
	15-24			55-64			15-24			55-64		
1983-1987	1.8	18.0	129.0	5.2	48.6	114.4	0.8	7.7	112.9	3.8	25.5	129.3
1988-1992	1.4	12.3	124.2	3.0	31.2	87.8	0.2	4.6	86.7	1.8	16.2	90.8
1993-1997	0.6	10.5	92.9	6.2	53.9	133.9	0.0	3.9	74.9	1.8	15.7	99.0
1998-2002	0.6	14.0	88.6	9.2	87.8	138.2	0.0	5.5	77.8	2.8	21.8	115.7
2003-2007	0.6	16.2	93.0	9.6	81.3	140.9	0.4	8.8	98.0	1.2	14.6	87.3
2008-2012	0.4	18.8	88.6	6.4	53.8	110.6	0.2	8.6	89.1	1.8	15.2	102.0
	25-34			65-74			25-34			65-74		
1983-1987	5.8	43.0	168.4	3.4	51.8	118.2	1.2	11.0	102.6	2.8	31.7	101.5
1988-1992	1.0	15.9	83.1	2.4	34.7	98.6	1.0	9.6	110.7	2.6	26.0	99.8
1993-1997	1.2	18.6	95.3	1.6	24.0	75.1	0.2	6.8	81.5	2.4	20.6	105.5
1998-2002	2.4	30.1	111.4	2.6	35.8	78.8	0.4	10.1	86.8	1.6	17.0	80.9
2003-2007	2.0	31.3	101.0	3.4	41.4	96.5	1.4	15.5	119.5	2.2	19.4	104.4
2008-2012	1.8	33.3	102.2	3.6	42.9	108.6	0.2	12.1	84.3	1.8	18.0	101.2
	35-44			>74 y/o			35-44			>74 y/o		
1983-1987	4.4	35.5	111.6	2.0	61.1	78.0	1.8	13.1	110.9	3.2	53.2	92.1
1988-1992	4.8	33.4	141.0	3.4	74.0	114.7	1.8	11.6	119.8	2.8	38.7	80.9
1993-1997	2.8	26.7	109.0	1.6	40.0	76.9	2.0	12.6	148.3	3.0	33.7	97.8
1998-2002	3.8	42.1	119.4	3.0	52.9	100.4	1.4	12.4	120.4	2.0	21.2	68.4
2003-2007	3.6	47.7	119.1	2.2	36.1	80.2	0.6	11.4	90.5	2.2	18.2	81.2
2008-2012	3.8	44.7	120.1	4.4	49.0	118.2	1.4	15.3	113.2	2.4	17.2	89.7

Secondary Medical Zone ID: 277

	Male						Female					
	Suicide			Suicide			Suicide			Suicide		
	Num per year	Rate /100,000	×100	Num per year	Rate /100,000	×100	Num per year	Rate /100,000	×100	Num per year	Rate /100,000	×100
	Total (>10 y/o)			45–54			Total (>10 y/o)			45–54		
1983–1987	14.8	36.7	127.9	3.4	57.4	113.4	5.6	14.4	91.5	1.0	17.4	101.0
1988–1992	10.6	26.8	119.8	2.4	40.4	117.5	4.0	11.7	80.6	0.6	14.0	95.7
1993–1997	12.6	29.5	134.8	3.0	44.4	121.3	4.6	10.6	96.7	1.0	14.3	110.4
1998–2002	12.8	37.6	108.5	2.2	51.2	91.4	3.8	11.7	85.7	1.0	16.1	106.9
2003–2007	15.6	41.9	133.6	3.8	75.3	134.6	4.0	12.4	94.6	1.0	15.8	115.0
2008–2012	13.2	37.2	125.2	2.6	58.4	120.1	4.2	13.2	100.6	0.8	16.6	111.5
	15–24			55–64			15–24			55–64		
1983–1987	0.4	13.3	95.2	3.6	57.1	134.3	0.2	6.6	97.1	1.4	21.1	107.2
1988–1992	0.8	11.9	120.4	2.0	37.1	104.3	0.2	5.4	102.3	0.4	14.6	81.7
1993–1997	0.0	9.9	87.1	4.0	56.8	141.1	0.0	4.6	88.5	0.8	15.6	98.0
1998–2002	0.0	13.5	85.7	4.2	76.0	119.6	0.0	6.3	90.2	0.8	18.2	96.5
2003–2007	0.6	18.8	107.7	4.6	75.0	129.9	0.2	9.1	101.4	0.4	14.9	88.8
2008–2012	0.4	21.5	101.6	3.6	57.7	118.7	0.2	10.0	102.6	0.2	12.9	86.2
	25–34			65–74			25–34			65–74		
1983–1987	2.0	32.6	127.4	0.8	37.3	85.2	0.0	8.3	77.4	1.8	35.5	113.7
1988–1992	0.4	16.7	87.4	1.6	40.2	114.0	0.8	10.9	125.5	1.2	25.3	97.1
1993–1997	0.8	21.1	108.1	0.8	26.8	83.9	0.0	7.2	86.5	1.2	20.8	106.6
1998–2002	1.0	28.7	106.3	1.6	42.0	92.4	0.0	10.2	87.6	1.0	20.1	95.9
2003–2007	1.4	36.0	115.9	2.2	46.6	108.7	0.4	13.4	103.0	0.4	15.4	82.8
2008–2012	1.0	34.5	106.0	2.6	48.1	121.6	0.6	16.0	111.5	0.8	17.7	99.3
	35–44			>74 y/o			35–44			>74 y/o		
1983–1987	3.0	41.3	129.9	1.4	80.5	102.7	0.6	11.6	98.2	0.6	34.4	59.6
1988–1992	2.4	32.2	135.9	1.0	57.6	89.3	0.4	9.3	96.2	0.4	23.3	48.6
1993–1997	3.0	41.7	170.1	1.0	48.6	93.3	0.4	8.7	102.6	1.2	29.4	85.3
1998–2002	2.6	49.3	139.9	0.8	42.5	80.7	0.0	8.6	83.4	1.0	23.4	75.4
2003–2007	1.6	45.6	113.8	1.4	43.9	97.5	0.2	11.8	93.7	1.4	22.8	101.8
2008–2012	1.0	37.4	100.4	2.0	46.2	111.4	0.4	13.7	101.3	1.2	18.6	96.7

Secondary Medical Zone ID: 278

	Male						Female					
	Suicide			Suicide			Suicide			Suicide		
	Num per year	Rate /100,000	×100	Num per year	Rate /100,000	×100	Num per year	Rate /100,000	×100	Num per year	Rate /100,000	×100
	Total (>10 y/o)			45–54			Total (>10 y/o)			45–54		
1983–1987	102.2	42.0	136.2	24.6	68.8	136.1	46.4	16.1	105.7	8.2	20.1	116.5
1988–1992	81.6	31.5	133.6	16.6	44.5	129.6	42.6	13.2	101.9	5.6	14.4	98.3
1993–1997	70.4	26.8	108.7	18.2	42.4	115.8	38.6	11.7	103.5	6.6	14.4	111.5
1998–2002	103.2	37.4	107.1	29.2	67.2	120.1	40.8	12.1	94.0	8.2	17.6	116.9
2003–2007	110.8	40.4	114.4	26.0	70.2	125.4	44.6	13.4	106.9	7.4	17.6	128.3
2008–2012	100.8	38.0	115.1	20.2	60.6	124.7	37.8	12.6	96.0	5.4	15.6	104.2
	15–24			55–64			15–24			55–64		
1983–1987	4.0	13.1	93.7	17.2	53.4	125.7	3.0	8.1	118.5	9.4	23.6	119.8
1988–1992	3.8	10.6	107.1	15.0	42.8	120.4	1.6	4.7	89.2	9.4	22.1	123.8
1993–1997	3.8	10.8	95.1	13.0	39.4	97.9	1.6	4.6	89.0	7.0	17.6	110.6
1998–2002	3.8	12.6	79.6	22.2	63.9	100.5	1.4	5.3	74.9	9.0	21.6	115.0
2003–2007	4.2	15.2	87.0	31.6	75.8	131.3	3.6	11.1	123.8	6.4	15.3	91.2
2008–2012	3.4	16.0	75.6	27.6	63.7	131.2	2.2	9.2	94.6	7.0	15.7	105.0
	25–34			65–74			25–34			65–74		
1983–1987	11.4	31.0	121.4	10.6	56.8	129.8	4.8	12.2	113.7	7.6	30.3	96.8
1988–1992	8.6	25.7	134.5	11.4	49.2	139.7	2.8	8.4	96.8	10.8	33.8	129.5
1993–1997	7.2	22.3	114.4	10.8	38.6	121.1	3.4	9.4	113.3	7.4	20.4	104.7
1998–2002	7.6	23.4	86.6	15.6	50.6	111.3	4.0	11.3	97.1	6.2	17.1	81.4
2003–2007	11.0	31.4	101.2	14.8	48.7	113.4	4.8	13.2	101.8	8.2	21.2	113.9
2008–2012	10.4	34.8	106.7	14.4	45.9	116.3	3.6	12.8	89.1	6.8	18.3	102.8
	35–44			>74 y/o			35–44			>74 y/o		
1983–1987	24.6	53.2	167.3	9.8	89.9	114.7	5.6	12.2	103.6	7.6	46.4	80.5
1988–1992	16.4	36.2	152.9	9.8	72.0	111.7	4.8	10.5	109.0	7.6	32.8	68.6
1993–1997	9.0	24.7	100.7	8.4	54.0	103.7	5.6	12.8	150.0	7.0	24.5	71.2
1998–2002	12.8	38.1	108.1	11.6	59.5	112.8	2.6	8.4	81.1	9.4	26.2	84.3
2003–2007	12.8	39.8	99.3	10.4	43.3	96.2	4.0	12.0	94.6	10.2	22.8	101.4
2008–2012	13.0	37.7	101.5	11.4	41.1	99.1	4.8	13.4	98.6	7.8	15.7	81.8

Secondary Medical Zone ID: 279

	Male						Female					
	Suicide			Suicide			Suicide			Suicide		
	Num per year	Rate /100,000	×100	Num per year	Rate /100,000	×100	Num per year	Rate /100,000	×100	Num per year	Rate /100,000	×100
	Total (>10 y/o)			45–54			Total (>10 y/o)			45–54		
1983–1987	16.4	37.1	129.6	3.8	60.9	120.4	8.2	16.1	111.0	1.2	18.2	105.9
1988–1992	14.6	29.6	142.4	2.8	44.1	128.4	5.4	12.2	90.9	1.0	15.6	106.8
1993–1997	14.0	29.2	136.1	3.4	46.8	127.9	5.0	10.7	96.7	1.0	14.3	110.7
1998–2002	15.4	38.4	115.3	3.2	57.6	102.9	5.4	12.4	96.1	1.4	17.7	117.6
2003–2007	20.2	46.1	150.8	4.6	78.8	140.8	6.2	13.3	110.2	1.0	15.5	113.1
2008–2012	11.2	34.6	104.5	2.2	52.8	108.7	3.0	12.1	87.0	0.2	13.3	89.1
	15–24			55–64			15–24			55–64		
1983–1987	0.8	16.2	116.4	3.6	54.0	127.0	0.0	5.9	85.7	1.0	18.3	92.8
1988–1992	0.4	10.0	101.2	2.6	40.4	113.9	0.0	4.5	86.0	0.0	12.2	68.1
1993–1997	0.0	9.5	84.1	3.6	52.8	131.1	0.2	5.3	101.7	0.6	14.2	89.3
1998–2002	0.8	17.3	109.2	3.6	70.3	110.6	0.2	7.0	99.7	0.4	16.3	86.5
2003–2007	0.4	17.0	97.2	6.2	89.0	154.3	0.2	8.9	98.8	1.6	19.7	117.9
2008–2012	0.6	22.1	104.1	2.8	50.1	103.1	0.0	8.5	88.0	0.0	12.1	80.9
	25–34			65–74			25–34			65–74		
1983–1987	1.8	29.8	116.5	0.8	36.0	82.2	0.4	10.2	94.9	2.2	39.3	125.8
1988–1992	1.4	24.6	128.9	2.2	44.7	126.8	0.6	9.9	113.8	1.0	22.7	87.1
1993–1997	1.6	26.7	136.9	1.6	33.0	103.4	0.4	8.9	106.9	0.6	15.3	78.3
1998–2002	1.2	29.3	108.7	2.2	44.9	98.8	0.2	10.9	94.2	0.8	18.0	85.5
2003–2007	1.2	33.0	106.6	1.4	37.7	88.0	0.4	13.2	101.3	1.0	18.6	99.7
2008–2012	0.2	28.2	86.5	1.8	41.0	103.8	0.0	12.7	88.9	1.0	18.5	103.9
	35–44			>74 y/o			35–44			>74 y/o		
1983–1987	2.4	37.0	116.1	3.0	106.4	135.8	1.4	15.0	126.9	2.0	60.2	104.4
1988–1992	2.8	34.3	144.6	2.4	79.3	122.9	0.6	10.0	103.9	2.2	50.6	105.8
1993–1997	1.6	28.1	114.5	2.2	66.1	126.9	0.6	9.5	112.0	1.6	32.5	94.4
1998–2002	2.2	43.5	123.4	2.2	59.2	112.3	0.4	10.4	100.8	2.0	32.2	103.7
2003–2007	3.6	68.0	169.6	2.6	53.9	119.6	0.6	13.6	107.7	1.4	20.4	90.9
2008–2012	1.0	36.4	97.8	2.6	47.6	114.7	0.4	13.6	100.1	1.4	18.0	93.7

Secondary Medical Zone ID: 280

	Male						Female					
	Suicide			Suicide			Suicide			Suicide		
	Num per year	Rate /100,000	×100	Num per year	Rate /100,000	×100	Num per year	Rate /100,000	×100	Num per year	Rate /100,000	×100
	Total (>10 y/o)			45–54			Total (>10 y/o)			45–54		
1983–1987	23.4	39.0	137.6	4.4	56.0	110.8	8.6	14.4	91.5	1.0	15.4	89.6
1988–1992	17.4	28.4	129.5	3.6	43.0	125.1	8.0	12.2	92.2	1.0	14.2	97.4
1993–1997	14.4	26.3	108.4	2.2	32.4	88.4	8.2	10.9	104.5	0.6	11.3	87.2
1998–2002	26.0	41.5	131.0	6.6	69.0	123.3	7.2	12.0	89.4	0.6	12.7	84.5
2003–2007	28.6	45.2	148.3	7.0	83.2	148.8	8.0	12.5	101.9	0.6	12.3	89.5
2008–2012	26.0	41.0	145.8	5.8	72.5	149.2	8.2	13.1	107.2	1.4	17.4	116.3
	15–24			55–64			15–24			55–64		
1983–1987	1.0	15.9	114.0	4.0	50.4	118.5	1.0	9.3	135.7	2.2	22.0	111.7
1988–1992	1.2	12.6	127.6	2.6	34.7	97.6	0.2	5.0	95.0	1.0	15.3	85.6
1993–1997	0.6	11.4	100.5	3.2	42.7	106.1	0.2	5.0	95.8	1.6	17.4	109.7
1998–2002	0.8	16.2	102.3	3.8	59.5	93.7	0.2	6.6	94.1	1.2	17.9	95.0
2003–2007	1.0	19.3	110.9	7.8	82.6	143.2	0.2	8.3	92.7	2.2	19.7	117.8
2008–2012	0.6	21.2	99.8	7.4	68.0	139.9	0.2	9.3	96.0	1.6	16.1	107.7
	25–34			65–74			25–34			65–74		
1983–1987	3.2	34.6	135.3	2.4	50.6	115.5	0.6	9.9	91.9	1.4	26.9	86.0
1988–1992	2.2	27.2	142.2	3.0	47.3	134.4	0.2	7.3	84.6	1.4	22.1	84.8
1993–1997	1.4	22.3	114.3	1.4	26.8	84.1	0.6	9.0	107.6	1.0	15.5	79.3
1998–2002	3.4	38.9	144.0	4.8	58.0	127.6	0.8	12.4	106.6	1.6	20.0	95.0
2003–2007	2.6	37.8	121.9	5.2	62.6	145.9	0.8	13.7	105.2	2.2	22.4	120.3
2008–2012	2.4	38.7	118.9	2.8	42.6	107.8	0.2	12.6	88.1	1.4	18.3	103.1
	35–44			>74 y/o			35–44			>74 y/o		
1983–1987	5.2	47.8	150.1	3.0	95.0	121.2	1.0	11.8	100.2	1.4	38.5	66.8
1988–1992	2.0	23.6	99.7	2.6	73.9	114.6	1.2	10.9	113.0	3.0	50.9	106.4
1993–1997	2.6	30.2	123.2	3.0	70.9	136.2	0.8	9.3	108.9	3.4	45.3	131.5
1998–2002	3.2	44.1	125.2	3.4	67.1	127.2	0.6	10.2	98.9	2.2	27.1	87.4
2003–2007	2.2	41.6	103.9	2.8	48.5	107.8	0.4	11.5	91.2	1.6	17.6	78.4
2008–2012	2.4	40.3	108.4	4.6	58.9	142.0	0.8	14.0	103.2	2.6	21.4	111.4

Secondary Medical Zone ID: 281

	Male						Female					
	Suicide			Suicide			Suicide			Suicide		
	Num per year	Rate /100,000	×100	Num per year	Rate /100,000	×100	Num per year	Rate /100,000	×100	Num per year	Rate /100,000	×100
	Total (>10 y/o)			45–54			Total (>10 y/o)			45–54		
1983–1987	169.8	36.9	114.8	47.8	67.5	133.5	73.2	14.5	95.0	13.8	17.8	103.2
1988–1992	136.4	26.6	109.0	32.8	41.4	120.5	68.0	11.6	89.8	12.4	14.4	98.7
1993–1997	159.2	27.6	111.0	42.0	43.5	118.9	69.8	10.5	95.9	14.6	14.2	110.0
1998–2002	256.4	40.4	114.3	68.6	66.9	119.6	92.8	12.8	99.5	18.2	16.8	111.7
2003–2007	270.0	40.8	112.4	68.0	71.9	128.5	92.8	12.3	95.5	14.2	14.2	103.9
2008–2012	245.2	35.7	105.3	40.0	44.6	91.7	107.2	13.5	103.6	17.8	17.9	119.8
	15–24			55–64			15–24			55–64		
1983–1987	12.8	12.3	88.1	23.4	49.2	115.8	5.6	6.2	90.6	10.8	19.0	96.2
1988–1992	9.8	8.2	82.8	28.4	47.3	133.3	6.2	5.6	105.7	12.0	17.8	99.8
1993–1997	12.0	9.6	84.8	32.0	47.7	118.4	5.8	5.0	96.3	10.4	14.1	88.8
1998–2002	16.8	14.2	89.8	64.2	85.2	134.1	7.6	6.8	97.0	19.8	22.8	121.3
2003–2007	14.6	14.2	81.4	63.8	70.4	122.0	8.8	8.7	96.7	17.4	17.5	104.4
2008–2012	16.4	18.8	88.7	58.2	57.5	118.3	9.2	10.2	105.4	18.0	16.3	109.5
	25–34			65–74			25–34			65–74		
1983–1987	27.0	27.5	107.4	11.6	47.0	107.4	11.8	11.6	108.0	9.6	27.8	88.9
1988–1992	17.8	19.0	99.3	9.8	32.5	92.3	7.8	8.0	92.5	8.8	20.7	79.2
1993–1997	20.0	20.0	102.6	14.2	34.7	108.8	10.4	9.6	115.1	8.6	16.3	83.5
1998–2002	30.0	26.6	98.6	29.2	56.3	123.7	12.4	10.5	90.6	14.4	22.4	106.8
2003–2007	34.8	30.4	97.9	27.4	47.2	109.9	16.6	13.3	102.5	10.6	15.3	82.4
2008–2012	38.2	36.6	112.4	32.4	49.1	124.3	16.2	13.9	96.7	13.8	17.3	97.2
	35–44			>74 y/o			35–44			>74 y/o		
1983–1987	37.6	38.3	120.5	8.8	70.8	90.4	11.2	10.9	92.6	10.2	50.3	87.2
1988–1992	26.6	25.7	108.3	10.8	63.9	99.0	10.8	10.0	103.1	9.6	31.9	66.6
1993–1997	28.4	29.5	120.3	10.6	52.1	100.0	10.2	10.0	118.2	9.0	23.3	67.6
1998–2002	35.6	38.8	110.1	11.8	44.9	85.2	11.0	11.3	109.0	9.4	19.0	61.0
2003–2007	45.4	45.9	114.6	15.4	43.2	95.9	13.2	12.6	99.4	12.0	18.4	82.0
2008–2012	43.2	38.4	103.3	16.2	36.2	87.4	18.0	14.6	108.0	14.2	17.5	90.9

Secondary Medical Zone ID: 282

	Male						Female					
	Suicide			Suicide			Suicide			Suicide		
	Num per year	Rate /100,000	×100	Num per year	Rate /100,000	×100	Num per year	Rate /100,000	×100	Num per year	Rate /100,000	×100
	Total (>10 y/o)			45–54			Total (>10 y/o)			45–54		
1983–1987	26.8	34.9	112.1	7.4	60.4	119.4	11.8	14.7	96.5	1.4	14.7	85.2
1988–1992	23.2	25.6	109.0	5.0	34.7	101.0	10.6	11.6	87.1	2.0	14.3	97.9
1993–1997	31.2	28.6	121.5	6.6	36.2	99.0	12.4	10.7	99.4	2.6	13.6	105.2
1998–2002	48.0	39.9	116.6	15.2	71.4	127.6	15.6	12.5	97.4	2.4	13.5	90.0
2003–2007	49.6	39.2	112.5	11.0	60.7	108.6	11.4	10.7	76.5	0.8	9.1	66.4
2008–2012	45.0	34.9	105.8	9.2	56.2	115.7	13.6	11.4	84.2	1.6	12.4	83.2
	15–24			55–64			15–24			55–64		
1983–1987	2.0	15.0	107.9	4.4	49.5	116.5	0.2	4.9	72.1	2.6	23.3	118.2
1988–1992	1.0	8.4	85.1	3.8	36.3	102.3	0.4	4.2	79.0	1.6	16.3	91.4
1993–1997	1.8	10.8	95.6	8.0	56.6	140.6	0.4	3.9	75.4	1.8	15.0	94.3
1998–2002	1.8	13.1	82.8	12.2	78.3	123.2	1.2	7.1	100.7	2.8	18.7	99.4
2003–2007	1.6	14.0	80.1	14.4	74.5	129.0	1.0	7.8	86.4	2.2	14.4	85.9
2008–2012	3.2	22.7	107.1	10.0	50.3	103.5	0.8	7.8	80.5	3.4	15.8	105.8
	25–34			65–74			25–34			65–74		
1983–1987	2.6	21.6	84.7	3.2	58.7	134.1	1.8	11.7	108.7	2.0	31.3	100.3
1988–1992	1.4	14.2	74.3	1.8	33.9	96.1	1.2	8.8	101.8	1.2	19.5	74.6
1993–1997	3.6	22.7	116.4	4.4	48.3	151.4	1.4	8.9	106.6	1.8	19.2	98.7
1998–2002	4.0	23.9	88.5	5.2	52.1	114.6	3.0	14.0	121.1	1.6	17.6	83.8
2003–2007	6.6	32.3	104.0	5.4	47.6	110.9	1.6	10.4	79.8	2.6	19.8	106.6
2008–2012	4.2	26.5	81.4	6.2	44.9	113.6	1.8	11.7	82.0	2.0	15.9	89.5
	35–44			>74 y/o			35–44			>74 y/o		
1983–1987	6.4	35.9	112.9	0.8	55.3	70.5	2.2	12.3	104.5	1.4	44.2	76.6
1988–1992	7.2	33.9	142.9	2.8	77.7	120.5	2.4	11.2	116.1	1.8	34.9	72.9
1993–1997	4.8	26.9	109.8	2.0	52.2	100.3	1.6	8.9	104.3	2.6	33.7	97.9
1998–2002	7.2	42.2	120.0	2.0	45.7	86.6	1.6	10.1	97.7	2.8	28.9	93.2
2003–2007	6.8	40.2	100.4	3.6	52.0	115.4	2.0	12.3	97.7	1.2	13.0	57.9
2008–2012	7.6	36.8	99.0	4.6	51.1	123.2	2.2	12.4	91.1	1.8	14.6	76.1

Secondary Medical Zone ID: 283

	Male						Female					
	Suicide			Suicide			Suicide			Suicide		
	Num per year	Rate /100,000	×100	Num per year	Rate /100,000	×100	Num per year	Rate /100,000	×100	Num per year	Rate /100,000	×100
	Total (>10 y/o)			45-54			Total (>10 y/o)			45-54		
1983-1987	14.6	32.9	98.5	4.2	55.5	109.7	5.8	13.8	81.5	0.6	13.7	79.8
1988-1992	13.8	25.4	104.6	3.8	40.1	116.8	8.4	12.8	100.4	1.2	14.2	96.8
1993-1997	17.8	26.6	113.1	4.2	38.4	105.1	8.2	10.9	100.1	0.6	10.1	78.3
1998-2002	25.2	36.6	104.0	4.8	47.5	84.9	11.4	13.0	106.7	2.8	18.0	119.7
2003-2007	26.0	36.4	103.4	6.6	62.9	112.4	10.0	12.7	97.4	1.6	13.9	101.2
2008-2012	25.2	35.1	105.3	3.8	46.1	94.9	9.0	12.0	91.3	0.6	11.5	77.3
	15-24			55-64			15-24			55-64		
1983-1987	0.8	12.1	86.8	2.0	36.7	86.3	0.4	6.3	92.8	0.8	16.7	84.7
1988-1992	0.6	8.9	90.0	2.4	33.7	95.0	0.6	5.5	105.2	1.8	19.4	108.5
1993-1997	0.6	9.5	83.7	4.8	50.9	126.4	0.8	5.9	113.0	1.0	14.0	87.9
1998-2002	2.2	18.2	115.3	6.2	66.0	103.8	1.0	7.8	110.7	1.4	17.1	90.9
2003-2007	3.4	24.7	141.8	5.6	53.3	92.3	0.8	8.7	96.6	1.4	14.9	88.8
2008-2012	1.6	21.2	100.0	5.6	48.4	99.7	0.2	6.9	71.6	1.6	14.0	93.8
	25-34			65-74			25-34			65-74		
1983-1987	2.0	27.3	106.6	0.6	32.9	75.1	0.6	9.7	90.5	1.2	30.1	96.4
1988-1992	1.2	19.4	101.6	1.4	33.5	95.1	1.2	10.9	126.0	1.0	22.2	85.2
1993-1997	2.0	23.7	121.4	2.6	37.8	118.4	1.0	9.6	114.8	1.8	22.6	116.1
1998-2002	2.8	30.5	113.0	3.4	47.3	104.0	0.8	10.8	93.1	2.4	24.8	118.2
2003-2007	2.0	27.6	89.1	3.0	41.4	96.6	1.6	14.5	111.6	1.8	19.4	104.1
2008-2012	3.6	37.2	114.3	4.2	44.0	111.5	1.0	13.4	93.5	2.0	19.0	106.6
	35-44			>74 y/o			35-44			>74 y/o		
1983-1987	2.6	30.1	94.5	2.4	108.5	138.5	0.8	10.5	88.8	1.4	55.4	96.1
1988-1992	2.6	25.3	106.6	1.8	69.2	107.2	1.0	9.5	98.8	1.6	42.4	88.6
1993-1997	1.8	21.5	87.8	1.8	56.7	109.0	1.2	9.6	113.1	1.6	31.6	91.8
1998-2002	3.4	38.0	107.9	2.2	54.4	103.1	0.8	9.6	92.7	2.2	32.0	103.0
2003-2007	2.0	30.9	77.1	3.2	53.4	118.7	1.4	13.5	106.9	1.4	17.9	79.7
2008-2012	3.4	38.0	102.2	3.0	44.3	106.8	1.4	13.9	102.6	2.2	19.8	103.2

Secondary Medical Zone ID: 284

	Male						Female					
	Suicide			Suicide			Suicide			Suicide		
	Num per year	Rate /100,000	×100	Num per year	Rate /100,000	×100	Num per year	Rate /100,000	×100	Num per year	Rate /100,000	×100
	Total (>10 y/o)			45–54			Total (>10 y/o)			45–54		
1983–1987	33.6	31.7	96.9	11.0	59.3	117.3	15.8	14.2	91.4	4.0	19.8	114.9
1988–1992	32.4	25.0	102.3	9.8	43.0	125.1	14.0	10.8	79.2	2.6	13.0	89.2
1993–1997	42.0	26.3	108.6	12.6	44.2	120.8	19.8	11.1	104.2	4.6	15.0	115.9
1998–2002	64.6	36.9	103.4	20.8	68.2	121.9	20.6	11.5	85.9	5.0	15.7	104.4
2003–2007	70.6	37.9	105.6	16.8	61.7	110.3	23.0	11.8	90.4	3.8	13.4	97.8
2008–2012	59.2	32.7	93.1	12.2	48.9	100.5	20.6	10.7	80.5	2.2	10.6	71.1
	15–24			55–64			15–24			55–64		
1983–1987	2.2	11.7	83.9	4.2	38.5	90.7	1.0	5.8	84.9	1.8	16.7	84.4
1988–1992	1.0	6.3	63.7	6.6	40.7	114.7	0.8	4.0	75.7	3.4	19.4	108.5
1993–1997	2.4	9.2	81.6	10.4	51.9	128.8	1.6	5.3	101.3	4.4	20.0	125.8
1998–2002	4.0	14.3	90.5	14.6	66.4	104.6	2.0	7.1	100.6	3.0	15.5	82.3
2003–2007	4.4	17.2	98.7	19.2	69.9	121.2	1.6	7.3	81.1	4.6	16.4	98.1
2008–2012	3.6	17.9	84.5	13.6	47.5	97.9	1.0	6.4	65.8	5.2	16.0	107.0
	25–34			65–74			25–34			65–74		
1983–1987	5.0	24.3	95.0	1.8	37.5	85.7	2.8	11.6	107.3	1.8	27.0	86.3
1988–1992	3.4	16.7	87.4	3.0	37.3	105.8	2.6	10.0	115.3	1.0	15.5	59.2
1993–1997	4.8	19.2	98.3	3.2	30.3	95.0	2.6	9.1	108.8	2.4	18.8	96.2
1998–2002	6.2	22.6	83.8	5.8	42.7	93.8	2.4	9.2	79.2	3.0	19.5	92.6
2003–2007	6.6	24.3	78.3	8.6	49.4	115.0	5.0	14.8	114.1	3.4	18.3	98.0
2008–2012	9.6	35.5	109.1	7.4	38.4	97.3	3.0	12.1	84.2	2.4	13.9	77.9
	35–44			>74 y/o			35–44			>74 y/o		
1983–1987	7.8	31.2	98.0	1.4	62.1	79.3	2.8	11.2	94.4	1.6	41.3	71.6
1988–1992	5.6	21.1	88.9	2.8	68.1	105.6	1.6	7.3	75.9	2.0	31.4	65.5
1993–1997	5.8	22.3	90.8	2.0	43.4	83.4	2.2	8.1	95.6	1.8	21.6	62.9
1998–2002	10.2	38.3	108.7	2.6	42.0	79.7	3.2	11.1	107.8	2.0	18.7	60.2
2003–2007	11.4	41.3	103.1	3.6	39.6	87.9	2.8	10.7	84.7	1.8	13.1	58.5
2008–2012	8.4	29.7	79.8	4.4	36.6	88.2	3.6	11.8	87.3	3.2	16.3	84.7

Secondary Medical Zone ID: 285

	Male						Female					
	Suicide			Suicide			Suicide			Suicide		
	Num per year	Rate /100,000	×100	Num per year	Rate /100,000	×100	Num per year	Rate /100,000	×100	Num per year	Rate /100,000	×100
	Total (>10 y/o)			45-54			Total (>10 y/o)			45-54		
1983-1987	15.2	36.1	118.9	4.2	64.2	126.9	6.6	14.6	93.2	1.0	16.8	97.6
1988-1992	12.4	26.9	119.6	3.2	45.2	131.6	4.4	11.1	76.7	0.6	13.5	92.4
1993-1997	11.4	26.5	106.9	1.4	30.5	83.3	4.8	10.1	87.1	0.6	11.9	92.4
1998-2002	17.4	37.7	109.5	5.2	64.7	115.7	4.8	11.4	81.4	0.8	14.0	93.2
2003-2007	19.2	39.5	119.2	4.2	62.1	111.0	6.2	12.6	96.8	0.4	11.8	85.9
2008-2012	15.0	35.6	104.9	3.2	54.5	112.2	5.2	12.2	92.1	0.4	13.0	87.1
	15-24			55-64			15-24			55-64		
1983-1987	1.2	17.1	122.9	2.0	41.4	97.4	0.4	6.9	101.2	1.2	19.7	99.9
1988-1992	1.0	11.7	118.2	2.6	41.2	115.9	0.4	5.6	106.6	0.8	16.1	90.3
1993-1997	0.0	8.7	77.3	2.8	45.4	112.9	0.2	4.8	92.6	1.2	17.0	106.7
1998-2002	1.0	16.4	103.7	3.2	61.2	96.4	0.0	5.6	79.5	1.0	18.1	96.3
2003-2007	0.8	17.2	98.7	4.8	66.8	115.8	0.2	7.7	86.1	1.4	17.7	105.9
2008-2012	0.8	21.0	98.9	3.6	49.0	100.9	0.4	9.7	99.7	1.0	14.5	97.4
	25-34			65-74			25-34			65-74		
1983-1987	1.8	27.6	108.0	1.6	47.2	107.9	0.6	10.5	97.4	1.2	29.7	94.9
1988-1992	0.8	18.2	95.4	1.4	36.8	104.3	0.0	6.7	77.8	0.6	19.2	73.6
1993-1997	2.0	27.2	139.3	1.6	34.1	107.0	0.2	7.5	89.7	1.0	18.6	95.2
1998-2002	1.8	29.9	110.6	2.2	45.5	100.1	0.4	10.8	93.3	1.2	20.4	97.1
2003-2007	1.8	32.4	104.6	3.2	53.6	124.9	0.8	13.6	104.9	0.8	16.7	89.9
2008-2012	2.4	37.8	116.0	1.2	34.2	86.7	0.4	13.2	91.9	1.0	17.6	99.0
	35-44			>74 y/o			35-44			>74 y/o		
1983-1987	3.6	42.9	134.6	0.8	57.9	73.9	1.4	14.3	121.3	0.8	35.1	60.8
1988-1992	2.8	31.3	132.0	0.6	43.6	67.6	0.8	10.2	105.2	1.2	33.8	70.7
1993-1997	3.0	35.4	144.5	0.6	36.1	69.2	0.4	7.9	93.4	1.2	26.4	76.8
1998-2002	2.2	38.2	108.4	1.8	53.9	102.3	0.0	7.8	75.3	1.4	24.9	80.3
2003-2007	2.4	45.1	112.6	1.8	45.6	101.2	1.2	15.0	118.9	1.4	20.0	89.2
2008-2012	1.8	37.1	99.8	2.0	43.3	104.4	0.6	13.3	97.8	1.4	17.9	93.3

Secondary Medical Zone ID: 286

	Male						Female					
	Suicide			Suicide			Suicide			Suicide		
	Num per year	Rate /100,000	×100	Num per year	Rate /100,000	×100	Num per year	Rate /100,000	×100	Num per year	Rate /100,000	×100
	Total (>10 y/o)			45–54			Total (>10 y/o)			45–54		
1983–1987	56.8	33.3	102.8	16.0	57.8	114.3	28.6	14.2	90.6	5.8	18.5	107.5
1988–1992	47.2	25.2	103.9	10.6	37.4	109.0	22.2	10.7	76.4	4.8	15.1	103.2
1993–1997	60.4	29.1	120.3	14.2	42.9	117.3	26.6	10.8	97.7	5.8	15.4	119.6
1998–2002	86.2	40.1	115.4	22.2	65.1	116.3	28.2	11.8	88.1	6.2	16.7	110.9
2003–2007	83.4	40.2	109.7	18.0	59.9	107.1	31.0	12.3	98.0	6.0	17.0	124.0
2008–2012	76.2	37.0	108.1	15.4	56.5	116.2	27.6	12.7	90.6	5.4	17.5	117.1
	15–24			55–64			15–24			55–64		
1983–1987	5.2	17.0	121.5	7.2	36.3	85.4	1.8	6.3	92.9	4.4	18.2	92.3
1988–1992	3.4	10.1	102.4	9.8	38.9	109.4	2.4	6.6	124.7	3.4	13.9	78.1
1993–1997	4.2	12.0	105.8	10.2	39.7	98.7	1.8	5.4	102.6	4.0	14.2	89.3
1998–2002	6.2	18.4	116.5	20.0	73.1	115.1	2.4	7.5	107.0	5.2	17.6	93.5
2003–2007	4.2	15.9	91.2	21.8	69.6	120.7	1.2	6.1	67.6	5.4	16.3	97.2
2008–2012	4.4	19.4	91.7	16.2	49.4	101.6	2.2	9.6	98.5	3.8	12.4	83.0
	25–34			65–74			25–34			65–74		
1983–1987	7.8	26.7	104.6	4.4	38.9	89.0	4.4	13.0	120.4	3.8	24.3	77.6
1988–1992	5.4	20.3	106.2	4.8	34.4	97.6	1.8	7.2	82.7	3.0	17.2	65.9
1993–1997	7.8	26.3	134.7	6.8	36.6	114.7	2.8	9.0	108.5	3.8	16.9	86.9
1998–2002	9.0	29.7	110.1	11.2	51.4	112.9	4.4	13.1	113.1	3.8	16.1	76.8
2003–2007	9.6	32.6	105.0	7.8	36.4	84.9	3.8	12.6	97.0	6.4	21.6	116.2
2008–2012	9.8	36.6	112.5	7.8	34.8	88.1	4.4	15.6	109.1	4.0	15.3	85.9
	35–44			>74 y/o			35–44			>74 y/o		
1983–1987	12.4	37.4	117.5	3.8	57.4	73.2	4.4	12.4	104.9	4.0	36.7	63.6
1988–1992	9.8	28.1	118.7	3.2	41.9	64.9	2.8	8.6	89.1	4.0	27.6	57.7
1993–1997	11.0	33.9	138.3	5.8	57.4	110.2	3.6	10.3	120.9	4.8	26.2	76.1
1998–2002	11.8	41.5	117.8	5.6	47.8	90.7	2.0	8.1	78.4	4.2	19.6	63.2
2003–2007	15.8	55.3	138.1	6.2	41.0	90.9	2.4	9.9	78.6	5.4	19.4	86.6
2008–2012	13.4	44.1	118.6	9.2	47.2	113.8	3.2	11.8	87.2	4.4	14.2	73.8

Secondary Medical Zone ID: 287

	Male						Female					
	Suicide			Suicide			Suicide			Suicide		
	Num per year	Rate /100,000	×100	Num per year	Rate /100,000	×100	Num per year	Rate /100,000	×100	Num per year	Rate /100,000	×100
	Total (>10 y/o)			45–54			Total (>10 y/o)			45–54		
1983–1987	24.4	36.8	121.5	4.8	51.8	102.5	9.8	14.4	89.3	1.8	17.4	100.8
1988–1992	19.2	28.1	119.6	4.8	46.1	134.3	9.4	12.2	91.6	1.6	15.3	104.4
1993–1997	20.6	28.5	121.5	5.4	46.3	126.5	9.4	11.0	101.1	1.2	12.6	97.3
1998–2002	28.6	39.6	117.0	8.2	69.8	124.7	10.8	12.7	100.4	1.8	15.8	104.9
2003–2007	36.6	46.7	146.7	10.6	94.0	168.0	9.4	12.3	96.0	1.2	13.3	96.7
2008–2012	29.8	38.2	128.2	5.8	60.1	123.7	8.8	13.0	94.8	1.2	14.6	97.9
	15–24			55–64			15–24			55–64		
1983–1987	1.2	13.9	99.9	4.2	47.7	112.2	0.2	5.4	79.0	1.4	17.5	88.5
1988–1992	1.2	11.0	111.2	4.0	40.6	114.3	0.2	4.3	82.3	1.2	15.3	85.7
1993–1997	1.2	12.0	105.8	4.8	48.4	120.1	0.6	5.6	107.6	2.0	17.7	111.4
1998–2002	2.4	20.3	128.2	5.8	66.8	105.2	0.4	6.4	90.8	2.2	20.3	107.8
2003–2007	2.0	20.3	116.6	6.6	65.7	113.7	0.2	6.9	76.5	2.4	19.2	114.4
2008–2012	1.0	19.2	90.4	6.6	56.4	116.2	1.0	11.5	118.7	1.6	15.0	100.3
	25–34			65–74			25–34			65–74		
1983–1987	4.6	38.3	149.8	1.6	38.9	88.9	1.2	11.5	107.2	1.8	29.0	92.8
1988–1992	3.6	31.7	166.1	1.6	31.9	90.5	1.4	11.2	128.6	1.2	20.0	76.6
1993–1997	3.0	28.2	144.4	1.8	28.5	89.4	0.8	8.8	105.2	1.0	15.0	76.9
1998–2002	2.8	30.7	113.8	2.6	38.9	85.6	1.0	11.7	101.0	2.0	21.0	100.0
2003–2007	4.8	45.1	145.3	4.2	49.6	115.7	0.8	12.0	92.6	2.2	20.6	110.7
2008–2012	2.0	31.0	95.1	4.8	51.5	130.3	0.8	13.4	93.2	1.2	16.2	90.8
	35–44			>74 y/o			35–44			>74 y/o		
1983–1987	5.4	45.4	142.6	2.6	78.7	100.5	1.0	11.2	94.9	2.4	50.0	86.7
1988–1992	3.0	26.5	112.0	1.0	41.4	64.2	1.4	10.9	112.9	2.4	39.4	82.3
1993–1997	2.8	27.1	110.5	1.6	44.5	85.5	1.2	9.9	116.7	2.6	34.3	99.6
1998–2002	4.2	43.2	122.6	2.6	53.3	101.1	0.8	9.9	96.2	2.4	27.4	88.2
2003–2007	4.4	50.2	125.2	4.0	57.7	128.1	1.0	12.4	98.5	1.6	16.7	74.3
2008–2012	3.6	40.7	109.4	6.0	66.4	159.9	1.6	15.2	112.4	1.4	14.0	72.8

Secondary Medical Zone ID: 288

	Male						Female					
	Suicide			Suicide			Suicide			Suicide		
	Num per year	Rate /100,000	×100	Num per year	Rate /100,000	×100	Num per year	Rate /100,000	×100	Num per year	Rate /100,000	×100
	Total (>10 y/o)			45–54			Total (>10 y/o)			45–54		
1983–1987	40.0	34.2	104.1	9.0	50.0	98.9	15.4	12.4	71.1	2.8	14.9	86.3
1988–1992	31.8	26.8	105.2	8.2	43.8	127.6	18.2	11.9	87.0	3.6	16.5	112.8
1993–1997	36.6	28.7	116.3	9.2	44.6	122.0	15.8	10.2	88.2	2.6	12.9	99.6
1998–2002	49.4	39.0	111.9	15.6	74.6	133.3	17.0	12.0	85.5	4.2	17.9	119.0
2003–2007	49.6	41.2	116.4	11.8	68.3	122.1	14.8	11.3	83.9	3.2	15.9	116.2
2008–2012	42.0	37.2	110.4	9.8	63.6	130.8	14.4	11.9	87.5	1.8	13.6	91.4
	15–24			55–64			15–24			55–64		
1983–1987	5.0	23.7	170.2	4.6	32.5	76.5	0.2	4.1	60.0	2.2	13.9	70.3
1988–1992	1.8	10.4	105.2	3.8	25.6	72.0	0.4	3.9	73.2	3.4	16.7	93.7
1993–1997	2.4	13.0	115.0	8.6	48.1	119.4	0.8	4.9	94.7	2.6	14.0	88.2
1998–2002	2.0	14.8	93.6	10.8	65.8	103.6	1.2	7.3	104.5	3.2	17.8	94.6
2003–2007	2.8	19.6	112.1	10.6	58.9	102.1	0.2	5.5	61.0	3.0	16.0	95.7
2008–2012	1.6	18.5	87.3	12.4	60.1	123.7	0.8	8.7	89.5	1.0	10.1	67.6
	25–34			65–74			25–34			65–74		
1983–1987	5.0	27.4	107.1	4.4	43.5	99.3	2.8	13.0	120.5	2.0	19.0	60.8
1988–1992	6.0	33.4	174.5	3.4	31.3	88.7	1.6	9.2	106.6	3.0	20.1	77.0
1993–1997	3.0	21.4	109.7	3.8	29.3	91.9	1.4	8.8	105.8	2.4	14.6	75.0
1998–2002	5.2	33.9	125.7	5.8	41.9	92.1	0.6	8.5	73.4	3.0	17.2	81.9
2003–2007	4.6	34.6	111.7	7.4	49.2	114.6	0.8	10.0	76.7	3.0	17.0	91.2
2008–2012	2.4	27.4	84.2	5.4	38.8	98.3	2.0	15.6	108.8	2.4	15.6	87.9
	35–44			>74 y/o			35–44			>74 y/o		
1983–1987	8.8	41.0	129.0	3.0	56.2	71.8	3.0	13.1	111.2	2.4	30.0	52.0
1988–1992	6.4	29.6	124.9	1.8	33.5	51.9	2.8	11.5	119.1	3.4	29.7	62.1
1993–1997	7.0	35.9	146.5	2.6	35.8	68.7	2.4	10.9	128.7	3.6	25.3	73.4
1998–2002	5.4	36.9	104.8	4.4	47.1	89.3	1.8	11.0	106.3	3.0	18.5	59.5
2003–2007	7.2	51.9	129.6	5.2	45.2	100.3	1.4	11.6	91.5	3.2	15.8	70.5
2008–2012	6.4	44.6	119.9	4.0	33.7	81.3	1.2	11.9	87.5	5.2	20.7	107.4

Secondary Medical Zone ID: 289

	Male						Female					
	Suicide			Suicide			Suicide			Suicide		
	Num per year	Rate /100,000	×100	Num per year	Rate /100,000	×100	Num per year	Rate /100,000	×100	Num per year	Rate /100,000	×100
	Total (>10 y/o)			45–54			Total (>10 y/o)			45–54		
1983–1987	38.0	40.2	138.7	8.6	65.7	130.0	14.8	14.6	92.6	2.4	17.1	99.2
1988–1992	31.2	30.8	139.7	6.8	49.1	143.0	14.8	12.7	97.9	2.8	16.9	115.8
1993–1997	30.4	29.7	129.3	6.4	41.8	114.1	11.4	10.3	89.0	2.8	15.3	118.1
1998–2002	53.0	47.8	153.4	14.8	84.0	150.0	17.0	13.2	108.5	2.8	16.0	106.3
2003–2007	48.4	47.2	143.9	14.4	97.6	174.6	16.0	13.7	109.1	3.6	18.8	137.3
2008–2012	44.0	41.5	139.5	7.8	65.5	134.8	14.0	12.5	102.0	1.6	14.7	98.6
	15–24			55–64			15–24			55–64		
1983–1987	1.2	12.4	89.1	9.6	71.9	169.2	1.0	7.5	110.5	1.8	15.7	79.7
1988–1992	1.0	8.9	89.6	8.0	56.7	159.8	0.4	4.6	86.3	2.4	17.2	96.2
1993–1997	0.6	8.1	71.3	6.0	47.9	118.9	0.6	5.0	95.2	2.6	17.3	108.7
1998–2002	2.4	16.2	102.6	12.0	92.1	145.0	1.0	7.4	105.9	3.0	20.1	106.9
2003–2007	2.4	18.2	104.5	10.2	70.2	121.6	1.2	10.0	111.3	3.6	19.7	117.9
2008–2012	1.2	16.8	79.3	11.4	64.2	132.2	0.8	9.5	97.7	1.8	13.1	88.0
	25–34			65–74			25–34			65–74		
1983–1987	4.8	31.8	124.6	3.6	48.3	110.4	2.2	13.0	120.4	2.8	28.6	91.4
1988–1992	2.8	23.0	120.4	2.6	34.2	97.2	1.6	10.6	122.1	3.4	27.9	107.0
1993–1997	2.4	21.7	111.2	4.4	42.4	132.8	0.6	7.4	88.8	1.6	14.7	75.6
1998–2002	4.4	33.6	124.6	7.6	64.1	140.9	0.8	10.1	86.8	2.8	20.8	99.2
2003–2007	3.4	30.5	98.5	7.2	61.5	143.3	1.4	12.7	98.0	2.4	18.4	98.6
2008–2012	3.2	31.7	97.3	5.8	50.1	126.8	0.8	11.9	82.7	3.2	21.0	118.0
	35–44			>74 y/o			35–44			>74 y/o		
1983–1987	5.8	37.2	116.8	4.4	102.5	130.8	1.8	11.7	99.2	2.8	47.2	81.8
1988–1992	5.4	30.5	128.8	4.6	84.3	130.7	1.4	9.2	95.0	2.8	34.9	72.9
1993–1997	6.2	38.6	157.3	4.4	71.8	137.8	1.4	9.1	106.6	1.8	20.0	58.2
1998–2002	7.0	54.2	153.9	4.8	68.5	129.9	1.6	11.8	114.0	5.0	37.3	120.0
2003–2007	6.0	55.6	138.7	4.4	53.4	118.6	1.4	13.1	103.5	2.2	16.0	71.2
2008–2012	7.8	55.8	150.1	6.8	64.4	155.2	1.4	13.5	99.6	4.4	23.5	122.4

Secondary Medical Zone ID: 290

	Male						Female					
	Suicide			Suicide			Suicide			Suicide		
	Num per year	Rate /100,000	×100	Num per year	Rate /100,000	×100	Num per year	Rate /100,000	×100	Num per year	Rate /100,000	×100
	Total (>10 y/o)			45-54			Total (>10 y/o)			45-54		
1983-1987	25.4	40.7	141.7	7.4	76.1	150.6	10.2	15.0	100.8	1.6	17.2	99.6
1988-1992	23.4	32.4	159.6	5.8	55.2	160.6	9.8	12.8	103.0	1.8	16.3	111.4
1993-1997	19.2	29.5	129.2	3.8	39.6	108.1	6.8	10.0	88.2	0.8	11.4	87.9
1998-2002	27.8	41.4	131.0	9.2	82.1	146.7	10.8	13.1	109.3	2.4	18.2	120.9
2003-2007	26.0	42.4	127.0	7.6	84.3	150.8	8.8	12.7	100.6	1.6	15.4	112.3
2008-2012	23.6	37.7	122.6	3.8	55.1	113.4	6.6	12.5	89.1	1.6	17.6	118.2
	15-24			55-64			15-24			55-64		
1983-1987	1.8	18.8	134.5	4.2	50.8	119.6	0.0	5.0	73.7	3.2	26.8	136.0
1988-1992	1.6	12.9	130.4	5.4	54.1	152.3	0.0	3.9	73.3	2.0	19.4	108.5
1993-1997	1.0	11.6	102.9	4.6	50.6	125.8	0.2	4.5	86.6	1.6	16.6	104.5
1998-2002	2.0	19.7	124.9	5.6	69.5	109.3	0.0	5.3	75.8	2.4	21.6	114.8
2003-2007	1.0	17.3	98.9	5.4	60.6	105.0	0.0	6.5	71.7	1.8	17.4	104.1
2008-2012	0.4	17.5	82.7	5.6	54.6	112.4	0.4	9.3	95.9	1.2	14.0	93.6
	25-34			65-74			25-34			65-74		
1983-1987	3.0	31.3	122.4	2.0	45.6	104.1	1.2	11.9	110.6	1.6	28.5	91.3
1988-1992	1.8	23.2	121.2	2.8	45.5	129.3	0.4	7.9	90.6	2.2	28.7	110.2
1993-1997	2.4	27.4	140.4	1.4	27.2	85.4	0.4	7.8	93.8	1.6	19.5	100.2
1998-2002	1.2	24.3	90.1	4.4	56.2	123.6	0.8	11.8	101.5	1.2	17.6	83.7
2003-2007	3.4	39.8	128.3	3.6	49.2	114.8	1.0	13.7	105.3	2.2	21.6	115.8
2008-2012	2.8	36.6	112.5	3.4	44.6	112.8	0.2	11.4	79.9	1.4	17.3	97.5
	35-44			>74 y/o			35-44			>74 y/o		
1983-1987	4.8	43.0	135.0	2.0	83.0	106.0	1.0	11.3	95.4	1.6	47.0	81.5
1988-1992	4.2	34.8	147.0	1.8	63.0	97.7	1.0	9.8	101.5	2.4	45.7	95.5
1993-1997	4.0	38.3	156.4	1.8	53.5	102.7	0.6	8.0	94.6	1.6	27.1	78.9
1998-2002	2.8	40.0	113.5	2.6	60.2	114.1	1.0	11.6	112.2	3.0	35.4	114.1
2003-2007	3.0	47.2	117.7	2.0	42.3	94.0	1.0	13.6	107.5	1.2	15.3	68.0
2008-2012	3.6	44.5	119.5	4.0	56.5	136.1	1.0	14.1	104.2	0.8	11.8	61.5

Secondary Medical Zone ID: 291

	Male						Female					
	Suicide			Suicide			Suicide			Suicide		
	Num per year	Rate /100,000	×100	Num per year	Rate /100,000	×100	Num per year	Rate /100,000	×100	Num per year	Rate /100,000	×100
	Total (>10 y/o)			45–54			Total (>10 y/o)			45–54		
1983–1987	29.4	39.2	134.2	6.4	60.8	120.3	13.6	15.5	103.7	2.6	19.6	114.0
1988–1992	26.0	31.3	145.8	6.0	51.5	150.0	15.0	14.0	117.6	2.6	17.9	122.3
1993–1997	24.6	29.8	135.1	6.8	51.1	139.7	10.0	10.8	96.7	1.4	12.5	97.1
1998–2002	29.2	38.7	117.3	8.4	68.1	121.7	12.8	13.3	105.8	3.0	18.8	125.1
2003–2007	33.0	44.6	135.0	5.4	57.6	103.0	11.6	13.1	105.6	1.4	13.7	100.3
2008–2012	30.6	40.9	134.7	5.6	64.3	132.4	11.8	13.6	109.7	1.6	16.7	112.1
	15–24			55–64			15–24			55–64		
1983–1987	1.6	16.5	118.1	4.0	44.6	104.8	0.8	7.5	110.0	2.2	19.3	97.6
1988–1992	0.2	7.7	77.3	5.4	48.1	135.5	0.4	5.0	94.7	2.4	19.1	106.7
1993–1997	0.8	10.5	92.6	4.2	43.2	107.3	0.6	5.6	106.7	1.2	13.4	84.1
1998–2002	1.6	16.9	107.1	4.2	53.3	83.9	0.4	6.4	90.8	1.6	17.3	91.7
2003–2007	2.2	21.8	124.9	7.8	69.7	120.7	0.6	8.7	97.0	3.0	20.2	120.5
2008–2012	2.4	27.6	130.4	6.8	55.1	113.4	0.6	9.7	99.6	2.8	17.8	119.6
	25–34			65–74			25–34			65–74		
1983–1987	4.2	34.1	133.5	2.8	46.2	105.6	1.8	13.2	122.5	2.6	30.8	98.4
1988–1992	1.8	21.2	111.1	3.2	43.3	122.9	0.8	8.9	102.3	3.2	30.4	116.4
1993–1997	1.6	21.2	108.3	3.0	36.8	115.3	0.8	8.9	107.5	2.4	21.3	109.4
1998–2002	2.0	27.4	101.5	7.0	68.2	150.0	1.4	13.4	115.8	2.6	22.6	107.6
2003–2007	5.6	50.9	164.3	4.2	48.3	112.5	1.0	13.1	100.9	2.8	22.3	120.0
2008–2012	3.4	39.0	119.8	6.0	57.6	145.8	0.2	11.2	78.0	1.6	17.1	95.9
	35–44			>74 y/o			35–44			>74 y/o		
1983–1987	6.0	45.1	141.7	4.2	110.8	141.5	2.0	13.7	116.1	1.6	38.9	67.4
1988–1992	5.6	37.9	159.8	3.8	81.1	125.7	2.2	12.6	130.7	3.4	47.1	98.5
1993–1997	3.8	33.0	134.7	4.4	80.9	155.3	1.6	11.1	130.0	2.0	25.1	72.8
1998–2002	3.2	39.9	113.4	2.8	53.5	101.5	1.2	11.7	113.6	2.6	25.8	83.0
2003–2007	4.6	58.3	145.5	3.2	49.2	109.3	1.2	13.8	109.4	1.6	15.1	67.4
2008–2012	3.2	40.8	109.7	3.2	43.7	105.4	2.2	17.9	131.8	2.8	19.7	102.5

Secondary Medical Zone ID: 292

	Male						Female					
	Suicide			Suicide			Suicide			Suicide		
	Num per year	Rate /100,000	×100	Num per year	Rate /100,000	×100	Num per year	Rate /100,000	×100	Num per year	Rate /100,000	×100
	Total (>10 y/o)			45–54			Total (>10 y/o)			45–54		
1983–1987	168.2	35.6	111.3	47.6	61.3	121.2	70.6	13.2	85.7	13.8	16.1	93.3
1988–1992	140.0	28.0	116.2	30.0	37.7	109.7	65.8	11.3	85.3	11.4	13.3	90.8
1993–1997	151.6	28.7	116.5	40.6	45.9	125.5	58.8	9.6	84.2	10.2	11.1	86.2
1998–2002	217.8	39.7	113.2	62.0	71.9	128.4	76.4	12.2	91.0	13.2	14.5	96.3
2003–2007	216.6	40.9	113.6	46.8	64.8	115.9	73.8	12.5	92.0	11.8	15.0	109.4
2008–2012	185.2	36.0	106.9	32.0	51.6	106.2	79.2	13.9	101.0	12.2	17.6	118.0
	15–24			55–64			15–24			55–64		
1983–1987	11.8	15.3	109.5	31.0	51.4	121.0	4.0	5.4	78.6	12.2	17.9	90.7
1988–1992	9.4	11.2	112.7	32.0	45.9	129.4	3.0	3.9	74.7	10.8	14.4	80.5
1993–1997	9.6	11.5	102.1	36.8	50.9	126.3	5.0	5.9	114.0	10.4	13.0	82.0
1998–2002	9.2	13.2	83.6	48.2	64.5	101.5	5.0	7.0	100.1	17.2	20.0	106.1
2003–2007	9.2	15.4	88.0	59.8	72.4	125.3	6.0	9.7	108.3	15.2	16.6	99.0
2008–2012	10.2	19.6	92.5	42.0	51.2	105.5	5.6	10.7	110.0	14.2	15.6	104.5
	25–34			65–74			25–34			65–74		
1983–1987	17.6	22.9	89.5	15.6	47.5	108.5	4.4	6.2	57.2	11.8	27.7	88.6
1988–1992	13.0	19.7	103.3	14.8	37.9	107.5	8.6	11.3	130.0	11.4	22.0	84.1
1993–1997	13.6	20.7	106.1	18.2	36.3	113.8	5.8	8.1	97.5	12.0	18.9	97.1
1998–2002	21.6	30.0	111.3	30.0	51.0	112.2	9.8	12.7	109.4	12.6	17.7	84.3
2003–2007	24.2	34.4	110.8	30.6	49.5	115.3	9.2	12.7	97.6	11.6	15.5	83.3
2008–2012	19.4	32.1	98.7	30.4	46.5	117.6	12.2	18.5	128.8	12.4	16.1	90.5
	35–44			>74 y/o			35–44			>74 y/o		
1983–1987	32.8	35.0	110.1	11.6	75.2	96.0	11.0	11.1	93.9	13.2	52.6	91.2
1988–1992	26.2	29.3	123.8	14.2	67.2	104.1	7.8	8.6	88.7	12.6	35.6	74.5
1993–1997	22.4	29.5	120.4	10.0	40.8	78.3	6.8	8.6	101.4	8.6	19.6	57.0
1998–2002	25.8	39.1	111.0	20.8	64.1	121.6	8.0	11.2	108.2	10.4	18.5	59.4
2003–2007	29.8	45.9	114.5	16.0	39.3	87.3	10.4	14.4	113.8	9.4	13.3	59.4
2008–2012	29.0	41.2	110.6	22.0	44.3	106.8	9.0	12.5	92.3	13.6	15.8	82.0

Secondary Medical Zone ID: 293

	Male						Female					
	Suicide			Suicide			Suicide			Suicide		
	Num per year	Rate /100,000	×100	Num per year	Rate /100,000	×100	Num per year	Rate /100,000	×100	Num per year	Rate /100,000	×100
	Total (>10 y/o)			45–54			Total (>10 y/o)			45–54		
1983–1987	27.4	34.2	107.9	7.4	57.5	113.7	11.6	13.6	84.5	2.6	18.2	105.9
1988–1992	25.0	27.8	119.0	6.0	43.0	125.1	13.2	12.4	96.4	2.0	14.6	99.6
1993–1997	22.2	25.5	100.9	6.2	40.8	111.5	11.8	10.7	96.8	2.0	13.1	101.2
1998–2002	35.4	37.1	107.6	10.6	67.6	120.8	14.0	12.7	97.4	2.2	14.8	98.2
2003–2007	40.0	42.3	120.9	10.8	78.4	140.1	11.2	11.8	87.4	2.0	14.3	104.1
2008–2012	32.4	35.8	106.6	6.0	53.1	109.3	13.4	12.8	100.6	2.0	16.0	107.5
	15–24			55–64			15–24			55–64		
1983–1987	3.0	20.4	146.1	3.6	38.7	91.1	0.8	6.7	98.7	1.6	16.5	83.4
1988–1992	1.0	8.9	89.7	5.4	43.1	121.4	0.2	3.9	73.6	2.6	18.9	105.7
1993–1997	1.0	9.7	85.6	4.4	38.3	95.2	1.2	6.8	131.0	2.6	17.7	111.2
1998–2002	1.4	13.9	88.3	5.8	51.9	81.6	0.8	7.1	101.0	2.2	17.5	92.7
2003–2007	3.6	24.6	141.0	8.2	58.6	101.5	0.4	7.1	79.2	3.4	19.2	114.6
2008–2012	2.4	24.0	113.1	4.8	39.1	80.5	1.0	10.7	110.0	3.2	17.3	116.1
	25–34			65–74			25–34			65–74		
1983–1987	4.4	31.4	122.7	1.8	36.2	82.7	0.8	8.6	79.8	2.0	26.5	84.8
1988–1992	3.6	27.8	145.4	3.2	41.4	117.5	0.8	8.1	93.0	1.8	21.0	80.6
1993–1997	3.0	24.4	125.1	2.0	26.5	83.0	0.6	7.3	87.9	2.2	19.5	99.9
1998–2002	4.6	33.7	125.0	5.0	48.4	106.4	2.2	14.3	123.3	2.4	20.0	95.4
2003–2007	4.8	37.4	120.7	4.8	44.6	104.0	1.0	11.4	87.4	2.2	17.8	95.7
2008–2012	4.4	36.9	113.4	5.4	44.8	113.4	1.0	12.8	89.2	3.2	20.8	116.6
	35–44			>74 y/o			35–44			>74 y/o		
1983–1987	4.6	31.8	100.1	2.4	72.6	92.7	2.2	13.0	110.0	1.4	33.2	57.5
1988–1992	3.8	25.3	106.6	2.0	52.8	81.8	2.0	11.1	115.1	3.8	48.3	101.0
1993–1997	2.8	23.0	93.7	2.2	47.3	90.7	1.0	8.1	95.1	2.2	25.3	73.5
1998–2002	3.8	35.0	99.2	4.0	62.3	118.1	1.2	10.3	99.8	2.8	25.5	82.2
2003–2007	4.6	42.9	107.0	3.2	43.9	97.5	1.2	12.0	94.8	1.0	10.6	47.1
2008–2012	4.4	37.5	100.9	5.0	51.5	124.1	0.8	11.2	82.3	2.2	15.4	79.8

Secondary Medical Zone ID: 294

	Male						Female					
	Suicide			Suicide			Suicide			Suicide		
	Num per year	Rate /100,000	×100	Num per year	Rate /100,000	×100	Num per year	Rate /100,000	×100	Num per year	Rate /100,000	×100
	Total (>10 y/o)			45–54			Total (>10 y/o)			45–54		
1983–1987	49.2	35.0	109.4	13.2	60.5	119.6	23.8	14.4	91.5	5.2	20.3	117.6
1988–1992	36.2	24.5	99.9	9.0	41.1	119.7	16.8	10.5	72.5	2.0	11.4	77.8
1993–1997	35.8	23.8	92.7	9.8	40.1	109.5	19.2	10.1	89.3	3.8	14.1	109.5
1998–2002	62.4	38.7	107.8	19.8	72.5	129.6	19.6	10.9	80.0	3.0	12.9	85.4
2003–2007	66.0	40.8	111.7	18.2	73.4	131.2	19.0	10.9	81.3	3.4	13.6	99.5
2008–2012	52.8	33.4	97.5	10.2	48.6	100.1	19.6	12.1	85.2	3.6	15.5	103.7
	15–24			55–64			15–24			55–64		
1983–1987	3.4	14.7	105.1	8.0	43.9	103.2	1.6	6.6	96.4	4.2	19.6	99.5
1988–1992	2.2	9.4	95.1	8.2	39.3	110.8	1.0	4.5	85.4	4.0	17.4	97.7
1993–1997	3.6	12.9	114.0	7.4	37.9	94.2	1.8	6.2	118.1	2.0	11.2	70.5
1998–2002	5.0	18.9	119.7	12.6	63.5	100.0	0.8	5.1	72.7	4.6	19.6	104.3
2003–2007	4.0	18.1	103.5	11.4	51.9	89.9	1.4	7.6	84.1	3.6	15.5	92.6
2008–2012	3.2	18.8	88.7	12.2	48.4	99.6	2.6	12.3	126.5	3.2	13.3	89.2
	25–34			65–74			25–34			65–74		
1983–1987	7.0	28.9	112.9	4.4	42.6	97.3	3.6	13.1	121.3	3.4	25.1	80.2
1988–1992	2.8	14.9	78.2	3.4	29.7	84.4	1.2	6.7	77.7	3.8	22.9	88.0
1993–1997	3.4	17.5	89.5	2.0	17.8	55.9	1.4	7.2	86.1	2.6	14.7	75.2
1998–2002	4.2	22.2	82.2	7.4	43.7	96.0	1.8	9.6	82.7	4.0	19.2	91.3
2003–2007	8.6	37.3	120.2	8.6	47.8	111.5	2.0	10.7	82.4	3.2	16.2	86.8
2008–2012	5.4	29.4	90.3	7.0	40.4	102.3	1.6	10.8	75.6	3.0	15.7	88.4
	35–44			>74 y/o			35–44			>74 y/o		
1983–1987	8.8	35.7	112.1	4.4	71.3	91.0	2.6	10.9	92.2	3.2	34.7	60.2
1988–1992	6.8	25.6	108.0	3.6	51.3	79.6	2.8	10.1	104.6	2.0	18.9	39.4
1993–1997	5.4	22.8	92.9	4.2	49.5	95.0	2.6	9.6	112.8	5.0	30.9	89.7
1998–2002	10.6	45.5	129.2	2.8	33.1	62.7	1.6	8.4	80.9	3.8	20.7	66.5
2003–2007	10.8	49.5	123.6	4.4	36.0	80.0	1.6	9.6	76.1	3.8	16.7	74.3
2008–2012	8.2	38.0	102.0	6.4	41.0	98.9	2.6	12.5	92.0	3.0	12.4	64.4

Secondary Medical Zone ID: 295

	Male						Female					
	Suicide			Suicide			Suicide			Suicide		
	Num per year	Rate /100,000	×100	Num per year	Rate /100,000	×100	Num per year	Rate /100,000	×100	Num per year	Rate /100,000	×100
	Total (>10 y/o)			45–54			Total (>10 y/o)			45–54		
1983–1987	16.0	35.6	118.6	5.2	68.9	136.3	3.8	13.0	70.0	0.4	13.6	78.7
1988–1992	12.6	26.2	113.0	2.6	36.2	105.5	4.6	11.2	76.7	0.2	11.4	77.7
1993–1997	12.0	25.1	99.1	1.8	29.7	81.2	6.4	10.6	97.7	1.0	12.6	97.4
1998–2002	18.2	35.1	97.3	5.4	59.0	105.4	7.2	12.3	93.1	1.6	15.8	105.2
2003–2007	19.8	37.0	100.0	5.0	60.5	108.1	8.0	12.1	99.1	1.0	13.1	95.9
2008–2012	19.6	34.5	103.5	2.6	43.1	88.7	5.6	12.2	83.3	0.6	12.7	85.3
	15–24			55–64			15–24			55–64		
1983–1987	0.8	13.4	95.8	2.0	41.7	98.0	0.0	5.1	74.5	1.0	19.0	96.2
1988–1992	1.2	11.6	117.0	1.6	31.5	88.7	0.0	3.8	72.2	1.2	18.1	101.2
1993–1997	0.2	8.7	77.3	3.4	46.8	116.2	0.6	5.7	109.1	1.2	16.4	103.1
1998–2002	0.6	13.1	83.1	4.4	63.6	100.1	0.4	6.5	91.9	1.2	18.0	95.4
2003–2007	1.6	19.4	111.1	2.8	43.5	75.4	0.0	6.1	67.8	2.6	20.8	124.4
2008–2012	0.4	16.8	79.1	5.2	53.4	109.9	0.8	10.8	110.9	0.4	11.6	78.1
	25–34			65–74			25–34			65–74		
1983–1987	2.6	31.2	122.2	0.6	35.7	81.5	0.2	8.1	75.6	0.6	24.6	78.9
1988–1992	1.0	18.2	95.0	1.4	38.0	107.8	0.4	8.0	92.3	0.8	22.0	84.2
1993–1997	1.6	21.9	112.0	1.2	30.0	94.2	0.4	7.7	92.7	0.2	12.5	64.1
1998–2002	2.0	27.1	100.3	3.2	52.6	115.6	0.2	9.1	78.7	1.4	21.5	102.6
2003–2007	3.4	36.5	117.7	2.6	44.5	103.7	0.2	9.6	73.8	1.4	19.4	104.1
2008–2012	2.8	35.0	107.6	3.6	47.5	120.2	0.8	13.2	91.9	0.4	13.9	78.2
	35–44			>74 y/o			35–44			>74 y/o		
1983–1987	3.6	38.8	121.8	1.0	70.0	89.3	0.4	9.7	82.4	1.2	49.8	86.3
1988–1992	3.4	32.4	136.8	1.2	61.5	95.4	0.6	8.9	92.5	1.4	39.9	83.4
1993–1997	2.2	26.7	109.0	1.4	55.3	106.3	1.0	9.8	114.9	1.8	36.7	106.8
1998–2002	2.0	32.1	91.1	0.6	37.5	71.2	1.0	11.4	109.9	1.4	26.6	85.6
2003–2007	2.6	38.6	96.4	1.8	45.8	101.7	1.0	12.9	102.4	1.8	24.0	107.1
2008–2012	3.6	40.5	108.9	1.2	33.4	80.4	1.6	15.2	112.4	1.0	15.1	78.5

Secondary Medical Zone ID: 296

	Male						Female					
	Suicide			Suicide			Suicide			Suicide		
	Num per year	Rate /100,000	×100	Num per year	Rate /100,000	×100	Num per year	Rate /100,000	×100	Num per year	Rate /100,000	×100
	Total (>10 y/o)			45–54			Total (>10 y/o)			45–54		
1983–1987	23.6	36.3	119.5	6.2	62.7	124.0	12.6	15.8	106.4	1.2	15.0	87.3
1988–1992	17.8	26.9	113.8	4.0	41.8	121.6	7.8	11.4	80.5	1.4	14.7	100.6
1993–1997	18.4	26.6	112.4	4.2	40.1	109.6	8.6	10.5	95.6	1.6	14.0	108.4
1998–2002	31.6	42.6	131.0	10.4	82.0	146.6	9.0	12.1	90.3	1.8	15.7	104.2
2003–2007	34.0	46.7	141.8	10.4	92.3	165.1	7.2	11.3	83.5	1.0	12.4	90.2
2008–2012	31.6	41.7	139.0	4.8	54.6	112.4	10.4	13.5	105.6	1.6	16.1	107.9
	15–24			55–64			15–24			55–64		
1983–1987	1.4	15.5	110.9	4.0	46.3	109.1	1.2	8.8	129.3	1.8	19.0	96.4
1988–1992	1.0	10.6	106.6	4.2	42.6	119.9	0.4	5.1	96.0	1.0	14.4	80.4
1993–1997	0.8	10.7	94.6	4.6	48.2	119.8	0.4	5.0	96.7	1.8	17.0	106.9
1998–2002	2.2	19.8	125.3	5.2	65.3	102.7	0.2	5.8	82.9	2.4	21.4	113.7
2003–2007	1.8	20.1	115.4	6.6	67.3	116.6	0.4	8.0	88.9	1.8	17.3	103.3
2008–2012	1.2	20.9	98.5	7.2	59.3	122.0	0.6	9.9	101.8	1.6	14.9	100.1
	25–34			65–74			25–34			65–74		
1983–1987	4.4	35.3	138.3	1.6	38.9	89.0	1.4	11.7	108.8	1.6	26.5	84.6
1988–1992	2.8	26.3	137.4	1.4	30.2	85.7	0.8	8.6	99.8	0.6	14.9	57.1
1993–1997	2.0	22.5	115.4	1.8	28.9	90.5	0.8	8.7	104.8	1.4	17.2	88.0
1998–2002	2.8	31.1	115.0	4.8	55.3	121.6	0.8	11.1	96.1	1.2	16.7	79.4
2003–2007	3.6	38.0	122.7	3.0	42.0	97.9	0.2	9.7	74.8	0.6	13.3	71.2
2008–2012	4.4	43.7	134.2	4.2	49.7	125.9	1.6	16.7	116.3	1.8	18.9	106.3
	35–44			>74 y/o			35–44			>74 y/o		
1983–1987	3.6	34.3	107.8	2.0	74.1	94.6	2.4	16.0	135.2	2.8	59.7	103.5
1988–1992	3.4	27.7	116.7	1.0	43.1	66.9	0.8	8.9	91.8	2.8	45.8	95.7
1993–1997	2.4	24.0	98.0	2.4	59.3	113.8	0.8	8.2	96.7	1.8	27.0	78.4
1998–2002	4.2	43.6	123.8	2.0	47.8	90.7	0.8	9.8	95.2	1.8	23.0	74.2
2003–2007	5.2	58.8	146.8	3.4	53.7	119.3	0.8	11.9	93.9	2.4	22.4	99.7
2008–2012	6.0	54.7	147.0	3.8	49.7	119.8	1.0	13.4	98.9	2.2	18.5	96.0

Secondary Medical Zone ID: 297

	Male						Female					
	Suicide			Suicide			Suicide			Suicide		
	Num per year	Rate /100,000	×100	Num per year	Rate /100,000	×100	Num per year	Rate /100,000	×100	Num per year	Rate /100,000	×100
	Total (>10 y/o)			45–54			Total (>10 y/o)			45–54		
1983–1987	13.2	35.2	114.8	4.0	64.0	126.6	4.8	13.9	83.2	0.8	16.3	94.5
1988–1992	11.2	26.8	119.7	2.6	42.1	122.7	4.2	11.5	80.6	0.4	13.0	89.1
1993–1997	13.6	28.8	132.4	3.8	48.4	132.3	5.8	11.2	105.5	1.0	13.9	107.8
1998–2002	17.6	40.2	122.4	5.0	67.6	120.8	5.2	12.3	92.2	1.0	15.3	101.5
2003–2007	19.2	43.2	133.0	6.2	85.6	153.1	4.8	12.1	92.6	1.4	16.1	117.8
2008–2012	14.4	35.7	110.6	3.2	55.7	114.5	4.8	12.2	94.8	0.6	14.3	95.5
	15–24			55–64			15–24			55–64		
1983–1987	0.2	11.2	80.4	3.0	51.8	121.9	0.2	6.4	94.0	1.0	18.9	95.9
1988–1992	0.4	9.8	99.1	3.8	53.1	149.7	0.2	5.1	97.2	0.4	14.5	81.2
1993–1997	0.6	11.7	103.1	1.8	38.8	96.5	0.2	5.1	97.5	1.6	19.8	124.8
1998–2002	1.0	17.4	110.3	3.2	65.6	103.3	0.2	6.6	94.6	0.8	17.9	95.0
2003–2007	0.6	17.1	98.1	3.6	62.6	108.4	0.2	8.3	91.7	1.0	16.9	101.0
2008–2012	0.0	17.0	80.1	3.4	51.1	105.2	0.0	7.9	81.2	1.0	15.1	101.5
	25–34			65–74			25–34			65–74		
1983–1987	1.8	27.5	107.8	1.6	49.4	112.8	0.4	9.6	88.8	1.0	28.5	91.2
1988–1992	0.4	15.1	79.1	0.8	30.8	87.5	0.4	8.5	97.8	1.2	25.8	99.1
1993–1997	1.2	22.1	113.3	2.4	44.0	138.1	1.0	10.9	130.9	0.8	17.5	90.0
1998–2002	1.8	31.1	115.3	2.0	45.6	100.3	0.6	12.0	103.5	1.2	21.3	101.4
2003–2007	3.2	44.3	142.9	2.2	46.4	108.1	0.2	11.3	87.2	0.6	16.3	87.8
2008–2012	2.2	37.5	115.1	1.8	41.0	103.9	0.4	13.4	93.5	1.2	19.3	108.4
	35–44			>74 y/o			35–44			>74 y/o		
1983–1987	1.8	31.7	99.6	0.8	65.3	83.4	0.6	11.4	96.6	0.8	39.6	68.6
1988–1992	2.0	27.0	113.9	1.2	63.1	97.8	0.6	9.6	99.6	1.0	34.6	72.3
1993–1997	3.0	36.1	147.4	0.8	44.5	85.4	0.6	8.9	104.7	0.6	21.2	61.7
1998–2002	3.6	51.2	145.2	1.0	45.7	86.7	0.6	10.6	103.0	0.8	21.0	67.5
2003–2007	2.2	45.1	112.6	1.2	40.7	90.4	0.2	11.0	87.1	1.2	20.2	90.1
2008–2012	1.4	36.0	96.7	2.4	49.9	120.3	0.2	12.0	88.4	1.4	19.2	99.8

Secondary Medical Zone ID: 298

	Male						Female					
	Suicide			Suicide			Suicide			Suicide		
	Num per year	Rate /100,000	×100	Num per year	Rate /100,000	×100	Num per year	Rate /100,000	×100	Num per year	Rate /100,000	×100
	Total (>10 y/o)			45–54			Total (>10 y/o)			45–54		
1983–1987	25.8	34.7	106.6	5.6	50.9	100.7	11.0	13.2	79.8	1.6	15.2	88.0
1988–1992	19.6	25.3	102.3	4.2	38.7	112.6	14.4	13.1	103.7	2.8	18.3	124.9
1993–1997	23.2	27.4	114.3	3.8	34.8	95.2	12.2	10.8	102.6	2.8	17.0	131.5
1998–2002	37.4	42.6	127.0	10.0	70.7	126.3	10.8	12.0	86.3	1.6	14.1	93.5
2003–2007	35.8	41.8	123.9	9.4	72.9	130.4	9.6	11.8	85.4	1.2	12.2	89.2
2008–2012	26.6	35.9	103.1	7.0	60.7	125.0	8.2	11.1	82.1	1.2	13.5	90.5
	15–24			55–64			15–24			55–64		
1983–1987	0.6	9.9	71.0	3.4	35.9	84.4	0.6	6.4	93.1	2.2	18.2	92.1
1988–1992	0.6	8.8	88.6	3.6	33.2	93.4	0.2	4.2	78.9	3.0	20.3	113.7
1993–1997	1.8	13.7	121.1	6.6	54.0	134.1	0.4	4.8	91.8	2.2	16.9	106.4
1998–2002	0.6	12.3	77.8	7.2	70.7	111.3	0.6	6.8	96.2	3.2	22.7	120.4
2003–2007	1.2	16.5	94.8	7.6	65.8	114.0	0.4	7.4	82.1	2.4	18.2	108.4
2008–2012	1.4	20.6	97.2	5.8	46.9	96.5	0.4	8.2	84.3	1.4	13.4	90.1
	25–34			65–74			25–34			65–74		
1983–1987	5.2	35.7	139.5	2.0	36.5	83.3	0.6	8.0	74.6	2.6	29.2	93.5
1988–1992	2.8	23.6	123.4	1.8	28.6	81.3	1.8	11.5	133.1	2.4	23.7	90.7
1993–1997	3.2	26.7	136.9	2.2	27.1	84.9	0.8	8.2	98.9	3.0	22.9	117.6
1998–2002	4.2	36.0	133.4	4.0	43.0	94.6	1.0	11.3	97.6	1.6	16.4	78.2
2003–2007	2.4	29.6	95.6	6.8	62.2	145.0	1.2	13.2	101.3	1.4	15.4	82.7
2008–2012	2.0	29.4	90.2	3.2	39.0	98.7	0.2	10.5	73.0	1.2	15.3	85.9
	35–44			>74 y/o			35–44			>74 y/o		
1983–1987	6.4	46.3	145.4	2.4	68.1	87.0	1.2	11.2	94.6	2.2	39.1	67.7
1988–1992	3.6	26.0	109.9	3.0	65.0	100.7	0.6	7.8	81.1	3.6	43.0	89.8
1993–1997	3.4	26.5	108.2	2.2	45.5	87.4	0.4	6.3	74.6	2.6	27.2	78.9
1998–2002	7.0	54.7	155.2	4.2	62.5	118.6	1.2	10.6	102.9	1.6	16.6	53.5
2003–2007	5.2	50.7	126.5	3.0	40.6	90.2	1.0	11.9	93.8	2.0	15.5	68.9
2008–2012	4.0	41.1	110.5	3.2	37.6	90.6	0.4	10.8	79.4	3.4	20.2	104.9

Secondary Medical Zone ID: 299

	Male						Female					
	Suicide			Suicide			Suicide			Suicide		
	Num per year	Rate /100,000	×100	Num per year	Rate /100,000	×100	Num per year	Rate /100,000	×100	Num per year	Rate /100,000	×100
	Total (>10 y/o)			45–54			Total (>10 y/o)			45–54		
1983–1987	81.0	35.0	110.5	21.0	58.3	115.4	30.4	12.2	75.7	4.0	11.8	68.8
1988–1992	66.0	26.8	111.7	14.4	40.0	116.3	30.2	10.7	78.6	6.0	15.0	102.3
1993–1997	66.0	26.1	104.8	17.0	41.3	112.8	25.2	8.7	74.0	5.4	12.6	97.3
1998–2002	92.2	36.0	99.8	28.6	64.6	115.4	31.8	10.9	78.6	5.4	12.8	84.7
2003–2007	110.6	42.6	118.7	29.0	72.9	130.4	32.8	11.5	84.2	6.4	14.8	108.3
2008–2012	88.6	34.9	104.5	19.0	55.9	114.9	29.8	11.0	80.0	5.4	14.7	98.8
	15–24			55–64			15–24			55–64		
1983–1987	5.0	13.2	94.3	14.6	49.4	116.3	3.0	7.0	102.5	6.0	17.7	89.8
1988–1992	3.4	9.1	91.7	12.6	38.4	108.2	2.4	5.4	103.1	4.4	13.2	74.1
1993–1997	4.8	12.1	107.0	13.8	42.1	104.5	1.6	4.3	82.0	5.0	13.7	86.4
1998–2002	4.8	14.6	92.2	19.0	58.8	92.6	2.4	6.7	95.7	7.8	19.9	106.0
2003–2007	4.4	15.6	89.5	24.6	64.8	112.2	3.0	9.2	102.7	4.4	12.5	74.7
2008–2012	3.2	15.1	71.1	27.8	63.4	130.6	1.6	7.1	73.2	6.8	15.0	100.8
	25–34			65–74			25–34			65–74		
1983–1987	13.6	31.1	121.5	5.6	37.0	84.6	3.8	8.9	83.0	5.2	25.1	80.2
1988–1992	6.8	19.2	100.5	6.4	33.1	93.8	2.4	6.8	78.8	5.6	21.1	81.0
1993–1997	6.4	19.6	100.4	6.6	27.9	87.3	1.8	6.0	72.4	3.0	11.1	56.7
1998–2002	6.6	22.0	81.4	11.4	42.3	93.0	3.6	10.4	89.6	2.8	10.7	50.7
2003–2007	13.6	40.8	131.6	10.8	39.1	91.2	4.8	13.6	104.3	4.4	13.8	74.2
2008–2012	8.4	31.5	96.7	8.2	31.4	79.5	3.2	12.1	84.4	4.2	13.7	76.9
	35–44			>74 y/o			35–44			>74 y/o		
1983–1987	16.4	37.7	118.5	4.2	57.2	73.1	4.8	10.9	92.4	3.6	31.0	53.6
1988–1992	15.8	33.1	139.7	6.4	60.6	93.9	3.4	7.9	82.1	6.0	33.0	68.9
1993–1997	12.0	28.7	117.1	5.2	42.3	81.3	2.8	7.0	82.9	5.4	23.5	68.2
1998–2002	15.6	43.3	122.9	6.2	40.5	76.8	4.0	10.4	100.5	5.8	19.9	64.2
2003–2007	16.4	49.8	124.3	11.6	54.0	119.9	3.6	10.9	86.0	6.0	16.3	72.4
2008–2012	12.0	37.3	100.2	10.0	40.7	98.0	3.0	10.2	75.2	5.6	13.3	69.0

Secondary Medical Zone ID: 300

	Male						Female					
	Suicide			Suicide			Suicide			Suicide		
	Num per year	Rate /100,000	×100	Num per year	Rate /100,000	×100	Num per year	Rate /100,000	×100	Num per year	Rate /100,000	×100
	Total (>10 y/o)			45–54			Total (>10 y/o)			45–54		
1983–1987	58.4	38.3	121.8	11.4	50.5	99.8	30.2	15.8	104.7	6.6	22.3	129.6
1988–1992	48.0	29.0	124.3	10.8	46.2	134.5	24.2	12.2	92.2	3.4	14.0	95.6
1993–1997	48.0	28.1	117.9	14.8	53.3	145.7	19.8	10.0	87.2	4.8	15.8	122.4
1998–2002	68.6	40.9	116.5	18.0	65.0	116.2	24.2	12.2	91.6	5.2	17.3	115.0
2003–2007	81.4	47.8	137.8	19.8	79.7	142.5	22.4	12.2	91.6	3.0	12.9	94.2
2008–2012	56.8	36.5	108.0	10.6	52.8	108.6	21.2	12.4	91.7	1.8	11.5	76.9
	15–24			55–64			15–24			55–64		
1983–1987	3.4	16.3	116.7	10.6	49.4	116.3	1.2	6.1	89.8	5.0	19.5	98.8
1988–1992	2.4	10.6	107.0	11.8	49.1	138.4	1.8	6.7	127.6	5.2	19.1	107.0
1993–1997	2.8	12.0	106.5	8.8	41.1	102.2	0.8	4.4	84.2	2.6	12.1	76.1
1998–2002	3.0	15.3	96.7	15.8	74.5	117.2	1.0	6.1	86.3	4.2	17.8	94.7
2003–2007	3.2	17.5	100.5	20.4	81.6	141.3	1.8	9.8	108.9	5.4	19.0	113.5
2008–2012	5.4	28.9	136.5	14.8	54.7	112.5	1.0	8.3	85.7	3.6	14.0	93.7
	25–34			65–74			25–34			65–74		
1983–1987	7.8	31.1	121.5	5.6	45.7	104.3	3.6	12.8	118.9	4.2	25.9	82.8
1988–1992	5.2	23.9	125.3	6.0	40.3	114.4	2.2	9.4	108.8	2.8	16.1	61.6
1993–1997	1.6	12.4	63.5	7.8	42.2	132.1	1.8	8.5	102.6	2.6	13.0	66.8
1998–2002	4.6	24.8	91.9	10.4	52.5	115.4	2.4	11.6	99.7	3.8	17.0	81.0
2003–2007	7.6	36.8	118.6	10.2	51.9	120.9	1.4	9.8	75.3	4.0	17.4	93.3
2008–2012	5.8	33.6	103.0	6.8	38.8	98.2	2.6	14.7	102.5	3.0	15.0	84.5
	35–44			>74 y/o			35–44			>74 y/o		
1983–1987	14.0	49.3	154.9	5.6	84.6	108.1	3.4	12.0	101.9	6.2	59.1	102.5
1988–1992	7.4	26.2	110.4	4.2	54.9	85.2	2.8	9.8	101.2	6.0	42.3	88.4
1993–1997	8.2	31.4	127.9	4.0	44.9	86.3	2.4	9.1	107.1	4.8	27.7	80.5
1998–2002	12.2	53.4	151.6	4.6	42.9	81.4	2.4	10.8	104.1	5.2	24.1	77.6
2003–2007	13.4	64.3	160.6	6.6	45.5	101.0	3.0	13.8	109.0	3.6	14.4	64.1
2008–2012	6.2	34.2	92.0	7.2	42.5	102.5	3.8	16.2	119.4	5.4	17.6	91.7

Secondary Medical Zone ID: 301

	Male						Female					
	Suicide			Suicide			Suicide			Suicide		
	Num per year	Rate /100,000	×100	Num per year	Rate /100,000	×100	Num per year	Rate /100,000	×100	Num per year	Rate /100,000	×100
	Total (>10 y/o)			45–54			Total (>10 y/o)			45–54		
1983–1987	37.8	38.2	125.5	11.0	70.5	139.3	14.2	13.7	85.9	2.8	17.6	102.3
1988–1992	24.2	24.2	97.5	5.0	34.1	99.2	15.6	12.2	94.0	1.8	13.0	89.1
1993–1997	28.6	25.7	102.6	7.2	39.7	108.4	13.4	10.3	89.4	3.6	16.1	124.8
1998–2002	48.8	39.1	113.1	13.2	64.1	114.5	16.6	12.0	91.0	2.6	13.9	92.1
2003–2007	52.8	41.5	117.3	12.8	65.7	117.5	15.8	11.6	89.3	2.6	13.5	98.2
2008–2012	44.8	35.3	106.8	8.0	48.1	98.9	14.6	11.8	85.2	1.8	12.3	82.4
	15–24			55–64			15–24			55–64		
1983–1987	3.2	19.5	139.6	4.6	40.7	95.9	1.2	7.2	106.0	1.8	16.0	80.9
1988–1992	2.0	11.3	113.7	4.6	34.0	95.8	0.4	4.0	76.1	3.8	21.5	120.2
1993–1997	1.6	10.7	94.2	5.4	38.5	95.6	0.6	4.4	85.2	2.0	14.0	87.9
1998–2002	2.0	14.3	90.7	11.2	72.1	113.6	1.0	6.7	95.0	3.4	19.6	104.3
2003–2007	2.0	15.7	89.9	12.8	69.8	120.9	0.4	5.8	64.9	3.0	16.3	97.5
2008–2012	2.0	18.5	87.0	10.0	49.8	102.6	0.6	7.2	73.9	2.6	13.7	92.0
	25–34			65–74			25–34			65–74		
1983–1987	6.0	33.0	129.3	3.6	50.2	114.7	1.4	9.3	86.3	3.4	33.7	107.9
1988–1992	3.0	19.6	102.8	2.0	28.7	81.4	1.8	9.8	112.9	2.6	23.5	90.1
1993–1997	2.6	18.2	93.1	3.2	31.1	97.4	1.0	7.3	87.9	2.6	19.1	98.2
1998–2002	4.4	27.2	100.8	6.4	49.7	109.3	1.6	10.5	90.8	2.8	19.5	92.7
2003–2007	5.8	33.9	109.5	5.4	41.9	97.6	1.6	11.2	86.1	3.2	19.7	105.7
2008–2012	5.0	33.6	103.0	7.0	47.5	120.1	2.6	15.5	108.5	2.2	15.7	88.5
	35–44			>74 y/o			35–44			>74 y/o		
1983–1987	5.6	33.0	103.7	3.4	84.9	108.4	1.0	8.8	74.1	2.6	44.1	76.4
1988–1992	4.6	23.9	100.7	3.0	61.7	95.6	2.2	10.3	107.0	3.0	35.2	73.6
1993–1997	6.6	32.2	131.5	1.6	35.2	67.5	1.8	8.9	105.1	1.8	19.1	55.6
1998–2002	7.8	42.5	120.6	3.6	51.6	97.8	1.8	10.1	98.1	3.4	25.5	82.0
2003–2007	9.4	53.2	132.7	4.6	48.5	107.7	2.0	12.0	95.2	3.0	18.4	81.9
2008–2012	7.4	41.4	111.3	5.4	45.5	109.8	2.2	13.0	95.7	2.4	13.7	71.4

Secondary Medical Zone ID: 302

	Male						Female					
	Suicide			Suicide			Suicide			Suicide		
	Num per year	Rate /100,000	×100	Num per year	Rate /100,000	×100	Num per year	Rate /100,000	×100	Num per year	Rate /100,000	×100
	Total (>10 y/o)			45–54			Total (>10 y/o)			45–54		
1983–1987	20.8	30.6	91.1	4.2	42.0	83.1	14.0	14.9	97.4	2.4	18.1	105.0
1988–1992	24.0	28.3	126.0	3.6	35.8	104.1	14.2	13.2	105.5	2.4	17.1	116.7
1993–1997	18.6	25.4	100.0	4.2	38.5	105.3	14.0	11.8	119.3	1.8	14.1	109.2
1998–2002	30.6	38.5	112.7	6.2	54.4	97.2	13.6	13.1	106.2	1.8	15.2	100.7
2003–2007	32.2	41.2	120.1	8.8	73.1	130.6	11.8	12.3	102.5	1.4	13.2	96.3
2008–2012	25.8	35.1	108.2	5.0	50.3	103.5	8.6	11.7	88.8	0.4	10.8	72.2
	15–24			55–64			15–24			55–64		
1983–1987	0.8	11.1	79.9	4.4	42.9	100.8	1.0	7.7	113.3	2.2	18.6	94.5
1988–1992	1.2	10.9	110.3	5.8	46.3	130.4	0.2	4.3	81.8	2.4	18.2	101.8
1993–1997	0.6	10.1	89.0	3.4	36.0	89.5	0.0	3.8	72.5	2.6	18.6	117.0
1998–2002	1.2	15.6	99.0	7.2	72.2	113.7	0.4	6.6	93.5	3.0	22.1	117.6
2003–2007	1.0	16.7	95.6	5.6	56.5	97.9	0.0	6.2	69.1	2.6	19.2	114.8
2008–2012	1.0	20.5	96.7	6.0	50.8	104.6	0.4	9.1	93.6	1.4	13.9	93.0
	25–34			65–74			25–34			65–74		
1983–1987	3.8	29.6	115.9	3.2	49.4	112.9	0.4	7.4	68.4	3.2	34.4	110.2
1988–1992	2.2	20.9	109.2	4.0	47.3	134.3	0.6	7.6	87.1	2.8	27.1	103.9
1993–1997	1.2	17.4	89.2	3.0	33.3	104.5	1.0	9.2	110.5	2.6	21.4	109.9
1998–2002	2.2	28.0	103.7	4.0	43.0	94.6	0.8	11.1	95.4	3.4	25.0	118.8
2003–2007	2.4	32.3	104.1	5.0	49.6	115.6	0.4	10.7	82.4	1.8	17.1	91.8
2008–2012	1.4	29.0	89.1	3.8	42.6	107.8	0.8	13.8	96.3	1.6	16.8	94.7
	35–44			>74 y/o			35–44			>74 y/o		
1983–1987	3.4	31.5	99.0	1.0	43.9	56.0	1.4	12.0	101.3	3.4	54.2	93.9
1988–1992	4.6	32.0	135.2	2.2	54.8	85.0	1.2	9.7	100.7	4.6	53.9	112.6
1993–1997	4.2	32.2	131.2	2.0	44.1	84.6	0.8	7.9	92.5	5.2	50.3	146.1
1998–2002	5.4	47.0	133.3	4.4	65.4	124.1	1.4	11.6	112.3	2.8	25.8	83.0
2003–2007	5.2	53.8	134.3	4.2	51.1	113.5	1.4	13.6	108.0	4.2	28.3	126.3
2008–2012	3.6	41.5	111.6	5.0	50.2	121.0	0.4	11.2	82.3	3.6	21.6	112.2

Secondary Medical Zone ID: 303

	Male						Female					
	Suicide			Suicide			Suicide			Suicide		
	Num per year	Rate /100,000	×100	Num per year	Rate /100,000	×100	Num per year	Rate /100,000	×100	Num per year	Rate /100,000	×100
	Total (>10 y/o)			45–54			Total (>10 y/o)			45–54		
1983–1987	11.0	36.1	129.7	1.8	50.0	98.8	5.4	15.7	107.2	0.8	17.8	103.1
1988–1992	7.6	26.9	120.0	1.8	42.1	122.4	4.6	12.9	105.1	0.6	15.0	102.7
1993–1997	6.8	26.3	110.9	1.8	42.1	115.0	2.2	10.0	82.1	0.4	12.8	99.2
1998–2002	10.2	38.3	115.9	1.4	50.3	89.8	2.8	12.1	88.1	0.8	16.2	107.9
2003–2007	10.4	40.1	122.7	2.6	65.3	116.8	3.8	13.0	104.0	0.8	15.2	111.1
2008–2012	10.2	38.1	125.3	2.2	56.7	116.8	1.8	12.5	87.9	0.0	12.6	84.6
	15–24			55–64			15–24			55–64		
1983–1987	0.4	14.1	101.3	2.8	57.2	134.5	0.0	6.0	88.0	1.8	25.9	131.4
1988–1992	0.2	9.8	98.7	1.2	34.8	98.0	0.0	4.7	89.3	0.6	16.9	94.9
1993–1997	0.2	11.1	98.3	1.6	42.9	106.7	0.0	4.7	90.6	0.6	15.8	99.2
1998–2002	0.4	16.3	103.4	3.0	77.9	122.6	0.0	6.4	91.3	0.2	16.7	88.6
2003–2007	0.8	20.6	118.2	3.2	75.3	130.5	0.2	9.3	103.2	0.8	17.9	106.9
2008–2012	0.8	25.1	118.2	1.6	48.2	99.2	0.2	10.3	105.8	0.6	15.3	102.4
	25–34			65–74			25–34			65–74		
1983–1987	1.4	29.4	114.8	1.4	51.6	117.8	1.0	13.9	129.2	0.6	27.0	86.4
1988–1992	1.8	30.1	157.5	0.6	32.4	91.9	0.4	9.2	106.4	1.0	27.6	105.6
1993–1997	1.0	23.7	121.4	0.6	28.3	88.6	0.0	7.3	88.2	0.4	16.3	83.5
1998–2002	1.2	31.5	116.7	0.8	39.2	86.2	0.0	10.4	89.9	0.8	20.7	98.4
2003–2007	0.4	29.5	95.1	1.6	46.9	109.4	0.2	12.8	98.3	0.4	16.7	89.9
2008–2012	0.6	33.5	102.9	1.2	41.4	104.9	0.4	15.3	106.6	0.2	15.9	89.1
	35–44			>74 y/o			35–44			>74 y/o		
1983–1987	1.6	37.0	116.2	1.6	96.3	123.0	0.2	10.9	91.9	1.0	51.9	89.9
1988–1992	1.2	26.4	111.5	0.8	60.9	94.5	0.2	9.1	93.7	1.8	59.5	124.4
1993–1997	1.2	27.6	112.7	0.4	41.4	79.5	0.4	8.9	105.2	0.4	22.2	64.5
1998–2002	2.4	49.1	139.3	0.8	48.9	92.9	0.4	10.7	103.6	0.6	22.9	73.6
2003–2007	1.6	48.5	121.0	0.2	31.0	68.8	0.4	13.0	102.8	0.8	19.9	88.8
2008–2012	2.0	46.1	123.8	1.6	47.4	114.2	0.2	13.2	97.3	0.2	12.7	66.3

Secondary Medical Zone ID: 304

	Male						Female					
	Suicide			Suicide			Suicide			Suicide		
	Num per year	Rate /100,000	×100	Num per year	Rate /100,000	×100	Num per year	Rate /100,000	×100	Num per year	Rate /100,000	×100
	Total (>10 y/o)			45-54			Total (>10 y/o)			45-54		
1983-1987	6.0	32.9	105.9	1.6	53.0	104.8	1.2	13.6	74.3	0.0	14.3	83.2
1988-1992	3.2	23.9	90.5	1.4	41.4	120.5	0.8	11.2	70.8	0.0	13.0	88.8
1993-1997	3.2	24.3	91.8	0.6	35.0	95.7	1.2	10.1	82.6	0.0	11.6	89.6
1998-2002	6.2	37.7	108.6	0.8	50.3	89.8	1.0	11.8	81.0	0.0	13.5	90.0
2003-2007	4.8	36.3	98.6	0.8	49.0	87.6	1.4	12.3	90.9	0.2	13.3	97.0
2008-2012	4.2	34.0	100.4	0.8	47.8	98.4	2.2	13.1	103.0	0.0	13.4	90.0
	15-24			55-64			15-24			55-64		
1983-1987	0.4	14.5	103.9	1.4	46.4	109.2	0.0	6.2	91.0	0.2	17.0	86.0
1988-1992	0.2	10.0	101.2	1.0	36.7	103.3	0.0	4.9	92.5	0.6	18.3	102.7
1993-1997	0.2	11.5	101.4	0.8	38.9	96.7	0.0	4.9	93.7	0.2	14.4	90.5
1998-2002	0.6	18.3	115.6	1.4	65.8	103.6	0.2	7.5	107.2	0.2	17.6	93.6
2003-2007	0.0	16.5	94.3	1.2	59.1	102.4	0.2	9.7	107.9	0.2	16.1	95.9
2008-2012	0.0	20.1	95.0	1.2	49.9	102.7	0.2	10.6	109.5	0.8	17.0	114.2
	25-34			65-74			25-34			65-74		
1983-1987	1.0	27.8	108.9	0.8	47.5	108.4	0.4	11.5	106.8	0.2	25.7	82.3
1988-1992	0.2	17.1	89.5	0.2	29.7	84.3	0.2	8.8	101.3	0.0	18.0	69.1
1993-1997	0.4	20.2	103.4	0.4	28.9	90.8	0.0	7.7	92.2	0.4	18.3	93.9
1998-2002	0.8	30.4	112.6	0.4	39.5	86.8	0.2	11.8	101.6	0.4	19.7	93.6
2003-2007	0.4	31.6	101.9	1.2	47.7	111.2	0.0	12.2	94.1	0.2	16.8	90.2
2008-2012	0.0	30.5	93.8	1.2	45.1	114.2	0.2	14.8	103.3	0.0	15.7	88.0
	35-44			>74 y/o			35-44			>74 y/o		
1983-1987	0.4	28.2	88.5	0.4	72.6	92.7	0.0	10.5	88.9	0.4	46.0	79.8
1988-1992	0.2	20.1	84.8	0.0	47.4	73.5	0.0	8.7	90.4	0.0	27.1	56.6
1993-1997	0.4	22.5	91.9	0.4	49.2	94.5	0.2	8.6	100.6	0.4	28.9	84.1
1998-2002	2.0	50.2	142.4	0.2	43.0	81.6	0.0	9.3	89.6	0.0	17.9	57.5
2003-2007	0.8	42.5	106.1	0.4	40.1	89.1	0.4	13.6	107.8	0.2	16.2	72.2
2008-2012	0.4	36.7	98.7	0.6	39.8	95.9	0.2	13.7	100.9	0.8	21.2	110.0

Secondary Medical Zone ID: 305

	Male						Female					
	Suicide			Suicide			Suicide			Suicide		
	Num per year	Rate /100,000	×100	Num per year	Rate /100,000	×100	Num per year	Rate /100,000	×100	Num per year	Rate /100,000	×100
	Total (>10 y/o)			45–54			Total (>10 y/o)			45–54		
1983–1987	7.0	34.3	118.6	1.4	51.8	102.4	3.8	15.6	108.3	0.2	15.6	90.8
1988–1992	6.8	27.5	135.3	0.6	32.8	95.4	2.0	12.1	87.3	0.4	14.9	101.6
1993–1997	4.8	25.5	108.8	1.2	39.7	108.4	2.0	10.6	94.0	0.4	13.3	103.2
1998–2002	6.0	36.1	102.9	0.2	43.0	76.8	2.0	12.2	92.0	0.4	15.2	101.2
2003–2007	8.6	40.3	133.7	2.6	77.7	138.9	2.2	12.6	98.5	0.6	15.2	111.0
2008–2012	6.4	36.5	113.6	1.8	58.5	120.4	2.2	12.8	100.7	0.4	15.7	105.0
	15–24			55–64			15–24			55–64		
1983–1987	0.4	15.2	108.7	2.0	53.6	126.2	0.2	7.1	104.4	1.2	24.1	122.1
1988–1992	0.2	10.0	101.1	2.2	49.6	139.8	0.0	4.9	92.4	0.2	15.9	89.0
1993–1997	0.6	13.2	116.9	0.8	38.7	96.2	0.6	7.4	142.3	0.2	14.4	90.8
1998–2002	0.4	16.7	105.6	1.8	70.7	111.2	0.2	7.5	106.0	0.8	20.7	110.1
2003–2007	0.2	17.3	99.4	1.8	64.9	112.4	0.0	8.3	92.5	0.2	15.9	94.8
2008–2012	0.4	22.6	106.3	1.0	47.4	97.5	0.0	9.1	93.6	0.4	15.1	101.3
	25–34			65–74			25–34			65–74		
1983–1987	0.8	27.6	108.1	0.6	43.3	99.0	0.2	10.5	97.6	0.4	28.3	90.4
1988–1992	1.0	25.5	133.6	0.6	35.4	100.6	0.0	7.9	90.7	0.4	23.3	89.4
1993–1997	0.2	18.7	95.9	0.8	33.8	106.0	0.2	8.7	104.0	0.0	14.1	72.5
1998–2002	0.8	30.0	111.2	0.6	40.6	89.3	0.0	10.8	93.2	0.0	16.2	77.2
2003–2007	0.6	32.4	104.3	1.4	49.4	115.1	0.2	13.1	100.7	0.0	15.4	82.7
2008–2012	0.4	32.4	99.4	0.6	38.6	97.7	0.0	13.4	93.4	0.4	17.9	100.7
	35–44			>74 y/o			35–44			>74 y/o		
1983–1987	1.4	38.1	119.7	0.4	67.8	86.5	0.8	14.2	120.0	0.8	56.7	98.3
1988–1992	1.0	27.3	115.3	1.2	80.3	124.5	0.6	11.1	115.1	0.4	35.7	74.5
1993–1997	0.6	25.0	101.9	0.6	52.1	100.1	0.0	7.5	88.8	0.6	31.7	92.0
1998–2002	1.2	41.4	117.6	1.0	58.7	111.4	0.0	9.3	90.1	0.6	27.5	88.4
2003–2007	0.8	42.5	106.1	1.2	52.8	117.3	0.4	13.6	107.9	0.8	23.9	106.5
2008–2012	1.4	43.5	116.9	0.8	41.1	99.0	0.2	13.6	100.1	0.8	20.5	106.5

Secondary Medical Zone ID: 306

	Male						Female					
	Suicide			Suicide			Suicide			Suicide		
	Num per year	Rate /100,000	×100	Num per year	Rate /100,000	×100	Num per year	Rate /100,000	×100	Num per year	Rate /100,000	×100
	Total (>10 y/o)			45–54			Total (>10 y/o)			45–54		
1983–1987	6.4	32.5	100.5	2.0	53.8	106.3	1.8	13.8	77.4	0.2	15.0	86.9
1988–1992	6.8	26.8	122.5	1.6	40.8	118.8	2.6	12.3	92.1	0.4	14.4	98.6
1993–1997	4.8	25.1	97.9	1.0	36.0	98.5	3.2	11.1	108.3	0.2	12.1	93.4
1998–2002	8.2	37.1	109.1	2.4	61.9	110.6	2.8	12.7	97.5	0.4	14.8	98.1
2003–2007	9.8	39.2	127.3	2.0	59.9	107.0	4.2	13.2	117.5	0.0	11.9	86.7
2008–2012	7.8	35.6	115.3	1.2	48.5	99.8	2.4	13.1	99.8	0.4	15.3	102.6
	15–24			55–64			15–24			55–64		
1983–1987	0.4	14.6	104.4	1.6	47.8	112.6	0.0	6.1	89.6	0.2	16.9	85.6
1988–1992	0.4	10.7	108.6	1.2	36.8	103.6	0.0	4.8	90.9	0.2	15.5	87.0
1993–1997	0.2	11.2	98.6	1.0	38.5	95.6	0.0	4.8	91.9	0.8	17.8	112.0
1998–2002	0.8	18.7	118.4	1.8	66.0	103.8	0.0	6.5	92.5	0.6	19.1	101.7
2003–2007	0.4	18.3	104.8	3.4	80.9	140.1	0.4	10.7	119.0	1.6	21.8	129.9
2008–2012	0.2	21.0	99.0	2.2	55.0	113.2	0.2	10.5	107.9	0.4	14.7	98.9
	25–34			65–74			25–34			65–74		
1983–1987	0.4	21.6	84.3	0.8	46.8	106.9	0.0	8.7	81.1	0.4	28.1	89.8
1988–1992	1.4	26.8	140.1	0.6	35.2	99.8	0.2	8.4	97.4	0.4	23.2	89.0
1993–1997	0.8	22.4	114.4	0.6	30.7	96.2	0.0	7.5	89.7	1.2	26.2	134.3
1998–2002	0.4	25.8	95.4	1.4	48.4	106.3	0.2	11.4	98.7	0.6	20.7	98.7
2003–2007	0.8	32.5	104.7	1.4	46.9	109.3	0.2	12.8	98.4	1.2	23.2	124.4
2008–2012	1.0	36.0	110.7	1.4	45.1	114.2	0.2	14.2	99.3	0.8	19.7	110.6
	35–44			>74 y/o			35–44			>74 y/o		
1983–1987	1.0	31.8	100.0	0.2	61.9	79.1	0.4	11.8	100.1	0.6	51.1	88.6
1988–1992	1.2	26.7	112.8	0.4	57.4	89.0	1.0	12.3	127.4	0.4	36.9	77.2
1993–1997	1.2	28.1	114.6	0.0	35.7	68.6	0.8	11.1	130.6	0.2	23.6	68.6
1998–2002	1.2	37.6	106.8	0.2	41.5	78.8	0.8	13.1	126.3	0.2	21.0	67.6
2003–2007	1.0	41.5	103.6	0.8	45.8	101.7	0.2	12.3	97.6	0.6	21.6	96.1
2008–2012	0.6	36.3	97.6	1.2	46.0	111.0	0.2	13.4	98.8	0.2	14.9	77.5

Secondary Medical Zone ID: 307

	Male						Female					
	Suicide			Suicide			Suicide			Suicide		
	Num per year	Rate /100,000	×100	Num per year	Rate /100,000	×100	Num per year	Rate /100,000	×100	Num per year	Rate /100,000	×100
	Total (>10 y/o)			45–54			Total (>10 y/o)			45–54		
1983–1987	78.0	32.1	98.8	18.0	48.4	95.7	38.0	13.4	85.3	7.0	16.6	96.1
1988–1992	67.0	24.8	100.7	15.4	38.5	112.1	29.2	9.6	68.7	4.4	11.3	77.1
1993–1997	65.6	22.9	89.2	19.8	41.7	114.0	34.0	9.8	84.7	5.4	11.2	86.7
1998–2002	105.8	34.7	94.6	29.0	58.0	103.6	44.2	11.9	89.9	8.2	15.1	100.4
2003–2007	114.4	36.5	98.1	29.2	61.9	110.7	45.2	11.7	92.5	7.8	14.9	108.4
2008–2012	104.6	33.7	95.6	21.8	50.0	102.8	44.6	12.7	91.0	6.0	13.0	87.3
	15–24			55–64			15–24			55–64		
1983–1987	7.4	15.2	109.2	11.8	41.4	97.4	3.4	6.9	101.2	6.0	17.5	88.9
1988–1992	4.2	8.4	84.8	11.2	33.1	93.1	1.8	4.0	75.4	5.2	14.2	79.7
1993–1997	4.4	8.5	75.1	12.8	36.7	91.1	2.4	4.7	89.8	6.2	15.1	94.8
1998–2002	6.2	12.3	77.7	23.4	63.1	99.4	3.2	6.6	93.8	7.2	17.2	91.2
2003–2007	5.4	12.7	72.8	24.6	56.7	98.2	2.8	6.9	76.3	10.4	19.8	118.5
2008–2012	6.8	17.8	83.9	23.6	49.3	101.5	3.4	8.8	90.3	8.2	15.3	102.8
	25–34			65–74			25–34			65–74		
1983–1987	11.6	25.4	99.3	6.8	42.0	95.9	5.2	10.4	96.9	5.2	24.2	77.6
1988–1992	8.2	19.0	99.2	6.6	33.7	95.5	3.0	6.8	78.8	3.6	14.8	56.7
1993–1997	6.8	16.3	83.2	4.8	21.1	66.1	6.0	11.0	132.3	5.0	15.6	80.1
1998–2002	14.6	30.2	111.8	10.6	36.8	81.0	7.4	13.5	116.6	9.0	23.2	110.5
2003–2007	15.8	32.8	105.6	12.6	40.1	93.4	5.4	11.1	85.6	7.4	18.6	99.9
2008–2012	10.4	25.8	79.1	11.8	36.9	93.4	7.0	14.7	102.5	5.0	13.8	77.5
	35–44			>74 y/o			35–44			>74 y/o		
1983–1987	17.4	36.3	113.9	4.6	55.3	70.6	5.0	10.2	86.2	6.2	43.2	74.9
1988–1992	16.4	30.8	130.1	4.8	43.1	66.8	5.4	9.7	100.9	5.8	28.1	58.6
1993–1997	11.0	23.0	93.7	5.6	40.8	78.4	4.2	8.2	96.4	4.8	18.9	54.8
1998–2002	16.2	35.8	101.8	5.6	34.5	65.5	2.8	6.8	66.1	6.0	18.7	60.2
2003–2007	19.0	42.7	106.6	7.8	34.9	77.4	4.2	9.6	76.2	7.2	17.5	78.0
2008–2012	22.6	46.3	124.5	7.6	28.8	69.5	9.6	17.2	127.1	5.2	11.5	59.9

Secondary Medical Zone ID: 308

	Male						Female					
	Suicide			Suicide			Suicide			Suicide		
	Num per year	Rate /100,000	×100	Num per year	Rate /100,000	×100	Num per year	Rate /100,000	×100	Num per year	Rate /100,000	×100
	Total (>10 y/o)			45–54			Total (>10 y/o)			45–54		
1983–1987	17.0	34.4	110.9	4.4	57.9	114.5	7.8	14.5	90.7	1.0	15.7	90.9
1988–1992	15.6	28.0	122.3	3.6	44.0	128.1	6.0	11.5	79.9	1.2	15.0	102.8
1993–1997	11.6	24.6	92.6	2.6	35.6	97.3	5.0	9.6	78.3	1.0	12.9	100.0
1998–2002	18.2	35.4	96.6	5.0	58.5	104.6	7.0	12.1	88.0	1.2	14.9	98.9
2003–2007	20.2	37.4	104.1	5.4	64.7	115.6	5.6	11.6	81.8	0.8	12.6	91.9
2008–2012	19.6	36.0	107.5	4.8	58.8	121.1	8.4	12.8	102.8	1.0	14.6	97.7
	15–24			55–64			15–24			55–64		
1983–1987	1.6	18.3	131.2	4.2	54.9	129.3	0.8	8.0	117.8	0.6	14.7	74.7
1988–1992	0.4	9.2	92.5	2.4	34.5	97.2	0.0	4.1	77.1	0.6	14.0	78.1
1993–1997	0.2	9.4	82.7	2.4	37.5	93.1	0.2	4.7	90.8	0.4	11.7	73.3
1998–2002	1.0	15.9	100.4	3.4	56.2	88.4	0.4	6.9	97.7	1.0	17.0	90.6
2003–2007	0.8	16.4	94.2	4.8	60.7	105.1	0.4	8.4	93.5	1.2	16.3	97.3
2008–2012	1.2	22.0	103.8	4.0	47.9	98.7	0.4	9.3	95.5	2.0	17.3	116.3
	25–34			65–74			25–34			65–74		
1983–1987	3.2	34.2	133.6	0.8	33.0	75.3	1.0	11.6	107.9	2.2	35.7	114.2
1988–1992	2.8	30.1	157.5	1.6	35.4	100.4	1.2	11.1	128.6	0.8	18.3	70.1
1993–1997	1.8	23.8	121.8	1.0	24.5	76.9	0.4	7.9	94.5	0.8	14.9	76.6
1998–2002	0.8	22.7	84.1	2.8	44.2	97.3	1.2	13.4	115.8	1.0	17.2	82.0
2003–2007	2.6	35.9	115.8	2.4	40.1	93.4	0.4	11.3	86.8	0.8	15.4	82.5
2008–2012	1.8	32.2	98.9	2.6	40.6	102.8	0.8	14.2	99.2	1.0	16.4	92.4
	35–44			>74 y/o			35–44			>74 y/o		
1983–1987	2.0	29.4	92.3	0.8	52.5	67.1	1.0	12.2	103.5	1.2	38.8	67.3
1988–1992	3.4	32.7	138.1	1.2	50.4	78.1	1.0	10.4	108.0	1.2	30.0	62.8
1993–1997	2.2	26.7	108.7	1.4	45.3	87.0	0.6	8.3	97.1	1.6	27.8	80.9
1998–2002	3.4	42.1	119.6	1.8	47.6	90.3	0.8	10.5	102.0	1.4	21.7	69.8
2003–2007	2.0	35.7	89.1	2.2	43.4	96.4	1.0	13.2	104.7	1.0	14.1	62.9
2008–2012	3.0	41.7	112.2	2.2	37.9	91.2	0.6	12.6	92.9	2.6	22.0	114.6

Secondary Medical Zone ID: 309

	Male						Female					
	Suicide			Suicide			Suicide			Suicide		
	Num per year	Rate /100,000	×100	Num per year	Rate /100,000	×100	Num per year	Rate /100,000	×100	Num per year	Rate /100,000	×100
	Total (>10 y/o)			45–54			Total (>10 y/o)			45–54		
1983–1987	24.4	31.8	97.1	6.2	52.1	103.1	11.0	13.3	79.5	2.2	16.9	98.1
1988–1992	24.6	27.9	119.4	3.6	33.3	97.0	12.0	11.8	89.6	1.2	12.7	86.8
1993–1997	23.0	26.7	109.1	7.6	51.2	139.9	10.6	10.3	91.1	1.2	11.5	89.0
1998–2002	30.0	36.5	99.8	8.0	58.2	104.0	13.6	12.6	98.8	2.0	15.0	99.5
2003–2007	38.6	42.7	125.8	8.4	65.8	117.7	12.8	12.2	100.0	1.2	12.1	88.3
2008–2012	31.4	36.2	112.8	5.0	50.3	103.4	13.2	13.2	104.8	2.0	16.6	111.4
	15–24			55–64			15–24			55–64		
1983–1987	1.6	14.9	107.1	5.6	48.7	114.7	1.0	7.7	113.3	2.0	17.3	87.8
1988–1992	0.8	9.4	94.8	4.2	35.8	100.8	0.4	4.8	91.7	2.0	16.3	91.2
1993–1997	1.6	12.9	114.1	4.2	38.4	95.3	0.0	3.5	68.0	2.2	16.3	102.5
1998–2002	1.4	15.1	95.6	6.8	62.6	98.6	0.8	7.3	103.9	3.2	21.7	115.1
2003–2007	2.2	20.2	115.5	8.6	66.3	114.9	0.6	8.0	89.4	3.4	20.5	122.2
2008–2012	1.8	22.3	105.2	8.2	55.0	113.2	0.6	8.9	91.9	3.4	18.7	125.4
	25–34			65–74			25–34			65–74		
1983–1987	4.0	29.7	116.2	1.2	27.9	63.8	1.2	10.3	95.3	1.8	23.8	76.3
1988–1992	4.2	32.5	170.1	2.8	35.2	99.9	0.8	8.3	96.3	3.4	29.6	113.3
1993–1997	1.0	16.1	82.6	2.2	26.5	82.9	1.0	9.1	109.9	3.0	22.7	116.4
1998–2002	2.4	27.6	102.1	3.6	39.2	86.1	1.0	11.5	98.9	3.0	22.3	106.4
2003–2007	4.0	38.1	123.0	6.0	52.9	123.4	0.2	9.4	72.0	2.8	20.4	109.8
2008–2012	2.6	31.7	97.2	4.8	44.6	113.0	1.2	14.3	99.9	1.2	14.4	81.2
	35–44			>74 y/o			35–44			>74 y/o		
1983–1987	3.2	27.8	87.3	2.6	67.5	86.2	1.2	10.8	91.4	1.6	32.2	55.9
1988–1992	5.6	34.6	145.8	3.4	66.4	102.9	1.0	8.8	91.5	3.2	40.3	84.2
1993–1997	3.8	28.9	118.1	2.6	47.0	90.2	1.2	9.1	106.5	2.0	22.4	65.1
1998–2002	4.8	43.2	122.7	2.8	44.1	83.6	1.0	10.0	97.0	2.6	22.7	73.2
2003–2007	4.8	49.7	124.0	4.6	51.4	114.2	1.2	12.7	100.6	3.4	22.5	100.4
2008–2012	3.8	40.1	107.9	5.0	48.2	116.2	1.2	13.5	99.7	3.6	20.5	106.7

Secondary Medical Zone ID: 310

	Male						Female					
	Suicide			Suicide			Suicide			Suicide		
	Num per year	Rate /100,000	×100	Num per year	Rate /100,000	×100	Num per year	Rate /100,000	×100	Num per year	Rate /100,000	×100
	Total (>10 y/o)			45–54			Total (>10 y/o)			45–54		
1983–1987	10.8	35.0	116.7	2.2	53.0	104.9	3.2	13.5	77.1	0.8	17.4	100.9
1988–1992	8.2	25.8	112.8	2.0	41.7	121.4	4.8	12.5	97.9	0.6	14.6	100.0
1993–1997	7.2	25.6	101.4	1.6	37.8	103.3	4.6	11.0	104.1	0.4	12.3	95.1
1998–2002	10.2	36.1	100.9	3.0	61.1	109.2	4.4	12.5	96.1	0.6	14.8	98.2
2003–2007	11.0	38.1	107.9	1.4	45.9	82.1	4.0	12.4	96.8	0.2	12.1	88.5
2008–2012	9.4	33.9	103.0	1.0	43.4	89.4	2.4	12.1	85.7	0.4	14.4	96.4
	15–24			55–64			15–24			55–64		
1983–1987	0.4	13.7	98.2	1.4	40.6	95.5	0.2	6.7	98.1	0.4	16.4	82.9
1988–1992	0.6	11.2	112.8	1.8	39.1	110.1	0.2	5.4	102.0	1.4	20.8	116.7
1993–1997	0.2	10.6	94.0	2.8	51.8	128.6	0.0	4.6	87.3	1.0	17.6	110.7
1998–2002	0.8	17.5	110.9	1.6	56.4	88.8	0.2	7.0	99.4	0.8	18.9	100.5
2003–2007	0.4	17.0	97.4	3.2	67.5	116.9	0.4	9.9	110.1	1.6	20.4	122.1
2008–2012	0.2	19.2	90.8	3.0	54.8	112.8	0.2	9.5	97.6	0.4	13.9	92.9
	25–34			65–74			25–34			65–74		
1983–1987	1.6	30.1	117.8	1.4	48.5	110.7	0.2	9.5	88.1	0.8	28.1	89.9
1988–1992	0.6	19.0	99.5	0.8	33.0	93.5	0.4	9.1	105.1	0.8	23.5	90.0
1993–1997	0.8	21.6	110.4	0.6	26.5	83.2	0.2	8.1	97.1	0.6	17.2	88.2
1998–2002	2.0	35.8	132.6	1.6	44.9	98.8	0.8	13.6	117.5	1.0	21.3	101.6
2003–2007	1.4	35.5	114.4	1.4	41.8	97.3	0.2	12.2	94.0	0.8	18.6	99.7
2008–2012	0.6	30.7	94.4	1.6	42.3	107.2	0.2	13.5	94.4	0.6	17.3	97.2
	35–44			>74 y/o			35–44			>74 y/o		
1983–1987	1.4	33.0	103.5	2.4	110.2	140.7	0.4	11.4	96.3	0.4	32.2	55.8
1988–1992	1.2	24.9	105.2	1.0	58.7	91.1	0.4	9.5	98.8	1.0	37.3	78.0
1993–1997	1.2	26.4	107.8	0.0	27.4	52.7	0.6	9.7	113.8	1.8	41.5	120.6
1998–2002	0.6	29.7	84.2	0.6	39.3	74.5	0.2	9.6	92.5	0.8	22.5	72.4
2003–2007	2.2	53.7	134.0	1.0	38.8	86.3	0.2	11.8	93.1	0.6	15.7	70.0
2008–2012	1.0	37.0	99.5	2.0	47.1	113.6	0.0	11.9	87.7	0.6	14.6	75.8

Secondary Medical Zone ID: 311

	Male						Female					
	Suicide			Suicide			Suicide			Suicide		
	Num per year	Rate /100,000	×100	Num per year	Rate /100,000	×100	Num per year	Rate /100,000	×100	Num per year	Rate /100,000	×100
	Total (>10 y/o)			45-54			Total (>10 y/o)			45-54		
1983-1987	20.8	35.6	118.3	4.2	53.0	104.8	7.6	13.9	85.5	0.8	14.2	82.5
1988-1992	12.2	22.6	86.7	2.4	31.6	92.1	9.4	12.7	98.4	2.0	17.2	117.5
1993-1997	20.0	27.7	116.9	3.4	34.9	95.5	7.2	10.1	86.2	1.0	11.7	91.0
1998-2002	28.8	38.9	110.9	8.6	67.2	120.0	11.2	13.0	101.0	2.4	16.9	112.4
2003-2007	32.0	40.5	115.0	8.8	72.1	129.0	9.2	11.8	89.1	1.0	11.6	84.9
2008-2012	29.8	35.3	108.1	5.4	50.4	103.7	9.6	12.0	90.2	1.4	14.0	93.9
	15-24			55-64			15-24			55-64		
1983-1987	2.0	19.4	138.8	4.4	53.1	125.0	0.8	7.7	113.2	1.4	18.7	94.7
1988-1992	0.8	9.7	98.4	1.8	27.9	78.4	0.6	5.7	108.1	1.6	17.7	99.4
1993-1997	1.0	10.9	96.8	4.6	48.4	120.2	0.4	4.8	92.9	1.2	14.6	91.8
1998-2002	2.2	18.2	115.5	5.6	64.7	101.9	0.6	6.7	95.0	1.0	15.9	84.2
2003-2007	1.2	15.5	89.0	5.8	56.5	97.9	1.0	9.6	106.6	2.0	17.3	103.1
2008-2012	1.6	19.7	92.8	7.8	57.1	117.6	0.6	8.7	89.4	1.2	12.9	86.3
	25-34			65-74			25-34			65-74		
1983-1987	2.6	27.0	105.7	1.2	36.9	84.2	1.2	11.4	105.6	1.4	28.8	92.2
1988-1992	0.8	14.3	74.8	1.4	31.2	88.6	0.8	8.7	100.3	1.6	24.9	95.3
1993-1997	1.8	20.6	105.5	2.2	32.7	102.7	0.8	8.5	102.2	1.8	20.9	107.4
1998-2002	2.4	26.7	98.9	2.4	38.0	83.7	1.0	11.2	96.9	2.2	22.7	107.9
2003-2007	3.2	30.6	98.6	4.0	47.6	110.9	1.2	12.4	95.4	2.2	20.9	112.3
2008-2012	3.0	29.8	91.4	3.8	43.5	110.2	1.4	13.7	95.4	0.8	14.5	81.4
	35-44			>74 y/o			35-44			>74 y/o		
1983-1987	4.0	37.7	118.5	2.4	90.4	115.4	1.0	11.4	96.6	1.0	36.4	63.1
1988-1992	3.2	26.8	112.9	1.8	59.7	92.6	1.0	9.5	97.9	1.8	37.2	77.6
1993-1997	4.6	35.1	143.1	2.4	58.8	112.9	0.8	7.9	93.5	1.2	22.2	64.6
1998-2002	5.2	45.4	128.8	2.4	51.5	97.7	2.2	14.4	138.9	1.8	24.0	77.2
2003-2007	5.6	52.0	129.8	3.4	51.3	113.9	0.6	10.2	81.0	1.2	14.6	64.9
2008-2012	3.8	36.0	96.8	4.4	52.1	125.6	1.2	12.6	92.8	3.0	22.6	117.3

Secondary Medical Zone ID: 312

	Male						Female					
	Suicide			Suicide			Suicide			Suicide		
	Num per year	Rate /100,000	×100	Num per year	Rate /100,000	×100	Num per year	Rate /100,000	×100	Num per year	Rate /100,000	×100
	Total (>10 y/o)			45–54			Total (>10 y/o)			45–54		
1983–1987	15.4	37.1	130.7	3.6	61.0	120.6	8.6	16.9	120.5	1.8	21.1	122.5
1988–1992	11.4	27.7	123.7	2.4	42.5	123.7	7.8	14.1	120.2	1.8	19.0	130.1
1993–1997	12.2	28.6	126.8	2.4	41.3	112.9	4.4	10.3	93.2	0.8	13.7	105.9
1998–2002	19.0	41.3	136.8	3.6	61.1	109.2	5.2	12.5	95.4	0.4	13.6	90.0
2003–2007	17.8	42.4	133.1	3.4	62.5	111.8	7.2	13.4	117.4	1.2	16.0	116.6
2008–2012	18.0	39.3	136.8	4.8	72.7	149.5	6.2	13.1	109.5	0.8	15.7	105.6
	15–24			55–64			15–24			55–64		
1983–1987	0.4	12.7	90.9	3.6	56.1	132.0	0.6	8.2	119.7	1.0	18.9	95.7
1988–1992	0.6	10.7	108.4	1.0	27.7	78.0	0.2	5.3	100.6	0.6	15.4	86.3
1993–1997	0.8	12.8	113.0	1.8	38.8	96.5	0.4	6.1	116.4	1.0	16.3	102.8
1998–2002	1.2	18.9	119.9	5.2	88.3	139.1	0.4	7.7	109.1	0.2	15.4	81.6
2003–2007	0.6	17.4	99.6	3.8	67.3	116.7	0.0	7.4	82.6	1.4	19.0	113.3
2008–2012	0.6	20.9	98.5	3.4	52.6	108.3	0.2	9.4	96.6	1.4	17.0	114.0
	25–34			65–74			25–34			65–74		
1983–1987	3.0	38.0	148.6	1.2	43.5	99.3	1.6	16.1	149.2	0.4	22.4	71.6
1988–1992	1.2	22.3	116.7	2.0	44.8	127.2	0.4	8.8	101.9	1.4	27.8	106.6
1993–1997	1.4	24.9	127.6	1.4	32.2	101.0	0.0	7.1	84.8	1.0	19.1	98.1
1998–2002	2.0	35.0	129.5	2.6	49.3	108.3	0.6	12.6	108.5	1.6	23.7	112.8
2003–2007	2.2	40.8	131.6	4.2	64.5	150.4	1.0	16.0	122.8	1.2	19.6	105.1
2008–2012	1.6	36.7	112.8	2.2	44.5	112.7	0.4	14.3	99.8	0.8	17.4	97.8
	35–44			>74 y/o			35–44			>74 y/o		
1983–1987	2.6	40.7	127.7	1.0	65.3	83.4	1.0	13.7	116.0	2.2	71.6	124.1
1988–1992	2.6	33.3	140.4	1.6	68.7	106.4	0.8	10.8	111.8	2.6	63.6	132.9
1993–1997	3.2	40.6	165.7	1.2	50.0	96.1	0.0	6.7	78.6	1.2	29.8	86.5
1998–2002	2.0	40.1	113.7	2.4	64.8	122.8	0.8	12.1	117.0	1.2	25.0	80.5
2003–2007	2.8	56.6	141.3	0.8	32.0	71.2	0.2	11.4	90.5	2.2	28.6	127.5
2008–2012	1.4	38.8	104.3	4.0	62.8	151.5	0.2	12.5	92.2	2.4	25.3	131.4

Secondary Medical Zone ID: 313

	Male						Female					
	Suicide			Suicide			Suicide			Suicide		
	Num per year	Rate /100,000	×100	Num per year	Rate /100,000	×100	Num per year	Rate /100,000	×100	Num per year	Rate /100,000	×100
	Total (>10 y/o)			45–54			Total (>10 y/o)			45–54		
1983–1987	15.4	35.8	118.8	3.6	56.5	111.8	5.4	14.0	83.2	1.2	17.6	102.2
1988–1992	9.4	24.3	95.9	3.0	43.9	127.9	5.0	11.5	82.4	0.6	13.5	92.6
1993–1997	12.6	26.9	113.8	2.6	38.9	106.3	5.2	10.5	91.0	0.4	11.3	87.6
1998–2002	14.6	35.4	95.4	4.6	60.8	108.6	6.4	12.3	94.5	1.2	15.7	104.3
2003–2007	17.6	38.4	110.2	4.0	59.9	107.1	7.2	12.7	104.9	1.0	14.2	103.3
2008–2012	17.8	36.3	116.5	2.6	49.1	101.0	9.6	14.2	123.3	1.2	16.6	111.2
	15–24			55–64			15–24			55–64		
1983–1987	1.4	18.9	135.5	2.8	46.8	110.1	0.0	5.5	79.9	0.6	16.0	80.9
1988–1992	0.4	9.7	97.6	1.6	31.0	87.3	0.2	5.0	94.7	1.2	17.8	99.7
1993–1997	0.6	11.5	101.3	3.8	51.4	127.6	0.8	7.2	137.3	1.8	19.9	125.2
1998–2002	0.2	12.9	81.6	2.2	51.9	81.7	0.4	7.2	102.7	1.4	19.8	105.4
2003–2007	1.0	18.6	106.4	4.4	64.4	111.6	0.2	8.0	88.9	1.4	18.0	107.2
2008–2012	1.2	23.6	111.3	4.4	53.7	110.5	0.6	11.1	114.6	1.0	14.7	98.7
	25–34			65–74			25–34			65–74		
1983–1987	2.2	30.4	119.0	1.2	41.2	94.0	1.0	12.5	115.8	0.8	25.3	81.1
1988–1992	1.4	22.7	119.0	1.4	35.5	100.8	0.0	6.8	77.9	0.4	16.8	64.4
1993–1997	1.0	20.7	106.0	1.6	32.3	101.4	0.2	7.5	90.7	0.6	14.9	76.7
1998–2002	1.2	27.3	101.1	1.8	39.8	87.6	0.6	11.8	102.1	0.8	17.4	82.9
2003–2007	1.0	28.0	90.4	1.8	38.5	89.7	0.6	12.9	99.3	1.4	19.8	106.6
2008–2012	2.0	36.0	110.6	2.4	42.7	108.2	1.2	16.9	117.6	1.8	21.3	119.6
	35–44			>74 y/o			35–44			>74 y/o		
1983–1987	2.8	38.8	122.1	1.4	72.9	93.0	0.6	11.5	97.1	1.2	43.8	76.0
1988–1992	0.4	16.0	67.4	1.2	56.2	87.1	0.4	8.9	92.1	2.0	47.5	99.2
1993–1997	2.6	32.6	132.8	0.2	27.5	52.9	0.6	8.8	103.3	0.8	21.2	61.7
1998–2002	2.6	41.6	118.2	2.0	54.1	102.6	0.2	8.7	83.9	1.8	28.5	91.9
2003–2007	2.8	50.4	125.8	2.6	51.6	114.6	0.6	12.5	99.2	2.0	24.3	108.5
2008–2012	1.4	35.7	95.9	3.8	56.5	136.3	0.6	13.4	98.9	3.2	28.5	148.4

Secondary Medical Zone ID: 314

	Male						Female					
	Suicide			Suicide			Suicide			Suicide		
	Num per year	Rate /100,000	×100	Num per year	Rate /100,000	×100	Num per year	Rate /100,000	×100	Num per year	Rate /100,000	×100
	Total (>10 y/o)			45-54			Total (>10 y/o)			45-54		
1983-1987	25.0	35.7	114.9	6.4	57.2	113.1	10.6	14.0	87.9	2.2	17.5	101.6
1988-1992	23.0	29.2	130.7	5.4	46.3	134.8	9.2	11.6	84.1	1.6	14.4	98.3
1993-1997	23.6	28.3	127.4	6.2	47.8	130.5	9.4	10.6	94.6	1.6	13.3	102.7
1998-2002	33.2	40.8	124.8	10.0	76.4	136.5	12.0	12.8	101.2	2.0	15.8	104.9
2003-2007	32.8	42.5	125.8	9.0	78.9	141.0	11.8	12.7	104.9	1.4	13.5	98.3
2008-2012	27.2	36.5	115.3	3.8	45.6	93.8	7.6	11.7	84.2	0.8	12.6	84.5
	15-24			55-64			15-24			55-64		
1983-1987	1.4	14.6	104.5	3.8	42.3	99.4	0.2	5.3	77.5	2.4	20.7	104.8
1988-1992	1.0	10.3	103.7	5.0	43.8	123.2	0.4	5.0	94.0	1.4	14.9	83.4
1993-1997	0.8	10.5	92.9	6.4	54.3	135.0	0.4	5.0	95.2	2.4	18.0	113.2
1998-2002	1.4	16.1	101.9	7.2	71.8	113.0	0.2	5.8	81.8	2.8	21.2	112.9
2003-2007	1.2	17.0	97.3	6.6	61.8	107.1	0.2	6.9	77.1	1.8	16.4	97.9
2008-2012	1.4	21.5	101.5	8.6	64.4	132.6	0.2	7.7	79.4	1.0	12.6	84.8
	25-34			65-74			25-34			65-74		
1983-1987	3.8	31.2	122.0	1.6	37.6	85.8	0.2	6.9	64.1	2.0	29.3	93.8
1988-1992	3.6	29.9	156.4	2.0	34.3	97.5	1.6	11.3	130.4	1.2	18.5	71.1
1993-1997	0.8	15.2	78.0	3.6	40.8	128.0	1.4	10.6	127.9	1.2	14.9	76.3
1998-2002	1.8	25.2	93.3	6.0	58.1	127.8	1.0	11.7	100.9	1.4	16.3	77.5
2003-2007	2.4	31.3	100.9	5.6	55.6	129.5	1.4	14.5	111.7	2.4	20.0	107.2
2008-2012	2.6	34.8	106.9	3.2	39.1	98.9	0.8	13.5	94.3	1.4	16.0	89.9
	35-44			>74 y/o			35-44			>74 y/o		
1983-1987	5.6	42.5	133.5	2.4	81.6	104.2	0.6	9.2	77.9	3.0	61.1	105.9
1988-1992	2.6	22.9	96.4	3.4	85.4	132.4	0.8	8.6	89.0	2.2	38.0	79.4
1993-1997	2.8	25.7	104.6	3.0	66.6	127.9	0.8	8.0	94.4	1.6	24.3	70.5
1998-2002	4.0	40.0	113.7	2.8	55.5	105.2	1.6	12.6	121.8	3.0	31.1	100.1
2003-2007	4.6	49.9	124.4	3.2	47.4	105.3	1.2	13.0	102.9	3.4	26.6	118.7
2008-2012	3.0	37.7	101.2	4.2	48.1	115.9	1.2	13.9	102.6	2.2	16.6	86.1

Secondary Medical Zone ID: 315

	Male						Female					
	Suicide			Suicide			Suicide			Suicide		
	Num per year	Rate /100,000	×100	Num per year	Rate /100,000	×100	Num per year	Rate /100,000	×100	Num per year	Rate /100,000	×100
	Total (>10 y/o)			45–54			Total (>10 y/o)			45–54		
1983–1987	9.0	32.0	98.0	1.0	37.6	74.4	10.6	18.6	147.8	1.4	20.0	116.3
1988–1992	10.4	27.3	129.1	2.0	40.1	116.8	8.0	14.5	130.1	0.8	15.2	104.1
1993–1997	9.8	27.0	121.8	2.6	44.7	122.3	4.6	11.0	101.9	0.6	13.1	101.3
1998–2002	10.2	35.8	100.2	3.2	62.9	112.4	4.2	12.1	93.3	0.2	13.1	87.3
2003–2007	12.2	38.8	118.7	2.6	61.0	109.1	5.0	12.6	106.8	0.6	13.9	101.6
2008–2012	9.6	34.9	108.0	1.8	51.6	106.2	3.0	12.8	91.9	0.2	13.4	89.9
	15–24			55–64			15–24			55–64		
1983–1987	0.8	16.6	119.2	1.4	39.2	92.2	0.2	6.6	96.8	1.8	24.3	123.3
1988–1992	0.2	9.5	96.1	2.6	45.6	128.4	0.4	6.2	117.4	2.0	23.5	131.7
1993–1997	0.2	10.8	95.6	2.2	45.1	112.1	0.0	4.6	88.3	0.4	13.7	86.5
1998–2002	0.0	13.6	85.8	2.8	67.6	106.5	0.0	6.3	89.7	1.4	21.5	114.3
2003–2007	0.4	17.6	100.6	2.0	55.1	95.5	0.2	9.0	100.5	1.0	17.9	107.1
2008–2012	0.8	24.1	113.6	2.4	51.2	105.4	0.4	11.1	114.9	0.4	13.9	93.4
	25–34			65–74			25–34			65–74		
1983–1987	1.6	29.8	116.5	1.2	44.3	101.2	0.8	12.4	114.9	3.4	55.5	177.6
1988–1992	0.8	20.6	107.6	1.6	42.0	119.2	0.4	9.0	104.2	1.4	28.6	109.7
1993–1997	0.8	21.5	109.9	1.0	30.6	95.8	0.0	7.2	86.6	1.0	19.9	102.1
1998–2002	0.8	27.6	102.1	1.2	40.6	89.3	0.2	11.1	95.5	0.8	19.4	92.2
2003–2007	1.4	36.6	118.1	3.6	64.0	149.1	0.0	11.4	87.9	0.8	18.3	98.2
2008–2012	0.2	29.2	89.8	1.8	43.4	109.9	0.6	15.9	111.0	0.2	15.1	84.7
	35–44			>74 y/o			35–44			>74 y/o		
1983–1987	1.2	30.6	96.1	1.8	91.8	117.3	1.0	13.8	117.1	2.0	72.4	125.4
1988–1992	1.0	23.3	98.2	2.2	86.4	134.0	1.4	13.3	137.4	1.6	48.7	101.7
1993–1997	0.8	22.8	93.1	2.2	74.5	143.1	1.4	13.3	156.8	1.2	31.5	91.7
1998–2002	0.6	29.6	84.0	1.6	56.9	108.0	0.0	8.6	83.0	1.6	32.1	103.2
2003–2007	1.4	44.0	109.8	0.8	36.2	80.5	0.2	11.9	94.0	2.2	31.4	140.0
2008–2012	0.4	33.5	90.1	2.2	50.3	121.1	0.0	12.1	89.1	1.2	18.9	98.1

Secondary Medical Zone ID: 316

	Male						Female					
	Suicide			Suicide			Suicide			Suicide		
	Num per year	Rate /100,000	×100	Num per year	Rate /100,000	×100	Num per year	Rate /100,000	×100	Num per year	Rate /100,000	×100
	Total (>10 y/o)			45-54			Total (>10 y/o)			45-54		
1983-1987	19.2	36.1	120.3	5.6	65.4	129.2	14.6	18.5	139.6	2.0	19.3	112.3
1988-1992	21.4	32.5	161.6	4.8	54.0	157.3	10.2	13.6	114.3	0.4	12.0	82.0
1993-1997	21.2	31.0	154.7	3.8	44.2	120.8	7.0	10.6	97.5	1.4	14.6	113.0
1998-2002	24.4	40.7	130.7	7.2	76.0	135.9	11.6	13.8	124.8	1.2	15.2	100.8
2003-2007	26.0	44.9	142.6	7.8	90.5	161.9	7.6	12.4	100.8	1.2	14.5	106.1
2008-2012	22.8	38.9	135.0	5.0	65.1	134.0	8.2	13.2	108.1	1.2	16.2	108.6
	15-24			55-64			15-24			55-64		
1983-1987	0.4	11.5	82.7	3.4	45.6	107.2	0.8	8.2	120.7	2.2	22.1	111.9
1988-1992	0.8	11.0	111.5	3.8	43.6	122.7	0.4	5.7	108.5	1.8	18.7	104.8
1993-1997	0.4	10.6	93.8	5.2	57.5	142.7	0.0	4.2	81.2	2.0	19.1	120.2
1998-2002	0.2	13.0	82.4	2.8	54.4	85.7	0.6	8.0	114.3	2.2	22.1	117.5
2003-2007	0.6	16.7	96.0	5.2	68.6	118.8	0.2	8.1	90.0	1.0	15.9	95.0
2008-2012	0.6	20.6	97.3	5.0	56.0	115.2	0.2	9.0	93.1	2.0	17.8	119.2
	25-34			65-74			25-34			65-74		
1983-1987	2.0	26.0	101.6	3.0	58.7	134.1	0.8	10.6	98.0	3.8	49.0	156.8
1988-1992	1.8	23.6	123.3	2.6	44.3	125.6	0.6	8.8	101.4	3.6	40.6	155.5
1993-1997	1.0	19.6	100.3	4.2	52.3	163.9	0.2	7.3	87.4	2.2	23.7	121.5
1998-2002	2.0	31.4	116.4	6.2	69.5	152.9	0.8	12.5	107.5	3.4	30.3	144.1
2003-2007	2.0	35.2	113.5	3.4	49.1	114.3	0.0	10.2	78.6	2.0	21.2	114.0
2008-2012	2.6	40.4	124.1	2.4	41.5	104.9	0.6	14.3	100.1	0.8	15.8	88.9
	35-44			>74 y/o			35-44			>74 y/o		
1983-1987	2.8	34.8	109.3	2.0	85.0	108.5	1.4	13.7	116.1	3.6	89.5	155.1
1988-1992	3.8	35.2	148.3	3.6	102.8	159.3	0.8	9.8	101.2	2.6	52.8	110.4
1993-1997	2.8	31.4	128.3	3.8	89.8	172.4	0.6	8.4	98.8	0.6	17.0	49.3
1998-2002	3.2	43.2	122.7	2.8	62.4	118.4	0.4	9.2	89.4	3.0	36.9	118.8
2003-2007	3.2	51.2	127.7	3.8	60.9	135.3	0.8	13.1	103.8	2.4	24.5	109.0
2008-2012	1.4	35.2	94.6	5.8	70.9	170.8	0.8	14.1	104.2	2.6	22.0	114.4

Secondary Medical Zone ID: 317

	Male						Female					
	Suicide			Suicide			Suicide			Suicide		
	Num per year	Rate /100,000	×100	Num per year	Rate /100,000	×100	Num per year	Rate /100,000	×100	Num per year	Rate /100,000	×100
	Total (>10 y/o)			45-54			Total (>10 y/o)			45-54		
1983-1987	20.0	31.1	90.5	5.0	49.3	97.4	12.6	14.5	90.4	1.8	16.3	94.5
1988-1992	18.6	25.2	104.9	2.6	31.1	90.4	8.6	10.6	73.3	0.4	10.9	74.8
1993-1997	19.2	26.3	106.5	3.4	35.7	97.4	9.4	10.4	89.0	2.2	16.0	124.0
1998-2002	25.2	36.7	100.8	5.6	53.5	95.6	12.2	12.8	100.3	1.4	14.5	96.1
2003-2007	27.2	39.6	112.4	8.4	75.9	135.7	10.4	11.9	98.7	2.0	16.0	116.5
2008-2012	25.8	38.6	118.3	7.2	69.4	142.8	9.8	12.7	100.9	1.0	13.9	93.0
	15-24			55-64			15-24			55-64		
1983-1987	1.4	15.8	113.1	2.4	30.8	72.4	0.6	7.0	102.5	1.8	16.7	84.5
1988-1992	0.2	8.2	82.6	5.8	46.8	131.9	0.0	4.0	75.0	1.4	14.4	80.7
1993-1997	0.6	10.8	95.2	3.8	39.3	97.7	0.6	6.1	117.3	1.2	13.1	82.7
1998-2002	1.2	16.9	107.1	6.0	66.6	104.8	0.0	5.5	78.3	3.6	25.0	133.1
2003-2007	0.8	17.2	98.4	6.8	66.9	115.9	0.0	6.8	75.1	1.8	17.2	102.5
2008-2012	0.8	21.1	99.7	6.2	54.1	111.3	0.4	9.8	101.0	1.4	14.4	96.5
	25-34			65-74			25-34			65-74		
1983-1987	2.4	23.8	93.3	3.4	49.6	113.4	2.4	15.4	143.0	2.8	30.6	97.8
1988-1992	2.6	24.6	128.5	1.8	29.2	82.9	0.6	7.9	91.0	2.0	21.0	80.6
1993-1997	1.8	21.7	111.3	3.6	37.5	117.6	0.6	8.1	97.3	2.6	20.7	106.2
1998-2002	2.0	28.2	104.6	3.6	40.7	89.5	0.8	11.5	99.6	2.2	19.2	91.4
2003-2007	1.4	28.1	90.6	2.8	36.2	84.4	0.4	11.3	87.1	1.8	17.4	93.3
2008-2012	3.0	40.5	124.3	2.4	35.1	88.8	0.6	13.8	96.1	2.6	21.4	120.0
	35-44			>74 y/o			35-44			>74 y/o		
1983-1987	3.8	35.2	110.5	1.6	51.1	65.3	0.4	9.0	76.4	2.8	44.8	77.6
1988-1992	3.0	25.6	108.2	2.6	58.2	90.2	0.6	8.3	85.9	3.6	42.5	88.7
1993-1997	3.6	30.5	124.3	2.2	45.3	87.0	0.6	7.5	88.1	1.6	19.1	55.4
1998-2002	4.0	41.2	116.9	2.8	47.7	90.6	1.2	11.4	110.3	3.0	25.3	81.5
2003-2007	3.4	44.9	112.0	3.6	46.4	103.1	0.2	9.7	77.2	4.2	27.2	121.4
2008-2012	2.0	35.6	95.6	4.2	45.4	109.5	0.4	11.8	86.8	3.4	20.4	106.3

Secondary Medical Zone ID: 318

	Male						Female					
	Suicide			Suicide			Suicide			Suicide		
	Num per year	Rate /100,000	×100	Num per year	Rate /100,000	×100	Num per year	Rate /100,000	×100	Num per year	Rate /100,000	×100
	Total (>10 y/o)			45–54			Total (>10 y/o)			45–54		
1983–1987	32.0	33.7	103.8	8.2	54.7	108.1	19.0	15.0	97.9	3.2	17.7	102.6
1988–1992	25.0	24.8	100.8	4.2	31.3	91.2	15.2	11.9	86.3	3.2	16.6	113.2
1993–1997	23.2	23.9	91.6	5.0	34.2	93.6	12.4	9.6	82.8	2.0	12.1	94.0
1998–2002	42.0	38.5	111.2	9.6	60.9	108.9	18.0	12.7	99.7	3.6	18.0	119.3
2003–2007	39.0	37.6	104.6	7.0	53.4	95.5	20.8	14.3	118.1	3.4	18.1	132.0
2008–2012	35.6	35.1	103.9	7.2	56.5	116.3	14.2	12.8	92.5	1.6	13.9	93.3
	15–24			55–64			15–24			55–64		
1983–1987	1.0	10.8	77.6	5.2	39.9	93.8	1.8	9.2	134.0	4.4	22.7	115.2
1988–1992	1.0	9.1	91.7	6.2	39.6	111.7	0.2	3.7	69.7	2.8	16.3	91.4
1993–1997	1.4	11.2	98.9	5.0	36.6	91.0	0.6	4.8	92.7	2.2	13.9	87.2
1998–2002	3.0	19.8	125.0	10.0	69.4	109.2	0.2	5.0	71.8	3.6	19.9	105.7
2003–2007	2.4	18.8	108.0	9.6	61.8	107.0	1.4	10.4	115.7	4.2	19.9	118.9
2008–2012	2.4	22.4	105.5	7.0	46.4	95.5	1.4	11.5	118.1	1.6	12.3	82.7
	25–34			65–74			25–34			65–74		
1983–1987	4.0	29.0	113.6	3.2	39.8	91.0	1.0	8.9	82.9	2.4	23.6	75.4
1988–1992	2.6	21.2	111.0	3.0	32.2	91.4	1.8	10.8	125.1	1.6	15.1	57.8
1993–1997	2.0	18.8	96.4	4.0	32.9	103.2	1.4	9.4	113.1	1.4	11.7	60.0
1998–2002	3.2	27.1	100.2	6.8	49.1	107.9	1.6	11.9	102.3	3.2	19.4	92.2
2003–2007	3.2	28.3	91.2	7.2	51.1	119.1	1.8	13.5	103.8	2.8	17.4	93.7
2008–2012	3.6	32.4	99.5	4.8	38.2	96.8	1.8	15.1	105.1	2.0	14.9	84.0
	35–44			>74 y/o			35–44			>74 y/o		
1983–1987	7.6	43.3	136.1	2.8	61.9	79.0	2.6	13.1	111.0	3.6	48.3	83.7
1988–1992	4.4	26.9	113.3	3.6	59.6	92.3	2.2	11.1	115.4	3.2	33.0	68.9
1993–1997	3.6	25.9	105.7	2.2	36.7	70.5	0.8	7.2	84.5	4.0	32.0	92.9
1998–2002	4.8	38.3	108.7	4.4	52.3	99.2	1.4	10.6	102.3	4.4	28.3	91.2
2003–2007	6.0	48.6	121.4	3.6	36.5	81.1	2.6	16.0	126.5	4.6	23.8	105.9
2008–2012	4.4	36.8	98.9	6.2	47.1	113.5	2.0	14.4	105.9	3.8	17.7	92.2

Secondary Medical Zone ID: 319

	Male						Female					
	Suicide			Suicide			Suicide			Suicide		
	Num per year	Rate /100,000	×100	Num per year	Rate /100,000	×100	Num per year	Rate /100,000	×100	Num per year	Rate /100,000	×100
	Total (>10 y/o)			45−54			Total (>10 y/o)			45−54		
1983−1987	64.0	32.4	98.6	16.6	53.0	104.8	34.4	14.9	98.1	6.6	18.9	109.5
1988−1992	53.2	24.1	98.4	11.6	33.8	98.6	29.2	11.5	87.9	4.4	12.9	88.5
1993−1997	53.4	23.0	89.3	14.2	34.9	95.5	25.0	9.4	82.0	5.4	12.7	98.4
1998−2002	86.0	34.4	94.0	26.2	60.0	107.3	30.2	10.9	81.0	6.8	15.0	99.7
2003−2007	90.2	35.4	95.5	22.4	58.5	104.6	28.8	10.6	78.7	5.8	14.0	101.9
2008−2012	85.2	33.9	95.7	15.2	46.3	95.2	34.8	12.4	91.6	5.4	14.8	99.3
	15−24			55−64			15−24			55−64		
1983−1987	4.4	13.3	95.1	8.8	38.1	89.8	1.8	5.8	85.3	6.8	22.9	116.3
1988−1992	2.4	7.4	74.8	11.4	39.2	110.4	0.8	3.3	62.1	5.8	18.1	101.5
1993−1997	5.0	11.7	103.5	9.8	34.0	84.5	1.6	4.5	86.8	6.2	17.8	112.1
1998−2002	5.4	14.9	94.4	17.8	55.3	87.0	1.8	5.9	83.8	4.8	15.1	80.1
2003−2007	5.0	16.4	94.1	21.0	53.7	93.1	0.8	4.7	52.6	4.2	12.1	72.4
2008−2012	7.2	24.2	114.3	21.2	49.7	102.3	2.4	9.2	95.2	8.0	16.8	112.3
	25−34			65−74			25−34			65−74		
1983−1987	10.2	27.3	107.0	5.0	39.1	89.4	4.0	10.1	93.8	4.0	24.4	77.9
1988−1992	3.8	13.3	69.4	5.2	32.6	92.5	2.2	6.9	79.7	6.6	29.3	112.4
1993−1997	5.2	17.0	87.0	3.4	19.3	60.6	2.4	7.3	87.6	3.4	14.2	72.7
1998−2002	10.2	28.5	105.5	10.6	42.8	94.1	4.8	12.3	106.0	4.2	15.6	74.2
2003−2007	9.4	26.6	85.7	9.0	35.0	81.5	4.0	11.3	86.7	4.4	15.1	80.9
2008−2012	11.0	32.4	99.5	10.0	35.3	89.3	5.6	15.5	108.5	5.2	16.1	90.6
	35−44			>74 y/o			35−44			>74 y/o		
1983−1987	12.4	29.7	93.4	6.6	88.1	112.5	5.4	12.2	102.9	5.6	53.1	92.1
1988−1992	12.2	27.3	115.3	6.4	67.5	104.7	4.4	9.6	99.8	5.0	34.0	71.0
1993−1997	9.6	24.7	100.9	6.2	54.1	103.9	2.8	7.3	85.4	3.2	18.3	53.2
1998−2002	11.6	33.9	96.4	4.2	33.5	63.6	2.6	8.0	77.5	5.2	21.7	70.0
2003−2007	15.4	45.4	113.2	8.0	42.9	95.3	4.0	11.5	91.4	5.4	17.4	77.7
2008−2012	13.4	36.3	97.5	6.8	31.1	75.1	3.4	10.4	77.0	4.8	13.3	69.4

Secondary Medical Zone ID: 320

	Male						Female					
	Suicide			Suicide			Suicide			Suicide		
	Num per year	Rate /100,000	×100	Num per year	Rate /100,000	×100	Num per year	Rate /100,000	×100	Num per year	Rate /100,000	×100
	Total (>10 y/o)			45-54			Total (>10 y/o)			45-54		
1983-1987	14.8	34.9	112.3	3.0	48.8	96.5	6.4	14.2	87.9	0.4	13.3	77.2
1988-1992	12.6	27.0	120.1	2.4	37.7	109.7	7.4	12.8	102.7	0.6	13.1	89.8
1993-1997	10.4	25.2	101.7	2.6	38.8	105.9	5.4	10.2	93.2	0.4	11.0	85.4
1998-2002	20.2	41.5	129.3	6.4	78.3	139.8	6.6	12.3	97.8	1.4	16.5	109.6
2003-2007	20.4	43.2	136.3	5.4	80.1	143.2	7.8	12.8	113.2	0.8	13.7	99.9
2008-2012	14.6	35.9	111.3	1.6	43.6	89.8	6.6	13.2	105.6	0.4	13.4	89.7
	15-24			55-64			15-24			55-64		
1983-1987	1.6	20.6	147.6	3.0	48.0	113.0	0.0	5.4	79.4	1.2	19.0	96.2
1988-1992	0.4	9.8	98.8	3.6	46.7	131.5	0.0	4.3	81.0	2.0	21.3	119.1
1993-1997	0.0	9.3	82.1	2.0	37.3	92.8	0.4	5.8	111.3	0.4	12.2	76.9
1998-2002	0.6	15.8	100.1	4.2	69.7	109.6	0.0	6.0	84.9	1.4	19.5	103.4
2003-2007	0.8	18.8	108.0	5.4	74.7	129.5	0.4	9.7	107.5	1.4	17.7	105.8
2008-2012	0.6	21.5	101.2	3.6	52.0	107.0	0.2	9.4	97.0	1.0	14.8	99.5
	25-34			65-74			25-34			65-74		
1983-1987	2.0	29.0	113.5	1.2	41.1	93.8	0.8	11.3	105.1	2.0	36.2	115.8
1988-1992	1.0	20.0	104.7	1.6	38.4	109.0	0.8	10.1	116.3	1.0	22.2	84.9
1993-1997	1.0	21.1	108.0	1.6	32.9	103.0	0.2	7.6	90.9	2.0	25.3	129.9
1998-2002	1.6	30.4	112.6	2.2	43.4	95.4	0.0	9.5	81.9	2.0	24.6	116.9
2003-2007	1.2	30.9	99.7	2.6	46.1	107.5	0.0	10.5	80.8	1.8	21.7	116.5
2008-2012	2.0	38.2	117.3	1.6	36.7	93.0	0.4	13.9	96.9	2.2	22.8	127.9
	35-44			>74 y/o			35-44			>74 y/o		
1983-1987	2.6	35.4	111.2	1.4	75.0	95.8	0.6	10.8	91.6	1.4	47.9	83.0
1988-1992	2.6	30.5	128.8	1.0	53.4	82.8	0.6	9.4	97.4	2.4	54.9	114.8
1993-1997	1.6	26.0	105.9	1.6	57.2	109.9	0.0	6.3	74.2	2.0	38.4	111.5
1998-2002	2.8	45.4	128.9	2.2	61.8	117.1	0.4	9.8	94.4	1.4	26.0	83.7
2003-2007	2.6	51.0	127.2	2.4	52.8	117.3	0.6	12.8	101.3	2.8	32.7	145.7
2008-2012	2.8	45.7	122.8	2.4	45.4	109.4	1.0	15.3	113.0	1.4	17.5	90.9

Secondary Medical Zone ID: 321

	Male						Female					
	Suicide			Suicide			Suicide			Suicide		
	Num per year	Rate /100,000	×100	Num per year	Rate /100,000	×100	Num per year	Rate /100,000	×100	Num per year	Rate /100,000	×100
	Total (>10 y/o)			45–54			Total (>10 y/o)			45–54		
1983–1987	21.2	40.9	152.4	4.6	65.2	129.0	11.4	17.6	131.7	2.0	20.9	121.3
1988–1992	13.8	28.0	131.7	2.4	41.2	120.1	10.8	14.9	136.9	1.4	16.9	115.5
1993–1997	14.2	28.8	134.7	2.6	42.5	116.1	6.8	11.2	111.1	0.8	13.4	104.1
1998–2002	15.8	37.5	115.6	4.4	69.3	123.8	7.8	13.1	113.1	0.8	15.2	101.2
2003–2007	17.2	41.1	130.7	2.8	59.8	106.9	6.2	12.9	106.3	1.4	17.2	125.3
2008–2012	14.6	36.4	122.6	2.0	51.7	106.4	5.8	13.5	106.1	0.4	14.2	95.1
	15–24			55–64			15–24			55–64		
1983–1987	0.6	14.5	103.8	5.0	61.0	143.5	0.2	6.6	96.8	1.6	20.2	102.4
1988–1992	0.6	10.9	110.4	4.4	51.6	145.3	0.0	4.6	86.3	1.8	19.9	111.3
1993–1997	1.0	13.8	122.4	3.8	52.3	129.9	0.2	5.3	101.9	1.8	19.7	123.8
1998–2002	1.2	19.4	122.8	3.4	68.1	107.2	0.4	7.8	110.9	1.0	18.5	98.4
2003–2007	1.4	22.7	129.9	3.4	63.4	109.8	0.0	7.7	85.9	0.4	14.8	88.3
2008–2012	0.4	20.7	97.7	4.2	59.5	122.4	0.2	9.7	100.2	0.8	15.0	100.4
	25–34			65–74			25–34			65–74		
1983–1987	3.4	43.0	168.2	1.8	44.7	102.2	0.2	9.3	86.5	3.2	46.4	148.4
1988–1992	0.4	17.0	88.8	2.8	47.5	134.9	0.8	10.9	125.6	2.6	35.2	135.0
1993–1997	1.2	24.5	125.5	1.6	30.7	96.1	0.2	8.1	97.5	1.4	19.8	101.5
1998–2002	0.2	23.1	85.6	3.2	50.1	110.2	0.4	11.9	102.7	1.8	22.9	108.9
2003–2007	1.4	35.2	113.5	2.4	44.9	104.7	0.2	12.2	93.8	1.4	19.8	106.1
2008–2012	0.4	29.6	91.0	2.4	45.6	115.5	1.2	18.6	130.0	1.4	19.9	111.7
	35–44			>74 y/o			35–44			>74 y/o		
1983–1987	2.6	40.2	126.2	3.2	108.9	139.0	1.0	13.4	113.3	3.2	84.6	146.7
1988–1992	2.0	29.4	124.1	1.2	52.4	81.2	0.8	10.8	111.9	3.4	68.8	143.8
1993–1997	1.2	25.5	103.9	2.8	71.5	137.3	0.2	7.7	90.5	2.2	39.4	114.4
1998–2002	0.4	26.9	76.4	3.0	66.1	125.5	0.2	9.5	91.5	3.2	42.9	138.0
2003–2007	2.2	53.2	132.8	3.6	60.8	134.9	0.6	13.6	108.0	2.2	25.5	113.7
2008–2012	1.4	40.0	107.6	3.8	55.0	132.6	0.2	12.8	94.6	1.6	18.0	93.4

Secondary Medical Zone ID: 322

	Male						Female					
	Suicide			Suicide			Suicide			Suicide		
	Num per year	Rate /100,000	×100	Num per year	Rate /100,000	×100	Num per year	Rate /100,000	×100	Num per year	Rate /100,000	×100
	Total (>10 y/o)			45-54			Total (>10 y/o)			45-54		
1983-1987	19.4	35.1	116.7	4.6	55.9	110.6	6.0	13.1	73.9	0.4	12.4	72.0
1988-1992	14.4	26.1	111.3	2.4	34.0	99.0	7.0	11.8	86.9	1.2	14.8	101.2
1993-1997	18.0	28.7	129.9	3.6	41.4	113.1	5.0	9.6	78.8	0.6	11.4	87.9
1998-2002	24.6	40.4	126.4	8.2	81.0	144.8	8.0	12.2	97.1	0.6	12.9	85.4
2003-2007	25.2	43.6	133.5	6.0	72.8	130.1	6.4	11.9	91.2	0.4	11.5	83.7
2008-2012	16.4	34.2	102.2	2.2	42.7	87.8	5.4	12.0	88.5	1.0	15.4	103.0
	15-24			55-64			15-24			55-64		
1983-1987	1.0	15.2	108.8	3.6	46.5	109.4	0.2	6.0	87.4	0.4	13.2	67.0
1988-1992	0.8	10.8	108.8	3.2	38.7	109.0	0.4	5.5	105.2	1.2	16.2	90.5
1993-1997	0.6	11.2	98.9	4.4	50.8	126.2	0.4	5.6	106.8	0.6	12.4	77.9
1998-2002	0.8	15.8	100.1	5.6	75.0	118.0	0.0	5.7	80.9	1.6	19.5	103.5
2003-2007	0.6	16.6	95.0	7.4	83.0	143.8	0.2	8.0	89.0	2.2	20.0	119.5
2008-2012	0.8	21.4	100.7	5.2	56.4	116.1	0.0	7.7	79.7	0.6	12.9	86.3
	25-34			65-74			25-34			65-74		
1983-1987	2.2	27.4	107.3	2.4	51.2	117.0	1.4	13.3	123.5	1.4	27.4	87.6
1988-1992	1.8	23.1	121.0	1.4	32.0	90.8	0.4	8.0	92.3	0.8	17.9	68.8
1993-1997	1.2	20.1	103.0	2.4	35.9	112.5	0.6	8.8	105.5	1.4	18.6	95.5
1998-2002	1.4	26.7	98.9	3.4	48.4	106.4	0.2	9.8	84.8	1.8	21.4	101.9
2003-2007	2.2	34.9	112.5	3.0	45.6	106.3	0.4	11.7	90.2	1.4	18.3	98.3
2008-2012	1.0	29.5	90.6	2.6	41.8	105.8	0.2	12.3	85.6	1.4	18.4	103.4
	35-44			>74 y/o			35-44			>74 y/o		
1983-1987	4.0	41.5	130.4	1.4	63.4	81.0	1.0	11.9	100.7	1.2	37.7	65.3
1988-1992	2.4	26.2	110.5	2.2	70.6	109.4	0.4	8.3	86.4	2.6	50.7	105.9
1993-1997	2.4	28.2	115.0	3.0	75.0	144.0	0.2	6.8	79.8	1.2	24.0	69.6
1998-2002	2.2	34.8	98.7	2.8	61.5	116.7	0.6	10.1	97.9	3.2	38.9	125.3
2003-2007	3.8	54.8	136.9	2.2	43.6	96.7	0.6	12.2	96.5	1.2	15.9	70.9
2008-2012	2.0	37.5	100.8	2.6	42.4	102.3	0.6	13.1	96.5	1.6	16.8	87.2

Secondary Medical Zone ID: 323

	Male						Female					
	Suicide			Suicide			Suicide			Suicide		
	Num per year	Rate /100,000	×100	Num per year	Rate /100,000	×100	Num per year	Rate /100,000	×100	Num per year	Rate /100,000	×100
	Total (>10 y/o)			45–54			Total (>10 y/o)			45–54		
1983–1987	36.0	40.7	138.1	8.6	67.7	133.9	19.2	16.9	117.4	3.0	19.8	114.9
1988–1992	24.0	27.4	118.3	4.4	38.5	112.0	13.8	12.5	96.8	2.2	15.9	108.4
1993–1997	18.2	24.2	91.6	5.2	40.7	111.2	10.6	10.1	89.8	1.4	12.2	94.2
1998–2002	28.2	34.9	97.7	6.0	50.5	90.2	13.6	12.9	98.9	1.6	14.0	93.1
2003–2007	28.4	37.8	98.7	9.6	76.9	137.4	14.2	12.7	108.6	1.4	13.0	95.2
2008–2012	26.8	34.5	100.5	5.2	51.8	106.6	10.4	12.3	91.8	1.0	13.0	87.0
	15–24			55–64			15–24			55–64		
1983–1987	2.0	17.9	128.0	6.6	54.0	127.0	0.4	5.9	86.1	4.4	26.2	133.0
1988–1992	1.2	10.9	109.6	5.2	41.3	116.2	1.0	6.8	128.2	2.2	16.7	93.5
1993–1997	1.0	11.2	99.1	4.2	39.0	96.9	0.6	5.5	105.9	2.8	18.6	116.8
1998–2002	2.0	18.6	117.7	6.2	60.8	95.6	1.0	8.3	118.6	2.4	19.0	100.9
2003–2007	2.2	21.1	120.7	5.0	47.5	82.3	0.2	6.9	76.6	3.0	19.3	115.3
2008–2012	1.4	20.8	97.9	6.2	48.5	99.9	0.4	8.6	88.2	1.6	13.8	92.7
	25–34			65–74			25–34			65–74		
1983–1987	4.2	33.1	129.3	3.6	46.9	107.1	2.0	13.7	127.2	3.8	34.7	110.9
1988–1992	1.8	19.4	101.3	3.4	39.4	111.8	0.4	7.0	80.6	2.2	21.3	81.5
1993–1997	2.4	23.4	119.6	2.0	25.0	78.4	0.6	7.7	92.5	2.0	16.9	86.8
1998–2002	2.0	25.1	93.0	5.0	47.3	104.0	1.4	12.7	109.3	1.6	15.7	74.9
2003–2007	3.4	34.2	110.2	1.8	27.5	64.1	1.0	12.3	94.6	2.2	17.9	96.3
2008–2012	3.0	33.0	101.3	3.4	37.6	95.3	1.0	13.6	94.6	1.4	15.2	85.5
	35–44			>74 y/o			35–44			>74 y/o		
1983–1987	7.0	48.2	151.6	4.0	85.8	109.6	1.4	11.5	97.3	4.0	58.2	100.9
1988–1992	4.4	30.5	128.5	3.6	67.2	104.1	1.8	11.3	116.8	3.8	42.8	89.5
1993–1997	1.8	19.2	78.4	1.6	34.6	66.5	0.8	7.9	92.4	2.4	24.7	71.7
1998–2002	3.2	34.0	96.6	3.6	53.1	100.8	1.2	10.8	104.9	4.2	32.5	104.7
2003–2007	3.2	37.2	92.8	3.2	41.1	91.4	1.6	14.1	111.5	4.8	29.7	132.4
2008–2012	3.0	34.3	92.1	4.4	45.0	108.6	1.6	14.5	107.0	3.4	19.8	102.8

Secondary Medical Zone ID: 324

	Male						Female					
	Suicide			Suicide			Suicide			Suicide		
	Num per year	Rate /100,000	×100	Num per year	Rate /100,000	×100	Num per year	Rate /100,000	×100	Num per year	Rate /100,000	×100
	Total (>10 y/o)			45–54			Total (>10 y/o)			45–54		
1983–1987	31.4	37.9	127.9	7.2	57.1	112.9	13.4	15.0	98.1	1.8	15.2	88.4
1988–1992	23.6	27.8	123.9	5.0	41.4	120.5	12.8	13.0	101.6	1.6	14.0	95.6
1993–1997	26.2	29.9	130.7	5.6	42.4	115.9	14.8	12.2	126.5	2.6	16.0	123.7
1998–2002	35.0	40.8	122.6	9.4	68.1	121.6	13.2	12.3	103.5	1.8	14.6	96.7
2003–2007	32.4	39.7	117.2	6.6	58.6	104.8	11.2	12.5	97.4	2.6	17.0	124.3
2008–2012	26.4	34.9	106.5	4.8	50.5	104.0	11.6	12.7	102.5	1.2	13.9	93.1
	15–24			55–64			15–24			55–64		
1983–1987	1.0	12.5	89.6	6.0	53.8	126.5	0.4	5.9	86.2	2.2	18.9	95.6
1988–1992	0.6	9.0	91.0	6.4	49.1	138.3	0.4	4.9	93.4	1.8	15.8	88.4
1993–1997	1.0	11.4	100.6	5.0	43.8	108.7	0.4	5.0	95.9	2.4	17.4	109.2
1998–2002	1.6	17.2	108.6	7.6	71.4	112.3	0.2	5.7	81.8	3.0	21.6	114.5
2003–2007	0.8	15.4	88.1	7.2	62.4	108.1	0.0	6.0	66.7	2.2	17.3	103.4
2008–2012	0.8	18.4	86.8	8.0	58.3	120.0	0.4	8.7	89.8	2.8	17.6	117.8
	25–34			65–74			25–34			65–74		
1983–1987	5.4	36.2	141.5	2.4	43.9	100.3	1.6	11.3	105.2	1.8	26.4	84.3
1988–1992	1.6	17.5	91.4	2.6	38.1	108.0	0.8	8.1	93.8	3.8	35.4	135.9
1993–1997	2.4	23.4	119.7	3.2	36.1	113.3	0.8	8.4	100.6	3.4	26.3	134.9
1998–2002	2.6	28.6	105.8	5.4	51.6	113.4	0.4	9.4	81.1	2.4	20.2	96.2
2003–2007	2.6	31.4	101.1	5.4	51.4	119.8	1.0	12.6	97.1	2.8	21.0	113.0
2008–2012	1.4	27.3	83.9	3.6	40.4	102.2	1.2	14.8	103.3	2.2	18.8	105.8
	35–44			>74 y/o			35–44			>74 y/o		
1983–1987	5.8	39.8	125.2	3.2	94.8	121.0	3.0	16.2	136.9	2.6	55.0	95.3
1988–1992	4.2	28.4	119.7	3.0	74.8	116.0	1.8	11.0	113.5	2.6	41.7	87.1
1993–1997	5.8	39.9	162.9	3.2	66.3	127.3	1.2	9.0	106.4	4.0	46.0	133.7
1998–2002	5.2	46.0	130.6	3.2	57.7	109.4	0.6	8.6	83.3	4.6	41.9	134.8
2003–2007	5.2	53.4	133.4	4.6	58.1	129.0	1.0	12.1	96.1	1.6	14.8	65.8
2008–2012	4.0	41.7	112.2	3.8	42.7	102.9	0.4	10.9	80.6	3.2	20.3	105.5

Secondary Medical Zone ID: 325

	Male						Female					
	Suicide			Suicide			Suicide			Suicide		
	Num per year	Rate /100,000	×100	Num per year	Rate /100,000	×100	Num per year	Rate /100,000	×100	Num per year	Rate /100,000	×100
	Total (>10 y/o)			45–54			Total (>10 y/o)			45–54		
1983–1987	14.2	33.6	107.2	3.8	55.0	108.8	10.4	17.0	124.4	2.4	22.8	132.2
1988–1992	14.0	27.6	126.2	2.0	33.4	97.4	7.2	12.7	101.9	1.0	14.6	100.0
1993–1997	15.2	28.3	127.4	2.6	37.2	101.6	5.6	10.5	94.1	0.8	12.3	95.5
1998–2002	22.0	39.8	127.1	5.0	62.0	110.8	8.4	13.1	109.2	2.0	18.2	120.7
2003–2007	21.4	42.6	125.7	6.0	75.8	135.5	8.4	13.2	112.5	1.2	14.6	106.8
2008–2012	20.0	37.8	123.1	4.4	60.8	125.0	7.4	13.5	105.6	0.8	14.5	97.0
	15–24			55–64			15–24			55–64		
1983–1987	1.2	17.1	122.7	2.6	45.6	107.4	0.2	6.1	89.4	1.2	19.4	98.2
1988–1992	0.6	10.3	104.0	1.6	30.7	86.4	0.2	5.0	93.9	2.6	24.5	137.3
1993–1997	1.0	13.0	115.0	1.8	35.1	87.1	0.4	5.7	109.1	1.0	15.4	97.1
1998–2002	2.2	23.1	146.2	3.6	61.9	97.4	0.4	7.3	103.2	0.6	16.0	85.1
2003–2007	0.6	16.9	97.1	3.0	51.0	88.3	0.2	8.1	90.4	1.0	15.9	95.1
2008–2012	0.4	19.1	90.1	5.8	61.4	126.4	0.6	11.2	115.7	0.8	13.7	91.9
	25–34			65–74			25–34			65–74		
1983–1987	2.8	31.8	124.3	0.6	34.5	78.9	1.2	12.6	116.7	1.8	36.2	115.8
1988–1992	1.2	19.9	103.9	2.4	48.5	137.7	1.0	10.5	121.0	1.2	25.0	95.8
1993–1997	1.2	20.9	107.0	2.8	45.7	143.2	0.8	9.6	115.7	1.2	19.8	101.6
1998–2002	2.4	33.1	122.5	3.8	56.7	124.6	0.8	12.2	105.7	1.4	21.2	100.7
2003–2007	2.6	38.0	122.6	3.2	50.1	116.7	1.2	15.3	117.3	1.8	21.8	117.1
2008–2012	1.2	30.6	93.9	2.2	40.2	101.7	0.4	13.0	91.0	1.6	20.0	112.2
	35–44			>74 y/o			35–44			>74 y/o		
1983–1987	1.6	27.3	85.9	1.6	85.0	108.5	2.0	16.0	135.4	1.6	57.8	100.1
1988–1992	3.6	34.7	146.5	2.6	93.2	144.5	0.2	7.8	80.8	1.0	33.5	69.9
1993–1997	3.4	36.1	147.4	2.4	75.0	144.1	0.4	7.6	89.9	1.0	25.6	74.4
1998–2002	2.2	36.0	102.3	2.8	72.3	137.2	0.8	11.0	106.3	2.4	37.4	120.4
2003–2007	4.2	60.2	150.2	1.8	45.2	100.3	0.6	12.2	96.6	2.4	29.2	129.9
2008–2012	3.0	43.3	116.4	3.0	50.8	122.4	1.4	16.1	118.6	1.8	20.3	105.4

Secondary Medical Zone ID: 326

	Male						Female					
	Suicide			Suicide			Suicide			Suicide		
	Num per year	Rate /100,000	×100	Num per year	Rate /100,000	×100	Num per year	Rate /100,000	×100	Num per year	Rate /100,000	×100
	Total (>10 y/o)			45–54			Total (>10 y/o)			45–54		
1983–1987	51.0	35.6	111.7	14.6	64.2	126.9	24.6	14.9	97.2	2.2	12.1	70.1
1988–1992	41.0	25.9	106.8	8.8	36.5	106.3	21.2	11.6	87.1	5.8	19.0	130.2
1993–1997	48.2	26.8	110.0	13.4	44.0	120.4	20.4	9.8	88.8	5.4	15.7	121.8
1998–2002	81.8	42.0	120.8	22.8	69.7	124.6	27.8	12.3	96.4	4.6	14.2	94.3
2003–2007	83.8	41.2	119.3	19.4	66.0	117.9	30.8	13.0	105.2	4.4	13.9	101.6
2008–2012	71.2	35.7	108.0	12.8	49.5	101.8	24.2	11.9	85.9	2.6	11.2	75.0
	15–24			55–64			15–24			55–64		
1983–1987	2.4	11.7	83.6	7.6	44.9	105.7	3.8	11.2	164.4	3.6	18.6	94.1
1988–1992	2.6	9.8	99.4	7.4	36.5	102.7	1.2	4.6	87.6	3.0	14.7	82.4
1993–1997	3.0	10.6	93.8	9.8	44.2	109.9	0.8	3.7	70.4	4.2	16.6	104.7
1998–2002	5.4	18.4	116.5	16.8	70.5	111.0	2.2	7.5	106.6	5.0	19.0	100.9
2003–2007	5.8	21.8	125.1	20.8	71.5	123.8	2.4	9.4	104.7	4.8	16.0	95.8
2008–2012	4.4	21.3	100.4	16.0	51.0	104.9	3.2	12.7	130.8	3.2	11.5	77.2
	25–34			65–74			25–34			65–74		
1983–1987	9.0	31.1	121.8	2.0	29.3	66.9	3.4	11.1	103.5	3.6	29.0	92.9
1988–1992	7.2	26.8	140.4	3.8	34.3	97.3	2.0	7.8	90.1	2.6	18.8	72.2
1993–1997	3.8	16.7	85.6	4.4	30.1	94.2	1.2	6.0	72.1	2.6	15.0	76.9
1998–2002	9.0	31.8	117.9	6.8	39.5	86.9	3.8	12.4	107.0	6.4	26.3	125.0
2003–2007	6.4	25.7	82.8	11.0	52.8	123.0	2.8	10.8	83.4	4.8	19.7	105.5
2008–2012	8.8	35.5	108.9	9.6	43.9	111.1	4.2	15.4	107.2	4.2	17.3	97.2
	35–44			>74 y/o			35–44			>74 y/o		
1983–1987	10.4	35.3	110.8	5.0	94.4	120.5	4.0	12.5	105.6	3.8	50.0	86.7
1988–1992	9.0	27.6	116.5	2.2	41.7	64.7	2.6	8.5	88.0	4.0	36.4	76.0
1993–1997	9.0	29.3	119.4	4.8	59.0	113.3	1.6	6.3	74.0	4.6	31.5	91.6
1998–2002	14.8	51.3	145.5	6.2	59.9	113.5	2.2	8.6	82.9	3.6	20.7	66.6
2003–2007	11.8	45.3	113.0	8.2	57.0	126.6	4.8	15.2	120.7	6.8	27.3	121.8
2008–2012	10.2	37.6	101.1	9.4	51.5	124.2	2.4	10.3	76.0	4.4	15.7	81.6

Secondary Medical Zone ID: 327

	Male						Female					
	Suicide			Suicide			Suicide			Suicide		
	Num per year	Rate /100,000	×100	Num per year	Rate /100,000	×100	Num per year	Rate /100,000	×100	Num per year	Rate /100,000	×100
	Total (>10 y/o)			45-54			Total (>10 y/o)			45-54		
1983-1987	21.2	37.7	129.9	5.8	67.6	133.6	10.8	16.3	113.8	2.4	21.9	127.3
1988-1992	14.8	26.5	115.5	3.4	41.5	120.9	8.2	12.5	99.4	0.8	13.5	92.1
1993-1997	19.4	30.0	136.7	5.4	50.8	138.9	8.0	11.0	106.5	1.2	13.4	103.6
1998-2002	23.2	38.8	116.5	5.0	56.6	101.1	7.0	11.7	89.3	1.0	13.9	92.4
2003-2007	25.4	41.9	127.2	7.0	77.7	138.9	8.0	12.3	101.1	0.8	12.7	92.4
2008-2012	23.2	37.6	124.7	3.4	50.9	104.7	8.8	13.5	108.3	0.8	14.1	94.7
	15-24			55-64			15-24			55-64		
1983-1987	1.4	16.8	120.1	4.2	54.9	129.3	1.2	9.6	139.9	1.8	21.2	107.4
1988-1992	0.0	7.2	72.9	3.0	39.0	109.8	0.2	4.7	88.9	2.0	20.5	115.0
1993-1997	0.6	10.3	90.7	4.0	48.5	120.6	0.0	4.0	75.8	1.2	15.8	99.1
1998-2002	1.6	17.9	113.2	4.4	63.7	100.3	0.2	6.1	87.3	1.8	20.3	108.0
2003-2007	1.0	17.1	97.9	5.0	61.2	106.1	0.0	6.6	73.0	1.0	15.3	91.5
2008-2012	0.8	19.9	94.0	6.6	61.8	127.2	0.6	10.5	108.6	1.4	15.1	101.4
	25-34			65-74			25-34			65-74		
1983-1987	2.6	29.2	114.2	1.2	37.3	85.2	0.6	9.6	89.2	2.0	33.5	107.2
1988-1992	2.2	25.9	135.6	2.8	48.2	136.7	0.4	8.0	91.8	1.8	27.2	104.2
1993-1997	1.6	22.8	116.7	2.8	41.7	130.9	0.4	8.0	95.8	2.2	25.0	128.5
1998-2002	1.4	25.9	96.1	4.0	54.2	119.2	0.0	8.9	76.7	1.4	19.8	94.5
2003-2007	3.6	42.0	135.6	3.0	45.1	105.2	0.2	10.5	81.0	2.0	21.9	117.4
2008-2012	2.2	34.7	106.5	2.6	40.5	102.6	1.4	17.1	119.1	1.4	18.3	103.0
	35-44			>74 y/o			35-44			>74 y/o		
1983-1987	4.2	41.8	131.2	1.8	81.1	103.6	0.6	10.3	87.4	2.2	62.0	107.4
1988-1992	2.0	23.2	98.1	1.4	57.6	89.3	1.2	10.8	111.5	1.8	41.2	86.1
1993-1997	3.6	35.5	144.8	1.4	47.4	91.0	0.4	7.4	87.3	2.6	41.1	119.5
1998-2002	3.8	46.4	131.7	3.0	67.4	127.8	0.6	9.9	96.0	2.0	28.5	91.7
2003-2007	2.6	42.8	106.9	3.2	57.1	126.8	1.2	14.4	114.3	2.8	28.9	129.0
2008-2012	3.0	42.1	113.3	4.4	60.0	144.7	1.0	14.3	105.6	2.2	21.0	109.2

Secondary Medical Zone ID: 328

	Male						Female					
	Suicide			Suicide			Suicide			Suicide		
	Num per year	Rate /100,000	×100	Num per year	Rate /100,000	×100	Num per year	Rate /100,000	×100	Num per year	Rate /100,000	×100
	Total (>10 y/o)			45–54			Total (>10 y/o)			45–54		
1983–1987	18.6	37.2	130.4	4.6	62.0	122.6	7.8	15.0	97.4	3.0	25.0	144.9
1988–1992	15.2	29.0	135.7	3.0	42.8	124.5	9.6	14.1	119.9	1.6	17.1	117.0
1993–1997	18.2	30.7	153.2	3.2	43.3	118.4	7.8	11.8	115.3	1.0	13.7	105.8
1998–2002	21.2	40.3	132.3	4.8	65.3	116.7	8.6	13.1	113.0	1.4	16.7	111.1
2003–2007	20.0	43.7	130.9	5.4	76.1	136.1	7.0	13.2	105.6	1.4	16.1	117.2
2008–2012	18.8	38.0	131.0	3.4	57.3	117.8	6.2	13.2	102.5	0.2	12.3	82.4
	15–24			55–64			15–24			55–64		
1983–1987	0.8	15.1	108.2	4.0	55.3	130.0	0.0	5.5	80.9	0.8	16.3	82.6
1988–1992	1.0	12.3	123.7	4.0	49.1	138.4	0.2	5.1	96.2	1.2	17.0	95.3
1993–1997	1.4	15.1	133.8	2.6	41.5	103.2	0.4	5.8	111.4	2.2	21.2	133.3
1998–2002	0.2	13.5	85.7	6.0	86.9	136.7	0.2	6.6	94.3	2.4	23.9	127.1
2003–2007	0.8	18.4	105.7	3.4	58.3	101.1	0.4	9.3	103.7	1.8	19.7	117.6
2008–2012	0.0	17.3	81.5	4.0	54.2	111.7	0.6	11.5	118.8	1.2	15.8	105.8
	25–34			65–74			25–34			65–74		
1983–1987	2.8	33.3	130.4	1.6	44.0	100.6	0.6	10.2	95.0	1.2	27.8	89.0
1988–1992	1.6	24.0	125.7	1.4	34.7	98.4	0.8	10.0	115.9	2.2	32.0	122.8
1993–1997	1.8	26.6	136.4	4.2	57.5	180.3	0.2	7.7	91.9	1.2	18.7	95.8
1998–2002	1.4	29.5	109.3	4.4	60.5	133.1	0.0	9.7	83.6	2.0	23.9	113.7
2003–2007	1.6	34.5	111.2	2.2	41.7	97.3	0.2	11.6	89.2	0.6	14.9	80.1
2008–2012	1.2	32.9	101.1	4.6	59.8	151.4	0.4	13.8	96.6	1.6	20.3	113.9
	35–44			>74 y/o			35–44			>74 y/o		
1983–1987	2.6	36.0	113.2	2.2	91.4	116.8	0.6	11.1	94.1	1.6	50.3	87.2
1988–1992	2.2	27.8	117.2	2.0	72.8	112.9	0.4	8.8	91.2	3.2	65.2	136.2
1993–1997	2.0	28.3	115.4	3.0	81.1	155.7	1.0	10.5	123.6	1.8	34.0	98.7
1998–2002	1.4	32.2	91.5	3.0	71.3	135.2	0.4	9.7	93.8	2.2	33.0	106.1
2003–2007	4.2	68.7	171.5	2.4	50.5	112.1	1.0	14.7	116.4	1.6	20.9	93.1
2008–2012	2.4	43.9	118.1	3.2	52.2	125.7	0.4	13.2	97.0	1.8	19.4	101.0

Secondary Medical Zone ID: 329

	Male						Female					
	Suicide			Suicide			Suicide			Suicide		
	Num per year	Rate /100,000	×100	Num per year	Rate /100,000	×100	Num per year	Rate /100,000	×100	Num per year	Rate /100,000	×100
	Total (>10 y/o)			45-54			Total (>10 y/o)			45-54		
1983-1987	40.2	43.7	154.5	10.8	78.3	154.7	20.8	18.1	132.0	4.4	24.5	142.0
1988-1992	34.2	34.0	160.3	7.2	51.7	150.5	19.6	15.1	132.7	1.2	12.5	85.8
1993-1997	36.4	33.8	157.4	7.2	47.1	128.8	20.0	13.8	147.5	5.4	24.0	186.2
1998-2002	49.2	44.8	146.1	10.8	68.3	122.0	19.2	13.7	123.7	2.6	16.1	106.9
2003-2007	53.8	48.8	158.2	12.2	81.3	145.3	18.8	13.6	124.7	2.0	13.8	100.6
2008-2012	39.6	38.2	125.5	9.0	65.9	135.6	17.8	12.9	119.1	1.8	14.6	97.7
	15-24			55-64			15-24			55-64		
1983-1987	3.2	23.9	171.7	7.2	59.5	139.9	0.8	7.0	103.2	4.8	28.8	145.9
1988-1992	1.6	11.8	118.7	7.0	51.0	143.7	1.4	7.8	147.8	4.6	25.5	142.7
1993-1997	2.2	13.9	123.1	7.4	54.2	134.7	0.4	4.6	88.9	2.6	17.6	110.5
1998-2002	0.8	12.1	76.8	14.0	102.9	161.9	0.2	5.3	75.2	4.2	24.8	132.0
2003-2007	1.6	16.9	96.9	10.8	75.3	130.5	0.4	6.9	77.2	3.8	21.3	127.0
2008-2012	1.4	19.0	89.6	9.6	58.9	121.2	0.4	7.6	78.7	2.6	15.8	106.1
	25-34			65-74			25-34			65-74		
1983-1987	3.0	24.3	95.0	5.0	64.3	146.9	1.8	11.8	109.8	3.2	34.2	109.4
1988-1992	3.8	28.3	148.0	3.6	42.8	121.4	1.2	9.1	105.5	5.2	41.3	158.2
1993-1997	2.8	24.2	123.7	4.0	39.3	123.3	1.0	8.6	103.2	4.0	27.6	141.4
1998-2002	3.4	30.4	112.7	7.8	62.9	138.2	0.4	8.8	75.9	4.8	29.8	141.7
2003-2007	5.8	43.7	140.9	8.8	68.0	158.6	1.4	12.8	98.5	4.8	28.0	150.5
2008-2012	2.6	29.1	89.2	7.0	55.4	140.3	1.2	13.0	90.9	4.0	24.4	137.0
	35-44			>74 y/o			35-44			>74 y/o		
1983-1987	7.2	46.1	145.0	3.6	97.7	124.7	1.6	11.8	100.1	4.2	73.4	127.2
1988-1992	6.0	34.8	147.0	4.8	98.1	152.0	1.0	8.5	88.1	5.0	62.7	131.0
1993-1997	6.8	41.0	167.1	6.0	98.2	188.5	1.0	7.8	92.3	5.6	54.1	157.1
1998-2002	5.8	44.1	125.3	6.6	89.1	169.0	1.6	11.3	109.0	5.4	42.2	135.8
2003-2007	7.0	58.6	146.4	7.6	79.0	175.4	1.2	11.8	93.3	5.2	32.3	143.7
2008-2012	3.4	35.1	94.3	6.6	59.3	143.0	0.8	11.3	83.0	7.0	34.1	177.5

Secondary Medical Zone ID: 330

	Male						Female					
	Suicide			Suicide			Suicide			Suicide		
	Num per year	Rate /100,000	×100	Num per year	Rate /100,000	×100	Num per year	Rate /100,000	×100	Num per year	Rate /100,000	×100
	Total (>10 y/o)			45–54			Total (>10 y/o)			45–54		
1983–1987	25.6	45.6	177.9	6.8	82.9	163.8	18.8	22.7	193.5	2.2	21.8	126.5
1988–1992	25.2	37.2	210.6	5.0	60.2	175.4	12.8	16.0	153.2	1.4	16.5	113.2
1993–1997	26.6	37.3	209.8	3.2	43.7	119.5	14.2	14.6	182.0	1.4	15.3	118.7
1998–2002	29.2	47.0	170.7	6.0	73.3	131.1	15.2	16.0	167.7	2.0	19.0	126.3
2003–2007	31.0	49.3	188.9	5.6	76.9	137.5	17.0	16.3	187.3	1.4	16.0	116.6
2008–2012	28.8	44.0	178.7	5.8	76.9	158.2	15.4	16.2	169.3	1.6	18.7	125.5
	15–24			55–64			15–24			55–64		
1983–1987	1.2	18.4	131.8	4.8	63.2	148.7	0.4	7.1	104.2	4.6	37.2	188.7
1988–1992	0.8	11.5	116.0	4.2	50.9	143.4	0.2	5.2	97.8	2.2	22.1	123.9
1993–1997	1.0	13.4	118.2	4.6	56.8	141.2	0.0	4.4	83.7	3.6	28.8	181.3
1998–2002	0.6	15.3	96.9	5.4	82.9	130.4	0.2	6.7	95.1	2.0	22.4	119.1
2003–2007	1.4	21.4	122.7	6.4	85.2	147.7	0.0	7.2	80.6	2.0	20.5	122.6
2008–2012	0.4	19.8	93.4	6.2	66.6	137.1	0.8	12.9	132.8	1.4	16.4	110.1
	25–34			65–74			25–34			65–74		
1983–1987	2.4	31.5	123.2	2.0	49.8	113.7	1.6	15.1	140.0	2.4	39.8	127.4
1988–1992	2.0	27.4	143.5	3.8	61.3	173.9	0.4	8.4	96.9	4.2	50.1	192.2
1993–1997	1.6	25.3	129.6	5.4	69.2	216.8	0.4	8.5	101.7	3.0	31.9	163.8
1998–2002	2.2	34.8	129.0	6.0	73.1	160.7	0.4	11.3	97.8	4.4	38.9	185.4
2003–2007	0.6	26.8	86.4	5.8	73.1	170.5	1.4	17.3	133.2	4.4	35.7	191.5
2008–2012	1.6	35.4	108.5	5.0	63.7	161.1	1.0	16.6	115.7	2.6	25.0	140.4
	35–44			>74 y/o			35–44			>74 y/o		
1983–1987	5.2	56.6	178.0	3.0	115.2	147.1	2.4	18.6	157.3	5.2	133.1	230.6
1988–1992	4.2	40.8	172.3	5.0	136.4	211.5	0.8	10.2	106.0	3.6	75.6	158.1
1993–1997	4.4	45.5	185.7	6.4	142.8	274.3	1.0	10.5	123.4	4.8	74.7	217.0
1998–2002	4.4	57.8	164.0	4.6	93.5	177.4	1.0	12.3	119.3	5.0	61.9	199.4
2003–2007	4.0	66.2	165.1	7.0	105.0	233.1	1.0	14.6	115.4	6.8	64.1	285.6
2008–2012	2.6	45.3	121.6	7.2	89.1	214.7	0.6	13.9	102.6	7.4	53.8	279.8

Secondary Medical Zone ID: 331

	Male						Female					
	Suicide			Suicide			Suicide			Suicide		
	Num per year	Rate /100,000	×100	Num per year	Rate /100,000	×100	Num per year	Rate /100,000	×100	Num per year	Rate /100,000	×100
	Total (>10 y/o)			45–54			Total (>10 y/o)			45–54		
1983–1987	88.8	35.4	110.9	20.6	55.7	110.2	43.8	14.6	94.3	7.8	18.1	104.9
1988–1992	74.2	27.4	113.1	19.0	46.8	136.4	41.0	12.3	93.5	6.2	14.5	99.2
1993–1997	81.0	28.1	112.8	21.2	44.5	121.7	35.8	10.2	90.2	6.4	13.2	101.8
1998–2002	113.2	37.6	104.5	31.0	61.1	109.1	37.8	10.4	79.9	6.0	12.4	82.2
2003–2007	109.0	35.7	97.6	27.8	58.9	105.3	36.2	10.8	78.1	6.8	13.4	98.1
2008–2012	93.2	31.3	90.0	17.4	43.4	89.2	36.4	10.9	79.2	6.2	13.8	92.2
	15–24			55–64			15–24			55–64		
1983–1987	5.4	12.5	89.4	17.4	55.1	129.7	2.8	6.1	88.7	7.8	20.1	101.8
1988–1992	4.2	9.2	92.9	12.2	35.3	99.5	2.4	4.8	90.7	10.4	23.3	130.2
1993–1997	5.2	10.7	94.9	13.4	38.4	95.5	3.2	5.7	109.5	6.0	14.7	92.7
1998–2002	3.8	9.9	62.4	25.2	67.2	105.8	2.6	5.6	80.4	8.8	20.3	107.9
2003–2007	5.2	13.5	77.3	24.8	56.6	98.0	3.4	7.9	87.7	6.0	13.9	82.9
2008–2012	5.0	15.8	74.7	24.4	49.2	101.2	1.4	5.3	54.2	7.2	14.1	94.2
	25–34			65–74			25–34			65–74		
1983–1987	13.0	27.2	106.5	6.8	39.3	89.8	4.2	8.7	80.7	7.8	31.1	99.5
1988–1992	9.4	22.5	117.7	7.2	34.2	97.1	4.2	8.8	101.9	6.4	21.6	82.9
1993–1997	11.6	28.1	144.1	8.0	30.6	95.8	2.8	6.8	81.9	6.6	18.6	95.2
1998–2002	11.6	28.2	104.3	13.4	44.2	97.2	3.2	8.2	70.6	7.6	19.8	94.0
2003–2007	12.8	30.7	99.1	14.0	44.2	103.0	4.6	10.5	80.9	5.6	15.2	81.5
2008–2012	12.8	33.1	101.7	11.6	36.0	91.0	5.0	12.1	84.4	4.6	13.5	75.7
	35–44			>74 y/o			35–44			>74 y/o		
1983–1987	19.0	39.0	122.4	5.8	65.8	84.0	8.0	15.0	126.8	5.4	37.0	64.1
1988–1992	14.6	27.9	117.9	7.4	62.1	96.3	5.6	10.2	105.7	5.4	27.2	56.8
1993–1997	15.6	31.6	128.9	5.8	42.5	81.5	5.2	9.7	114.4	5.4	21.3	61.8
1998–2002	20.2	46.1	130.9	8.0	46.3	87.9	2.4	6.5	62.7	7.0	21.3	68.6
2003–2007	14.8	37.8	94.4	9.6	42.3	94.0	4.8	11.2	88.8	4.8	12.2	54.2
2008–2012	11.8	30.8	82.9	10.2	37.2	89.5	5.6	12.6	93.1	6.0	12.7	65.9

Secondary Medical Zone ID: 332

	Male						Female					
	Suicide			Suicide			Suicide			Suicide		
	Num per year	Rate /100,000	×100	Num per year	Rate /100,000	×100	Num per year	Rate /100,000	×100	Num per year	Rate /100,000	×100
	Total (>10 y/o)			45–54			Total (>10 y/o)			45–54		
1983–1987	33.8	38.8	131.8	6.6	56.6	111.9	21.4	17.5	120.9	4.2	24.2	140.5
1988–1992	28.8	30.7	141.1	6.6	53.3	155.3	15.8	13.0	102.0	2.0	15.8	107.9
1993–1997	31.4	32.7	150.3	7.6	55.4	151.4	16.6	12.1	122.5	2.0	14.9	115.3
1998–2002	32.4	39.1	115.2	6.8	56.4	100.8	14.4	12.5	101.9	1.2	13.3	88.7
2003–2007	34.4	41.1	125.7	8.8	71.9	128.5	12.8	12.4	102.9	1.8	14.7	107.2
2008–2012	28.8	37.2	117.6	6.4	61.7	127.0	10.2	12.6	94.7	0.8	12.6	84.4
	15–24			55–64			15–24			55–64		
1983–1987	0.8	12.2	87.7	7.2	56.0	131.8	0.6	6.8	100.1	3.4	20.7	105.0
1988–1992	0.6	9.5	96.2	4.8	38.7	108.9	0.6	5.8	109.1	2.6	17.3	96.9
1993–1997	1.0	12.0	106.2	5.6	46.6	115.8	0.2	4.4	84.1	3.8	22.4	140.7
1998–2002	1.2	16.1	102.1	7.2	68.9	108.5	0.4	6.4	91.0	2.0	18.3	97.1
2003–2007	0.8	16.0	91.5	7.2	64.4	111.5	0.6	8.7	96.9	2.0	17.1	102.3
2008–2012	1.4	22.9	107.9	6.6	51.4	105.8	1.2	12.8	131.5	1.2	13.2	88.4
	25–34			65–74			25–34			65–74		
1983–1987	5.4	38.7	151.4	5.2	57.8	132.0	1.4	11.6	107.4	4.0	32.4	103.8
1988–1992	1.2	16.7	87.5	4.6	46.0	130.6	1.0	9.4	108.3	4.4	30.4	116.5
1993–1997	3.2	30.9	158.1	4.4	39.8	124.8	0.4	7.5	89.8	5.0	28.9	148.2
1998–2002	1.6	26.6	98.6	5.8	51.2	112.5	1.4	13.8	119.2	4.0	24.8	117.9
2003–2007	2.2	32.8	105.7	6.4	56.0	130.6	0.0	9.4	71.9	3.6	23.5	126.2
2008–2012	1.0	27.3	83.8	4.6	45.4	114.9	0.2	11.4	79.3	2.4	19.6	110.2
	35–44			>74 y/o			35–44			>74 y/o		
1983–1987	5.2	42.0	131.9	3.4	77.1	98.5	3.2	18.4	155.7	4.6	57.2	99.2
1988–1992	6.0	39.0	164.7	5.0	84.1	130.4	1.0	9.3	96.8	4.2	41.3	86.3
1993–1997	4.6	34.4	140.4	4.8	70.8	136.0	1.0	8.8	102.9	4.2	33.4	97.1
1998–2002	5.2	48.4	137.6	4.6	61.0	115.8	0.4	8.2	79.1	5.0	32.7	105.5
2003–2007	3.4	44.3	110.6	5.6	59.0	131.1	0.8	11.9	94.3	4.0	22.6	100.8
2008–2012	3.0	40.6	109.2	5.6	52.5	126.5	0.8	13.1	96.3	3.6	18.9	98.4

Secondary Medical Zone ID: 333

	Male						Female					
	Suicide			Suicide			Suicide			Suicide		
	Num per year	Rate /100,000	×100	Num per year	Rate /100,000	×100	Num per year	Rate /100,000	×100	Num per year	Rate /100,000	×100
	Total (>10 y/o)			45–54			Total (>10 y/o)			45–54		
1983–1987	28.4	41.4	142.3	9.6	92.8	183.5	14.8	16.7	117.3	2.2	19.7	114.4
1988–1992	20.8	29.0	132.3	3.8	43.3	125.9	10.2	12.5	95.3	1.0	14.0	95.5
1993–1997	24.6	31.9	147.6	5.2	48.9	133.8	11.8	12.1	118.9	2.0	16.4	126.8
1998–2002	25.8	39.0	114.3	7.0	66.5	118.9	12.0	13.5	109.8	2.0	17.4	115.7
2003–2007	29.0	42.2	128.9	4.8	54.5	97.5	7.4	11.5	86.8	1.0	13.0	95.0
2008–2012	23.0	36.0	112.4	3.4	46.2	95.0	9.8	12.9	105.4	1.6	16.8	112.5
	15–24			55–64			15–24			55–64		
1983–1987	1.2	15.4	110.4	5.8	57.5	135.2	0.4	6.5	95.7	3.0	23.4	118.7
1988–1992	0.4	9.0	91.1	3.2	36.3	102.3	0.6	6.2	116.7	1.8	17.4	97.2
1993–1997	1.0	12.1	107.4	5.8	57.5	142.9	0.2	4.7	89.8	3.4	24.5	154.1
1998–2002	1.0	15.4	97.6	5.4	69.7	109.7	0.6	7.5	106.5	2.4	22.1	117.6
2003–2007	1.2	18.1	104.0	8.0	81.9	141.8	0.8	10.2	113.3	0.6	13.9	82.8
2008–2012	1.4	22.8	107.7	6.4	57.9	119.2	0.2	8.1	83.1	2.0	17.0	114.1
	25–34			65–74			25–34			65–74		
1983–1987	3.8	34.3	134.3	2.4	42.6	97.4	1.2	11.8	109.6	2.6	31.4	100.5
1988–1992	1.0	16.1	84.3	3.6	46.7	132.5	0.6	8.3	95.9	4.0	36.7	140.6
1993–1997	1.8	22.1	113.3	3.4	40.0	125.3	1.0	9.8	117.7	1.8	18.2	93.2
1998–2002	1.6	25.9	95.9	3.2	43.2	95.0	0.2	9.3	80.6	3.2	26.2	124.5
2003–2007	3.2	38.3	123.4	4.2	51.6	120.2	0.4	11.0	84.8	1.4	17.1	92.1
2008–2012	2.6	35.7	109.5	3.0	42.3	107.1	0.6	13.1	91.4	2.2	21.2	119.1
	35–44			>74 y/o			35–44			>74 y/o		
1983–1987	2.8	32.8	103.1	2.8	81.5	104.1	0.6	10.5	88.7	4.8	81.3	140.8
1988–1992	5.4	41.2	174.0	3.4	77.5	120.2	0.8	9.5	98.0	1.4	25.8	53.9
1993–1997	4.8	40.4	164.6	2.2	49.1	94.3	0.6	8.0	93.6	2.8	32.7	94.9
1998–2002	4.0	43.6	123.7	3.6	61.0	115.8	1.4	12.7	122.5	2.2	23.2	74.8
2003–2007	4.4	53.6	133.7	3.2	47.5	105.6	0.2	9.9	78.2	3.0	23.7	105.4
2008–2012	2.2	36.0	96.7	4.0	49.5	119.3	0.4	11.7	85.9	2.8	20.0	104.2

Secondary Medical Zone ID: 334

	Male						Female					
	Suicide			Suicide			Suicide			Suicide		
	Num per year	Rate /100,000	×100	Num per year	Rate /100,000	×100	Num per year	Rate /100,000	×100	Num per year	Rate /100,000	×100
	Total (>10 y/o)			45-54			Total (>10 y/o)			45-54		
1983-1987	18.6	37.5	128.5	5.2	67.2	132.8	11.0	16.9	119.2	2.2	21.4	124.1
1988-1992	12.0	25.9	108.3	4.2	52.0	151.5	8.4	12.8	104.7	1.0	14.8	101.4
1993-1997	11.8	25.5	104.0	2.6	38.7	105.8	7.4	11.1	106.2	0.6	12.1	93.4
1998-2002	17.6	37.1	107.0	6.8	78.2	139.7	7.4	12.4	98.1	1.0	15.0	99.5
2003-2007	19.8	39.8	119.5	4.4	63.4	113.4	7.4	12.7	103.3	0.8	13.3	97.3
2008-2012	18.4	37.3	119.7	3.4	54.1	111.2	6.2	12.4	97.5	0.6	13.8	92.4
	15-24			55-64			15-24			55-64		
1983-1987	0.4	12.2	87.6	3.0	45.7	107.5	0.4	7.0	102.6	0.8	15.8	80.0
1988-1992	0.4	9.7	98.2	1.8	30.9	87.1	0.0	4.2	79.7	2.6	23.1	129.6
1993-1997	0.6	11.6	102.8	2.2	37.8	93.8	0.0	4.2	80.3	1.6	18.0	113.0
1998-2002	0.6	15.3	96.8	2.2	50.7	79.9	0.4	7.2	103.1	0.8	16.9	90.0
2003-2007	0.6	16.9	97.0	3.8	59.9	103.8	0.4	9.0	100.3	1.2	17.2	102.6
2008-2012	0.8	21.6	101.9	4.4	55.0	113.1	0.0	7.6	78.0	0.6	13.3	89.4
	25-34			65-74			25-34			65-74		
1983-1987	2.4	31.2	122.2	2.0	47.2	107.8	0.8	11.2	104.0	3.0	42.5	135.9
1988-1992	0.4	14.6	76.6	1.8	37.6	106.6	0.4	8.3	95.7	0.8	18.5	71.1
1993-1997	0.0	13.4	68.4	1.4	28.9	90.5	0.8	9.8	117.7	1.8	21.9	112.5
1998-2002	0.6	22.8	84.6	3.2	48.9	107.6	0.2	10.1	86.8	2.6	26.7	127.2
2003-2007	1.8	33.5	108.0	3.0	47.0	109.7	1.0	14.7	113.2	1.0	16.8	90.2
2008-2012	1.0	30.6	93.9	2.4	42.1	106.5	0.6	14.3	99.7	1.4	19.1	107.3
	35-44			>74 y/o			35-44			>74 y/o		
1983-1987	4.0	46.6	146.5	1.6	74.2	94.7	1.0	12.9	108.9	2.8	71.2	123.5
1988-1992	1.6	23.7	100.1	1.6	60.6	93.9	0.8	10.2	105.4	2.8	54.8	114.6
1993-1997	2.4	30.4	123.9	2.4	65.6	125.9	0.8	9.5	112.0	1.8	31.7	92.2
1998-2002	2.0	35.1	99.6	2.0	53.6	101.6	0.6	10.3	100.1	1.8	26.8	86.3
2003-2007	2.8	47.8	119.2	3.4	60.4	134.0	0.6	12.4	98.0	2.4	25.8	114.9
2008-2012	3.2	46.1	124.0	3.2	50.2	121.1	0.4	12.5	92.3	2.4	22.2	115.6

Secondary Medical Zone ID: 335

	Male						Female					
	Suicide			Suicide			Suicide			Suicide		
	Num per year	Rate /100,000	×100	Num per year	Rate /100,000	×100	Num per year	Rate /100,000	×100	Num per year	Rate /100,000	×100
	Total (>10 y/o)			45–54			Total (>10 y/o)			45–54		
1983–1987	41.0	39.7	132.6	9.6	66.1	130.8	24.2	17.8	124.4	3.6	20.3	117.9
1988–1992	32.8	28.3	126.5	6.6	45.4	132.1	22.2	14.5	120.9	3.6	19.2	131.1
1993–1997	39.2	31.3	138.6	7.6	45.0	123.0	21.2	12.5	127.9	2.4	14.2	110.1
1998–2002	51.2	40.7	123.6	11.6	63.5	113.5	23.8	14.0	122.7	4.6	20.6	136.9
2003–2007	55.8	43.4	132.5	14.6	80.6	144.0	23.8	14.1	127.4	3.8	17.8	130.1
2008–2012	43.0	35.8	110.8	9.6	59.1	121.6	17.8	12.8	103.0	2.8	16.3	109.4
	15–24			55–64			15–24			55–64		
1983–1987	2.2	16.3	116.9	6.6	47.6	111.9	1.4	8.4	123.3	4.6	24.0	121.8
1988–1992	0.6	7.5	75.3	7.2	45.2	127.4	0.0	3.2	61.0	5.8	26.6	148.7
1993–1997	1.6	10.8	95.2	7.8	50.7	125.9	0.8	5.3	102.2	6.2	28.5	179.4
1998–2002	2.0	14.0	88.9	10.4	73.4	115.6	0.4	5.4	76.3	3.6	21.2	112.5
2003–2007	1.6	14.6	83.4	12.0	72.1	124.9	0.4	6.2	69.1	3.8	19.8	118.5
2008–2012	1.4	16.8	79.1	12.2	61.2	126.0	0.4	6.9	70.8	1.2	11.1	74.5
	25–34			65–74			25–34			65–74		
1983–1987	5.6	34.2	133.7	5.2	53.9	123.1	3.4	16.7	154.9	4.0	32.4	103.6
1988–1992	2.0	16.8	87.7	6.2	52.0	147.5	2.0	11.1	127.9	3.6	25.1	96.1
1993–1997	4.4	27.9	142.7	5.4	40.4	126.7	0.8	7.3	88.2	5.0	27.0	138.8
1998–2002	3.0	24.6	91.2	9.2	60.8	133.8	2.0	12.7	110.0	4.2	23.4	111.5
2003–2007	4.6	32.6	105.1	9.8	64.8	151.0	1.8	12.9	99.3	4.4	23.7	127.5
2008–2012	3.4	29.4	90.3	4.8	38.9	98.5	2.2	15.2	106.1	3.2	19.7	110.9
	35–44			>74 y/o			35–44			>74 y/o		
1983–1987	7.0	43.1	135.5	4.6	89.7	114.5	1.8	12.2	103.3	5.4	67.3	116.6
1988–1992	4.4	25.6	107.8	5.6	83.1	128.8	2.4	11.9	122.9	4.8	45.2	94.4
1993–1997	5.6	31.1	127.0	6.4	78.8	151.3	1.2	7.9	93.3	4.8	35.3	102.7
1998–2002	7.4	45.2	128.3	7.4	75.7	143.7	1.6	10.3	99.7	7.4	42.1	135.7
2003–2007	6.2	43.8	109.4	6.8	58.1	129.0	2.0	13.1	103.7	7.4	34.5	153.9
2008–2012	5.0	36.8	99.0	6.6	49.3	118.9	2.4	14.8	109.2	5.6	23.5	122.2

Secondary Medical Zone ID: 336

	Male						Female					
	Suicide			Suicide			Suicide			Suicide		
	Num per year	Rate /100,000	×100	Num per year	Rate /100,000	×100	Num per year	Rate /100,000	×100	Num per year	Rate /100,000	×100
	Total (>10 y/o)			45–54			Total (>10 y/o)			45–54		
1983–1987	22.4	40.2	141.8	5.6	63.8	126.1	13.8	18.5	137.3	2.2	20.3	117.7
1988–1992	21.8	33.2	169.4	4.8	54.7	159.3	9.6	13.5	113.3	1.0	14.5	99.1
1993–1997	18.4	29.7	141.9	3.6	44.8	122.4	7.6	11.0	106.9	2.0	17.5	135.6
1998–2002	25.0	42.8	137.4	6.4	72.4	129.3	13.4	15.0	143.5	1.4	16.5	109.4
2003–2007	29.0	45.4	163.0	5.8	73.1	130.7	12.6	14.4	143.7	1.8	17.2	125.5
2008–2012	25.8	41.2	153.9	4.6	63.2	130.0	9.4	14.2	120.8	0.6	13.7	91.9
	15–24			55–64			15–24			55–64		
1983–1987	1.0	16.4	117.5	4.8	58.1	136.8	0.6	7.8	114.8	1.6	19.5	98.7
1988–1992	0.2	9.1	92.3	4.6	49.4	139.0	0.2	5.2	97.8	2.2	20.7	115.6
1993–1997	0.8	12.7	112.7	4.8	53.0	131.7	0.2	5.1	98.2	1.4	16.2	101.8
1998–2002	1.8	21.5	135.9	5.8	79.8	125.5	0.6	8.1	115.8	2.2	22.4	119.0
2003–2007	1.8	23.4	134.0	6.4	81.0	140.3	0.0	7.1	78.4	1.8	19.5	116.4
2008–2012	0.4	19.7	93.1	6.8	67.6	139.1	0.8	12.6	129.6	1.8	17.6	118.1
	25–34			65–74			25–34			65–74		
1983–1987	3.0	33.9	132.4	1.4	39.1	89.3	1.2	13.0	120.4	4.0	50.9	162.8
1988–1992	2.2	27.9	145.9	2.8	47.2	133.9	0.6	9.1	105.0	3.6	41.5	159.2
1993–1997	0.8	19.3	98.8	2.8	40.8	127.8	0.0	6.7	80.9	1.4	18.8	96.3
1998–2002	1.8	31.8	117.8	2.8	43.5	95.7	0.4	11.3	97.6	2.6	25.9	123.2
2003–2007	2.2	38.6	124.6	6.0	68.1	158.7	0.6	13.5	103.7	3.0	26.2	141.0
2008–2012	2.2	38.8	119.1	4.4	56.1	142.1	1.0	16.5	115.3	2.2	22.4	125.7
	35–44			>74 y/o			35–44			>74 y/o		
1983–1987	5.2	54.9	172.6	1.2	65.6	83.8	0.8	12.0	102.0	3.4	83.0	143.9
1988–1992	2.8	30.6	128.9	4.4	117.0	181.4	0.6	9.5	97.9	1.4	35.1	73.3
1993–1997	2.6	30.8	125.7	3.0	75.6	145.2	0.2	7.0	82.0	2.4	39.2	113.9
1998–2002	3.2	45.0	127.9	3.2	69.4	131.7	1.2	13.0	125.4	5.0	57.3	184.6
2003–2007	1.6	38.0	94.8	5.2	79.4	176.3	0.8	13.5	107.0	4.6	42.6	189.7
2008–2012	2.0	40.7	109.5	5.4	68.3	164.5	0.4	13.0	95.7	2.6	23.0	119.7

Secondary Medical Zone ID: 337

	Male						Female					
	Suicide			Suicide			Suicide			Suicide		
	Num per year	Rate /100,000	×100	Num per year	Rate /100,000	×100	Num per year	Rate /100,000	×100	Num per year	Rate /100,000	×100
	Total (>10 y/o)			45–54			Total (>10 y/o)			45–54		
1983–1987	33.8	39.3	131.4	7.2	56.4	111.5	16.6	15.8	106.9	3.4	20.7	120.0
1988–1992	24.8	28.2	122.4	6.6	51.7	150.4	16.2	13.6	112.6	2.0	15.5	105.9
1993–1997	26.8	29.2	126.7	5.0	41.2	112.5	11.4	10.9	96.5	3.2	18.7	144.5
1998–2002	36.6	41.0	121.3	9.4	68.3	122.1	10.2	11.5	82.0	1.6	14.4	95.6
2003–2007	39.6	43.7	131.8	11.2	82.2	147.0	11.8	11.9	96.5	1.4	13.0	94.7
2008–2012	30.4	35.5	112.2	6.8	57.7	118.8	11.2	12.6	96.9	1.4	14.2	95.2
	15–24			55–64			15–24			55–64		
1983–1987	1.8	17.6	126.0	5.6	46.8	110.1	0.6	6.7	97.6	4.0	24.6	124.9
1988–1992	1.2	11.2	113.3	4.4	34.9	98.3	0.4	5.0	95.4	3.8	22.1	123.9
1993–1997	0.2	8.6	75.8	6.4	49.1	122.0	0.2	4.4	83.8	2.6	17.5	110.2
1998–2002	2.0	18.4	116.6	6.4	63.2	99.4	0.6	7.0	99.9	1.4	16.0	84.8
2003–2007	1.8	19.3	110.5	7.8	67.1	116.3	0.6	8.5	94.4	1.6	15.8	94.4
2008–2012	0.8	18.1	85.1	8.4	59.1	121.6	0.4	8.5	87.9	1.8	14.9	99.7
	25–34			65–74			25–34			65–74		
1983–1987	4.8	34.8	136.1	5.6	66.6	152.1	1.2	10.4	97.1	3.2	32.2	103.2
1988–1992	2.6	23.2	121.4	3.4	39.7	112.7	0.8	8.2	95.1	2.8	24.9	95.4
1993–1997	3.4	29.0	148.3	4.6	41.3	129.6	0.8	8.4	100.5	2.2	17.8	91.2
1998–2002	3.0	31.0	114.9	5.6	48.0	105.6	0.6	10.0	86.4	2.6	19.7	93.7
2003–2007	2.8	32.2	103.9	7.2	58.6	136.6	0.4	10.1	78.0	3.2	21.6	116.2
2008–2012	1.8	28.4	87.2	4.6	45.5	115.2	1.2	14.4	100.3	1.6	16.4	92.1
	35–44			>74 y/o			35–44			>74 y/o		
1983–1987	5.0	39.7	124.7	3.8	91.2	116.4	1.4	12.0	101.9	2.8	46.1	79.9
1988–1992	2.6	21.9	92.2	4.0	79.3	122.9	1.6	10.9	112.7	4.8	55.7	116.3
1993–1997	4.6	32.6	133.0	2.6	50.0	96.1	1.0	8.5	99.5	1.4	17.8	51.6
1998–2002	6.2	49.3	139.9	3.8	56.7	107.5	1.0	9.9	96.0	2.4	21.5	69.3
2003–2007	4.8	49.0	122.2	4.0	46.3	102.9	0.6	10.5	82.8	4.0	25.5	113.6
2008–2012	2.2	33.1	88.9	5.8	51.3	123.7	1.2	13.6	100.0	3.6	20.2	105.0

Secondary Medical Zone ID: 338

	Male						Female					
	Suicide			Suicide			Suicide			Suicide		
	Num per year	Rate /100,000	×100	Num per year	Rate /100,000	×100	Num per year	Rate /100,000	×100	Num per year	Rate /100,000	×100
	Total (>10 y/o)			45–54			Total (>10 y/o)			45–54		
1983–1987	12.6	38.1	139.8	3.2	62.9	124.4	4.2	14.7	95.8	0.8	17.5	101.3
1988–1992	11.0	30.1	153.2	3.4	56.7	165.2	4.2	12.7	102.5	0.4	14.0	95.8
1993–1997	7.0	26.2	109.8	0.8	33.3	91.1	2.2	10.0	83.6	0.2	11.9	92.0
1998–2002	9.2	37.2	104.2	2.2	56.5	101.0	2.6	12.0	86.5	1.0	17.1	113.6
2003–2007	13.0	41.0	135.8	2.2	57.0	101.9	4.6	13.1	111.9	0.6	14.3	104.4
2008–2012	11.8	37.1	128.7	2.6	58.7	120.8	3.4	12.9	101.3	0.0	12.6	84.7
	15–24			55–64			15–24			55–64		
1983–1987	1.0	19.3	138.3	2.0	47.3	111.3	0.4	7.7	113.1	1.0	20.9	106.1
1988–1992	0.2	10.0	100.5	1.6	37.5	105.5	0.4	6.5	122.6	1.2	20.3	113.9
1993–1997	0.0	10.3	91.4	2.0	45.3	112.5	0.2	5.6	107.9	0.0	11.9	75.1
1998–2002	0.0	14.1	89.2	2.6	70.8	111.5	0.2	7.3	104.2	0.6	18.4	98.0
2003–2007	0.4	18.1	103.8	3.2	72.7	125.9	0.4	10.5	117.1	1.4	20.4	121.7
2008–2012	0.0	19.4	91.3	3.0	57.1	117.6	0.0	8.9	91.6	0.6	15.2	101.8
	25–34			65–74			25–34			65–74		
1983–1987	1.4	29.4	114.9	1.2	48.3	110.4	0.4	10.8	100.3	0.6	27.7	88.8
1988–1992	2.2	33.9	177.3	0.8	34.7	98.6	0.6	10.3	118.3	0.8	25.6	98.0
1993–1997	0.8	22.1	113.2	0.6	27.6	86.4	0.0	7.4	88.8	0.4	16.5	84.7
1998–2002	0.2	24.1	89.3	1.4	44.0	96.8	0.0	10.4	89.7	0.2	16.2	77.2
2003–2007	1.2	35.6	114.7	2.0	49.9	116.4	0.0	11.6	88.9	0.6	17.8	95.7
2008–2012	1.0	34.9	107.2	2.4	51.6	130.6	0.2	13.9	97.0	1.0	19.8	111.4
	35–44			>74 y/o			35–44			>74 y/o		
1983–1987	2.2	41.6	130.7	1.6	99.3	126.8	0.6	12.6	107.1	0.4	37.8	65.5
1988–1992	1.2	26.2	110.3	1.6	84.1	130.3	0.2	9.1	94.2	0.6	35.7	74.5
1993–1997	1.4	29.0	118.2	1.2	62.0	119.0	0.4	9.0	106.1	1.0	34.8	101.0
1998–2002	2.4	48.0	136.2	0.4	41.3	78.4	0.2	9.8	94.6	0.4	20.7	66.7
2003–2007	2.2	54.1	134.9	1.8	56.2	124.7	0.2	12.0	95.2	1.4	27.7	123.6
2008–2012	0.8	36.4	97.8	2.0	51.4	123.9	0.8	15.6	115.0	0.8	18.1	94.3

Secondary Medical Zone ID: 339

	Male						Female					
	Suicide			Suicide			Suicide			Suicide		
	Num per year	Rate /100,000	×100	Num per year	Rate /100,000	×100	Num per year	Rate /100,000	×100	Num per year	Rate /100,000	×100
	Total (>10 y/o)			45–54			Total (>10 y/o)			45–54		
1983–1987	26.0	38.3	127.6	6.6	65.6	129.7	10.2	14.0	85.0	1.4	16.3	94.3
1988–1992	25.0	32.4	153.1	3.0	36.6	106.5	10.6	12.9	95.3	0.8	13.3	90.8
1993–1997	23.6	31.4	142.4	5.0	47.7	130.5	7.4	10.3	85.6	0.4	10.6	82.2
1998–2002	31.4	43.3	134.5	8.2	71.7	128.2	9.2	12.7	93.2	1.8	16.7	111.0
2003–2007	34.8	44.8	150.0	5.8	56.1	100.3	8.2	12.5	93.8	1.8	15.7	114.8
2008–2012	29.8	41.5	138.7	7.8	71.0	146.0	8.0	12.9	97.5	1.0	14.2	95.2
	15–24			55–64			15–24			55–64		
1983–1987	2.4	22.8	163.4	4.0	45.2	106.3	0.4	6.4	94.1	2.6	21.8	110.5
1988–1992	1.8	14.7	148.1	5.0	47.9	135.0	1.0	7.7	145.8	2.4	20.1	112.5
1993–1997	1.0	12.8	113.5	5.0	51.5	128.0	0.4	5.5	106.3	0.6	12.0	75.5
1998–2002	1.2	17.4	110.4	8.6	94.4	148.5	0.2	6.3	89.2	1.8	20.2	107.4
2003–2007	1.4	20.4	116.7	9.6	94.8	164.2	0.2	7.8	86.5	2.0	19.2	115.0
2008–2012	0.2	17.7	83.5	8.2	66.7	137.4	0.2	8.8	90.4	0.4	11.9	79.6
	25–34			65–74			25–34			65–74		
1983–1987	3.6	30.1	117.6	2.6	46.0	105.0	1.4	11.9	110.1	1.4	23.0	73.5
1988–1992	3.2	29.3	153.1	3.0	42.2	119.7	1.8	12.6	145.9	1.6	20.6	78.9
1993–1997	4.0	36.2	185.5	3.4	40.7	127.7	0.8	9.2	111.0	1.4	16.2	83.1
1998–2002	2.2	30.6	113.1	4.2	50.2	110.4	0.8	11.9	102.6	1.6	18.5	88.0
2003–2007	2.6	36.4	117.2	6.8	71.0	165.4	0.4	11.3	87.2	1.0	15.7	84.1
2008–2012	3.4	42.5	130.5	2.2	37.3	94.4	0.8	14.4	100.5	1.8	20.0	112.6
	35–44			>74 y/o			35–44			>74 y/o		
1983–1987	4.6	42.8	134.4	2.2	71.5	91.2	1.6	14.1	119.4	1.4	28.6	49.6
1988–1992	5.6	40.7	171.7	3.4	78.5	121.7	1.0	10.0	103.3	1.8	27.6	57.7
1993–1997	3.4	29.6	120.7	1.8	43.9	84.3	1.8	12.3	144.3	2.0	24.4	70.8
1998–2002	4.8	46.3	131.6	2.2	45.5	86.4	1.4	12.5	121.1	1.6	18.3	58.8
2003–2007	4.0	49.2	122.8	4.4	59.6	132.4	1.2	13.9	109.7	1.6	15.0	66.9
2008–2012	4.0	46.1	123.9	3.8	47.6	114.7	1.6	16.3	120.0	2.2	17.2	89.5

Secondary Medical Zone ID: 340

	Male						Female					
	Suicide			Suicide			Suicide			Suicide		
	Num per year	Rate /100,000	×100	Num per year	Rate /100,000	×100	Num per year	Rate /100,000	×100	Num per year	Rate /100,000	×100
	Total (>10 y/o)			45–54			Total (>10 y/o)			45–54		
1983–1987	18.6	39.6	142.6	3.2	56.1	111.0	6.0	14.6	91.4	1.6	21.2	122.8
1988–1992	17.4	31.8	157.0	2.8	43.2	125.9	5.6	12.5	91.3	1.8	19.3	132.1
1993–1997	16.8	31.0	141.8	3.8	47.5	129.9	5.2	11.0	95.0	0.6	12.8	98.8
1998–2002	22.8	41.3	130.8	6.4	71.0	126.9	5.2	12.0	88.2	1.4	16.9	112.3
2003–2007	27.0	45.8	145.0	8.4	86.7	155.1	4.8	12.0	87.2	0.6	12.4	90.2
2008–2012	23.6	39.6	131.5	6.4	69.8	143.5	7.8	14.0	110.6	1.8	18.7	125.5
	15–24			55–64			15–24			55–64		
1983–1987	1.8	20.6	148.0	1.4	39.0	91.8	0.0	5.5	80.6	1.0	19.3	97.8
1988–1992	1.0	11.7	118.0	2.4	41.8	117.6	0.4	5.8	109.1	0.6	16.0	89.7
1993–1997	0.2	9.3	82.1	2.6	45.3	112.6	0.4	5.5	106.0	0.8	16.0	100.9
1998–2002	0.8	14.4	91.0	3.4	66.2	104.3	0.0	5.4	77.2	0.8	18.5	98.6
2003–2007	1.4	18.3	105.1	6.4	83.6	144.8	0.2	7.3	80.8	0.6	15.8	94.1
2008–2012	1.0	19.7	93.0	5.6	59.2	121.9	0.6	10.1	103.9	1.8	18.0	120.5
	25–34			65–74			25–34			65–74		
1983–1987	5.4	46.2	180.6	1.0	39.9	91.0	2.0	16.6	154.6	0.2	19.9	63.7
1988–1992	4.4	40.3	210.6	1.4	38.6	109.4	1.6	13.1	150.6	0.2	15.7	60.3
1993–1997	3.4	33.9	173.6	1.6	36.4	114.2	0.2	7.3	87.9	0.0	11.2	57.7
1998–2002	2.6	33.0	122.1	2.8	53.3	117.1	0.4	10.7	91.9	0.6	17.7	84.3
2003–2007	2.6	35.1	113.2	1.4	37.8	88.0	1.2	14.8	113.6	0.6	16.9	90.6
2008–2012	2.8	37.7	115.7	2.6	45.8	115.8	0.8	14.1	98.6	0.6	16.9	94.9
	35–44			>74 y/o			35–44			>74 y/o		
1983–1987	4.6	52.3	164.2	1.2	70.5	90.0	1.0	13.7	115.6	0.2	21.5	37.2
1988–1992	4.0	37.3	157.4	1.2	57.1	88.5	0.6	9.7	100.0	0.4	20.7	43.3
1993–1997	4.8	42.9	175.0	0.4	32.0	61.5	2.6	16.8	198.0	0.6	18.4	53.3
1998–2002	4.4	48.9	138.8	2.2	61.5	116.7	0.8	10.9	105.7	1.2	23.0	74.2
2003–2007	4.6	58.9	147.1	2.2	51.5	114.4	0.8	12.9	102.4	0.8	15.2	67.8
2008–2012	3.0	42.0	112.8	2.2	45.5	109.6	1.2	15.3	112.5	1.0	16.0	83.1

Secondary Medical Zone ID: 341

	Male						Female					
	Suicide			Suicide			Suicide			Suicide		
	Num per year	Rate /100,000	×100	Num per year	Rate /100,000	×100	Num per year	Rate /100,000	×100	Num per year	Rate /100,000	×100
	Total (>10 y/o)			45–54			Total (>10 y/o)			45–54		
1983–1987	62.0	41.4	143.8	13.4	59.0	116.7	18.4	13.4	83.8	4.2	18.1	105.3
1988–1992	59.2	34.8	160.1	12.0	50.2	146.2	19.4	11.9	88.8	3.0	14.0	95.6
1993–1997	69.2	36.5	160.5	12.8	48.2	131.7	16.8	9.9	82.4	3.4	13.5	104.8
1998–2002	94.8	47.9	141.2	25.4	80.0	142.9	25.0	12.8	95.0	5.8	18.1	120.3
2003–2007	98.4	47.0	134.3	27.2	82.5	147.6	23.8	12.0	88.4	6.0	17.4	126.7
2008–2012	90.4	40.5	123.6	23.2	71.5	147.1	26.8	12.7	93.4	6.4	19.2	128.5
	15–24			55–64			15–24			55–64		
1983–1987	9.0	26.2	187.5	6.0	48.5	114.0	1.8	6.5	95.2	2.8	19.1	96.6
1988–1992	5.4	14.0	141.2	8.4	45.6	128.5	1.6	5.1	96.8	2.4	14.8	83.0
1993–1997	3.0	9.6	85.1	14.8	63.4	157.6	1.2	4.2	80.9	3.8	16.8	105.7
1998–2002	6.0	17.7	111.9	17.4	78.4	123.4	2.0	6.7	95.2	3.4	16.8	89.4
2003–2007	4.0	14.6	83.7	18.6	74.0	128.2	2.0	7.5	83.7	4.2	17.1	102.3
2008–2012	4.6	17.5	82.3	17.4	56.1	115.4	1.6	6.9	71.5	5.0	16.3	109.0
	25–34			65–74			25–34			65–74		
1983–1987	16.8	47.7	186.7	2.0	36.4	83.2	4.4	13.6	126.6	1.4	19.8	63.4
1988–1992	14.2	42.7	223.3	2.8	35.2	100.0	4.2	12.4	143.0	2.0	19.2	73.8
1993–1997	12.6	36.6	187.5	4.8	40.4	126.8	3.2	9.7	116.8	1.0	10.8	55.5
1998–2002	14.6	40.4	149.6	6.4	42.9	94.3	3.6	11.1	96.2	2.8	17.3	82.1
2003–2007	15.0	42.0	135.5	9.6	49.0	114.3	3.0	10.2	78.8	2.4	14.0	75.0
2008–2012	10.8	34.5	105.9	10.0	48.5	122.7	4.4	14.1	98.8	2.4	14.1	79.2
	35–44			>74 y/o			35–44			>74 y/o		
1983–1987	12.6	46.9	147.2	2.2	64.3	82.1	3.2	12.8	108.6	0.6	15.4	26.7
1988–1992	14.0	41.6	175.6	2.4	52.6	81.5	4.8	13.7	141.7	1.4	17.8	37.3
1993–1997	17.4	49.1	200.2	3.4	54.1	104.0	3.2	9.7	114.6	0.8	10.2	29.6
1998–2002	23.0	68.3	193.8	1.8	32.2	61.1	5.2	14.7	142.6	2.2	16.4	52.8
2003–2007	20.0	61.1	152.5	4.0	41.0	90.9	4.6	13.9	110.1	1.6	10.5	46.7
2008–2012	17.2	47.3	127.0	7.0	47.0	113.2	5.0	14.1	104.1	2.0	10.6	55.3

Secondary Medical Zone ID: 342

	Male						Female					
	Suicide			Suicide			Suicide			Suicide		
	Num per year	Rate /100,000	×100	Num per year	Rate /100,000	×100	Num per year	Rate /100,000	×100	Num per year	Rate /100,000	×100
	Total (>10 y/o)			45–54			Total (>10 y/o)			45–54		
1983–1987	80.6	36.9	120.1	17.0	50.1	99.0	26.8	12.4	77.6	6.2	17.9	103.6
1988–1992	71.2	29.2	125.7	14.2	39.7	115.6	23.4	10.0	71.2	3.4	11.5	78.3
1993–1997	83.6	30.7	128.5	17.4	42.8	116.9	28.6	10.3	89.7	5.0	13.0	100.3
1998–2002	120.2	41.6	119.3	31.6	66.3	118.4	33.2	11.7	84.4	6.0	13.8	91.9
2003–2007	123.0	40.2	112.0	30.0	61.7	110.4	35.2	11.8	86.0	8.4	16.6	121.1
2008–2012	126.8	38.8	117.5	26.6	57.5	118.4	41.0	12.8	94.8	9.4	19.2	128.7
	15–24			55–64			15–24			55–64		
1983–1987	10.2	21.1	151.6	9.0	45.9	108.0	3.6	7.5	109.8	4.0	17.7	89.7
1988–1992	6.2	12.0	121.0	10.8	40.2	113.3	2.0	4.4	84.0	5.0	17.6	98.4
1993–1997	7.6	14.1	124.9	15.0	46.2	114.7	3.4	6.3	121.0	4.8	14.9	94.0
1998–2002	7.0	15.1	95.8	23.0	69.1	108.8	3.2	6.9	98.1	5.2	16.4	87.3
2003–2007	4.8	12.4	71.1	27.4	71.5	123.9	2.8	7.0	77.4	4.0	12.8	76.3
2008–2012	6.0	16.3	76.9	29.2	61.4	126.5	2.0	6.2	63.7	6.0	13.8	92.9
	25–34			65–74			25–34			65–74		
1983–1987	19.0	35.7	139.6	5.2	48.6	111.1	5.8	11.4	105.4	1.6	15.5	49.5
1988–1992	15.2	31.2	163.3	3.8	31.1	88.3	4.4	9.2	106.3	1.8	13.2	50.6
1993–1997	13.6	28.4	145.2	5.6	32.6	102.2	4.8	9.7	117.1	3.2	15.3	78.7
1998–2002	18.2	36.1	133.8	11.4	48.5	106.6	7.2	13.8	119.1	3.4	14.6	69.5
2003–2007	17.8	35.5	114.6	12.2	43.4	101.1	6.0	12.2	94.2	3.4	12.9	69.5
2008–2012	17.4	37.4	114.8	15.2	49.7	125.7	6.6	14.2	99.0	5.2	16.7	93.7
	35–44			>74 y/o			35–44			>74 y/o		
1983–1987	17.4	41.1	129.0	2.4	51.6	65.9	4.0	10.7	90.6	1.6	18.8	32.5
1988–1992	17.6	34.1	143.9	3.2	47.4	73.5	5.2	10.6	110.1	1.4	12.8	26.8
1993–1997	20.4	38.9	158.7	4.0	44.5	85.4	5.4	10.4	122.8	2.0	12.6	36.6
1998–2002	24.2	50.3	142.9	4.8	43.0	81.5	5.8	11.9	115.6	2.0	10.8	34.9
2003–2007	23.0	48.5	121.0	7.8	48.6	107.9	7.0	14.2	112.5	3.4	12.7	56.5
2008–2012	22.4	43.0	115.6	9.8	45.4	109.5	7.4	14.2	105.0	3.8	12.0	62.6

Secondary Medical Zone ID: 343

	Male						Female					
	Suicide			Suicide			Suicide			Suicide		
	Num per year	Rate /100,000	×100	Num per year	Rate /100,000	×100	Num per year	Rate /100,000	×100	Num per year	Rate /100,000	×100
	Total (>10 y/o)			45–54			Total (>10 y/o)			45–54		
1983–1987	7.8	32.8	104.2	1.2	44.4	87.8	1.2	12.7	63.6	0.0	14.0	81.3
1988–1992	8.0	26.9	124.0	1.0	34.1	99.3	1.2	11.0	65.5	0.2	13.7	93.6
1993–1997	5.8	24.9	97.3	1.4	38.4	104.9	1.6	9.8	76.6	0.0	11.2	86.9
1998–2002	12.6	39.4	124.5	2.0	53.5	95.7	2.0	11.7	80.5	0.4	14.4	95.6
2003–2007	9.8	36.4	103.2	2.4	54.5	97.4	1.0	11.3	73.1	0.0	11.2	81.4
2008–2012	11.8	36.8	120.0	3.0	57.9	119.2	2.6	12.4	92.2	0.4	14.0	94.0
	15–24			55–64			15–24			55–64		
1983–1987	0.6	14.9	106.5	1.4	44.1	103.8	0.0	5.8	85.4	0.2	16.4	82.9
1988–1992	0.8	12.1	122.3	1.2	35.6	100.3	0.2	5.5	104.0	0.0	14.1	79.2
1993–1997	0.0	9.8	86.6	1.0	37.6	93.5	0.4	6.2	119.3	0.2	13.9	87.6
1998–2002	1.2	19.7	125.0	1.2	56.0	88.1	0.0	6.2	88.2	0.2	17.2	91.4
2003–2007	0.2	16.1	92.3	2.4	64.1	111.0	0.2	8.9	98.9	0.2	15.5	92.4
2008–2012	0.2	19.6	92.5	3.6	60.9	125.4	0.0	8.5	87.5	0.8	15.9	106.4
	25–34			65–74			25–34			65–74		
1983–1987	1.6	28.6	112.1	0.8	43.6	99.5	0.4	10.3	95.4	0.2	23.8	76.2
1988–1992	1.4	24.9	130.2	0.6	33.7	95.6	0.4	9.0	104.0	0.2	19.3	74.0
1993–1997	0.2	16.7	85.3	0.8	31.8	99.6	0.2	8.1	97.8	0.2	15.2	78.1
1998–2002	1.8	33.9	125.6	1.2	43.9	96.5	0.4	11.9	103.0	0.4	18.6	88.6
2003–2007	1.0	31.5	101.6	1.6	47.0	109.6	0.2	12.1	92.8	0.0	14.8	79.4
2008–2012	1.0	33.0	101.4	1.4	43.1	109.0	0.4	14.2	99.1	0.4	17.5	98.4
	35–44			>74 y/o			35–44			>74 y/o		
1983–1987	1.2	33.3	104.7	1.0	83.1	106.1	0.2	11.1	94.2	0.2	30.2	52.4
1988–1992	1.6	28.8	121.5	1.4	78.3	121.4	0.2	9.0	93.3	0.0	21.1	44.0
1993–1997	1.4	27.2	110.8	1.0	56.3	108.2	0.2	7.8	91.4	0.4	23.2	67.5
1998–2002	3.6	55.3	157.0	1.6	65.1	123.5	0.4	10.4	100.1	0.2	17.7	57.1
2003–2007	0.8	33.6	83.9	1.4	50.5	112.1	0.2	11.7	92.4	0.2	13.6	60.5
2008–2012	1.2	37.4	100.5	1.4	44.6	107.4	0.2	12.7	93.5	0.4	15.2	79.0

Secondary Medical Zone ID: 344

	Male						Female					
	Suicide			Suicide			Suicide			Suicide		
	Num per year	Rate /100,000	×100	Num per year	Rate /100,000	×100	Num per year	Rate /100,000	×100	Num per year	Rate /100,000	×100
	Total (>10 y/o)			45–54			Total (>10 y/o)			45–54		
1983–1987	6.2	33.0	104.9	1.4	51.2	101.3	1.2	13.7	76.6	0.4	17.0	98.9
1988–1992	7.2	27.5	131.9	1.6	42.3	123.1	1.0	11.5	74.2	0.0	13.1	89.7
1993–1997	7.6	27.7	128.3	0.8	34.6	94.5	1.2	10.0	80.6	0.0	11.5	88.8
1998–2002	8.0	36.9	101.9	2.2	56.8	101.5	2.8	12.6	97.6	0.6	15.6	103.7
2003–2007	10.8	39.1	117.7	3.6	70.8	126.7	4.0	12.9	109.8	0.4	13.3	96.9
2008–2012	12.0	38.1	123.3	5.2	78.4	161.3	2.8	12.5	96.2	0.4	14.3	96.1
	15–24			55–64			15–24			55–64		
1983–1987	0.2	12.7	91.2	0.2	34.2	80.5	0.2	6.9	101.5	0.0	16.3	82.7
1988–1992	0.4	10.6	107.4	1.0	37.4	105.4	0.2	5.6	105.7	0.2	16.3	91.5
1993–1997	0.6	12.7	112.3	2.2	53.2	132.1	0.0	4.7	89.5	0.6	17.4	109.5
1998–2002	0.0	13.4	85.1	1.8	67.6	106.5	0.2	7.1	101.0	0.4	18.7	99.6
2003–2007	1.0	20.7	118.5	1.4	54.7	94.8	0.2	8.9	98.5	0.6	17.6	105.1
2008–2012	0.6	22.1	104.1	1.6	46.4	95.4	0.0	8.4	86.7	0.6	15.4	103.0
	25–34			65–74			25–34			65–74		
1983–1987	2.2	34.2	133.8	0.2	37.4	85.4	0.2	9.6	89.4	0.0	24.3	77.7
1988–1992	1.4	25.5	133.3	1.2	45.8	129.9	0.0	7.3	84.6	0.4	24.9	95.6
1993–1997	0.0	15.2	78.0	0.6	32.5	101.8	0.4	9.0	107.9	0.2	17.0	87.2
1998–2002	0.4	24.0	88.9	0.4	39.5	86.9	0.8	13.4	115.4	0.0	16.7	79.6
2003–2007	0.6	27.2	87.6	1.2	46.7	108.7	0.8	14.4	110.8	0.4	18.4	98.9
2008–2012	1.4	34.2	105.0	1.2	43.5	110.0	0.0	11.8	82.4	0.8	20.2	113.5
	35–44			>74 y/o			35–44			>74 y/o		
1983–1987	1.8	39.8	125.1	0.2	65.3	83.3	0.2	11.4	96.4	0.2	42.0	72.9
1988–1992	1.0	24.4	103.1	0.6	65.1	100.9	0.0	8.4	87.1	0.2	33.5	70.0
1993–1997	2.6	38.3	156.2	0.8	59.1	113.5	0.0	7.0	82.8	0.0	19.9	57.9
1998–2002	2.8	49.4	140.1	0.4	46.5	88.2	0.4	10.5	102.0	0.4	25.6	82.6
2003–2007	1.8	44.8	111.7	1.2	53.9	119.7	0.4	12.5	99.2	1.2	31.0	138.0
2008–2012	1.2	36.2	97.2	0.8	42.0	101.3	0.8	14.8	109.4	0.2	15.2	79.2
